# Disease Gene Interaction Pathways: A Potential Framework for How Disease Genes Associate by Disease-Risk Modules

**DOI:** 10.1371/journal.pone.0024495

**Published:** 2011-09-06

**Authors:** Lina Chen, Wan Li, Liangcai Zhang, Hong Wang, Weiming He, Jingxie Tai, Xu Li, Xia Li

**Affiliations:** 1 College of Bioinformatics Science and Technology, Harbin Medical University, Harbin, Hei Longjiang Province, China; 2 Institute of Opto-Electronics, Harbin Institute of Technology, Harbin, Hei Longjiang Province, China; University of Colorado Denver, United States of America

## Abstract

**Background:**

Disease genes that interact cooperatively play crucial roles in the process of complex diseases, yet how to analyze and represent their associations is still an open problem. Traditional methods have failed to represent direct biological evidences that disease genes associate with each other in the pathogenesis of complex diseases. Molecular networks, assumed as ‘a form of biological systems’, consist of a set of interacting biological modules (functional modules or pathways) and this notion could provide a promising insight into deciphering this topic.

**Methodology/Principal Findings:**

In this paper, we hypothesized that disease genes might associate by virtue of the associations between biological modules in molecular networks. Then we introduced a novel disease gene interaction pathway representation and analysis paradigm, and managed to identify the disease gene interaction pathway for 61 known disease genes of coronary artery disease (CAD), which contained 46 disease-risk modules and 182 interaction relationships. As demonstrated, disease genes associate through prescribed communication protocols of common biological functions and pathways.

**Conclusions/Significance:**

Our analysis was proved to be coincident with our primary hypothesis that disease genes of complex diseases interact with their neighbors in a cooperative manner, associate with each other through shared biological functions and pathways of disease-risk modules, and finally cause dysfunctions of a series of biological processes in molecular networks. We hope our paradigm could be a promising method to identify disease gene interaction pathways for other types of complex diseases, affording additional clues in the pathogenesis of complex diseases.

## Introduction

Complex diseases are caused by disease risk genes in the form of biological modules or pathways in molecular networks [1], [2], [3]. A major challenge of the post-genomic era is to find disease-risk genes, identify their functions, and develop new techniques to uncover disease pathways [4]. The complexity of these diseases can be interpreted by their multiple gene products and the cooperative behavior of specific disease-risk modules or pathways in molecular networks. Many studies have demonstrated that targeting disease-associated functions or pathways provides additional insights into the mechanisms of disease [5], [6], which is essential to developing disease treatments.

Many biological pathways have been derived experimentally [7], [8]. Similarly, most disease pathways, *e.g.* the Alzheimer’s disease pathway, have been determined from experiments [9], [10]. However, *in vivo* methods are too time-consuming and laborious for discovering a large number of pathways. With the development of genomics, functional proteomics and metabolomics, many computational algorithms have been generated to identify biological modules or pathways in the context of biological molecular networks. For example, literature searches are used to discover signal transduction pathways [11]. Metabolic networks [12], [13], [14], [15] and gene interaction networks[16] are also employed to detect pathways. Protein-protein interaction networks (PPINs) are often used for pathway extractions [17], [18], [19], [20], [21]. Several online tools or software have been developed to discover biological pathways, such as PathFinder [22], BowTieBuilder [23], FASPAD [24], [25] and Pandora [26]. With the accumulation of high-throughput datasets, other computational algorithms have been developed to detect disease-related gene modules or dysfunctional pathways based on the literature or global characteristics of the interactome coupled with gene expression data [1], [11]. Although these methods have effectively identified some disease-risk modules or dysfunctional pathways, it is not clear how biological modules control each other in a cooperative manner and lead to the dysfunctions of multiple biological processes. Most disease genes and proteins of complex diseases are scattered in networks without direct interactions [27], [28] and crucial biological associations might be missed during the manipulation of these algorithms.

Previous studies demonstrated that neighborhood similarity is an effective measurement for evaluating the likelihood of two proteins in a network sharing a number of interacting neighbors [29], [30]. Proteins with higher neighborhood similarity may tend to have common or related biological functions. Li et al. used the neighborhood similarity to predict functional associations of proteins [31], while Li et al. introduced this concept to identify brain cancer-related genes [32]. In addition, Rives et al. and Sales-Pardo et al. found that biological networks have the characteristics of hierarchical structures based on measurements of neighborhood similarity [33], [34]. Once disease genes are identified, neighborhood similarity can be a promising metric that provides the basis for further studying disease genes association mechanisms in a hierarchically structured network.

PPIN is an intuitive form of a set of interacting biological modules. The underlying rationale is that disease genes might associate by virtue of associations between biological modules in molecular networks. In this study, we introduced a novel disease gene interaction pathway representation and analysis paradigm to demonstrate disease gene association mechanisms. First, we clustered proteins using hierarchical clustering method, constructed a hierarchical tree of biological modules according to their neighborhood similarities in a PPIN and obtained different lossless network representations. Then, we searched for disease-risk modules that contained disease proteins and other proteins with similar functions, and linked these modules to form disease gene interaction pathways. This connected disease genes that did not interact directly in the network. We applied this algorithm to extract disease gene interaction pathways of coronary artery disease (CAD), hypertension (HT), and type 2 diabetes (T2D), and evaluated our results by functional annotations, pathway-wide analyses and randomization tests.

## Materials and Methods

### Data

Human protein-protein interaction data were derived from Human Protein Reference Database (HPRD, Release 7, http://www.hprd.org/) [35], which contained 9463 proteins and 37,107 interactions. A PPIN was obtained from the data, in which nodes were proteins, and edges were interactions between the proteins. The largest connected component of the PPIN, which was composed of 9048 proteins with 36,755 pairwise interactions, was used for further analysis.

Disease genes for CAD, HT and T2D were the union of disease genes in Disease Ontology (DO, version 0.8, http://www.obofoundry.org/cgi-bin/detail.cgi?id=disease_ontology) [36], Online Mendelian Inheritance in Man (OMIM, May 2009, http://www.ncbi.nlm.nih.gov/entrez/query.fcgi?db=OMIM) [37] and the Genetic Association Database (GAD, October 1 2007 update, http://geneticassociationdb.nih.gov/) [38]. After mapping to the largest connected component of PPIN, we obtained 62 CAD, 122 HT, and 164 T2D genes.

### Methods

We used the neighborhood similarity to hierarchically cluster proteins in a PPIN into biological modules with similar functions, and constructed a hierarchical tree for all biological modules using a bottom-up approach. We then marked biological modules that contained disease genes or proteins as disease-risk modules in each hierarchy until disease-risk modules in one hierarchy could be linked together. This gave our final disease gene interaction pathway. In other words, these modules can connect disease genes that did not interact with each other directly, uncovering a possible potential framework for how scattered disease genes associated with each other by disease-risk modules.

#### Hierarchical tree construction

Neighborhood similarity measurement was used to cluster proteins into biological modules. Here, the measurement was the Jaccard index of two protein sets [39], [40] (other neighborhood similarity measures were alternates). This was always between 0 and 1; 0 if the sets had no common neighbors, and 1 if their neighbors were identical. The hierarchical tree was constructed in a bottom-up way based on the maximal Jaccard index value of protein sets from primary protein interactions. Thus, each protein started as a single module in the 1st hierarchy, and proteins or modules were merged as they moved up the hierarchy, as shown in *Algorithm 1*.

In *Algorithm 1* and *2*, 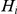 is the 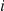
th hierarchy, which contains the protein set 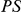, the module set 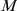 and their interaction relationships; 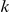 is the hierarchy index; 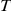 is a temporary module set; and 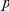 is a protein; and 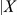 denotes the number of elements (proteins or modules) 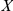 contains.

Algorithm 1 is shown here:

**input**: all proteins 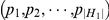 in 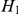


**output**: 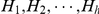


**cluster** (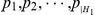):

*Initialize the hierarchy index* 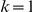;

*Initialize the protein set* 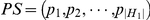 *and the module set* 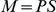;

*Initialize module interactions in the hierarchy* 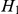;

*output* 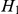;

*while* 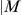 *( >1)*


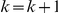
 *; // raise to a higher hierarchy*

*compute neighborhood similarity values between every module pairs in* 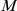;

*choose module pairs with the maximum value of neighborhood similarity;*

*merge modules into* 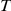;

*discard modules from* 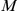 *and add* 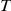 *to* 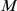;

*calculate number of all the modules in current hierarchy;*

*discard interactions between modules in* 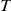;

*search for new module interactions according to module interactions in* 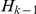;

*output* 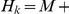 *module interactions;*

**Figure 1:**
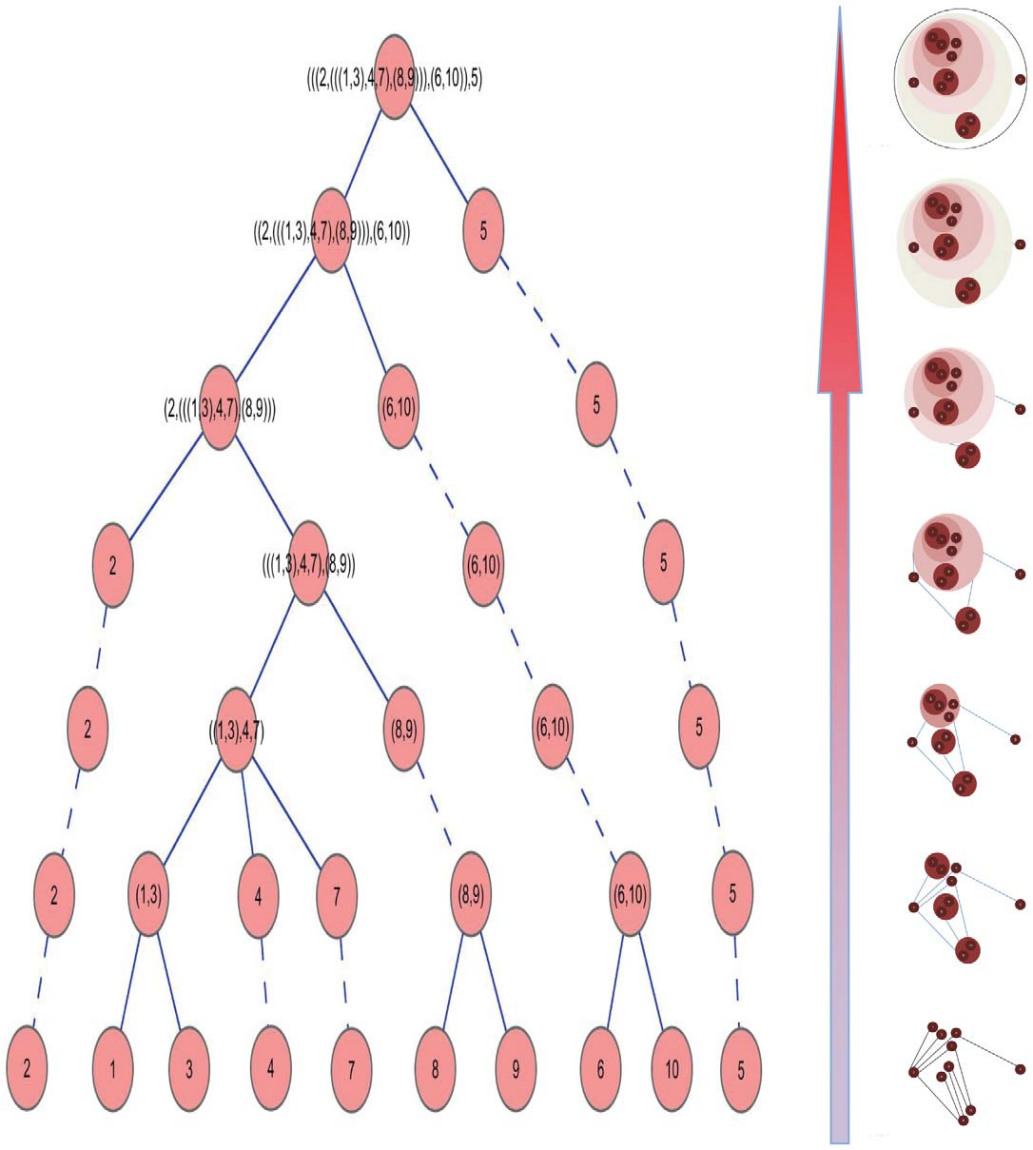
The hierarchical tree and its corresponding module representations of a PPIN. The bottom figure on the right is the initial PPIN, which is composed of 10 proteins. The left figure is the hierarchical tree, in which the solid lines mean the descendant modules or proteins are clustered into the ancestor modules, and the dashed lines mean the ancestor modules are identical to the descendant modules or proteins. And the right figures are the corresponding clustering results of each hierarchy in the left.

As an example, a sample PPIN composed of 10 proteins (1, 2, …, 10) was clustered hierarchically with *Algorithm 1* and the resulting hierarchical tree and interacting modules in each hierarchy are in Figure 1.

#### Searching disease gene interaction pathways

We first marked disease genes and disease-risk modules that contained disease proteins in the hierarchical tree. Then, the disease gene interaction pathway was searched according to interaction relationships between disease-risk modules in each hierarchy of the hierarchical tree using a bottom-up approach. If a pair of proteins in two modules interacted with each other, the two modules interacted. The process is illustrated in *Algorithm 2*.

In *Algorithm 2*, 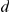 is a disease protein or disease-risk module, 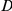 denotes the disease protein set, 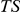 is the disease-risk module set, and 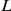 is the disease gene interaction pathway.

Algorithm 2 is shown here:

**input**: all disease proteins in the protein set 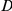, 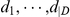 (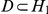)

**output**: disease gene interaction pathway 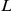


**searchPath** (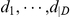):

*initialize disease gene interaction pathway* 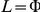;

*initialize the hierarchy index* 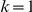;

*LABEL:*

*mark disease-risk module set* 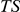 *using disease genes;*

*detect module interactions between disease-risk modules in* 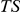;

*construct* 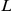 *using* 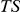 *and the module interactions;*

*if all disease-risk modules are not obtainable in* 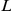




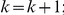



*goto LABEL;*

*else*

**Figure 2:**
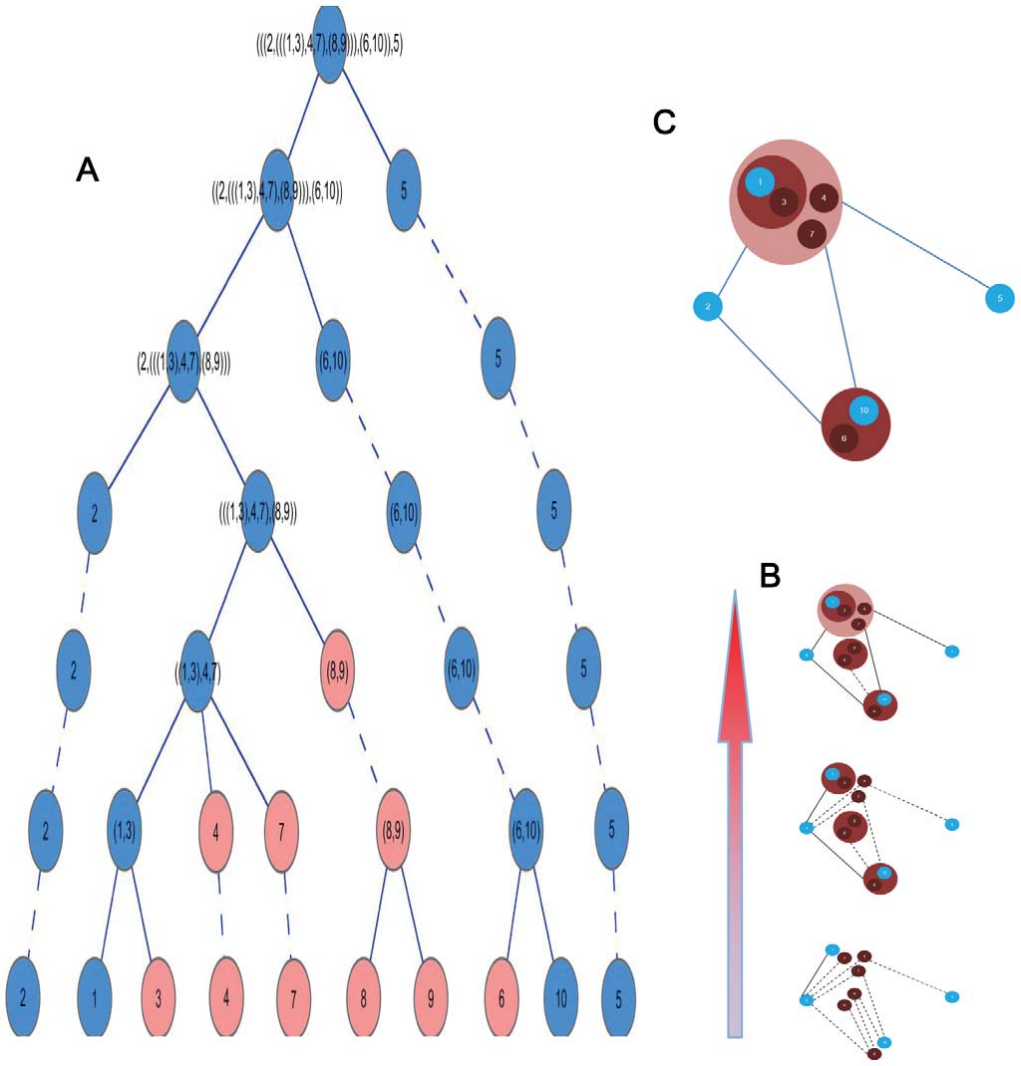
Sample process of searching disease gene interaction pathway using the hierarchical tree construction algorithm. (A) The hierarchical tree in Figure 1 with marked disease-risk modules, which are denoted by blue nodes. (B) The corresponding clustering results of each hierarchy in Figure 2A. The solid lines are the path that is searched out using Algorithm 2, and the dashed lines are the interaction relationships. (C) The resulting disease gene interaction pathway of sample PPIN. The blue nodes are disease proteins.

*output* 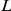;

As an example, for four disease gene products, labeled 1, 2, 5, and 10 in the sample PPIN in Figure 1, we used *Algorithm 2* to search for the disease gene interaction pathway for the sample network (Figure 2A and 2B). The resulting disease gene interaction pathway is shown in Figure 2C.

#### Evaluation

We generated 100 random networks, keeping the degree of each protein and rewiring the PPIN. The same processes for hierarchical tree construction and disease gene interaction pathway searching were performed on these random networks using the same disease genes for CAD, HT and T2D. We evaluated the performance of our method by comparing proteins and interactions of disease gene interaction pathways from random networks with those from HPRD PPIN.

## Results

In this paper, based on the neighborhood similarities, we represented a primary PPIN as a hierarchical tree of biological modules generated in a bottom-up way. The disease gene interaction pathway for CAD was derived according to our proposed algorithms. This disease gene interaction pathway contained 46 disease-risk modules and 182 interaction relationships between these modules. The results of disease gene interaction pathways for HT and T2D are shown in Figure S1 and S2. After further biological analysis, the effectiveness of the disease gene interaction pathway was evaluated and validated by two separate steps: *i*) comparing with random networks; and *ii*) validating of disease-risk modules and their interaction relationships.

### The hierarchical tree

We constructed a hierarchical tree using a bottom-up approach based on the neighborhood similarity of every two proteins or modules in the largest connected component of the PPIN (see Hierarchical tree construction in Methods). Proteins with large neighborhood similarities were organized into modules in lower hierarchies of the hierarchical tree, which were grouped into modules in higher hierarchies until all proteins are clustered into one module.

We obtained a hierarchical tree with 86 hierarchies using *Algorithm 1*. Each hierarchy was a different presentation of the largest connected component of the PPIN, and clustered the proteins into modules of various sizes. Every module comprised two or more submodules or proteins.

To evaluate the function consistency of each module, we used the online toolkit, Functional Annotation Tool in Database for Annotation, Visualization and Integrated Discovery (DAVID) Bioinformatics Resources 6.7 (http://david.abcc.ncifcrf.gov/) [41], [42], selecting the standard annotation categories: biological process (BP), cellular component (CC) and molecular function (MF), and the significance threshold p-value 0.05. We found that proteins in each of the modules had significant characteristics of sharing common functions in functional annotation categories, some of which are in Table S1.

**Figure 3:**
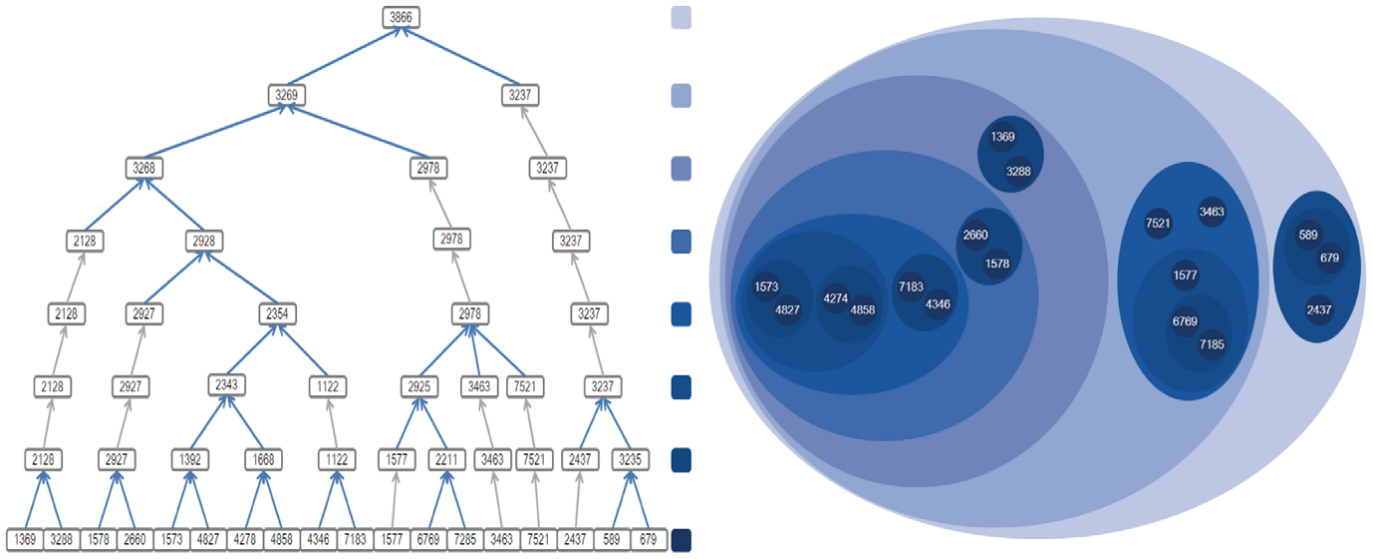
The clustering process of Module ‘3866’. The left figure is the hierarchical tree, while the right figure is the corresponding clustering result. Different colors correspond to different hierarchies. The labels in the bottom of the left figure and in the right figure are HPRD protein IDs, and the other labels are module IDs. All of Module ‘3866’ and its sub-modules ‘3237’ and ‘3269’, which contain 3 and 15 proteins separately, have function enrichment in GO functions with 100%, such as ‘regulation of transcription, DNA-dependent’, ‘regulation of transcription’, ‘transcription’, ‘regulation of cellular metabolic process’, ‘RNA metabolic process’, ‘transcription, DNA-dependent’, ‘RNA biosynthetic process’ in BP and ‘ligand-dependent nuclear receptor activity’, ‘sequence-specific DNA binding’, ‘transcription factor activity’, ‘steroid hormone receptor activity’ in MF. All the modules yielded in the clustering process have the same property.

**Figure 4:**
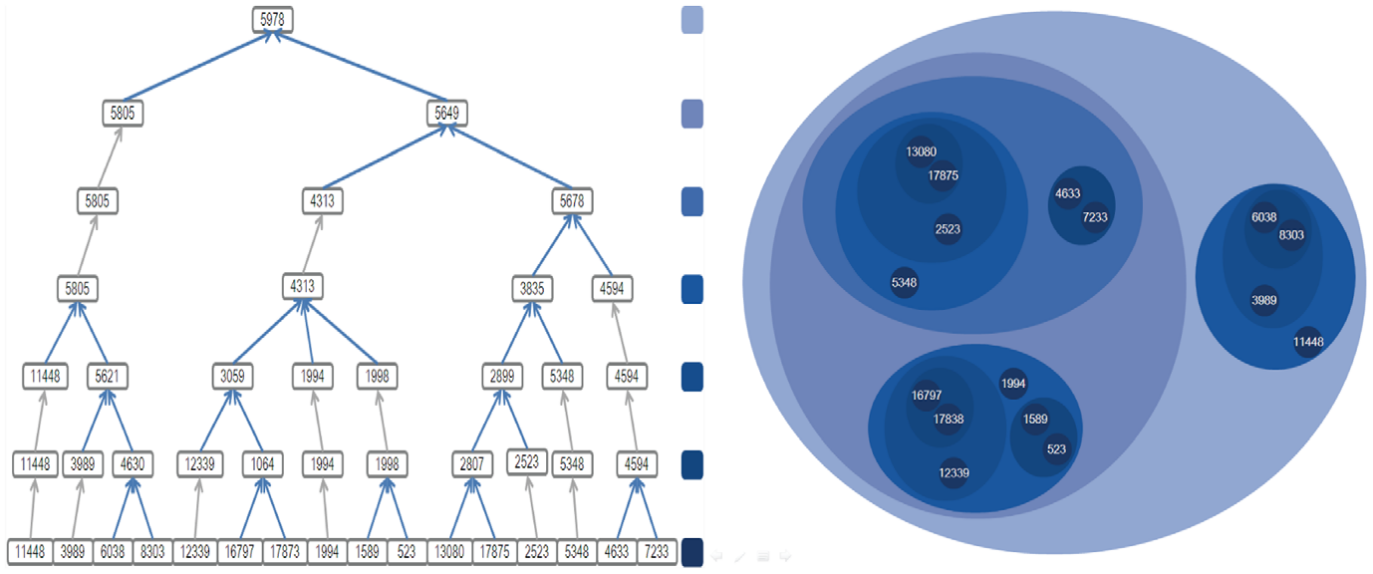
The clustering process of Module ‘5978’. The left figure is the hierarchical tree, while the right figure is the corresponding clustering result. Different colors correspond to different hierarchies. The labels in the bottom of the left figure and in the right figure are HPRD protein IDs, and the other labels are module IDs. Both Module ‘5978’ and its sub-module ‘5649’, which contains 12 proteins, have function enrichment in GO functions with 75%, such as ‘positive regulation of cellular process’ in BP, ‘NuRD complex’, ‘nucleus’, ‘histone deacetylase complex’ in CC and ‘hydro-lyase activity’, ‘carbon-oxygen lyase activity’ in MF. All the modules yielded in the clustering process have the same property.

We note that the entire hierarchical tree not only reconstructed the PPIN into different representations, but also associated biological modules through functional similarities of ancestor and descendant modules. Then, with the enriched functions for each module, we considered the functional stability of the ancestor and descendant modules. By comparison, some functions of modules were consistent with those of its submodules in lower hierarchies. In other words, modules may share most functions with their submodules (see Samples in Figure 3 and 4). Therefore, modules with disease genes were denoted as disease-risk modules. This encouraging characteristic that ancestor modules shared biological functions with descendant modules, might contribute to the further identification of disease gene interaction pathways for CAD, HT and T2D.

### Disease gene interaction pathway

**Figure 5:**
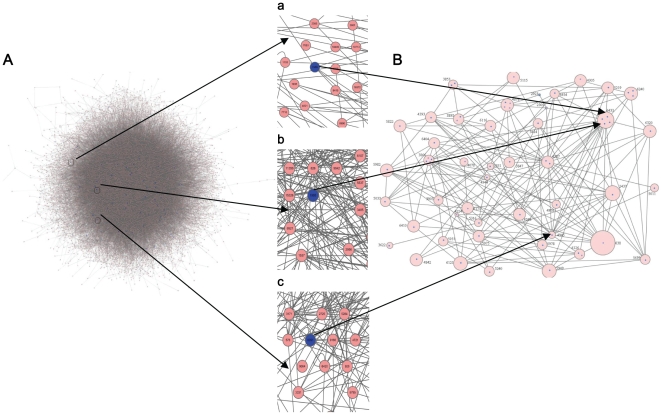
The initial PPIN and the resulting disease gene interaction pathway. (A) The PPIN of the largest connected component in HPRD. The nodes are proteins, in which the blue ones are disease proteins, and the edges are interactions between proteins. 27 (44.3%) disease proteins have 23 direct interactions. a, b and c are 3 enlarged part of Figure 5A containing CAD disease proteins 1989, 1993 and 6091 that do not interact directly respectively. (B) The resulting CAD disease gene interaction pathway derived from the PPIN by our method. 46 nodes in pink are disease-risk modules that contain CAD disease proteins (blue dots) and other proteins with similar functions, and the labels beside the nodes are their module IDs. The sizes of the nodes are directly proportional to the log number of proteins (2∼866, of which 1∼4 are disease proteins) they contain. 182 edges are the interaction relationships between disease-risk modules they connect. In the disease gene interaction pathway, 1989 and 1993 are both in disease-risk module 6433 of Figure 5B, and participate in CAD disease gene interaction pathway jointly. 1993 and 6091 locate in disease-risk module 6433 and 4945 of Figure 5B separately, which can interact with each other, and can be linked by these interacting disease-risk modules.

We marked disease-risk modules in the hierarchical tree according to 61 CAD genes or proteins (see Data section). Then, we used the proposed pathway searching algorithm (see Searching disease gene interaction pathway in Methods section) to search for a CAD disease gene interaction pathway. In the pathway, CAD disease genes associated by the mechanism that disease-risk modules interacted with each other. This led to multiple dysfunctions of biological processes in CAD pathogenesis. Finally, we derived a CAD disease gene interaction pathway containing 46 disease-risk modules and 182 interaction relationships. This data arrangement is in Figure 5.

**Figure 6:**
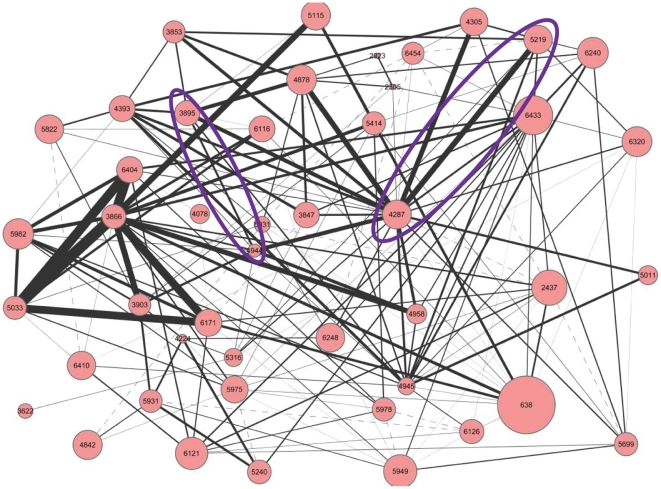
Interacting modules sharing common GO functions in the disease gene interaction pathway. The nodes are modules, and the edges are their interaction relationships. The solid edges denote two modules connected share common functions, while the dashed ones indicate no common functions, and the width of the lines are directly proportional to the number of shared functions (1∼92) of two modules. Two purple circles denote function sharing between disease gene pair *VWF* and *F12* of interacting disease-risk modules “3895” and “4944”, and gene pair *COL3A1* and *SERPINE1* of interacting disease-risk modules “4287” and “5219”.

To evaluate the interaction relationships between disease-risk modules, we examined enriched functions for each pair of 182 interacting disease-risk modules. According to results from enriched Gene Ontology (GO, http://www.geneontology.org/) functions [43], we found that 167 (91.8%) interaction relationships in the disease gene interaction pathway for CAD shared at least one common function (Figure 6 and Table S2), which suggested that disease genes associated by virtue of interacting disease-risk modules with shared functions, leading to multiple dysfunctions of biological processes in the pathogenesis of complex diseases. For example, disease gene pairs *VWF* (geneID: 7450) and *F12* (geneID: 2161), and *COL3A1* (geneID: 1281) and *SERPINE1* (geneID: 5054) are scattered in human PPIN. Notably, we found genes *VWF* and *F12* associated *via* the shared function “blood coagulation” for interacting disease-risk modules “3895” and “4944”, while genes *COL3A1* and *SERPINE1* associated through the “fibrinolysis” process shared by interacting disease-risk modules “4287” and “5219” in the resulting pathway we derived (purple circles in Figure 6). Many studies have reported that the biological processes of blood coagulation and fibrinolysis are significantly correlated with CAD pathogenesis [44], [45], [46], [47].

The disease gene interaction pathways for HT and T2D are in Figure S1 and S2.

### Evaluation

**Figure 7:**
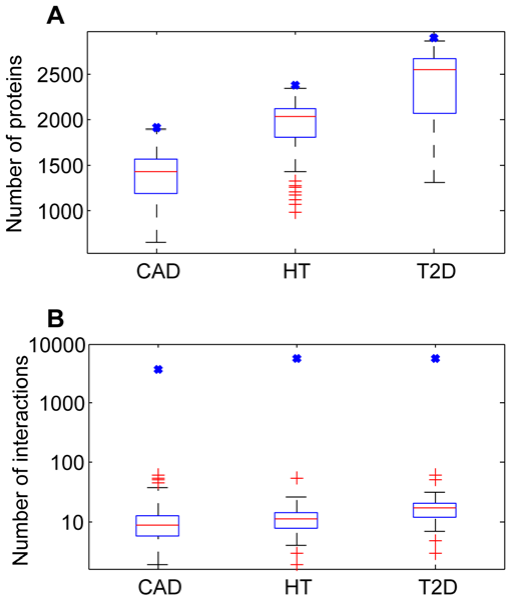
Network overlaps of resulting disease gene interaction pathways derived from random and HPRD networks. Boxes are the distribution of the number of proteins (A) and the distribution of the number of interactions (B). Blue crosses are the number for disease gene interaction pathways from HPRD PPIN, which are larger than those for random networks.

Using the same disease genes for CAD, HT, and T2D, we performed similar procedures on 100 random networks to search for disease gene interaction pathways. We compared the proteins and interactions of disease gene interaction pathways from random networks with those from HPRD PPIN. Only some of the proteins and interactions of the disease gene interaction pathway from HPRD PPIN could be found in pathways from random networks (Figure 7). These results illustrated that the disease gene interaction pathway could not be obtained from random networks, demonstrating the effectiveness of our method.

We also compared network metrics, *i.e.* the number of vertices, diameters, characteristic path lengths, and clustering coefficients for disease gene interaction pathways for the three diseases from the PPIN of HPRD and from random networks (Figure S3) to demonstrate the effectiveness of our method.

### Validation

To verify the associations between the resulting disease gene interaction pathway and CAD, we tested for cross-validations of CAD in disease-risk modules under the framework of Kyoto Encyclopedia of Genes and Genomes (KEGG, http://www.genome.jp/kegg/) pathways [48], [49], [50], and examined the resulting interaction relationships using online research literature on CAD.

**Figure 8:**
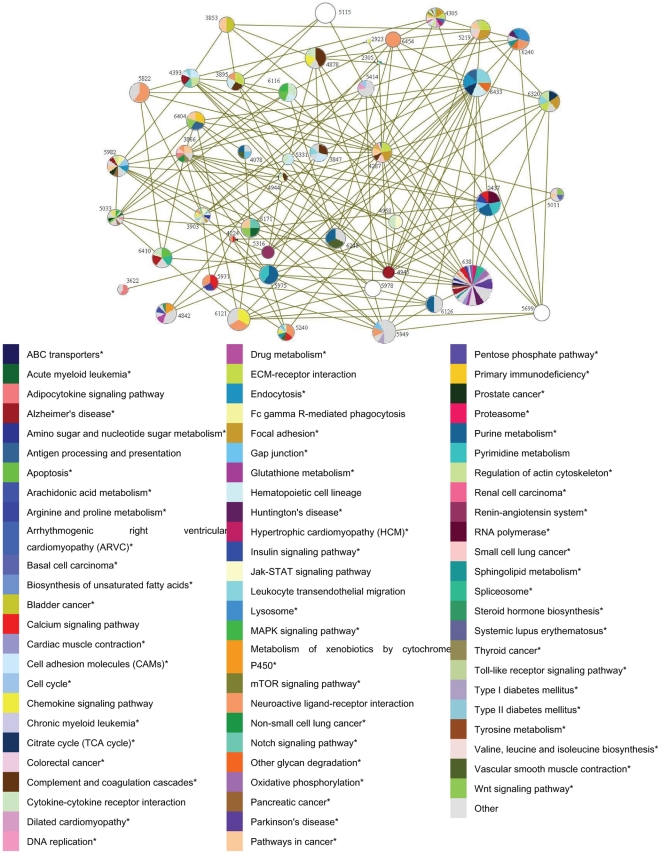
KEGG pathway enrichment results of disease-risk modules in the disease gene interaction pathway. The nodes are disease-risk modules, and the edges are the interactions between disease-risk modules. Different colors of each disease-risk modules represent different pathways, the sizes of which are proportional to the number of proteins or genes in the pathway enriched. 3 white nodes have no pathway enrichment. The pathways indicated by ‘*’ are CAD related, which are validated by literature retrieving.

Using DAVID online toolkits, we found that 46 disease-risk modules in the CAD disease gene interaction pathway were significantly correlated with 123 biological pathways (Figure 8). Of these, 43 (93.5%) were enriched in pathways including “Apoptosis”, “Alzheimer's disease”, “Arrhythmogenic right ventricular cardiomyopathy (ARVC)”, “Dilated cardiomyopathy”, “Other glycan degradation”. Note that only three disease-risk modules had no pathway enrichment. However, their annotated pathways were consistent with pathways enriched in their interacting disease-risk modules, which were important in disease gene association through interaction relationships. For example, disease-risk module 5115, with no significantly enriched pathways, had several genes annotated onto CAD-associated pathways such as “PPAR signaling pathway”, “Pathways in cancer”, and the “Non-small cell lung cancer” pathway. Furthermore, these pathways were enriched in the interacting disease-risk module 3866, which suggested biological associations between these two interacting disease-risk modules.

Of all enriched pathways, 61 were validated to be CAD-related by a literature retrieval (Table S3). For example, the “Apoptosis” pathway by which a cell is directed to a programmed death, has been shown to be correlated with CAD in previous studies [51], [52], [53], [54]. These indicated that CAD was caused by apoptosis (especially cardiomyocyte apoptosis)-inducing factors, such as angiotensin-converting enzyme inhibitor, low-density lipoprotein cholesterol, lyso-phosphatidylcholine and oxidized nonesterified fatty acids. Evidence indicated that the Alzheimer's disease (AD) pathway is also related to CAD because some factors that induced AD could also be CAD risk factors, *e.g.* the variants of apolipoprotein E (especially Allele epsilon4), apolipoprotein B, altered cholesterol levels, particularly high levels of low-density lipoproteins together with low levels of high-density lipoproteins [55], [56], [57], [58]. Some studies have shown that the “Other glycan degradation” pathway is related to CAD. Pro-angiogenic effects of perlecan involved in the pathway may be used to treat various ischemic diseases such as intractable CAD and peripheral vascular disease [59]. Carbohydrate that is malabsorbed and fermented in the colon, which is known as glycan degradation, increases CAD associated risk factors [60].

**Figure 9:**
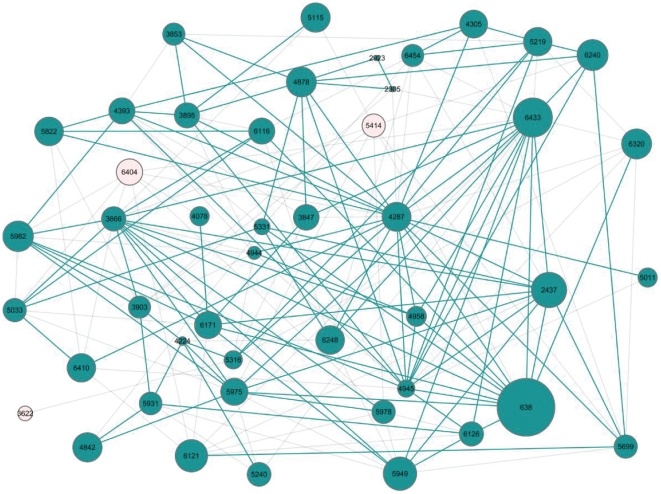
Interacting disease-risk modules supported by literature evidences. The nodes are disease-risk modules, and the edges are the interaction relationships between disease-risk modules. The green nodes and edges indicate that gene pairs between these interacting disease-risk modules are related to CAD after literature search.

The CAD disease gene interaction pathway covered 182 interaction relationships between 46 disease-risk modules. To further evaluate the reliability of interaction relationships between disease-risk modules, we used the NCBI PubMed module to retrieve correlations between gene pairs and CAD with the term “GENE symbol 1+GENE symbol 2+coronary artery disease” (e.g. *IL1R2*+*ESR2*+coronary artery disease). Verified were 107 interactions (58.8%) related to the pathogenesis of CAD (Figure 9 and Table S4). For example, gene pairs *IL1R2* (GeneID: 7850) and *ESR2* (GeneID: 2100) in interacting modules 6116 and 5033 and *IL1R2* and *PLA2G7* (GeneID: 7941) in interacting modules 6116 and 638 were related to CAD by [61]. Willer et al. identified the relationships between CAD and genes *MVK* (GeneID: 4598), *LDLR* (GeneID: 300438) and *APOA1* (GeneID: 335) in disease-risk modules 638, 4945, and 6433 that interact with each other [62]. McCarthy et al. validated that CAD is associated with gene pairs *LRP1* (GeneID: 4035) and *MTHFR* (GeneID: 4524) in interacting disease-risk modules 4945 and 638, and *LRP1* and *SELP* (Gene ID: 6403) in interacting modules 4945 and 4393 [63]. They also detected connections between CAD and genes *LDLR*, *SELP*, and *IL6* (GeneID: 3569) in disease-risk modules 4945, 4393, and 5982, which interact with each other [64]. The relationship between CAD and gene pair *TMEM57* (GeneID: 55219) and *CTCF* (GeneID: 10664) in interacting disease-risk modules 638 and 6433 is also recognized [65]. Genotype information has shown a relationship between CAD and gene pair *LDLR* and *APOA1* in interacting disease-risk modules 4945 and 6433 [66].

Detailed results for literature validation of modules and interaction relationships for HT and T2D disease gene interaction pathways are in Table S5, S6, S7, S8.

Based on our analysis, we concluded that both disease-risk modules and their interaction relationships were verified as associated with the pathogenesis of CAD, HT, and T2D. This demonstrated the effectiveness of our hypothesis that disease-risk modules can associate with each other in proposed disease gene interaction pathways. Furthermore, we must note that disease-risk module associations without significant evidence in the literature still need to be verified by further studies.

## Discussion

The rapid accumulation of genomics and proteomics information, especially protein interaction data, motivated us to develop computational approaches to mine biological pathways. In this study, we considered function similarities of proteins in a PPIN, and introduced a novel disease gene interaction pathway representation and analysis paradigm. We applied our method to find disease gene interaction pathways of CAD, HT and T2D, and demonstrated that the pathways correlated with information on these diseases in the literature.

We demonstrated that complex diseases often have dysfunctions of multiple biological modules or pathways. Similar to traditional approaches (e.g. PathFinder, BowTieBuilder and FASPAD), our method also allows inferring biological pathways in molecular networks when a set of source and/or target proteins are given. As for FASPAD and Pandora, our method is similar to these approaches at the aspect of taking into account of ‘similarity’ features of neighboring proteins in the background of biological molecular networks. It must be noted that our method has the following advantages. First, using the disease gene interaction pathway reveals potential associations between disease genes or proteins that do not connect directly. Second, representing biological networks as combinations of multiple modules is a lossless, compact, and less redundant representation of the PPIN that preserves the connectivity information between modules. Finally, our novel disease gene interaction pathway representation and analysis paradigm could elucidate that disease genes can associate by the mechanism of disease-risk modules with mutual functions interacting with each other. This leads to multiple dysfunctions of biological processes in the pathogenesis of complex diseases.

Our method also has some limitations. For example, constructing a hierarchical tree and searching for underlying associations between disease genes based on the high-throughput biological network is time-consuming. Another limiting factor is that upstream or downstream relationships could not be identified in disease gene interaction pathways using our analysis.

As demonstrated, the disease genes of CAD, HT, and T2D associated by virtue of associations between biological modules in the PPIN. We hypothesize that if the interaction relationships between disease-risk modules were blocked, communications would break down, preventing disease-risk modules from associating with each other. This might provide additional insights into the pathogenesis of CAD, HT, and T2D. Therefore, the interactions between disease-risk modules might be informational for CAD, HT, and T2D treatment and even in fields such as drug target analysis.

We used the examples of CAD, HT, and T2D to determine the feasibility of this method. Once disease genes are determined in the PPIN, our proposed method can be used to identify disease gene interaction pathways for other types of complex diseases, yielding additional clues in the pathogenesis of complex diseases.

## Supporting Information

Figure S1 **The resulting HT disease gene interaction pathway derived from the PPIN by our method.** 87 nodes in pink are disease-risk modules that contain HT disease proteins (purple dots) and other proteins with similar functions, and the labels beside the nodes are their module IDs. The sizes of the nodes are directly proportional to the log number of proteins (1∼866, of which 1∼6 are disease proteins) they contain. 306 edges are the interaction relationships between disease-risk modules they connect.
(TIF)

Figure S2 **The resulting T2D disease gene interaction pathway derived from the PPIN by our method.** 123 nodes in pink are disease-risk modules that contain T2D disease proteins (orange dots) and other proteins with similar functions, and the labels beside the nodes are their module IDs. The sizes of the nodes are directly proportional to the log number of proteins (1∼866, of which 1∼3 are disease proteins) they contain. 579 edges are the interaction relationships between disease-risk modules they connect.
(TIF)

Figure S3 **The distribution of four network metrics of disease gene interaction pathways from random networks.** Boxes are values for disease gene interaction pathways from random networks, and blue diamonds are values for those from HPRD PPIN.
(TIF)

Table S1 **GO functions enriched for disease-risk modules for CAD.**
(DOC)

Table S2 **Common GO functions shared by interacting terms in the CAD disease gene interaction pathway.**
(DOC)

Table S3 **PubMed ID in which KEGG pathways enriched have been proved to be correlated with CAD.**
(DOC)

Table S4 **PubMed ID in which gene pairs between interacting disease-risk terms have been proved to be correlated with CAD.**
(DOC)

Table S5 **PubMed ID in which KEGG pathways enriched have been proved to be correlated with HT.**
(DOC)

Table S6 **PubMed ID in which gene pairs between interacting disease-risk terms have been proved to be correlated with HT.**
(DOC)

Table S7 **PubMed ID in which KEGG pathways enriched have been proved to be correlated with T2D.**
(DOC)

Table S8 **PubMed ID in which gene pairs between interacting disease-risk terms have been proved to be correlated with T2D.**
(DOC)
