# Supplementary figures and images for: Disease Gene Interaction Pathways: A Potential Framework for How Disease Genes Associate by Disease-Risk Modules
Source: PLoS One. 2011 Sep 6;6(9):e24495. doi: 10.1371/journal.pone.0024495 (PMC3167857; doi:10.1371/journal.pone.0024495)

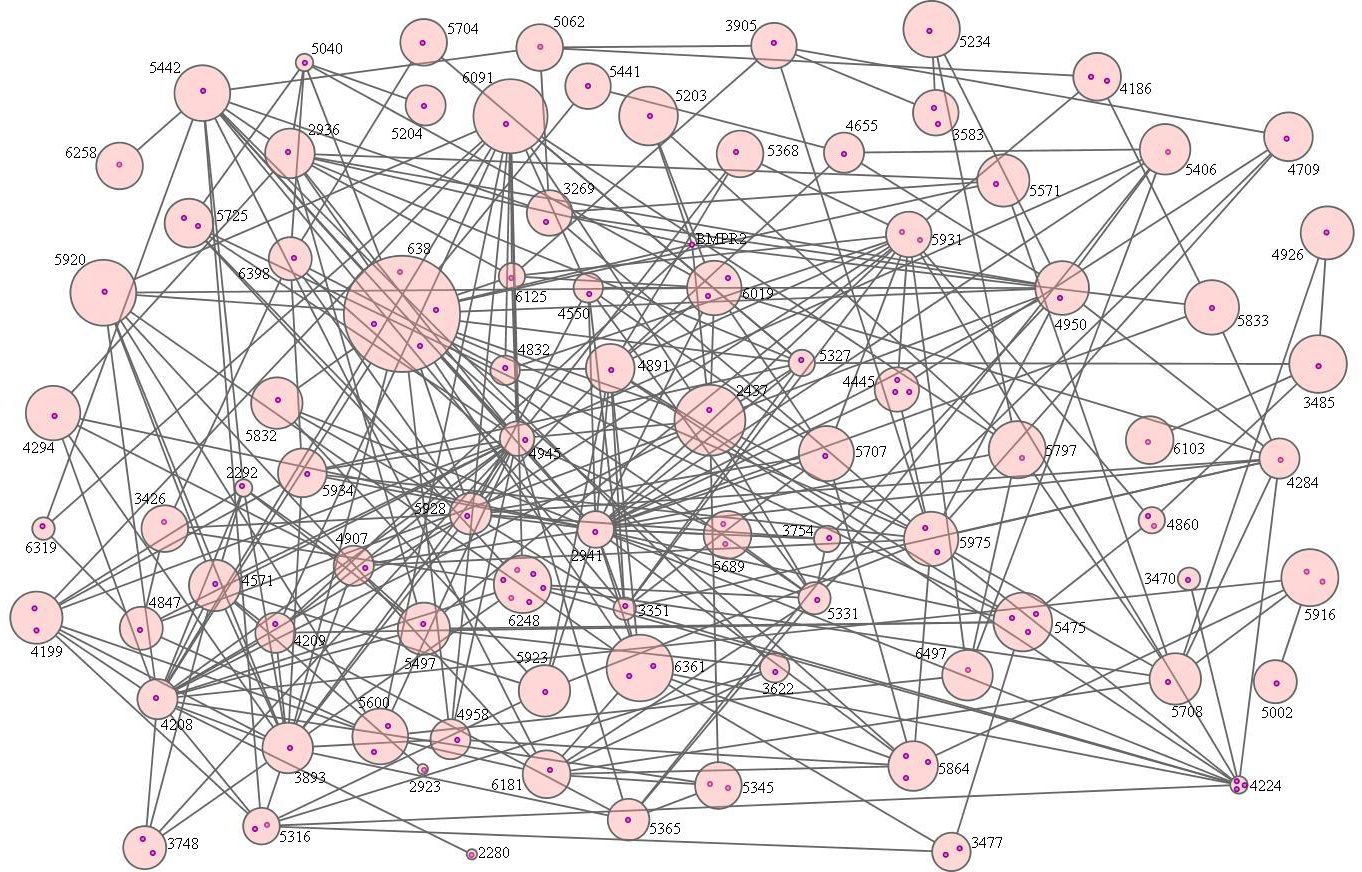

Supplement: Figure S1 — The resulting HT disease gene interaction pathway derived from the PPIN by our method. 87 nodes in pink are disease-risk modules that contain HT disease proteins (purple dots) and other proteins with similar functions, and the labels beside the nodes are their module IDs. The sizes of the nodes are directly proportional to the log number of proteins (1∼866, of which 1∼6 are disease proteins) they contain. 306 edges are the interaction relationships between disease-risk modules they connect. (TIF) [file pone.0024495.s001.tif]

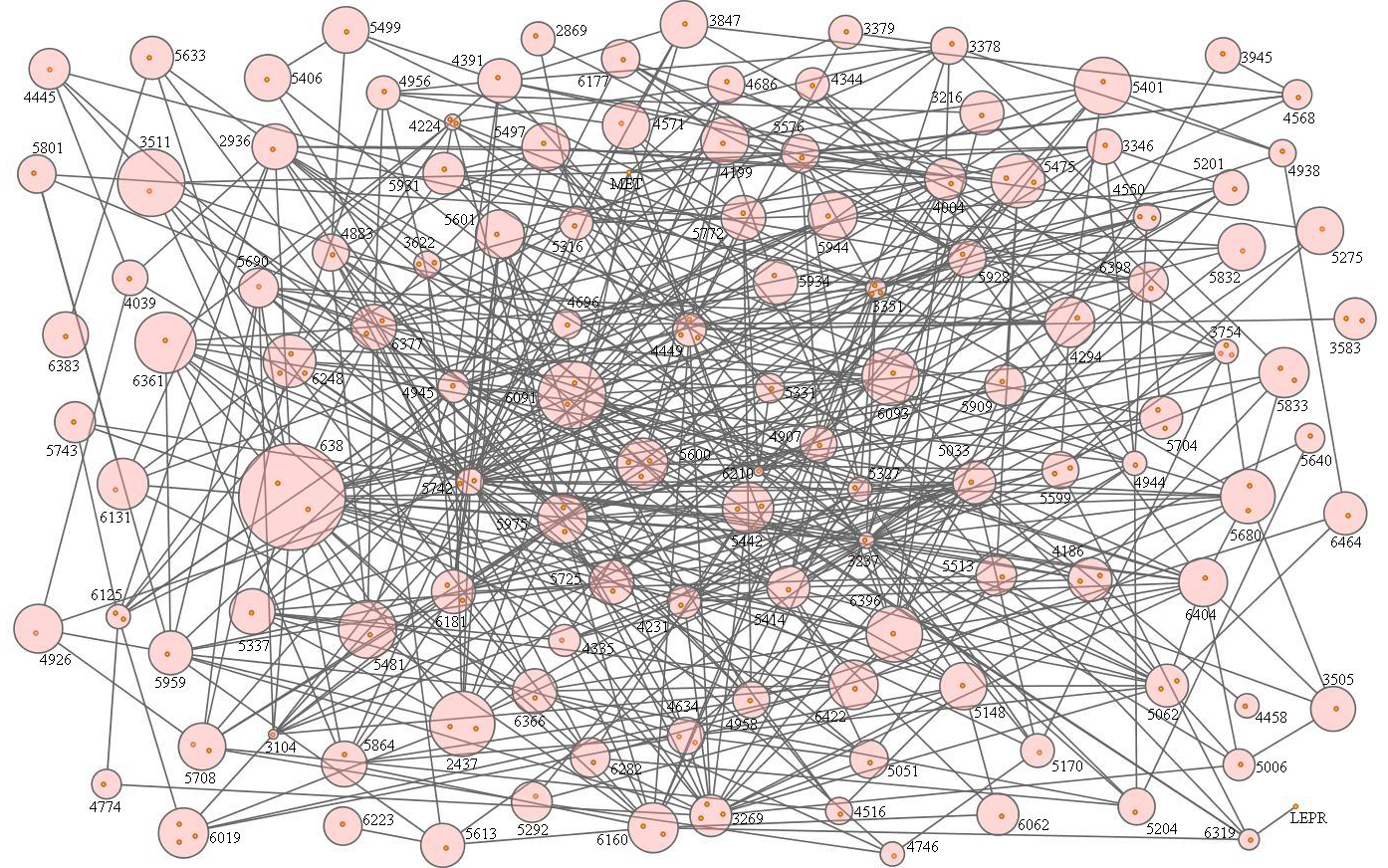

Supplement: Figure S2 — The resulting T2D disease gene interaction pathway derived from the PPIN by our method. 123 nodes in pink are disease-risk modules that contain T2D disease proteins (orange dots) and other proteins with similar functions, and the labels beside the nodes are their module IDs. The sizes of the nodes are directly proportional to the log number of proteins (1∼866, of which 1∼3 are disease proteins) they contain. 579 edges are the interaction relationships between disease-risk modules they connect. (TIF) [file pone.0024495.s002.tif]

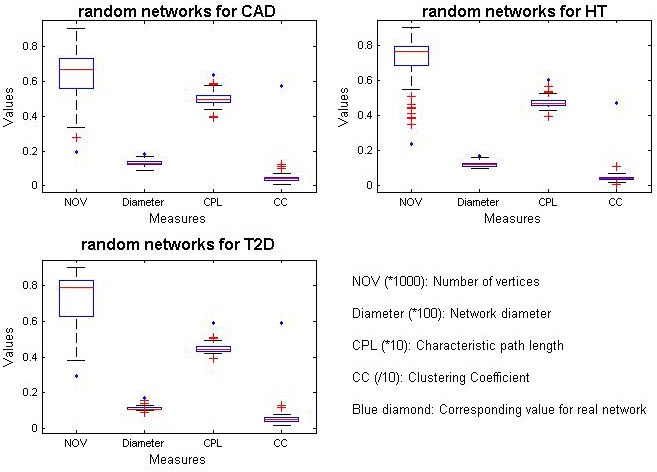

Supplement: Figure S3 — The distribution of four network metrics of disease gene interaction pathways from random networks. Boxes are values for disease gene interaction pathways from random networks, and blue diamonds are values for those from HPRD PPIN. (TIF) [file pone.0024495.s003.tif]
