# Supplementary material for: Disease Gene Interaction Pathways: A Potential Framework for How Disease Genes Associate by Disease-Risk Modules
Source: PLoS One. 2011 Sep 6;6(9):e24495. doi: 10.1371/journal.pone.0024495 (PMC3167857; doi:10.1371/journal.pone.0024495)
Supplement: Table S1 — GO functions enriched for disease-risk modules for CAD. (DOC) [file pone.0024495.s004.doc]

**Table S1. GO functions enriched for disease-risk modules for CAD.**

| **disease-risk module** | **Category** | **Term** | **Pvalue** |
| --- | --- | --- | --- |
| 638 | GOTERM_CC_3 | GO:0005622~intracellular | 9.72E-12 |
|  | GOTERM_CC_2 | GO:0005622~intracellular | 1.32E-11 |
|  | GOTERM_CC_4 | GO:0044424~intracellular part | 2.02E-11 |
|  | GOTERM_CC_3 | GO:0044424~intracellular part | 2.87E-09 |
|  | GOTERM_CC_2 | GO:0044424~intracellular part | 3.65E-09 |
|  | GOTERM_MF_4 | GO:0003779~actin binding | 1.42E-08 |
|  | GOTERM_CC_5 | GO:0043229~intracellular organelle | 9.03E-07 |
|  | GOTERM_CC_4 | GO:0043229~intracellular organelle | 1.83E-06 |
|  | GOTERM_CC_1 | GO:0043226~organelle | 2.20E-06 |
|  | GOTERM_CC_5 | GO:0005634~nucleus | 9.14E-06 |
|  | GOTERM_CC_4 | GO:0005634~nucleus | 1.21E-05 |
|  | GOTERM_CC_3 | GO:0043229~intracellular organelle | 1.44E-05 |
|  | GOTERM_CC_2 | GO:0043229~intracellular organelle | 1.62E-05 |
|  | GOTERM_MF_3 | GO:0042166~acetylcholine binding | 2.43E-05 |
|  | GOTERM_CC_5 | GO:0005892~nicotinic acetylcholine-gated receptor-channel complex | 4.07E-05 |
|  | GOTERM_CC_4 | GO:0005892~nicotinic acetylcholine-gated receptor-channel complex | 4.12E-05 |
|  | GOTERM_MF_3 | GO:0008092~cytoskeletal protein binding | 4.18E-05 |
|  | GOTERM_MF_5 | GO:0015464~acetylcholine receptor activity | 8.48E-05 |
|  | GOTERM_MF_4 | GO:0015464~acetylcholine receptor activity | 8.68E-05 |
|  | GOTERM_MF_5 | GO:0046914~transition metal ion binding | 1.88E-04 |
|  | GOTERM_BP_3 | GO:0010467~gene expression | 6.44E-04 |
|  | GOTERM_BP_3 | GO:0051640~organelle localization | 8.11E-04 |
|  | GOTERM_CC_5 | GO:0043232~intracellular non-membrane-bounded organelle | 9.34E-04 |
|  | GOTERM_BP_2 | GO:0009058~biosynthetic process | 9.47E-04 |
|  | GOTERM_BP_3 | GO:0044249~cellular biosynthetic process | 9.83E-04 |
|  | GOTERM_CC_4 | GO:0043232~intracellular non-membrane-bounded organelle | 0.001025 |
|  | GOTERM_CC_5 | GO:0005856~cytoskeleton | 0.001111 |
|  | GOTERM_CC_5 | GO:0043231~intracellular membrane-bounded organelle | 0.001119 |
|  | GOTERM_CC_4 | GO:0005856~cytoskeleton | 0.001184 |
|  | GOTERM_MF_5 | GO:0070003~threonine-type peptidase activity | 0.001471 |
|  | GOTERM_CC_5 | GO:0005839~proteasome core complex | 0.001504 |
|  | GOTERM_CC_4 | GO:0005839~proteasome core complex | 0.001518 |
|  | GOTERM_CC_4 | GO:0043231~intracellular membrane-bounded organelle | 0.001523 |
|  | GOTERM_CC_3 | GO:0043232~intracellular non-membrane-bounded organelle | 0.001532 |
|  | GOTERM_CC_3 | GO:0005839~proteasome core complex | 0.001576 |
|  | GOTERM_CC_2 | GO:0043228~non-membrane-bounded organelle | 0.001593 |
|  | GOTERM_BP_1 | GO:0009987~cellular process | 0.001664 |
|  | GOTERM_MF_2 | GO:0043176~amine binding | 0.001713 |
|  | GOTERM_BP_2 | GO:0022613~ribonucleoprotein complex biogenesis | 0.003217 |
|  | GOTERM_BP_3 | GO:0042254~ribosome biogenesis | 0.003243 |
|  | GOTERM_BP_4 | GO:0034645~cellular macromolecule biosynthetic process | 0.003285 |
|  | GOTERM_BP_4 | GO:0006350~transcription | 0.00386 |
|  | GOTERM_MF_1 | GO:0005488~binding | 0.003938 |
|  | GOTERM_BP_3 | GO:0009059~macromolecule biosynthetic process | 0.003983 |
|  | GOTERM_CC_3 | GO:0043231~intracellular membrane-bounded organelle | 0.004078 |
|  | GOTERM_CC_5 | GO:0015629~actin cytoskeleton | 0.004328 |
|  | GOTERM_MF_2 | GO:0003676~nucleic acid binding | 0.004416 |
|  | GOTERM_CC_2 | GO:0043227~membrane-bounded organelle | 0.004686 |
|  | GOTERM_BP_5 | GO:0007080~mitotic metaphase plate congression | 0.005959 |
|  | GOTERM_BP_4 | GO:0007080~mitotic metaphase plate congression | 0.006072 |
|  | GOTERM_BP_3 | GO:0007080~mitotic metaphase plate congression | 0.006161 |
|  | GOTERM_MF_3 | GO:0003723~RNA binding | 0.006254 |
|  | GOTERM_MF_2 | GO:0005515~protein binding | 0.006347 |
|  | GOTERM_MF_5 | GO:0016462~pyrophosphatase activity | 0.006695 |
|  | GOTERM_MF_4 | GO:0016818~hydrolase activity, acting on acid anhydrides, in phosphorus-containing anhydrides | 0.008101 |
|  | GOTERM_CC_5 | GO:0030054~cell junction | 0.008709 |
|  | GOTERM_CC_5 | GO:0005884~actin filament | 0.008905 |
|  | GOTERM_CC_4 | GO:0005884~actin filament | 0.008991 |
|  | GOTERM_CC_4 | GO:0030054~cell junction | 0.009102 |
|  | GOTERM_CC_5 | GO:0005737~cytoplasm | 0.009825 |
|  | GOTERM_BP_5 | GO:0051310~metaphase plate congression | 0.010954 |
|  | GOTERM_BP_4 | GO:0051310~metaphase plate congression | 0.011158 |
|  | GOTERM_BP_3 | GO:0051310~metaphase plate congression | 0.011317 |
|  | GOTERM_BP_4 | GO:0051656~establishment of organelle localization | 0.011435 |
|  | GOTERM_BP_3 | GO:0051656~establishment of organelle localization | 0.011775 |
|  | GOTERM_MF_3 | GO:0016817~hydrolase activity, acting on acid anhydrides | 0.011923 |
|  | GOTERM_CC_4 | GO:0005737~cytoplasm | 0.011976 |
|  | GOTERM_BP_2 | GO:0051656~establishment of organelle localization | 0.012143 |
|  | GOTERM_BP_5 | GO:0043632~modification-dependent macromolecule catabolic process | 0.013781 |
|  | GOTERM_BP_3 | GO:0044260~cellular macromolecule metabolic process | 0.014759 |
|  | GOTERM_MF_4 | GO:0016790~thiolester hydrolase activity | 0.014877 |
|  | GOTERM_CC_4 | GO:0045211~postsynaptic membrane | 0.017152 |
|  | GOTERM_BP_5 | GO:0006555~methionine metabolic process | 0.017776 |
|  | GOTERM_CC_1 | GO:0044464~cell part | 0.018104 |
|  | GOTERM_CC_3 | GO:0045211~postsynaptic membrane | 0.018203 |
|  | GOTERM_CC_2 | GO:0045211~postsynaptic membrane | 0.018298 |
|  | GOTERM_CC_1 | GO:0005623~cell | 0.018392 |
|  | GOTERM_CC_1 | GO:0044422~organelle part | 0.01879 |
|  | GOTERM_BP_5 | GO:0009066~aspartate family amino acid metabolic process | 0.020047 |
|  | GOTERM_CC_5 | GO:0044446~intracellular organelle part | 0.020876 |
|  | GOTERM_BP_5 | GO:0006412~translation | 0.022191 |
|  | GOTERM_BP_3 | GO:0042180~cellular ketone metabolic process | 0.022338 |
|  | GOTERM_BP_5 | GO:0019752~carboxylic acid metabolic process | 0.022867 |
|  | GOTERM_CC_3 | GO:0005737~cytoplasm | 0.023229 |
|  | GOTERM_CC_4 | GO:0044446~intracellular organelle part | 0.023483 |
|  | GOTERM_BP_2 | GO:0044237~cellular metabolic process | 0.02417 |
|  | GOTERM_BP_4 | GO:0006412~translation | 0.024332 |
|  | GOTERM_MF_4 | GO:0030594~neurotransmitter receptor activity | 0.024791 |
|  | GOTERM_BP_4 | GO:0043436~oxoacid metabolic process | 0.025858 |
|  | GOTERM_BP_3 | GO:0034641~cellular nitrogen compound metabolic process | 0.026478 |
|  | GOTERM_BP_5 | GO:0051303~establishment of chromosome localization | 0.026483 |
|  | GOTERM_BP_5 | GO:0046847~filopodium assembly | 0.026483 |
|  | GOTERM_CC_5 | GO:0031941~filamentous actin | 0.026533 |
|  | GOTERM_CC_4 | GO:0031941~filamentous actin | 0.026673 |
|  | GOTERM_BP_4 | GO:0051303~establishment of chromosome localization | 0.026951 |
|  | GOTERM_BP_4 | GO:0050000~chromosome localization | 0.026951 |
|  | GOTERM_MF_3 | GO:0030594~neurotransmitter receptor activity | 0.02718 |
|  | GOTERM_CC_3 | GO:0031941~filamentous actin | 0.027258 |
|  | GOTERM_BP_3 | GO:0051303~establishment of chromosome localization | 0.027316 |
|  | GOTERM_BP_5 | GO:0044257~cellular protein catabolic process | 0.02734 |
|  | GOTERM_MF_5 | GO:0051015~actin filament binding | 0.028559 |
|  | GOTERM_BP_3 | GO:0006082~organic acid metabolic process | 0.02936 |
|  | GOTERM_BP_1 | GO:0008152~metabolic process | 0.029931 |
|  | GOTERM_BP_4 | GO:0030035~microspike assembly | 0.032097 |
|  | GOTERM_CC_3 | GO:0044446~intracellular organelle part | 0.033032 |
|  | GOTERM_MF_5 | GO:0004864~phosphoprotein phosphatase inhibitor activity | 0.033228 |
|  | GOTERM_MF_4 | GO:0004864~phosphoprotein phosphatase inhibitor activity | 0.033628 |
|  | GOTERM_BP_5 | GO:0043242~negative regulation of protein complex disassembly | 0.033824 |
|  | GOTERM_BP_5 | GO:0051494~negative regulation of cytoskeleton organization | 0.034093 |
|  | GOTERM_BP_4 | GO:0043242~negative regulation of protein complex disassembly | 0.034682 |
|  | GOTERM_CC_2 | GO:0044446~intracellular organelle part | 0.034763 |
|  | GOTERM_BP_4 | GO:0051494~negative regulation of cytoskeleton organization | 0.035076 |
|  | GOTERM_BP_3 | GO:0043242~negative regulation of protein complex disassembly | 0.035351 |
|  | GOTERM_CC_5 | GO:0016461~unconventional myosin complex | 0.03557 |
|  | GOTERM_CC_4 | GO:0016461~unconventional myosin complex | 0.035703 |
|  | GOTERM_CC_2 | GO:0044422~organelle part | 0.035782 |
|  | GOTERM_BP_4 | GO:0035095~behavioral response to nicotine | 0.035971 |
|  | GOTERM_BP_3 | GO:0035095~behavioral response to nicotine | 0.036317 |
|  | GOTERM_CC_5 | GO:0044430~cytoskeletal part | 0.036527 |
|  | GOTERM_CC_5 | GO:0005874~microtubule | 0.036609 |
|  | GOTERM_CC_4 | GO:0005874~microtubule | 0.03775 |
|  | GOTERM_CC_4 | GO:0044430~cytoskeletal part | 0.038087 |
|  | GOTERM_BP_3 | GO:0006139~nucleobase, nucleoside, nucleotide and nucleic acid metabolic process | 0.038784 |
|  | GOTERM_BP_5 | GO:0030163~protein catabolic process | 0.040896 |
|  | GOTERM_BP_5 | GO:0010468~regulation of gene expression | 0.042 |
|  | GOTERM_MF_4 | GO:0019212~phosphatase inhibitor activity | 0.042015 |
|  | GOTERM_BP_5 | GO:0001843~neural tube closure | 0.042022 |
|  | GOTERM_BP_5 | GO:0060606~tube closure | 0.042022 |
|  | GOTERM_CC_5 | GO:0070161~anchoring junction | 0.042759 |
|  | GOTERM_BP_5 | GO:0046394~carboxylic acid biosynthetic process | 0.042865 |
|  | GOTERM_BP_4 | GO:0060606~tube closure | 0.042908 |
|  | GOTERM_MF_4 | GO:0016780~phosphotransferase activity, for other substituted phosphate groups | 0.043066 |
|  | GOTERM_CC_3 | GO:0044430~cytoskeletal part | 0.043355 |
|  | GOTERM_CC_5 | GO:0005911~cell-cell junction | 0.043647 |
|  | GOTERM_BP_5 | GO:0006520~cellular amino acid metabolic process | 0.043874 |
|  | GOTERM_BP_5 | GO:0006487~protein amino acid N-linked glycosylation | 0.044045 |
|  | GOTERM_MF_3 | GO:0019212~phosphatase inhibitor activity | 0.044165 |
|  | GOTERM_BP_4 | GO:0016053~organic acid biosynthetic process | 0.044772 |
|  | GOTERM_BP_5 | GO:0060429~epithelium development | 0.045458 |
|  | GOTERM_BP_4 | GO:0006520~cellular amino acid metabolic process | 0.046109 |
|  | GOTERM_BP_4 | GO:0030163~protein catabolic process | 0.046735 |
|  | GOTERM_MF_4 | GO:0046872~metal ion binding | 0.046982 |
|  | GOTERM_MF_5 | GO:0004221~ubiquitin thiolesterase activity | 0.047196 |
|  | GOTERM_BP_4 | GO:0060429~epithelium development | 0.048045 |
|  | GOTERM_CC_5 | GO:0070469~respiratory chain | 0.048243 |
|  | GOTERM_CC_4 | GO:0070469~respiratory chain | 0.048682 |
|  | GOTERM_MF_2 | GO:0042165~neurotransmitter binding | 0.049378 |
| 3866 | GOTERM_MF_5 | GO:0003707~steroid hormone receptor activity | 2.52E-41 |
|  | GOTERM_MF_4 | GO:0004879~ligand-dependent nuclear receptor activity | 4.43E-41 |
|  | GOTERM_MF_4 | GO:0043565~sequence-specific DNA binding | 1.04E-22 |
|  | GOTERM_MF_2 | GO:0003700~transcription factor activity | 7.07E-21 |
|  | GOTERM_MF_4 | GO:0003700~transcription factor activity | 3.57E-19 |
|  | GOTERM_MF_1 | GO:0030528~transcription regulator activity | 9.06E-18 |
|  | GOTERM_MF_2 | GO:0016563~transcription activator activity | 9.23E-16 |
|  | GOTERM_BP_4 | GO:0051252~regulation of RNA metabolic process | 2.13E-15 |
|  | GOTERM_MF_3 | GO:0004872~receptor activity | 3.16E-15 |
|  | GOTERM_BP_5 | GO:0006355~regulation of transcription, DNA-dependent | 4.74E-15 |
|  | GOTERM_BP_5 | GO:0051252~regulation of RNA metabolic process | 6.94E-15 |
|  | GOTERM_MF_1 | GO:0060089~molecular transducer activity | 9.33E-15 |
|  | GOTERM_MF_2 | GO:0004871~signal transducer activity | 1.33E-14 |
|  | GOTERM_BP_4 | GO:0006350~transcription | 2.64E-14 |
|  | GOTERM_MF_3 | GO:0003677~DNA binding | 1.83E-13 |
|  | GOTERM_BP_4 | GO:0010628~positive regulation of gene expression | 2.45E-13 |
|  | GOTERM_BP_5 | GO:0010628~positive regulation of gene expression | 5.55E-13 |
|  | GOTERM_BP_4 | GO:0051254~positive regulation of RNA metabolic process | 1.39E-12 |
|  | GOTERM_BP_5 | GO:0045893~positive regulation of transcription, DNA-dependent | 2.69E-12 |
|  | GOTERM_BP_5 | GO:0051254~positive regulation of RNA metabolic process | 2.95E-12 |
|  | GOTERM_BP_3 | GO:0009059~macromolecule biosynthetic process | 2.98E-12 |
|  | GOTERM_BP_3 | GO:0051171~regulation of nitrogen compound metabolic process | 3.10E-12 |
|  | GOTERM_BP_5 | GO:0045449~regulation of transcription | 3.28E-12 |
|  | GOTERM_BP_4 | GO:0034645~cellular macromolecule biosynthetic process | 3.82E-12 |
|  | GOTERM_BP_4 | GO:0019219~regulation of nucleobase, nucleoside, nucleotide and nucleic acid metabolic process | 3.86E-12 |
|  | GOTERM_BP_4 | GO:0010556~regulation of macromolecule biosynthetic process | 4.25E-12 |
|  | GOTERM_BP_5 | GO:0030522~intracellular receptor-mediated signaling pathway | 4.38E-12 |
|  | GOTERM_BP_4 | GO:0051171~regulation of nitrogen compound metabolic process | 4.49E-12 |
|  | GOTERM_BP_4 | GO:0010468~regulation of gene expression | 4.97E-12 |
|  | GOTERM_MF_2 | GO:0003676~nucleic acid binding | 6.50E-12 |
|  | GOTERM_BP_3 | GO:0009889~regulation of biosynthetic process | 6.55E-12 |
|  | GOTERM_BP_2 | GO:0048522~positive regulation of cellular process | 6.74E-12 |
|  | GOTERM_BP_3 | GO:0010467~gene expression | 7.90E-12 |
|  | GOTERM_BP_4 | GO:0031326~regulation of cellular biosynthetic process | 8.49E-12 |
|  | GOTERM_BP_4 | GO:0009889~regulation of biosynthetic process | 9.47E-12 |
|  | GOTERM_BP_3 | GO:0048522~positive regulation of cellular process | 1.13E-11 |
|  | GOTERM_BP_5 | GO:0019219~regulation of nucleobase, nucleoside, nucleotide and nucleic acid metabolic process | 1.26E-11 |
|  | GOTERM_BP_5 | GO:0010556~regulation of macromolecule biosynthetic process | 1.38E-11 |
|  | GOTERM_BP_4 | GO:0048522~positive regulation of cellular process | 1.56E-11 |
|  | GOTERM_BP_5 | GO:0010468~regulation of gene expression | 1.62E-11 |
|  | GOTERM_BP_5 | GO:0045941~positive regulation of transcription | 1.66E-11 |
|  | GOTERM_BP_3 | GO:0010604~positive regulation of macromolecule metabolic process | 1.89E-11 |
|  | GOTERM_BP_4 | GO:0045935~positive regulation of nucleobase, nucleoside, nucleotide and nucleic acid metabolic process | 2.36E-11 |
|  | GOTERM_BP_4 | GO:0010604~positive regulation of macromolecule metabolic process | 2.44E-11 |
|  | GOTERM_BP_3 | GO:0051173~positive regulation of nitrogen compound metabolic process | 2.63E-11 |
|  | GOTERM_BP_5 | GO:0031326~regulation of cellular biosynthetic process | 2.76E-11 |
|  | GOTERM_BP_2 | GO:0048518~positive regulation of biological process | 2.78E-11 |
|  | GOTERM_BP_2 | GO:0009893~positive regulation of metabolic process | 2.92E-11 |
|  | GOTERM_BP_3 | GO:0060255~regulation of macromolecule metabolic process | 3.26E-11 |
|  | GOTERM_BP_4 | GO:0051173~positive regulation of nitrogen compound metabolic process | 3.32E-11 |
|  | GOTERM_BP_3 | GO:0080090~regulation of primary metabolic process | 3.85E-11 |
|  | GOTERM_BP_4 | GO:0010557~positive regulation of macromolecule biosynthetic process | 3.92E-11 |
|  | GOTERM_BP_3 | GO:0009893~positive regulation of metabolic process | 4.41E-11 |
|  | GOTERM_BP_3 | GO:0048518~positive regulation of biological process | 4.65E-11 |
|  | GOTERM_BP_4 | GO:0060255~regulation of macromolecule metabolic process | 4.72E-11 |
|  | GOTERM_BP_5 | GO:0045935~positive regulation of nucleobase, nucleoside, nucleotide and nucleic acid metabolic process | 4.96E-11 |
|  | GOTERM_BP_5 | GO:0010604~positive regulation of macromolecule metabolic process | 5.47E-11 |
|  | GOTERM_BP_4 | GO:0080090~regulation of primary metabolic process | 5.57E-11 |
|  | GOTERM_BP_4 | GO:0009893~positive regulation of metabolic process | 5.68E-11 |
|  | GOTERM_BP_3 | GO:0009891~positive regulation of biosynthetic process | 5.98E-11 |
|  | GOTERM_BP_4 | GO:0031328~positive regulation of cellular biosynthetic process | 6.45E-11 |
|  | GOTERM_BP_5 | GO:0051173~positive regulation of nitrogen compound metabolic process | 6.97E-11 |
|  | GOTERM_BP_3 | GO:0006139~nucleobase, nucleoside, nucleotide and nucleic acid metabolic process | 7.02E-11 |
|  | GOTERM_BP_5 | GO:0016481~negative regulation of transcription | 7.36E-11 |
|  | GOTERM_BP_2 | GO:0009058~biosynthetic process | 7.41E-11 |
|  | GOTERM_BP_4 | GO:0009891~positive regulation of biosynthetic process | 7.55E-11 |
|  | GOTERM_BP_5 | GO:0010557~positive regulation of macromolecule biosynthetic process | 8.23E-11 |
|  | GOTERM_BP_3 | GO:0044249~cellular biosynthetic process | 8.27E-11 |
|  | GOTERM_BP_3 | GO:0031323~regulation of cellular metabolic process | 9.22E-11 |
|  | GOTERM_BP_4 | GO:0010629~negative regulation of gene expression | 9.40E-11 |
|  | GOTERM_BP_3 | GO:0051172~negative regulation of nitrogen compound metabolic process | 1.01E-10 |
|  | GOTERM_BP_2 | GO:0019222~regulation of metabolic process | 1.08E-10 |
|  | GOTERM_BP_4 | GO:0045934~negative regulation of nucleobase, nucleoside, nucleotide and nucleic acid metabolic process | 1.10E-10 |
|  | GOTERM_MF_3 | GO:0008134~transcription factor binding | 1.23E-10 |
|  | GOTERM_BP_4 | GO:0051172~negative regulation of nitrogen compound metabolic process | 1.25E-10 |
|  | GOTERM_MF_3 | GO:0005496~steroid binding | 1.30E-10 |
|  | GOTERM_BP_4 | GO:0031323~regulation of cellular metabolic process | 1.33E-10 |
|  | GOTERM_BP_5 | GO:0031328~positive regulation of cellular biosynthetic process | 1.35E-10 |
|  | GOTERM_BP_5 | GO:0009891~positive regulation of biosynthetic process | 1.58E-10 |
|  | GOTERM_BP_5 | GO:0010629~negative regulation of gene expression | 1.85E-10 |
|  | GOTERM_BP_3 | GO:0019222~regulation of metabolic process | 1.96E-10 |
|  | GOTERM_BP_4 | GO:0010558~negative regulation of macromolecule biosynthetic process | 2.10E-10 |
|  | GOTERM_BP_5 | GO:0045934~negative regulation of nucleobase, nucleoside, nucleotide and nucleic acid metabolic process | 2.16E-10 |
|  | GOTERM_BP_2 | GO:0006807~nitrogen compound metabolic process | 2.22E-10 |
|  | GOTERM_BP_5 | GO:0051172~negative regulation of nitrogen compound metabolic process | 2.46E-10 |
|  | GOTERM_BP_3 | GO:0034641~cellular nitrogen compound metabolic process | 2.47E-10 |
|  | GOTERM_BP_3 | GO:0009890~negative regulation of biosynthetic process | 2.68E-10 |
|  | GOTERM_BP_4 | GO:0031327~negative regulation of cellular biosynthetic process | 2.69E-10 |
|  | GOTERM_BP_4 | GO:0009890~negative regulation of biosynthetic process | 3.31E-10 |
|  | GOTERM_MF_5 | GO:0046914~transition metal ion binding | 3.78E-10 |
|  | GOTERM_MF_2 | GO:0008289~lipid binding | 3.96E-10 |
|  | GOTERM_BP_5 | GO:0010558~negative regulation of macromolecule biosynthetic process | 4.12E-10 |
|  | GOTERM_BP_5 | GO:0031327~negative regulation of cellular biosynthetic process | 5.28E-10 |
|  | GOTERM_MF_2 | GO:0043167~ion binding | 5.62E-10 |
|  | GOTERM_BP_5 | GO:0009890~negative regulation of biosynthetic process | 6.49E-10 |
|  | GOTERM_BP_3 | GO:0031325~positive regulation of cellular metabolic process | 7.53E-10 |
|  | GOTERM_BP_4 | GO:0031325~positive regulation of cellular metabolic process | 9.49E-10 |
|  | GOTERM_BP_5 | GO:0031325~positive regulation of cellular metabolic process | 1.98E-09 |
|  | GOTERM_BP_3 | GO:0031324~negative regulation of cellular metabolic process | 2.49E-09 |
|  | GOTERM_MF_5 | GO:0003708~retinoic acid receptor activity | 2.85E-09 |
|  | GOTERM_BP_3 | GO:0010605~negative regulation of macromolecule metabolic process | 3.00E-09 |
|  | GOTERM_BP_4 | GO:0031324~negative regulation of cellular metabolic process | 3.07E-09 |
|  | GOTERM_BP_4 | GO:0010605~negative regulation of macromolecule metabolic process | 3.70E-09 |
|  | GOTERM_MF_3 | GO:0043169~cation binding | 3.83E-09 |
|  | GOTERM_BP_2 | GO:0009892~negative regulation of metabolic process | 3.85E-09 |
|  | GOTERM_BP_3 | GO:0009892~negative regulation of metabolic process | 5.40E-09 |
|  | GOTERM_BP_5 | GO:0031324~negative regulation of cellular metabolic process | 5.98E-09 |
|  | GOTERM_BP_4 | GO:0009892~negative regulation of metabolic process | 6.65E-09 |
|  | GOTERM_BP_5 | GO:0010605~negative regulation of macromolecule metabolic process | 7.20E-09 |
|  | GOTERM_CC_4 | GO:0005634~nucleus | 1.44E-08 |
|  | GOTERM_MF_4 | GO:0046872~metal ion binding | 1.88E-08 |
|  | GOTERM_CC_5 | GO:0005634~nucleus | 1.91E-08 |
|  | GOTERM_BP_2 | GO:0042221~response to chemical stimulus | 2.77E-08 |
|  | GOTERM_BP_2 | GO:0048519~negative regulation of biological process | 7.37E-08 |
|  | GOTERM_BP_3 | GO:0044260~cellular macromolecule metabolic process | 9.76E-08 |
|  | GOTERM_BP_3 | GO:0048519~negative regulation of biological process | 1.10E-07 |
|  | GOTERM_MF_5 | GO:0046965~retinoid X receptor binding | 2.30E-07 |
|  | GOTERM_BP_2 | GO:0043170~macromolecule metabolic process | 2.52E-07 |
|  | GOTERM_BP_2 | GO:0048523~negative regulation of cellular process | 4.11E-07 |
|  | GOTERM_BP_3 | GO:0048523~negative regulation of cellular process | 5.90E-07 |
|  | GOTERM_BP_4 | GO:0048523~negative regulation of cellular process | 7.36E-07 |
|  | GOTERM_BP_3 | GO:0010033~response to organic substance | 1.03E-06 |
|  | GOTERM_BP_4 | GO:0033273~response to vitamin | 1.30E-06 |
|  | GOTERM_BP_3 | GO:0007165~signal transduction | 1.60E-06 |
|  | GOTERM_MF_4 | GO:0042974~retinoic acid receptor binding | 1.68E-06 |
|  | GOTERM_MF_2 | GO:0003712~transcription cofactor activity | 2.04E-06 |
|  | GOTERM_BP_4 | GO:0007165~signal transduction | 2.08E-06 |
|  | GOTERM_BP_2 | GO:0044237~cellular metabolic process | 3.26E-06 |
|  | GOTERM_BP_3 | GO:0009725~response to hormone stimulus | 3.78E-06 |
|  | GOTERM_BP_4 | GO:0031667~response to nutrient levels | 3.80E-06 |
|  | GOTERM_BP_4 | GO:0051253~negative regulation of RNA metabolic process | 3.96E-06 |
|  | GOTERM_BP_2 | GO:0050794~regulation of cellular process | 5.17E-06 |
|  | GOTERM_BP_5 | GO:0045892~negative regulation of transcription, DNA-dependent | 5.34E-06 |
|  | GOTERM_BP_2 | GO:0009719~response to endogenous stimulus | 5.45E-06 |
|  | GOTERM_BP_3 | GO:0009991~response to extracellular stimulus | 5.87E-06 |
|  | GOTERM_BP_5 | GO:0051253~negative regulation of RNA metabolic process | 5.88E-06 |
|  | GOTERM_MF_3 | GO:0003713~transcription coactivator activity | 5.96E-06 |
|  | GOTERM_MF_4 | GO:0051427~hormone receptor binding | 6.44E-06 |
|  | GOTERM_BP_2 | GO:0044238~primary metabolic process | 6.69E-06 |
|  | GOTERM_MF_5 | GO:0035257~nuclear hormone receptor binding | 7.29E-06 |
|  | GOTERM_MF_4 | GO:0003712~transcription cofactor activity | 7.65E-06 |
|  | GOTERM_BP_3 | GO:0050794~regulation of cellular process | 9.41E-06 |
|  | GOTERM_BP_3 | GO:0048513~organ development | 1.03E-05 |
|  | GOTERM_BP_2 | GO:0050789~regulation of biological process | 1.04E-05 |
|  | GOTERM_BP_4 | GO:0019216~regulation of lipid metabolic process | 1.08E-05 |
|  | GOTERM_BP_4 | GO:0048513~organ development | 1.25E-05 |
|  | GOTERM_BP_5 | GO:0019216~regulation of lipid metabolic process | 1.42E-05 |
|  | GOTERM_CC_3 | GO:0043231~intracellular membrane-bounded organelle | 1.59E-05 |
|  | GOTERM_CC_2 | GO:0043227~membrane-bounded organelle | 1.59E-05 |
|  | GOTERM_MF_5 | GO:0003713~transcription coactivator activity | 2.19E-05 |
|  | GOTERM_BP_1 | GO:0065007~biological regulation | 2.22E-05 |
|  | GOTERM_BP_3 | GO:0010887~negative regulation of cholesterol storage | 2.24E-05 |
|  | GOTERM_BP_4 | GO:0010887~negative regulation of cholesterol storage | 2.34E-05 |
|  | GOTERM_BP_3 | GO:0007584~response to nutrient | 2.40E-05 |
|  | GOTERM_BP_5 | GO:0033189~response to vitamin A | 2.44E-05 |
|  | GOTERM_BP_5 | GO:0010887~negative regulation of cholesterol storage | 2.69E-05 |
|  | GOTERM_BP_4 | GO:0032774~RNA biosynthetic process | 2.74E-05 |
|  | GOTERM_CC_4 | GO:0043231~intracellular membrane-bounded organelle | 3.19E-05 |
|  | GOTERM_BP_1 | GO:0008152~metabolic process | 3.20E-05 |
|  | GOTERM_BP_5 | GO:0007584~response to nutrient | 3.42E-05 |
|  | GOTERM_BP_5 | GO:0006351~transcription, DNA-dependent | 3.58E-05 |
|  | GOTERM_BP_5 | GO:0032774~RNA biosynthetic process | 3.82E-05 |
|  | GOTERM_CC_5 | GO:0043231~intracellular membrane-bounded organelle | 4.23E-05 |
|  | GOTERM_BP_4 | GO:0010565~regulation of cellular ketone metabolic process | 5.00E-05 |
|  | GOTERM_MF_5 | GO:0004887~thyroid hormone receptor activity | 5.73E-05 |
|  | GOTERM_CC_1 | GO:0043226~organelle | 6.06E-05 |
|  | GOTERM_BP_5 | GO:0010565~regulation of cellular ketone metabolic process | 6.14E-05 |
|  | GOTERM_BP_4 | GO:0007242~intracellular signaling cascade | 7.39E-05 |
|  | GOTERM_BP_4 | GO:0010885~regulation of cholesterol storage | 8.56E-05 |
|  | GOTERM_BP_2 | GO:0010888~negative regulation of lipid storage | 9.17E-05 |
|  | GOTERM_BP_5 | GO:0010885~regulation of cholesterol storage | 9.83E-05 |
|  | GOTERM_BP_3 | GO:0010888~negative regulation of lipid storage | 9.83E-05 |
|  | GOTERM_BP_4 | GO:0010888~negative regulation of lipid storage | 1.03E-04 |
|  | GOTERM_BP_3 | GO:0009888~tissue development | 1.10E-04 |
|  | GOTERM_MF_5 | GO:0010843~promoter binding | 1.14E-04 |
|  | GOTERM_CC_2 | GO:0043229~intracellular organelle | 1.16E-04 |
|  | GOTERM_CC_3 | GO:0043229~intracellular organelle | 1.17E-04 |
|  | GOTERM_BP_5 | GO:0010888~negative regulation of lipid storage | 1.18E-04 |
|  | GOTERM_BP_5 | GO:0007242~intracellular signaling cascade | 1.21E-04 |
|  | GOTERM_BP_4 | GO:0009888~tissue development | 1.24E-04 |
|  | GOTERM_BP_3 | GO:0048731~system development | 1.41E-04 |
|  | GOTERM_BP_5 | GO:0009888~tissue development | 1.81E-04 |
|  | GOTERM_BP_2 | GO:0048856~anatomical structure development | 2.10E-04 |
|  | GOTERM_CC_4 | GO:0043229~intracellular organelle | 2.36E-04 |
|  | GOTERM_BP_3 | GO:0010883~regulation of lipid storage | 3.11E-04 |
|  | GOTERM_CC_5 | GO:0043229~intracellular organelle | 3.12E-04 |
|  | GOTERM_BP_4 | GO:0010883~regulation of lipid storage | 3.24E-04 |
|  | GOTERM_BP_4 | GO:0045923~positive regulation of fatty acid metabolic process | 3.24E-04 |
|  | GOTERM_BP_5 | GO:0032868~response to insulin stimulus | 3.27E-04 |
|  | GOTERM_BP_3 | GO:0006629~lipid metabolic process | 3.31E-04 |
|  | GOTERM_BP_5 | GO:0045923~positive regulation of fatty acid metabolic process | 3.72E-04 |
|  | GOTERM_MF_2 | GO:0016564~transcription repressor activity | 3.87E-04 |
|  | GOTERM_BP_5 | GO:0055007~cardiac muscle cell differentiation | 4.48E-04 |
|  | GOTERM_BP_3 | GO:0009887~organ morphogenesis | 5.16E-04 |
|  | GOTERM_BP_4 | GO:0009887~organ morphogenesis | 5.69E-04 |
|  | GOTERM_MF_2 | GO:0005515~protein binding | 5.72E-04 |
|  | GOTERM_BP_4 | GO:0035051~cardiac cell differentiation | 5.81E-04 |
|  | GOTERM_BP_1 | GO:0050896~response to stimulus | 6.03E-04 |
|  | GOTERM_BP_2 | GO:0007275~multicellular organismal development | 6.14E-04 |
|  | GOTERM_BP_5 | GO:0035051~cardiac cell differentiation | 6.66E-04 |
|  | GOTERM_BP_5 | GO:0009887~organ morphogenesis | 7.80E-04 |
|  | GOTERM_BP_3 | GO:0008285~negative regulation of cell proliferation | 9.17E-04 |
|  | GOTERM_BP_4 | GO:0043434~response to peptide hormone stimulus | 9.44E-04 |
|  | GOTERM_BP_4 | GO:0008285~negative regulation of cell proliferation | 9.94E-04 |
|  | GOTERM_BP_1 | GO:0032502~developmental process | 0.001265 |
|  | GOTERM_BP_5 | GO:0008285~negative regulation of cell proliferation | 0.001286 |
|  | GOTERM_MF_3 | GO:0003704~specific RNA polymerase II transcription factor activity | 0.001398 |
|  | GOTERM_BP_3 | GO:0045834~positive regulation of lipid metabolic process | 0.001503 |
|  | GOTERM_BP_4 | GO:0045834~positive regulation of lipid metabolic process | 0.001569 |
|  | GOTERM_BP_4 | GO:0048545~response to steroid hormone stimulus | 0.001783 |
|  | GOTERM_BP_5 | GO:0045834~positive regulation of lipid metabolic process | 0.001798 |
|  | GOTERM_BP_3 | GO:0010942~positive regulation of cell death | 0.001831 |
|  | GOTERM_BP_4 | GO:0043068~positive regulation of programmed cell death | 0.001949 |
|  | GOTERM_BP_4 | GO:0010942~positive regulation of cell death | 0.001982 |
|  | GOTERM_CC_2 | GO:0044424~intracellular part | 0.002034 |
|  | GOTERM_BP_5 | GO:0019217~regulation of fatty acid metabolic process | 0.002038 |
|  | GOTERM_CC_3 | GO:0044424~intracellular part | 0.002059 |
|  | GOTERM_BP_4 | GO:0008202~steroid metabolic process | 0.002061 |
|  | GOTERM_MF_3 | GO:0033293~monocarboxylic acid binding | 0.002196 |
|  | GOTERM_BP_5 | GO:0043065~positive regulation of apoptosis | 0.002446 |
|  | GOTERM_BP_5 | GO:0008202~steroid metabolic process | 0.002508 |
|  | GOTERM_BP_5 | GO:0043068~positive regulation of programmed cell death | 0.002508 |
|  | GOTERM_BP_5 | GO:0010942~positive regulation of cell death | 0.002551 |
|  | GOTERM_BP_4 | GO:0001890~placenta development | 0.002655 |
|  | GOTERM_BP_3 | GO:0010941~regulation of cell death | 0.002696 |
|  | GOTERM_BP_5 | GO:0048738~cardiac muscle tissue development | 0.002748 |
|  | GOTERM_BP_3 | GO:0045595~regulation of cell differentiation | 0.002869 |
|  | GOTERM_BP_4 | GO:0043067~regulation of programmed cell death | 0.002917 |
|  | GOTERM_BP_4 | GO:0010941~regulation of cell death | 0.002964 |
|  | GOTERM_BP_5 | GO:0001890~placenta development | 0.00304 |
|  | GOTERM_BP_4 | GO:0045595~regulation of cell differentiation | 0.003103 |
|  | GOTERM_CC_2 | GO:0005622~intracellular | 0.003647 |
|  | GOTERM_CC_3 | GO:0005622~intracellular | 0.003692 |
|  | GOTERM_BP_5 | GO:0042981~regulation of apoptosis | 0.003776 |
|  | GOTERM_BP_2 | GO:0009605~response to external stimulus | 0.003813 |
|  | GOTERM_BP_5 | GO:0043067~regulation of programmed cell death | 0.003941 |
|  | GOTERM_CC_4 | GO:0044424~intracellular part | 0.004141 |
|  | GOTERM_MF_3 | GO:0005102~receptor binding | 0.004436 |
|  | GOTERM_BP_3 | GO:0010891~negative regulation of sequestering of triglyceride | 0.005042 |
|  | GOTERM_BP_4 | GO:0010891~negative regulation of sequestering of triglyceride | 0.005152 |
|  | GOTERM_BP_4 | GO:0016070~RNA metabolic process | 0.005442 |
|  | GOTERM_BP_5 | GO:0010891~negative regulation of sequestering of triglyceride | 0.005521 |
|  | GOTERM_BP_5 | GO:0051146~striated muscle cell differentiation | 0.006427 |
|  | GOTERM_BP_5 | GO:0010871~negative regulation of receptor biosynthetic process | 0.006897 |
|  | GOTERM_BP_5 | GO:0016070~RNA metabolic process | 0.007299 |
|  | GOTERM_BP_1 | GO:0009987~cellular process | 0.007526 |
|  | GOTERM_BP_2 | GO:0050793~regulation of developmental process | 0.007776 |
|  | GOTERM_BP_3 | GO:0050793~regulation of developmental process | 0.008789 |
|  | GOTERM_BP_3 | GO:0060419~heart growth | 0.008808 |
|  | GOTERM_BP_3 | GO:0055017~cardiac muscle tissue growth | 0.008808 |
|  | GOTERM_BP_4 | GO:0055017~cardiac muscle tissue growth | 0.009 |
|  | GOTERM_BP_4 | GO:0014855~striated muscle cell proliferation | 0.009 |
|  | GOTERM_BP_4 | GO:0010889~regulation of sequestering of triglyceride | 0.009 |
|  | GOTERM_BP_5 | GO:0043627~response to estrogen stimulus | 0.009041 |
|  | GOTERM_BP_5 | GO:0060038~cardiac muscle cell proliferation | 0.009643 |
|  | GOTERM_BP_5 | GO:0010889~regulation of sequestering of triglyceride | 0.009643 |
|  | GOTERM_BP_5 | GO:0060419~heart growth | 0.009643 |
|  | GOTERM_BP_4 | GO:0046321~positive regulation of fatty acid oxidation | 0.01028 |
|  | GOTERM_BP_4 | GO:0042692~muscle cell differentiation | 0.010398 |
|  | GOTERM_BP_5 | GO:0046321~positive regulation of fatty acid oxidation | 0.011014 |
|  | GOTERM_BP_4 | GO:0060537~muscle tissue development | 0.011066 |
|  | GOTERM_BP_3 | GO:0033002~muscle cell proliferation | 0.011311 |
|  | GOTERM_BP_5 | GO:0014706~striated muscle tissue development | 0.011495 |
|  | GOTERM_MF_2 | GO:0031406~carboxylic acid binding | 0.011581 |
|  | GOTERM_MF_5 | GO:0003690~double-stranded DNA binding | 0.011612 |
|  | GOTERM_BP_2 | GO:0009653~anatomical structure morphogenesis | 0.01192 |
|  | GOTERM_BP_3 | GO:0032870~cellular response to hormone stimulus | 0.011955 |
|  | GOTERM_BP_5 | GO:0010869~regulation of receptor biosynthetic process | 0.012382 |
|  | GOTERM_BP_4 | GO:0032870~cellular response to hormone stimulus | 0.012458 |
|  | GOTERM_BP_5 | GO:0060537~muscle tissue development | 0.012627 |
|  | GOTERM_BP_3 | GO:0009653~anatomical structure morphogenesis | 0.013764 |
|  | GOTERM_BP_1 | GO:0032501~multicellular organismal process | 0.014162 |
|  | GOTERM_CC_4 | GO:0030424~axon | 0.014273 |
|  | GOTERM_CC_5 | GO:0030424~axon | 0.014727 |
|  | GOTERM_BP_3 | GO:0042127~regulation of cell proliferation | 0.014966 |
|  | GOTERM_BP_2 | GO:0051716~cellular response to stimulus | 0.01527 |
|  | GOTERM_BP_4 | GO:0042127~regulation of cell proliferation | 0.016103 |
|  | GOTERM_BP_5 | GO:0048048~embryonic eye morphogenesis | 0.016478 |
|  | GOTERM_BP_2 | GO:0035265~organ growth | 0.01694 |
|  | GOTERM_MF_4 | GO:0043566~structure-specific DNA binding | 0.018152 |
|  | GOTERM_BP_1 | GO:0040007~growth | 0.020179 |
|  | GOTERM_BP_5 | GO:0033280~response to vitamin D | 0.021913 |
|  | GOTERM_BP_2 | GO:0065009~regulation of molecular function | 0.026608 |
|  | GOTERM_BP_2 | GO:0065008~regulation of biological quality | 0.027068 |
|  | GOTERM_BP_3 | GO:0008361~regulation of cell size | 0.027248 |
|  | GOTERM_BP_4 | GO:0007517~muscle organ development | 0.029648 |
|  | GOTERM_BP_3 | GO:0045596~negative regulation of cell differentiation | 0.029744 |
|  | GOTERM_BP_4 | GO:0007507~heart development | 0.030693 |
|  | GOTERM_BP_4 | GO:0045596~negative regulation of cell differentiation | 0.030956 |
|  | GOTERM_MF_2 | GO:0003702~RNA polymerase II transcription factor activity | 0.031186 |
|  | GOTERM_BP_3 | GO:0045597~positive regulation of cell differentiation | 0.033122 |
|  | GOTERM_BP_5 | GO:0007517~muscle organ development | 0.033673 |
|  | GOTERM_BP_4 | GO:0045597~positive regulation of cell differentiation | 0.034465 |
|  | GOTERM_BP_5 | GO:0007507~heart development | 0.034852 |
|  | GOTERM_BP_5 | GO:0045596~negative regulation of cell differentiation | 0.03515 |
|  | GOTERM_BP_5 | GO:0035137~hindlimb morphogenesis | 0.035379 |
|  | GOTERM_BP_4 | GO:0046320~regulation of fatty acid oxidation | 0.035545 |
|  | GOTERM_BP_4 | GO:0015908~fatty acid transport | 0.035545 |
|  | GOTERM_BP_5 | GO:0046320~regulation of fatty acid oxidation | 0.038051 |
|  | GOTERM_BP_5 | GO:0015908~fatty acid transport | 0.038051 |
|  | GOTERM_BP_2 | GO:0051093~negative regulation of developmental process | 0.038902 |
|  | GOTERM_BP_5 | GO:0045597~positive regulation of cell differentiation | 0.039106 |
|  | GOTERM_MF_1 | GO:0005488~binding | 0.040249 |
|  | GOTERM_BP_3 | GO:0051093~negative regulation of developmental process | 0.041465 |
|  | GOTERM_BP_4 | GO:0051093~negative regulation of developmental process | 0.043125 |
|  | GOTERM_BP_2 | GO:0051094~positive regulation of developmental process | 0.04423 |
|  | GOTERM_BP_3 | GO:0032535~regulation of cellular component size | 0.045006 |
|  | GOTERM_BP_4 | GO:0048565~gut development | 0.04548 |
|  | GOTERM_BP_3 | GO:0001818~negative regulation of cytokine production | 0.046942 |
|  | GOTERM_BP_3 | GO:0051094~positive regulation of developmental process | 0.047122 |
|  | GOTERM_BP_4 | GO:0001818~negative regulation of cytokine production | 0.047949 |
|  | GOTERM_BP_5 | GO:0048565~gut development | 0.04867 |
|  | GOTERM_BP_4 | GO:0051094~positive regulation of developmental process | 0.048994 |
|  | GOTERM_BP_4 | GO:0006109~regulation of carbohydrate metabolic process | 0.049181 |
| 4287 | GOTERM_CC_2 | GO:0031012~extracellular matrix | 1.13E-32 |
|  | GOTERM_CC_3 | GO:0031012~extracellular matrix | 1.14E-32 |
|  | GOTERM_CC_1 | GO:0044421~extracellular region part | 1.91E-32 |
|  | GOTERM_CC_4 | GO:0005578~proteinaceous extracellular matrix | 4.59E-32 |
|  | GOTERM_CC_2 | GO:0044421~extracellular region part | 5.74E-32 |
|  | GOTERM_CC_3 | GO:0005578~proteinaceous extracellular matrix | 2.79E-31 |
|  | GOTERM_MF_2 | GO:0005201~extracellular matrix structural constituent | 2.39E-29 |
|  | GOTERM_CC_4 | GO:0044420~extracellular matrix part | 2.59E-27 |
|  | GOTERM_CC_2 | GO:0044420~extracellular matrix part | 6.28E-27 |
|  | GOTERM_CC_3 | GO:0044420~extracellular matrix part | 6.35E-27 |
|  | GOTERM_CC_1 | GO:0005576~extracellular region | 1.94E-25 |
|  | GOTERM_CC_5 | GO:0005604~basement membrane | 3.71E-20 |
|  | GOTERM_CC_5 | GO:0005581~collagen | 4.61E-19 |
|  | GOTERM_CC_4 | GO:0005604~basement membrane | 9.16E-19 |
|  | GOTERM_CC_3 | GO:0005604~basement membrane | 1.47E-18 |
|  | GOTERM_MF_1 | GO:0005198~structural molecule activity | 2.73E-18 |
|  | GOTERM_CC_4 | GO:0005581~collagen | 5.46E-18 |
|  | GOTERM_CC_3 | GO:0005581~collagen | 7.79E-18 |
|  | GOTERM_CC_5 | GO:0005587~collagen type IV | 3.03E-14 |
|  | GOTERM_CC_4 | GO:0005587~collagen type IV | 1.04E-13 |
|  | GOTERM_CC_5 | GO:0030935~sheet-forming collagen | 1.06E-13 |
|  | GOTERM_CC_4 | GO:0030935~sheet-forming collagen | 3.64E-13 |
|  | GOTERM_CC_2 | GO:0005615~extracellular space | 1.54E-10 |
|  | GOTERM_CC_3 | GO:0005615~extracellular space | 1.56E-10 |
|  | GOTERM_MF_5 | GO:0004175~endopeptidase activity | 2.58E-09 |
|  | GOTERM_MF_4 | GO:0048407~platelet-derived growth factor binding | 3.62E-09 |
|  | GOTERM_BP_3 | GO:0030198~extracellular matrix organization | 2.89E-08 |
|  | GOTERM_BP_4 | GO:0030155~regulation of cell adhesion | 1.31E-07 |
|  | GOTERM_MF_5 | GO:0004252~serine-type endopeptidase activity | 1.33E-07 |
|  | GOTERM_BP_3 | GO:0030155~regulation of cell adhesion | 1.50E-07 |
|  | GOTERM_BP_4 | GO:0001944~vasculature development | 2.20E-07 |
|  | GOTERM_BP_5 | GO:0001568~blood vessel development | 2.97E-07 |
|  | GOTERM_MF_5 | GO:0008236~serine-type peptidase activity | 3.15E-07 |
|  | GOTERM_BP_3 | GO:0051239~regulation of multicellular organismal process | 3.15E-07 |
|  | GOTERM_BP_5 | GO:0001944~vasculature development | 3.50E-07 |
|  | GOTERM_BP_2 | GO:0051239~regulation of multicellular organismal process | 3.60E-07 |
|  | GOTERM_BP_3 | GO:0009653~anatomical structure morphogenesis | 4.02E-07 |
|  | GOTERM_MF_3 | GO:0008233~peptidase activity | 4.18E-07 |
|  | GOTERM_BP_2 | GO:0043062~extracellular structure organization | 4.41E-07 |
|  | GOTERM_MF_4 | GO:0070011~peptidase activity, acting on L-amino acid peptides | 4.44E-07 |
|  | GOTERM_MF_5 | GO:0005509~calcium ion binding | 4.55E-07 |
|  | GOTERM_BP_2 | GO:0009653~anatomical structure morphogenesis | 4.69E-07 |
|  | GOTERM_BP_3 | GO:0051917~regulation of fibrinolysis | 4.72E-07 |
|  | GOTERM_BP_2 | GO:0051917~regulation of fibrinolysis | 4.80E-07 |
|  | GOTERM_BP_4 | GO:0030193~regulation of blood coagulation | 4.80E-07 |
|  | GOTERM_BP_5 | GO:0030193~regulation of blood coagulation | 6.31E-07 |
|  | GOTERM_CC_5 | GO:0005583~fibrillar collagen | 6.71E-07 |
|  | GOTERM_MF_3 | GO:0017171~serine hydrolase activity | 6.93E-07 |
|  | GOTERM_BP_4 | GO:0050818~regulation of coagulation | 8.20E-07 |
|  | GOTERM_BP_3 | GO:0050818~regulation of coagulation | 8.94E-07 |
|  | GOTERM_MF_4 | GO:0008236~serine-type peptidase activity | 8.95E-07 |
|  | GOTERM_BP_4 | GO:0042060~wound healing | 9.30E-07 |
|  | GOTERM_BP_4 | GO:0007596~blood coagulation | 9.53E-07 |
|  | GOTERM_BP_3 | GO:0007596~blood coagulation | 1.07E-06 |
|  | GOTERM_BP_2 | GO:0050817~coagulation | 1.10E-06 |
|  | GOTERM_BP_4 | GO:0001501~skeletal system development | 1.11E-06 |
|  | GOTERM_MF_3 | GO:0019838~growth factor binding | 1.15E-06 |
|  | GOTERM_BP_4 | GO:0007599~hemostasis | 1.27E-06 |
|  | GOTERM_BP_4 | GO:0010810~regulation of cell-substrate adhesion | 1.31E-06 |
|  | GOTERM_BP_5 | GO:0007596~blood coagulation | 1.34E-06 |
|  | GOTERM_CC_4 | GO:0005583~fibrillar collagen | 1.35E-06 |
|  | GOTERM_BP_3 | GO:0007599~hemostasis | 1.42E-06 |
|  | GOTERM_BP_5 | GO:0010810~regulation of cell-substrate adhesion | 1.73E-06 |
|  | GOTERM_BP_4 | GO:0048513~organ development | 2.12E-06 |
|  | GOTERM_BP_2 | GO:0009605~response to external stimulus | 2.82E-06 |
|  | GOTERM_BP_3 | GO:0048513~organ development | 3.04E-06 |
|  | GOTERM_BP_3 | GO:0050878~regulation of body fluid levels | 5.27E-06 |
|  | GOTERM_BP_2 | GO:0050878~regulation of body fluid levels | 5.46E-06 |
|  | GOTERM_BP_2 | GO:0048856~anatomical structure development | 7.33E-06 |
|  | GOTERM_BP_4 | GO:0016477~cell migration | 7.85E-06 |
|  | GOTERM_BP_4 | GO:0032101~regulation of response to external stimulus | 8.49E-06 |
|  | GOTERM_BP_3 | GO:0016477~cell migration | 8.97E-06 |
|  | GOTERM_BP_4 | GO:0016525~negative regulation of angiogenesis | 9.19E-06 |
|  | GOTERM_BP_4 | GO:0030195~negative regulation of blood coagulation | 9.19E-06 |
|  | GOTERM_BP_1 | GO:0032501~multicellular organismal process | 9.25E-06 |
|  | GOTERM_BP_3 | GO:0032101~regulation of response to external stimulus | 9.47E-06 |
|  | GOTERM_BP_3 | GO:0016525~negative regulation of angiogenesis | 9.78E-06 |
|  | GOTERM_BP_5 | GO:0016525~negative regulation of angiogenesis | 1.13E-05 |
|  | GOTERM_BP_5 | GO:0030195~negative regulation of blood coagulation | 1.13E-05 |
|  | GOTERM_BP_5 | GO:0016477~cell migration | 1.16E-05 |
|  | GOTERM_BP_4 | GO:0050819~negative regulation of coagulation | 1.34E-05 |
|  | GOTERM_BP_3 | GO:0050819~negative regulation of coagulation | 1.43E-05 |
|  | GOTERM_BP_4 | GO:0048870~cell motility | 1.44E-05 |
|  | GOTERM_BP_3 | GO:0048870~cell motility | 1.64E-05 |
|  | GOTERM_BP_5 | GO:0050819~negative regulation of coagulation | 1.65E-05 |
|  | GOTERM_BP_4 | GO:0032963~collagen metabolic process | 1.69E-05 |
|  | GOTERM_BP_2 | GO:0048870~cell motility | 1.72E-05 |
|  | GOTERM_BP_2 | GO:0051674~localization of cell | 1.72E-05 |
|  | GOTERM_BP_2 | GO:0006950~response to stress | 1.76E-05 |
|  | GOTERM_BP_4 | GO:0030199~collagen fibril organization | 1.88E-05 |
|  | GOTERM_BP_4 | GO:0051919~positive regulation of fibrinolysis | 1.90E-05 |
|  | GOTERM_BP_3 | GO:0051919~positive regulation of fibrinolysis | 1.98E-05 |
|  | GOTERM_BP_1 | GO:0032502~developmental process | 1.99E-05 |
|  | GOTERM_BP_2 | GO:0051919~positive regulation of fibrinolysis | 2.00E-05 |
|  | GOTERM_CC_5 | GO:0001527~microfibril | 2.21E-05 |
|  | GOTERM_BP_3 | GO:0044259~multicellular organismal macromolecule metabolic process | 2.46E-05 |
|  | GOTERM_BP_1 | GO:0022610~biological adhesion | 2.58E-05 |
|  | GOTERM_BP_2 | GO:0007155~cell adhesion | 2.70E-05 |
|  | GOTERM_CC_4 | GO:0001527~microfibril | 3.50E-05 |
|  | GOTERM_BP_2 | GO:0007275~multicellular organismal development | 3.58E-05 |
|  | GOTERM_BP_3 | GO:0009611~response to wounding | 3.65E-05 |
|  | GOTERM_BP_2 | GO:0044236~multicellular organismal metabolic process | 4.29E-05 |
|  | GOTERM_BP_4 | GO:0001503~ossification | 5.12E-05 |
|  | GOTERM_BP_2 | GO:0001503~ossification | 5.70E-05 |
|  | GOTERM_CC_5 | GO:0043205~fibril | 6.18E-05 |
|  | GOTERM_BP_3 | GO:0060348~bone development | 7.23E-05 |
|  | GOTERM_BP_3 | GO:0048731~system development | 7.90E-05 |
|  | GOTERM_BP_5 | GO:0060348~bone development | 8.69E-05 |
|  | GOTERM_BP_4 | GO:0001666~response to hypoxia | 9.29E-05 |
|  | GOTERM_BP_5 | GO:0006508~proteolysis | 9.68E-05 |
|  | GOTERM_CC_4 | GO:0043205~fibril | 9.77E-05 |
|  | GOTERM_BP_3 | GO:0001666~response to hypoxia | 1.01E-04 |
|  | GOTERM_CC_3 | GO:0043205~fibril | 1.04E-04 |
|  | GOTERM_BP_1 | GO:0040011~locomotion | 1.10E-04 |
|  | GOTERM_BP_4 | GO:0080134~regulation of response to stress | 1.15E-04 |
|  | GOTERM_BP_3 | GO:0070482~response to oxygen levels | 1.23E-04 |
|  | GOTERM_BP_3 | GO:0080134~regulation of response to stress | 1.28E-04 |
|  | GOTERM_BP_4 | GO:0009888~tissue development | 1.32E-04 |
|  | GOTERM_BP_4 | GO:0030194~positive regulation of blood coagulation | 1.42E-04 |
|  | GOTERM_BP_3 | GO:0009888~tissue development | 1.53E-04 |
|  | GOTERM_BP_5 | GO:0030194~positive regulation of blood coagulation | 1.63E-04 |
|  | GOTERM_BP_3 | GO:0048583~regulation of response to stimulus | 1.66E-04 |
|  | GOTERM_BP_3 | GO:0050793~regulation of developmental process | 1.66E-04 |
|  | GOTERM_BP_4 | GO:0010812~negative regulation of cell-substrate adhesion | 1.73E-04 |
|  | GOTERM_BP_2 | GO:0048583~regulation of response to stimulus | 1.74E-04 |
|  | GOTERM_BP_2 | GO:0050793~regulation of developmental process | 1.75E-04 |
|  | GOTERM_BP_2 | GO:0065008~regulation of biological quality | 1.77E-04 |
|  | GOTERM_BP_3 | GO:0048519~negative regulation of biological process | 1.85E-04 |
|  | GOTERM_BP_3 | GO:0006928~cell motion | 1.87E-04 |
|  | GOTERM_BP_2 | GO:0006928~cell motion | 1.95E-04 |
|  | GOTERM_BP_4 | GO:0045765~regulation of angiogenesis | 1.96E-04 |
|  | GOTERM_MF_4 | GO:0005178~integrin binding | 1.97E-04 |
|  | GOTERM_BP_5 | GO:0010812~negative regulation of cell-substrate adhesion | 1.99E-04 |
|  | GOTERM_BP_5 | GO:0009888~tissue development | 2.02E-04 |
|  | GOTERM_BP_4 | GO:0051241~negative regulation of multicellular organismal process | 2.03E-04 |
|  | GOTERM_BP_2 | GO:0048519~negative regulation of biological process | 2.05E-04 |
|  | GOTERM_BP_3 | GO:0045765~regulation of angiogenesis | 2.09E-04 |
|  | GOTERM_BP_3 | GO:0051241~negative regulation of multicellular organismal process | 2.20E-04 |
|  | GOTERM_BP_2 | GO:0051241~negative regulation of multicellular organismal process | 2.25E-04 |
|  | GOTERM_BP_5 | GO:0042730~fibrinolysis | 2.38E-04 |
|  | GOTERM_BP_5 | GO:0045765~regulation of angiogenesis | 2.40E-04 |
|  | GOTERM_BP_4 | GO:0050820~positive regulation of coagulation | 2.45E-04 |
|  | GOTERM_BP_3 | GO:0050820~positive regulation of coagulation | 2.55E-04 |
|  | GOTERM_BP_5 | GO:0050820~positive regulation of coagulation | 2.81E-04 |
|  | GOTERM_BP_3 | GO:0040012~regulation of locomotion | 4.01E-04 |
|  | GOTERM_BP_2 | GO:0040012~regulation of locomotion | 4.11E-04 |
|  | GOTERM_BP_4 | GO:0019538~protein metabolic process | 4.22E-04 |
|  | GOTERM_BP_2 | GO:0009628~response to abiotic stimulus | 5.19E-04 |
|  | GOTERM_BP_4 | GO:0007160~cell-matrix adhesion | 5.44E-04 |
|  | GOTERM_BP_3 | GO:0019538~protein metabolic process | 5.68E-04 |
|  | GOTERM_BP_4 | GO:0022603~regulation of anatomical structure morphogenesis | 6.08E-04 |
|  | GOTERM_BP_3 | GO:0022603~regulation of anatomical structure morphogenesis | 6.59E-04 |
|  | GOTERM_BP_3 | GO:0031589~cell-substrate adhesion | 7.66E-04 |
|  | GOTERM_BP_4 | GO:0010811~positive regulation of cell-substrate adhesion | 0.001168 |
|  | GOTERM_MF_2 | GO:0050840~extracellular matrix binding | 0.001258 |
|  | GOTERM_BP_5 | GO:0010811~positive regulation of cell-substrate adhesion | 0.001339 |
|  | GOTERM_BP_5 | GO:0043588~skin development | 0.001436 |
|  | GOTERM_BP_1 | GO:0050896~response to stimulus | 0.001466 |
|  | GOTERM_BP_3 | GO:0048518~positive regulation of biological process | 0.002231 |
|  | GOTERM_BP_2 | GO:0048518~positive regulation of biological process | 0.002406 |
|  | GOTERM_BP_4 | GO:0007162~negative regulation of cell adhesion | 0.002744 |
|  | GOTERM_BP_3 | GO:0007162~negative regulation of cell adhesion | 0.002853 |
|  | GOTERM_CC_5 | GO:0005584~collagen type I | 0.003048 |
|  | GOTERM_BP_4 | GO:0009887~organ morphogenesis | 0.003051 |
|  | GOTERM_BP_1 | GO:0016043~cellular component organization | 0.003072 |
|  | GOTERM_BP_5 | GO:0007162~negative regulation of cell adhesion | 0.003142 |
|  | GOTERM_BP_3 | GO:0009887~organ morphogenesis | 0.003355 |
|  | GOTERM_BP_4 | GO:0030334~regulation of cell migration | 0.003443 |
|  | GOTERM_BP_4 | GO:0048646~anatomical structure formation involved in morphogenesis | 0.003487 |
|  | GOTERM_BP_4 | GO:0060346~bone trabecula formation | 0.00364 |
|  | GOTERM_BP_3 | GO:0030334~regulation of cell migration | 0.003648 |
|  | GOTERM_BP_4 | GO:0048522~positive regulation of cellular process | 0.003674 |
|  | GOTERM_BP_3 | GO:0048646~anatomical structure formation involved in morphogenesis | 0.003762 |
|  | GOTERM_BP_2 | GO:0048646~anatomical structure formation involved in morphogenesis | 0.003844 |
|  | GOTERM_BP_5 | GO:0060346~bone trabecula formation | 0.003901 |
|  | GOTERM_BP_5 | GO:0009887~organ morphogenesis | 0.004108 |
|  | GOTERM_BP_4 | GO:0048729~tissue morphogenesis | 0.004111 |
|  | GOTERM_BP_5 | GO:0030334~regulation of cell migration | 0.004179 |
|  | GOTERM_BP_4 | GO:0042476~odontogenesis | 0.004296 |
|  | GOTERM_BP_3 | GO:0048522~positive regulation of cellular process | 0.004347 |
|  | GOTERM_BP_3 | GO:0048729~tissue morphogenesis | 0.004354 |
|  | GOTERM_BP_4 | GO:0008544~epidermis development | 0.004371 |
|  | GOTERM_BP_2 | GO:0048522~positive regulation of cellular process | 0.004618 |
|  | GOTERM_BP_3 | GO:0009612~response to mechanical stimulus | 0.004795 |
|  | GOTERM_BP_5 | GO:0042476~odontogenesis | 0.004914 |
|  | GOTERM_BP_4 | GO:0048545~response to steroid hormone stimulus | 0.004923 |
|  | GOTERM_BP_5 | GO:0048729~tissue morphogenesis | 0.004984 |
|  | GOTERM_BP_4 | GO:0051270~regulation of cell motion | 0.004995 |
|  | GOTERM_BP_4 | GO:0045785~positive regulation of cell adhesion | 0.005279 |
|  | GOTERM_BP_3 | GO:0051270~regulation of cell motion | 0.005288 |
|  | GOTERM_BP_5 | GO:0008544~epidermis development | 0.005299 |
|  | GOTERM_MF_3 | GO:0032403~protein complex binding | 0.005351 |
|  | GOTERM_BP_4 | GO:0007398~ectoderm development | 0.005439 |
|  | GOTERM_BP_4 | GO:0040013~negative regulation of locomotion | 0.005451 |
|  | GOTERM_BP_3 | GO:0045785~positive regulation of cell adhesion | 0.005486 |
|  | GOTERM_BP_3 | GO:0040013~negative regulation of locomotion | 0.005665 |
|  | GOTERM_BP_2 | GO:0040013~negative regulation of locomotion | 0.005718 |
|  | GOTERM_MF_2 | GO:0005515~protein binding | 0.005843 |
|  | GOTERM_BP_5 | GO:0045785~positive regulation of cell adhesion | 0.006035 |
|  | GOTERM_BP_4 | GO:0048514~blood vessel morphogenesis | 0.006397 |
|  | GOTERM_BP_5 | GO:0007398~ectoderm development | 0.006584 |
|  | GOTERM_BP_2 | GO:0042221~response to chemical stimulus | 0.007499 |
|  | GOTERM_BP_5 | GO:0048514~blood vessel morphogenesis | 0.007736 |
|  | GOTERM_BP_5 | GO:0043589~skin morphogenesis | 0.007788 |
|  | GOTERM_BP_4 | GO:0051216~cartilage development | 0.007931 |
|  | GOTERM_BP_5 | GO:0051216~cartilage development | 0.009058 |
|  | GOTERM_BP_4 | GO:0051895~negative regulation of focal adhesion formation | 0.009077 |
|  | GOTERM_BP_4 | GO:0032964~collagen biosynthetic process | 0.009077 |
|  | GOTERM_BP_3 | GO:0001957~intramembranous ossification | 0.009252 |
|  | GOTERM_BP_3 | GO:0051895~negative regulation of focal adhesion formation | 0.009252 |
|  | GOTERM_BP_3 | GO:0010033~response to organic substance | 0.009437 |
|  | GOTERM_BP_4 | GO:0051240~positive regulation of multicellular organismal process | 0.009529 |
|  | GOTERM_BP_5 | GO:0032964~collagen biosynthetic process | 0.009726 |
|  | GOTERM_BP_5 | GO:0051895~negative regulation of focal adhesion formation | 0.009726 |
|  | GOTERM_BP_5 | GO:0001957~intramembranous ossification | 0.009726 |
|  | GOTERM_BP_3 | GO:0051240~positive regulation of multicellular organismal process | 0.010074 |
|  | GOTERM_BP_2 | GO:0051240~positive regulation of multicellular organismal process | 0.010221 |
|  | GOTERM_CC_5 | GO:0005606~laminin-1 complex | 0.010628 |
|  | GOTERM_BP_4 | GO:0051918~negative regulation of fibrinolysis | 0.010883 |
|  | GOTERM_BP_3 | GO:0051918~negative regulation of fibrinolysis | 0.011092 |
|  | GOTERM_BP_2 | GO:0051918~negative regulation of fibrinolysis | 0.011137 |
|  | GOTERM_BP_4 | GO:0051093~negative regulation of developmental process | 0.011204 |
|  | GOTERM_BP_5 | GO:0070613~regulation of protein processing | 0.01166 |
|  | GOTERM_BP_5 | GO:0060351~cartilage development involved in endochondral bone morphogenesis | 0.01166 |
|  | GOTERM_BP_3 | GO:0051093~negative regulation of developmental process | 0.011839 |
|  | GOTERM_BP_2 | GO:0051093~negative regulation of developmental process | 0.012009 |
|  | GOTERM_BP_4 | GO:0042127~regulation of cell proliferation | 0.012348 |
|  | GOTERM_BP_4 | GO:0034446~substrate adhesion-dependent cell spreading | 0.012686 |
|  | GOTERM_MF_5 | GO:0008201~heparin binding | 0.013023 |
|  | GOTERM_BP_4 | GO:0001822~kidney development | 0.013068 |
|  | GOTERM_CC_4 | GO:0005606~laminin-1 complex | 0.013289 |
|  | GOTERM_CC_4 | GO:0005577~fibrinogen complex | 0.013289 |
|  | GOTERM_BP_3 | GO:0042127~regulation of cell proliferation | 0.013471 |
|  | GOTERM_BP_5 | GO:0034446~substrate adhesion-dependent cell spreading | 0.013591 |
|  | GOTERM_CC_5 | GO:0043256~laminin complex | 0.013645 |
|  | GOTERM_CC_2 | GO:0005577~fibrinogen complex | 0.013653 |
|  | GOTERM_CC_3 | GO:0005577~fibrinogen complex | 0.013663 |
|  | GOTERM_BP_4 | GO:0035107~appendage morphogenesis | 0.013856 |
|  | GOTERM_BP_4 | GO:0035108~limb morphogenesis | 0.013856 |
|  | GOTERM_BP_3 | GO:0035107~appendage morphogenesis | 0.014385 |
|  | GOTERM_BP_4 | GO:0051893~regulation of focal adhesion formation | 0.014485 |
|  | GOTERM_BP_4 | GO:0060343~trabecula formation | 0.014485 |
|  | GOTERM_BP_3 | GO:0060343~trabecula formation | 0.014763 |
|  | GOTERM_BP_3 | GO:0051893~regulation of focal adhesion formation | 0.014763 |
|  | GOTERM_BP_5 | GO:0001822~kidney development | 0.014899 |
|  | GOTERM_BP_4 | GO:0060173~limb development | 0.014938 |
|  | GOTERM_BP_5 | GO:0002526~acute inflammatory response | 0.015493 |
|  | GOTERM_BP_3 | GO:0048736~appendage development | 0.015507 |
|  | GOTERM_BP_5 | GO:0060343~trabecula formation | 0.015518 |
|  | GOTERM_BP_5 | GO:0035108~limb morphogenesis | 0.015793 |
|  | GOTERM_BP_4 | GO:0060325~face morphogenesis | 0.016282 |
|  | GOTERM_BP_3 | GO:0060325~face morphogenesis | 0.016594 |
|  | GOTERM_BP_4 | GO:0001655~urogenital system development | 0.016916 |
|  | GOTERM_CC_4 | GO:0043256~laminin complex | 0.017054 |
|  | GOTERM_BP_5 | GO:0001738~morphogenesis of a polarized epithelium | 0.017441 |
|  | GOTERM_BP_5 | GO:0060325~face morphogenesis | 0.017441 |
|  | GOTERM_BP_4 | GO:0048705~skeletal system morphogenesis | 0.017501 |
|  | GOTERM_CC_3 | GO:0043256~laminin complex | 0.017533 |
|  | GOTERM_BP_4 | GO:0048598~embryonic morphogenesis | 0.017656 |
|  | GOTERM_BP_2 | GO:0050789~regulation of biological process | 0.018356 |
|  | GOTERM_BP_3 | GO:0048598~embryonic morphogenesis | 0.018632 |
|  | GOTERM_BP_5 | GO:0001953~negative regulation of cell-matrix adhesion | 0.019361 |
|  | GOTERM_BP_3 | GO:0009790~embryonic development | 0.019594 |
|  | GOTERM_BP_4 | GO:0060323~head morphogenesis | 0.019865 |
|  | GOTERM_BP_4 | GO:0060324~face development | 0.019865 |
|  | GOTERM_BP_5 | GO:0016485~protein processing | 0.019929 |
|  | GOTERM_BP_5 | GO:0048705~skeletal system morphogenesis | 0.019929 |
|  | GOTERM_BP_2 | GO:0009790~embryonic development | 0.019967 |
|  | GOTERM_BP_3 | GO:0060323~head morphogenesis | 0.020245 |
|  | GOTERM_BP_3 | GO:0060324~face development | 0.020245 |
|  | GOTERM_BP_4 | GO:0006954~inflammatory response | 0.020516 |
|  | GOTERM_MF_3 | GO:0005102~receptor binding | 0.020542 |
|  | GOTERM_BP_4 | GO:0051604~protein maturation | 0.020553 |
|  | GOTERM_BP_5 | GO:0060323~head morphogenesis | 0.021278 |
|  | GOTERM_BP_3 | GO:0001958~endochondral ossification | 0.022066 |
|  | GOTERM_BP_5 | GO:0001958~endochondral ossification | 0.02319 |
|  | GOTERM_BP_5 | GO:0051604~protein maturation | 0.023386 |
|  | GOTERM_BP_4 | GO:0045995~regulation of embryonic development | 0.023436 |
|  | GOTERM_BP_3 | GO:0045995~regulation of embryonic development | 0.023884 |
|  | GOTERM_BP_3 | GO:0032879~regulation of localization | 0.024749 |
|  | GOTERM_BP_2 | GO:0032879~regulation of localization | 0.025207 |
|  | GOTERM_BP_4 | GO:0010171~body morphogenesis | 0.025217 |
|  | GOTERM_BP_3 | GO:0006952~defense response | 0.025413 |
|  | GOTERM_CC_5 | GO:0005605~basal lamina | 0.025624 |
|  | GOTERM_BP_3 | GO:0010171~body morphogenesis | 0.025698 |
|  | GOTERM_BP_3 | GO:0060322~head development | 0.025698 |
|  | GOTERM_MF_2 | GO:0016787~hydrolase activity | 0.026013 |
|  | GOTERM_BP_5 | GO:0010596~negative regulation of endothelial cell migration | 0.027005 |
|  | GOTERM_BP_3 | GO:0044087~regulation of cellular component biogenesis | 0.028282 |
|  | GOTERM_BP_2 | GO:0044087~regulation of cellular component biogenesis | 0.028525 |
|  | GOTERM_BP_4 | GO:0060350~endochondral bone morphogenesis | 0.02877 |
|  | GOTERM_BP_4 | GO:0001525~angiogenesis | 0.029436 |
|  | GOTERM_BP_3 | GO:0009725~response to hormone stimulus | 0.029641 |
|  | GOTERM_MF_4 | GO:0005539~glycosaminoglycan binding | 0.030174 |
|  | GOTERM_CC_5 | GO:0030141~secretory granule | 0.030363 |
|  | GOTERM_BP_3 | GO:0001525~angiogenesis | 0.030525 |
|  | GOTERM_BP_5 | GO:0043535~regulation of blood vessel endothelial cell migration | 0.030805 |
|  | GOTERM_BP_5 | GO:0060350~endochondral bone morphogenesis | 0.030805 |
|  | GOTERM_BP_1 | GO:0065007~biological regulation | 0.031776 |
|  | GOTERM_CC_4 | GO:0005605~basal lamina | 0.031978 |
|  | GOTERM_MF_3 | GO:0030247~polysaccharide binding | 0.032219 |
|  | GOTERM_CC_3 | GO:0005605~basal lamina | 0.032868 |
|  | GOTERM_BP_5 | GO:0001525~angiogenesis | 0.033425 |
|  | GOTERM_BP_4 | GO:0030574~collagen catabolic process | 0.035837 |
|  | GOTERM_BP_4 | GO:0060349~bone morphogenesis | 0.035837 |
|  | GOTERM_MF_2 | GO:0001871~pattern binding | 0.036241 |
|  | GOTERM_BP_3 | GO:0060349~bone morphogenesis | 0.036516 |
|  | GOTERM_MF_5 | GO:0008237~metallopeptidase activity | 0.03812 |
|  | GOTERM_BP_5 | GO:0030574~collagen catabolic process | 0.038363 |
|  | GOTERM_BP_5 | GO:0060349~bone morphogenesis | 0.038363 |
|  | GOTERM_BP_2 | GO:0009719~response to endogenous stimulus | 0.03856 |
|  | GOTERM_BP_4 | GO:0007044~cell-substrate junction assembly | 0.041105 |
|  | GOTERM_MF_3 | GO:0016504~peptidase activator activity | 0.041485 |
|  | GOTERM_BP_4 | GO:0048730~epidermis morphogenesis | 0.044602 |
|  | GOTERM_BP_4 | GO:0010594~regulation of endothelial cell migration | 0.044602 |
|  | GOTERM_BP_3 | GO:0044243~multicellular organismal catabolic process | 0.047219 |
|  | GOTERM_BP_5 | GO:0048730~epidermis morphogenesis | 0.047731 |
|  | GOTERM_BP_5 | GO:0010594~regulation of endothelial cell migration | 0.047731 |
|  | GOTERM_CC_5 | GO:0016023~cytoplasmic membrane-bounded vesicle | 0.049543 |
| 4945 | GOTERM_CC_4 | GO:0005905~coated pit | 2.45E-09 |
|  | GOTERM_CC_5 | GO:0005905~coated pit | 2.62E-09 |
|  | GOTERM_BP_5 | GO:0006897~endocytosis | 1.04E-08 |
|  | GOTERM_BP_3 | GO:0006897~endocytosis | 2.28E-08 |
|  | GOTERM_BP_3 | GO:0010324~membrane invagination | 2.28E-08 |
|  | GOTERM_BP_4 | GO:0006897~endocytosis | 2.54E-08 |
|  | GOTERM_BP_2 | GO:0016044~membrane organization | 2.98E-07 |
|  | GOTERM_MF_3 | GO:0008034~lipoprotein binding | 6.14E-07 |
|  | GOTERM_MF_4 | GO:0034189~very-low-density lipoprotein binding | 1.82E-06 |
|  | GOTERM_BP_2 | GO:0016192~vesicle-mediated transport | 2.32E-06 |
|  | GOTERM_MF_5 | GO:0030229~very-low-density lipoprotein receptor activity | 2.54E-06 |
|  | GOTERM_BP_3 | GO:0016192~vesicle-mediated transport | 2.76E-06 |
|  | GOTERM_BP_4 | GO:0016192~vesicle-mediated transport | 3.07E-06 |
|  | GOTERM_MF_5 | GO:0005509~calcium ion binding | 3.93E-06 |
|  | GOTERM_BP_5 | GO:0043549~regulation of kinase activity | 1.00E-05 |
|  | GOTERM_MF_3 | GO:0034185~apolipoprotein binding | 1.11E-05 |
|  | GOTERM_BP_4 | GO:0051338~regulation of transferase activity | 2.05E-05 |
|  | GOTERM_CC_1 | GO:0005576~extracellular region | 2.52E-05 |
|  | GOTERM_MF_4 | GO:0030228~lipoprotein receptor activity | 3.17E-05 |
|  | GOTERM_BP_5 | GO:0019220~regulation of phosphate metabolic process | 3.37E-05 |
|  | GOTERM_BP_5 | GO:0051174~regulation of phosphorus metabolic process | 3.37E-05 |
|  | GOTERM_BP_5 | GO:0030900~forebrain development | 3.61E-05 |
|  | GOTERM_MF_5 | GO:0030228~lipoprotein receptor activity | 4.43E-05 |
|  | GOTERM_BP_3 | GO:0030900~forebrain development | 4.78E-05 |
|  | GOTERM_BP_4 | GO:0051174~regulation of phosphorus metabolic process | 5.81E-05 |
|  | GOTERM_BP_4 | GO:0007399~nervous system development | 6.94E-05 |
|  | GOTERM_CC_2 | GO:0005615~extracellular space | 1.25E-04 |
|  | GOTERM_CC_3 | GO:0005615~extracellular space | 1.26E-04 |
|  | GOTERM_BP_5 | GO:0051347~positive regulation of transferase activity | 1.41E-04 |
|  | GOTERM_MF_2 | GO:0043167~ion binding | 1.56E-04 |
|  | GOTERM_BP_1 | GO:0016043~cellular component organization | 1.86E-04 |
|  | GOTERM_CC_2 | GO:0012505~endomembrane system | 2.10E-04 |
|  | GOTERM_CC_3 | GO:0012505~endomembrane system | 2.10E-04 |
|  | GOTERM_BP_1 | GO:0032501~multicellular organismal process | 2.42E-04 |
|  | GOTERM_BP_5 | GO:0007420~brain development | 2.44E-04 |
|  | GOTERM_BP_4 | GO:0007420~brain development | 3.43E-04 |
|  | GOTERM_MF_3 | GO:0043169~cation binding | 3.44E-04 |
|  | GOTERM_CC_1 | GO:0044421~extracellular region part | 3.98E-04 |
|  | GOTERM_BP_3 | GO:0006629~lipid metabolic process | 3.99E-04 |
|  | GOTERM_BP_2 | GO:0007275~multicellular organismal development | 4.32E-04 |
|  | GOTERM_CC_2 | GO:0044421~extracellular region part | 4.63E-04 |
|  | GOTERM_BP_3 | GO:0050790~regulation of catalytic activity | 4.65E-04 |
|  | GOTERM_BP_1 | GO:0051179~localization | 5.33E-04 |
|  | GOTERM_MF_4 | GO:0046872~metal ion binding | 6.63E-04 |
|  | GOTERM_BP_2 | GO:0065009~regulation of molecular function | 6.84E-04 |
|  | GOTERM_BP_1 | GO:0032502~developmental process | 7.13E-04 |
|  | GOTERM_BP_5 | GO:0007417~central nervous system development | 7.59E-04 |
|  | GOTERM_CC_3 | GO:0044459~plasma membrane part | 0.001009 |
|  | GOTERM_BP_4 | GO:0007417~central nervous system development | 0.001059 |
|  | GOTERM_CC_4 | GO:0044459~plasma membrane part | 0.001225 |
|  | GOTERM_CC_3 | GO:0005886~plasma membrane | 0.001254 |
|  | GOTERM_CC_5 | GO:0044459~plasma membrane part | 0.001324 |
|  | GOTERM_CC_4 | GO:0005886~plasma membrane | 0.001586 |
|  | GOTERM_CC_3 | GO:0031988~membrane-bounded vesicle | 0.001598 |
|  | GOTERM_CC_4 | GO:0016023~cytoplasmic membrane-bounded vesicle | 0.001639 |
|  | GOTERM_CC_5 | GO:0016023~cytoplasmic membrane-bounded vesicle | 0.001719 |
|  | GOTERM_BP_4 | GO:0043085~positive regulation of catalytic activity | 0.0019 |
|  | GOTERM_CC_3 | GO:0031410~cytoplasmic vesicle | 0.002275 |
|  | GOTERM_BP_3 | GO:0048731~system development | 0.002383 |
|  | GOTERM_BP_3 | GO:0044093~positive regulation of molecular function | 0.002518 |
|  | GOTERM_CC_4 | GO:0031410~cytoplasmic vesicle | 0.002559 |
|  | GOTERM_CC_2 | GO:0031982~vesicle | 0.002566 |
|  | GOTERM_CC_5 | GO:0031410~cytoplasmic vesicle | 0.002684 |
|  | GOTERM_CC_2 | GO:0042995~cell projection | 0.002874 |
|  | GOTERM_CC_3 | GO:0042995~cell projection | 0.00288 |
|  | GOTERM_BP_2 | GO:0048856~anatomical structure development | 0.002952 |
|  | GOTERM_BP_2 | GO:0006810~transport | 0.003548 |
|  | GOTERM_BP_1 | GO:0051234~establishment of localization | 0.003592 |
|  | GOTERM_BP_2 | GO:0051234~establishment of localization | 0.00372 |
|  | GOTERM_BP_3 | GO:0006810~transport | 0.004174 |
|  | GOTERM_CC_3 | GO:0005626~insoluble fraction | 0.004884 |
|  | GOTERM_CC_4 | GO:0005624~membrane fraction | 0.004948 |
|  | GOTERM_CC_5 | GO:0005624~membrane fraction | 0.005186 |
|  | GOTERM_CC_4 | GO:0005626~insoluble fraction | 0.005485 |
|  | GOTERM_BP_5 | GO:0021795~cerebral cortex cell migration | 0.006816 |
|  | GOTERM_BP_4 | GO:0021795~cerebral cortex cell migration | 0.007417 |
|  | GOTERM_MF_5 | GO:0005041~low-density lipoprotein receptor activity | 0.007709 |
|  | GOTERM_BP_5 | GO:0022029~telencephalon cell migration | 0.007786 |
|  | GOTERM_BP_5 | GO:0021885~forebrain cell migration | 0.008271 |
|  | GOTERM_BP_4 | GO:0034381~lipoprotein particle clearance | 0.008473 |
|  | GOTERM_BP_4 | GO:0022029~telencephalon cell migration | 0.008473 |
|  | GOTERM_CC_4 | GO:0005768~endosome | 0.00899 |
|  | GOTERM_BP_4 | GO:0021885~forebrain cell migration | 0.009 |
|  | GOTERM_CC_5 | GO:0005768~endosome | 0.009281 |
|  | GOTERM_MF_3 | GO:0004872~receptor activity | 0.009618 |
|  | GOTERM_CC_3 | GO:0043005~neuron projection | 0.009734 |
|  | GOTERM_CC_2 | GO:0000267~cell fraction | 0.009983 |
|  | GOTERM_CC_3 | GO:0000267~cell fraction | 0.010003 |
|  | GOTERM_CC_3 | GO:0031224~intrinsic to membrane | 0.010342 |
|  | GOTERM_CC_4 | GO:0043005~neuron projection | 0.010534 |
|  | GOTERM_CC_4 | GO:0016021~integral to membrane | 0.010704 |
|  | GOTERM_CC_5 | GO:0016021~integral to membrane | 0.011734 |
|  | GOTERM_MF_1 | GO:0060089~molecular transducer activity | 0.012075 |
|  | GOTERM_MF_4 | GO:0030169~low-density lipoprotein binding | 0.012423 |
|  | GOTERM_CC_4 | GO:0031224~intrinsic to membrane | 0.012987 |
|  | GOTERM_MF_2 | GO:0004871~signal transducer activity | 0.01301 |
|  | GOTERM_CC_5 | GO:0031224~intrinsic to membrane | 0.014231 |
|  | GOTERM_CC_5 | GO:0005887~integral to plasma membrane | 0.015144 |
|  | GOTERM_MF_2 | GO:0005515~protein binding | 0.01521 |
|  | GOTERM_CC_4 | GO:0031226~intrinsic to plasma membrane | 0.015392 |
|  | GOTERM_CC_2 | GO:0032994~protein-lipid complex | 0.015913 |
|  | GOTERM_CC_2 | GO:0034358~plasma lipoprotein particle | 0.015913 |
|  | GOTERM_CC_3 | GO:0034358~plasma lipoprotein particle | 0.015924 |
|  | GOTERM_BP_5 | GO:0021987~cerebral cortex development | 0.016004 |
|  | GOTERM_CC_5 | GO:0031226~intrinsic to plasma membrane | 0.016106 |
|  | GOTERM_CC_4 | GO:0034358~plasma lipoprotein particle | 0.016587 |
|  | GOTERM_BP_2 | GO:0009653~anatomical structure morphogenesis | 0.016966 |
|  | GOTERM_BP_3 | GO:0021987~cerebral cortex development | 0.017036 |
|  | GOTERM_BP_4 | GO:0021987~cerebral cortex development | 0.017407 |
|  | GOTERM_BP_3 | GO:0009653~anatomical structure morphogenesis | 0.018673 |
|  | GOTERM_BP_2 | GO:0007610~behavior | 0.021187 |
|  | GOTERM_BP_2 | GO:0006928~cell motion | 0.021702 |
|  | GOTERM_BP_5 | GO:0021543~pallium development | 0.022249 |
|  | GOTERM_BP_3 | GO:0006928~cell motion | 0.023189 |
|  | GOTERM_BP_3 | GO:0021543~pallium development | 0.023679 |
|  | GOTERM_BP_4 | GO:0021543~pallium development | 0.024193 |
|  | GOTERM_CC_4 | GO:0005901~caveola | 0.024559 |
|  | GOTERM_CC_5 | GO:0005901~caveola | 0.024964 |
|  | GOTERM_BP_5 | GO:0006464~protein modification process | 0.025019 |
|  | GOTERM_BP_5 | GO:0006898~receptor-mediated endocytosis | 0.025598 |
|  | GOTERM_BP_4 | GO:0006898~receptor-mediated endocytosis | 0.02783 |
|  | GOTERM_CC_2 | GO:0044425~membrane part | 0.027949 |
|  | GOTERM_CC_3 | GO:0044425~membrane part | 0.028058 |
|  | GOTERM_CC_5 | GO:0030139~endocytic vesicle | 0.028284 |
|  | GOTERM_BP_2 | GO:0065008~regulation of biological quality | 0.029489 |
|  | GOTERM_BP_3 | GO:0021537~telencephalon development | 0.033318 |
|  | GOTERM_BP_4 | GO:0021537~telencephalon development | 0.034038 |
|  | GOTERM_CC_4 | GO:0044425~membrane part | 0.035021 |
|  | GOTERM_BP_4 | GO:0043412~biopolymer modification | 0.037914 |
|  | GOTERM_BP_3 | GO:0019538~protein metabolic process | 0.038645 |
|  | GOTERM_BP_4 | GO:0019538~protein metabolic process | 0.041608 |
|  | GOTERM_BP_4 | GO:0042157~lipoprotein metabolic process | 0.041751 |
|  | GOTERM_BP_1 | GO:0051704~multi-organism process | 0.041877 |
|  | GOTERM_BP_1 | GO:0022610~biological adhesion | 0.044161 |
|  | GOTERM_BP_2 | GO:0007155~cell adhesion | 0.044661 |
|  | GOTERM_CC_2 | GO:0016020~membrane | 0.047676 |
|  | GOTERM_CC_3 | GO:0016020~membrane | 0.047858 |
|  | GOTERM_BP_4 | GO:0008203~cholesterol metabolic process | 0.047883 |
|  | GOTERM_BP_5 | GO:0016125~sterol metabolic process | 0.048308 |
| 2305 | GOTERM_MF_5 | GO:0019957~C-C chemokine binding | 0.001607 |
|  | GOTERM_BP_3 | GO:0019059~initiation of viral infection | 0.001634 |
|  | GOTERM_BP_4 | GO:0019059~initiation of viral infection | 0.00167 |
|  | GOTERM_MF_4 | GO:0015026~coreceptor activity | 0.001699 |
|  | GOTERM_BP_5 | GO:0019059~initiation of viral infection | 0.00179 |
|  | GOTERM_MF_4 | GO:0019956~chemokine binding | 0.002293 |
|  | GOTERM_MF_5 | GO:0004950~chemokine receptor activity | 0.002511 |
|  | GOTERM_BP_3 | GO:0019058~viral infectious cycle | 0.003194 |
|  | GOTERM_BP_4 | GO:0019058~viral infectious cycle | 0.003264 |
|  | GOTERM_BP_2 | GO:0022415~viral reproductive process | 0.004446 |
|  | GOTERM_BP_3 | GO:0022415~viral reproductive process | 0.004605 |
|  | GOTERM_BP_1 | GO:0016032~viral reproduction | 0.005053 |
|  | GOTERM_MF_3 | GO:0019955~cytokine binding | 0.008352 |
|  | GOTERM_MF_3 | GO:0001653~peptide receptor activity | 0.008735 |
|  | GOTERM_MF_4 | GO:0008528~peptide receptor activity, G-protein coupled | 0.009683 |
|  | GOTERM_MF_4 | GO:0001653~peptide receptor activity | 0.009683 |
|  | GOTERM_MF_5 | GO:0008528~peptide receptor activity, G-protein coupled | 0.011448 |
|  | GOTERM_BP_2 | GO:0042330~taxis | 0.011474 |
|  | GOTERM_BP_3 | GO:0006935~chemotaxis | 0.011884 |
|  | GOTERM_BP_3 | GO:0042330~taxis | 0.011884 |
|  | GOTERM_BP_4 | GO:0006935~chemotaxis | 0.012145 |
|  | GOTERM_BP_4 | GO:0042330~taxis | 0.012145 |
|  | GOTERM_BP_5 | GO:0006935~chemotaxis | 0.013017 |
|  | GOTERM_MF_2 | GO:0042277~peptide binding | 0.013693 |
|  | GOTERM_BP_2 | GO:0044419~interspecies interaction between organisms | 0.020294 |
|  | GOTERM_BP_3 | GO:0007626~locomotory behavior | 0.020352 |
|  | GOTERM_BP_5 | GO:0030003~cellular cation homeostasis | 0.020664 |
|  | GOTERM_BP_4 | GO:0006954~inflammatory response | 0.02467 |
|  | GOTERM_MF_4 | GO:0003779~actin binding | 0.02769 |
|  | GOTERM_BP_3 | GO:0055082~cellular chemical homeostasis | 0.028226 |
|  | GOTERM_BP_4 | GO:0006873~cellular ion homeostasis | 0.028389 |
|  | GOTERM_BP_1 | GO:0040011~locomotion | 0.030674 |
|  | GOTERM_BP_5 | GO:0055082~cellular chemical homeostasis | 0.030914 |
|  | GOTERM_BP_5 | GO:0050801~ion homeostasis | 0.033274 |
|  | GOTERM_BP_2 | GO:0019725~cellular homeostasis | 0.033417 |
|  | GOTERM_BP_2 | GO:0007610~behavior | 0.033632 |
|  | GOTERM_BP_4 | GO:0019725~cellular homeostasis | 0.035373 |
|  | GOTERM_MF_3 | GO:0008092~cytoskeletal protein binding | 0.038618 |
|  | GOTERM_BP_4 | GO:0048878~chemical homeostasis | 0.038864 |
|  | GOTERM_BP_3 | GO:0009611~response to wounding | 0.039367 |
|  | GOTERM_BP_3 | GO:0006952~defense response | 0.045681 |
|  | GOTERM_BP_1 | GO:0051704~multi-organism process | 0.048466 |
|  | GOTERM_BP_2 | GO:0006955~immune response | 0.04948 |
| 2437 | GOTERM_MF_5 | GO:0034062~RNA polymerase activity | 5.11E-05 |
|  | GOTERM_CC_4 | GO:0005730~nucleolus | 5.49E-05 |
|  | GOTERM_CC_5 | GO:0005730~nucleolus | 6.37E-05 |
|  | GOTERM_CC_5 | GO:0005736~DNA-directed RNA polymerase I complex | 8.84E-05 |
|  | GOTERM_MF_1 | GO:0005488~binding | 1.58E-04 |
|  | GOTERM_MF_2 | GO:0043167~ion binding | 6.61E-04 |
|  | GOTERM_CC_4 | GO:0005634~nucleus | 0.001149 |
|  | GOTERM_MF_4 | GO:0046872~metal ion binding | 0.001227 |
|  | GOTERM_CC_5 | GO:0005634~nucleus | 0.001564 |
|  | GOTERM_MF_3 | GO:0043169~cation binding | 0.001648 |
|  | GOTERM_MF_4 | GO:0016779~nucleotidyltransferase activity | 0.002059 |
|  | GOTERM_CC_3 | GO:0070013~intracellular organelle lumen | 0.002108 |
|  | GOTERM_CC_4 | GO:0070013~intracellular organelle lumen | 0.002175 |
|  | GOTERM_MF_5 | GO:0046914~transition metal ion binding | 0.002537 |
|  | GOTERM_CC_5 | GO:0070013~intracellular organelle lumen | 0.002562 |
|  | GOTERM_CC_1 | GO:0031974~membrane-enclosed lumen | 0.002588 |
|  | GOTERM_CC_2 | GO:0043233~organelle lumen | 0.002622 |
|  | GOTERM_CC_3 | GO:0043233~organelle lumen | 0.00264 |
|  | GOTERM_CC_4 | GO:0031981~nuclear lumen | 0.002685 |
|  | GOTERM_CC_2 | GO:0005622~intracellular | 0.002967 |
|  | GOTERM_CC_3 | GO:0005622~intracellular | 0.003042 |
|  | GOTERM_CC_1 | GO:0043226~organelle | 0.003104 |
|  | GOTERM_CC_5 | GO:0031981~nuclear lumen | 0.003105 |
|  | GOTERM_CC_4 | GO:0000428~DNA-directed RNA polymerase complex | 0.00406 |
|  | GOTERM_CC_5 | GO:0055029~nuclear DNA-directed RNA polymerase complex | 0.004192 |
|  | GOTERM_CC_5 | GO:0000428~DNA-directed RNA polymerase complex | 0.004192 |
|  | GOTERM_CC_3 | GO:0030880~RNA polymerase complex | 0.004339 |
|  | GOTERM_CC_4 | GO:0030880~RNA polymerase complex | 0.004387 |
|  | GOTERM_CC_4 | GO:0043229~intracellular organelle | 0.004391 |
|  | GOTERM_CC_5 | GO:0030880~RNA polymerase complex | 0.00453 |
|  | GOTERM_CC_4 | GO:0044452~nucleolar part | 0.004727 |
|  | GOTERM_CC_2 | GO:0043229~intracellular organelle | 0.00475 |
|  | GOTERM_CC_3 | GO:0043229~intracellular organelle | 0.004839 |
|  | GOTERM_CC_5 | GO:0044452~nucleolar part | 0.004881 |
|  | GOTERM_CC_4 | GO:0044424~intracellular part | 0.005753 |
|  | GOTERM_CC_2 | GO:0043228~non-membrane-bounded organelle | 0.00604 |
|  | GOTERM_CC_3 | GO:0043232~intracellular non-membrane-bounded organelle | 0.006087 |
|  | GOTERM_CC_4 | GO:0043232~intracellular non-membrane-bounded organelle | 0.006283 |
|  | GOTERM_CC_5 | GO:0043229~intracellular organelle | 0.006793 |
|  | GOTERM_CC_2 | GO:0044424~intracellular part | 0.006917 |
|  | GOTERM_CC_3 | GO:0044424~intracellular part | 0.00707 |
|  | GOTERM_CC_3 | GO:0044428~nuclear part | 0.007073 |
|  | GOTERM_CC_4 | GO:0044428~nuclear part | 0.007301 |
|  | GOTERM_CC_5 | GO:0043232~intracellular non-membrane-bounded organelle | 0.007486 |
|  | GOTERM_CC_5 | GO:0044428~nuclear part | 0.008429 |
|  | GOTERM_CC_4 | GO:0043231~intracellular membrane-bounded organelle | 0.014444 |
|  | GOTERM_CC_3 | GO:0043231~intracellular membrane-bounded organelle | 0.014596 |
|  | GOTERM_CC_2 | GO:0043227~membrane-bounded organelle | 0.014642 |
|  | GOTERM_BP_4 | GO:0015980~energy derivation by oxidation of organic compounds | 0.020124 |
|  | GOTERM_CC_5 | GO:0043231~intracellular membrane-bounded organelle | 0.020197 |
|  | GOTERM_MF_3 | GO:0016772~transferase activity, transferring phosphorus-containing groups | 0.020623 |
|  | GOTERM_BP_5 | GO:0060037~pharyngeal system development | 0.027023 |
|  | GOTERM_BP_4 | GO:0060037~pharyngeal system development | 0.02783 |
|  | GOTERM_BP_4 | GO:0006350~transcription | 0.034837 |
|  | GOTERM_BP_5 | GO:0031163~metallo-sulfur cluster assembly | 0.038384 |
|  | GOTERM_BP_5 | GO:0032411~positive regulation of transporter activity | 0.038384 |
|  | GOTERM_BP_3 | GO:0032411~positive regulation of transporter activity | 0.039407 |
|  | GOTERM_BP_4 | GO:0032411~positive regulation of transporter activity | 0.039523 |
|  | GOTERM_BP_3 | GO:0009792~embryonic development ending in birth or egg hatching | 0.04462 |
|  | GOTERM_BP_4 | GO:0009792~embryonic development ending in birth or egg hatching | 0.044988 |
|  | GOTERM_CC_1 | GO:0044422~organelle part | 0.048846 |
|  | GOTERM_CC_4 | GO:0005654~nucleoplasm | 0.049542 |
| 2923 | GOTERM_MF_5 | GO:0042379~chemokine receptor binding | 0.004921 |
|  | GOTERM_MF_4 | GO:0001664~G-protein-coupled receptor binding | 0.009428 |
|  | GOTERM_BP_2 | GO:0042330~taxis | 0.011474 |
|  | GOTERM_BP_3 | GO:0042330~taxis | 0.011884 |
|  | GOTERM_BP_3 | GO:0006935~chemotaxis | 0.011884 |
|  | GOTERM_BP_4 | GO:0006935~chemotaxis | 0.012145 |
|  | GOTERM_BP_4 | GO:0042330~taxis | 0.012145 |
|  | GOTERM_BP_5 | GO:0006935~chemotaxis | 0.013017 |
|  | GOTERM_BP_3 | GO:0007626~locomotory behavior | 0.020352 |
|  | GOTERM_BP_5 | GO:0030003~cellular cation homeostasis | 0.020664 |
|  | GOTERM_BP_3 | GO:0055082~cellular chemical homeostasis | 0.028226 |
|  | GOTERM_BP_4 | GO:0006873~cellular ion homeostasis | 0.028389 |
|  | GOTERM_BP_1 | GO:0040011~locomotion | 0.030674 |
|  | GOTERM_BP_5 | GO:0055082~cellular chemical homeostasis | 0.030914 |
|  | GOTERM_BP_5 | GO:0050801~ion homeostasis | 0.033274 |
|  | GOTERM_BP_2 | GO:0019725~cellular homeostasis | 0.033417 |
|  | GOTERM_BP_2 | GO:0007610~behavior | 0.033632 |
|  | GOTERM_BP_4 | GO:0019725~cellular homeostasis | 0.035373 |
|  | GOTERM_BP_4 | GO:0048878~chemical homeostasis | 0.038864 |
|  | GOTERM_BP_2 | GO:0006955~immune response | 0.04948 |
| 3622 | GOTERM_BP_3 | GO:0007631~feeding behavior | 5.14E-07 |
|  | GOTERM_MF_4 | GO:0005179~hormone activity | 7.51E-07 |
|  | GOTERM_BP_3 | GO:0008343~adult feeding behavior | 2.38E-06 |
|  | GOTERM_BP_4 | GO:0008343~adult feeding behavior | 4.14E-06 |
|  | GOTERM_MF_3 | GO:0005102~receptor binding | 9.98E-05 |
|  | GOTERM_BP_2 | GO:0007610~behavior | 1.47E-04 |
|  | GOTERM_BP_2 | GO:0030534~adult behavior | 2.24E-04 |
|  | GOTERM_BP_3 | GO:0030534~adult behavior | 2.40E-04 |
|  | GOTERM_BP_5 | GO:0007186~G-protein coupled receptor protein signaling pathway | 3.21E-04 |
|  | GOTERM_BP_3 | GO:0007267~cell-cell signaling | 3.41E-04 |
|  | GOTERM_BP_2 | GO:0007154~cell communication | 7.07E-04 |
|  | GOTERM_CC_1 | GO:0005576~extracellular region | 0.001143 |
|  | GOTERM_BP_4 | GO:0007166~cell surface receptor linked signal transduction | 0.001743 |
|  | GOTERM_BP_5 | GO:0007166~cell surface receptor linked signal transduction | 0.002279 |
|  | GOTERM_MF_5 | GO:0005184~neuropeptide hormone activity | 0.00231 |
|  | GOTERM_BP_1 | GO:0032501~multicellular organismal process | 0.0086 |
|  | GOTERM_BP_4 | GO:0009755~hormone-mediated signaling | 0.019584 |
|  | GOTERM_BP_5 | GO:0009755~hormone-mediated signaling | 0.020977 |
|  | GOTERM_BP_2 | GO:0007586~digestion | 0.025851 |
|  | GOTERM_BP_3 | GO:0008217~regulation of blood pressure | 0.029385 |
|  | GOTERM_BP_3 | GO:0007165~signal transduction | 0.031586 |
|  | GOTERM_BP_3 | GO:0032870~cellular response to hormone stimulus | 0.038938 |
|  | GOTERM_BP_5 | GO:0008217~regulation of blood pressure | 0.040027 |
|  | GOTERM_CC_4 | GO:0005625~soluble fraction | 0.042224 |
|  | GOTERM_BP_4 | GO:0032870~cellular response to hormone stimulus | 0.049477 |
| 3847 | GOTERM_MF_5 | GO:0015171~amino acid transmembrane transporter activity | 5.54E-11 |
|  | GOTERM_MF_4 | GO:0005275~amine transmembrane transporter activity | 5.88E-10 |
|  | GOTERM_MF_5 | GO:0046943~carboxylic acid transmembrane transporter activity | 8.59E-10 |
|  | GOTERM_BP_4 | GO:0006865~amino acid transport | 1.00E-09 |
|  | GOTERM_BP_5 | GO:0006865~amino acid transport | 1.51E-09 |
|  | GOTERM_BP_3 | GO:0006865~amino acid transport | 1.91E-09 |
|  | GOTERM_MF_4 | GO:0005342~organic acid transmembrane transporter activity | 2.38E-09 |
|  | GOTERM_BP_4 | GO:0015837~amine transport | 5.19E-09 |
|  | GOTERM_BP_3 | GO:0015837~amine transport | 9.86E-09 |
|  | GOTERM_BP_4 | GO:0046942~carboxylic acid transport | 1.95E-08 |
|  | GOTERM_BP_4 | GO:0015849~organic acid transport | 2.03E-08 |
|  | GOTERM_BP_5 | GO:0046942~carboxylic acid transport | 2.93E-08 |
|  | GOTERM_BP_3 | GO:0015849~organic acid transport | 3.84E-08 |
|  | GOTERM_CC_3 | GO:0005886~plasma membrane | 8.31E-08 |
|  | GOTERM_CC_4 | GO:0005886~plasma membrane | 1.54E-07 |
|  | GOTERM_MF_3 | GO:0042605~peptide antigen binding | 9.92E-07 |
|  | GOTERM_BP_4 | GO:0015804~neutral amino acid transport | 2.34E-06 |
|  | GOTERM_BP_5 | GO:0015804~neutral amino acid transport | 2.88E-06 |
|  | GOTERM_BP_4 | GO:0007159~leukocyte adhesion | 5.73E-06 |
|  | GOTERM_MF_3 | GO:0022804~active transmembrane transporter activity | 6.21E-06 |
|  | GOTERM_BP_4 | GO:0019062~virion attachment to host cell surface receptor | 2.34E-05 |
|  | GOTERM_BP_5 | GO:0019062~virion attachment to host cell surface receptor | 2.69E-05 |
|  | GOTERM_BP_3 | GO:0019062~virion attachment to host cell surface receptor | 2.82E-05 |
|  | GOTERM_BP_3 | GO:0016337~cell-cell adhesion | 3.21E-05 |
|  | GOTERM_MF_2 | GO:0003823~antigen binding | 4.00E-05 |
|  | GOTERM_MF_4 | GO:0005178~integrin binding | 7.73E-05 |
|  | GOTERM_BP_4 | GO:0052126~movement in host environment | 8.56E-05 |
|  | GOTERM_BP_4 | GO:0052192~movement in environment of other organism during symbiotic interaction | 8.56E-05 |
|  | GOTERM_BP_4 | GO:0046718~entry of virus into host cell | 8.56E-05 |
|  | GOTERM_BP_4 | GO:0051806~entry into cell of other organism during symbiotic interaction | 8.56E-05 |
|  | GOTERM_BP_4 | GO:0044409~entry into host | 8.56E-05 |
|  | GOTERM_BP_2 | GO:0052192~movement in environment of other organism during symbiotic interaction | 9.60E-05 |
|  | GOTERM_BP_5 | GO:0046718~entry of virus into host cell | 9.83E-05 |
|  | GOTERM_BP_5 | GO:0052126~movement in host environment | 9.83E-05 |
|  | GOTERM_BP_5 | GO:0044409~entry into host | 9.83E-05 |
|  | GOTERM_BP_5 | GO:0030260~entry into host cell | 9.83E-05 |
|  | GOTERM_BP_5 | GO:0051828~entry into other organism during symbiotic interaction | 9.83E-05 |
|  | GOTERM_CC_3 | GO:0044459~plasma membrane part | 1.01E-04 |
|  | GOTERM_BP_3 | GO:0006519~cellular amino acid and derivative metabolic process | 1.02E-04 |
|  | GOTERM_BP_3 | GO:0052126~movement in host environment | 1.03E-04 |
|  | GOTERM_BP_3 | GO:0051828~entry into other organism during symbiotic interaction | 1.03E-04 |
|  | GOTERM_MF_3 | GO:0032403~protein complex binding | 1.28E-04 |
|  | GOTERM_CC_4 | GO:0044459~plasma membrane part | 1.43E-04 |
|  | GOTERM_CC_5 | GO:0044459~plasma membrane part | 1.65E-04 |
|  | GOTERM_CC_2 | GO:0016020~membrane | 1.65E-04 |
|  | GOTERM_CC_3 | GO:0016020~membrane | 1.67E-04 |
|  | GOTERM_BP_4 | GO:0006520~cellular amino acid metabolic process | 1.68E-04 |
|  | GOTERM_BP_5 | GO:0006520~cellular amino acid metabolic process | 2.19E-04 |
|  | GOTERM_BP_1 | GO:0022610~biological adhesion | 2.34E-04 |
|  | GOTERM_BP_2 | GO:0007155~cell adhesion | 2.42E-04 |
|  | GOTERM_MF_1 | GO:0005215~transporter activity | 2.48E-04 |
|  | GOTERM_CC_2 | GO:0044425~membrane part | 2.79E-04 |
|  | GOTERM_CC_3 | GO:0044425~membrane part | 2.82E-04 |
|  | GOTERM_BP_4 | GO:0007596~blood coagulation | 2.83E-04 |
|  | GOTERM_BP_4 | GO:0007599~hemostasis | 3.35E-04 |
|  | GOTERM_BP_2 | GO:0050817~coagulation | 3.38E-04 |
|  | GOTERM_BP_5 | GO:0007596~blood coagulation | 3.47E-04 |
|  | GOTERM_BP_4 | GO:0019059~initiation of viral infection | 3.57E-04 |
|  | GOTERM_BP_4 | GO:0007157~heterophilic cell adhesion | 3.57E-04 |
|  | GOTERM_BP_3 | GO:0007596~blood coagulation | 3.75E-04 |
|  | GOTERM_BP_4 | GO:0015807~L-amino acid transport | 3.90E-04 |
|  | GOTERM_BP_5 | GO:0019059~initiation of viral infection | 4.09E-04 |
|  | GOTERM_BP_3 | GO:0019059~initiation of viral infection | 4.29E-04 |
|  | GOTERM_BP_3 | GO:0007599~hemostasis | 4.43E-04 |
|  | GOTERM_BP_5 | GO:0015807~L-amino acid transport | 4.48E-04 |
|  | GOTERM_CC_4 | GO:0044425~membrane part | 5.00E-04 |
|  | GOTERM_MF_2 | GO:0022857~transmembrane transporter activity | 5.03E-04 |
|  | GOTERM_BP_2 | GO:0044419~interspecies interaction between organisms | 5.06E-04 |
|  | GOTERM_CC_5 | GO:0005887~integral to plasma membrane | 5.25E-04 |
|  | GOTERM_BP_4 | GO:0044106~cellular amine metabolic process | 5.28E-04 |
|  | GOTERM_CC_4 | GO:0031226~intrinsic to plasma membrane | 5.44E-04 |
|  | GOTERM_BP_1 | GO:0051179~localization | 5.46E-04 |
|  | GOTERM_CC_5 | GO:0031226~intrinsic to plasma membrane | 6.02E-04 |
|  | GOTERM_MF_3 | GO:0022891~substrate-specific transmembrane transporter activity | 6.10E-04 |
|  | GOTERM_BP_5 | GO:0044106~cellular amine metabolic process | 6.85E-04 |
|  | GOTERM_MF_2 | GO:0022892~substrate-specific transporter activity | 7.09E-04 |
|  | GOTERM_CC_2 | GO:0009986~cell surface | 7.79E-04 |
|  | GOTERM_CC_3 | GO:0009986~cell surface | 7.81E-04 |
|  | GOTERM_BP_2 | GO:0050878~regulation of body fluid levels | 8.71E-04 |
|  | GOTERM_CC_3 | GO:0031224~intrinsic to membrane | 8.94E-04 |
|  | GOTERM_CC_4 | GO:0016021~integral to membrane | 9.60E-04 |
|  | GOTERM_BP_3 | GO:0050878~regulation of body fluid levels | 9.64E-04 |
|  | GOTERM_BP_4 | GO:0051701~interaction with host | 0.001072 |
|  | GOTERM_CC_3 | GO:0009897~external side of plasma membrane | 0.001149 |
|  | GOTERM_CC_5 | GO:0016021~integral to membrane | 0.001161 |
|  | GOTERM_BP_3 | GO:0051701~interaction with host | 0.001287 |
|  | GOTERM_CC_4 | GO:0009897~external side of plasma membrane | 0.001293 |
|  | GOTERM_CC_5 | GO:0031093~platelet alpha granule lumen | 0.001307 |
|  | GOTERM_CC_5 | GO:0009897~external side of plasma membrane | 0.001356 |
|  | GOTERM_BP_4 | GO:0019058~viral infectious cycle | 0.001372 |
|  | GOTERM_CC_4 | GO:0031224~intrinsic to membrane | 0.001431 |
|  | GOTERM_CC_4 | GO:0060205~cytoplasmic membrane-bounded vesicle lumen | 0.001456 |
|  | GOTERM_CC_3 | GO:0031983~vesicle lumen | 0.001467 |
|  | GOTERM_CC_5 | GO:0060205~cytoplasmic membrane-bounded vesicle lumen | 0.001504 |
|  | GOTERM_BP_3 | GO:0044403~symbiosis, encompassing mutualism through parasitism | 0.001571 |
|  | GOTERM_CC_4 | GO:0031983~vesicle lumen | 0.00159 |
|  | GOTERM_BP_3 | GO:0019058~viral infectious cycle | 0.001646 |
|  | GOTERM_CC_5 | GO:0031224~intrinsic to membrane | 0.001727 |
|  | GOTERM_BP_4 | GO:0042060~wound healing | 0.001756 |
|  | GOTERM_MF_2 | GO:0042277~peptide binding | 0.001774 |
|  | GOTERM_CC_1 | GO:0005576~extracellular region | 0.001972 |
|  | GOTERM_BP_3 | GO:0009308~amine metabolic process | 0.002087 |
|  | GOTERM_BP_4 | GO:0046795~intracellular virion transport | 0.002579 |
|  | GOTERM_BP_4 | GO:0046794~virion transport | 0.002579 |
|  | GOTERM_BP_4 | GO:0046968~peptide antigen transport | 0.002579 |
|  | GOTERM_BP_5 | GO:0046795~intracellular virion transport | 0.002764 |
|  | GOTERM_BP_5 | GO:0046968~peptide antigen transport | 0.002764 |
|  | GOTERM_BP_5 | GO:0046794~virion transport | 0.002764 |
|  | GOTERM_BP_3 | GO:0046794~virion transport | 0.002821 |
|  | GOTERM_BP_2 | GO:0022415~viral reproductive process | 0.003168 |
|  | GOTERM_BP_3 | GO:0022415~viral reproductive process | 0.003393 |
|  | GOTERM_BP_1 | GO:0016032~viral reproduction | 0.004072 |
|  | GOTERM_BP_2 | GO:0006810~transport | 0.004392 |
|  | GOTERM_BP_1 | GO:0051234~establishment of localization | 0.004474 |
|  | GOTERM_BP_2 | GO:0051234~establishment of localization | 0.004718 |
|  | GOTERM_BP_4 | GO:0043436~oxoacid metabolic process | 0.004815 |
|  | GOTERM_BP_2 | GO:0019882~antigen processing and presentation | 0.005604 |
|  | GOTERM_BP_3 | GO:0006810~transport | 0.005613 |
|  | GOTERM_MF_3 | GO:0005102~receptor binding | 0.005804 |
|  | GOTERM_BP_5 | GO:0019752~carboxylic acid metabolic process | 0.006155 |
|  | GOTERM_BP_2 | GO:0001775~cell activation | 0.006548 |
|  | GOTERM_BP_3 | GO:0006082~organic acid metabolic process | 0.006972 |
|  | GOTERM_BP_3 | GO:0022614~membrane to membrane docking | 0.007038 |
|  | GOTERM_MF_2 | GO:0046790~virion binding | 0.007264 |
|  | GOTERM_BP_3 | GO:0042180~cellular ketone metabolic process | 0.007282 |
|  | GOTERM_CC_2 | GO:0005577~fibrinogen complex | 0.008666 |
|  | GOTERM_CC_3 | GO:0005577~fibrinogen complex | 0.008672 |
|  | GOTERM_CC_4 | GO:0005577~fibrinogen complex | 0.009034 |
|  | GOTERM_BP_1 | GO:0051704~multi-organism process | 0.011842 |
|  | GOTERM_BP_1 | GO:0040011~locomotion | 0.019273 |
|  | GOTERM_MF_5 | GO:0005537~mannose binding | 0.019418 |
|  | GOTERM_BP_4 | GO:0019079~viral genome replication | 0.02046 |
|  | GOTERM_BP_5 | GO:0019079~viral genome replication | 0.021913 |
|  | GOTERM_CC_3 | GO:0044433~cytoplasmic vesicle part | 0.022213 |
|  | GOTERM_BP_3 | GO:0019079~viral genome replication | 0.022355 |
|  | GOTERM_CC_5 | GO:0030141~secretory granule | 0.02303 |
|  | GOTERM_MF_3 | GO:0043499~eukaryotic cell surface binding | 0.023204 |
|  | GOTERM_CC_4 | GO:0044433~cytoplasmic vesicle part | 0.023977 |
|  | GOTERM_CC_5 | GO:0044433~cytoplasmic vesicle part | 0.024726 |
|  | GOTERM_BP_4 | GO:0019048~virus-host interaction | 0.025513 |
|  | GOTERM_MF_4 | GO:0016755~transferase activity, transferring amino-acyl groups | 0.027102 |
|  | GOTERM_MF_5 | GO:0008415~acyltransferase activity | 0.027319 |
|  | GOTERM_BP_5 | GO:0019048~virus-host interaction | 0.027321 |
|  | GOTERM_BP_3 | GO:0009988~cell-cell recognition | 0.02787 |
|  | GOTERM_BP_3 | GO:0019048~virus-host interaction | 0.02787 |
|  | GOTERM_BP_3 | GO:0051186~cofactor metabolic process | 0.030362 |
|  | GOTERM_CC_3 | GO:0031988~membrane-bounded vesicle | 0.031764 |
|  | GOTERM_CC_4 | GO:0016023~cytoplasmic membrane-bounded vesicle | 0.032489 |
|  | GOTERM_MF_3 | GO:0016746~transferase activity, transferring acyl groups | 0.03358 |
|  | GOTERM_CC_5 | GO:0016023~cytoplasmic membrane-bounded vesicle | 0.033891 |
|  | GOTERM_MF_4 | GO:0016747~transferase activity, transferring acyl groups other than amino-acyl groups | 0.033946 |
|  | GOTERM_MF_2 | GO:0043498~cell surface binding | 0.034651 |
|  | GOTERM_BP_3 | GO:0009611~response to wounding | 0.036766 |
|  | GOTERM_BP_3 | GO:0048002~antigen processing and presentation of peptide antigen | 0.03881 |
|  | GOTERM_BP_4 | GO:0030168~platelet activation | 0.040525 |
|  | GOTERM_BP_2 | GO:0022406~membrane docking | 0.041429 |
|  | GOTERM_BP_2 | GO:0065008~regulation of biological quality | 0.042656 |
|  | GOTERM_CC_3 | GO:0031410~cytoplasmic vesicle | 0.043311 |
|  | GOTERM_BP_5 | GO:0030168~platelet activation | 0.043374 |
|  | GOTERM_BP_3 | GO:0030168~platelet activation | 0.044237 |
|  | GOTERM_CC_4 | GO:0031410~cytoplasmic vesicle | 0.047974 |
|  | GOTERM_CC_2 | GO:0031982~vesicle | 0.048086 |
|  | GOTERM_CC_5 | GO:0031410~cytoplasmic vesicle | 0.049982 |
| 3853 | GOTERM_CC_3 | GO:0005578~proteinaceous extracellular matrix | 3.36E-18 |
|  | GOTERM_CC_4 | GO:0005578~proteinaceous extracellular matrix | 5.28E-18 |
|  | GOTERM_CC_2 | GO:0031012~extracellular matrix | 7.72E-18 |
|  | GOTERM_CC_3 | GO:0031012~extracellular matrix | 7.78E-18 |
|  | GOTERM_CC_1 | GO:0044421~extracellular region part | 2.19E-15 |
|  | GOTERM_CC_2 | GO:0044421~extracellular region part | 3.51E-15 |
|  | GOTERM_CC_1 | GO:0005576~extracellular region | 1.61E-11 |
|  | GOTERM_BP_5 | GO:0030574~collagen catabolic process | 1.67E-09 |
|  | GOTERM_BP_4 | GO:0030574~collagen catabolic process | 1.90E-09 |
|  | GOTERM_MF_5 | GO:0005540~hyaluronic acid binding | 4.64E-09 |
|  | GOTERM_BP_3 | GO:0044243~multicellular organismal catabolic process | 5.35E-09 |
|  | GOTERM_BP_4 | GO:0032963~collagen metabolic process | 7.99E-09 |
|  | GOTERM_BP_3 | GO:0044259~multicellular organismal macromolecule metabolic process | 1.12E-08 |
|  | GOTERM_BP_2 | GO:0044236~multicellular organismal metabolic process | 2.95E-08 |
|  | GOTERM_MF_5 | GO:0008237~metallopeptidase activity | 2.99E-08 |
|  | GOTERM_MF_5 | GO:0004175~endopeptidase activity | 2.09E-06 |
|  | GOTERM_MF_4 | GO:0070011~peptidase activity, acting on L-amino acid peptides | 7.27E-06 |
|  | GOTERM_MF_2 | GO:0001871~pattern binding | 7.44E-06 |
|  | GOTERM_BP_5 | GO:0006508~proteolysis | 8.10E-06 |
|  | GOTERM_MF_4 | GO:0005539~glycosaminoglycan binding | 8.81E-06 |
|  | GOTERM_MF_3 | GO:0008233~peptidase activity | 9.29E-06 |
|  | GOTERM_MF_3 | GO:0030247~polysaccharide binding | 1.23E-05 |
|  | GOTERM_MF_2 | GO:0030246~carbohydrate binding | 1.93E-04 |
|  | GOTERM_MF_5 | GO:0005509~calcium ion binding | 3.45E-04 |
|  | GOTERM_CC_2 | GO:0005615~extracellular space | 0.00148 |
|  | GOTERM_CC_3 | GO:0005615~extracellular space | 0.001484 |
|  | GOTERM_BP_1 | GO:0022610~biological adhesion | 0.003059 |
|  | GOTERM_BP_2 | GO:0007155~cell adhesion | 0.003128 |
|  | GOTERM_BP_3 | GO:0030198~extracellular matrix organization | 0.003709 |
|  | GOTERM_BP_1 | GO:0032501~multicellular organismal process | 0.004504 |
|  | GOTERM_BP_3 | GO:0019538~protein metabolic process | 0.005029 |
|  | GOTERM_BP_4 | GO:0019538~protein metabolic process | 0.005715 |
|  | GOTERM_MF_2 | GO:0016787~hydrolase activity | 0.008557 |
|  | GOTERM_BP_2 | GO:0043062~extracellular structure organization | 0.009733 |
|  | GOTERM_MF_3 | GO:0005529~sugar binding | 0.015546 |
|  | GOTERM_BP_4 | GO:0030199~collagen fibril organization | 0.026109 |
|  | GOTERM_MF_3 | GO:0005518~collagen binding | 0.035288 |
|  | GOTERM_MF_2 | GO:0043167~ion binding | 0.049067 |
| 3895 | GOTERM_BP_5 | GO:0007596~blood coagulation | 2.11E-09 |
|  | GOTERM_BP_4 | GO:0007596~blood coagulation | 2.14E-09 |
|  | GOTERM_BP_2 | GO:0050817~coagulation | 2.28E-09 |
|  | GOTERM_BP_3 | GO:0007596~blood coagulation | 2.81E-09 |
|  | GOTERM_BP_4 | GO:0007599~hemostasis | 3.03E-09 |
|  | GOTERM_BP_3 | GO:0007599~hemostasis | 3.98E-09 |
|  | GOTERM_BP_2 | GO:0050878~regulation of body fluid levels | 1.61E-08 |
|  | GOTERM_BP_3 | GO:0050878~regulation of body fluid levels | 1.99E-08 |
|  | GOTERM_BP_4 | GO:0042060~wound healing | 9.30E-08 |
|  | GOTERM_CC_5 | GO:0030141~secretory granule | 4.64E-06 |
|  | GOTERM_CC_1 | GO:0005576~extracellular region | 1.69E-05 |
|  | GOTERM_BP_3 | GO:0009611~response to wounding | 4.48E-05 |
|  | GOTERM_BP_2 | GO:0009605~response to external stimulus | 8.52E-05 |
|  | GOTERM_CC_5 | GO:0030667~secretory granule membrane | 1.74E-04 |
|  | GOTERM_CC_5 | GO:0044433~cytoplasmic vesicle part | 2.40E-04 |
|  | GOTERM_CC_5 | GO:0016023~cytoplasmic membrane-bounded vesicle | 3.65E-04 |
|  | GOTERM_CC_4 | GO:0044433~cytoplasmic vesicle part | 5.31E-04 |
|  | GOTERM_MF_4 | GO:0005179~hormone activity | 5.54E-04 |
|  | GOTERM_CC_5 | GO:0031410~cytoplasmic vesicle | 6.58E-04 |
|  | GOTERM_CC_3 | GO:0044433~cytoplasmic vesicle part | 7.36E-04 |
|  | GOTERM_BP_4 | GO:0030168~platelet activation | 7.60E-04 |
|  | GOTERM_BP_5 | GO:0030168~platelet activation | 7.70E-04 |
|  | GOTERM_BP_3 | GO:0030168~platelet activation | 8.18E-04 |
|  | GOTERM_CC_4 | GO:0016023~cytoplasmic membrane-bounded vesicle | 0.001067 |
|  | GOTERM_BP_1 | GO:0022610~biological adhesion | 0.001516 |
|  | GOTERM_BP_2 | GO:0007155~cell adhesion | 0.001558 |
|  | GOTERM_BP_2 | GO:0065008~regulation of biological quality | 0.001583 |
|  | GOTERM_CC_2 | GO:0044421~extracellular region part | 0.001704 |
|  | GOTERM_CC_3 | GO:0031988~membrane-bounded vesicle | 0.001858 |
|  | GOTERM_CC_4 | GO:0031410~cytoplasmic vesicle | 0.001894 |
|  | GOTERM_CC_3 | GO:0031410~cytoplasmic vesicle | 0.002907 |
|  | GOTERM_CC_2 | GO:0031982~vesicle | 0.003384 |
|  | GOTERM_CC_1 | GO:0044421~extracellular region part | 0.003505 |
|  | GOTERM_CC_5 | GO:0030659~cytoplasmic vesicle membrane | 0.003943 |
|  | GOTERM_CC_5 | GO:0012506~vesicle membrane | 0.004635 |
|  | GOTERM_CC_4 | GO:0030659~cytoplasmic vesicle membrane | 0.006494 |
|  | GOTERM_CC_4 | GO:0005886~plasma membrane | 0.006837 |
|  | GOTERM_BP_1 | GO:0032501~multicellular organismal process | 0.006867 |
|  | GOTERM_MF_5 | GO:0004175~endopeptidase activity | 0.006976 |
|  | GOTERM_CC_4 | GO:0012506~vesicle membrane | 0.007623 |
|  | GOTERM_CC_3 | GO:0012506~vesicle membrane | 0.009359 |
|  | GOTERM_MF_5 | GO:0004252~serine-type endopeptidase activity | 0.011926 |
|  | GOTERM_BP_1 | GO:0022414~reproductive process | 0.014274 |
|  | GOTERM_BP_1 | GO:0000003~reproduction | 0.014594 |
|  | GOTERM_BP_2 | GO:0022414~reproductive process | 0.014644 |
|  | GOTERM_MF_5 | GO:0008236~serine-type peptidase activity | 0.015717 |
|  | GOTERM_BP_2 | GO:0006950~response to stress | 0.015802 |
|  | GOTERM_CC_3 | GO:0005886~plasma membrane | 0.016085 |
|  | GOTERM_MF_2 | GO:0001871~pattern binding | 0.016328 |
|  | GOTERM_MF_3 | GO:0030247~polysaccharide binding | 0.018701 |
|  | GOTERM_BP_1 | GO:0050896~response to stimulus | 0.018901 |
|  | GOTERM_MF_4 | GO:0005539~glycosaminoglycan binding | 0.018965 |
|  | GOTERM_MF_3 | GO:0017171~serine hydrolase activity | 0.025039 |
|  | GOTERM_CC_2 | GO:0005615~extracellular space | 0.027207 |
|  | GOTERM_CC_3 | GO:0005615~extracellular space | 0.027258 |
|  | GOTERM_MF_2 | GO:0042277~peptide binding | 0.02738 |
|  | GOTERM_BP_4 | GO:0030195~negative regulation of blood coagulation | 0.029286 |
|  | GOTERM_BP_5 | GO:0030195~negative regulation of blood coagulation | 0.029539 |
|  | GOTERM_MF_4 | GO:0008236~serine-type peptidase activity | 0.029674 |
|  | GOTERM_BP_1 | GO:0065007~biological regulation | 0.029679 |
|  | GOTERM_MF_3 | GO:0005102~receptor binding | 0.030033 |
|  | GOTERM_BP_4 | GO:0050819~negative regulation of coagulation | 0.033046 |
|  | GOTERM_BP_5 | GO:0050819~negative regulation of coagulation | 0.033332 |
|  | GOTERM_BP_3 | GO:0050819~negative regulation of coagulation | 0.034218 |
|  | GOTERM_MF_3 | GO:0008233~peptidase activity | 0.042213 |
|  | GOTERM_BP_5 | GO:0006508~proteolysis | 0.04249 |
|  | GOTERM_BP_4 | GO:0030193~regulation of blood coagulation | 0.04548 |
|  | GOTERM_BP_5 | GO:0030193~regulation of blood coagulation | 0.045872 |
|  | GOTERM_MF_4 | GO:0070011~peptidase activity, acting on L-amino acid peptides | 0.04884 |
|  | GOTERM_MF_3 | GO:0001948~glycoprotein binding | 0.049851 |
| 3903 | GOTERM_BP_5 | GO:0051347~positive regulation of transferase activity | 2.22E-08 |
|  | GOTERM_BP_4 | GO:0007242~intracellular signaling cascade | 2.92E-08 |
|  | GOTERM_BP_3 | GO:0007165~signal transduction | 3.62E-08 |
|  | GOTERM_BP_3 | GO:0032870~cellular response to hormone stimulus | 3.84E-08 |
|  | GOTERM_BP_4 | GO:0032870~cellular response to hormone stimulus | 4.28E-08 |
|  | GOTERM_BP_4 | GO:0007165~signal transduction | 4.60E-08 |
|  | GOTERM_BP_5 | GO:0007242~intracellular signaling cascade | 5.37E-08 |
|  | GOTERM_BP_4 | GO:0043434~response to peptide hormone stimulus | 8.93E-08 |
|  | GOTERM_MF_5 | GO:0043548~phosphoinositide 3-kinase binding | 1.20E-07 |
|  | GOTERM_MF_3 | GO:0005102~receptor binding | 1.69E-07 |
|  | GOTERM_BP_5 | GO:0007167~enzyme linked receptor protein signaling pathway | 1.82E-07 |
|  | GOTERM_BP_4 | GO:0051338~regulation of transferase activity | 2.00E-07 |
|  | GOTERM_BP_5 | GO:0043549~regulation of kinase activity | 2.35E-07 |
|  | GOTERM_BP_4 | GO:0007166~cell surface receptor linked signal transduction | 9.03E-07 |
|  | GOTERM_BP_4 | GO:0051174~regulation of phosphorus metabolic process | 9.53E-07 |
|  | GOTERM_BP_3 | GO:0050790~regulation of catalytic activity | 9.97E-07 |
|  | GOTERM_BP_5 | GO:0051174~regulation of phosphorus metabolic process | 1.43E-06 |
|  | GOTERM_BP_5 | GO:0019220~regulation of phosphate metabolic process | 1.43E-06 |
|  | GOTERM_BP_4 | GO:0043085~positive regulation of catalytic activity | 1.43E-06 |
|  | GOTERM_BP_4 | GO:0009966~regulation of signal transduction | 1.49E-06 |
|  | GOTERM_BP_5 | GO:0007166~cell surface receptor linked signal transduction | 1.65E-06 |
|  | GOTERM_BP_2 | GO:0065009~regulation of molecular function | 1.97E-06 |
|  | GOTERM_BP_5 | GO:0009966~regulation of signal transduction | 2.37E-06 |
|  | GOTERM_BP_3 | GO:0044093~positive regulation of molecular function | 2.54E-06 |
|  | GOTERM_BP_3 | GO:0010646~regulation of cell communication | 3.98E-06 |
|  | GOTERM_BP_5 | GO:0008286~insulin receptor signaling pathway | 4.07E-06 |
|  | GOTERM_BP_4 | GO:0010646~regulation of cell communication | 4.60E-06 |
|  | GOTERM_BP_3 | GO:0009725~response to hormone stimulus | 5.91E-06 |
|  | GOTERM_BP_3 | GO:0045834~positive regulation of lipid metabolic process | 6.04E-06 |
|  | GOTERM_BP_4 | GO:0045834~positive regulation of lipid metabolic process | 6.45E-06 |
|  | GOTERM_BP_5 | GO:0045834~positive regulation of lipid metabolic process | 7.93E-06 |
|  | GOTERM_BP_2 | GO:0009719~response to endogenous stimulus | 8.06E-06 |
|  | GOTERM_BP_5 | GO:0007243~protein kinase cascade | 9.57E-06 |
|  | GOTERM_BP_5 | GO:0030879~mammary gland development | 2.06E-05 |
|  | GOTERM_BP_4 | GO:0032869~cellular response to insulin stimulus | 2.11E-05 |
|  | GOTERM_BP_5 | GO:0032869~cellular response to insulin stimulus | 2.59E-05 |
|  | GOTERM_BP_2 | GO:0009893~positive regulation of metabolic process | 2.83E-05 |
|  | GOTERM_BP_5 | GO:0043550~regulation of lipid kinase activity | 3.26E-05 |
|  | GOTERM_BP_5 | GO:0045725~positive regulation of glycogen biosynthetic process | 3.26E-05 |
|  | GOTERM_BP_3 | GO:0009893~positive regulation of metabolic process | 3.46E-05 |
|  | GOTERM_BP_4 | GO:0009893~positive regulation of metabolic process | 3.91E-05 |
|  | GOTERM_BP_4 | GO:0030335~positive regulation of cell migration | 4.73E-05 |
|  | GOTERM_BP_2 | GO:0040017~positive regulation of locomotion | 5.33E-05 |
|  | GOTERM_BP_5 | GO:0030335~positive regulation of cell migration | 5.81E-05 |
|  | GOTERM_BP_3 | GO:0051272~positive regulation of cell motion | 5.92E-05 |
|  | GOTERM_BP_3 | GO:0040017~positive regulation of locomotion | 5.92E-05 |
|  | GOTERM_MF_4 | GO:0005159~insulin-like growth factor receptor binding | 6.25E-05 |
|  | GOTERM_BP_4 | GO:0051272~positive regulation of cell motion | 6.31E-05 |
|  | GOTERM_BP_4 | GO:0040017~positive regulation of locomotion | 6.31E-05 |
|  | GOTERM_BP_5 | GO:0032885~regulation of polysaccharide biosynthetic process | 6.59E-05 |
|  | GOTERM_BP_5 | GO:0010962~regulation of glucan biosynthetic process | 6.59E-05 |
|  | GOTERM_BP_5 | GO:0005979~regulation of glycogen biosynthetic process | 6.59E-05 |
|  | GOTERM_BP_4 | GO:0032881~regulation of polysaccharide metabolic process | 6.62E-05 |
|  | GOTERM_BP_5 | GO:0032881~regulation of polysaccharide metabolic process | 7.60E-05 |
|  | GOTERM_BP_5 | GO:0051272~positive regulation of cell motion | 7.74E-05 |
|  | GOTERM_BP_5 | GO:0032868~response to insulin stimulus | 8.22E-05 |
|  | GOTERM_BP_4 | GO:0019216~regulation of lipid metabolic process | 9.39E-05 |
|  | GOTERM_MF_3 | GO:0019838~growth factor binding | 1.06E-04 |
|  | GOTERM_BP_3 | GO:0045913~positive regulation of carbohydrate metabolic process | 1.14E-04 |
|  | GOTERM_BP_5 | GO:0019216~regulation of lipid metabolic process | 1.15E-04 |
|  | GOTERM_BP_4 | GO:0010676~positive regulation of cellular carbohydrate metabolic process | 1.19E-04 |
|  | GOTERM_BP_4 | GO:0045913~positive regulation of carbohydrate metabolic process | 1.19E-04 |
|  | GOTERM_BP_5 | GO:0010907~positive regulation of glucose metabolic process | 1.23E-04 |
|  | GOTERM_BP_4 | GO:0043255~regulation of carbohydrate biosynthetic process | 1.32E-04 |
|  | GOTERM_BP_5 | GO:0010676~positive regulation of cellular carbohydrate metabolic process | 1.37E-04 |
|  | GOTERM_BP_5 | GO:0045913~positive regulation of carbohydrate metabolic process | 1.37E-04 |
|  | GOTERM_BP_3 | GO:0010828~positive regulation of glucose transport | 1.39E-04 |
|  | GOTERM_BP_2 | GO:0048522~positive regulation of cellular process | 1.43E-04 |
|  | GOTERM_BP_4 | GO:0046326~positive regulation of glucose import | 1.45E-04 |
|  | GOTERM_BP_4 | GO:0010828~positive regulation of glucose transport | 1.45E-04 |
|  | GOTERM_BP_5 | GO:0043255~regulation of carbohydrate biosynthetic process | 1.51E-04 |
|  | GOTERM_BP_3 | GO:0010033~response to organic substance | 1.53E-04 |
|  | GOTERM_MF_1 | GO:0060089~molecular transducer activity | 1.60E-04 |
|  | GOTERM_BP_4 | GO:0048732~gland development | 1.64E-04 |
|  | GOTERM_BP_5 | GO:0010828~positive regulation of glucose transport | 1.67E-04 |
|  | GOTERM_BP_5 | GO:0046326~positive regulation of glucose import | 1.67E-04 |
|  | GOTERM_BP_3 | GO:0048522~positive regulation of cellular process | 1.80E-04 |
|  | GOTERM_MF_2 | GO:0004871~signal transducer activity | 1.86E-04 |
|  | GOTERM_BP_5 | GO:0048732~gland development | 2.00E-04 |
|  | GOTERM_BP_4 | GO:0048522~positive regulation of cellular process | 2.07E-04 |
|  | GOTERM_BP_2 | GO:0051716~cellular response to stimulus | 2.38E-04 |
|  | GOTERM_BP_2 | GO:0048518~positive regulation of biological process | 2.66E-04 |
|  | GOTERM_MF_3 | GO:0017046~peptide hormone binding | 2.69E-04 |
|  | GOTERM_BP_4 | GO:0043467~regulation of generation of precursor metabolites and energy | 2.72E-04 |
|  | GOTERM_BP_3 | GO:0010941~regulation of cell death | 2.73E-04 |
|  | GOTERM_BP_4 | GO:0043067~regulation of programmed cell death | 2.97E-04 |
|  | GOTERM_BP_3 | GO:0030334~regulation of cell migration | 2.98E-04 |
|  | GOTERM_BP_4 | GO:0010941~regulation of cell death | 3.02E-04 |
|  | GOTERM_BP_5 | GO:0043467~regulation of generation of precursor metabolites and energy | 3.12E-04 |
|  | GOTERM_BP_4 | GO:0030334~regulation of cell migration | 3.17E-04 |
|  | GOTERM_BP_3 | GO:0048518~positive regulation of biological process | 3.34E-04 |
|  | GOTERM_BP_2 | GO:0022607~cellular component assembly | 3.44E-04 |
|  | GOTERM_BP_4 | GO:0010827~regulation of glucose transport | 3.50E-04 |
|  | GOTERM_MF_4 | GO:0005158~insulin receptor binding | 3.55E-04 |
|  | GOTERM_BP_5 | GO:0046324~regulation of glucose import | 3.79E-04 |
|  | GOTERM_BP_2 | GO:0050794~regulation of cellular process | 3.81E-04 |
|  | GOTERM_BP_5 | GO:0030334~regulation of cell migration | 3.88E-04 |
|  | GOTERM_BP_2 | GO:0040012~regulation of locomotion | 3.91E-04 |
|  | GOTERM_BP_5 | GO:0042981~regulation of apoptosis | 3.92E-04 |
|  | GOTERM_BP_4 | GO:0031331~positive regulation of cellular catabolic process | 3.93E-04 |
|  | GOTERM_BP_5 | GO:0010827~regulation of glucose transport | 4.02E-04 |
|  | GOTERM_BP_5 | GO:0043067~regulation of programmed cell death | 4.10E-04 |
|  | GOTERM_MF_2 | GO:0005515~protein binding | 4.20E-04 |
|  | GOTERM_BP_5 | GO:0010906~regulation of glucose metabolic process | 4.26E-04 |
|  | GOTERM_BP_3 | GO:0040012~regulation of locomotion | 4.33E-04 |
|  | GOTERM_BP_4 | GO:0010675~regulation of cellular carbohydrate metabolic process | 4.38E-04 |
|  | GOTERM_BP_3 | GO:0051270~regulation of cell motion | 4.40E-04 |
|  | GOTERM_BP_5 | GO:0031331~positive regulation of cellular catabolic process | 4.51E-04 |
|  | GOTERM_BP_4 | GO:0006109~regulation of carbohydrate metabolic process | 4.62E-04 |
|  | GOTERM_BP_4 | GO:0051270~regulation of cell motion | 4.68E-04 |
|  | GOTERM_BP_5 | GO:0010675~regulation of cellular carbohydrate metabolic process | 5.03E-04 |
|  | GOTERM_BP_5 | GO:0006109~regulation of carbohydrate metabolic process | 5.30E-04 |
|  | GOTERM_BP_3 | GO:0050794~regulation of cellular process | 5.61E-04 |
|  | GOTERM_BP_1 | GO:0044085~cellular component biogenesis | 5.84E-04 |
|  | GOTERM_BP_2 | GO:0050789~regulation of biological process | 5.99E-04 |
|  | GOTERM_MF_3 | GO:0032403~protein complex binding | 6.64E-04 |
|  | GOTERM_MF_4 | GO:0019900~kinase binding | 6.87E-04 |
|  | GOTERM_BP_3 | GO:0009896~positive regulation of catabolic process | 6.99E-04 |
|  | GOTERM_BP_4 | GO:0009896~positive regulation of catabolic process | 7.30E-04 |
|  | GOTERM_MF_2 | GO:0042562~hormone binding | 8.16E-04 |
|  | GOTERM_BP_5 | GO:0009896~positive regulation of catabolic process | 8.37E-04 |
|  | GOTERM_BP_5 | GO:0051291~protein heterooligomerization | 9.42E-04 |
|  | GOTERM_BP_1 | GO:0065007~biological regulation | 9.75E-04 |
|  | GOTERM_BP_4 | GO:0031329~regulation of cellular catabolic process | 0.001129 |
|  | GOTERM_BP_2 | GO:0048519~negative regulation of biological process | 0.00123 |
|  | GOTERM_BP_5 | GO:0031329~regulation of cellular catabolic process | 0.001295 |
|  | GOTERM_MF_4 | GO:0004896~cytokine receptor activity | 0.001373 |
|  | GOTERM_BP_3 | GO:0031323~regulation of cellular metabolic process | 0.001452 |
|  | GOTERM_CC_4 | GO:0005886~plasma membrane | 0.001461 |
|  | GOTERM_BP_3 | GO:0048519~negative regulation of biological process | 0.001485 |
|  | GOTERM_BP_2 | GO:0019222~regulation of metabolic process | 0.00155 |
|  | GOTERM_MF_5 | GO:0004896~cytokine receptor activity | 0.001596 |
|  | GOTERM_BP_4 | GO:0031323~regulation of cellular metabolic process | 0.001695 |
|  | GOTERM_MF_3 | GO:0019992~diacylglycerol binding | 0.001707 |
|  | GOTERM_BP_2 | GO:0042221~response to chemical stimulus | 0.001869 |
|  | GOTERM_CC_5 | GO:0005899~insulin receptor complex | 0.00187 |
|  | GOTERM_BP_3 | GO:0019222~regulation of metabolic process | 0.00199 |
|  | GOTERM_MF_4 | GO:0019978~interleukin-3 binding | 0.002038 |
|  | GOTERM_BP_2 | GO:0040008~regulation of growth | 0.002067 |
|  | GOTERM_MF_5 | GO:0004912~interleukin-3 receptor activity | 0.002208 |
|  | GOTERM_CC_4 | GO:0005899~insulin receptor complex | 0.002248 |
|  | GOTERM_BP_3 | GO:0040008~regulation of growth | 0.002285 |
|  | GOTERM_CC_3 | GO:0005886~plasma membrane | 0.00256 |
|  | GOTERM_BP_3 | GO:0009894~regulation of catabolic process | 0.002654 |
|  | GOTERM_BP_4 | GO:0009894~regulation of catabolic process | 0.002769 |
|  | GOTERM_MF_5 | GO:0005148~prolactin receptor binding | 0.003311 |
|  | GOTERM_MF_3 | GO:0004872~receptor activity | 0.003336 |
|  | GOTERM_CC_3 | GO:0043235~receptor complex | 0.003587 |
|  | GOTERM_BP_3 | GO:0010604~positive regulation of macromolecule metabolic process | 0.003749 |
|  | GOTERM_BP_5 | GO:0043627~response to estrogen stimulus | 0.00378 |
|  | GOTERM_BP_3 | GO:0008284~positive regulation of cell proliferation | 0.003962 |
|  | GOTERM_MF_5 | GO:0005088~Ras guanyl-nucleotide exchange factor activity | 0.004032 |
|  | GOTERM_BP_4 | GO:0010604~positive regulation of macromolecule metabolic process | 0.004056 |
|  | GOTERM_BP_3 | GO:0031325~positive regulation of cellular metabolic process | 0.004127 |
|  | GOTERM_BP_4 | GO:0008284~positive regulation of cell proliferation | 0.004212 |
|  | GOTERM_MF_3 | GO:0019955~cytokine binding | 0.004319 |
|  | GOTERM_BP_4 | GO:0031325~positive regulation of cellular metabolic process | 0.004464 |
|  | GOTERM_BP_5 | GO:0032000~positive regulation of fatty acid beta-oxidation | 0.004467 |
|  | GOTERM_MF_4 | GO:0043559~insulin binding | 0.005087 |
|  | GOTERM_BP_5 | GO:0008284~positive regulation of cell proliferation | 0.005115 |
|  | GOTERM_BP_5 | GO:0010604~positive regulation of macromolecule metabolic process | 0.005209 |
|  | GOTERM_BP_3 | GO:0030155~regulation of cell adhesion | 0.005324 |
|  | GOTERM_MF_5 | GO:0005131~growth hormone receptor binding | 0.005512 |
|  | GOTERM_BP_4 | GO:0030155~regulation of cell adhesion | 0.005553 |
|  | GOTERM_BP_5 | GO:0031325~positive regulation of cellular metabolic process | 0.005729 |
|  | GOTERM_BP_2 | GO:0048523~negative regulation of cellular process | 0.00591 |
|  | GOTERM_BP_2 | GO:0070271~protein complex biogenesis | 0.006268 |
|  | GOTERM_BP_1 | GO:0016043~cellular component organization | 0.006405 |
|  | GOTERM_MF_5 | GO:0031994~insulin-like growth factor I binding | 0.006611 |
|  | GOTERM_BP_4 | GO:0046321~positive regulation of fatty acid oxidation | 0.006662 |
|  | GOTERM_BP_4 | GO:0060396~growth hormone receptor signaling pathway | 0.006662 |
|  | GOTERM_MF_4 | GO:0004888~transmembrane receptor activity | 0.006737 |
|  | GOTERM_BP_3 | GO:0048523~negative regulation of cellular process | 0.006885 |
|  | GOTERM_BP_3 | GO:0006461~protein complex assembly | 0.006911 |
|  | GOTERM_BP_5 | GO:0046321~positive regulation of fatty acid oxidation | 0.007139 |
|  | GOTERM_BP_5 | GO:0060396~growth hormone receptor signaling pathway | 0.007139 |
|  | GOTERM_BP_5 | GO:0031998~regulation of fatty acid beta-oxidation | 0.007139 |
|  | GOTERM_BP_4 | GO:0006461~protein complex assembly | 0.007339 |
|  | GOTERM_BP_4 | GO:0048523~negative regulation of cellular process | 0.007562 |
|  | GOTERM_BP_5 | GO:0060416~response to growth hormone stimulus | 0.008028 |
|  | GOTERM_BP_4 | GO:0051259~protein oligomerization | 0.008821 |
|  | GOTERM_BP_3 | GO:0030238~male sex determination | 0.008954 |
|  | GOTERM_BP_4 | GO:0030238~male sex determination | 0.00915 |
|  | GOTERM_CC_5 | GO:0042598~vesicular fraction | 0.009483 |
|  | GOTERM_BP_5 | GO:0030238~male sex determination | 0.009804 |
|  | GOTERM_MF_4 | GO:0005085~guanyl-nucleotide exchange factor activity | 0.01004 |
|  | GOTERM_MF_3 | GO:0043560~insulin receptor substrate binding | 0.010072 |
|  | GOTERM_BP_5 | GO:0051259~protein oligomerization | 0.010076 |
|  | GOTERM_BP_2 | GO:0032879~regulation of localization | 0.010562 |
|  | GOTERM_BP_4 | GO:0048545~response to steroid hormone stimulus | 0.010659 |
|  | GOTERM_MF_3 | GO:0019899~enzyme binding | 0.010739 |
|  | GOTERM_BP_4 | GO:0050996~positive regulation of lipid catabolic process | 0.010805 |
|  | GOTERM_MF_5 | GO:0019901~protein kinase binding | 0.010909 |
|  | GOTERM_BP_5 | GO:0050996~positive regulation of lipid catabolic process | 0.011577 |
|  | GOTERM_BP_3 | GO:0032879~regulation of localization | 0.011626 |
|  | GOTERM_CC_2 | GO:0016020~membrane | 0.01251 |
|  | GOTERM_CC_3 | GO:0016020~membrane | 0.012587 |
|  | GOTERM_BP_2 | GO:0051050~positive regulation of transport | 0.012732 |
|  | GOTERM_MF_2 | GO:0042277~peptide binding | 0.013178 |
|  | GOTERM_BP_4 | GO:0030032~lamellipodium assembly | 0.013284 |
|  | GOTERM_BP_3 | GO:0051050~positive regulation of transport | 0.013614 |
|  | GOTERM_BP_5 | GO:0006916~anti-apoptosis | 0.013916 |
|  | GOTERM_BP_4 | GO:0009968~negative regulation of signal transduction | 0.013946 |
|  | GOTERM_BP_4 | GO:0002053~positive regulation of mesenchymal cell proliferation | 0.014109 |
|  | GOTERM_MF_4 | GO:0005154~epidermal growth factor receptor binding | 0.014184 |
|  | GOTERM_BP_4 | GO:0051050~positive regulation of transport | 0.014187 |
|  | GOTERM_BP_3 | GO:0065003~macromolecular complex assembly | 0.014698 |
|  | GOTERM_BP_4 | GO:0010557~positive regulation of macromolecule biosynthetic process | 0.014898 |
|  | GOTERM_BP_4 | GO:0010464~regulation of mesenchymal cell proliferation | 0.014933 |
|  | GOTERM_BP_5 | GO:0002053~positive regulation of mesenchymal cell proliferation | 0.015114 |
|  | GOTERM_BP_3 | GO:0007530~sex determination | 0.015421 |
|  | GOTERM_BP_4 | GO:0007530~sex determination | 0.015757 |
|  | GOTERM_BP_5 | GO:0009968~negative regulation of signal transduction | 0.015905 |
|  | GOTERM_BP_2 | GO:0043933~macromolecular complex subunit organization | 0.015952 |
|  | GOTERM_BP_5 | GO:0010464~regulation of mesenchymal cell proliferation | 0.015997 |
|  | GOTERM_BP_5 | GO:0051050~positive regulation of transport | 0.016179 |
|  | GOTERM_BP_3 | GO:0009891~positive regulation of biosynthetic process | 0.016554 |
|  | GOTERM_BP_3 | GO:0010648~negative regulation of cell communication | 0.016658 |
|  | GOTERM_BP_4 | GO:0031328~positive regulation of cellular biosynthetic process | 0.016877 |
|  | GOTERM_BP_4 | GO:0010648~negative regulation of cell communication | 0.017356 |
|  | GOTERM_BP_4 | GO:0045923~positive regulation of fatty acid metabolic process | 0.017402 |
|  | GOTERM_BP_4 | GO:0009891~positive regulation of biosynthetic process | 0.017546 |
|  | GOTERM_BP_5 | GO:0010557~positive regulation of macromolecule biosynthetic process | 0.017947 |
|  | GOTERM_BP_5 | GO:0045923~positive regulation of fatty acid metabolic process | 0.018641 |
|  | GOTERM_BP_5 | GO:0010648~negative regulation of cell communication | 0.019776 |
|  | GOTERM_BP_5 | GO:0031328~positive regulation of cellular biosynthetic process | 0.02031 |
|  | GOTERM_BP_5 | GO:0051056~regulation of small GTPase mediated signal transduction | 0.020381 |
|  | GOTERM_BP_5 | GO:0009891~positive regulation of biosynthetic process | 0.021108 |
|  | GOTERM_BP_4 | GO:0050994~regulation of lipid catabolic process | 0.021505 |
|  | GOTERM_BP_5 | GO:0050994~regulation of lipid catabolic process | 0.023032 |
|  | GOTERM_BP_3 | GO:0042127~regulation of cell proliferation | 0.023063 |
|  | GOTERM_BP_4 | GO:0046320~regulation of fatty acid oxidation | 0.023141 |
|  | GOTERM_BP_4 | GO:0009967~positive regulation of signal transduction | 0.024053 |
|  | GOTERM_MF_4 | GO:0042169~SH2 domain binding | 0.024202 |
|  | GOTERM_BP_4 | GO:0042127~regulation of cell proliferation | 0.024423 |
|  | GOTERM_BP_5 | GO:0046320~regulation of fatty acid oxidation | 0.024783 |
|  | GOTERM_BP_5 | GO:0045740~positive regulation of DNA replication | 0.024783 |
|  | GOTERM_MF_4 | GO:0005520~insulin-like growth factor binding | 0.025198 |
|  | GOTERM_BP_5 | GO:0009967~positive regulation of signal transduction | 0.027365 |
|  | GOTERM_BP_3 | GO:0010647~positive regulation of cell communication | 0.028306 |
|  | GOTERM_BP_4 | GO:0010647~positive regulation of cell communication | 0.029467 |
|  | GOTERM_MF_4 | GO:0005083~small GTPase regulator activity | 0.030541 |
|  | GOTERM_BP_3 | GO:0080090~regulation of primary metabolic process | 0.030835 |
|  | GOTERM_MF_1 | GO:0030234~enzyme regulator activity | 0.031976 |
|  | GOTERM_CC_5 | GO:0005901~caveola | 0.031984 |
|  | GOTERM_BP_5 | GO:0012502~induction of programmed cell death | 0.032002 |
|  | GOTERM_CC_5 | GO:0005887~integral to plasma membrane | 0.032089 |
|  | GOTERM_BP_2 | GO:0030030~cell projection organization | 0.032634 |
|  | GOTERM_BP_3 | GO:0043069~negative regulation of programmed cell death | 0.033265 |
|  | GOTERM_BP_3 | GO:0060548~negative regulation of cell death | 0.033436 |
|  | GOTERM_BP_5 | GO:0010647~positive regulation of cell communication | 0.033488 |
|  | GOTERM_BP_4 | GO:0043066~negative regulation of apoptosis | 0.033737 |
|  | GOTERM_BP_1 | GO:0050896~response to stimulus | 0.033782 |
|  | GOTERM_CC_5 | GO:0031226~intrinsic to plasma membrane | 0.03403 |
|  | GOTERM_BP_4 | GO:0080090~regulation of primary metabolic process | 0.034145 |
|  | GOTERM_BP_5 | GO:0051262~protein tetramerization | 0.034366 |
|  | GOTERM_BP_4 | GO:0043069~negative regulation of programmed cell death | 0.034619 |
|  | GOTERM_BP_4 | GO:0060548~negative regulation of cell death | 0.034797 |
|  | GOTERM_MF_3 | GO:0019904~protein domain specific binding | 0.035786 |
|  | GOTERM_BP_5 | GO:0043066~negative regulation of apoptosis | 0.038309 |
|  | GOTERM_CC_4 | GO:0005901~caveola | 0.038325 |
|  | GOTERM_BP_5 | GO:0043069~negative regulation of programmed cell death | 0.039305 |
|  | GOTERM_BP_5 | GO:0060548~negative regulation of cell death | 0.039505 |
|  | GOTERM_MF_5 | GO:0019903~protein phosphatase binding | 0.042268 |
|  | GOTERM_BP_5 | GO:0033500~carbohydrate homeostasis | 0.043003 |
|  | GOTERM_BP_5 | GO:0019217~regulation of fatty acid metabolic process | 0.043003 |
|  | GOTERM_CC_5 | GO:0005829~cytosol | 0.043006 |
|  | GOTERM_BP_3 | GO:0048513~organ development | 0.043182 |
|  | GOTERM_MF_4 | GO:0019902~phosphatase binding | 0.043958 |
|  | GOTERM_MF_3 | GO:0005070~SH3/SH2 adaptor activity | 0.044153 |
|  | GOTERM_BP_4 | GO:0051054~positive regulation of DNA metabolic process | 0.045795 |
|  | GOTERM_BP_4 | GO:0048513~organ development | 0.046301 |
|  | GOTERM_BP_4 | GO:0010565~regulation of cellular ketone metabolic process | 0.046595 |
|  | GOTERM_BP_3 | GO:0010942~positive regulation of cell death | 0.047238 |
|  | GOTERM_BP_3 | GO:0051049~regulation of transport | 0.047238 |
|  | GOTERM_BP_3 | GO:0045785~positive regulation of cell adhesion | 0.047963 |
|  | GOTERM_BP_4 | GO:0043068~positive regulation of programmed cell death | 0.048718 |
|  | GOTERM_MF_4 | GO:0005070~SH3/SH2 adaptor activity | 0.048839 |
|  | GOTERM_BP_4 | GO:0045785~positive regulation of cell adhesion | 0.048991 |
|  | GOTERM_BP_5 | GO:0051054~positive regulation of DNA metabolic process | 0.049007 |
|  | GOTERM_BP_4 | GO:0010942~positive regulation of cell death | 0.049125 |
|  | GOTERM_BP_4 | GO:0051049~regulation of transport | 0.049125 |
|  | GOTERM_MF_2 | GO:0060589~nucleoside-triphosphatase regulator activity | 0.049306 |
|  | GOTERM_BP_5 | GO:0010565~regulation of cellular ketone metabolic process | 0.049862 |
| 4078 | GOTERM_BP_5 | GO:0006164~purine nucleotide biosynthetic process | 3.33E-05 |
|  | GOTERM_BP_5 | GO:0034654~nucleobase, nucleoside, nucleotide and nucleic acid biosynthetic process | 7.36E-05 |
|  | GOTERM_BP_5 | GO:0034404~nucleobase, nucleoside and nucleotide biosynthetic process | 7.36E-05 |
|  | GOTERM_BP_4 | GO:0009165~nucleotide biosynthetic process | 9.30E-05 |
|  | GOTERM_BP_4 | GO:0006163~purine nucleotide metabolic process | 9.30E-05 |
|  | GOTERM_BP_4 | GO:0034654~nucleobase, nucleoside, nucleotide and nucleic acid biosynthetic process | 1.04E-04 |
|  | GOTERM_BP_5 | GO:0006753~nucleoside phosphate metabolic process | 2.54E-04 |
|  | GOTERM_BP_4 | GO:0055086~nucleobase, nucleoside and nucleotide metabolic process | 4.45E-04 |
|  | GOTERM_BP_4 | GO:0044271~nitrogen compound biosynthetic process | 4.84E-04 |
|  | GOTERM_BP_3 | GO:0046483~heterocycle metabolic process | 4.92E-04 |
|  | GOTERM_CC_4 | GO:0005829~cytosol | 5.61E-04 |
|  | GOTERM_CC_5 | GO:0005829~cytosol | 6.07E-04 |
|  | GOTERM_CC_3 | GO:0044444~cytoplasmic part | 7.11E-04 |
|  | GOTERM_CC_4 | GO:0044444~cytoplasmic part | 9.71E-04 |
|  | GOTERM_BP_5 | GO:0015677~copper ion import | 9.76E-04 |
|  | GOTERM_BP_4 | GO:0015677~copper ion import | 0.001062 |
|  | GOTERM_CC_5 | GO:0044444~cytoplasmic part | 0.0011 |
|  | GOTERM_CC_3 | GO:0008074~guanylate cyclase complex, soluble | 0.001177 |
|  | GOTERM_CC_5 | GO:0008074~guanylate cyclase complex, soluble | 0.001247 |
|  | GOTERM_CC_3 | GO:0005737~cytoplasm | 0.001322 |
|  | GOTERM_MF_3 | GO:0032767~copper-dependent protein binding | 0.001379 |
|  | GOTERM_MF_4 | GO:0032555~purine ribonucleotide binding | 0.001461 |
|  | GOTERM_MF_2 | GO:0016531~copper chaperone activity | 0.00182 |
|  | GOTERM_CC_4 | GO:0005737~cytoplasm | 0.001913 |
|  | GOTERM_CC_5 | GO:0005737~cytoplasm | 0.00222 |
|  | GOTERM_MF_1 | GO:0016530~metallochaperone activity | 0.002377 |
|  | GOTERM_BP_3 | GO:0006979~response to oxidative stress | 0.002975 |
|  | GOTERM_MF_3 | GO:0032553~ribonucleotide binding | 0.004214 |
|  | GOTERM_BP_3 | GO:0010035~response to inorganic substance | 0.004607 |
|  | GOTERM_MF_4 | GO:0004383~guanylate cyclase activity | 0.004748 |
|  | GOTERM_MF_3 | GO:0017076~purine nucleotide binding | 0.005123 |
|  | GOTERM_MF_3 | GO:0004383~guanylate cyclase activity | 0.005505 |
|  | GOTERM_BP_5 | GO:0046688~response to copper ion | 0.005844 |
|  | GOTERM_MF_2 | GO:0000166~nucleotide binding | 0.005855 |
|  | GOTERM_BP_5 | GO:0030003~cellular cation homeostasis | 0.006039 |
|  | GOTERM_CC_4 | GO:0016323~basolateral plasma membrane | 0.006436 |
|  | GOTERM_CC_5 | GO:0016323~basolateral plasma membrane | 0.006646 |
|  | GOTERM_BP_5 | GO:0046068~cGMP metabolic process | 0.006816 |
|  | GOTERM_BP_5 | GO:0007263~nitric oxide mediated signal transduction | 0.006816 |
|  | GOTERM_BP_5 | GO:0055082~cellular chemical homeostasis | 0.013166 |
|  | GOTERM_MF_2 | GO:0009975~cyclase activity | 0.01388 |
|  | GOTERM_BP_5 | GO:0034614~cellular response to reactive oxygen species | 0.014558 |
|  | GOTERM_MF_3 | GO:0016849~phosphorus-oxygen lyase activity | 0.015074 |
|  | GOTERM_BP_5 | GO:0050801~ion homeostasis | 0.015158 |
|  | GOTERM_BP_3 | GO:0055082~cellular chemical homeostasis | 0.01519 |
|  | GOTERM_BP_4 | GO:0006873~cellular ion homeostasis | 0.015357 |
|  | GOTERM_BP_5 | GO:0030001~metal ion transport | 0.019359 |
|  | GOTERM_MF_4 | GO:0019001~guanyl nucleotide binding | 0.019792 |
|  | GOTERM_MF_2 | GO:0043167~ion binding | 0.019899 |
|  | GOTERM_BP_2 | GO:0042221~response to chemical stimulus | 0.020406 |
|  | GOTERM_BP_2 | GO:0019725~cellular homeostasis | 0.020932 |
|  | GOTERM_MF_5 | GO:0046914~transition metal ion binding | 0.021152 |
|  | GOTERM_BP_4 | GO:0034599~cellular response to oxidative stress | 0.022631 |
|  | GOTERM_BP_4 | GO:0019725~cellular homeostasis | 0.0233 |
|  | GOTERM_BP_5 | GO:0034220~ion transmembrane transport | 0.023686 |
|  | GOTERM_BP_5 | GO:0009124~nucleoside monophosphate biosynthetic process | 0.024164 |
|  | GOTERM_BP_3 | GO:0034220~ion transmembrane transport | 0.025206 |
|  | GOTERM_BP_4 | GO:0034220~ion transmembrane transport | 0.025753 |
|  | GOTERM_BP_5 | GO:0006812~cation transport | 0.026862 |
|  | GOTERM_MF_5 | GO:0032561~guanyl ribonucleotide binding | 0.027121 |
|  | GOTERM_BP_2 | GO:0006810~transport | 0.027149 |
|  | GOTERM_BP_1 | GO:0051234~establishment of localization | 0.0274 |
|  | GOTERM_BP_4 | GO:0048878~chemical homeostasis | 0.027803 |
|  | GOTERM_CC_4 | GO:0005802~trans-Golgi network | 0.027881 |
|  | GOTERM_BP_2 | GO:0051234~establishment of localization | 0.02813 |
|  | GOTERM_CC_5 | GO:0005802~trans-Golgi network | 0.028341 |
|  | GOTERM_BP_2 | GO:0065008~regulation of biological quality | 0.029489 |
|  | GOTERM_BP_3 | GO:0006810~transport | 0.03067 |
|  | GOTERM_BP_4 | GO:0006812~cation transport | 0.0321 |
|  | GOTERM_BP_3 | GO:0006800~oxygen and reactive oxygen species metabolic process | 0.034328 |
|  | GOTERM_CC_5 | GO:0005770~late endosome | 0.034406 |
|  | GOTERM_CC_2 | GO:0044424~intracellular part | 0.037623 |
|  | GOTERM_CC_3 | GO:0044424~intracellular part | 0.037867 |
|  | GOTERM_BP_4 | GO:0000302~response to reactive oxygen species | 0.039186 |
|  | GOTERM_BP_1 | GO:0051179~localization | 0.041319 |
|  | GOTERM_BP_5 | GO:0009142~nucleoside triphosphate biosynthetic process | 0.048777 |
| 4224 | GOTERM_BP_4 | GO:0003018~vascular process in circulatory system | 1.91E-05 |
|  | GOTERM_BP_3 | GO:0008217~regulation of blood pressure | 5.46E-05 |
|  | GOTERM_BP_5 | GO:0008217~regulation of blood pressure | 6.55E-05 |
|  | GOTERM_BP_3 | GO:0003013~circulatory system process | 1.90E-04 |
|  | GOTERM_BP_4 | GO:0008015~blood circulation | 1.98E-04 |
|  | GOTERM_BP_3 | GO:0044057~regulation of system process | 5.25E-04 |
|  | GOTERM_BP_4 | GO:0044057~regulation of system process | 5.48E-04 |
|  | GOTERM_MF_5 | GO:0004947~bradykinin receptor activity | 6.02E-04 |
|  | GOTERM_MF_3 | GO:0046983~protein dimerization activity | 0.001722 |
|  | GOTERM_BP_4 | GO:0042312~regulation of vasodilation | 0.003034 |
|  | GOTERM_BP_3 | GO:0042592~homeostatic process | 0.003108 |
|  | GOTERM_BP_5 | GO:0042312~regulation of vasodilation | 0.003252 |
|  | GOTERM_BP_3 | GO:0042127~regulation of cell proliferation | 0.003413 |
|  | GOTERM_BP_3 | GO:0045776~negative regulation of blood pressure | 0.003414 |
|  | GOTERM_BP_4 | GO:0045776~negative regulation of blood pressure | 0.003489 |
|  | GOTERM_BP_4 | GO:0042127~regulation of cell proliferation | 0.003564 |
|  | GOTERM_BP_5 | GO:0003044~regulation of systemic arterial blood pressure mediated by a chemical signal | 0.004388 |
|  | GOTERM_BP_2 | GO:0051239~regulation of multicellular organismal process | 0.00451 |
|  | GOTERM_BP_3 | GO:0051239~regulation of multicellular organismal process | 0.004839 |
|  | GOTERM_BP_4 | GO:0019229~regulation of vasoconstriction | 0.005155 |
|  | GOTERM_BP_4 | GO:0003073~regulation of systemic arterial blood pressure | 0.005307 |
|  | GOTERM_BP_5 | GO:0019229~regulation of vasoconstriction | 0.005525 |
|  | GOTERM_CC_5 | GO:0005887~integral to plasma membrane | 0.006776 |
|  | GOTERM_CC_4 | GO:0031226~intrinsic to plasma membrane | 0.006856 |
|  | GOTERM_CC_5 | GO:0031226~intrinsic to plasma membrane | 0.007087 |
|  | GOTERM_MF_5 | GO:0004930~G-protein coupled receptor activity | 0.007678 |
|  | GOTERM_BP_3 | GO:0035150~regulation of tube size | 0.007858 |
|  | GOTERM_BP_4 | GO:0050880~regulation of blood vessel size | 0.00803 |
|  | GOTERM_BP_5 | GO:0007186~G-protein coupled receptor protein signaling pathway | 0.00834 |
|  | GOTERM_BP_5 | GO:0050880~regulation of blood vessel size | 0.008605 |
|  | GOTERM_BP_2 | GO:0065008~regulation of biological quality | 0.01109 |
|  | GOTERM_BP_4 | GO:0050727~regulation of inflammatory response | 0.011505 |
|  | GOTERM_BP_2 | GO:0003008~system process | 0.01161 |
|  | GOTERM_MF_4 | GO:0004888~transmembrane receptor activity | 0.012279 |
|  | GOTERM_BP_5 | GO:0050727~regulation of inflammatory response | 0.012328 |
|  | GOTERM_MF_3 | GO:0001653~peptide receptor activity | 0.017394 |
|  | GOTERM_MF_4 | GO:0001664~G-protein-coupled receptor binding | 0.018769 |
|  | GOTERM_MF_4 | GO:0001653~peptide receptor activity | 0.019273 |
|  | GOTERM_MF_4 | GO:0008528~peptide receptor activity, G-protein coupled | 0.019273 |
|  | GOTERM_MF_3 | GO:0004872~receptor activity | 0.019824 |
|  | GOTERM_BP_4 | GO:0007166~cell surface receptor linked signal transduction | 0.019839 |
|  | GOTERM_CC_3 | GO:0044459~plasma membrane part | 0.02077 |
|  | GOTERM_BP_4 | GO:0031347~regulation of defense response | 0.021592 |
|  | GOTERM_MF_1 | GO:0060089~molecular transducer activity | 0.022484 |
|  | GOTERM_CC_4 | GO:0044459~plasma membrane part | 0.022549 |
|  | GOTERM_MF_5 | GO:0008528~peptide receptor activity, G-protein coupled | 0.022766 |
|  | GOTERM_BP_5 | GO:0007166~cell surface receptor linked signal transduction | 0.022788 |
|  | GOTERM_BP_5 | GO:0031347~regulation of defense response | 0.023133 |
|  | GOTERM_CC_5 | GO:0044459~plasma membrane part | 0.023308 |
|  | GOTERM_MF_2 | GO:0004871~signal transducer activity | 0.023437 |
|  | GOTERM_BP_3 | GO:0032101~regulation of response to external stimulus | 0.023482 |
|  | GOTERM_BP_4 | GO:0032101~regulation of response to external stimulus | 0.023994 |
|  | GOTERM_MF_2 | GO:0042277~peptide binding | 0.0272 |
|  | GOTERM_MF_4 | GO:0046982~protein heterodimerization activity | 0.035024 |
|  | GOTERM_BP_5 | GO:0019932~second-messenger-mediated signaling | 0.037872 |
|  | GOTERM_BP_2 | GO:0051094~positive regulation of developmental process | 0.039475 |
|  | GOTERM_BP_3 | GO:0080134~regulation of response to stress | 0.040291 |
|  | GOTERM_BP_3 | GO:0051094~positive regulation of developmental process | 0.040873 |
|  | GOTERM_BP_5 | GO:0030003~cellular cation homeostasis | 0.040902 |
|  | GOTERM_BP_4 | GO:0080134~regulation of response to stress | 0.041166 |
|  | GOTERM_BP_4 | GO:0051094~positive regulation of developmental process | 0.041761 |
|  | GOTERM_CC_4 | GO:0005768~endosome | 0.042491 |
|  | GOTERM_CC_5 | GO:0005768~endosome | 0.043193 |
|  | GOTERM_BP_3 | GO:0007165~signal transduction | 0.044487 |
|  | GOTERM_BP_4 | GO:0007165~signal transduction | 0.04646 |
|  | GOTERM_BP_2 | GO:0040008~regulation of growth | 0.04831 |
| 4305 | GOTERM_BP_1 | GO:0022610~biological adhesion | 1.18E-13 |
|  | GOTERM_BP_2 | GO:0007155~cell adhesion | 1.29E-13 |
|  | GOTERM_CC_5 | GO:0005887~integral to plasma membrane | 1.85E-09 |
|  | GOTERM_CC_4 | GO:0031226~intrinsic to plasma membrane | 1.99E-09 |
|  | GOTERM_CC_5 | GO:0031226~intrinsic to plasma membrane | 2.44E-09 |
|  | GOTERM_CC_4 | GO:0044459~plasma membrane part | 2.55E-08 |
|  | GOTERM_CC_5 | GO:0044459~plasma membrane part | 3.21E-08 |
|  | GOTERM_CC_3 | GO:0044459~plasma membrane part | 8.28E-08 |
|  | GOTERM_CC_4 | GO:0008305~integrin complex | 8.79E-08 |
|  | GOTERM_CC_5 | GO:0008305~integrin complex | 9.39E-08 |
|  | GOTERM_BP_5 | GO:0007229~integrin-mediated signaling pathway | 2.52E-07 |
|  | GOTERM_CC_4 | GO:0005886~plasma membrane | 5.21E-06 |
|  | GOTERM_MF_4 | GO:0005178~integrin binding | 5.61E-06 |
|  | GOTERM_BP_4 | GO:0001944~vasculature development | 7.53E-06 |
|  | GOTERM_BP_5 | GO:0001568~blood vessel development | 7.60E-06 |
|  | GOTERM_BP_5 | GO:0001944~vasculature development | 8.73E-06 |
|  | GOTERM_CC_3 | GO:0005886~plasma membrane | 1.70E-05 |
|  | GOTERM_BP_4 | GO:0007160~cell-matrix adhesion | 2.60E-05 |
|  | GOTERM_BP_3 | GO:0031589~cell-substrate adhesion | 2.97E-05 |
|  | GOTERM_CC_3 | GO:0043235~receptor complex | 2.98E-05 |
|  | GOTERM_MF_2 | GO:0005515~protein binding | 4.21E-05 |
|  | GOTERM_BP_4 | GO:0048514~blood vessel morphogenesis | 5.03E-05 |
|  | GOTERM_BP_5 | GO:0048514~blood vessel morphogenesis | 5.71E-05 |
|  | GOTERM_BP_3 | GO:0032879~regulation of localization | 8.91E-05 |
|  | GOTERM_BP_2 | GO:0032879~regulation of localization | 9.41E-05 |
|  | GOTERM_BP_3 | GO:0048518~positive regulation of biological process | 1.03E-04 |
|  | GOTERM_BP_2 | GO:0048518~positive regulation of biological process | 1.17E-04 |
|  | GOTERM_BP_3 | GO:0001525~angiogenesis | 1.48E-04 |
|  | GOTERM_BP_3 | GO:0045785~positive regulation of cell adhesion | 1.81E-04 |
|  | GOTERM_BP_4 | GO:0001525~angiogenesis | 1.89E-04 |
|  | GOTERM_BP_5 | GO:0001525~angiogenesis | 2.10E-04 |
|  | GOTERM_BP_4 | GO:0045785~positive regulation of cell adhesion | 2.17E-04 |
|  | GOTERM_BP_3 | GO:0048522~positive regulation of cellular process | 2.20E-04 |
|  | GOTERM_MF_3 | GO:0005102~receptor binding | 2.27E-04 |
|  | GOTERM_BP_5 | GO:0045785~positive regulation of cell adhesion | 2.36E-04 |
|  | GOTERM_BP_2 | GO:0048522~positive regulation of cellular process | 2.44E-04 |
|  | GOTERM_BP_3 | GO:0030334~regulation of cell migration | 2.47E-04 |
|  | GOTERM_BP_4 | GO:0030334~regulation of cell migration | 3.13E-04 |
|  | GOTERM_BP_5 | GO:0030334~regulation of cell migration | 3.48E-04 |
|  | GOTERM_BP_3 | GO:0040012~regulation of locomotion | 4.01E-04 |
|  | GOTERM_BP_4 | GO:0048522~positive regulation of cellular process | 4.04E-04 |
|  | GOTERM_BP_3 | GO:0051270~regulation of cell motion | 4.09E-04 |
|  | GOTERM_BP_2 | GO:0040012~regulation of locomotion | 4.11E-04 |
|  | GOTERM_MF_3 | GO:0004872~receptor activity | 4.21E-04 |
|  | GOTERM_BP_4 | GO:0007166~cell surface receptor linked signal transduction | 4.22E-04 |
|  | GOTERM_BP_5 | GO:0007166~cell surface receptor linked signal transduction | 5.16E-04 |
|  | GOTERM_BP_4 | GO:0051270~regulation of cell motion | 5.19E-04 |
|  | GOTERM_MF_3 | GO:0032403~protein complex binding | 5.69E-04 |
|  | GOTERM_BP_4 | GO:0030335~positive regulation of cell migration | 6.93E-04 |
|  | GOTERM_MF_1 | GO:0060089~molecular transducer activity | 7.32E-04 |
|  | GOTERM_BP_5 | GO:0030335~positive regulation of cell migration | 7.52E-04 |
|  | GOTERM_BP_3 | GO:0051272~positive regulation of cell motion | 7.66E-04 |
|  | GOTERM_BP_3 | GO:0040017~positive regulation of locomotion | 7.66E-04 |
|  | GOTERM_BP_2 | GO:0040017~positive regulation of locomotion | 7.78E-04 |
|  | GOTERM_BP_3 | GO:0008284~positive regulation of cell proliferation | 8.57E-04 |
|  | GOTERM_MF_2 | GO:0004871~signal transducer activity | 8.76E-04 |
|  | GOTERM_BP_4 | GO:0051272~positive regulation of cell motion | 9.17E-04 |
|  | GOTERM_BP_4 | GO:0040017~positive regulation of locomotion | 9.17E-04 |
|  | GOTERM_BP_4 | GO:0007044~cell-substrate junction assembly | 9.24E-04 |
|  | GOTERM_BP_5 | GO:0051272~positive regulation of cell motion | 9.95E-04 |
|  | GOTERM_BP_4 | GO:0008284~positive regulation of cell proliferation | 0.00114 |
|  | GOTERM_BP_5 | GO:0008284~positive regulation of cell proliferation | 0.001287 |
|  | GOTERM_BP_4 | GO:0010811~positive regulation of cell-substrate adhesion | 0.001372 |
|  | GOTERM_BP_5 | GO:0010811~positive regulation of cell-substrate adhesion | 0.001453 |
|  | GOTERM_CC_2 | GO:0016020~membrane | 0.001543 |
|  | GOTERM_CC_3 | GO:0016020~membrane | 0.00156 |
|  | GOTERM_CC_2 | GO:0009986~cell surface | 0.001953 |
|  | GOTERM_CC_3 | GO:0009986~cell surface | 0.001958 |
|  | GOTERM_CC_4 | GO:0009897~external side of plasma membrane | 0.002003 |
|  | GOTERM_BP_3 | GO:0030155~regulation of cell adhesion | 0.002012 |
|  | GOTERM_CC_4 | GO:0044425~membrane part | 0.002034 |
|  | GOTERM_CC_5 | GO:0009897~external side of plasma membrane | 0.002099 |
|  | GOTERM_CC_4 | GO:0016021~integral to membrane | 0.002225 |
|  | GOTERM_CC_3 | GO:0009897~external side of plasma membrane | 0.002305 |
|  | GOTERM_BP_4 | GO:0030155~regulation of cell adhesion | 0.002401 |
|  | GOTERM_BP_3 | GO:0034329~cell junction assembly | 0.002597 |
|  | GOTERM_BP_3 | GO:0042127~regulation of cell proliferation | 0.002658 |
|  | GOTERM_CC_5 | GO:0016021~integral to membrane | 0.00269 |
|  | GOTERM_MF_5 | GO:0005509~calcium ion binding | 0.002753 |
|  | GOTERM_CC_4 | GO:0016323~basolateral plasma membrane | 0.003313 |
|  | GOTERM_CC_4 | GO:0031224~intrinsic to membrane | 0.003313 |
|  | GOTERM_CC_5 | GO:0016323~basolateral plasma membrane | 0.00347 |
|  | GOTERM_BP_4 | GO:0042127~regulation of cell proliferation | 0.003662 |
|  | GOTERM_BP_4 | GO:0010810~regulation of cell-substrate adhesion | 0.003675 |
|  | GOTERM_BP_3 | GO:0048646~anatomical structure formation involved in morphogenesis | 0.003762 |
|  | GOTERM_BP_2 | GO:0048646~anatomical structure formation involved in morphogenesis | 0.003844 |
|  | GOTERM_BP_5 | GO:0010810~regulation of cell-substrate adhesion | 0.003891 |
|  | GOTERM_CC_5 | GO:0031224~intrinsic to membrane | 0.003994 |
|  | GOTERM_MF_5 | GO:0043185~vascular endothelial growth factor receptor 3 binding | 0.004414 |
|  | GOTERM_BP_4 | GO:0048646~anatomical structure formation involved in morphogenesis | 0.004704 |
|  | GOTERM_BP_2 | GO:0034330~cell junction organization | 0.005009 |
|  | GOTERM_CC_2 | GO:0044425~membrane part | 0.005464 |
|  | GOTERM_CC_3 | GO:0044425~membrane part | 0.005513 |
|  | GOTERM_CC_3 | GO:0031224~intrinsic to membrane | 0.00713 |
|  | GOTERM_BP_3 | GO:0050793~regulation of developmental process | 0.007141 |
|  | GOTERM_MF_1 | GO:0005488~binding | 0.007335 |
|  | GOTERM_BP_2 | GO:0050793~regulation of developmental process | 0.007336 |
|  | GOTERM_MF_4 | GO:0005172~vascular endothelial growth factor receptor binding | 0.00813 |
|  | GOTERM_BP_3 | GO:0048513~organ development | 0.010772 |
|  | GOTERM_BP_1 | GO:0065007~biological regulation | 0.01152 |
|  | GOTERM_BP_3 | GO:0045595~regulation of cell differentiation | 0.012111 |
|  | GOTERM_BP_5 | GO:0043277~apoptotic cell clearance | 0.012144 |
|  | GOTERM_BP_3 | GO:0033627~cell adhesion mediated by integrin | 0.012929 |
|  | GOTERM_BP_4 | GO:0045595~regulation of cell differentiation | 0.014959 |
|  | GOTERM_BP_4 | GO:0048513~organ development | 0.015671 |
|  | GOTERM_BP_2 | GO:0050789~regulation of biological process | 0.018356 |
|  | GOTERM_MF_3 | GO:0019838~growth factor binding | 0.018376 |
|  | GOTERM_BP_3 | GO:0048731~system development | 0.019759 |
|  | GOTERM_BP_3 | GO:0009653~anatomical structure morphogenesis | 0.019813 |
|  | GOTERM_BP_2 | GO:0009653~anatomical structure morphogenesis | 0.020414 |
|  | GOTERM_BP_3 | GO:0050794~regulation of cellular process | 0.025114 |
|  | GOTERM_BP_1 | GO:0009987~cellular process | 0.025866 |
|  | GOTERM_BP_2 | GO:0007275~multicellular organismal development | 0.027563 |
|  | GOTERM_BP_2 | GO:0050794~regulation of cellular process | 0.02929 |
|  | GOTERM_BP_5 | GO:0046847~filopodium assembly | 0.030094 |
|  | GOTERM_CC_5 | GO:0030141~secretory granule | 0.030363 |
|  | GOTERM_BP_4 | GO:0030035~microspike assembly | 0.031132 |
|  | GOTERM_BP_5 | GO:0007167~enzyme linked receptor protein signaling pathway | 0.031219 |
|  | GOTERM_CC_4 | GO:0044433~cytoplasmic vesicle part | 0.031595 |
|  | GOTERM_CC_5 | GO:0044433~cytoplasmic vesicle part | 0.032569 |
|  | GOTERM_BP_2 | GO:0048856~anatomical structure development | 0.033759 |
|  | GOTERM_BP_3 | GO:0010876~lipid localization | 0.034018 |
|  | GOTERM_CC_3 | GO:0044433~cytoplasmic vesicle part | 0.034455 |
|  | GOTERM_BP_4 | GO:0019915~lipid storage | 0.034957 |
|  | GOTERM_BP_2 | GO:0043062~extracellular structure organization | 0.036736 |
|  | GOTERM_CC_1 | GO:0005576~extracellular region | 0.038146 |
|  | GOTERM_BP_3 | GO:0010883~regulation of lipid storage | 0.038308 |
|  | GOTERM_BP_4 | GO:0010883~regulation of lipid storage | 0.040668 |
|  | GOTERM_MF_4 | GO:0008083~growth factor activity | 0.042139 |
|  | GOTERM_BP_1 | GO:0040011~locomotion | 0.044204 |
|  | GOTERM_BP_3 | GO:0051049~regulation of transport | 0.045513 |
|  | GOTERM_MF_2 | GO:0050840~extracellular matrix binding | 0.046328 |
|  | GOTERM_BP_1 | GO:0032502~developmental process | 0.047642 |
|  | GOTERM_BP_5 | GO:0015674~di-, tri-valent inorganic cation transport | 0.04931 |
| 4393 | GOTERM_CC_5 | GO:0005887~integral to plasma membrane | 1.28E-10 |
|  | GOTERM_CC_5 | GO:0031226~intrinsic to plasma membrane | 1.69E-10 |
|  | GOTERM_CC_4 | GO:0031226~intrinsic to plasma membrane | 3.63E-10 |
|  | GOTERM_CC_5 | GO:0008305~integrin complex | 5.00E-08 |
|  | GOTERM_CC_4 | GO:0008305~integrin complex | 5.84E-08 |
|  | GOTERM_CC_2 | GO:0009986~cell surface | 1.79E-07 |
|  | GOTERM_CC_3 | GO:0009986~cell surface | 1.79E-07 |
|  | GOTERM_BP_1 | GO:0022610~biological adhesion | 1.86E-07 |
|  | GOTERM_BP_2 | GO:0007155~cell adhesion | 1.96E-07 |
|  | GOTERM_CC_5 | GO:0044459~plasma membrane part | 2.60E-07 |
|  | GOTERM_CC_3 | GO:0044459~plasma membrane part | 3.22E-07 |
|  | GOTERM_CC_4 | GO:0044459~plasma membrane part | 5.24E-07 |
|  | GOTERM_BP_3 | GO:0016337~cell-cell adhesion | 2.14E-06 |
|  | GOTERM_BP_5 | GO:0007229~integrin-mediated signaling pathway | 2.78E-06 |
|  | GOTERM_CC_3 | GO:0005886~plasma membrane | 3.21E-06 |
|  | GOTERM_CC_4 | GO:0005886~plasma membrane | 5.58E-06 |
|  | GOTERM_BP_4 | GO:0007159~leukocyte adhesion | 9.57E-06 |
|  | GOTERM_CC_3 | GO:0043235~receptor complex | 1.39E-05 |
|  | GOTERM_BP_5 | GO:0016485~protein processing | 1.81E-05 |
|  | GOTERM_BP_5 | GO:0051604~protein maturation | 2.54E-05 |
|  | GOTERM_BP_4 | GO:0051604~protein maturation | 3.02E-05 |
|  | GOTERM_CC_5 | GO:0031224~intrinsic to membrane | 4.85E-05 |
|  | GOTERM_BP_5 | GO:0007219~Notch signaling pathway | 5.57E-05 |
|  | GOTERM_CC_3 | GO:0009897~external side of plasma membrane | 6.23E-05 |
|  | GOTERM_CC_5 | GO:0009897~external side of plasma membrane | 6.28E-05 |
|  | GOTERM_CC_3 | GO:0031224~intrinsic to membrane | 7.03E-05 |
|  | GOTERM_CC_4 | GO:0009897~external side of plasma membrane | 7.30E-05 |
|  | GOTERM_BP_5 | GO:0045123~cellular extravasation | 9.05E-05 |
|  | GOTERM_BP_3 | GO:0045123~cellular extravasation | 9.37E-05 |
|  | GOTERM_BP_5 | GO:0042982~amyloid precursor protein metabolic process | 1.11E-04 |
|  | GOTERM_CC_4 | GO:0031224~intrinsic to membrane | 1.24E-04 |
|  | GOTERM_CC_5 | GO:0031410~cytoplasmic vesicle | 1.25E-04 |
|  | GOTERM_CC_3 | GO:0031410~cytoplasmic vesicle | 1.26E-04 |
|  | GOTERM_CC_4 | GO:0031410~cytoplasmic vesicle | 1.57E-04 |
|  | GOTERM_CC_2 | GO:0031982~vesicle | 1.58E-04 |
|  | GOTERM_CC_5 | GO:0016021~integral to membrane | 2.12E-04 |
|  | GOTERM_BP_4 | GO:0009100~glycoprotein metabolic process | 2.14E-04 |
|  | GOTERM_MF_5 | GO:0008201~heparin binding | 2.85E-04 |
|  | GOTERM_BP_5 | GO:0006516~glycoprotein catabolic process | 3.06E-04 |
|  | GOTERM_BP_2 | GO:0006928~cell motion | 4.55E-04 |
|  | GOTERM_CC_4 | GO:0016021~integral to membrane | 4.56E-04 |
|  | GOTERM_BP_3 | GO:0006928~cell motion | 5.35E-04 |
|  | GOTERM_MF_4 | GO:0005539~glycosaminoglycan binding | 5.44E-04 |
|  | GOTERM_CC_5 | GO:0016023~cytoplasmic membrane-bounded vesicle | 5.89E-04 |
|  | GOTERM_CC_3 | GO:0031988~membrane-bounded vesicle | 6.79E-04 |
|  | GOTERM_CC_4 | GO:0016023~cytoplasmic membrane-bounded vesicle | 7.06E-04 |
|  | GOTERM_CC_2 | GO:0044425~membrane part | 8.36E-04 |
|  | GOTERM_CC_3 | GO:0044425~membrane part | 8.45E-04 |
|  | GOTERM_MF_3 | GO:0030247~polysaccharide binding | 9.70E-04 |
|  | GOTERM_MF_2 | GO:0001871~pattern binding | 0.001274 |
|  | GOTERM_MF_2 | GO:0030246~carbohydrate binding | 0.001387 |
|  | GOTERM_CC_4 | GO:0044425~membrane part | 0.001454 |
|  | GOTERM_CC_5 | GO:0030141~secretory granule | 0.001598 |
|  | GOTERM_CC_3 | GO:0044433~cytoplasmic vesicle part | 0.001763 |
|  | GOTERM_CC_5 | GO:0044433~cytoplasmic vesicle part | 0.001782 |
|  | GOTERM_CC_4 | GO:0044433~cytoplasmic vesicle part | 0.001981 |
|  | GOTERM_BP_5 | GO:0007166~cell surface receptor linked signal transduction | 0.002816 |
|  | GOTERM_BP_2 | GO:0050900~leukocyte migration | 0.002975 |
|  | GOTERM_CC_2 | GO:0016020~membrane | 0.003076 |
|  | GOTERM_BP_5 | GO:0050900~leukocyte migration | 0.003082 |
|  | GOTERM_CC_3 | GO:0016020~membrane | 0.003105 |
|  | GOTERM_BP_4 | GO:0050900~leukocyte migration | 0.003324 |
|  | GOTERM_BP_4 | GO:0007166~cell surface receptor linked signal transduction | 0.003968 |
|  | GOTERM_MF_4 | GO:0043208~glycosphingolipid binding | 0.004749 |
|  | GOTERM_MF_5 | GO:0004867~serine-type endopeptidase inhibitor activity | 0.006164 |
|  | GOTERM_BP_4 | GO:0043085~positive regulation of catalytic activity | 0.007024 |
|  | GOTERM_BP_5 | GO:0016477~cell migration | 0.007117 |
|  | GOTERM_BP_3 | GO:0016477~cell migration | 0.007504 |
|  | GOTERM_MF_3 | GO:0046625~sphingolipid binding | 0.007792 |
|  | GOTERM_BP_4 | GO:0016477~cell migration | 0.007964 |
|  | GOTERM_BP_5 | GO:0050901~leukocyte tethering or rolling | 0.008756 |
|  | GOTERM_BP_4 | GO:0050901~leukocyte tethering or rolling | 0.009076 |
|  | GOTERM_BP_2 | GO:0051674~localization of cell | 0.009125 |
|  | GOTERM_BP_2 | GO:0048870~cell motility | 0.009125 |
|  | GOTERM_BP_3 | GO:0044093~positive regulation of molecular function | 0.009867 |
|  | GOTERM_BP_3 | GO:0048870~cell motility | 0.01004 |
|  | GOTERM_BP_5 | GO:0044257~cellular protein catabolic process | 0.010121 |
|  | GOTERM_BP_4 | GO:0048870~cell motility | 0.010648 |
|  | GOTERM_BP_5 | GO:0012502~induction of programmed cell death | 0.010761 |
|  | GOTERM_BP_5 | GO:0030163~protein catabolic process | 0.011262 |
|  | GOTERM_BP_1 | GO:0002376~immune system process | 0.011302 |
|  | GOTERM_MF_3 | GO:0051861~glycolipid binding | 0.011666 |
|  | GOTERM_MF_4 | GO:0004866~endopeptidase inhibitor activity | 0.012442 |
|  | GOTERM_MF_2 | GO:0005515~protein binding | 0.01255 |
|  | GOTERM_BP_4 | GO:0030163~protein catabolic process | 0.013032 |
|  | GOTERM_BP_5 | GO:0006508~proteolysis | 0.015288 |
|  | GOTERM_MF_3 | GO:0030414~peptidase inhibitor activity | 0.016546 |
|  | GOTERM_MF_5 | GO:0000287~magnesium ion binding | 0.018907 |
|  | GOTERM_CC_5 | GO:0005624~membrane fraction | 0.019367 |
|  | GOTERM_CC_3 | GO:0005626~insoluble fraction | 0.021605 |
|  | GOTERM_BP_4 | GO:0044265~cellular macromolecule catabolic process | 0.021767 |
|  | GOTERM_CC_4 | GO:0005624~membrane fraction | 0.021935 |
|  | GOTERM_BP_1 | GO:0040011~locomotion | 0.022169 |
|  | GOTERM_BP_3 | GO:0032101~regulation of response to external stimulus | 0.02291 |
|  | GOTERM_BP_5 | GO:0043065~positive regulation of apoptosis | 0.023466 |
|  | GOTERM_BP_4 | GO:0032101~regulation of response to external stimulus | 0.023853 |
|  | GOTERM_BP_5 | GO:0043068~positive regulation of programmed cell death | 0.023896 |
|  | GOTERM_BP_5 | GO:0010942~positive regulation of cell death | 0.024185 |
|  | GOTERM_CC_4 | GO:0005626~insoluble fraction | 0.024717 |
|  | GOTERM_CC_5 | GO:0005794~Golgi apparatus | 0.0248 |
|  | GOTERM_BP_3 | GO:0010942~positive regulation of cell death | 0.025382 |
|  | GOTERM_BP_3 | GO:0009057~macromolecule catabolic process | 0.02587 |
|  | GOTERM_MF_3 | GO:0005529~sugar binding | 0.026068 |
|  | GOTERM_MF_5 | GO:0005509~calcium ion binding | 0.026177 |
|  | GOTERM_BP_4 | GO:0043068~positive regulation of programmed cell death | 0.02653 |
|  | GOTERM_BP_4 | GO:0010942~positive regulation of cell death | 0.026848 |
|  | GOTERM_CC_4 | GO:0005794~Golgi apparatus | 0.028019 |
|  | GOTERM_BP_2 | GO:0009056~catabolic process | 0.028754 |
|  | GOTERM_BP_4 | GO:0007157~heterophilic cell adhesion | 0.032898 |
|  | GOTERM_BP_3 | GO:0050790~regulation of catalytic activity | 0.033475 |
|  | GOTERM_MF_3 | GO:0008233~peptidase activity | 0.036331 |
|  | GOTERM_CC_5 | GO:0030667~secretory granule membrane | 0.037532 |
|  | GOTERM_BP_2 | GO:0009605~response to external stimulus | 0.038269 |
|  | GOTERM_BP_3 | GO:0009611~response to wounding | 0.042026 |
|  | GOTERM_BP_2 | GO:0065009~regulation of molecular function | 0.045949 |
|  | GOTERM_MF_3 | GO:0001948~glycoprotein binding | 0.047146 |
|  | GOTERM_CC_2 | GO:0000267~cell fraction | 0.048726 |
|  | GOTERM_CC_3 | GO:0000267~cell fraction | 0.048834 |
| 4842 | GOTERM_CC_4 | GO:0034703~cation channel complex | 1.26E-17 |
|  | GOTERM_CC_5 | GO:0034703~cation channel complex | 1.54E-17 |
|  | GOTERM_MF_2 | GO:0019825~oxygen binding | 1.14E-16 |
|  | GOTERM_MF_3 | GO:0020037~heme binding | 8.12E-16 |
|  | GOTERM_MF_2 | GO:0046906~tetrapyrrole binding | 1.01E-15 |
|  | GOTERM_CC_3 | GO:0034702~ion channel complex | 1.69E-15 |
|  | GOTERM_CC_4 | GO:0034702~ion channel complex | 2.74E-15 |
|  | GOTERM_CC_5 | GO:0034702~ion channel complex | 3.44E-15 |
|  | GOTERM_MF_5 | GO:0031420~alkali metal ion binding | 3.42E-13 |
|  | GOTERM_BP_5 | GO:0015672~monovalent inorganic cation transport | 5.11E-13 |
|  | GOTERM_MF_2 | GO:0022892~substrate-specific transporter activity | 6.68E-13 |
|  | GOTERM_CC_4 | GO:0008076~voltage-gated potassium channel complex | 5.28E-12 |
|  | GOTERM_CC_5 | GO:0034705~potassium channel complex | 6.01E-12 |
|  | GOTERM_CC_5 | GO:0008076~voltage-gated potassium channel complex | 6.01E-12 |
|  | GOTERM_MF_1 | GO:0005215~transporter activity | 1.13E-11 |
|  | GOTERM_MF_5 | GO:0022836~gated channel activity | 1.31E-11 |
|  | GOTERM_MF_3 | GO:0022803~passive transmembrane transporter activity | 2.74E-11 |
|  | GOTERM_BP_5 | GO:0030001~metal ion transport | 4.45E-11 |
|  | GOTERM_MF_4 | GO:0015267~channel activity | 8.71E-11 |
|  | GOTERM_MF_3 | GO:0043169~cation binding | 1.60E-10 |
|  | GOTERM_MF_5 | GO:0005216~ion channel activity | 1.69E-10 |
|  | GOTERM_MF_2 | GO:0043167~ion binding | 2.40E-10 |
|  | GOTERM_MF_5 | GO:0022838~substrate specific channel activity | 2.40E-10 |
|  | GOTERM_BP_5 | GO:0006812~cation transport | 3.31E-10 |
|  | GOTERM_BP_4 | GO:0006812~cation transport | 4.37E-10 |
|  | GOTERM_MF_5 | GO:0008324~cation transmembrane transporter activity | 7.87E-10 |
|  | GOTERM_MF_4 | GO:0046872~metal ion binding | 1.60E-09 |
|  | GOTERM_MF_4 | GO:0015075~ion transmembrane transporter activity | 4.46E-09 |
|  | GOTERM_CC_3 | GO:0005833~hemoglobin complex | 5.91E-09 |
|  | GOTERM_MF_3 | GO:0022891~substrate-specific transmembrane transporter activity | 6.35E-09 |
|  | GOTERM_CC_5 | GO:0005833~hemoglobin complex | 7.43E-09 |
|  | GOTERM_MF_2 | GO:0022857~transmembrane transporter activity | 1.12E-08 |
|  | GOTERM_MF_3 | GO:0016705~oxidoreductase activity, acting on paired donors, with incorporation or reduction of molecular oxygen | 1.40E-08 |
|  | GOTERM_BP_4 | GO:0006811~ion transport | 1.81E-08 |
|  | GOTERM_BP_3 | GO:0017144~drug metabolic process | 4.11E-08 |
|  | GOTERM_BP_3 | GO:0006811~ion transport | 5.63E-08 |
|  | GOTERM_CC_4 | GO:0005783~endoplasmic reticulum | 1.66E-07 |
|  | GOTERM_CC_5 | GO:0005783~endoplasmic reticulum | 1.99E-07 |
|  | GOTERM_BP_1 | GO:0051234~establishment of localization | 2.29E-07 |
|  | GOTERM_BP_2 | GO:0051234~establishment of localization | 2.59E-07 |
|  | GOTERM_CC_3 | GO:0044459~plasma membrane part | 6.40E-07 |
|  | GOTERM_CC_2 | GO:0043234~protein complex | 9.32E-07 |
|  | GOTERM_CC_4 | GO:0044459~plasma membrane part | 1.13E-06 |
|  | GOTERM_CC_5 | GO:0044459~plasma membrane part | 1.41E-06 |
|  | GOTERM_BP_2 | GO:0006810~transport | 1.42E-06 |
|  | GOTERM_BP_1 | GO:0051179~localization | 1.59E-06 |
|  | GOTERM_MF_3 | GO:0004497~monooxygenase activity | 2.11E-06 |
|  | GOTERM_BP_5 | GO:0015671~oxygen transport | 2.37E-06 |
|  | GOTERM_BP_3 | GO:0006810~transport | 2.41E-06 |
|  | GOTERM_BP_4 | GO:0015671~oxygen transport | 2.42E-06 |
|  | GOTERM_CC_3 | GO:0005626~insoluble fraction | 2.59E-06 |
|  | GOTERM_CC_4 | GO:0005624~membrane fraction | 2.70E-06 |
|  | GOTERM_MF_3 | GO:0005344~oxygen transporter activity | 2.78E-06 |
|  | GOTERM_CC_5 | GO:0005624~membrane fraction | 3.13E-06 |
|  | GOTERM_CC_5 | GO:0034706~sodium channel complex | 3.63E-06 |
|  | GOTERM_CC_4 | GO:0005626~insoluble fraction | 3.74E-06 |
|  | GOTERM_MF_1 | GO:0009055~electron carrier activity | 4.91E-06 |
|  | GOTERM_CC_2 | GO:0044425~membrane part | 6.75E-06 |
|  | GOTERM_BP_2 | GO:0055114~oxidation reduction | 6.85E-06 |
|  | GOTERM_CC_3 | GO:0044425~membrane part | 6.86E-06 |
|  | GOTERM_BP_4 | GO:0015669~gas transport | 6.87E-06 |
|  | GOTERM_MF_2 | GO:0016491~oxidoreductase activity | 7.70E-06 |
|  | GOTERM_BP_3 | GO:0015669~gas transport | 8.81E-06 |
|  | GOTERM_MF_4 | GO:0050699~WW domain binding | 8.93E-06 |
|  | GOTERM_CC_1 | GO:0032991~macromolecular complex | 9.10E-06 |
|  | GOTERM_CC_5 | GO:0042598~vesicular fraction | 9.30E-06 |
|  | GOTERM_CC_4 | GO:0044425~membrane part | 1.65E-05 |
|  | GOTERM_BP_4 | GO:0055085~transmembrane transport | 1.66E-05 |
|  | GOTERM_CC_2 | GO:0000267~cell fraction | 2.47E-05 |
|  | GOTERM_CC_3 | GO:0000267~cell fraction | 2.48E-05 |
|  | GOTERM_BP_2 | GO:0055085~transmembrane transport | 2.51E-05 |
|  | GOTERM_BP_3 | GO:0055085~transmembrane transport | 3.23E-05 |
|  | GOTERM_MF_4 | GO:0016709~oxidoreductase activity, acting on paired donors, with incorporation or reduction of molecular oxygen, NADH or NADPH as one donor, and incorporation of one atom of oxygen | 4.22E-05 |
|  | GOTERM_MF_4 | GO:0016712~oxidoreductase activity, acting on paired donors, with incorporation or reduction of molecular oxygen, reduced flavin or flavoprotein as one donor, and incorporation of one atom of oxygen | 5.22E-05 |
|  | GOTERM_CC_2 | GO:0016020~membrane | 5.58E-05 |
|  | GOTERM_CC_3 | GO:0016020~membrane | 5.66E-05 |
|  | GOTERM_CC_5 | GO:0005887~integral to plasma membrane | 8.97E-05 |
|  | GOTERM_CC_4 | GO:0031226~intrinsic to plasma membrane | 9.44E-05 |
|  | GOTERM_CC_5 | GO:0031226~intrinsic to plasma membrane | 1.08E-04 |
|  | GOTERM_CC_3 | GO:0005789~endoplasmic reticulum membrane | 1.62E-04 |
|  | GOTERM_CC_4 | GO:0005789~endoplasmic reticulum membrane | 1.96E-04 |
|  | GOTERM_CC_3 | GO:0042175~nuclear envelope-endoplasmic reticulum network | 2.08E-04 |
|  | GOTERM_CC_5 | GO:0005789~endoplasmic reticulum membrane | 2.11E-04 |
|  | GOTERM_BP_5 | GO:0007588~excretion | 2.40E-04 |
|  | GOTERM_BP_4 | GO:0007588~excretion | 2.45E-04 |
|  | GOTERM_CC_4 | GO:0044445~cytosolic part | 2.46E-04 |
|  | GOTERM_CC_4 | GO:0042175~nuclear envelope-endoplasmic reticulum network | 2.52E-04 |
|  | GOTERM_CC_5 | GO:0044445~cytosolic part | 2.62E-04 |
|  | GOTERM_CC_5 | GO:0042175~nuclear envelope-endoplasmic reticulum network | 2.72E-04 |
|  | GOTERM_BP_3 | GO:0007588~excretion | 3.13E-04 |
|  | GOTERM_CC_3 | GO:0044444~cytoplasmic part | 4.14E-04 |
|  | GOTERM_CC_3 | GO:0044432~endoplasmic reticulum part | 5.24E-04 |
|  | GOTERM_CC_4 | GO:0044432~endoplasmic reticulum part | 6.30E-04 |
|  | GOTERM_CC_5 | GO:0044432~endoplasmic reticulum part | 6.79E-04 |
|  | GOTERM_CC_3 | GO:0005886~plasma membrane | 7.06E-04 |
|  | GOTERM_CC_4 | GO:0044444~cytoplasmic part | 7.40E-04 |
|  | GOTERM_CC_5 | GO:0044444~cytoplasmic part | 9.33E-04 |
|  | GOTERM_CC_4 | GO:0005886~plasma membrane | 0.001144 |
|  | GOTERM_BP_5 | GO:0006778~porphyrin metabolic process | 0.001686 |
|  | GOTERM_BP_4 | GO:0033013~tetrapyrrole metabolic process | 0.001707 |
|  | GOTERM_BP_4 | GO:0006778~porphyrin metabolic process | 0.001707 |
|  | GOTERM_MF_5 | GO:0070330~aromatase activity | 0.002198 |
|  | GOTERM_BP_4 | GO:0001666~response to hypoxia | 0.002799 |
|  | GOTERM_BP_3 | GO:0001666~response to hypoxia | 0.003535 |
|  | GOTERM_CC_2 | GO:0012505~endomembrane system | 0.003625 |
|  | GOTERM_CC_3 | GO:0012505~endomembrane system | 0.003638 |
|  | GOTERM_BP_3 | GO:0070482~response to oxygen levels | 0.004078 |
|  | GOTERM_BP_5 | GO:0018894~dibenzo-p-dioxin metabolic process | 0.004226 |
|  | GOTERM_BP_4 | GO:0018894~dibenzo-p-dioxin metabolic process | 0.004246 |
|  | GOTERM_CC_2 | GO:0031090~organelle membrane | 0.004499 |
|  | GOTERM_CC_3 | GO:0031090~organelle membrane | 0.004517 |
|  | GOTERM_BP_3 | GO:0018894~dibenzo-p-dioxin metabolic process | 0.0046 |
|  | GOTERM_MF_5 | GO:0033767~4-hydroxyacetophenone monooxygenase activity | 0.005616 |
|  | GOTERM_CC_4 | GO:0031090~organelle membrane | 0.005657 |
|  | GOTERM_CC_3 | GO:0031224~intrinsic to membrane | 0.006059 |
|  | GOTERM_CC_4 | GO:0031224~intrinsic to membrane | 0.009853 |
|  | GOTERM_BP_5 | GO:0042537~benzene and derivative metabolic process | 0.010533 |
|  | GOTERM_BP_4 | GO:0042537~benzene and derivative metabolic process | 0.010584 |
|  | GOTERM_BP_4 | GO:0009404~toxin metabolic process | 0.010584 |
|  | GOTERM_BP_3 | GO:0009404~toxin metabolic process | 0.011462 |
|  | GOTERM_CC_5 | GO:0031224~intrinsic to membrane | 0.011935 |
|  | GOTERM_BP_5 | GO:0050665~hydrogen peroxide biosynthetic process | 0.014716 |
|  | GOTERM_BP_4 | GO:0050665~hydrogen peroxide biosynthetic process | 0.014787 |
|  | GOTERM_CC_3 | GO:0019898~extrinsic to membrane | 0.015184 |
|  | GOTERM_BP_5 | GO:0035313~wound healing, spreading of epidermal cells | 0.016802 |
|  | GOTERM_BP_4 | GO:0035313~wound healing, spreading of epidermal cells | 0.016882 |
|  | GOTERM_CC_4 | GO:0016021~integral to membrane | 0.017167 |
|  | GOTERM_CC_4 | GO:0019898~extrinsic to membrane | 0.017403 |
|  | GOTERM_CC_5 | GO:0019898~extrinsic to membrane | 0.018382 |
|  | GOTERM_CC_5 | GO:0016021~integral to membrane | 0.020416 |
|  | GOTERM_MF_2 | GO:0032451~demethylase activity | 0.020721 |
|  | GOTERM_BP_4 | GO:0046903~secretion | 0.025155 |
|  | GOTERM_CC_3 | GO:0016324~apical plasma membrane | 0.027891 |
|  | GOTERM_MF_5 | GO:0046914~transition metal ion binding | 0.028347 |
|  | GOTERM_CC_4 | GO:0016324~apical plasma membrane | 0.030079 |
|  | GOTERM_BP_4 | GO:0008610~lipid biosynthetic process | 0.030418 |
|  | GOTERM_CC_5 | GO:0016324~apical plasma membrane | 0.031007 |
|  | GOTERM_BP_3 | GO:0046903~secretion | 0.031083 |
|  | GOTERM_MF_3 | GO:0019904~protein domain specific binding | 0.036307 |
|  | GOTERM_MF_4 | GO:0008395~steroid hydroxylase activity | 0.03634 |
|  | GOTERM_BP_3 | GO:0008610~lipid biosynthetic process | 0.037476 |
|  | GOTERM_BP_4 | GO:0006805~xenobiotic metabolic process | 0.04169 |
|  | GOTERM_CC_3 | GO:0009897~external side of plasma membrane | 0.043657 |
|  | GOTERM_BP_3 | GO:0006805~xenobiotic metabolic process | 0.04509 |
|  | GOTERM_CC_4 | GO:0009897~external side of plasma membrane | 0.046996 |
|  | GOTERM_CC_2 | GO:0045177~apical part of cell | 0.047835 |
|  | GOTERM_CC_3 | GO:0045177~apical part of cell | 0.047897 |
|  | GOTERM_CC_5 | GO:0009897~external side of plasma membrane | 0.048409 |
|  | GOTERM_CC_4 | GO:0005829~cytosol | 0.049371 |
| 4878 | GOTERM_CC_1 | GO:0005576~extracellular region | 2.36E-19 |
|  | GOTERM_MF_3 | GO:0030414~peptidase inhibitor activity | 2.17E-17 |
|  | GOTERM_MF_4 | GO:0004866~endopeptidase inhibitor activity | 2.43E-17 |
|  | GOTERM_BP_3 | GO:0009611~response to wounding | 3.32E-17 |
|  | GOTERM_MF_2 | GO:0004857~enzyme inhibitor activity | 1.85E-16 |
|  | GOTERM_BP_2 | GO:0050817~coagulation | 5.04E-15 |
|  | GOTERM_BP_3 | GO:0007596~blood coagulation | 7.08E-15 |
|  | GOTERM_BP_4 | GO:0007596~blood coagulation | 8.75E-15 |
|  | GOTERM_BP_3 | GO:0007599~hemostasis | 1.27E-14 |
|  | GOTERM_BP_4 | GO:0007599~hemostasis | 1.58E-14 |
|  | GOTERM_BP_5 | GO:0007596~blood coagulation | 1.73E-14 |
|  | GOTERM_CC_2 | GO:0005615~extracellular space | 1.79E-14 |
|  | GOTERM_CC_3 | GO:0005615~extracellular space | 1.81E-14 |
|  | GOTERM_MF_5 | GO:0004867~serine-type endopeptidase inhibitor activity | 1.04E-13 |
|  | GOTERM_BP_2 | GO:0050878~regulation of body fluid levels | 1.38E-13 |
|  | GOTERM_BP_2 | GO:0009605~response to external stimulus | 1.62E-13 |
|  | GOTERM_CC_2 | GO:0044421~extracellular region part | 1.75E-13 |
|  | GOTERM_BP_3 | GO:0050878~regulation of body fluid levels | 1.94E-13 |
|  | GOTERM_CC_1 | GO:0044421~extracellular region part | 7.58E-13 |
|  | GOTERM_BP_4 | GO:0042060~wound healing | 5.11E-12 |
|  | GOTERM_MF_1 | GO:0030234~enzyme regulator activity | 7.09E-10 |
|  | GOTERM_BP_2 | GO:0006950~response to stress | 2.86E-09 |
|  | GOTERM_BP_2 | GO:0065008~regulation of biological quality | 3.79E-09 |
|  | GOTERM_CC_5 | GO:0031093~platelet alpha granule lumen | 1.30E-07 |
|  | GOTERM_MF_4 | GO:0002020~protease binding | 1.69E-07 |
|  | GOTERM_CC_5 | GO:0060205~cytoplasmic membrane-bounded vesicle lumen | 1.74E-07 |
|  | GOTERM_CC_4 | GO:0060205~cytoplasmic membrane-bounded vesicle lumen | 5.98E-07 |
|  | GOTERM_CC_4 | GO:0031983~vesicle lumen | 7.18E-07 |
|  | GOTERM_BP_1 | GO:0050896~response to stimulus | 7.32E-07 |
|  | GOTERM_MF_3 | GO:0008233~peptidase activity | 9.27E-07 |
|  | GOTERM_CC_3 | GO:0031983~vesicle lumen | 1.86E-06 |
|  | GOTERM_BP_1 | GO:0032501~multicellular organismal process | 3.91E-06 |
|  | GOTERM_BP_4 | GO:0006954~inflammatory response | 5.10E-06 |
|  | GOTERM_MF_5 | GO:0008009~chemokine activity | 1.14E-05 |
|  | GOTERM_MF_5 | GO:0004175~endopeptidase activity | 1.38E-05 |
|  | GOTERM_MF_5 | GO:0042379~chemokine receptor binding | 1.48E-05 |
|  | GOTERM_CC_5 | GO:0030141~secretory granule | 4.91E-05 |
|  | GOTERM_CC_5 | GO:0044433~cytoplasmic vesicle part | 5.70E-05 |
|  | GOTERM_MF_3 | GO:0017171~serine hydrolase activity | 8.19E-05 |
|  | GOTERM_MF_4 | GO:0070011~peptidase activity, acting on L-amino acid peptides | 8.27E-05 |
|  | GOTERM_MF_5 | GO:0004252~serine-type endopeptidase activity | 1.02E-04 |
|  | GOTERM_MF_4 | GO:0008236~serine-type peptidase activity | 1.08E-04 |
|  | GOTERM_BP_2 | GO:0042221~response to chemical stimulus | 1.62E-04 |
|  | GOTERM_CC_4 | GO:0044433~cytoplasmic vesicle part | 1.87E-04 |
|  | GOTERM_MF_5 | GO:0008236~serine-type peptidase activity | 2.01E-04 |
|  | GOTERM_MF_4 | GO:0001664~G-protein-coupled receptor binding | 2.20E-04 |
|  | GOTERM_BP_3 | GO:0006952~defense response | 2.60E-04 |
|  | GOTERM_MF_5 | GO:0008201~heparin binding | 2.74E-04 |
|  | GOTERM_MF_5 | GO:0005509~calcium ion binding | 3.12E-04 |
|  | GOTERM_BP_2 | GO:0042330~taxis | 3.17E-04 |
|  | GOTERM_BP_3 | GO:0032101~regulation of response to external stimulus | 3.53E-04 |
|  | GOTERM_BP_3 | GO:0006935~chemotaxis | 3.62E-04 |
|  | GOTERM_BP_3 | GO:0042330~taxis | 3.62E-04 |
|  | GOTERM_MF_2 | GO:0001871~pattern binding | 3.62E-04 |
|  | GOTERM_BP_4 | GO:0032101~regulation of response to external stimulus | 3.84E-04 |
|  | GOTERM_BP_4 | GO:0042330~taxis | 3.93E-04 |
|  | GOTERM_BP_4 | GO:0006935~chemotaxis | 3.93E-04 |
|  | GOTERM_CC_3 | GO:0044433~cytoplasmic vesicle part | 4.64E-04 |
|  | GOTERM_BP_5 | GO:0006935~chemotaxis | 5.09E-04 |
|  | GOTERM_MF_4 | GO:0005539~glycosaminoglycan binding | 5.32E-04 |
|  | GOTERM_MF_3 | GO:0030247~polysaccharide binding | 5.84E-04 |
|  | GOTERM_BP_4 | GO:0030574~collagen catabolic process | 8.67E-04 |
|  | GOTERM_BP_5 | GO:0030574~collagen catabolic process | 9.95E-04 |
|  | GOTERM_BP_3 | GO:0044243~multicellular organismal catabolic process | 0.00141 |
|  | GOTERM_BP_4 | GO:0032963~collagen metabolic process | 0.001707 |
|  | GOTERM_BP_1 | GO:0040011~locomotion | 0.001714 |
|  | GOTERM_MF_4 | GO:0005125~cytokine activity | 0.001825 |
|  | GOTERM_BP_3 | GO:0044259~multicellular organismal macromolecule metabolic process | 0.002004 |
|  | GOTERM_BP_5 | GO:0006508~proteolysis | 0.002387 |
|  | GOTERM_BP_2 | GO:0044236~multicellular organismal metabolic process | 0.002658 |
|  | GOTERM_BP_3 | GO:0007626~locomotory behavior | 0.00267 |
|  | GOTERM_CC_5 | GO:0016023~cytoplasmic membrane-bounded vesicle | 0.003346 |
|  | GOTERM_BP_1 | GO:0065007~biological regulation | 0.003415 |
|  | GOTERM_CC_2 | GO:0031012~extracellular matrix | 0.004387 |
|  | GOTERM_CC_3 | GO:0031012~extracellular matrix | 0.004398 |
|  | GOTERM_CC_5 | GO:0031410~cytoplasmic vesicle | 0.005816 |
|  | GOTERM_BP_5 | GO:0030162~regulation of proteolysis | 0.006874 |
|  | GOTERM_MF_2 | GO:0030246~carbohydrate binding | 0.007572 |
|  | GOTERM_CC_4 | GO:0016023~cytoplasmic membrane-bounded vesicle | 0.009817 |
|  | GOTERM_BP_4 | GO:0007598~blood coagulation, extrinsic pathway | 0.01096 |
|  | GOTERM_BP_5 | GO:0007598~blood coagulation, extrinsic pathway | 0.011743 |
|  | GOTERM_BP_4 | GO:0032496~response to lipopolysaccharide | 0.012362 |
|  | GOTERM_BP_2 | GO:0006955~immune response | 0.012923 |
|  | GOTERM_CC_4 | GO:0005578~proteinaceous extracellular matrix | 0.013184 |
|  | GOTERM_BP_5 | GO:0032496~response to lipopolysaccharide | 0.014096 |
|  | GOTERM_BP_3 | GO:0002237~response to molecule of bacterial origin | 0.014642 |
|  | GOTERM_BP_2 | GO:0048583~regulation of response to stimulus | 0.014972 |
|  | GOTERM_BP_2 | GO:0007586~digestion | 0.015252 |
|  | GOTERM_BP_4 | GO:0002237~response to molecule of bacterial origin | 0.015254 |
|  | GOTERM_BP_2 | GO:0007610~behavior | 0.015407 |
|  | GOTERM_CC_4 | GO:0031410~cytoplasmic vesicle | 0.016584 |
|  | GOTERM_BP_3 | GO:0048583~regulation of response to stimulus | 0.016832 |
|  | GOTERM_BP_5 | GO:0002237~response to molecule of bacterial origin | 0.01738 |
|  | GOTERM_BP_3 | GO:0080134~regulation of response to stress | 0.020619 |
|  | GOTERM_BP_4 | GO:0080134~regulation of response to stress | 0.021819 |
|  | GOTERM_BP_2 | GO:0051707~response to other organism | 0.02182 |
|  | GOTERM_BP_5 | GO:0002526~acute inflammatory response | 0.022211 |
|  | GOTERM_MF_2 | GO:0016787~hydrolase activity | 0.022963 |
|  | GOTERM_BP_3 | GO:0051707~response to other organism | 0.023907 |
|  | GOTERM_CC_3 | GO:0031988~membrane-bounded vesicle | 0.024044 |
|  | GOTERM_CC_3 | GO:0005578~proteinaceous extracellular matrix | 0.02432 |
|  | GOTERM_CC_5 | GO:0005737~cytoplasm | 0.02811 |
|  | GOTERM_CC_3 | GO:0031410~cytoplasmic vesicle | 0.035546 |
|  | GOTERM_CC_2 | GO:0031982~vesicle | 0.040524 |
|  | GOTERM_MF_3 | GO:0019899~enzyme binding | 0.041625 |
|  | GOTERM_BP_2 | GO:0009607~response to biotic stimulus | 0.044615 |
|  | GOTERM_BP_1 | GO:0051704~multi-organism process | 0.049649 |
| 4944 | GOTERM_BP_3 | GO:0032101~regulation of response to external stimulus | 6.41E-06 |
|  | GOTERM_BP_4 | GO:0032101~regulation of response to external stimulus | 6.84E-06 |
|  | GOTERM_BP_2 | GO:0009605~response to external stimulus | 1.83E-05 |
|  | GOTERM_BP_2 | GO:0048583~regulation of response to stimulus | 1.44E-04 |
|  | GOTERM_BP_3 | GO:0048583~regulation of response to stimulus | 1.60E-04 |
|  | GOTERM_BP_3 | GO:0009611~response to wounding | 2.36E-04 |
|  | GOTERM_CC_1 | GO:0005576~extracellular region | 2.54E-04 |
|  | GOTERM_BP_4 | GO:0051246~regulation of protein metabolic process | 2.75E-04 |
|  | GOTERM_BP_2 | GO:0050817~coagulation | 3.15E-04 |
|  | GOTERM_BP_5 | GO:0051246~regulation of protein metabolic process | 3.37E-04 |
|  | GOTERM_BP_3 | GO:0007596~blood coagulation | 3.38E-04 |
|  | GOTERM_CC_2 | GO:0005615~extracellular space | 3.46E-04 |
|  | GOTERM_CC_3 | GO:0005615~extracellular space | 3.47E-04 |
|  | GOTERM_BP_4 | GO:0007596~blood coagulation | 3.53E-04 |
|  | GOTERM_BP_3 | GO:0007599~hemostasis | 3.79E-04 |
|  | GOTERM_BP_4 | GO:0007599~hemostasis | 3.95E-04 |
|  | GOTERM_BP_5 | GO:0007596~blood coagulation | 4.05E-04 |
|  | GOTERM_CC_4 | GO:0005896~interleukin-6 receptor complex | 4.09E-04 |
|  | GOTERM_CC_5 | GO:0005896~interleukin-6 receptor complex | 4.16E-04 |
|  | GOTERM_BP_2 | GO:0050878~regulation of body fluid levels | 6.01E-04 |
|  | GOTERM_MF_5 | GO:0004915~interleukin-6 receptor activity | 6.02E-04 |
|  | GOTERM_BP_3 | GO:0050878~regulation of body fluid levels | 6.45E-04 |
|  | GOTERM_MF_4 | GO:0019981~interleukin-6 binding | 6.79E-04 |
|  | GOTERM_BP_2 | GO:0051241~negative regulation of multicellular organismal process | 8.12E-04 |
|  | GOTERM_CC_1 | GO:0044421~extracellular region part | 8.37E-04 |
|  | GOTERM_BP_3 | GO:0051241~negative regulation of multicellular organismal process | 8.71E-04 |
|  | GOTERM_BP_4 | GO:0051241~negative regulation of multicellular organismal process | 9.09E-04 |
|  | GOTERM_CC_2 | GO:0044421~extracellular region part | 9.40E-04 |
|  | GOTERM_BP_2 | GO:0051239~regulation of multicellular organismal process | 0.001149 |
|  | GOTERM_BP_4 | GO:0042060~wound healing | 0.001231 |
|  | GOTERM_BP_3 | GO:0051239~regulation of multicellular organismal process | 0.001275 |
|  | GOTERM_BP_2 | GO:0051240~positive regulation of multicellular organismal process | 0.001787 |
|  | GOTERM_BP_4 | GO:0032270~positive regulation of cellular protein metabolic process | 0.001826 |
|  | GOTERM_BP_3 | GO:0051240~positive regulation of multicellular organismal process | 0.001916 |
|  | GOTERM_BP_4 | GO:0051247~positive regulation of protein metabolic process | 0.001984 |
|  | GOTERM_BP_4 | GO:0051240~positive regulation of multicellular organismal process | 0.002 |
|  | GOTERM_BP_5 | GO:0032270~positive regulation of cellular protein metabolic process | 0.002093 |
|  | GOTERM_MF_5 | GO:0005138~interleukin-6 receptor binding | 0.002108 |
|  | GOTERM_BP_5 | GO:0051247~positive regulation of protein metabolic process | 0.002275 |
|  | GOTERM_BP_3 | GO:0080134~regulation of response to stress | 0.00241 |
|  | GOTERM_BP_4 | GO:0080134~regulation of response to stress | 0.002516 |
|  | GOTERM_BP_2 | GO:0042221~response to chemical stimulus | 0.002881 |
|  | GOTERM_CC_2 | GO:0009986~cell surface | 0.003005 |
|  | GOTERM_CC_3 | GO:0009986~cell surface | 0.003009 |
|  | GOTERM_BP_4 | GO:0006954~inflammatory response | 0.003523 |
|  | GOTERM_BP_1 | GO:0050896~response to stimulus | 0.003854 |
|  | GOTERM_BP_2 | GO:0065008~regulation of biological quality | 0.004299 |
|  | GOTERM_BP_2 | GO:0006950~response to stress | 0.006408 |
|  | GOTERM_BP_5 | GO:0002673~regulation of acute inflammatory response | 0.006817 |
|  | GOTERM_BP_4 | GO:0030195~negative regulation of blood coagulation | 0.006966 |
|  | GOTERM_BP_4 | GO:0032268~regulation of cellular protein metabolic process | 0.007386 |
|  | GOTERM_BP_5 | GO:0030195~negative regulation of blood coagulation | 0.007464 |
|  | GOTERM_BP_4 | GO:0045669~positive regulation of osteoblast differentiation | 0.00757 |
|  | GOTERM_BP_3 | GO:0050819~negative regulation of coagulation | 0.007703 |
|  | GOTERM_BP_4 | GO:0050819~negative regulation of coagulation | 0.007872 |
|  | GOTERM_BP_2 | GO:0048519~negative regulation of biological process | 0.00791 |
|  | GOTERM_BP_5 | GO:0045669~positive regulation of osteoblast differentiation | 0.008112 |
|  | GOTERM_BP_2 | GO:0048522~positive regulation of cellular process | 0.00836 |
|  | GOTERM_BP_5 | GO:0050819~negative regulation of coagulation | 0.008435 |
|  | GOTERM_BP_5 | GO:0032268~regulation of cellular protein metabolic process | 0.008455 |
|  | GOTERM_BP_2 | GO:0045768~positive regulation of anti-apoptosis | 0.008578 |
|  | GOTERM_BP_3 | GO:0048519~negative regulation of biological process | 0.008757 |
|  | GOTERM_BP_3 | GO:0045768~positive regulation of anti-apoptosis | 0.008885 |
|  | GOTERM_BP_4 | GO:0045768~positive regulation of anti-apoptosis | 0.009079 |
|  | GOTERM_BP_3 | GO:0048522~positive regulation of cellular process | 0.009254 |
|  | GOTERM_BP_4 | GO:0048522~positive regulation of cellular process | 0.009852 |
|  | GOTERM_BP_2 | GO:0045767~regulation of anti-apoptosis | 0.010857 |
|  | GOTERM_BP_4 | GO:0030193~regulation of blood coagulation | 0.010887 |
|  | GOTERM_BP_2 | GO:0048518~positive regulation of biological process | 0.011027 |
|  | GOTERM_BP_3 | GO:0045767~regulation of anti-apoptosis | 0.011244 |
|  | GOTERM_BP_5 | GO:0030193~regulation of blood coagulation | 0.011665 |
|  | GOTERM_BP_3 | GO:0006952~defense response | 0.011755 |
|  | GOTERM_BP_3 | GO:0050818~regulation of coagulation | 0.012127 |
|  | GOTERM_BP_3 | GO:0048518~positive regulation of biological process | 0.0122 |
|  | GOTERM_BP_4 | GO:0050818~regulation of coagulation | 0.012392 |
|  | GOTERM_BP_4 | GO:0045667~regulation of osteoblast differentiation | 0.012994 |
|  | GOTERM_BP_5 | GO:0045667~regulation of osteoblast differentiation | 0.013921 |
|  | GOTERM_BP_3 | GO:0010033~response to organic substance | 0.015986 |
|  | GOTERM_MF_5 | GO:0004896~cytokine receptor activity | 0.01648 |
|  | GOTERM_BP_5 | GO:0030162~regulation of proteolysis | 0.017138 |
|  | GOTERM_MF_4 | GO:0004896~cytokine receptor activity | 0.018559 |
|  | GOTERM_BP_3 | GO:0032103~positive regulation of response to external stimulus | 0.018882 |
|  | GOTERM_BP_4 | GO:0032103~positive regulation of response to external stimulus | 0.019293 |
|  | GOTERM_BP_5 | GO:0032103~positive regulation of response to external stimulus | 0.020667 |
|  | GOTERM_BP_3 | GO:0010604~positive regulation of macromolecule metabolic process | 0.022278 |
|  | GOTERM_BP_5 | GO:0019221~cytokine-mediated signaling pathway | 0.022588 |
|  | GOTERM_BP_4 | GO:0050727~regulation of inflammatory response | 0.022879 |
|  | GOTERM_BP_3 | GO:0030278~regulation of ossification | 0.022977 |
|  | GOTERM_BP_4 | GO:0010604~positive regulation of macromolecule metabolic process | 0.023221 |
|  | GOTERM_BP_3 | GO:0034097~response to cytokine stimulus | 0.023269 |
|  | GOTERM_BP_3 | GO:0031325~positive regulation of cellular metabolic process | 0.023435 |
|  | GOTERM_BP_4 | GO:0030278~regulation of ossification | 0.023476 |
|  | GOTERM_BP_2 | GO:0009893~positive regulation of metabolic process | 0.023904 |
|  | GOTERM_BP_4 | GO:0031325~positive regulation of cellular metabolic process | 0.024426 |
|  | GOTERM_BP_5 | GO:0050727~regulation of inflammatory response | 0.024506 |
|  | GOTERM_BP_5 | GO:0030278~regulation of ossification | 0.025145 |
|  | GOTERM_MF_3 | GO:0005102~receptor binding | 0.025191 |
|  | GOTERM_BP_3 | GO:0009893~positive regulation of metabolic process | 0.025563 |
|  | GOTERM_BP_3 | GO:0001819~positive regulation of cytokine production | 0.026476 |
|  | GOTERM_BP_5 | GO:0010604~positive regulation of macromolecule metabolic process | 0.026502 |
|  | GOTERM_BP_4 | GO:0009893~positive regulation of metabolic process | 0.026641 |
|  | GOTERM_BP_4 | GO:0030335~positive regulation of cell migration | 0.026753 |
|  | GOTERM_BP_4 | GO:0001819~positive regulation of cytokine production | 0.027051 |
|  | GOTERM_BP_2 | GO:0040017~positive regulation of locomotion | 0.027818 |
|  | GOTERM_BP_5 | GO:0031325~positive regulation of cellular metabolic process | 0.027872 |
|  | GOTERM_BP_5 | GO:0030335~positive regulation of cell migration | 0.028652 |
|  | GOTERM_BP_3 | GO:0040017~positive regulation of locomotion | 0.028804 |
|  | GOTERM_BP_3 | GO:0051272~positive regulation of cell motion | 0.028804 |
|  | GOTERM_BP_5 | GO:0001819~positive regulation of cytokine production | 0.028971 |
|  | GOTERM_BP_4 | GO:0040017~positive regulation of locomotion | 0.029429 |
|  | GOTERM_BP_4 | GO:0051272~positive regulation of cell motion | 0.029429 |
|  | GOTERM_CC_3 | GO:0043235~receptor complex | 0.03002 |
|  | GOTERM_BP_4 | GO:0010562~positive regulation of phosphorus metabolic process | 0.030022 |
|  | GOTERM_BP_5 | GO:0051272~positive regulation of cell motion | 0.031515 |
|  | GOTERM_BP_5 | GO:0002526~acute inflammatory response | 0.031515 |
|  | GOTERM_MF_3 | GO:0019838~growth factor binding | 0.031799 |
|  | GOTERM_BP_5 | GO:0010562~positive regulation of phosphorus metabolic process | 0.03215 |
|  | GOTERM_BP_5 | GO:0045937~positive regulation of phosphate metabolic process | 0.03215 |
|  | GOTERM_MF_3 | GO:0019955~cytokine binding | 0.032995 |
|  | GOTERM_BP_4 | GO:0031347~regulation of defense response | 0.042722 |
|  | GOTERM_BP_2 | GO:0042330~taxis | 0.045116 |
|  | GOTERM_BP_5 | GO:0031347~regulation of defense response | 0.045734 |
|  | GOTERM_BP_3 | GO:0060255~regulation of macromolecule metabolic process | 0.046413 |
|  | GOTERM_BP_3 | GO:0006935~chemotaxis | 0.046702 |
|  | GOTERM_BP_3 | GO:0042330~taxis | 0.046702 |
|  | GOTERM_BP_3 | GO:0080090~regulation of primary metabolic process | 0.04769 |
|  | GOTERM_BP_4 | GO:0042330~taxis | 0.047708 |
|  | GOTERM_BP_4 | GO:0006935~chemotaxis | 0.047708 |
|  | GOTERM_BP_4 | GO:0046486~glycerolipid metabolic process | 0.048293 |
|  | GOTERM_BP_3 | GO:0030334~regulation of cell migration | 0.04928 |
|  | GOTERM_BP_4 | GO:0060255~regulation of macromolecule metabolic process | 0.049295 |
| 4958 | GOTERM_CC_2 | GO:0005615~extracellular space | 3.74E-08 |
|  | GOTERM_CC_3 | GO:0005615~extracellular space | 3.76E-08 |
|  | GOTERM_BP_3 | GO:0008284~positive regulation of cell proliferation | 6.34E-08 |
|  | GOTERM_BP_4 | GO:0008284~positive regulation of cell proliferation | 7.20E-08 |
|  | GOTERM_BP_3 | GO:0042127~regulation of cell proliferation | 7.38E-08 |
|  | GOTERM_BP_4 | GO:0042127~regulation of cell proliferation | 8.57E-08 |
|  | GOTERM_BP_5 | GO:0008284~positive regulation of cell proliferation | 1.09E-07 |
|  | GOTERM_MF_4 | GO:0005125~cytokine activity | 1.41E-07 |
|  | GOTERM_BP_4 | GO:0032270~positive regulation of cellular protein metabolic process | 1.97E-07 |
|  | GOTERM_BP_4 | GO:0051247~positive regulation of protein metabolic process | 2.43E-07 |
|  | GOTERM_BP_5 | GO:0032270~positive regulation of cellular protein metabolic process | 2.78E-07 |
|  | GOTERM_CC_1 | GO:0044421~extracellular region part | 2.92E-07 |
|  | GOTERM_BP_5 | GO:0051247~positive regulation of protein metabolic process | 3.42E-07 |
|  | GOTERM_CC_2 | GO:0044421~extracellular region part | 3.81E-07 |
|  | GOTERM_BP_4 | GO:0010562~positive regulation of phosphorus metabolic process | 3.83E-07 |
|  | GOTERM_BP_5 | GO:0010562~positive regulation of phosphorus metabolic process | 5.04E-07 |
|  | GOTERM_BP_5 | GO:0045937~positive regulation of phosphate metabolic process | 5.04E-07 |
|  | GOTERM_MF_3 | GO:0005102~receptor binding | 6.50E-07 |
|  | GOTERM_BP_2 | GO:0048522~positive regulation of cellular process | 7.43E-07 |
|  | GOTERM_BP_3 | GO:0048522~positive regulation of cellular process | 9.79E-07 |
|  | GOTERM_BP_4 | GO:0048522~positive regulation of cellular process | 1.16E-06 |
|  | GOTERM_BP_2 | GO:0048518~positive regulation of biological process | 1.58E-06 |
|  | GOTERM_BP_3 | GO:0048518~positive regulation of biological process | 2.08E-06 |
|  | GOTERM_BP_5 | GO:0010740~positive regulation of protein kinase cascade | 3.93E-06 |
|  | GOTERM_BP_3 | GO:0010604~positive regulation of macromolecule metabolic process | 4.65E-06 |
|  | GOTERM_BP_4 | GO:0010604~positive regulation of macromolecule metabolic process | 5.28E-06 |
|  | GOTERM_BP_3 | GO:0031325~positive regulation of cellular metabolic process | 5.43E-06 |
|  | GOTERM_BP_2 | GO:0009893~positive regulation of metabolic process | 5.77E-06 |
|  | GOTERM_BP_5 | GO:0031401~positive regulation of protein modification process | 6.16E-06 |
|  | GOTERM_BP_4 | GO:0031325~positive regulation of cellular metabolic process | 6.16E-06 |
|  | GOTERM_BP_4 | GO:0032268~regulation of cellular protein metabolic process | 6.60E-06 |
|  | GOTERM_BP_3 | GO:0009893~positive regulation of metabolic process | 7.08E-06 |
|  | GOTERM_BP_4 | GO:0051174~regulation of phosphorus metabolic process | 7.38E-06 |
|  | GOTERM_BP_5 | GO:0010604~positive regulation of macromolecule metabolic process | 7.90E-06 |
|  | GOTERM_BP_4 | GO:0009893~positive regulation of metabolic process | 8.04E-06 |
|  | GOTERM_BP_5 | GO:0031325~positive regulation of cellular metabolic process | 9.22E-06 |
|  | GOTERM_BP_5 | GO:0032268~regulation of cellular protein metabolic process | 9.25E-06 |
|  | GOTERM_BP_5 | GO:0051174~regulation of phosphorus metabolic process | 1.03E-05 |
|  | GOTERM_BP_5 | GO:0019220~regulation of phosphate metabolic process | 1.03E-05 |
|  | GOTERM_BP_4 | GO:0051246~regulation of protein metabolic process | 1.32E-05 |
|  | GOTERM_BP_5 | GO:0019221~cytokine-mediated signaling pathway | 1.45E-05 |
|  | GOTERM_BP_5 | GO:0051246~regulation of protein metabolic process | 1.84E-05 |
|  | GOTERM_BP_5 | GO:0010627~regulation of protein kinase cascade | 1.91E-05 |
|  | GOTERM_BP_4 | GO:0009967~positive regulation of signal transduction | 2.84E-05 |
|  | GOTERM_BP_5 | GO:0009967~positive regulation of signal transduction | 3.72E-05 |
|  | GOTERM_BP_5 | GO:0031399~regulation of protein modification process | 3.72E-05 |
|  | GOTERM_BP_3 | GO:0010647~positive regulation of cell communication | 4.00E-05 |
|  | GOTERM_CC_1 | GO:0005576~extracellular region | 4.31E-05 |
|  | GOTERM_BP_4 | GO:0010647~positive regulation of cell communication | 4.36E-05 |
|  | GOTERM_BP_1 | GO:0032502~developmental process | 4.55E-05 |
|  | GOTERM_BP_5 | GO:0010647~positive regulation of cell communication | 5.71E-05 |
|  | GOTERM_BP_3 | GO:0048731~system development | 1.20E-04 |
|  | GOTERM_BP_4 | GO:0009966~regulation of signal transduction | 1.31E-04 |
|  | GOTERM_BP_2 | GO:0048856~anatomical structure development | 1.63E-04 |
|  | GOTERM_BP_5 | GO:0009966~regulation of signal transduction | 1.82E-04 |
|  | GOTERM_BP_3 | GO:0030154~cell differentiation | 1.94E-04 |
|  | GOTERM_MF_4 | GO:0008083~growth factor activity | 1.98E-04 |
|  | GOTERM_BP_2 | GO:0048869~cellular developmental process | 2.01E-04 |
|  | GOTERM_BP_3 | GO:0010646~regulation of cell communication | 2.61E-04 |
|  | GOTERM_BP_4 | GO:0010646~regulation of cell communication | 2.90E-04 |
|  | GOTERM_BP_4 | GO:0007399~nervous system development | 3.62E-04 |
|  | GOTERM_BP_2 | GO:0007275~multicellular organismal development | 3.73E-04 |
|  | GOTERM_BP_3 | GO:0080134~regulation of response to stress | 6.40E-04 |
|  | GOTERM_BP_4 | GO:0080134~regulation of response to stress | 6.81E-04 |
|  | GOTERM_BP_3 | GO:0009891~positive regulation of biosynthetic process | 7.20E-04 |
|  | GOTERM_BP_4 | GO:0031328~positive regulation of cellular biosynthetic process | 7.40E-04 |
|  | GOTERM_BP_4 | GO:0009891~positive regulation of biosynthetic process | 7.82E-04 |
|  | GOTERM_BP_4 | GO:0046883~regulation of hormone secretion | 8.70E-04 |
|  | GOTERM_BP_5 | GO:0031328~positive regulation of cellular biosynthetic process | 9.62E-04 |
|  | GOTERM_BP_5 | GO:0046883~regulation of hormone secretion | 9.98E-04 |
|  | GOTERM_BP_5 | GO:0009891~positive regulation of biosynthetic process | 0.001016 |
|  | GOTERM_BP_4 | GO:0045835~negative regulation of meiosis | 0.001366 |
|  | GOTERM_BP_3 | GO:0043069~negative regulation of programmed cell death | 0.001401 |
|  | GOTERM_BP_3 | GO:0060548~negative regulation of cell death | 0.001413 |
|  | GOTERM_BP_4 | GO:0043066~negative regulation of apoptosis | 0.001433 |
|  | GOTERM_BP_5 | GO:0045835~negative regulation of meiosis | 0.001464 |
|  | GOTERM_BP_4 | GO:0043069~negative regulation of programmed cell death | 0.001492 |
|  | GOTERM_BP_4 | GO:0060548~negative regulation of cell death | 0.001504 |
|  | GOTERM_MF_5 | GO:0005146~leukemia inhibitory factor receptor binding | 0.001606 |
|  | GOTERM_BP_5 | GO:0043066~negative regulation of apoptosis | 0.001748 |
|  | GOTERM_BP_5 | GO:0043069~negative regulation of programmed cell death | 0.00182 |
|  | GOTERM_BP_5 | GO:0060548~negative regulation of cell death | 0.001835 |
|  | GOTERM_BP_2 | GO:0048523~negative regulation of cellular process | 0.001979 |
|  | GOTERM_BP_3 | GO:0048523~negative regulation of cellular process | 0.002323 |
|  | GOTERM_BP_3 | GO:0051049~regulation of transport | 0.002433 |
|  | GOTERM_MF_2 | GO:0005515~protein binding | 0.002527 |
|  | GOTERM_BP_4 | GO:0048523~negative regulation of cellular process | 0.002564 |
|  | GOTERM_BP_4 | GO:0051049~regulation of transport | 0.002589 |
|  | GOTERM_BP_2 | GO:0048583~regulation of response to stimulus | 0.002663 |
|  | GOTERM_BP_3 | GO:0048513~organ development | 0.002862 |
|  | GOTERM_BP_3 | GO:0048583~regulation of response to stimulus | 0.002943 |
|  | GOTERM_BP_2 | GO:0048519~negative regulation of biological process | 0.002948 |
|  | GOTERM_MF_3 | GO:0019955~cytokine binding | 0.002977 |
|  | GOTERM_BP_4 | GO:0048513~organ development | 0.003156 |
|  | GOTERM_BP_3 | GO:0048519~negative regulation of biological process | 0.003455 |
|  | GOTERM_BP_3 | GO:0045595~regulation of cell differentiation | 0.003456 |
|  | GOTERM_BP_4 | GO:0045595~regulation of cell differentiation | 0.003675 |
|  | GOTERM_BP_4 | GO:0031347~regulation of defense response | 0.004007 |
|  | GOTERM_BP_3 | GO:0007165~signal transduction | 0.004069 |
|  | GOTERM_BP_4 | GO:0048644~muscle organ morphogenesis | 0.004093 |
|  | GOTERM_BP_5 | GO:0048644~muscle organ morphogenesis | 0.004386 |
|  | GOTERM_BP_4 | GO:0007165~signal transduction | 0.004569 |
|  | GOTERM_BP_5 | GO:0031347~regulation of defense response | 0.004586 |
|  | GOTERM_BP_1 | GO:0032501~multicellular organismal process | 0.00471 |
|  | GOTERM_BP_3 | GO:0032101~regulation of response to external stimulus | 0.004725 |
|  | GOTERM_BP_4 | GO:0032101~regulation of response to external stimulus | 0.004929 |
|  | GOTERM_BP_3 | GO:0050865~regulation of cell activation | 0.005696 |
|  | GOTERM_BP_2 | GO:0032879~regulation of localization | 0.00574 |
|  | GOTERM_BP_4 | GO:0050865~regulation of cell activation | 0.005941 |
|  | GOTERM_BP_4 | GO:0040020~regulation of meiosis | 0.006134 |
|  | GOTERM_BP_3 | GO:0032879~regulation of localization | 0.006334 |
|  | GOTERM_BP_5 | GO:0048699~generation of neurons | 0.006397 |
|  | GOTERM_BP_4 | GO:0022008~neurogenesis | 0.006456 |
|  | GOTERM_BP_5 | GO:0040020~regulation of meiosis | 0.006573 |
|  | GOTERM_BP_4 | GO:0051445~regulation of meiotic cell cycle | 0.006813 |
|  | GOTERM_BP_4 | GO:0045935~positive regulation of nucleobase, nucleoside, nucleotide and nucleic acid metabolic process | 0.007169 |
|  | GOTERM_BP_5 | GO:0051445~regulation of meiotic cell cycle | 0.0073 |
|  | GOTERM_BP_3 | GO:0048468~cell development | 0.007369 |
|  | GOTERM_BP_3 | GO:0051173~positive regulation of nitrogen compound metabolic process | 0.007369 |
|  | GOTERM_BP_2 | GO:0050793~regulation of developmental process | 0.007585 |
|  | GOTERM_BP_4 | GO:0048468~cell development | 0.007827 |
|  | GOTERM_BP_4 | GO:0051173~positive regulation of nitrogen compound metabolic process | 0.007827 |
|  | GOTERM_BP_5 | GO:0022008~neurogenesis | 0.007829 |
|  | GOTERM_BP_4 | GO:0051046~regulation of secretion | 0.007846 |
|  | GOTERM_BP_4 | GO:0010557~positive regulation of macromolecule biosynthetic process | 0.008169 |
|  | GOTERM_BP_3 | GO:0050793~regulation of developmental process | 0.008363 |
|  | GOTERM_BP_3 | GO:0060255~regulation of macromolecule metabolic process | 0.008429 |
|  | GOTERM_BP_4 | GO:0007517~muscle organ development | 0.008535 |
|  | GOTERM_BP_5 | GO:0045935~positive regulation of nucleobase, nucleoside, nucleotide and nucleic acid metabolic process | 0.008689 |
|  | GOTERM_BP_5 | GO:0051046~regulation of secretion | 0.008966 |
|  | GOTERM_BP_4 | GO:0060255~regulation of macromolecule metabolic process | 0.009439 |
|  | GOTERM_BP_5 | GO:0051173~positive regulation of nitrogen compound metabolic process | 0.009481 |
|  | GOTERM_BP_2 | GO:0048584~positive regulation of response to stimulus | 0.009493 |
|  | GOTERM_BP_3 | GO:0045597~positive regulation of cell differentiation | 0.009585 |
|  | GOTERM_BP_5 | GO:0007517~muscle organ development | 0.009752 |
|  | GOTERM_BP_5 | GO:0010557~positive regulation of macromolecule biosynthetic process | 0.009893 |
|  | GOTERM_BP_4 | GO:0045597~positive regulation of cell differentiation | 0.009993 |
|  | GOTERM_BP_3 | GO:0048584~positive regulation of response to stimulus | 0.010157 |
|  | GOTERM_CC_4 | GO:0019898~extrinsic to membrane | 0.01058 |
|  | GOTERM_BP_4 | GO:0048584~positive regulation of response to stimulus | 0.010588 |
|  | GOTERM_CC_5 | GO:0019898~extrinsic to membrane | 0.010924 |
|  | GOTERM_BP_3 | GO:0060341~regulation of cellular localization | 0.011171 |
|  | GOTERM_BP_5 | GO:0045597~positive regulation of cell differentiation | 0.011412 |
|  | GOTERM_BP_3 | GO:0031323~regulation of cellular metabolic process | 0.011575 |
|  | GOTERM_BP_4 | GO:0060341~regulation of cellular localization | 0.011645 |
|  | GOTERM_BP_2 | GO:0019222~regulation of metabolic process | 0.012136 |
|  | GOTERM_BP_4 | GO:0031323~regulation of cellular metabolic process | 0.012945 |
|  | GOTERM_BP_2 | GO:0051094~positive regulation of developmental process | 0.012997 |
|  | GOTERM_BP_3 | GO:0051094~positive regulation of developmental process | 0.013898 |
|  | GOTERM_BP_3 | GO:0010941~regulation of cell death | 0.014098 |
|  | GOTERM_BP_4 | GO:0051094~positive regulation of developmental process | 0.014484 |
|  | GOTERM_BP_3 | GO:0019222~regulation of metabolic process | 0.01454 |
|  | GOTERM_BP_4 | GO:0043067~regulation of programmed cell death | 0.014805 |
|  | GOTERM_BP_4 | GO:0010941~regulation of cell death | 0.014954 |
|  | GOTERM_BP_3 | GO:0010948~negative regulation of cell cycle process | 0.016594 |
|  | GOTERM_BP_2 | GO:0050794~regulation of cellular process | 0.016606 |
|  | GOTERM_BP_4 | GO:0010948~negative regulation of cell cycle process | 0.016955 |
|  | GOTERM_BP_3 | GO:0046888~negative regulation of hormone secretion | 0.017252 |
|  | GOTERM_BP_5 | GO:0042981~regulation of apoptosis | 0.017386 |
|  | GOTERM_BP_4 | GO:0046888~negative regulation of hormone secretion | 0.017628 |
|  | GOTERM_BP_5 | GO:0043067~regulation of programmed cell death | 0.017857 |
|  | GOTERM_BP_5 | GO:0010948~negative regulation of cell cycle process | 0.018162 |
|  | GOTERM_BP_2 | GO:0051239~regulation of multicellular organismal process | 0.0187 |
|  | GOTERM_BP_5 | GO:0046888~negative regulation of hormone secretion | 0.018883 |
|  | GOTERM_BP_2 | GO:0040008~regulation of growth | 0.019158 |
|  | GOTERM_BP_3 | GO:0040008~regulation of growth | 0.020471 |
|  | GOTERM_BP_3 | GO:0051239~regulation of multicellular organismal process | 0.02055 |
|  | GOTERM_BP_4 | GO:0048666~neuron development | 0.021089 |
|  | GOTERM_BP_3 | GO:0050794~regulation of cellular process | 0.021399 |
|  | GOTERM_BP_1 | GO:0002376~immune system process | 0.0217 |
|  | GOTERM_BP_2 | GO:0050789~regulation of biological process | 0.02235 |
|  | GOTERM_BP_5 | GO:0048666~neuron development | 0.024016 |
|  | GOTERM_BP_2 | GO:0002682~regulation of immune system process | 0.024071 |
|  | GOTERM_BP_5 | GO:0007167~enzyme linked receptor protein signaling pathway | 0.024416 |
|  | GOTERM_BP_3 | GO:0002682~regulation of immune system process | 0.025707 |
|  | GOTERM_BP_4 | GO:0007166~cell surface receptor linked signal transduction | 0.027441 |
|  | GOTERM_BP_5 | GO:0007243~protein kinase cascade | 0.02828 |
|  | GOTERM_BP_3 | GO:0009889~regulation of biosynthetic process | 0.029264 |
|  | GOTERM_BP_1 | GO:0065007~biological regulation | 0.030657 |
|  | GOTERM_BP_4 | GO:0031326~regulation of cellular biosynthetic process | 0.031163 |
|  | GOTERM_BP_4 | GO:0009889~regulation of biosynthetic process | 0.031996 |
|  | GOTERM_BP_4 | GO:0030182~neuron differentiation | 0.034009 |
|  | GOTERM_BP_5 | GO:0007166~cell surface receptor linked signal transduction | 0.03463 |
|  | GOTERM_BP_3 | GO:0051048~negative regulation of secretion | 0.036183 |
|  | GOTERM_BP_4 | GO:0051048~negative regulation of secretion | 0.036964 |
|  | GOTERM_CC_3 | GO:0019898~extrinsic to membrane | 0.039513 |
|  | GOTERM_BP_5 | GO:0051048~negative regulation of secretion | 0.039569 |
|  | GOTERM_BP_3 | GO:0050866~negative regulation of cell activation | 0.040059 |
|  | GOTERM_BP_4 | GO:0051254~positive regulation of RNA metabolic process | 0.040399 |
|  | GOTERM_BP_4 | GO:0050866~negative regulation of cell activation | 0.040922 |
|  | GOTERM_BP_5 | GO:0050770~regulation of axonogenesis | 0.040982 |
|  | GOTERM_MF_4 | GO:0004896~cytokine receptor activity | 0.041282 |
|  | GOTERM_BP_5 | GO:0031326~regulation of cellular biosynthetic process | 0.041317 |
|  | GOTERM_BP_3 | GO:0032103~positive regulation of response to external stimulus | 0.041992 |
|  | GOTERM_BP_4 | GO:0032103~positive regulation of response to external stimulus | 0.042895 |
|  | GOTERM_MF_5 | GO:0004896~cytokine receptor activity | 0.043356 |
|  | GOTERM_BP_5 | GO:0050866~negative regulation of cell activation | 0.043801 |
|  | GOTERM_BP_3 | GO:0080090~regulation of primary metabolic process | 0.044675 |
|  | GOTERM_BP_5 | GO:0045893~positive regulation of transcription, DNA-dependent | 0.045149 |
|  | GOTERM_BP_5 | GO:0051254~positive regulation of RNA metabolic process | 0.04584 |
|  | GOTERM_BP_5 | GO:0032103~positive regulation of response to external stimulus | 0.04591 |
|  | GOTERM_BP_4 | GO:0010975~regulation of neuron projection development | 0.046831 |
|  | GOTERM_BP_4 | GO:0007242~intracellular signaling cascade | 0.046866 |
|  | GOTERM_BP_3 | GO:0031349~positive regulation of defense response | 0.047769 |
|  | GOTERM_BP_4 | GO:0080090~regulation of primary metabolic process | 0.048721 |
|  | GOTERM_BP_4 | GO:0031349~positive regulation of defense response | 0.048794 |
| 5011 | GOTERM_BP_5 | GO:0016055~Wnt receptor signaling pathway | 1.35E-15 |
|  | GOTERM_BP_4 | GO:0007166~cell surface receptor linked signal transduction | 1.21E-06 |
|  | GOTERM_MF_1 | GO:0060089~molecular transducer activity | 1.98E-06 |
|  | GOTERM_BP_5 | GO:0007166~cell surface receptor linked signal transduction | 2.08E-06 |
|  | GOTERM_MF_2 | GO:0004871~signal transducer activity | 2.33E-06 |
|  | GOTERM_BP_2 | GO:0007275~multicellular organismal development | 2.32E-05 |
|  | GOTERM_BP_1 | GO:0032502~developmental process | 4.55E-05 |
|  | GOTERM_BP_4 | GO:0009952~anterior/posterior pattern formation | 9.42E-05 |
|  | GOTERM_BP_5 | GO:0009952~anterior/posterior pattern formation | 1.16E-04 |
|  | GOTERM_BP_2 | GO:0048856~anatomical structure development | 1.63E-04 |
|  | GOTERM_BP_3 | GO:0003002~regionalization | 2.43E-04 |
|  | GOTERM_BP_4 | GO:0003002~regionalization | 2.59E-04 |
|  | GOTERM_BP_2 | GO:0009653~anatomical structure morphogenesis | 4.34E-04 |
|  | GOTERM_BP_1 | GO:0032501~multicellular organismal process | 4.84E-04 |
|  | GOTERM_BP_3 | GO:0009653~anatomical structure morphogenesis | 5.12E-04 |
|  | GOTERM_BP_2 | GO:0007389~pattern specification process | 5.35E-04 |
|  | GOTERM_BP_3 | GO:0007389~pattern specification process | 5.93E-04 |
|  | GOTERM_BP_3 | GO:0016477~cell migration | 6.53E-04 |
|  | GOTERM_BP_4 | GO:0016477~cell migration | 6.96E-04 |
|  | GOTERM_BP_2 | GO:0048870~cell motility | 8.04E-04 |
|  | GOTERM_BP_2 | GO:0051674~localization of cell | 8.04E-04 |
|  | GOTERM_BP_5 | GO:0016477~cell migration | 8.51E-04 |
|  | GOTERM_BP_3 | GO:0048598~embryonic morphogenesis | 8.91E-04 |
|  | GOTERM_BP_3 | GO:0048870~cell motility | 8.91E-04 |
|  | GOTERM_BP_4 | GO:0048870~cell motility | 9.48E-04 |
|  | GOTERM_BP_4 | GO:0048598~embryonic morphogenesis | 9.48E-04 |
|  | GOTERM_BP_4 | GO:0007369~gastrulation | 9.78E-04 |
|  | GOTERM_BP_5 | GO:0007369~gastrulation | 0.001122 |
|  | GOTERM_BP_1 | GO:0016043~cellular component organization | 0.001612 |
|  | GOTERM_MF_4 | GO:0004888~transmembrane receptor activity | 0.001875 |
|  | GOTERM_BP_1 | GO:0040011~locomotion | 0.002097 |
|  | GOTERM_MF_3 | GO:0004872~receptor activity | 0.002128 |
|  | GOTERM_BP_3 | GO:0030154~cell differentiation | 0.00218 |
|  | GOTERM_BP_2 | GO:0048869~cellular developmental process | 0.002242 |
|  | GOTERM_BP_2 | GO:0050789~regulation of biological process | 0.002311 |
|  | GOTERM_MF_3 | GO:0017147~Wnt-protein binding | 0.00245 |
|  | GOTERM_BP_2 | GO:0006928~cell motion | 0.00283 |
|  | GOTERM_BP_3 | GO:0006928~cell motion | 0.003127 |
|  | GOTERM_BP_1 | GO:0065007~biological regulation | 0.003442 |
|  | GOTERM_BP_2 | GO:0009790~embryonic development | 0.004697 |
|  | GOTERM_BP_4 | GO:0042074~cell migration involved in gastrulation | 0.004773 |
|  | GOTERM_BP_5 | GO:0042074~cell migration involved in gastrulation | 0.005115 |
|  | GOTERM_BP_3 | GO:0009790~embryonic development | 0.005186 |
|  | GOTERM_MF_5 | GO:0042813~Wnt receptor activity | 0.005412 |
|  | GOTERM_BP_5 | GO:0048699~generation of neurons | 0.006397 |
|  | GOTERM_BP_4 | GO:0022008~neurogenesis | 0.006456 |
|  | GOTERM_BP_5 | GO:0022008~neurogenesis | 0.007829 |
|  | GOTERM_BP_3 | GO:0006897~endocytosis | 0.008873 |
|  | GOTERM_BP_3 | GO:0010324~membrane invagination | 0.008873 |
|  | GOTERM_BP_4 | GO:0006897~endocytosis | 0.009251 |
|  | GOTERM_BP_5 | GO:0006897~endocytosis | 0.010567 |
|  | GOTERM_CC_5 | GO:0016021~integral to membrane | 0.011734 |
|  | GOTERM_CC_5 | GO:0031224~intrinsic to membrane | 0.014231 |
|  | GOTERM_CC_3 | GO:0005578~proteinaceous extracellular matrix | 0.014276 |
|  | GOTERM_BP_5 | GO:0001702~gastrulation with mouth forming second | 0.015276 |
|  | GOTERM_CC_4 | GO:0005578~proteinaceous extracellular matrix | 0.015435 |
|  | GOTERM_BP_4 | GO:0007420~brain development | 0.015594 |
|  | GOTERM_CC_2 | GO:0031012~extracellular matrix | 0.016448 |
|  | GOTERM_CC_3 | GO:0031012~extracellular matrix | 0.016471 |
|  | GOTERM_BP_2 | GO:0050794~regulation of cellular process | 0.016606 |
|  | GOTERM_BP_4 | GO:0042733~embryonic digit morphogenesis | 0.016955 |
|  | GOTERM_BP_5 | GO:0007420~brain development | 0.017781 |
|  | GOTERM_BP_5 | GO:0042733~embryonic digit morphogenesis | 0.018162 |
|  | GOTERM_BP_3 | GO:0048513~organ development | 0.020366 |
|  | GOTERM_BP_3 | GO:0050794~regulation of cellular process | 0.021399 |
|  | GOTERM_BP_4 | GO:0048513~organ development | 0.02194 |
|  | GOTERM_BP_3 | GO:0000902~cell morphogenesis | 0.022198 |
|  | GOTERM_BP_4 | GO:0000902~cell morphogenesis | 0.02312 |
|  | GOTERM_BP_2 | GO:0016044~membrane organization | 0.023604 |
|  | GOTERM_BP_4 | GO:0001667~ameboidal cell migration | 0.025002 |
|  | GOTERM_BP_2 | GO:0032989~cellular component morphogenesis | 0.025494 |
|  | GOTERM_BP_5 | GO:0000902~cell morphogenesis | 0.026318 |
|  | GOTERM_BP_5 | GO:0001667~ameboidal cell migration | 0.026775 |
|  | GOTERM_BP_3 | GO:0032989~cellular component morphogenesis | 0.027222 |
|  | GOTERM_BP_4 | GO:0032989~cellular component morphogenesis | 0.028344 |
|  | GOTERM_BP_4 | GO:0007417~central nervous system development | 0.032166 |
|  | GOTERM_BP_4 | GO:0007399~nervous system development | 0.032331 |
|  | GOTERM_BP_4 | GO:0030182~neuron differentiation | 0.034009 |
|  | GOTERM_BP_5 | GO:0007417~central nervous system development | 0.036551 |
|  | GOTERM_BP_3 | GO:0042472~inner ear morphogenesis | 0.03683 |
|  | GOTERM_BP_4 | GO:0042472~inner ear morphogenesis | 0.037624 |
|  | GOTERM_CC_2 | GO:0044425~membrane part | 0.038302 |
|  | GOTERM_CC_3 | GO:0044425~membrane part | 0.038462 |
|  | GOTERM_MF_2 | GO:0005515~protein binding | 0.038483 |
|  | GOTERM_BP_5 | GO:0042472~inner ear morphogenesis | 0.040276 |
|  | GOTERM_BP_4 | GO:0042471~ear morphogenesis | 0.044209 |
|  | GOTERM_BP_5 | GO:0030879~mammary gland development | 0.045208 |
|  | GOTERM_BP_5 | GO:0042471~ear morphogenesis | 0.047314 |
|  | GOTERM_CC_4 | GO:0044425~membrane part | 0.048753 |
| 5033 | GOTERM_MF_1 | GO:0030528~transcription regulator activity | 1.27E-13 |
|  | GOTERM_BP_4 | GO:0006350~transcription | 1.05E-12 |
|  | GOTERM_BP_3 | GO:0009059~macromolecule biosynthetic process | 6.78E-11 |
|  | GOTERM_BP_3 | GO:0051171~regulation of nitrogen compound metabolic process | 7.04E-11 |
|  | GOTERM_BP_5 | GO:0045449~regulation of transcription | 7.39E-11 |
|  | GOTERM_BP_4 | GO:0034645~cellular macromolecule biosynthetic process | 8.45E-11 |
|  | GOTERM_BP_4 | GO:0019219~regulation of nucleobase, nucleoside, nucleotide and nucleic acid metabolic process | 8.54E-11 |
|  | GOTERM_BP_4 | GO:0010556~regulation of macromolecule biosynthetic process | 9.30E-11 |
|  | GOTERM_BP_4 | GO:0051171~regulation of nitrogen compound metabolic process | 9.75E-11 |
|  | GOTERM_BP_4 | GO:0010468~regulation of gene expression | 1.07E-10 |
|  | GOTERM_BP_3 | GO:0009889~regulation of biosynthetic process | 1.36E-10 |
|  | GOTERM_BP_3 | GO:0010467~gene expression | 1.61E-10 |
|  | GOTERM_BP_4 | GO:0031326~regulation of cellular biosynthetic process | 1.71E-10 |
|  | GOTERM_BP_4 | GO:0009889~regulation of biosynthetic process | 1.88E-10 |
|  | GOTERM_BP_5 | GO:0019219~regulation of nucleobase, nucleoside, nucleotide and nucleic acid metabolic process | 2.42E-10 |
|  | GOTERM_BP_5 | GO:0010556~regulation of macromolecule biosynthetic process | 2.63E-10 |
|  | GOTERM_BP_5 | GO:0010468~regulation of gene expression | 3.02E-10 |
|  | GOTERM_MF_3 | GO:0003677~DNA binding | 4.07E-10 |
|  | GOTERM_BP_4 | GO:0051254~positive regulation of RNA metabolic process | 4.41E-10 |
|  | GOTERM_BP_5 | GO:0031326~regulation of cellular biosynthetic process | 4.84E-10 |
|  | GOTERM_BP_4 | GO:0051252~regulation of RNA metabolic process | 4.90E-10 |
|  | GOTERM_BP_3 | GO:0060255~regulation of macromolecule metabolic process | 5.60E-10 |
|  | GOTERM_BP_3 | GO:0080090~regulation of primary metabolic process | 6.49E-10 |
|  | GOTERM_BP_5 | GO:0045893~positive regulation of transcription, DNA-dependent | 7.53E-10 |
|  | GOTERM_BP_4 | GO:0060255~regulation of macromolecule metabolic process | 7.76E-10 |
|  | GOTERM_BP_5 | GO:0051254~positive regulation of RNA metabolic process | 8.11E-10 |
|  | GOTERM_BP_5 | GO:0006355~regulation of transcription, DNA-dependent | 8.89E-10 |
|  | GOTERM_BP_4 | GO:0080090~regulation of primary metabolic process | 8.99E-10 |
|  | GOTERM_BP_3 | GO:0006139~nucleobase, nucleoside, nucleotide and nucleic acid metabolic process | 1.10E-09 |
|  | GOTERM_BP_2 | GO:0009058~biosynthetic process | 1.16E-09 |
|  | GOTERM_BP_5 | GO:0051252~regulation of RNA metabolic process | 1.18E-09 |
|  | GOTERM_BP_3 | GO:0044249~cellular biosynthetic process | 1.27E-09 |
|  | GOTERM_BP_3 | GO:0031323~regulation of cellular metabolic process | 1.40E-09 |
|  | GOTERM_BP_2 | GO:0019222~regulation of metabolic process | 1.61E-09 |
|  | GOTERM_BP_4 | GO:0031323~regulation of cellular metabolic process | 1.94E-09 |
|  | GOTERM_BP_4 | GO:0010628~positive regulation of gene expression | 2.34E-09 |
|  | GOTERM_BP_3 | GO:0019222~regulation of metabolic process | 2.73E-09 |
|  | GOTERM_BP_2 | GO:0006807~nitrogen compound metabolic process | 3.05E-09 |
|  | GOTERM_MF_2 | GO:0003700~transcription factor activity | 3.21E-09 |
|  | GOTERM_BP_5 | GO:0045941~positive regulation of transcription | 3.31E-09 |
|  | GOTERM_BP_3 | GO:0034641~cellular nitrogen compound metabolic process | 3.34E-09 |
|  | GOTERM_BP_5 | GO:0010628~positive regulation of gene expression | 4.30E-09 |
|  | GOTERM_BP_4 | GO:0045935~positive regulation of nucleobase, nucleoside, nucleotide and nucleic acid metabolic process | 4.39E-09 |
|  | GOTERM_BP_3 | GO:0051173~positive regulation of nitrogen compound metabolic process | 4.80E-09 |
|  | GOTERM_BP_4 | GO:0051173~positive regulation of nitrogen compound metabolic process | 5.80E-09 |
|  | GOTERM_BP_4 | GO:0010557~positive regulation of macromolecule biosynthetic process | 6.64E-09 |
|  | GOTERM_MF_2 | GO:0003676~nucleic acid binding | 7.34E-09 |
|  | GOTERM_BP_5 | GO:0045935~positive regulation of nucleobase, nucleoside, nucleotide and nucleic acid metabolic process | 8.05E-09 |
|  | GOTERM_BP_3 | GO:0009891~positive regulation of biosynthetic process | 9.36E-09 |
|  | GOTERM_BP_4 | GO:0031328~positive regulation of cellular biosynthetic process | 9.96E-09 |
|  | GOTERM_BP_5 | GO:0051173~positive regulation of nitrogen compound metabolic process | 1.06E-08 |
|  | GOTERM_BP_4 | GO:0009891~positive regulation of biosynthetic process | 1.13E-08 |
|  | GOTERM_BP_5 | GO:0010557~positive regulation of macromolecule biosynthetic process | 1.21E-08 |
|  | GOTERM_BP_5 | GO:0031328~positive regulation of cellular biosynthetic process | 1.82E-08 |
|  | GOTERM_BP_5 | GO:0009891~positive regulation of biosynthetic process | 2.07E-08 |
|  | GOTERM_MF_4 | GO:0003700~transcription factor activity | 2.97E-08 |
|  | GOTERM_BP_3 | GO:0010604~positive regulation of macromolecule metabolic process | 5.82E-08 |
|  | GOTERM_BP_4 | GO:0010604~positive regulation of macromolecule metabolic process | 7.02E-08 |
|  | GOTERM_BP_3 | GO:0031325~positive regulation of cellular metabolic process | 7.32E-08 |
|  | GOTERM_BP_2 | GO:0009893~positive regulation of metabolic process | 8.02E-08 |
|  | GOTERM_MF_2 | GO:0003702~RNA polymerase II transcription factor activity | 8.26E-08 |
|  | GOTERM_BP_4 | GO:0031325~positive regulation of cellular metabolic process | 8.83E-08 |
|  | GOTERM_BP_3 | GO:0009893~positive regulation of metabolic process | 1.09E-07 |
|  | GOTERM_CC_4 | GO:0005634~nucleus | 1.21E-07 |
|  | GOTERM_BP_5 | GO:0010604~positive regulation of macromolecule metabolic process | 1.28E-07 |
|  | GOTERM_BP_4 | GO:0009893~positive regulation of metabolic process | 1.31E-07 |
|  | GOTERM_CC_5 | GO:0005634~nucleus | 1.55E-07 |
|  | GOTERM_BP_5 | GO:0031325~positive regulation of cellular metabolic process | 1.60E-07 |
|  | GOTERM_MF_4 | GO:0043565~sequence-specific DNA binding | 2.22E-07 |
|  | GOTERM_BP_3 | GO:0044260~cellular macromolecule metabolic process | 6.53E-07 |
|  | GOTERM_MF_3 | GO:0008134~transcription factor binding | 6.81E-07 |
|  | GOTERM_MF_2 | GO:0003712~transcription cofactor activity | 8.59E-07 |
|  | GOTERM_BP_2 | GO:0043170~macromolecule metabolic process | 1.51E-06 |
|  | GOTERM_MF_2 | GO:0016563~transcription activator activity | 1.75E-06 |
|  | GOTERM_MF_3 | GO:0003713~transcription coactivator activity | 2.97E-06 |
|  | GOTERM_MF_5 | GO:0003713~transcription coactivator activity | 3.06E-06 |
|  | GOTERM_MF_4 | GO:0003712~transcription cofactor activity | 3.26E-06 |
|  | GOTERM_BP_2 | GO:0044237~cellular metabolic process | 1.44E-05 |
|  | GOTERM_BP_2 | GO:0050794~regulation of cellular process | 2.17E-05 |
|  | GOTERM_BP_2 | GO:0044238~primary metabolic process | 2.72E-05 |
|  | GOTERM_BP_2 | GO:0048522~positive regulation of cellular process | 2.90E-05 |
|  | GOTERM_BP_3 | GO:0050794~regulation of cellular process | 3.68E-05 |
|  | GOTERM_BP_3 | GO:0048522~positive regulation of cellular process | 3.87E-05 |
|  | GOTERM_BP_2 | GO:0050789~regulation of biological process | 4.03E-05 |
|  | GOTERM_BP_4 | GO:0048522~positive regulation of cellular process | 4.62E-05 |
|  | GOTERM_CC_3 | GO:0043231~intracellular membrane-bounded organelle | 5.83E-05 |
|  | GOTERM_CC_2 | GO:0043227~membrane-bounded organelle | 5.85E-05 |
|  | GOTERM_BP_2 | GO:0048518~positive regulation of biological process | 6.35E-05 |
|  | GOTERM_BP_1 | GO:0065007~biological regulation | 7.83E-05 |
|  | GOTERM_BP_3 | GO:0048518~positive regulation of biological process | 8.44E-05 |
|  | GOTERM_BP_4 | GO:0007399~nervous system development | 9.15E-05 |
|  | GOTERM_BP_5 | GO:0030522~intracellular receptor-mediated signaling pathway | 9.42E-05 |
|  | GOTERM_CC_4 | GO:0043231~intracellular membrane-bounded organelle | 1.08E-04 |
|  | GOTERM_BP_1 | GO:0008152~metabolic process | 1.08E-04 |
|  | GOTERM_CC_5 | GO:0043231~intracellular membrane-bounded organelle | 1.39E-04 |
|  | GOTERM_CC_1 | GO:0043226~organelle | 1.90E-04 |
|  | GOTERM_CC_2 | GO:0043229~intracellular organelle | 3.37E-04 |
|  | GOTERM_CC_3 | GO:0043229~intracellular organelle | 3.40E-04 |
|  | GOTERM_BP_4 | GO:0051253~negative regulation of RNA metabolic process | 6.02E-04 |
|  | GOTERM_CC_4 | GO:0043229~intracellular organelle | 6.30E-04 |
|  | GOTERM_BP_5 | GO:0045892~negative regulation of transcription, DNA-dependent | 7.33E-04 |
|  | GOTERM_BP_5 | GO:0051253~negative regulation of RNA metabolic process | 7.80E-04 |
|  | GOTERM_CC_5 | GO:0043229~intracellular organelle | 8.08E-04 |
|  | GOTERM_BP_3 | GO:0010605~negative regulation of macromolecule metabolic process | 9.02E-04 |
|  | GOTERM_BP_4 | GO:0010605~negative regulation of macromolecule metabolic process | 9.95E-04 |
|  | GOTERM_BP_2 | GO:0009892~negative regulation of metabolic process | 0.001014 |
|  | GOTERM_MF_3 | GO:0003704~specific RNA polymerase II transcription factor activity | 0.001083 |
|  | GOTERM_BP_3 | GO:0009892~negative regulation of metabolic process | 0.001188 |
|  | GOTERM_BP_4 | GO:0009892~negative regulation of metabolic process | 0.00131 |
|  | GOTERM_BP_5 | GO:0010605~negative regulation of macromolecule metabolic process | 0.00136 |
|  | GOTERM_BP_3 | GO:0048513~organ development | 0.001474 |
|  | GOTERM_MF_5 | GO:0003707~steroid hormone receptor activity | 0.001517 |
|  | GOTERM_BP_4 | GO:0007517~muscle organ development | 0.001599 |
|  | GOTERM_BP_4 | GO:0048513~organ development | 0.001679 |
|  | GOTERM_BP_5 | GO:0006916~anti-apoptosis | 0.001819 |
|  | GOTERM_BP_5 | GO:0016481~negative regulation of transcription | 0.001887 |
|  | GOTERM_BP_5 | GO:0007517~muscle organ development | 0.001948 |
|  | GOTERM_MF_5 | GO:0010843~promoter binding | 0.002048 |
|  | GOTERM_BP_4 | GO:0010629~negative regulation of gene expression | 0.002064 |
|  | GOTERM_BP_3 | GO:0051172~negative regulation of nitrogen compound metabolic process | 0.002122 |
|  | GOTERM_BP_4 | GO:0045934~negative regulation of nucleobase, nucleoside, nucleotide and nucleic acid metabolic process | 0.002186 |
|  | GOTERM_BP_4 | GO:0051172~negative regulation of nitrogen compound metabolic process | 0.002298 |
|  | GOTERM_BP_4 | GO:0001764~neuron migration | 0.002342 |
|  | GOTERM_MF_4 | GO:0004879~ligand-dependent nuclear receptor activity | 0.002404 |
|  | GOTERM_BP_5 | GO:0010629~negative regulation of gene expression | 0.002657 |
|  | GOTERM_BP_5 | GO:0001764~neuron migration | 0.002682 |
|  | GOTERM_BP_4 | GO:0010558~negative regulation of macromolecule biosynthetic process | 0.002783 |
|  | GOTERM_BP_5 | GO:0045934~negative regulation of nucleobase, nucleoside, nucleotide and nucleic acid metabolic process | 0.002814 |
|  | GOTERM_BP_5 | GO:0051172~negative regulation of nitrogen compound metabolic process | 0.002956 |
|  | GOTERM_BP_3 | GO:0009890~negative regulation of biosynthetic process | 0.003045 |
|  | GOTERM_BP_4 | GO:0031327~negative regulation of cellular biosynthetic process | 0.003051 |
|  | GOTERM_MF_3 | GO:0047485~protein N-terminus binding | 0.003175 |
|  | GOTERM_BP_4 | GO:0009890~negative regulation of biosynthetic process | 0.003294 |
|  | GOTERM_BP_5 | GO:0010558~negative regulation of macromolecule biosynthetic process | 0.003576 |
|  | GOTERM_MF_5 | GO:0035257~nuclear hormone receptor binding | 0.003705 |
|  | GOTERM_BP_4 | GO:0007420~brain development | 0.003909 |
|  | GOTERM_BP_5 | GO:0031327~negative regulation of cellular biosynthetic process | 0.003917 |
|  | GOTERM_CC_2 | GO:0044424~intracellular part | 0.004219 |
|  | GOTERM_BP_5 | GO:0009890~negative regulation of biosynthetic process | 0.004227 |
|  | GOTERM_CC_3 | GO:0044424~intracellular part | 0.004265 |
|  | GOTERM_BP_5 | GO:0007420~brain development | 0.004745 |
|  | GOTERM_BP_2 | GO:0048523~negative regulation of cellular process | 0.005434 |
|  | GOTERM_MF_4 | GO:0051427~hormone receptor binding | 0.005445 |
|  | GOTERM_BP_3 | GO:0030154~cell differentiation | 0.006041 |
|  | GOTERM_BP_2 | GO:0048869~cellular developmental process | 0.006229 |
|  | GOTERM_BP_3 | GO:0048523~negative regulation of cellular process | 0.006475 |
|  | GOTERM_BP_3 | GO:0043069~negative regulation of programmed cell death | 0.00674 |
|  | GOTERM_BP_3 | GO:0060548~negative regulation of cell death | 0.006792 |
|  | GOTERM_BP_5 | GO:0051090~regulation of transcription factor activity | 0.0068 |
|  | GOTERM_BP_4 | GO:0043066~negative regulation of apoptosis | 0.006884 |
|  | GOTERM_BP_3 | GO:0031324~negative regulation of cellular metabolic process | 0.006897 |
|  | GOTERM_CC_2 | GO:0005622~intracellular | 0.007062 |
|  | GOTERM_CC_3 | GO:0005622~intracellular | 0.007139 |
|  | GOTERM_BP_4 | GO:0043069~negative regulation of programmed cell death | 0.007156 |
|  | GOTERM_BP_4 | GO:0048523~negative regulation of cellular process | 0.00721 |
|  | GOTERM_BP_4 | GO:0060548~negative regulation of cell death | 0.007211 |
|  | GOTERM_BP_4 | GO:0031324~negative regulation of cellular metabolic process | 0.007444 |
|  | GOTERM_CC_4 | GO:0044424~intracellular part | 0.007899 |
|  | GOTERM_BP_4 | GO:0051101~regulation of DNA binding | 0.008124 |
|  | GOTERM_BP_3 | GO:0048731~system development | 0.008132 |
|  | GOTERM_BP_5 | GO:0043066~negative regulation of apoptosis | 0.008329 |
|  | GOTERM_BP_2 | GO:0048519~negative regulation of biological process | 0.008391 |
|  | GOTERM_BP_5 | GO:0043069~negative regulation of programmed cell death | 0.008656 |
|  | GOTERM_BP_5 | GO:0060548~negative regulation of cell death | 0.008723 |
|  | GOTERM_BP_1 | GO:0032502~developmental process | 0.008747 |
|  | GOTERM_BP_5 | GO:0031324~negative regulation of cellular metabolic process | 0.009482 |
|  | GOTERM_BP_3 | GO:0048519~negative regulation of biological process | 0.009964 |
|  | GOTERM_BP_2 | GO:0048856~anatomical structure development | 0.010512 |
|  | GOTERM_MF_2 | GO:0005515~protein binding | 0.010625 |
|  | GOTERM_BP_4 | GO:0007417~central nervous system development | 0.011361 |
|  | GOTERM_BP_3 | GO:0051098~regulation of binding | 0.012227 |
|  | GOTERM_BP_1 | GO:0009987~cellular process | 0.013382 |
|  | GOTERM_BP_5 | GO:0007417~central nervous system development | 0.013699 |
|  | GOTERM_BP_5 | GO:0045885~positive regulation of survival gene product expression | 0.014552 |
|  | GOTERM_BP_2 | GO:0007275~multicellular organismal development | 0.020781 |
|  | GOTERM_BP_3 | GO:0007165~signal transduction | 0.023892 |
|  | GOTERM_BP_5 | GO:0045884~regulation of survival gene product expression | 0.025336 |
|  | GOTERM_BP_4 | GO:0007165~signal transduction | 0.026764 |
|  | GOTERM_BP_3 | GO:0012501~programmed cell death | 0.028145 |
|  | GOTERM_BP_5 | GO:0048699~generation of neurons | 0.028287 |
|  | GOTERM_BP_4 | GO:0022008~neurogenesis | 0.028525 |
|  | GOTERM_BP_4 | GO:0006915~apoptosis | 0.028648 |
|  | GOTERM_BP_5 | GO:0022008~neurogenesis | 0.034095 |
|  | GOTERM_BP_3 | GO:0016477~cell migration | 0.036883 |
|  | GOTERM_MF_4 | GO:0030545~receptor regulator activity | 0.037571 |
|  | GOTERM_BP_4 | GO:0016477~cell migration | 0.038371 |
|  | GOTERM_BP_1 | GO:0016265~death | 0.038986 |
|  | GOTERM_BP_2 | GO:0008219~cell death | 0.039049 |
|  | GOTERM_MF_2 | GO:0016564~transcription repressor activity | 0.039608 |
|  | GOTERM_BP_2 | GO:0048870~cell motility | 0.041997 |
|  | GOTERM_BP_2 | GO:0051674~localization of cell | 0.041997 |
|  | GOTERM_BP_5 | GO:0016477~cell migration | 0.043513 |
|  | GOTERM_BP_3 | GO:0048870~cell motility | 0.044755 |
|  | GOTERM_MF_4 | GO:0030374~ligand-dependent nuclear receptor transcription coactivator activity | 0.044925 |
|  | GOTERM_BP_4 | GO:0048870~cell motility | 0.046541 |
| 5115 | GOTERM_MF_1 | GO:0030528~transcription regulator activity | 1.70E-07 |
|  | GOTERM_MF_2 | GO:0003676~nucleic acid binding | 1.10E-05 |
|  | GOTERM_BP_2 | GO:0009058~biosynthetic process | 1.28E-05 |
|  | GOTERM_MF_3 | GO:0003677~DNA binding | 2.05E-05 |
|  | GOTERM_BP_3 | GO:0010467~gene expression | 4.54E-05 |
|  | GOTERM_BP_2 | GO:0044237~cellular metabolic process | 5.76E-05 |
|  | GOTERM_BP_3 | GO:0044249~cellular biosynthetic process | 6.76E-05 |
|  | GOTERM_BP_4 | GO:0006350~transcription | 7.56E-05 |
|  | GOTERM_BP_4 | GO:0010468~regulation of gene expression | 1.42E-04 |
|  | GOTERM_BP_5 | GO:0010468~regulation of gene expression | 1.99E-04 |
|  | GOTERM_BP_3 | GO:0006139~nucleobase, nucleoside, nucleotide and nucleic acid metabolic process | 2.51E-04 |
|  | GOTERM_BP_5 | GO:0045449~regulation of transcription | 2.70E-04 |
|  | GOTERM_BP_3 | GO:0051171~regulation of nitrogen compound metabolic process | 4.18E-04 |
|  | GOTERM_BP_4 | GO:0019219~regulation of nucleobase, nucleoside, nucleotide and nucleic acid metabolic process | 4.84E-04 |
|  | GOTERM_BP_4 | GO:0010556~regulation of macromolecule biosynthetic process | 5.16E-04 |
|  | GOTERM_BP_4 | GO:0051171~regulation of nitrogen compound metabolic process | 5.35E-04 |
|  | GOTERM_BP_3 | GO:0060255~regulation of macromolecule metabolic process | 5.50E-04 |
|  | GOTERM_BP_2 | GO:0006807~nitrogen compound metabolic process | 5.97E-04 |
|  | GOTERM_BP_3 | GO:0034641~cellular nitrogen compound metabolic process | 6.45E-04 |
|  | GOTERM_BP_5 | GO:0019219~regulation of nucleobase, nucleoside, nucleotide and nucleic acid metabolic process | 6.65E-04 |
|  | GOTERM_BP_3 | GO:0009889~regulation of biosynthetic process | 6.86E-04 |
|  | GOTERM_BP_5 | GO:0010556~regulation of macromolecule biosynthetic process | 7.09E-04 |
|  | GOTERM_BP_4 | GO:0060255~regulation of macromolecule metabolic process | 7.13E-04 |
|  | GOTERM_BP_4 | GO:0031326~regulation of cellular biosynthetic process | 8.14E-04 |
|  | GOTERM_BP_4 | GO:0009889~regulation of biosynthetic process | 8.74E-04 |
|  | GOTERM_BP_1 | GO:0008152~metabolic process | 8.78E-04 |
|  | GOTERM_BP_5 | GO:0031326~regulation of cellular biosynthetic process | 0.001114 |
|  | GOTERM_BP_2 | GO:0019222~regulation of metabolic process | 0.00126 |
|  | GOTERM_BP_3 | GO:0009059~macromolecule biosynthetic process | 0.001498 |
|  | GOTERM_CC_5 | GO:0005634~nucleus | 0.001598 |
|  | GOTERM_BP_4 | GO:0034645~cellular macromolecule biosynthetic process | 0.001738 |
|  | GOTERM_BP_3 | GO:0019222~regulation of metabolic process | 0.001888 |
|  | GOTERM_BP_3 | GO:0080090~regulation of primary metabolic process | 0.002143 |
|  | GOTERM_CC_4 | GO:0005634~nucleus | 0.002296 |
|  | GOTERM_BP_2 | GO:0044238~primary metabolic process | 0.002297 |
|  | GOTERM_CC_3 | GO:0043231~intracellular membrane-bounded organelle | 0.002447 |
|  | GOTERM_CC_2 | GO:0043227~membrane-bounded organelle | 0.002454 |
|  | GOTERM_BP_4 | GO:0080090~regulation of primary metabolic process | 0.002698 |
|  | GOTERM_CC_5 | GO:0043231~intracellular membrane-bounded organelle | 0.002796 |
|  | GOTERM_BP_3 | GO:0031323~regulation of cellular metabolic process | 0.003678 |
|  | GOTERM_CC_5 | GO:0044451~nucleoplasm part | 0.003902 |
|  | GOTERM_BP_2 | GO:0043170~macromolecule metabolic process | 0.003915 |
|  | GOTERM_CC_4 | GO:0044451~nucleoplasm part | 0.004258 |
|  | GOTERM_CC_3 | GO:0070013~intracellular organelle lumen | 0.004384 |
|  | GOTERM_BP_4 | GO:0031323~regulation of cellular metabolic process | 0.004601 |
|  | GOTERM_CC_4 | GO:0043231~intracellular membrane-bounded organelle | 0.004896 |
|  | GOTERM_CC_5 | GO:0070013~intracellular organelle lumen | 0.004975 |
|  | GOTERM_CC_5 | GO:0031981~nuclear lumen | 0.00506 |
|  | GOTERM_CC_2 | GO:0043233~organelle lumen | 0.005085 |
|  | GOTERM_CC_3 | GO:0043233~organelle lumen | 0.005109 |
|  | GOTERM_BP_3 | GO:0044260~cellular macromolecule metabolic process | 0.005475 |
|  | GOTERM_CC_1 | GO:0031974~membrane-enclosed lumen | 0.005715 |
|  | GOTERM_CC_4 | GO:0070013~intracellular organelle lumen | 0.00577 |
|  | GOTERM_CC_4 | GO:0031981~nuclear lumen | 0.005776 |
|  | GOTERM_CC_5 | GO:0005654~nucleoplasm | 0.006059 |
|  | GOTERM_CC_4 | GO:0005654~nucleoplasm | 0.0067 |
|  | GOTERM_CC_3 | GO:0005667~transcription factor complex | 0.007183 |
|  | GOTERM_CC_5 | GO:0005667~transcription factor complex | 0.007617 |
|  | GOTERM_BP_4 | GO:0010628~positive regulation of gene expression | 0.009358 |
|  | GOTERM_BP_5 | GO:0045941~positive regulation of transcription | 0.009507 |
|  | GOTERM_BP_1 | GO:0009987~cellular process | 0.010599 |
|  | GOTERM_BP_5 | GO:0010628~positive regulation of gene expression | 0.01073 |
|  | GOTERM_BP_4 | GO:0045935~positive regulation of nucleobase, nucleoside, nucleotide and nucleic acid metabolic process | 0.012501 |
|  | GOTERM_BP_3 | GO:0051173~positive regulation of nitrogen compound metabolic process | 0.013007 |
|  | GOTERM_BP_4 | GO:0051173~positive regulation of nitrogen compound metabolic process | 0.014184 |
|  | GOTERM_BP_5 | GO:0045935~positive regulation of nucleobase, nucleoside, nucleotide and nucleic acid metabolic process | 0.014304 |
|  | GOTERM_BP_4 | GO:0010557~positive regulation of macromolecule biosynthetic process | 0.015081 |
|  | GOTERM_BP_5 | GO:0051173~positive regulation of nitrogen compound metabolic process | 0.016215 |
|  | GOTERM_BP_4 | GO:0051252~regulation of RNA metabolic process | 0.016437 |
|  | GOTERM_CC_2 | GO:0043229~intracellular organelle | 0.016576 |
|  | GOTERM_CC_3 | GO:0043229~intracellular organelle | 0.016763 |
|  | GOTERM_BP_5 | GO:0010557~positive regulation of macromolecule biosynthetic process | 0.017232 |
|  | GOTERM_CC_3 | GO:0044428~nuclear part | 0.017273 |
|  | GOTERM_BP_5 | GO:0006355~regulation of transcription, DNA-dependent | 0.017492 |
|  | GOTERM_BP_3 | GO:0009891~positive regulation of biosynthetic process | 0.017604 |
|  | GOTERM_BP_4 | GO:0031328~positive regulation of cellular biosynthetic process | 0.018106 |
|  | GOTERM_CC_1 | GO:0043226~organelle | 0.018175 |
|  | GOTERM_BP_4 | GO:0009891~positive regulation of biosynthetic process | 0.019163 |
|  | GOTERM_CC_5 | GO:0044428~nuclear part | 0.019294 |
|  | GOTERM_CC_5 | GO:0043229~intracellular organelle | 0.019944 |
|  | GOTERM_BP_5 | GO:0051252~regulation of RNA metabolic process | 0.02005 |
|  | GOTERM_BP_5 | GO:0031328~positive regulation of cellular biosynthetic process | 0.020656 |
|  | GOTERM_CC_4 | GO:0044428~nuclear part | 0.021708 |
|  | GOTERM_BP_5 | GO:0009891~positive regulation of biosynthetic process | 0.021851 |
|  | GOTERM_BP_4 | GO:0051254~positive regulation of RNA metabolic process | 0.022675 |
|  | GOTERM_BP_5 | GO:0045893~positive regulation of transcription, DNA-dependent | 0.024655 |
|  | GOTERM_BP_5 | GO:0051254~positive regulation of RNA metabolic process | 0.02533 |
|  | GOTERM_CC_4 | GO:0043229~intracellular organelle | 0.031265 |
|  | GOTERM_MF_4 | GO:0043565~sequence-specific DNA binding | 0.032686 |
|  | GOTERM_BP_3 | GO:0060042~retina morphogenesis in camera-type eye | 0.032925 |
|  | GOTERM_BP_4 | GO:0060042~retina morphogenesis in camera-type eye | 0.033637 |
|  | GOTERM_BP_3 | GO:0008610~lipid biosynthetic process | 0.034443 |
|  | GOTERM_BP_4 | GO:0008610~lipid biosynthetic process | 0.036379 |
|  | GOTERM_BP_2 | GO:0050878~regulation of body fluid levels | 0.036713 |
|  | GOTERM_BP_3 | GO:0045165~cell fate commitment | 0.038129 |
|  | GOTERM_BP_3 | GO:0010604~positive regulation of macromolecule metabolic process | 0.039079 |
|  | GOTERM_BP_3 | GO:0050878~regulation of body fluid levels | 0.039131 |
|  | GOTERM_BP_4 | GO:0045165~cell fate commitment | 0.039657 |
|  | GOTERM_MF_2 | GO:0016563~transcription activator activity | 0.041259 |
|  | GOTERM_BP_4 | GO:0010604~positive regulation of macromolecule metabolic process | 0.042305 |
|  | GOTERM_BP_3 | GO:0031325~positive regulation of cellular metabolic process | 0.043052 |
|  | GOTERM_BP_2 | GO:0009893~positive regulation of metabolic process | 0.044709 |
|  | GOTERM_BP_4 | GO:0031325~positive regulation of cellular metabolic process | 0.046569 |
|  | GOTERM_BP_1 | GO:0065007~biological regulation | 0.047294 |
|  | GOTERM_BP_5 | GO:0010604~positive regulation of macromolecule metabolic process | 0.047854 |
| 5219 | GOTERM_CC_1 | GO:0005576~extracellular region | 2.92E-18 |
|  | GOTERM_CC_4 | GO:0005578~proteinaceous extracellular matrix | 1.09E-11 |
|  | GOTERM_CC_2 | GO:0044421~extracellular region part | 1.46E-11 |
|  | GOTERM_CC_3 | GO:0005578~proteinaceous extracellular matrix | 2.13E-11 |
|  | GOTERM_CC_2 | GO:0031012~extracellular matrix | 4.44E-11 |
|  | GOTERM_CC_3 | GO:0031012~extracellular matrix | 4.47E-11 |
|  | GOTERM_CC_1 | GO:0044421~extracellular region part | 1.40E-10 |
|  | GOTERM_CC_5 | GO:0005604~basement membrane | 3.62E-10 |
|  | GOTERM_CC_4 | GO:0005604~basement membrane | 1.30E-09 |
|  | GOTERM_CC_3 | GO:0005604~basement membrane | 1.82E-09 |
|  | GOTERM_MF_4 | GO:0004866~endopeptidase inhibitor activity | 3.74E-09 |
|  | GOTERM_MF_3 | GO:0030414~peptidase inhibitor activity | 1.07E-08 |
|  | GOTERM_CC_4 | GO:0044420~extracellular matrix part | 1.52E-08 |
|  | GOTERM_CC_2 | GO:0044420~extracellular matrix part | 2.12E-08 |
|  | GOTERM_CC_3 | GO:0044420~extracellular matrix part | 2.13E-08 |
|  | GOTERM_BP_5 | GO:0009888~tissue development | 2.13E-07 |
|  | GOTERM_MF_2 | GO:0004857~enzyme inhibitor activity | 4.00E-07 |
|  | GOTERM_MF_4 | GO:0008191~metalloendopeptidase inhibitor activity | 5.82E-07 |
|  | GOTERM_MF_2 | GO:0010576~metalloenzyme regulator activity | 6.41E-07 |
|  | GOTERM_MF_3 | GO:0048551~metalloenzyme inhibitor activity | 7.39E-07 |
|  | GOTERM_MF_5 | GO:0008191~metalloendopeptidase inhibitor activity | 8.24E-07 |
|  | GOTERM_BP_4 | GO:0009888~tissue development | 8.70E-07 |
|  | GOTERM_BP_3 | GO:0009888~tissue development | 1.66E-06 |
|  | GOTERM_BP_3 | GO:0048731~system development | 2.04E-06 |
|  | GOTERM_BP_2 | GO:0048856~anatomical structure development | 7.33E-06 |
|  | GOTERM_CC_5 | GO:0031093~platelet alpha granule lumen | 1.69E-05 |
|  | GOTERM_CC_5 | GO:0060205~cytoplasmic membrane-bounded vesicle lumen | 2.09E-05 |
|  | GOTERM_BP_1 | GO:0022610~biological adhesion | 2.58E-05 |
|  | GOTERM_BP_2 | GO:0007155~cell adhesion | 2.70E-05 |
|  | GOTERM_BP_2 | GO:0007275~multicellular organismal development | 3.58E-05 |
|  | GOTERM_CC_4 | GO:0060205~cytoplasmic membrane-bounded vesicle lumen | 3.73E-05 |
|  | GOTERM_CC_4 | GO:0031983~vesicle lumen | 4.26E-05 |
|  | GOTERM_CC_3 | GO:0031983~vesicle lumen | 4.94E-05 |
|  | GOTERM_MF_4 | GO:0005539~glycosaminoglycan binding | 9.80E-05 |
|  | GOTERM_BP_1 | GO:0032502~developmental process | 1.04E-04 |
|  | GOTERM_BP_5 | GO:0008544~epidermis development | 1.58E-04 |
|  | GOTERM_MF_2 | GO:0001871~pattern binding | 1.63E-04 |
|  | GOTERM_MF_3 | GO:0030247~polysaccharide binding | 1.94E-04 |
|  | GOTERM_BP_5 | GO:0007398~ectoderm development | 2.13E-04 |
|  | GOTERM_MF_5 | GO:0004252~serine-type endopeptidase activity | 2.20E-04 |
|  | GOTERM_MF_4 | GO:0008236~serine-type peptidase activity | 2.47E-04 |
|  | GOTERM_BP_4 | GO:0008544~epidermis development | 2.65E-04 |
|  | GOTERM_BP_4 | GO:0048513~organ development | 3.47E-04 |
|  | GOTERM_MF_3 | GO:0017171~serine hydrolase activity | 3.53E-04 |
|  | GOTERM_BP_4 | GO:0007398~ectoderm development | 3.57E-04 |
|  | GOTERM_MF_5 | GO:0008236~serine-type peptidase activity | 3.82E-04 |
|  | GOTERM_MF_2 | GO:0030246~carbohydrate binding | 3.95E-04 |
|  | GOTERM_BP_3 | GO:0048513~organ development | 6.48E-04 |
|  | GOTERM_MF_1 | GO:0030234~enzyme regulator activity | 6.66E-04 |
|  | GOTERM_MF_5 | GO:0004175~endopeptidase activity | 7.16E-04 |
|  | GOTERM_MF_5 | GO:0004867~serine-type endopeptidase inhibitor activity | 7.76E-04 |
|  | GOTERM_BP_3 | GO:0051239~regulation of multicellular organismal process | 0.001224 |
|  | GOTERM_BP_2 | GO:0051239~regulation of multicellular organismal process | 0.001283 |
|  | GOTERM_CC_5 | GO:0030141~secretory granule | 0.001358 |
|  | GOTERM_CC_5 | GO:0044433~cytoplasmic vesicle part | 0.001515 |
|  | GOTERM_MF_4 | GO:0070011~peptidase activity, acting on L-amino acid peptides | 0.002365 |
|  | GOTERM_BP_4 | GO:0032101~regulation of response to external stimulus | 0.002559 |
|  | GOTERM_CC_4 | GO:0044433~cytoplasmic vesicle part | 0.002627 |
|  | GOTERM_CC_3 | GO:0044433~cytoplasmic vesicle part | 0.003019 |
|  | GOTERM_BP_3 | GO:0032101~regulation of response to external stimulus | 0.003072 |
|  | GOTERM_BP_1 | GO:0032501~multicellular organismal process | 0.003557 |
|  | GOTERM_CC_2 | GO:0005615~extracellular space | 0.003713 |
|  | GOTERM_CC_3 | GO:0005615~extracellular space | 0.003724 |
|  | GOTERM_MF_3 | GO:0008233~peptidase activity | 0.004132 |
|  | GOTERM_BP_3 | GO:0050793~regulation of developmental process | 0.007141 |
|  | GOTERM_BP_2 | GO:0050793~regulation of developmental process | 0.007336 |
|  | GOTERM_CC_3 | GO:0031988~membrane-bounded vesicle | 0.0111 |
|  | GOTERM_CC_5 | GO:0043256~laminin complex | 0.011176 |
|  | GOTERM_BP_4 | GO:0080134~regulation of response to stress | 0.011584 |
|  | GOTERM_CC_4 | GO:0043256~laminin complex | 0.013422 |
|  | GOTERM_BP_3 | GO:0080134~regulation of response to stress | 0.013773 |
|  | GOTERM_CC_3 | GO:0043256~laminin complex | 0.014049 |
|  | GOTERM_BP_3 | GO:0009611~response to wounding | 0.015565 |
|  | GOTERM_MF_5 | GO:0008201~heparin binding | 0.017832 |
|  | GOTERM_BP_5 | GO:0042730~fibrinolysis | 0.0184 |
|  | GOTERM_CC_2 | GO:0031982~vesicle | 0.01929 |
|  | GOTERM_CC_5 | GO:0005605~basal lamina | 0.021011 |
|  | GOTERM_BP_4 | GO:0030155~regulation of cell adhesion | 0.023548 |
|  | GOTERM_CC_4 | GO:0005605~basal lamina | 0.025208 |
|  | GOTERM_CC_3 | GO:0005605~basal lamina | 0.026377 |
|  | GOTERM_BP_3 | GO:0030155~regulation of cell adhesion | 0.026467 |
|  | GOTERM_MF_1 | GO:0005198~structural molecule activity | 0.02809 |
|  | GOTERM_CC_5 | GO:0016023~cytoplasmic membrane-bounded vesicle | 0.02935 |
|  | GOTERM_BP_4 | GO:0051241~negative regulation of multicellular organismal process | 0.03284 |
|  | GOTERM_MF_2 | GO:0005515~protein binding | 0.033695 |
|  | GOTERM_BP_5 | GO:0030195~negative regulation of blood coagulation | 0.034984 |
|  | GOTERM_BP_1 | GO:0051704~multi-organism process | 0.034992 |
|  | GOTERM_BP_4 | GO:0007399~nervous system development | 0.036617 |
|  | GOTERM_BP_3 | GO:0051241~negative regulation of multicellular organismal process | 0.036838 |
|  | GOTERM_BP_2 | GO:0051241~negative regulation of multicellular organismal process | 0.037148 |
|  | GOTERM_BP_4 | GO:0030195~negative regulation of blood coagulation | 0.039425 |
|  | GOTERM_BP_5 | GO:0050819~negative regulation of coagulation | 0.039461 |
|  | GOTERM_BP_3 | GO:0048519~negative regulation of biological process | 0.042179 |
|  | GOTERM_CC_5 | GO:0005581~collagen | 0.042804 |
|  | GOTERM_BP_4 | GO:0042060~wound healing | 0.043338 |
|  | GOTERM_CC_5 | GO:0031410~cytoplasmic vesicle | 0.043484 |
|  | GOTERM_BP_2 | GO:0048519~negative regulation of biological process | 0.043522 |
|  | GOTERM_BP_4 | GO:0050819~negative regulation of coagulation | 0.044457 |
|  | GOTERM_BP_3 | GO:0050819~negative regulation of coagulation | 0.047219 |
|  | GOTERM_CC_4 | GO:0016023~cytoplasmic membrane-bounded vesicle | 0.047558 |
|  | GOTERM_BP_5 | GO:0042246~tissue regeneration | 0.048356 |
| 5240 | GOTERM_BP_5 | GO:0019932~second-messenger-mediated signaling | 3.94E-16 |
|  | GOTERM_MF_5 | GO:0004930~G-protein coupled receptor activity | 2.30E-12 |
|  | GOTERM_BP_5 | GO:0007186~G-protein coupled receptor protein signaling pathway | 1.25E-11 |
|  | GOTERM_BP_4 | GO:0007166~cell surface receptor linked signal transduction | 3.06E-09 |
|  | GOTERM_MF_4 | GO:0004888~transmembrane receptor activity | 3.90E-09 |
|  | GOTERM_MF_1 | GO:0060089~molecular transducer activity | 6.75E-09 |
|  | GOTERM_BP_5 | GO:0007166~cell surface receptor linked signal transduction | 7.31E-09 |
|  | GOTERM_MF_2 | GO:0004871~signal transducer activity | 8.76E-09 |
|  | GOTERM_BP_4 | GO:0007242~intracellular signaling cascade | 1.58E-08 |
|  | GOTERM_BP_3 | GO:0007165~signal transduction | 2.61E-08 |
|  | GOTERM_CC_5 | GO:0005887~integral to plasma membrane | 2.83E-08 |
|  | GOTERM_CC_4 | GO:0031226~intrinsic to plasma membrane | 2.99E-08 |
|  | GOTERM_BP_5 | GO:0007242~intracellular signaling cascade | 3.27E-08 |
|  | GOTERM_BP_4 | GO:0007165~signal transduction | 3.50E-08 |
|  | GOTERM_CC_5 | GO:0031226~intrinsic to plasma membrane | 3.51E-08 |
|  | GOTERM_MF_3 | GO:0004872~receptor activity | 6.18E-08 |
|  | GOTERM_CC_3 | GO:0005886~plasma membrane | 7.89E-07 |
|  | GOTERM_CC_4 | GO:0005886~plasma membrane | 1.31E-06 |
|  | GOTERM_CC_3 | GO:0044459~plasma membrane part | 5.69E-06 |
|  | GOTERM_CC_4 | GO:0044459~plasma membrane part | 8.31E-06 |
|  | GOTERM_CC_5 | GO:0044459~plasma membrane part | 9.69E-06 |
|  | GOTERM_CC_3 | GO:0031224~intrinsic to membrane | 7.60E-05 |
|  | GOTERM_CC_4 | GO:0016021~integral to membrane | 8.19E-05 |
|  | GOTERM_CC_5 | GO:0016021~integral to membrane | 9.99E-05 |
|  | GOTERM_CC_4 | GO:0031224~intrinsic to membrane | 1.24E-04 |
|  | GOTERM_CC_5 | GO:0031224~intrinsic to membrane | 1.52E-04 |
|  | GOTERM_BP_5 | GO:0060191~regulation of lipase activity | 1.79E-04 |
|  | GOTERM_BP_3 | GO:0050790~regulation of catalytic activity | 2.81E-04 |
|  | GOTERM_BP_4 | GO:0043085~positive regulation of catalytic activity | 2.86E-04 |
|  | GOTERM_MF_3 | GO:0001653~peptide receptor activity | 3.35E-04 |
|  | GOTERM_MF_5 | GO:0008528~peptide receptor activity, G-protein coupled | 3.84E-04 |
|  | GOTERM_BP_3 | GO:0044093~positive regulation of molecular function | 4.50E-04 |
|  | GOTERM_MF_4 | GO:0001653~peptide receptor activity | 4.52E-04 |
|  | GOTERM_MF_4 | GO:0008528~peptide receptor activity, G-protein coupled | 4.52E-04 |
|  | GOTERM_BP_2 | GO:0065009~regulation of molecular function | 4.84E-04 |
|  | GOTERM_BP_4 | GO:0006954~inflammatory response | 5.23E-04 |
|  | GOTERM_CC_2 | GO:0044425~membrane part | 6.51E-04 |
|  | GOTERM_CC_3 | GO:0044425~membrane part | 6.56E-04 |
|  | GOTERM_CC_4 | GO:0044425~membrane part | 0.001059 |
|  | GOTERM_MF_2 | GO:0042277~peptide binding | 0.001242 |
|  | GOTERM_BP_5 | GO:0051345~positive regulation of hydrolase activity | 0.001479 |
|  | GOTERM_BP_2 | GO:0050794~regulation of cellular process | 0.001582 |
|  | GOTERM_BP_2 | GO:0007610~behavior | 0.001666 |
|  | GOTERM_CC_2 | GO:0016020~membrane | 0.002058 |
|  | GOTERM_CC_3 | GO:0016020~membrane | 0.002075 |
|  | GOTERM_BP_3 | GO:0050794~regulation of cellular process | 0.002437 |
|  | GOTERM_MF_5 | GO:0004918~interleukin-8 receptor activity | 0.002609 |
|  | GOTERM_BP_2 | GO:0050789~regulation of biological process | 0.002624 |
|  | GOTERM_BP_2 | GO:0009605~response to external stimulus | 0.002843 |
|  | GOTERM_BP_3 | GO:0009611~response to wounding | 0.002962 |
|  | GOTERM_BP_1 | GO:0065007~biological regulation | 0.00449 |
|  | GOTERM_BP_3 | GO:0006952~defense response | 0.005056 |
|  | GOTERM_MF_2 | GO:0042165~neurotransmitter binding | 0.005384 |
|  | GOTERM_MF_3 | GO:0030594~neurotransmitter receptor activity | 0.005887 |
|  | GOTERM_MF_2 | GO:0043176~amine binding | 0.006117 |
|  | GOTERM_BP_4 | GO:0031279~regulation of cyclase activity | 0.006264 |
|  | GOTERM_BP_4 | GO:0051339~regulation of lyase activity | 0.006511 |
|  | GOTERM_MF_5 | GO:0004994~somatostatin receptor activity | 0.006512 |
|  | GOTERM_BP_5 | GO:0045761~regulation of adenylate cyclase activity | 0.006745 |
|  | GOTERM_MF_4 | GO:0030594~neurotransmitter receptor activity | 0.007183 |
|  | GOTERM_BP_4 | GO:0051336~regulation of hydrolase activity | 0.007254 |
|  | GOTERM_BP_1 | GO:0050896~response to stimulus | 0.007279 |
|  | GOTERM_CC_3 | GO:0005626~insoluble fraction | 0.007567 |
|  | GOTERM_CC_4 | GO:0005624~membrane fraction | 0.00769 |
|  | GOTERM_CC_5 | GO:0005624~membrane fraction | 0.00815 |
|  | GOTERM_CC_4 | GO:0005626~insoluble fraction | 0.008736 |
|  | GOTERM_BP_5 | GO:0030808~regulation of nucleotide biosynthetic process | 0.008775 |
|  | GOTERM_BP_5 | GO:0006140~regulation of nucleotide metabolic process | 0.009718 |
|  | GOTERM_MF_5 | GO:0019958~C-X-C chemokine binding | 0.011693 |
|  | GOTERM_CC_4 | GO:0030425~dendrite | 0.01172 |
|  | GOTERM_CC_5 | GO:0030425~dendrite | 0.012096 |
|  | GOTERM_BP_5 | GO:0045744~negative regulation of G-protein coupled receptor protein signaling pathway | 0.018079 |
|  | GOTERM_CC_2 | GO:0000267~cell fraction | 0.018169 |
|  | GOTERM_CC_3 | GO:0000267~cell fraction | 0.018213 |
|  | GOTERM_BP_3 | GO:0003013~circulatory system process | 0.020061 |
|  | GOTERM_BP_4 | GO:0008015~blood circulation | 0.020893 |
|  | GOTERM_CC_2 | GO:0042995~cell projection | 0.028461 |
|  | GOTERM_CC_3 | GO:0042995~cell projection | 0.028514 |
|  | GOTERM_BP_3 | GO:0007267~cell-cell signaling | 0.031975 |
|  | GOTERM_MF_5 | GO:0004950~chemokine receptor activity | 0.032169 |
|  | GOTERM_BP_5 | GO:0003044~regulation of systemic arterial blood pressure mediated by a chemical signal | 0.034592 |
|  | GOTERM_BP_2 | GO:0006950~response to stress | 0.035663 |
|  | GOTERM_MF_4 | GO:0019956~chemokine binding | 0.036092 |
|  | GOTERM_BP_3 | GO:0007626~locomotory behavior | 0.041047 |
|  | GOTERM_BP_4 | GO:0003073~regulation of systemic arterial blood pressure | 0.041695 |
|  | GOTERM_CC_3 | GO:0043005~neuron projection | 0.043267 |
|  | GOTERM_CC_4 | GO:0043005~neuron projection | 0.046596 |
|  | GOTERM_MF_3 | GO:0042923~neuropeptide binding | 0.049125 |
| 5316 | GOTERM_MF_3 | GO:0008233~peptidase activity | 8.11E-06 |
|  | GOTERM_MF_4 | GO:0070011~peptidase activity, acting on L-amino acid peptides | 1.08E-05 |
|  | GOTERM_BP_5 | GO:0006508~proteolysis | 2.06E-04 |
|  | GOTERM_CC_2 | GO:0005615~extracellular space | 2.42E-04 |
|  | GOTERM_CC_3 | GO:0005615~extracellular space | 2.42E-04 |
|  | GOTERM_MF_5 | GO:0008237~metallopeptidase activity | 3.19E-04 |
|  | GOTERM_CC_1 | GO:0044421~extracellular region part | 7.58E-04 |
|  | GOTERM_CC_2 | GO:0044421~extracellular region part | 8.80E-04 |
|  | GOTERM_BP_2 | GO:0007586~digestion | 0.00115 |
|  | GOTERM_MF_5 | GO:0008238~exopeptidase activity | 0.001603 |
|  | GOTERM_BP_5 | GO:0002035~brain renin-angiotensin system | 0.001951 |
|  | GOTERM_MF_2 | GO:0016787~hydrolase activity | 0.003209 |
|  | GOTERM_BP_4 | GO:0042756~drinking behavior | 0.004849 |
|  | GOTERM_BP_4 | GO:0001976~neurological system process involved in regulation of systemic arterial blood pressure | 0.005454 |
|  | GOTERM_BP_5 | GO:0001976~neurological system process involved in regulation of systemic arterial blood pressure | 0.005844 |
|  | GOTERM_BP_5 | GO:0003081~regulation of systemic arterial blood pressure by renin-angiotensin | 0.007139 |
|  | GOTERM_CC_1 | GO:0005576~extracellular region | 0.011665 |
|  | GOTERM_BP_4 | GO:0001990~regulation of systemic arterial blood pressure by hormone | 0.012084 |
|  | GOTERM_BP_3 | GO:0019538~protein metabolic process | 0.012567 |
|  | GOTERM_BP_3 | GO:0050886~endocrine process | 0.013589 |
|  | GOTERM_BP_4 | GO:0019538~protein metabolic process | 0.013817 |
|  | GOTERM_CC_5 | GO:0005887~integral to plasma membrane | 0.015144 |
|  | GOTERM_CC_5 | GO:0031226~intrinsic to plasma membrane | 0.016106 |
|  | GOTERM_BP_5 | GO:0003044~regulation of systemic arterial blood pressure mediated by a chemical signal | 0.017443 |
|  | GOTERM_BP_4 | GO:0019229~regulation of vasoconstriction | 0.020467 |
|  | GOTERM_BP_4 | GO:0003073~regulation of systemic arterial blood pressure | 0.021063 |
|  | GOTERM_BP_5 | GO:0019229~regulation of vasoconstriction | 0.021921 |
|  | GOTERM_CC_4 | GO:0031226~intrinsic to plasma membrane | 0.023128 |
|  | GOTERM_BP_2 | GO:0008283~cell proliferation | 0.024102 |
|  | GOTERM_MF_5 | GO:0004175~endopeptidase activity | 0.03407 |
|  | GOTERM_BP_3 | GO:0006800~oxygen and reactive oxygen species metabolic process | 0.039136 |
|  | GOTERM_BP_3 | GO:0007631~feeding behavior | 0.040284 |
|  | GOTERM_BP_4 | GO:0050727~regulation of inflammatory response | 0.045242 |
|  | GOTERM_BP_5 | GO:0050727~regulation of inflammatory response | 0.048419 |
| 5331 | GOTERM_BP_2 | GO:0048518~positive regulation of biological process | 3.46E-04 |
|  | GOTERM_BP_2 | GO:0002682~regulation of immune system process | 3.93E-04 |
|  | GOTERM_BP_3 | GO:0048518~positive regulation of biological process | 4.10E-04 |
|  | GOTERM_BP_3 | GO:0002682~regulation of immune system process | 4.35E-04 |
|  | GOTERM_BP_4 | GO:0002697~regulation of immune effector process | 8.56E-04 |
|  | GOTERM_BP_5 | GO:0002697~regulation of immune effector process | 9.81E-04 |
|  | GOTERM_BP_2 | GO:0048522~positive regulation of cellular process | 0.003683 |
|  | GOTERM_BP_3 | GO:0050776~regulation of immune response | 0.004025 |
|  | GOTERM_BP_2 | GO:0048584~positive regulation of response to stimulus | 0.00409 |
|  | GOTERM_BP_4 | GO:0050776~regulation of immune response | 0.0042 |
|  | GOTERM_BP_3 | GO:0048522~positive regulation of cellular process | 0.004204 |
|  | GOTERM_BP_3 | GO:0048584~positive regulation of response to stimulus | 0.004381 |
|  | GOTERM_BP_4 | GO:0048522~positive regulation of cellular process | 0.004561 |
|  | GOTERM_BP_4 | GO:0048584~positive regulation of response to stimulus | 0.004571 |
|  | GOTERM_CC_2 | GO:0009986~cell surface | 0.004933 |
|  | GOTERM_CC_3 | GO:0009986~cell surface | 0.00494 |
|  | GOTERM_MF_3 | GO:0005102~receptor binding | 0.005338 |
|  | GOTERM_BP_4 | GO:0009967~positive regulation of signal transduction | 0.007063 |
|  | GOTERM_BP_5 | GO:0009967~positive regulation of signal transduction | 0.008078 |
|  | GOTERM_BP_3 | GO:0010647~positive regulation of cell communication | 0.008368 |
|  | GOTERM_BP_4 | GO:0010647~positive regulation of cell communication | 0.008727 |
|  | GOTERM_BP_5 | GO:0010647~positive regulation of cell communication | 0.009976 |
|  | GOTERM_BP_2 | GO:0031343~positive regulation of cell killing | 0.010284 |
|  | GOTERM_BP_3 | GO:0031343~positive regulation of cell killing | 0.01065 |
|  | GOTERM_BP_5 | GO:0045670~regulation of osteoclast differentiation | 0.010693 |
|  | GOTERM_BP_4 | GO:0031343~positive regulation of cell killing | 0.010883 |
|  | GOTERM_BP_2 | GO:0031341~regulation of cell killing | 0.011989 |
|  | GOTERM_BP_5 | GO:0032768~regulation of monooxygenase activity | 0.012144 |
|  | GOTERM_BP_3 | GO:0031341~regulation of cell killing | 0.012416 |
|  | GOTERM_BP_5 | GO:0051353~positive regulation of oxidoreductase activity | 0.012627 |
|  | GOTERM_BP_3 | GO:0008284~positive regulation of cell proliferation | 0.013035 |
|  | GOTERM_BP_4 | GO:0008284~positive regulation of cell proliferation | 0.013589 |
|  | GOTERM_BP_4 | GO:0002821~positive regulation of adaptive immune response | 0.014039 |
|  | GOTERM_BP_5 | GO:0002824~positive regulation of adaptive immune response based on somatic recombination of immune receptors built from immunoglobulin superfamily domains | 0.014558 |
|  | GOTERM_BP_5 | GO:0002821~positive regulation of adaptive immune response | 0.01504 |
|  | GOTERM_BP_2 | GO:0048583~regulation of response to stimulus | 0.015224 |
|  | GOTERM_BP_4 | GO:0002705~positive regulation of leukocyte mediated immunity | 0.015388 |
|  | GOTERM_BP_5 | GO:0008284~positive regulation of cell proliferation | 0.015514 |
|  | GOTERM_BP_3 | GO:0048583~regulation of response to stimulus | 0.016281 |
|  | GOTERM_BP_5 | GO:0002705~positive regulation of leukocyte mediated immunity | 0.016485 |
|  | GOTERM_BP_5 | GO:0002708~positive regulation of lymphocyte mediated immunity | 0.016485 |
|  | GOTERM_BP_5 | GO:0002573~myeloid leukocyte differentiation | 0.016485 |
|  | GOTERM_CC_1 | GO:0005576~extracellular region | 0.016525 |
|  | GOTERM_BP_4 | GO:0051341~regulation of oxidoreductase activity | 0.018083 |
|  | GOTERM_BP_2 | GO:0065008~regulation of biological quality | 0.018266 |
|  | GOTERM_CC_2 | GO:0005615~extracellular space | 0.018302 |
|  | GOTERM_CC_3 | GO:0005615~extracellular space | 0.018327 |
|  | GOTERM_BP_4 | GO:0002761~regulation of myeloid leukocyte differentiation | 0.018532 |
|  | GOTERM_MF_5 | GO:0004175~endopeptidase activity | 0.019185 |
|  | GOTERM_BP_3 | GO:0002699~positive regulation of immune effector process | 0.019453 |
|  | GOTERM_BP_5 | GO:0002761~regulation of myeloid leukocyte differentiation | 0.019851 |
|  | GOTERM_BP_4 | GO:0002699~positive regulation of immune effector process | 0.019877 |
|  | GOTERM_BP_4 | GO:0043085~positive regulation of catalytic activity | 0.020987 |
|  | GOTERM_BP_5 | GO:0002699~positive regulation of immune effector process | 0.02129 |
|  | GOTERM_BP_1 | GO:0065007~biological regulation | 0.022811 |
|  | GOTERM_BP_4 | GO:0002819~regulation of adaptive immune response | 0.02524 |
|  | GOTERM_BP_3 | GO:0044093~positive regulation of molecular function | 0.025246 |
|  | GOTERM_MF_3 | GO:0008233~peptidase activity | 0.025744 |
|  | GOTERM_BP_5 | GO:0002706~regulation of lymphocyte mediated immunity | 0.026076 |
|  | GOTERM_BP_3 | GO:0045785~positive regulation of cell adhesion | 0.026449 |
|  | GOTERM_BP_5 | GO:0002822~regulation of adaptive immune response based on somatic recombination of immune receptors built from immunoglobulin superfamily domains | 0.026554 |
|  | GOTERM_BP_4 | GO:0045785~positive regulation of cell adhesion | 0.027022 |
|  | GOTERM_BP_5 | GO:0002819~regulation of adaptive immune response | 0.027031 |
|  | GOTERM_BP_2 | GO:0051235~maintenance of location | 0.027228 |
|  | GOTERM_BP_4 | GO:0002703~regulation of leukocyte mediated immunity | 0.027468 |
|  | GOTERM_MF_2 | GO:0005515~protein binding | 0.027663 |
|  | GOTERM_BP_3 | GO:0051235~maintenance of location | 0.028191 |
|  | GOTERM_MF_4 | GO:0070011~peptidase activity, acting on L-amino acid peptides | 0.028732 |
|  | GOTERM_BP_5 | GO:0045785~positive regulation of cell adhesion | 0.028938 |
|  | GOTERM_BP_5 | GO:0002703~regulation of leukocyte mediated immunity | 0.029414 |
|  | GOTERM_MF_4 | GO:0005178~integrin binding | 0.029701 |
|  | GOTERM_BP_3 | GO:0045637~regulation of myeloid cell differentiation | 0.0308 |
|  | GOTERM_BP_4 | GO:0045637~regulation of myeloid cell differentiation | 0.031466 |
|  | GOTERM_CC_1 | GO:0044421~extracellular region part | 0.032194 |
|  | GOTERM_BP_5 | GO:0045637~regulation of myeloid cell differentiation | 0.033693 |
|  | GOTERM_CC_2 | GO:0044421~extracellular region part | 0.034658 |
|  | GOTERM_BP_3 | GO:0042592~homeostatic process | 0.040119 |
|  | GOTERM_BP_5 | GO:0006606~protein import into nucleus | 0.041259 |
|  | GOTERM_BP_4 | GO:0030099~myeloid cell differentiation | 0.041623 |
|  | GOTERM_BP_3 | GO:0042127~regulation of cell proliferation | 0.04374 |
|  | GOTERM_BP_5 | GO:0030099~myeloid cell differentiation | 0.044554 |
|  | GOTERM_BP_4 | GO:0042127~regulation of cell proliferation | 0.045521 |
|  | GOTERM_CC_3 | GO:0045121~membrane raft | 0.045923 |
|  | GOTERM_CC_4 | GO:0045121~membrane raft | 0.047811 |
|  | GOTERM_CC_5 | GO:0045121~membrane raft | 0.048594 |
|  | GOTERM_BP_3 | GO:0050790~regulation of catalytic activity | 0.049947 |
| 5414 | GOTERM_BP_5 | GO:0006281~DNA repair | 4.09E-20 |
|  | GOTERM_BP_4 | GO:0006259~DNA metabolic process | 1.84E-19 |
|  | GOTERM_BP_3 | GO:0006974~response to DNA damage stimulus | 4.59E-19 |
|  | GOTERM_BP_5 | GO:0006259~DNA metabolic process | 4.85E-19 |
|  | GOTERM_BP_5 | GO:0006310~DNA recombination | 3.85E-18 |
|  | GOTERM_MF_4 | GO:0043566~structure-specific DNA binding | 1.62E-17 |
|  | GOTERM_BP_3 | GO:0033554~cellular response to stress | 1.08E-16 |
|  | GOTERM_BP_2 | GO:0051716~cellular response to stimulus | 8.67E-15 |
|  | GOTERM_CC_4 | GO:0000228~nuclear chromosome | 9.30E-15 |
|  | GOTERM_CC_5 | GO:0000228~nuclear chromosome | 1.08E-14 |
|  | GOTERM_CC_3 | GO:0044428~nuclear part | 1.50E-12 |
|  | GOTERM_CC_4 | GO:0044428~nuclear part | 2.64E-12 |
|  | GOTERM_CC_5 | GO:0044428~nuclear part | 3.33E-12 |
|  | GOTERM_MF_5 | GO:0003697~single-stranded DNA binding | 3.57E-11 |
|  | GOTERM_BP_2 | GO:0006950~response to stress | 9.32E-11 |
|  | GOTERM_BP_4 | GO:0032200~telomere organization | 1.06E-10 |
|  | GOTERM_CC_4 | GO:0005694~chromosome | 1.15E-10 |
|  | GOTERM_BP_5 | GO:0000723~telomere maintenance | 1.24E-10 |
|  | GOTERM_CC_5 | GO:0005694~chromosome | 1.33E-10 |
|  | GOTERM_BP_4 | GO:0060249~anatomical structure homeostasis | 1.11E-09 |
|  | GOTERM_CC_4 | GO:0005654~nucleoplasm | 1.34E-09 |
|  | GOTERM_CC_4 | GO:0044454~nuclear chromosome part | 1.38E-09 |
|  | GOTERM_CC_5 | GO:0044454~nuclear chromosome part | 1.52E-09 |
|  | GOTERM_CC_5 | GO:0005654~nucleoplasm | 1.57E-09 |
|  | GOTERM_MF_3 | GO:0003677~DNA binding | 2.14E-09 |
|  | GOTERM_BP_3 | GO:0051276~chromosome organization | 1.38E-08 |
|  | GOTERM_CC_3 | GO:0044427~chromosomal part | 3.36E-08 |
|  | GOTERM_CC_4 | GO:0044427~chromosomal part | 4.44E-08 |
|  | GOTERM_BP_3 | GO:0009314~response to radiation | 4.46E-08 |
|  | GOTERM_CC_5 | GO:0044427~chromosomal part | 4.97E-08 |
|  | GOTERM_BP_3 | GO:0006139~nucleobase, nucleoside, nucleotide and nucleic acid metabolic process | 5.01E-08 |
|  | GOTERM_BP_5 | GO:0006260~DNA replication | 5.62E-08 |
|  | GOTERM_CC_1 | GO:0044422~organelle part | 1.05E-07 |
|  | GOTERM_CC_4 | GO:0005634~nucleus | 1.21E-07 |
|  | GOTERM_BP_2 | GO:0006807~nitrogen compound metabolic process | 1.26E-07 |
|  | GOTERM_BP_3 | GO:0034641~cellular nitrogen compound metabolic process | 1.38E-07 |
|  | GOTERM_CC_5 | GO:0005634~nucleus | 1.55E-07 |
|  | GOTERM_CC_4 | GO:0031981~nuclear lumen | 1.63E-07 |
|  | GOTERM_CC_2 | GO:0044446~intracellular organelle part | 1.65E-07 |
|  | GOTERM_CC_3 | GO:0044446~intracellular organelle part | 1.67E-07 |
|  | GOTERM_CC_2 | GO:0044422~organelle part | 1.80E-07 |
|  | GOTERM_MF_2 | GO:0003676~nucleic acid binding | 1.87E-07 |
|  | GOTERM_CC_5 | GO:0031981~nuclear lumen | 1.91E-07 |
|  | GOTERM_CC_4 | GO:0044446~intracellular organelle part | 2.93E-07 |
|  | GOTERM_CC_5 | GO:0044446~intracellular organelle part | 3.67E-07 |
|  | GOTERM_CC_3 | GO:0070013~intracellular organelle lumen | 7.70E-07 |
|  | GOTERM_CC_1 | GO:0031974~membrane-enclosed lumen | 7.87E-07 |
|  | GOTERM_BP_1 | GO:0050896~response to stimulus | 8.75E-07 |
|  | GOTERM_CC_2 | GO:0043233~organelle lumen | 9.48E-07 |
|  | GOTERM_CC_3 | GO:0043233~organelle lumen | 9.54E-07 |
|  | GOTERM_CC_4 | GO:0070013~intracellular organelle lumen | 1.13E-06 |
|  | GOTERM_CC_2 | GO:0032993~protein-DNA complex | 1.21E-06 |
|  | GOTERM_CC_5 | GO:0070013~intracellular organelle lumen | 1.32E-06 |
|  | GOTERM_BP_2 | GO:0009628~response to abiotic stimulus | 1.33E-06 |
|  | GOTERM_BP_2 | GO:0006996~organelle organization | 1.89E-06 |
|  | GOTERM_CC_4 | GO:0000781~chromosome, telomeric region | 3.11E-06 |
|  | GOTERM_CC_5 | GO:0000781~chromosome, telomeric region | 3.27E-06 |
|  | GOTERM_BP_3 | GO:0051321~meiotic cell cycle | 3.67E-06 |
|  | GOTERM_BP_4 | GO:0007126~meiosis | 3.69E-06 |
|  | GOTERM_BP_4 | GO:0051327~M phase of meiotic cell cycle | 3.69E-06 |
|  | GOTERM_CC_4 | GO:0005657~replication fork | 4.22E-06 |
|  | GOTERM_CC_5 | GO:0005657~replication fork | 4.43E-06 |
|  | GOTERM_BP_5 | GO:0007126~meiosis | 4.85E-06 |
|  | GOTERM_BP_5 | GO:0051327~M phase of meiotic cell cycle | 4.85E-06 |
|  | GOTERM_MF_5 | GO:0003690~double-stranded DNA binding | 5.66E-06 |
|  | GOTERM_CC_5 | GO:0005662~DNA replication factor A complex | 1.51E-05 |
|  | GOTERM_BP_3 | GO:0044260~cellular macromolecule metabolic process | 1.62E-05 |
|  | GOTERM_CC_5 | GO:0000794~condensed nuclear chromosome | 1.63E-05 |
|  | GOTERM_CC_2 | GO:0043228~non-membrane-bounded organelle | 2.56E-05 |
|  | GOTERM_CC_3 | GO:0043232~intracellular non-membrane-bounded organelle | 2.57E-05 |
|  | GOTERM_BP_2 | GO:0043170~macromolecule metabolic process | 3.42E-05 |
|  | GOTERM_CC_4 | GO:0043232~intracellular non-membrane-bounded organelle | 3.74E-05 |
|  | GOTERM_BP_1 | GO:0016043~cellular component organization | 3.89E-05 |
|  | GOTERM_CC_5 | GO:0043232~intracellular non-membrane-bounded organelle | 4.34E-05 |
|  | GOTERM_BP_5 | GO:0006308~DNA catabolic process | 4.83E-05 |
|  | GOTERM_CC_3 | GO:0043231~intracellular membrane-bounded organelle | 5.83E-05 |
|  | GOTERM_CC_2 | GO:0043227~membrane-bounded organelle | 5.85E-05 |
|  | GOTERM_BP_3 | GO:0022403~cell cycle phase | 6.25E-05 |
|  | GOTERM_BP_4 | GO:0022403~cell cycle phase | 6.92E-05 |
|  | GOTERM_CC_3 | GO:0030894~replisome | 8.13E-05 |
|  | GOTERM_CC_4 | GO:0043601~nuclear replisome | 8.82E-05 |
|  | GOTERM_CC_4 | GO:0030894~replisome | 8.82E-05 |
|  | GOTERM_CC_5 | GO:0043601~nuclear replisome | 9.12E-05 |
|  | GOTERM_CC_5 | GO:0030894~replisome | 9.12E-05 |
|  | GOTERM_BP_3 | GO:0042592~homeostatic process | 9.57E-05 |
|  | GOTERM_CC_5 | GO:0043596~nuclear replication fork | 1.05E-04 |
|  | GOTERM_CC_4 | GO:0043231~intracellular membrane-bounded organelle | 1.08E-04 |
|  | GOTERM_CC_5 | GO:0043231~intracellular membrane-bounded organelle | 1.39E-04 |
|  | GOTERM_BP_2 | GO:0002520~immune system development | 1.73E-04 |
|  | GOTERM_CC_1 | GO:0043226~organelle | 1.90E-04 |
|  | GOTERM_CC_5 | GO:0000784~nuclear chromosome, telomeric region | 2.09E-04 |
|  | GOTERM_BP_4 | GO:0002520~immune system development | 2.14E-04 |
|  | GOTERM_BP_2 | GO:0022402~cell cycle process | 2.30E-04 |
|  | GOTERM_BP_2 | GO:0044237~cellular metabolic process | 2.53E-04 |
|  | GOTERM_BP_3 | GO:0022402~cell cycle process | 2.70E-04 |
|  | GOTERM_CC_5 | GO:0000793~condensed chromosome | 2.94E-04 |
|  | GOTERM_BP_3 | GO:0007131~reciprocal meiotic recombination | 3.15E-04 |
|  | GOTERM_BP_4 | GO:0007131~reciprocal meiotic recombination | 3.29E-04 |
|  | GOTERM_CC_2 | GO:0043229~intracellular organelle | 3.37E-04 |
|  | GOTERM_CC_3 | GO:0043229~intracellular organelle | 3.40E-04 |
|  | GOTERM_BP_5 | GO:0007131~reciprocal meiotic recombination | 3.78E-04 |
|  | GOTERM_MF_3 | GO:0008022~protein C-terminus binding | 4.12E-04 |
|  | GOTERM_BP_4 | GO:0000279~M phase | 4.19E-04 |
|  | GOTERM_BP_2 | GO:0044238~primary metabolic process | 4.42E-04 |
|  | GOTERM_MF_2 | GO:0001882~nucleoside binding | 5.18E-04 |
|  | GOTERM_BP_5 | GO:0000279~M phase | 5.44E-04 |
|  | GOTERM_MF_4 | GO:0030554~adenyl nucleotide binding | 6.26E-04 |
|  | GOTERM_CC_4 | GO:0043229~intracellular organelle | 6.30E-04 |
|  | GOTERM_MF_3 | GO:0001883~purine nucleoside binding | 6.46E-04 |
|  | GOTERM_MF_2 | GO:0000166~nucleotide binding | 6.46E-04 |
|  | GOTERM_CC_5 | GO:0043229~intracellular organelle | 8.08E-04 |
|  | GOTERM_MF_3 | GO:0016817~hydrolase activity, acting on acid anhydrides | 8.71E-04 |
|  | GOTERM_MF_4 | GO:0016818~hydrolase activity, acting on acid anhydrides, in phosphorus-containing anhydrides | 9.21E-04 |
|  | GOTERM_BP_2 | GO:0007049~cell cycle | 9.90E-04 |
|  | GOTERM_BP_4 | GO:0007127~meiosis I | 0.001015 |
|  | GOTERM_BP_5 | GO:0007127~meiosis I | 0.001163 |
|  | GOTERM_MF_4 | GO:0003684~damaged DNA binding | 0.001338 |
|  | GOTERM_BP_1 | GO:0008152~metabolic process | 0.001469 |
|  | GOTERM_MF_5 | GO:0016462~pyrophosphatase activity | 0.001925 |
|  | GOTERM_MF_3 | GO:0017076~purine nucleotide binding | 0.001941 |
|  | GOTERM_CC_4 | GO:0044451~nucleoplasm part | 0.001983 |
|  | GOTERM_BP_4 | GO:0010212~response to ionizing radiation | 0.002062 |
|  | GOTERM_CC_5 | GO:0044451~nucleoplasm part | 0.002107 |
|  | GOTERM_BP_4 | GO:0030097~hemopoiesis | 0.002202 |
|  | GOTERM_BP_5 | GO:0009411~response to UV | 0.002285 |
|  | GOTERM_BP_5 | GO:0031297~replication fork processing | 0.002439 |
|  | GOTERM_BP_5 | GO:0030097~hemopoiesis | 0.00268 |
|  | GOTERM_BP_3 | GO:0048534~hemopoietic or lymphoid organ development | 0.002728 |
|  | GOTERM_BP_4 | GO:0048534~hemopoietic or lymphoid organ development | 0.0029 |
|  | GOTERM_BP_2 | GO:0065008~regulation of biological quality | 0.002923 |
|  | GOTERM_BP_5 | GO:0048534~hemopoietic or lymphoid organ development | 0.003525 |
|  | GOTERM_MF_5 | GO:0051880~G-quadruplex DNA binding | 0.003912 |
|  | GOTERM_CC_1 | GO:0032991~macromolecular complex | 0.003996 |
|  | GOTERM_BP_5 | GO:0042770~DNA damage response, signal transduction | 0.004157 |
|  | GOTERM_MF_2 | GO:0016787~hydrolase activity | 0.004179 |
|  | GOTERM_CC_2 | GO:0044424~intracellular part | 0.004219 |
|  | GOTERM_CC_3 | GO:0044424~intracellular part | 0.004265 |
|  | GOTERM_CC_3 | GO:0070419~nonhomologous end joining complex | 0.004898 |
|  | GOTERM_CC_4 | GO:0005958~DNA-dependent protein kinase-DNA ligase 4 complex | 0.005103 |
|  | GOTERM_CC_4 | GO:0070419~nonhomologous end joining complex | 0.005103 |
|  | GOTERM_CC_5 | GO:0070419~nonhomologous end joining complex | 0.005189 |
|  | GOTERM_CC_5 | GO:0005958~DNA-dependent protein kinase-DNA ligase 4 complex | 0.005189 |
|  | GOTERM_BP_5 | GO:0045005~maintenance of fidelity during DNA-dependent DNA replication | 0.006088 |
|  | GOTERM_CC_2 | GO:0005622~intracellular | 0.007062 |
|  | GOTERM_CC_3 | GO:0005622~intracellular | 0.007139 |
|  | GOTERM_CC_2 | GO:0043234~protein complex | 0.007167 |
|  | GOTERM_BP_4 | GO:0051052~regulation of DNA metabolic process | 0.007241 |
|  | GOTERM_MF_5 | GO:0032559~adenyl ribonucleotide binding | 0.007577 |
|  | GOTERM_CC_4 | GO:0044424~intracellular part | 0.007899 |
|  | GOTERM_BP_5 | GO:0051052~regulation of DNA metabolic process | 0.008274 |
|  | GOTERM_MF_3 | GO:0032553~ribonucleotide binding | 0.008388 |
|  | GOTERM_CC_3 | GO:0000782~telomere cap complex | 0.008801 |
|  | GOTERM_BP_3 | GO:0030069~lysogeny | 0.008881 |
|  | GOTERM_BP_3 | GO:0019047~provirus integration | 0.008881 |
|  | GOTERM_BP_4 | GO:0019047~provirus integration | 0.009075 |
|  | GOTERM_BP_4 | GO:0030069~lysogeny | 0.009075 |
|  | GOTERM_MF_4 | GO:0032555~purine ribonucleotide binding | 0.009101 |
|  | GOTERM_CC_4 | GO:0000782~telomere cap complex | 0.009169 |
|  | GOTERM_CC_4 | GO:0000783~nuclear telomere cap complex | 0.009169 |
|  | GOTERM_BP_2 | GO:0048522~positive regulation of cellular process | 0.009215 |
|  | GOTERM_CC_5 | GO:0000783~nuclear telomere cap complex | 0.009321 |
|  | GOTERM_CC_5 | GO:0000782~telomere cap complex | 0.009321 |
|  | GOTERM_BP_5 | GO:0019047~provirus integration | 0.009724 |
|  | GOTERM_BP_4 | GO:0009416~response to light stimulus | 0.010461 |
|  | GOTERM_BP_3 | GO:0048522~positive regulation of cellular process | 0.010933 |
|  | GOTERM_BP_4 | GO:0033151~V(D)J recombination | 0.011332 |
|  | GOTERM_MF_5 | GO:0000217~DNA secondary structure binding | 0.011693 |
|  | GOTERM_BP_4 | GO:0048522~positive regulation of cellular process | 0.012139 |
|  | GOTERM_BP_5 | GO:0033151~V(D)J recombination | 0.012141 |
|  | GOTERM_MF_4 | GO:0004518~nuclease activity | 0.012669 |
|  | GOTERM_CC_5 | GO:0016604~nuclear body | 0.012813 |
|  | GOTERM_BP_1 | GO:0009987~cellular process | 0.013382 |
|  | GOTERM_BP_2 | GO:0048518~positive regulation of biological process | 0.014639 |
|  | GOTERM_BP_3 | GO:0048518~positive regulation of biological process | 0.01729 |
|  | GOTERM_BP_1 | GO:0002376~immune system process | 0.01835 |
|  | GOTERM_MF_3 | GO:0046983~protein dimerization activity | 0.018413 |
|  | GOTERM_MF_5 | GO:0042162~telomeric DNA binding | 0.019418 |
|  | GOTERM_BP_5 | GO:0006297~nucleotide-excision repair, DNA gap filling | 0.020557 |
|  | GOTERM_CC_5 | GO:0043073~germ cell nucleus | 0.020604 |
|  | GOTERM_BP_5 | GO:0015074~DNA integration | 0.024144 |
|  | GOTERM_BP_3 | GO:0019059~initiation of viral infection | 0.024246 |
|  | GOTERM_BP_4 | GO:0019059~initiation of viral infection | 0.024772 |
|  | GOTERM_BP_3 | GO:0002562~somatic diversification of immune receptors via germline recombination within a single locus | 0.026422 |
|  | GOTERM_BP_5 | GO:0019059~initiation of viral infection | 0.026528 |
|  | GOTERM_BP_4 | GO:0002562~somatic diversification of immune receptors via germline recombination within a single locus | 0.026995 |
|  | GOTERM_MF_3 | GO:0042802~identical protein binding | 0.028502 |
|  | GOTERM_BP_2 | GO:0002200~somatic diversification of immune receptors | 0.028667 |
|  | GOTERM_BP_3 | GO:0002200~somatic diversification of immune receptors | 0.029679 |
|  | GOTERM_BP_4 | GO:0045935~positive regulation of nucleobase, nucleoside, nucleotide and nucleic acid metabolic process | 0.031432 |
|  | GOTERM_BP_3 | GO:0051173~positive regulation of nitrogen compound metabolic process | 0.03224 |
|  | GOTERM_BP_5 | GO:0002200~somatic diversification of immune receptors | 0.032465 |
|  | GOTERM_BP_4 | GO:0051173~positive regulation of nitrogen compound metabolic process | 0.034085 |
|  | GOTERM_BP_5 | GO:0045935~positive regulation of nucleobase, nucleoside, nucleotide and nucleic acid metabolic process | 0.037526 |
|  | GOTERM_BP_5 | GO:0051173~positive regulation of nitrogen compound metabolic process | 0.040653 |
|  | GOTERM_MF_2 | GO:0005515~protein binding | 0.042356 |
|  | GOTERM_MF_5 | GO:0004536~deoxyribonuclease activity | 0.043514 |
|  | GOTERM_BP_4 | GO:0051053~negative regulation of DNA metabolic process | 0.04352 |
|  | GOTERM_BP_4 | GO:0044265~cellular macromolecule catabolic process | 0.046018 |
|  | GOTERM_BP_5 | GO:0051053~negative regulation of DNA metabolic process | 0.046575 |
|  | GOTERM_BP_3 | GO:0019058~viral infectious cycle | 0.046876 |
|  | GOTERM_BP_4 | GO:0019058~viral infectious cycle | 0.047882 |
| 5699 | GOTERM_CC_4 | GO:0005886~plasma membrane | 1.61E-04 |
|  | GOTERM_BP_3 | GO:0006952~defense response | 1.85E-04 |
|  | GOTERM_CC_4 | GO:0044459~plasma membrane part | 3.22E-04 |
|  | GOTERM_CC_5 | GO:0044459~plasma membrane part | 3.58E-04 |
|  | GOTERM_BP_3 | GO:0006810~transport | 0.001048 |
|  | GOTERM_CC_5 | GO:0005887~integral to plasma membrane | 0.001138 |
|  | GOTERM_CC_4 | GO:0031226~intrinsic to plasma membrane | 0.001169 |
|  | GOTERM_CC_5 | GO:0031226~intrinsic to plasma membrane | 0.00126 |
|  | GOTERM_BP_1 | GO:0051179~localization | 0.001263 |
|  | GOTERM_BP_5 | GO:0019724~B cell mediated immunity | 0.001307 |
|  | GOTERM_CC_3 | GO:0044459~plasma membrane part | 0.00173 |
|  | GOTERM_CC_3 | GO:0005886~plasma membrane | 0.001965 |
|  | GOTERM_BP_5 | GO:0002449~lymphocyte mediated immunity | 0.002034 |
|  | GOTERM_CC_2 | GO:0000267~cell fraction | 0.002057 |
|  | GOTERM_CC_3 | GO:0000267~cell fraction | 0.002064 |
|  | GOTERM_BP_4 | GO:0002449~lymphocyte mediated immunity | 0.00209 |
|  | GOTERM_CC_4 | GO:0005624~membrane fraction | 0.002218 |
|  | GOTERM_CC_5 | GO:0005624~membrane fraction | 0.002358 |
|  | GOTERM_BP_3 | GO:0002250~adaptive immune response | 0.002418 |
|  | GOTERM_BP_4 | GO:0002460~adaptive immune response based on somatic recombination of immune receptors built from immunoglobulin superfamily domains | 0.002523 |
|  | GOTERM_CC_4 | GO:0005626~insoluble fraction | 0.002537 |
|  | GOTERM_BP_2 | GO:0006810~transport | 0.00286 |
|  | GOTERM_BP_1 | GO:0051234~establishment of localization | 0.002911 |
|  | GOTERM_BP_3 | GO:0002443~leukocyte mediated immunity | 0.003005 |
|  | GOTERM_BP_2 | GO:0051234~establishment of localization | 0.00306 |
|  | GOTERM_BP_4 | GO:0002443~leukocyte mediated immunity | 0.003135 |
|  | GOTERM_BP_4 | GO:0006954~inflammatory response | 0.00354 |
|  | GOTERM_BP_5 | GO:0002526~acute inflammatory response | 0.003942 |
|  | GOTERM_BP_1 | GO:0050896~response to stimulus | 0.004081 |
|  | GOTERM_BP_2 | GO:0006955~immune response | 0.005237 |
|  | GOTERM_CC_3 | GO:0005626~insoluble fraction | 0.005798 |
|  | GOTERM_BP_2 | GO:0006950~response to stress | 0.005856 |
|  | GOTERM_BP_3 | GO:0002252~immune effector process | 0.007138 |
|  | GOTERM_BP_2 | GO:0002252~immune effector process | 0.008867 |
|  | GOTERM_MF_5 | GO:0005509~calcium ion binding | 0.009614 |
|  | GOTERM_MF_2 | GO:0005515~protein binding | 0.010625 |
|  | GOTERM_BP_5 | GO:0015674~di-, tri-valent inorganic cation transport | 0.012244 |
|  | GOTERM_BP_3 | GO:0009611~response to wounding | 0.012916 |
|  | GOTERM_BP_5 | GO:0006812~cation transport | 0.014698 |
|  | GOTERM_BP_4 | GO:0006812~cation transport | 0.015356 |
|  | GOTERM_MF_5 | GO:0008324~cation transmembrane transporter activity | 0.015544 |
|  | GOTERM_BP_3 | GO:0055085~transmembrane transport | 0.015642 |
|  | GOTERM_BP_4 | GO:0055085~transmembrane transport | 0.016577 |
|  | GOTERM_BP_1 | GO:0002376~immune system process | 0.01835 |
|  | GOTERM_BP_3 | GO:0032879~regulation of localization | 0.018839 |
|  | GOTERM_BP_3 | GO:0050776~regulation of immune response | 0.019373 |
|  | GOTERM_BP_4 | GO:0050776~regulation of immune response | 0.020179 |
|  | GOTERM_BP_2 | GO:0055085~transmembrane transport | 0.021312 |
|  | GOTERM_CC_2 | GO:0005615~extracellular space | 0.022499 |
|  | GOTERM_CC_3 | GO:0005615~extracellular space | 0.022542 |
|  | GOTERM_MF_2 | GO:0043178~alcohol binding | 0.02501 |
|  | GOTERM_BP_2 | GO:0032879~regulation of localization | 0.025578 |
|  | GOTERM_BP_5 | GO:0006958~complement activation, classical pathway | 0.027959 |
|  | GOTERM_BP_4 | GO:0006958~complement activation, classical pathway | 0.028255 |
|  | GOTERM_BP_4 | GO:0002455~humoral immune response mediated by circulating immunoglobulin | 0.030176 |
|  | GOTERM_CC_1 | GO:0005576~extracellular region | 0.032325 |
|  | GOTERM_MF_4 | GO:0015075~ion transmembrane transporter activity | 0.033684 |
|  | GOTERM_BP_3 | GO:0006811~ion transport | 0.034422 |
|  | GOTERM_BP_4 | GO:0006811~ion transport | 0.036391 |
|  | GOTERM_MF_3 | GO:0004872~receptor activity | 0.036678 |
|  | GOTERM_MF_5 | GO:0022836~gated channel activity | 0.036854 |
|  | GOTERM_BP_3 | GO:0006956~complement activation | 0.039823 |
|  | GOTERM_BP_5 | GO:0006956~complement activation | 0.040258 |
|  | GOTERM_BP_4 | GO:0006956~complement activation | 0.04068 |
|  | GOTERM_MF_3 | GO:0019904~protein domain specific binding | 0.047733 |
| 5822 | GOTERM_CC_3 | GO:0044459~plasma membrane part | 7.61E-04 |
|  | GOTERM_CC_4 | GO:0044459~plasma membrane part | 0.001082 |
|  | GOTERM_CC_5 | GO:0044459~plasma membrane part | 0.001245 |
|  | GOTERM_BP_3 | GO:0002523~leukocyte migration during inflammatory response | 0.003414 |
|  | GOTERM_BP_5 | GO:0002523~leukocyte migration during inflammatory response | 0.003577 |
|  | GOTERM_CC_3 | GO:0031253~cell projection membrane | 0.005838 |
|  | GOTERM_CC_4 | GO:0031253~cell projection membrane | 0.006319 |
|  | GOTERM_CC_5 | GO:0031253~cell projection membrane | 0.006524 |
|  | GOTERM_CC_2 | GO:0042995~cell projection | 0.006788 |
|  | GOTERM_CC_3 | GO:0042995~cell projection | 0.006808 |
|  | GOTERM_CC_4 | GO:0031225~anchored to membrane | 0.007465 |
|  | GOTERM_BP_4 | GO:0050727~regulation of inflammatory response | 0.007683 |
|  | GOTERM_CC_5 | GO:0031225~anchored to membrane | 0.007811 |
|  | GOTERM_BP_5 | GO:0050727~regulation of inflammatory response | 0.008044 |
|  | GOTERM_BP_3 | GO:0016337~cell-cell adhesion | 0.011143 |
|  | GOTERM_CC_3 | GO:0005626~insoluble fraction | 0.014513 |
|  | GOTERM_BP_4 | GO:0007268~synaptic transmission | 0.014516 |
|  | GOTERM_CC_4 | GO:0005624~membrane fraction | 0.014781 |
|  | GOTERM_BP_5 | GO:0007268~synaptic transmission | 0.015443 |
|  | GOTERM_CC_5 | GO:0005624~membrane fraction | 0.015787 |
|  | GOTERM_CC_4 | GO:0005626~insoluble fraction | 0.017079 |
|  | GOTERM_BP_3 | GO:0007267~cell-cell signaling | 0.017605 |
|  | GOTERM_BP_3 | GO:0031323~regulation of cellular metabolic process | 0.01835 |
|  | GOTERM_CC_3 | GO:0005886~plasma membrane | 0.019691 |
|  | GOTERM_CC_4 | GO:0031226~intrinsic to plasma membrane | 0.020831 |
|  | GOTERM_BP_4 | GO:0031323~regulation of cellular metabolic process | 0.021544 |
|  | GOTERM_BP_4 | GO:0019226~transmission of nerve impulse | 0.022207 |
|  | GOTERM_CC_5 | GO:0031226~intrinsic to plasma membrane | 0.022425 |
|  | GOTERM_CC_2 | GO:0045211~postsynaptic membrane | 0.023503 |
|  | GOTERM_CC_3 | GO:0045211~postsynaptic membrane | 0.023534 |
|  | GOTERM_CC_4 | GO:0045211~postsynaptic membrane | 0.025396 |
|  | GOTERM_BP_3 | GO:0019222~regulation of metabolic process | 0.025416 |
|  | GOTERM_BP_4 | GO:0031347~regulation of defense response | 0.025502 |
|  | GOTERM_BP_5 | GO:0031347~regulation of defense response | 0.026664 |
|  | GOTERM_CC_4 | GO:0005886~plasma membrane | 0.026957 |
|  | GOTERM_BP_2 | GO:0019222~regulation of metabolic process | 0.027928 |
|  | GOTERM_BP_1 | GO:0022610~biological adhesion | 0.029448 |
|  | GOTERM_BP_3 | GO:0032101~regulation of response to external stimulus | 0.029813 |
|  | GOTERM_BP_2 | GO:0007155~cell adhesion | 0.030032 |
|  | GOTERM_BP_4 | GO:0032101~regulation of response to external stimulus | 0.031025 |
|  | GOTERM_BP_3 | GO:0060255~regulation of macromolecule metabolic process | 0.033029 |
|  | GOTERM_BP_4 | GO:0007166~cell surface receptor linked signal transduction | 0.03419 |
|  | GOTERM_MF_1 | GO:0060089~molecular transducer activity | 0.036697 |
|  | GOTERM_BP_4 | GO:0007157~heterophilic cell adhesion | 0.037743 |
|  | GOTERM_BP_5 | GO:0007166~cell surface receptor linked signal transduction | 0.037892 |
|  | GOTERM_MF_5 | GO:0016917~GABA receptor activity | 0.037901 |
|  | GOTERM_BP_4 | GO:0060255~regulation of macromolecule metabolic process | 0.037996 |
|  | GOTERM_CC_2 | GO:0000267~cell fraction | 0.038643 |
|  | GOTERM_CC_3 | GO:0000267~cell fraction | 0.038746 |
|  | GOTERM_CC_3 | GO:0031256~leading edge membrane | 0.039882 |
|  | GOTERM_CC_5 | GO:0046658~anchored to plasma membrane | 0.040403 |
|  | GOTERM_MF_2 | GO:0004871~signal transducer activity | 0.040557 |
|  | GOTERM_CC_4 | GO:0031256~leading edge membrane | 0.041521 |
|  | GOTERM_MF_3 | GO:0008134~transcription factor binding | 0.041862 |
|  | GOTERM_CC_5 | GO:0031256~leading edge membrane | 0.042201 |
|  | GOTERM_BP_2 | GO:0007154~cell communication | 0.044834 |
|  | GOTERM_BP_4 | GO:0007159~leukocyte adhesion | 0.047797 |
|  | GOTERM_BP_3 | GO:0009653~anatomical structure morphogenesis | 0.048063 |
|  | GOTERM_BP_2 | GO:0009653~anatomical structure morphogenesis | 0.049746 |
| 5931 | GOTERM_BP_5 | GO:0010959~regulation of metal ion transport | 9.52E-05 |
|  | GOTERM_CC_3 | GO:0005886~plasma membrane | 1.13E-04 |
|  | GOTERM_CC_3 | GO:0044459~plasma membrane part | 1.20E-04 |
|  | GOTERM_BP_4 | GO:0043269~regulation of ion transport | 1.41E-04 |
|  | GOTERM_CC_4 | GO:0044459~plasma membrane part | 1.62E-04 |
|  | GOTERM_CC_4 | GO:0005886~plasma membrane | 1.64E-04 |
|  | GOTERM_BP_5 | GO:0043269~regulation of ion transport | 1.73E-04 |
|  | GOTERM_CC_5 | GO:0044459~plasma membrane part | 1.82E-04 |
|  | GOTERM_BP_3 | GO:0035150~regulation of tube size | 0.001342 |
|  | GOTERM_BP_4 | GO:0050880~regulation of blood vessel size | 0.001401 |
|  | GOTERM_CC_4 | GO:0042383~sarcolemma | 0.001552 |
|  | GOTERM_CC_5 | GO:0042383~sarcolemma | 0.001603 |
|  | GOTERM_BP_5 | GO:0050880~regulation of blood vessel size | 0.001606 |
|  | GOTERM_BP_4 | GO:0003018~vascular process in circulatory system | 0.001676 |
|  | GOTERM_MF_5 | GO:0004962~endothelin receptor activity | 0.002008 |
|  | GOTERM_BP_5 | GO:0019932~second-messenger-mediated signaling | 0.002149 |
|  | GOTERM_MF_4 | GO:0034617~tetrahydrobiopterin binding | 0.002801 |
|  | GOTERM_MF_4 | GO:0034618~arginine binding | 0.002801 |
|  | GOTERM_MF_5 | GO:0004517~nitric-oxide synthase activity | 0.00301 |
|  | GOTERM_BP_3 | GO:0044057~regulation of system process | 0.003612 |
|  | GOTERM_BP_4 | GO:0044057~regulation of system process | 0.003839 |
|  | GOTERM_BP_3 | GO:0044092~negative regulation of molecular function | 0.004495 |
|  | GOTERM_BP_4 | GO:0031279~regulation of cyclase activity | 0.004797 |
|  | GOTERM_BP_4 | GO:0051339~regulation of lyase activity | 0.004987 |
|  | GOTERM_BP_5 | GO:0030808~regulation of nucleotide biosynthetic process | 0.006732 |
|  | GOTERM_BP_5 | GO:0006140~regulation of nucleotide metabolic process | 0.007461 |
|  | GOTERM_BP_3 | GO:0001666~response to hypoxia | 0.008274 |
|  | GOTERM_BP_4 | GO:0001666~response to hypoxia | 0.008626 |
|  | GOTERM_BP_5 | GO:0055117~regulation of cardiac muscle contraction | 0.009078 |
|  | GOTERM_BP_3 | GO:0070482~response to oxygen levels | 0.009126 |
|  | GOTERM_BP_3 | GO:0050790~regulation of catalytic activity | 0.009327 |
|  | GOTERM_BP_3 | GO:0051049~regulation of transport | 0.00935 |
|  | GOTERM_MF_3 | GO:0005516~calmodulin binding | 0.009555 |
|  | GOTERM_BP_4 | GO:0051049~regulation of transport | 0.00992 |
|  | GOTERM_BP_4 | GO:0043502~regulation of muscle adaptation | 0.01058 |
|  | GOTERM_MF_4 | GO:0010181~FMN binding | 0.01116 |
|  | GOTERM_BP_5 | GO:0043502~regulation of muscle adaptation | 0.011335 |
|  | GOTERM_MF_5 | GO:0010181~FMN binding | 0.011991 |
|  | GOTERM_BP_3 | GO:0045909~positive regulation of vasodilation | 0.012413 |
|  | GOTERM_BP_2 | GO:0003008~system process | 0.012569 |
|  | GOTERM_BP_4 | GO:0006809~nitric oxide biosynthetic process | 0.012683 |
|  | GOTERM_BP_4 | GO:0051926~negative regulation of calcium ion transport | 0.012683 |
|  | GOTERM_BP_4 | GO:0045909~positive regulation of vasodilation | 0.012683 |
|  | GOTERM_BP_3 | GO:0003012~muscle system process | 0.012765 |
|  | GOTERM_MF_3 | GO:0010181~FMN binding | 0.012802 |
|  | GOTERM_BP_2 | GO:0065009~regulation of molecular function | 0.013212 |
|  | GOTERM_BP_4 | GO:0051174~regulation of phosphorus metabolic process | 0.013332 |
|  | GOTERM_BP_3 | GO:0046209~nitric oxide metabolic process | 0.01344 |
|  | GOTERM_BP_5 | GO:0051926~negative regulation of calcium ion transport | 0.013588 |
|  | GOTERM_BP_5 | GO:0045909~positive regulation of vasodilation | 0.013588 |
|  | GOTERM_BP_5 | GO:0006809~nitric oxide biosynthetic process | 0.013588 |
|  | GOTERM_BP_3 | GO:0003013~circulatory system process | 0.01549 |
|  | GOTERM_BP_5 | GO:0019220~regulation of phosphate metabolic process | 0.016058 |
|  | GOTERM_BP_5 | GO:0051174~regulation of phosphorus metabolic process | 0.016058 |
|  | GOTERM_BP_4 | GO:0043085~positive regulation of catalytic activity | 0.016079 |
|  | GOTERM_BP_4 | GO:0008015~blood circulation | 0.016139 |
|  | GOTERM_CC_4 | GO:0030315~T-tubule | 0.017589 |
|  | GOTERM_CC_5 | GO:0030315~T-tubule | 0.01788 |
|  | GOTERM_CC_5 | GO:0005887~integral to plasma membrane | 0.01789 |
|  | GOTERM_CC_4 | GO:0031226~intrinsic to plasma membrane | 0.018252 |
|  | GOTERM_BP_2 | GO:0048523~negative regulation of cellular process | 0.018878 |
|  | GOTERM_BP_5 | GO:0042310~vasoconstriction | 0.019199 |
|  | GOTERM_CC_5 | GO:0031226~intrinsic to plasma membrane | 0.019302 |
|  | GOTERM_BP_3 | GO:0044093~positive regulation of molecular function | 0.020857 |
|  | GOTERM_BP_4 | GO:0042312~regulation of vasodilation | 0.021056 |
|  | GOTERM_BP_2 | GO:0032879~regulation of localization | 0.021135 |
|  | GOTERM_BP_3 | GO:0048523~negative regulation of cellular process | 0.021748 |
|  | GOTERM_BP_5 | GO:0042312~regulation of vasodilation | 0.022551 |
|  | GOTERM_BP_3 | GO:0032879~regulation of localization | 0.023185 |
|  | GOTERM_BP_4 | GO:0006520~cellular amino acid metabolic process | 0.023279 |
|  | GOTERM_BP_3 | GO:0043271~negative regulation of ion transport | 0.023665 |
|  | GOTERM_BP_4 | GO:0048523~negative regulation of cellular process | 0.023716 |
|  | GOTERM_BP_4 | GO:0043271~negative regulation of ion transport | 0.024178 |
|  | GOTERM_BP_5 | GO:0006942~regulation of striated muscle contraction | 0.02478 |
|  | GOTERM_MF_4 | GO:0016709~oxidoreductase activity, acting on paired donors, with incorporation or reduction of molecular oxygen, NADH or NADPH as one donor, and incorporation of one atom of oxygen | 0.025864 |
|  | GOTERM_BP_5 | GO:0043271~negative regulation of ion transport | 0.025893 |
|  | GOTERM_BP_5 | GO:0006520~cellular amino acid metabolic process | 0.026481 |
|  | GOTERM_BP_2 | GO:0048519~negative regulation of biological process | 0.026795 |
|  | GOTERM_CC_3 | GO:0005625~soluble fraction | 0.028099 |
|  | GOTERM_BP_3 | GO:0010522~regulation of calcium ion transport into cytosol | 0.029752 |
|  | GOTERM_CC_4 | GO:0005625~soluble fraction | 0.030316 |
|  | GOTERM_BP_4 | GO:0010522~regulation of calcium ion transport into cytosol | 0.030396 |
|  | GOTERM_BP_3 | GO:0048519~negative regulation of biological process | 0.030764 |
|  | GOTERM_CC_2 | GO:0016020~membrane | 0.031122 |
|  | GOTERM_CC_3 | GO:0016020~membrane | 0.031297 |
|  | GOTERM_BP_5 | GO:0010522~regulation of calcium ion transport into cytosol | 0.032545 |
|  | GOTERM_BP_5 | GO:0030003~cellular cation homeostasis | 0.032864 |
|  | GOTERM_BP_5 | GO:0007186~G-protein coupled receptor protein signaling pathway | 0.03296 |
|  | GOTERM_MF_4 | GO:0050661~NADP or NADPH binding | 0.033141 |
|  | GOTERM_BP_4 | GO:0043086~negative regulation of catalytic activity | 0.033938 |
|  | GOTERM_BP_1 | GO:0032501~multicellular organismal process | 0.034341 |
|  | GOTERM_BP_4 | GO:0007166~cell surface receptor linked signal transduction | 0.03673 |
|  | GOTERM_BP_5 | GO:0006939~smooth muscle contraction | 0.036956 |
|  | GOTERM_BP_5 | GO:0014032~neural crest cell development | 0.036956 |
|  | GOTERM_BP_5 | GO:0014033~neural crest cell differentiation | 0.036956 |
|  | GOTERM_MF_3 | GO:0050661~NADP or NADPH binding | 0.037951 |
|  | GOTERM_BP_2 | GO:0033555~multicellular organismal response to stress | 0.038468 |
|  | GOTERM_BP_4 | GO:0007268~synaptic transmission | 0.038793 |
|  | GOTERM_BP_3 | GO:0033555~multicellular organismal response to stress | 0.03982 |
|  | GOTERM_BP_4 | GO:0044106~cellular amine metabolic process | 0.040469 |
|  | GOTERM_BP_2 | GO:0048518~positive regulation of biological process | 0.041879 |
|  | GOTERM_BP_5 | GO:0007268~synaptic transmission | 0.043993 |
|  | GOTERM_CC_4 | GO:0005901~caveola | 0.045137 |
|  | GOTERM_BP_4 | GO:0044271~nitrogen compound biosynthetic process | 0.045409 |
|  | GOTERM_CC_5 | GO:0005901~caveola | 0.045875 |
|  | GOTERM_BP_5 | GO:0044106~cellular amine metabolic process | 0.04588 |
|  | GOTERM_BP_2 | GO:0048646~anatomical structure formation involved in morphogenesis | 0.047332 |
|  | GOTERM_BP_5 | GO:0007166~cell surface receptor linked signal transduction | 0.047816 |
|  | GOTERM_BP_3 | GO:0048518~positive regulation of biological process | 0.047841 |
|  | GOTERM_BP_4 | GO:0051336~regulation of hydrolase activity | 0.048477 |
| 5949 | GOTERM_CC_4 | GO:0044459~plasma membrane part | 5.46E-12 |
|  | GOTERM_CC_5 | GO:0044459~plasma membrane part | 8.13E-12 |
|  | GOTERM_CC_3 | GO:0044459~plasma membrane part | 8.60E-12 |
|  | GOTERM_CC_5 | GO:0043296~apical junction complex | 1.68E-11 |
|  | GOTERM_CC_4 | GO:0016327~apicolateral plasma membrane | 1.91E-11 |
|  | GOTERM_CC_5 | GO:0016327~apicolateral plasma membrane | 2.21E-11 |
|  | GOTERM_CC_4 | GO:0030054~cell junction | 2.97E-09 |
|  | GOTERM_CC_5 | GO:0030054~cell junction | 3.61E-09 |
|  | GOTERM_CC_5 | GO:0005911~cell-cell junction | 5.80E-09 |
|  | GOTERM_CC_4 | GO:0005886~plasma membrane | 2.64E-07 |
|  | GOTERM_CC_3 | GO:0005886~plasma membrane | 3.85E-07 |
|  | GOTERM_CC_4 | GO:0045211~postsynaptic membrane | 5.57E-05 |
|  | GOTERM_CC_2 | GO:0045211~postsynaptic membrane | 5.67E-05 |
|  | GOTERM_CC_3 | GO:0045211~postsynaptic membrane | 5.69E-05 |
|  | GOTERM_CC_1 | GO:0045202~synapse | 7.58E-05 |
|  | GOTERM_MF_2 | GO:0005515~protein binding | 4.14E-04 |
|  | GOTERM_CC_1 | GO:0044456~synapse part | 7.67E-04 |
|  | GOTERM_CC_2 | GO:0044456~synapse part | 9.14E-04 |
|  | GOTERM_MF_5 | GO:0019198~transmembrane receptor protein phosphatase activity | 0.001135 |
|  | GOTERM_CC_5 | GO:0019717~synaptosome | 0.002343 |
|  | GOTERM_CC_4 | GO:0044425~membrane part | 0.002571 |
|  | GOTERM_CC_5 | GO:0005887~integral to plasma membrane | 0.003064 |
|  | GOTERM_CC_2 | GO:0044425~membrane part | 0.003196 |
|  | GOTERM_CC_4 | GO:0031226~intrinsic to plasma membrane | 0.0032 |
|  | GOTERM_CC_3 | GO:0044425~membrane part | 0.003239 |
|  | GOTERM_CC_2 | GO:0016020~membrane | 0.00349 |
|  | GOTERM_CC_3 | GO:0016020~membrane | 0.00354 |
|  | GOTERM_MF_3 | GO:0004872~receptor activity | 0.003583 |
|  | GOTERM_CC_5 | GO:0031226~intrinsic to plasma membrane | 0.003607 |
|  | GOTERM_BP_4 | GO:0016192~vesicle-mediated transport | 0.004326 |
|  | GOTERM_BP_3 | GO:0016192~vesicle-mediated transport | 0.00448 |
|  | GOTERM_BP_2 | GO:0016192~vesicle-mediated transport | 0.004939 |
|  | GOTERM_BP_5 | GO:0016311~dephosphorylation | 0.00799 |
|  | GOTERM_BP_5 | GO:0007264~small GTPase mediated signal transduction | 0.008627 |
|  | GOTERM_BP_3 | GO:0008104~protein localization | 0.009233 |
|  | GOTERM_MF_3 | GO:0016788~hydrolase activity, acting on ester bonds | 0.012716 |
|  | GOTERM_MF_3 | GO:0019904~protein domain specific binding | 0.013883 |
|  | GOTERM_BP_5 | GO:0015031~protein transport | 0.014626 |
|  | GOTERM_MF_2 | GO:0004871~signal transducer activity | 0.014808 |
|  | GOTERM_CC_4 | GO:0009898~internal side of plasma membrane | 0.015739 |
|  | GOTERM_BP_5 | GO:0007242~intracellular signaling cascade | 0.015789 |
|  | GOTERM_BP_4 | GO:0015031~protein transport | 0.016157 |
|  | GOTERM_CC_5 | GO:0009898~internal side of plasma membrane | 0.016625 |
|  | GOTERM_BP_3 | GO:0015031~protein transport | 0.016669 |
|  | GOTERM_BP_4 | GO:0045184~establishment of protein localization | 0.016836 |
|  | GOTERM_CC_4 | GO:0019898~extrinsic to membrane | 0.017124 |
|  | GOTERM_BP_3 | GO:0045184~establishment of protein localization | 0.017368 |
|  | GOTERM_CC_3 | GO:0019898~extrinsic to membrane | 0.017371 |
|  | GOTERM_BP_4 | GO:0007242~intracellular signaling cascade | 0.017977 |
|  | GOTERM_MF_1 | GO:0060089~molecular transducer activity | 0.018139 |
|  | GOTERM_CC_5 | GO:0019898~extrinsic to membrane | 0.018263 |
|  | GOTERM_BP_2 | GO:0045184~establishment of protein localization | 0.018964 |
|  | GOTERM_BP_2 | GO:0007154~cell communication | 0.021966 |
|  | GOTERM_BP_3 | GO:0007267~cell-cell signaling | 0.023175 |
|  | GOTERM_CC_5 | GO:0017146~N-methyl-D-aspartate selective glutamate receptor complex | 0.024688 |
|  | GOTERM_BP_2 | GO:0033036~macromolecule localization | 0.027836 |
|  | GOTERM_MF_1 | GO:0005488~binding | 0.028181 |
|  | GOTERM_MF_5 | GO:0004385~guanylate kinase activity | 0.030514 |
|  | GOTERM_MF_5 | GO:0016791~phosphatase activity | 0.03193 |
|  | GOTERM_MF_4 | GO:0016594~glycine binding | 0.033138 |
|  | GOTERM_MF_2 | GO:0016787~hydrolase activity | 0.035928 |
|  | GOTERM_CC_4 | GO:0005624~membrane fraction | 0.03595 |
|  | GOTERM_CC_1 | GO:0044464~cell part | 0.036311 |
|  | GOTERM_CC_1 | GO:0005623~cell | 0.036426 |
|  | GOTERM_CC_5 | GO:0005624~membrane fraction | 0.038511 |
|  | GOTERM_CC_4 | GO:0005626~insoluble fraction | 0.041801 |
|  | GOTERM_CC_4 | GO:0008328~ionotropic glutamate receptor complex | 0.042123 |
|  | GOTERM_CC_3 | GO:0005626~insoluble fraction | 0.042438 |
|  | GOTERM_BP_2 | GO:0051641~cellular localization | 0.042447 |
|  | GOTERM_CC_5 | GO:0008328~ionotropic glutamate receptor complex | 0.042813 |
|  | GOTERM_MF_2 | GO:0043176~amine binding | 0.043919 |
|  | GOTERM_BP_5 | GO:0007268~synaptic transmission | 0.045066 |
|  | GOTERM_BP_4 | GO:0007268~synaptic transmission | 0.047264 |
| 5975 | GOTERM_BP_2 | GO:0048856~anatomical structure development | 0.00188 |
|  | GOTERM_BP_1 | GO:0032502~developmental process | 0.002567 |
|  | GOTERM_BP_5 | GO:0006457~protein folding | 0.003226 |
|  | GOTERM_MF_3 | GO:0046983~protein dimerization activity | 0.004546 |
|  | GOTERM_BP_5 | GO:0001757~somite specification | 0.006817 |
|  | GOTERM_MF_1 | GO:0005488~binding | 0.015634 |
|  | GOTERM_CC_3 | GO:0005833~hemoglobin complex | 0.016371 |
|  | GOTERM_BP_2 | GO:0007275~multicellular organismal development | 0.017054 |
|  | GOTERM_CC_5 | GO:0005833~hemoglobin complex | 0.017335 |
|  | GOTERM_BP_4 | GO:0007379~segment specification | 0.017402 |
|  | GOTERM_BP_5 | GO:0007379~segment specification | 0.018641 |
|  | GOTERM_BP_3 | GO:0048731~system development | 0.019266 |
|  | GOTERM_BP_3 | GO:0010817~regulation of hormone levels | 0.022825 |
|  | GOTERM_CC_2 | GO:0044424~intracellular part | 0.024086 |
|  | GOTERM_CC_3 | GO:0044424~intracellular part | 0.02435 |
|  | GOTERM_BP_1 | GO:0032501~multicellular organismal process | 0.029405 |
|  | GOTERM_BP_2 | GO:0009653~anatomical structure morphogenesis | 0.02945 |
|  | GOTERM_BP_3 | GO:0009653~anatomical structure morphogenesis | 0.03366 |
|  | GOTERM_CC_4 | GO:0005829~cytosol | 0.036255 |
|  | GOTERM_MF_4 | GO:0003700~transcription factor activity | 0.03805 |
|  | GOTERM_CC_5 | GO:0005829~cytosol | 0.038572 |
|  | GOTERM_CC_2 | GO:0005622~intracellular | 0.040291 |
|  | GOTERM_CC_3 | GO:0005622~intracellular | 0.040722 |
|  | GOTERM_BP_3 | GO:0048513~organ development | 0.044766 |
|  | GOTERM_CC_4 | GO:0044424~intracellular part | 0.044971 |
|  | GOTERM_MF_4 | GO:0043565~sequence-specific DNA binding | 0.04625 |
|  | GOTERM_BP_3 | GO:0009880~embryonic pattern specification | 0.048781 |
|  | GOTERM_BP_4 | GO:0048513~organ development | 0.049043 |
|  | GOTERM_BP_4 | GO:0009880~embryonic pattern specification | 0.049826 |
| 5978 | GOTERM_CC_2 | GO:0044424~intracellular part | 0.004219 |
|  | GOTERM_CC_3 | GO:0044424~intracellular part | 0.004265 |
|  | GOTERM_CC_3 | GO:0043231~intracellular membrane-bounded organelle | 0.006 |
|  | GOTERM_CC_2 | GO:0043227~membrane-bounded organelle | 0.00601 |
|  | GOTERM_CC_2 | GO:0005622~intracellular | 0.007062 |
|  | GOTERM_CC_3 | GO:0005622~intracellular | 0.007139 |
|  | GOTERM_CC_4 | GO:0044424~intracellular part | 0.007899 |
|  | GOTERM_CC_4 | GO:0043231~intracellular membrane-bounded organelle | 0.009451 |
|  | GOTERM_CC_5 | GO:0043231~intracellular membrane-bounded organelle | 0.011332 |
|  | GOTERM_CC_4 | GO:0016581~NuRD complex | 0.013218 |
|  | GOTERM_CC_5 | GO:0016581~NuRD complex | 0.013438 |
|  | GOTERM_BP_5 | GO:0006260~DNA replication | 0.014167 |
|  | GOTERM_CC_1 | GO:0043226~organelle | 0.014256 |
|  | GOTERM_BP_3 | GO:0007030~Golgi organization | 0.020098 |
|  | GOTERM_CC_2 | GO:0043229~intracellular organelle | 0.021443 |
|  | GOTERM_CC_3 | GO:0043229~intracellular organelle | 0.021608 |
|  | GOTERM_MF_4 | GO:0016836~hydro-lyase activity | 0.033149 |
|  | GOTERM_CC_4 | GO:0043229~intracellular organelle | 0.033298 |
|  | GOTERM_BP_4 | GO:0044271~nitrogen compound biosynthetic process | 0.034013 |
|  | GOTERM_BP_2 | GO:0006807~nitrogen compound metabolic process | 0.037085 |
|  | GOTERM_BP_3 | GO:0034641~cellular nitrogen compound metabolic process | 0.038292 |
|  | GOTERM_CC_4 | GO:0005634~nucleus | 0.039231 |
|  | GOTERM_CC_3 | GO:0017053~transcriptional repressor complex | 0.039512 |
|  | GOTERM_CC_5 | GO:0043229~intracellular organelle | 0.039536 |
|  | GOTERM_CC_4 | GO:0000118~histone deacetylase complex | 0.041137 |
|  | GOTERM_CC_5 | GO:0000118~histone deacetylase complex | 0.041811 |
|  | GOTERM_CC_5 | GO:0017053~transcriptional repressor complex | 0.041811 |
|  | GOTERM_CC_5 | GO:0005634~nucleus | 0.04359 |
|  | GOTERM_MF_3 | GO:0016835~carbon-oxygen lyase activity | 0.045409 |
|  | GOTERM_BP_5 | GO:0008652~cellular amino acid biosynthetic process | 0.048689 |
| 5982 | GOTERM_MF_2 | GO:0005515~protein binding | 1.01E-06 |
|  | GOTERM_BP_4 | GO:0007398~ectoderm development | 1.04E-05 |
|  | GOTERM_BP_5 | GO:0007398~ectoderm development | 1.07E-05 |
|  | GOTERM_CC_4 | GO:0005882~intermediate filament | 5.12E-05 |
|  | GOTERM_CC_5 | GO:0005882~intermediate filament | 5.53E-05 |
|  | GOTERM_CC_5 | GO:0045111~intermediate filament cytoskeleton | 6.13E-05 |
|  | GOTERM_BP_2 | GO:0007049~cell cycle | 9.31E-05 |
|  | GOTERM_BP_4 | GO:0008544~epidermis development | 1.01E-04 |
|  | GOTERM_BP_5 | GO:0008544~epidermis development | 1.03E-04 |
|  | GOTERM_BP_3 | GO:0009888~tissue development | 1.97E-04 |
|  | GOTERM_BP_4 | GO:0009888~tissue development | 2.28E-04 |
|  | GOTERM_BP_5 | GO:0009888~tissue development | 2.32E-04 |
|  | GOTERM_CC_1 | GO:0044422~organelle part | 3.02E-04 |
|  | GOTERM_BP_4 | GO:0000079~regulation of cyclin-dependent protein kinase activity | 3.56E-04 |
|  | GOTERM_BP_5 | GO:0000079~regulation of cyclin-dependent protein kinase activity | 3.63E-04 |
|  | GOTERM_CC_2 | GO:0044446~intracellular organelle part | 4.79E-04 |
|  | GOTERM_CC_3 | GO:0044446~intracellular organelle part | 4.84E-04 |
|  | GOTERM_CC_4 | GO:0044446~intracellular organelle part | 5.11E-04 |
|  | GOTERM_CC_2 | GO:0044422~organelle part | 5.22E-04 |
|  | GOTERM_MF_2 | GO:0005200~structural constituent of cytoskeleton | 5.85E-04 |
|  | GOTERM_CC_5 | GO:0044446~intracellular organelle part | 6.43E-04 |
|  | GOTERM_BP_3 | GO:0048513~organ development | 7.28E-04 |
|  | GOTERM_BP_2 | GO:0051301~cell division | 7.90E-04 |
|  | GOTERM_BP_4 | GO:0048513~organ development | 8.87E-04 |
|  | GOTERM_BP_3 | GO:0048731~system development | 9.06E-04 |
|  | GOTERM_BP_4 | GO:0051174~regulation of phosphorus metabolic process | 0.001349 |
|  | GOTERM_BP_3 | GO:0051726~regulation of cell cycle | 0.00135 |
|  | GOTERM_BP_5 | GO:0019220~regulation of phosphate metabolic process | 0.001382 |
|  | GOTERM_BP_5 | GO:0051174~regulation of phosphorus metabolic process | 0.001382 |
|  | GOTERM_BP_4 | GO:0051726~regulation of cell cycle | 0.001485 |
|  | GOTERM_BP_2 | GO:0048856~anatomical structure development | 0.002014 |
|  | GOTERM_BP_2 | GO:0007275~multicellular organismal development | 0.00229 |
|  | GOTERM_CC_2 | GO:0043234~protein complex | 0.002466 |
|  | GOTERM_CC_2 | GO:0043228~non-membrane-bounded organelle | 0.002537 |
|  | GOTERM_CC_3 | GO:0043232~intracellular non-membrane-bounded organelle | 0.002554 |
|  | GOTERM_MF_4 | GO:0003700~transcription factor activity | 0.00258 |
|  | GOTERM_CC_4 | GO:0043232~intracellular non-membrane-bounded organelle | 0.002728 |
|  | GOTERM_CC_5 | GO:0043232~intracellular non-membrane-bounded organelle | 0.003175 |
|  | GOTERM_BP_2 | GO:0008283~cell proliferation | 0.004371 |
|  | GOTERM_MF_2 | GO:0003700~transcription factor activity | 0.004918 |
|  | GOTERM_CC_3 | GO:0044428~nuclear part | 0.004987 |
|  | GOTERM_CC_4 | GO:0044428~nuclear part | 0.005294 |
|  | GOTERM_BP_1 | GO:0032502~developmental process | 0.005513 |
|  | GOTERM_CC_5 | GO:0044428~nuclear part | 0.00596 |
|  | GOTERM_CC_3 | GO:0016533~cyclin-dependent protein kinase 5 activator complex | 0.00666 |
|  | GOTERM_CC_4 | GO:0016533~cyclin-dependent protein kinase 5 activator complex | 0.006735 |
|  | GOTERM_CC_5 | GO:0016533~cyclin-dependent protein kinase 5 activator complex | 0.006847 |
|  | GOTERM_MF_3 | GO:0003677~DNA binding | 0.007799 |
|  | GOTERM_MF_5 | GO:0008081~phosphoric diester hydrolase activity | 0.008808 |
|  | GOTERM_BP_2 | GO:0019222~regulation of metabolic process | 0.009026 |
|  | GOTERM_BP_3 | GO:0019222~regulation of metabolic process | 0.009218 |
|  | GOTERM_CC_1 | GO:0032991~macromolecular complex | 0.010052 |
|  | GOTERM_BP_2 | GO:0050789~regulation of biological process | 0.011181 |
|  | GOTERM_CC_3 | GO:0005667~transcription factor complex | 0.011188 |
|  | GOTERM_MF_5 | GO:0005138~interleukin-6 receptor binding | 0.011893 |
|  | GOTERM_CC_5 | GO:0005667~transcription factor complex | 0.012031 |
|  | GOTERM_CC_4 | GO:0005654~nucleoplasm | 0.01274 |
|  | GOTERM_CC_1 | GO:0043226~organelle | 0.012862 |
|  | GOTERM_MF_1 | GO:0005198~structural molecule activity | 0.012906 |
|  | GOTERM_MF_1 | GO:0005488~binding | 0.013048 |
|  | GOTERM_CC_4 | GO:0031981~nuclear lumen | 0.013111 |
|  | GOTERM_BP_5 | GO:0043549~regulation of kinase activity | 0.013239 |
|  | GOTERM_CC_3 | GO:0070013~intracellular organelle lumen | 0.01347 |
|  | GOTERM_CC_1 | GO:0031974~membrane-enclosed lumen | 0.013667 |
|  | GOTERM_CC_5 | GO:0005654~nucleoplasm | 0.013744 |
|  | GOTERM_CC_4 | GO:0070013~intracellular organelle lumen | 0.014236 |
|  | GOTERM_CC_5 | GO:0031981~nuclear lumen | 0.014403 |
|  | GOTERM_BP_3 | GO:0050794~regulation of cellular process | 0.014644 |
|  | GOTERM_BP_2 | GO:0050794~regulation of cellular process | 0.014755 |
|  | GOTERM_BP_4 | GO:0051338~regulation of transferase activity | 0.014868 |
|  | GOTERM_BP_3 | GO:0031323~regulation of cellular metabolic process | 0.01513 |
|  | GOTERM_CC_2 | GO:0043233~organelle lumen | 0.015436 |
|  | GOTERM_CC_3 | GO:0043233~organelle lumen | 0.015504 |
|  | GOTERM_CC_5 | GO:0070013~intracellular organelle lumen | 0.015765 |
|  | GOTERM_CC_3 | GO:0044430~cytoskeletal part | 0.017255 |
|  | GOTERM_CC_4 | GO:0044430~cytoskeletal part | 0.018024 |
|  | GOTERM_BP_4 | GO:0031323~regulation of cellular metabolic process | 0.018447 |
|  | GOTERM_CC_4 | GO:0005730~nucleolus | 0.018894 |
|  | GOTERM_CC_5 | GO:0044430~cytoskeletal part | 0.019408 |
|  | GOTERM_CC_5 | GO:0005730~nucleolus | 0.020149 |
|  | GOTERM_MF_4 | GO:0017069~snRNA binding | 0.020207 |
|  | GOTERM_BP_3 | GO:0060255~regulation of macromolecule metabolic process | 0.021369 |
|  | GOTERM_BP_1 | GO:0065007~biological regulation | 0.022047 |
|  | GOTERM_BP_3 | GO:0080090~regulation of primary metabolic process | 0.023198 |
|  | GOTERM_CC_2 | GO:0043229~intracellular organelle | 0.023993 |
|  | GOTERM_CC_3 | GO:0000307~cyclin-dependent protein kinase holoenzyme complex | 0.024209 |
|  | GOTERM_BP_2 | GO:0048518~positive regulation of biological process | 0.024237 |
|  | GOTERM_CC_3 | GO:0043229~intracellular organelle | 0.02427 |
|  | GOTERM_CC_4 | GO:0000307~cyclin-dependent protein kinase holoenzyme complex | 0.024479 |
|  | GOTERM_BP_3 | GO:0048518~positive regulation of biological process | 0.024867 |
|  | GOTERM_CC_5 | GO:0000307~cyclin-dependent protein kinase holoenzyme complex | 0.024884 |
|  | GOTERM_CC_4 | GO:0043229~intracellular organelle | 0.025614 |
|  | GOTERM_BP_4 | GO:0060255~regulation of macromolecule metabolic process | 0.025617 |
|  | GOTERM_BP_4 | GO:0080090~regulation of primary metabolic process | 0.027768 |
|  | GOTERM_CC_4 | GO:0005856~cytoskeleton | 0.031245 |
|  | GOTERM_BP_3 | GO:0031324~negative regulation of cellular metabolic process | 0.033159 |
|  | GOTERM_CC_5 | GO:0043229~intracellular organelle | 0.033471 |
|  | GOTERM_BP_2 | GO:0009056~catabolic process | 0.033556 |
|  | GOTERM_CC_5 | GO:0005856~cytoskeleton | 0.03381 |
|  | GOTERM_CC_4 | GO:0044451~nucleoplasm part | 0.034825 |
|  | GOTERM_BP_2 | GO:0048522~positive regulation of cellular process | 0.034869 |
|  | GOTERM_BP_4 | GO:0006350~transcription | 0.034997 |
|  | GOTERM_BP_3 | GO:0010605~negative regulation of macromolecule metabolic process | 0.035613 |
|  | GOTERM_BP_3 | GO:0048522~positive regulation of cellular process | 0.035745 |
|  | GOTERM_BP_4 | GO:0031324~negative regulation of cellular metabolic process | 0.035931 |
|  | GOTERM_CC_5 | GO:0044451~nucleoplasm part | 0.036678 |
|  | GOTERM_BP_5 | GO:0031324~negative regulation of cellular metabolic process | 0.036795 |
|  | GOTERM_BP_4 | GO:0010605~negative regulation of macromolecule metabolic process | 0.038569 |
|  | GOTERM_BP_5 | GO:0010605~negative regulation of macromolecule metabolic process | 0.039495 |
|  | GOTERM_BP_5 | GO:0006351~transcription, DNA-dependent | 0.039628 |
|  | GOTERM_BP_4 | GO:0032774~RNA biosynthetic process | 0.040244 |
|  | GOTERM_BP_4 | GO:0048522~positive regulation of cellular process | 0.040385 |
|  | GOTERM_BP_1 | GO:0032501~multicellular organismal process | 0.040843 |
|  | GOTERM_BP_5 | GO:0032774~RNA biosynthetic process | 0.040999 |
|  | GOTERM_BP_5 | GO:0008306~associative learning | 0.043374 |
|  | GOTERM_BP_2 | GO:0009892~negative regulation of metabolic process | 0.043638 |
|  | GOTERM_BP_3 | GO:0009892~negative regulation of metabolic process | 0.044468 |
|  | GOTERM_BP_4 | GO:0010556~regulation of macromolecule biosynthetic process | 0.045196 |
|  | GOTERM_BP_3 | GO:0042127~regulation of cell proliferation | 0.045924 |
|  | GOTERM_BP_5 | GO:0010556~regulation of macromolecule biosynthetic process | 0.046363 |
|  | GOTERM_MF_1 | GO:0030528~transcription regulator activity | 0.047718 |
|  | GOTERM_CC_2 | GO:0031594~neuromuscular junction | 0.047815 |
|  | GOTERM_BP_4 | GO:0009892~negative regulation of metabolic process | 0.048072 |
|  | GOTERM_BP_3 | GO:0045104~intermediate filament cytoskeleton organization | 0.049349 |
|  | GOTERM_BP_4 | GO:0042127~regulation of cell proliferation | 0.049633 |
| 6116 | GOTERM_MF_5 | GO:0004908~interleukin-1 receptor activity | 4.67E-08 |
|  | GOTERM_MF_4 | GO:0019966~interleukin-1 binding | 4.99E-07 |
|  | GOTERM_MF_5 | GO:0004896~cytokine receptor activity | 3.39E-05 |
|  | GOTERM_MF_4 | GO:0004896~cytokine receptor activity | 1.04E-04 |
|  | GOTERM_BP_3 | GO:0045087~innate immune response | 3.53E-04 |
|  | GOTERM_BP_4 | GO:0045087~innate immune response | 3.76E-04 |
|  | GOTERM_MF_3 | GO:0019838~growth factor binding | 5.22E-04 |
|  | GOTERM_MF_3 | GO:0019955~cytokine binding | 5.83E-04 |
|  | GOTERM_BP_1 | GO:0050896~response to stimulus | 0.001593 |
|  | GOTERM_BP_5 | GO:0045872~positive regulation of rhodopsin gene expression | 0.002114 |
|  | GOTERM_BP_5 | GO:0007468~regulation of rhodopsin gene expression | 0.00317 |
|  | GOTERM_MF_2 | GO:0005515~protein binding | 0.004912 |
|  | GOTERM_BP_2 | GO:0006955~immune response | 0.006713 |
|  | GOTERM_BP_5 | GO:0045893~positive regulation of transcription, DNA-dependent | 0.012419 |
|  | GOTERM_BP_5 | GO:0051254~positive regulation of RNA metabolic process | 0.012703 |
|  | GOTERM_BP_4 | GO:0051254~positive regulation of RNA metabolic process | 0.013037 |
|  | GOTERM_MF_3 | GO:0004872~receptor activity | 0.015753 |
|  | GOTERM_MF_4 | GO:0043565~sequence-specific DNA binding | 0.017522 |
|  | GOTERM_BP_5 | GO:0048699~generation of neurons | 0.019018 |
|  | GOTERM_BP_5 | GO:0045941~positive regulation of transcription | 0.019474 |
|  | GOTERM_BP_3 | GO:0010467~gene expression | 0.020851 |
|  | GOTERM_BP_5 | GO:0010628~positive regulation of gene expression | 0.02107 |
|  | GOTERM_BP_4 | GO:0010628~positive regulation of gene expression | 0.021592 |
|  | GOTERM_MF_1 | GO:0060089~molecular transducer activity | 0.021838 |
|  | GOTERM_MF_1 | GO:0005488~binding | 0.022822 |
|  | GOTERM_BP_5 | GO:0022008~neurogenesis | 0.023039 |
|  | GOTERM_BP_1 | GO:0002376~immune system process | 0.023122 |
|  | GOTERM_BP_4 | GO:0022008~neurogenesis | 0.023603 |
|  | GOTERM_BP_4 | GO:0007399~nervous system development | 0.023669 |
|  | GOTERM_BP_3 | GO:0006952~defense response | 0.023687 |
|  | GOTERM_MF_2 | GO:0004871~signal transducer activity | 0.024274 |
|  | GOTERM_BP_5 | GO:0042461~photoreceptor cell development | 0.025099 |
|  | GOTERM_BP_5 | GO:0045935~positive regulation of nucleobase, nucleoside, nucleotide and nucleic acid metabolic process | 0.025427 |
|  | GOTERM_BP_4 | GO:0045935~positive regulation of nucleobase, nucleoside, nucleotide and nucleic acid metabolic process | 0.026041 |
|  | GOTERM_BP_5 | GO:0046530~photoreceptor cell differentiation | 0.026132 |
|  | GOTERM_BP_3 | GO:0051173~positive regulation of nitrogen compound metabolic process | 0.02672 |
|  | GOTERM_BP_5 | GO:0051173~positive regulation of nitrogen compound metabolic process | 0.027611 |
|  | GOTERM_BP_4 | GO:0051173~positive regulation of nitrogen compound metabolic process | 0.028271 |
|  | GOTERM_BP_5 | GO:0010557~positive regulation of macromolecule biosynthetic process | 0.028741 |
|  | GOTERM_BP_4 | GO:0010557~positive regulation of macromolecule biosynthetic process | 0.029423 |
|  | GOTERM_BP_3 | GO:0060255~regulation of macromolecule metabolic process | 0.032343 |
|  | GOTERM_BP_5 | GO:0031328~positive regulation of cellular biosynthetic process | 0.032404 |
|  | GOTERM_BP_3 | GO:0009891~positive regulation of biosynthetic process | 0.032552 |
|  | GOTERM_BP_4 | GO:0031328~positive regulation of cellular biosynthetic process | 0.033159 |
|  | GOTERM_BP_5 | GO:0009891~positive regulation of biosynthetic process | 0.033638 |
|  | GOTERM_BP_3 | GO:0080090~regulation of primary metabolic process | 0.034023 |
|  | GOTERM_BP_4 | GO:0009891~positive regulation of biosynthetic process | 0.034416 |
|  | GOTERM_BP_4 | GO:0060255~regulation of macromolecule metabolic process | 0.03618 |
|  | GOTERM_BP_4 | GO:0080090~regulation of primary metabolic process | 0.038041 |
|  | GOTERM_MF_2 | GO:0003700~transcription factor activity | 0.03869 |
|  | GOTERM_BP_5 | GO:0045449~regulation of transcription | 0.038746 |
|  | GOTERM_BP_3 | GO:0044260~cellular macromolecule metabolic process | 0.047399 |
|  | GOTERM_BP_5 | GO:0048666~neuron development | 0.048404 |
|  | GOTERM_BP_4 | GO:0048666~neuron development | 0.048996 |
|  | GOTERM_BP_5 | GO:0007600~sensory perception | 0.049618 |
| 6121 | GOTERM_CC_3 | GO:0005643~nuclear pore | 2.95E-11 |
|  | GOTERM_CC_4 | GO:0005643~nuclear pore | 4.08E-11 |
|  | GOTERM_CC_5 | GO:0005643~nuclear pore | 4.64E-11 |
|  | GOTERM_CC_3 | GO:0046930~pore complex | 1.33E-10 |
|  | GOTERM_CC_4 | GO:0046930~pore complex | 1.84E-10 |
|  | GOTERM_CC_5 | GO:0046930~pore complex | 2.09E-10 |
|  | GOTERM_CC_3 | GO:0005635~nuclear envelope | 5.97E-08 |
|  | GOTERM_CC_4 | GO:0005635~nuclear envelope | 8.17E-08 |
|  | GOTERM_CC_5 | GO:0005635~nuclear envelope | 9.26E-08 |
|  | GOTERM_BP_2 | GO:0045184~establishment of protein localization | 2.27E-07 |
|  | GOTERM_BP_3 | GO:0015031~protein transport | 3.05E-07 |
|  | GOTERM_BP_3 | GO:0045184~establishment of protein localization | 3.39E-07 |
|  | GOTERM_BP_4 | GO:0015031~protein transport | 3.91E-07 |
|  | GOTERM_BP_4 | GO:0045184~establishment of protein localization | 4.34E-07 |
|  | GOTERM_BP_5 | GO:0015031~protein transport | 8.58E-07 |
|  | GOTERM_BP_3 | GO:0008104~protein localization | 1.61E-06 |
|  | GOTERM_BP_2 | GO:0033036~macromolecule localization | 1.66E-06 |
|  | GOTERM_CC_1 | GO:0031975~envelope | 2.91E-06 |
|  | GOTERM_CC_2 | GO:0031967~organelle envelope | 4.00E-06 |
|  | GOTERM_CC_3 | GO:0031967~organelle envelope | 4.03E-06 |
|  | GOTERM_CC_4 | GO:0031967~organelle envelope | 5.79E-06 |
|  | GOTERM_CC_5 | GO:0031967~organelle envelope | 6.69E-06 |
|  | GOTERM_BP_2 | GO:0051236~establishment of RNA localization | 1.02E-05 |
|  | GOTERM_CC_4 | GO:0005634~nucleus | 1.12E-05 |
|  | GOTERM_MF_5 | GO:0031267~small GTPase binding | 1.13E-05 |
|  | GOTERM_BP_3 | GO:0051236~establishment of RNA localization | 1.21E-05 |
|  | GOTERM_BP_3 | GO:0050658~RNA transport | 1.21E-05 |
|  | GOTERM_BP_4 | GO:0050657~nucleic acid transport | 1.34E-05 |
|  | GOTERM_BP_4 | GO:0050658~RNA transport | 1.34E-05 |
|  | GOTERM_BP_4 | GO:0051236~establishment of RNA localization | 1.34E-05 |
|  | GOTERM_BP_3 | GO:0006403~RNA localization | 1.40E-05 |
|  | GOTERM_CC_5 | GO:0005634~nucleus | 1.59E-05 |
|  | GOTERM_MF_4 | GO:0051020~GTPase binding | 1.61E-05 |
|  | GOTERM_BP_5 | GO:0050658~RNA transport | 1.86E-05 |
|  | GOTERM_BP_5 | GO:0050657~nucleic acid transport | 1.86E-05 |
|  | GOTERM_BP_4 | GO:0009755~hormone-mediated signaling | 2.16E-05 |
|  | GOTERM_BP_5 | GO:0000059~protein import into nucleus, docking | 2.44E-05 |
|  | GOTERM_BP_3 | GO:0015931~nucleobase, nucleoside, nucleotide and nucleic acid transport | 2.53E-05 |
|  | GOTERM_BP_4 | GO:0015931~nucleobase, nucleoside, nucleotide and nucleic acid transport | 2.81E-05 |
|  | GOTERM_BP_5 | GO:0009755~hormone-mediated signaling | 2.83E-05 |
|  | GOTERM_MF_2 | GO:0005515~protein binding | 6.82E-05 |
|  | GOTERM_MF_3 | GO:0008565~protein transporter activity | 1.27E-04 |
|  | GOTERM_BP_4 | GO:0051169~nuclear transport | 1.38E-04 |
|  | GOTERM_BP_3 | GO:0046907~intracellular transport | 1.55E-04 |
|  | GOTERM_BP_4 | GO:0051028~mRNA transport | 1.64E-04 |
|  | GOTERM_BP_5 | GO:0006913~nucleocytoplasmic transport | 1.80E-04 |
|  | GOTERM_CC_2 | GO:0012505~endomembrane system | 1.82E-04 |
|  | GOTERM_BP_4 | GO:0046907~intracellular transport | 1.83E-04 |
|  | GOTERM_CC_3 | GO:0012505~endomembrane system | 1.83E-04 |
|  | GOTERM_BP_5 | GO:0051169~nuclear transport | 1.91E-04 |
|  | GOTERM_BP_5 | GO:0006606~protein import into nucleus | 2.04E-04 |
|  | GOTERM_BP_5 | GO:0051028~mRNA transport | 2.13E-04 |
|  | GOTERM_CC_2 | GO:0044424~intracellular part | 3.48E-04 |
|  | GOTERM_CC_3 | GO:0044424~intracellular part | 3.56E-04 |
|  | GOTERM_BP_3 | GO:0032870~cellular response to hormone stimulus | 7.62E-04 |
|  | GOTERM_BP_4 | GO:0051174~regulation of phosphorus metabolic process | 7.74E-04 |
|  | GOTERM_BP_4 | GO:0017038~protein import | 7.80E-04 |
|  | GOTERM_BP_5 | GO:0006605~protein targeting | 7.85E-04 |
|  | GOTERM_BP_2 | GO:0051649~establishment of localization in cell | 8.15E-04 |
|  | GOTERM_BP_4 | GO:0032870~cellular response to hormone stimulus | 8.26E-04 |
|  | GOTERM_BP_2 | GO:0006810~transport | 9.39E-04 |
|  | GOTERM_BP_1 | GO:0051234~establishment of localization | 9.69E-04 |
|  | GOTERM_BP_5 | GO:0017038~protein import | 0.001008 |
|  | GOTERM_CC_2 | GO:0005622~intracellular | 0.001024 |
|  | GOTERM_BP_3 | GO:0051649~establishment of localization in cell | 0.001047 |
|  | GOTERM_CC_3 | GO:0005622~intracellular | 0.001048 |
|  | GOTERM_BP_2 | GO:0051234~establishment of localization | 0.001059 |
|  | GOTERM_BP_4 | GO:0051338~regulation of transferase activity | 0.001081 |
|  | GOTERM_CC_1 | GO:0043226~organelle | 0.00112 |
|  | GOTERM_BP_5 | GO:0019220~regulation of phosphate metabolic process | 0.001159 |
|  | GOTERM_BP_5 | GO:0051174~regulation of phosphorus metabolic process | 0.001159 |
|  | GOTERM_BP_5 | GO:0043549~regulation of kinase activity | 0.001247 |
|  | GOTERM_BP_5 | GO:0051347~positive regulation of transferase activity | 0.001284 |
|  | GOTERM_CC_4 | GO:0044424~intracellular part | 0.001291 |
|  | GOTERM_BP_3 | GO:0006810~transport | 0.001414 |
|  | GOTERM_BP_2 | GO:0051641~cellular localization | 0.001493 |
|  | GOTERM_BP_5 | GO:0033365~protein localization in organelle | 0.001506 |
|  | GOTERM_CC_3 | GO:0043231~intracellular membrane-bounded organelle | 0.001822 |
|  | GOTERM_CC_2 | GO:0043227~membrane-bounded organelle | 0.001828 |
|  | GOTERM_CC_3 | GO:0044428~nuclear part | 0.002277 |
|  | GOTERM_CC_3 | GO:0005737~cytoplasm | 0.00256 |
|  | GOTERM_CC_2 | GO:0043229~intracellular organelle | 0.002698 |
|  | GOTERM_CC_3 | GO:0043229~intracellular organelle | 0.002742 |
|  | GOTERM_BP_3 | GO:0034622~cellular macromolecular complex assembly | 0.00296 |
|  | GOTERM_CC_4 | GO:0044428~nuclear part | 0.003223 |
|  | GOTERM_BP_4 | GO:0034622~cellular macromolecular complex assembly | 0.003248 |
|  | GOTERM_CC_5 | GO:0044428~nuclear part | 0.0037 |
|  | GOTERM_BP_1 | GO:0051179~localization | 0.00376 |
|  | GOTERM_BP_3 | GO:0065003~macromolecular complex assembly | 0.004142 |
|  | GOTERM_BP_2 | GO:0034621~cellular macromolecular complex subunit organization | 0.004178 |
|  | GOTERM_CC_4 | GO:0043231~intracellular membrane-bounded organelle | 0.004248 |
|  | GOTERM_MF_3 | GO:0019899~enzyme binding | 0.004258 |
|  | GOTERM_BP_3 | GO:0034621~cellular macromolecular complex subunit organization | 0.004845 |
|  | GOTERM_BP_2 | GO:0043933~macromolecular complex subunit organization | 0.004891 |
|  | GOTERM_BP_4 | GO:0007242~intracellular signaling cascade | 0.005161 |
|  | GOTERM_CC_1 | GO:0044422~organelle part | 0.005402 |
|  | GOTERM_BP_3 | GO:0009725~response to hormone stimulus | 0.005441 |
|  | GOTERM_CC_4 | GO:0005737~cytoplasm | 0.005489 |
|  | GOTERM_BP_4 | GO:0043085~positive regulation of catalytic activity | 0.005756 |
|  | GOTERM_CC_5 | GO:0043231~intracellular membrane-bounded organelle | 0.005917 |
|  | GOTERM_BP_4 | GO:0006886~intracellular protein transport | 0.006443 |
|  | GOTERM_CC_4 | GO:0043229~intracellular organelle | 0.006819 |
|  | GOTERM_BP_2 | GO:0009719~response to endogenous stimulus | 0.007086 |
|  | GOTERM_CC_5 | GO:0005737~cytoplasm | 0.007398 |
|  | GOTERM_BP_2 | GO:0065009~regulation of molecular function | 0.007501 |
|  | GOTERM_CC_2 | GO:0044446~intracellular organelle part | 0.007949 |
|  | GOTERM_CC_3 | GO:0044446~intracellular organelle part | 0.008015 |
|  | GOTERM_BP_5 | GO:0007242~intracellular signaling cascade | 0.008367 |
|  | GOTERM_CC_2 | GO:0044422~organelle part | 0.008527 |
|  | GOTERM_BP_5 | GO:0006886~intracellular protein transport | 0.008565 |
|  | GOTERM_BP_1 | GO:0044085~cellular component biogenesis | 0.008676 |
|  | GOTERM_BP_3 | GO:0070727~cellular macromolecule localization | 0.008956 |
|  | GOTERM_BP_3 | GO:0044093~positive regulation of molecular function | 0.009154 |
|  | GOTERM_BP_4 | GO:0034613~cellular protein localization | 0.009495 |
|  | GOTERM_CC_5 | GO:0043229~intracellular organelle | 0.009725 |
|  | GOTERM_CC_4 | GO:0044446~intracellular organelle part | 0.012727 |
|  | GOTERM_BP_4 | GO:0043623~cellular protein complex assembly | 0.014756 |
|  | GOTERM_BP_3 | GO:0050790~regulation of catalytic activity | 0.014793 |
|  | GOTERM_CC_2 | GO:0043228~non-membrane-bounded organelle | 0.01513 |
|  | GOTERM_CC_3 | GO:0043232~intracellular non-membrane-bounded organelle | 0.015217 |
|  | GOTERM_CC_5 | GO:0044446~intracellular organelle part | 0.015263 |
|  | GOTERM_BP_2 | GO:0022607~cellular component assembly | 0.015735 |
|  | GOTERM_BP_2 | GO:0070271~protein complex biogenesis | 0.017223 |
|  | GOTERM_BP_5 | GO:0043623~cellular protein complex assembly | 0.017717 |
|  | GOTERM_BP_3 | GO:0006461~protein complex assembly | 0.019741 |
|  | GOTERM_BP_3 | GO:0007165~signal transduction | 0.020651 |
|  | GOTERM_CC_4 | GO:0043232~intracellular non-membrane-bounded organelle | 0.021025 |
|  | GOTERM_BP_4 | GO:0006461~protein complex assembly | 0.02146 |
|  | GOTERM_CC_5 | GO:0043232~intracellular non-membrane-bounded organelle | 0.023888 |
|  | GOTERM_BP_4 | GO:0007165~signal transduction | 0.024874 |
|  | GOTERM_BP_4 | GO:0046605~regulation of centrosome cycle | 0.025229 |
|  | GOTERM_BP_1 | GO:0016043~cellular component organization | 0.026533 |
|  | GOTERM_BP_5 | GO:0046605~regulation of centrosome cycle | 0.027018 |
|  | GOTERM_BP_3 | GO:0033043~regulation of organelle organization | 0.029908 |
|  | GOTERM_MF_1 | GO:0030234~enzyme regulator activity | 0.03014 |
|  | GOTERM_BP_1 | GO:0009987~cellular process | 0.03066 |
|  | GOTERM_MF_2 | GO:0060589~nucleoside-triphosphatase regulator activity | 0.031172 |
|  | GOTERM_BP_4 | GO:0033043~regulation of organelle organization | 0.031602 |
|  | GOTERM_MF_3 | GO:0030695~GTPase regulator activity | 0.031709 |
|  | GOTERM_MF_1 | GO:0005488~binding | 0.047265 |
|  | GOTERM_BP_2 | GO:0006323~DNA packaging | 0.048336 |
|  | GOTERM_CC_1 | GO:0032991~macromolecular complex | 0.0489 |
| 6126 | GOTERM_BP_3 | GO:0034367~macromolecular complex remodeling | 1.14E-04 |
|  | GOTERM_BP_4 | GO:0034368~protein-lipid complex remodeling | 1.19E-04 |
|  | GOTERM_BP_5 | GO:0034369~plasma lipoprotein particle remodeling | 1.37E-04 |
|  | GOTERM_CC_4 | GO:0034358~plasma lipoprotein particle | 4.24E-04 |
|  | GOTERM_CC_2 | GO:0032994~protein-lipid complex | 4.55E-04 |
|  | GOTERM_CC_2 | GO:0034358~plasma lipoprotein particle | 4.55E-04 |
|  | GOTERM_CC_3 | GO:0034358~plasma lipoprotein particle | 4.56E-04 |
|  | GOTERM_BP_5 | GO:0006641~triglyceride metabolic process | 6.44E-04 |
|  | GOTERM_BP_4 | GO:0006639~acylglycerol metabolic process | 7.30E-04 |
|  | GOTERM_BP_3 | GO:0006662~glycerol ether metabolic process | 7.57E-04 |
|  | GOTERM_BP_4 | GO:0006638~neutral lipid metabolic process | 7.60E-04 |
|  | GOTERM_BP_5 | GO:0006639~acylglycerol metabolic process | 8.37E-04 |
|  | GOTERM_BP_5 | GO:0006638~neutral lipid metabolic process | 8.71E-04 |
|  | GOTERM_BP_5 | GO:0055088~lipid homeostasis | 9.06E-04 |
|  | GOTERM_BP_2 | GO:0018904~organic ether metabolic process | 9.13E-04 |
|  | GOTERM_MF_3 | GO:0017129~triglyceride binding | 0.002144 |
|  | GOTERM_MF_2 | GO:0043167~ion binding | 0.002459 |
|  | GOTERM_MF_3 | GO:0043169~cation binding | 0.002876 |
|  | GOTERM_BP_4 | GO:0046486~glycerolipid metabolic process | 0.007685 |
|  | GOTERM_BP_5 | GO:0046486~glycerolipid metabolic process | 0.008781 |
|  | GOTERM_CC_4 | GO:0042627~chylomicron | 0.010588 |
|  | GOTERM_CC_5 | GO:0042627~chylomicron | 0.010764 |
|  | GOTERM_CC_3 | GO:0042627~chylomicron | 0.010941 |
|  | GOTERM_CC_4 | GO:0034361~very-low-density lipoprotein particle | 0.017589 |
|  | GOTERM_CC_4 | GO:0034385~triglyceride-rich lipoprotein particle | 0.017589 |
|  | GOTERM_BP_5 | GO:0032371~regulation of sterol transport | 0.01776 |
|  | GOTERM_CC_5 | GO:0034361~very-low-density lipoprotein particle | 0.01788 |
|  | GOTERM_CC_5 | GO:0034385~triglyceride-rich lipoprotein particle | 0.01788 |
|  | GOTERM_CC_3 | GO:0034385~triglyceride-rich lipoprotein particle | 0.018174 |
|  | GOTERM_MF_4 | GO:0019003~GDP binding | 0.020038 |
|  | GOTERM_BP_2 | GO:0043933~macromolecular complex subunit organization | 0.020477 |
|  | GOTERM_CC_4 | GO:0034364~high-density lipoprotein particle | 0.021941 |
|  | GOTERM_CC_5 | GO:0034364~high-density lipoprotein particle | 0.022304 |
|  | GOTERM_CC_3 | GO:0034364~high-density lipoprotein particle | 0.022669 |
|  | GOTERM_BP_4 | GO:0032368~regulation of lipid transport | 0.024775 |
|  | GOTERM_MF_4 | GO:0046872~metal ion binding | 0.025061 |
|  | GOTERM_BP_5 | GO:0032368~regulation of lipid transport | 0.026532 |
|  | GOTERM_BP_4 | GO:0015914~phospholipid transport | 0.027222 |
|  | GOTERM_BP_5 | GO:0015914~phospholipid transport | 0.02915 |
|  | GOTERM_BP_4 | GO:0015918~sterol transport | 0.030475 |
|  | GOTERM_BP_5 | GO:0015918~sterol transport | 0.03263 |
|  | GOTERM_BP_5 | GO:0030301~cholesterol transport | 0.03263 |
|  | GOTERM_BP_3 | GO:0009896~positive regulation of catabolic process | 0.039329 |
|  | GOTERM_BP_4 | GO:0009896~positive regulation of catabolic process | 0.040176 |
|  | GOTERM_MF_2 | GO:0005515~protein binding | 0.042356 |
|  | GOTERM_BP_5 | GO:0009896~positive regulation of catabolic process | 0.043003 |
|  | GOTERM_BP_1 | GO:0016043~cellular component organization | 0.046593 |
|  | GOTERM_BP_4 | GO:0018130~heterocycle biosynthetic process | 0.048193 |
| 6171 | GOTERM_MF_1 | GO:0030528~transcription regulator activity | 5.04E-10 |
|  | GOTERM_BP_5 | GO:0016481~negative regulation of transcription | 6.32E-10 |
|  | GOTERM_MF_2 | GO:0016564~transcription repressor activity | 8.00E-10 |
|  | GOTERM_BP_4 | GO:0010629~negative regulation of gene expression | 8.05E-10 |
|  | GOTERM_BP_3 | GO:0051172~negative regulation of nitrogen compound metabolic process | 8.68E-10 |
|  | GOTERM_BP_4 | GO:0051252~regulation of RNA metabolic process | 8.94E-10 |
|  | GOTERM_BP_4 | GO:0045934~negative regulation of nucleobase, nucleoside, nucleotide and nucleic acid metabolic process | 9.38E-10 |
|  | GOTERM_BP_4 | GO:0051172~negative regulation of nitrogen compound metabolic process | 1.07E-09 |
|  | GOTERM_BP_5 | GO:0010629~negative regulation of gene expression | 1.57E-09 |
|  | GOTERM_BP_5 | GO:0006355~regulation of transcription, DNA-dependent | 1.75E-09 |
|  | GOTERM_BP_4 | GO:0010558~negative regulation of macromolecule biosynthetic process | 1.78E-09 |
|  | GOTERM_BP_5 | GO:0045934~negative regulation of nucleobase, nucleoside, nucleotide and nucleic acid metabolic process | 1.83E-09 |
|  | GOTERM_BP_5 | GO:0051172~negative regulation of nitrogen compound metabolic process | 2.09E-09 |
|  | GOTERM_BP_3 | GO:0009890~negative regulation of biosynthetic process | 2.27E-09 |
|  | GOTERM_BP_4 | GO:0031327~negative regulation of cellular biosynthetic process | 2.28E-09 |
|  | GOTERM_BP_5 | GO:0051252~regulation of RNA metabolic process | 2.40E-09 |
|  | GOTERM_BP_4 | GO:0009890~negative regulation of biosynthetic process | 2.79E-09 |
|  | GOTERM_BP_5 | GO:0010558~negative regulation of macromolecule biosynthetic process | 3.47E-09 |
|  | GOTERM_BP_5 | GO:0031327~negative regulation of cellular biosynthetic process | 4.43E-09 |
|  | GOTERM_BP_5 | GO:0009890~negative regulation of biosynthetic process | 5.43E-09 |
|  | GOTERM_BP_3 | GO:0031324~negative regulation of cellular metabolic process | 2.04E-08 |
|  | GOTERM_BP_3 | GO:0010605~negative regulation of macromolecule metabolic process | 2.45E-08 |
|  | GOTERM_BP_4 | GO:0031324~negative regulation of cellular metabolic process | 2.51E-08 |
|  | GOTERM_BP_4 | GO:0051253~negative regulation of RNA metabolic process | 2.84E-08 |
|  | GOTERM_BP_3 | GO:0051171~regulation of nitrogen compound metabolic process | 2.97E-08 |
|  | GOTERM_BP_4 | GO:0010605~negative regulation of macromolecule metabolic process | 3.01E-08 |
|  | GOTERM_BP_5 | GO:0045449~regulation of transcription | 3.12E-08 |
|  | GOTERM_BP_4 | GO:0019219~regulation of nucleobase, nucleoside, nucleotide and nucleic acid metabolic process | 3.61E-08 |
|  | GOTERM_BP_4 | GO:0010556~regulation of macromolecule biosynthetic process | 3.93E-08 |
|  | GOTERM_BP_4 | GO:0051171~regulation of nitrogen compound metabolic process | 4.12E-08 |
|  | GOTERM_BP_5 | GO:0045892~negative regulation of transcription, DNA-dependent | 4.25E-08 |
|  | GOTERM_BP_3 | GO:0009892~negative regulation of metabolic process | 4.37E-08 |
|  | GOTERM_BP_4 | GO:0010468~regulation of gene expression | 4.50E-08 |
|  | GOTERM_BP_5 | GO:0031324~negative regulation of cellular metabolic process | 4.83E-08 |
|  | GOTERM_BP_5 | GO:0051253~negative regulation of RNA metabolic process | 4.84E-08 |
|  | GOTERM_BP_4 | GO:0009892~negative regulation of metabolic process | 5.37E-08 |
|  | GOTERM_BP_2 | GO:0009892~negative regulation of metabolic process | 5.68E-08 |
|  | GOTERM_BP_3 | GO:0009889~regulation of biosynthetic process | 5.74E-08 |
|  | GOTERM_BP_5 | GO:0010605~negative regulation of macromolecule metabolic process | 5.80E-08 |
|  | GOTERM_BP_4 | GO:0031326~regulation of cellular biosynthetic process | 7.21E-08 |
|  | GOTERM_BP_4 | GO:0009889~regulation of biosynthetic process | 7.94E-08 |
|  | GOTERM_BP_4 | GO:0006350~transcription | 9.86E-08 |
|  | GOTERM_BP_5 | GO:0019219~regulation of nucleobase, nucleoside, nucleotide and nucleic acid metabolic process | 1.02E-07 |
|  | GOTERM_MF_2 | GO:0003700~transcription factor activity | 1.06E-07 |
|  | GOTERM_BP_5 | GO:0010556~regulation of macromolecule biosynthetic process | 1.11E-07 |
|  | GOTERM_BP_5 | GO:0010468~regulation of gene expression | 1.27E-07 |
|  | GOTERM_BP_5 | GO:0031326~regulation of cellular biosynthetic process | 2.03E-07 |
|  | GOTERM_BP_3 | GO:0060255~regulation of macromolecule metabolic process | 2.34E-07 |
|  | GOTERM_BP_3 | GO:0080090~regulation of primary metabolic process | 2.71E-07 |
|  | GOTERM_CC_4 | GO:0005634~nucleus | 3.19E-07 |
|  | GOTERM_BP_4 | GO:0060255~regulation of macromolecule metabolic process | 3.23E-07 |
|  | GOTERM_MF_4 | GO:0003700~transcription factor activity | 3.57E-07 |
|  | GOTERM_BP_4 | GO:0080090~regulation of primary metabolic process | 3.74E-07 |
|  | GOTERM_CC_5 | GO:0005634~nucleus | 4.33E-07 |
|  | GOTERM_BP_3 | GO:0031323~regulation of cellular metabolic process | 5.78E-07 |
|  | GOTERM_BP_3 | GO:0010467~gene expression | 7.78E-07 |
|  | GOTERM_BP_4 | GO:0031323~regulation of cellular metabolic process | 7.96E-07 |
|  | GOTERM_BP_3 | GO:0019222~regulation of metabolic process | 1.11E-06 |
|  | GOTERM_BP_2 | GO:0019222~regulation of metabolic process | 2.11E-06 |
|  | GOTERM_MF_3 | GO:0003677~DNA binding | 2.45E-06 |
|  | GOTERM_BP_3 | GO:0009059~macromolecule biosynthetic process | 3.41E-06 |
|  | GOTERM_MF_2 | GO:0003676~nucleic acid binding | 3.45E-06 |
|  | GOTERM_BP_4 | GO:0034645~cellular macromolecule biosynthetic process | 4.10E-06 |
|  | GOTERM_BP_3 | GO:0006139~nucleobase, nucleoside, nucleotide and nucleic acid metabolic process | 4.45E-06 |
|  | GOTERM_CC_2 | GO:0043229~intracellular organelle | 4.71E-06 |
|  | GOTERM_CC_3 | GO:0043229~intracellular organelle | 4.79E-06 |
|  | GOTERM_BP_3 | GO:0048523~negative regulation of cellular process | 5.61E-06 |
|  | GOTERM_BP_4 | GO:0048523~negative regulation of cellular process | 6.95E-06 |
|  | GOTERM_BP_2 | GO:0048523~negative regulation of cellular process | 7.42E-06 |
|  | GOTERM_BP_3 | GO:0034641~cellular nitrogen compound metabolic process | 1.20E-05 |
|  | GOTERM_CC_4 | GO:0043229~intracellular organelle | 1.23E-05 |
|  | GOTERM_BP_3 | GO:0048519~negative regulation of biological process | 1.33E-05 |
|  | GOTERM_BP_2 | GO:0048519~negative regulation of biological process | 1.75E-05 |
|  | GOTERM_CC_5 | GO:0043229~intracellular organelle | 1.80E-05 |
|  | GOTERM_CC_1 | GO:0043226~organelle | 2.16E-05 |
|  | GOTERM_BP_2 | GO:0006807~nitrogen compound metabolic process | 2.89E-05 |
|  | GOTERM_BP_3 | GO:0044249~cellular biosynthetic process | 3.82E-05 |
|  | GOTERM_CC_3 | GO:0043231~intracellular membrane-bounded organelle | 7.52E-05 |
|  | GOTERM_CC_2 | GO:0043227~membrane-bounded organelle | 7.54E-05 |
|  | GOTERM_BP_2 | GO:0009058~biosynthetic process | 8.11E-05 |
|  | GOTERM_BP_1 | GO:0032502~developmental process | 1.13E-04 |
|  | GOTERM_CC_4 | GO:0043231~intracellular membrane-bounded organelle | 1.64E-04 |
|  | GOTERM_CC_5 | GO:0043231~intracellular membrane-bounded organelle | 2.24E-04 |
|  | GOTERM_CC_2 | GO:0044424~intracellular part | 2.28E-04 |
|  | GOTERM_CC_3 | GO:0044424~intracellular part | 2.32E-04 |
|  | GOTERM_BP_3 | GO:0050794~regulation of cellular process | 2.46E-04 |
|  | GOTERM_CC_2 | GO:0005622~intracellular | 5.02E-04 |
|  | GOTERM_CC_3 | GO:0005622~intracellular | 5.10E-04 |
|  | GOTERM_BP_2 | GO:0050794~regulation of cellular process | 5.25E-04 |
|  | GOTERM_CC_4 | GO:0044424~intracellular part | 5.96E-04 |
|  | GOTERM_BP_2 | GO:0050789~regulation of biological process | 9.88E-04 |
|  | GOTERM_BP_3 | GO:0044260~cellular macromolecule metabolic process | 0.001081 |
|  | GOTERM_BP_2 | GO:0043170~macromolecule metabolic process | 0.001122 |
|  | GOTERM_BP_1 | GO:0065007~biological regulation | 0.00193 |
|  | GOTERM_BP_3 | GO:0048731~system development | 0.003682 |
|  | GOTERM_BP_4 | GO:0007507~heart development | 0.003981 |
|  | GOTERM_BP_5 | GO:0007507~heart development | 0.004829 |
|  | GOTERM_BP_2 | GO:0007275~multicellular organismal development | 0.004961 |
|  | GOTERM_BP_3 | GO:0045786~negative regulation of cell cycle | 0.006332 |
|  | GOTERM_BP_4 | GO:0045786~negative regulation of cell cycle | 0.006603 |
|  | GOTERM_BP_3 | GO:0030154~cell differentiation | 0.007148 |
|  | GOTERM_BP_2 | GO:0048856~anatomical structure development | 0.007475 |
|  | GOTERM_BP_5 | GO:0045786~negative regulation of cell cycle | 0.007545 |
|  | GOTERM_MF_3 | GO:0008134~transcription factor binding | 0.009822 |
|  | GOTERM_BP_3 | GO:0048513~organ development | 0.00991 |
|  | GOTERM_BP_2 | GO:0048869~cellular developmental process | 0.00994 |
|  | GOTERM_BP_4 | GO:0048513~organ development | 0.011138 |
|  | GOTERM_BP_2 | GO:0044238~primary metabolic process | 0.012186 |
|  | GOTERM_BP_4 | GO:0045736~negative regulation of cyclin-dependent protein kinase activity | 0.01658 |
|  | GOTERM_MF_5 | GO:0004861~cyclin-dependent protein kinase inhibitor activity | 0.016949 |
|  | GOTERM_BP_5 | GO:0045736~negative regulation of cyclin-dependent protein kinase activity | 0.01776 |
|  | GOTERM_MF_4 | GO:0017069~snRNA binding | 0.018537 |
|  | GOTERM_BP_2 | GO:0044237~cellular metabolic process | 0.023665 |
|  | GOTERM_MF_3 | GO:0003714~transcription corepressor activity | 0.024487 |
|  | GOTERM_MF_5 | GO:0003714~transcription corepressor activity | 0.02482 |
|  | GOTERM_MF_5 | GO:0030291~protein serine/threonine kinase inhibitor activity | 0.028652 |
|  | GOTERM_BP_1 | GO:0032501~multicellular organismal process | 0.029405 |
|  | GOTERM_MF_4 | GO:0043566~structure-specific DNA binding | 0.029623 |
|  | GOTERM_MF_4 | GO:0016538~cyclin-dependent protein kinase regulator activity | 0.031318 |
|  | GOTERM_BP_1 | GO:0008152~metabolic process | 0.034604 |
|  | GOTERM_MF_2 | GO:0005515~protein binding | 0.03632 |
|  | GOTERM_CC_3 | GO:0044428~nuclear part | 0.048169 |
| 6240 | GOTERM_BP_2 | GO:0006810~transport | 2.37E-05 |
|  | GOTERM_BP_2 | GO:0051234~establishment of localization | 2.73E-05 |
|  | GOTERM_BP_4 | GO:0040018~positive regulation of multicellular organism growth | 3.80E-05 |
|  | GOTERM_BP_3 | GO:0040018~positive regulation of multicellular organism growth | 3.88E-05 |
|  | GOTERM_BP_3 | GO:0006810~transport | 3.88E-05 |
|  | GOTERM_BP_1 | GO:0051234~establishment of localization | 3.94E-05 |
|  | GOTERM_BP_5 | GO:0040018~positive regulation of multicellular organism growth | 4.27E-05 |
|  | GOTERM_CC_5 | GO:0016023~cytoplasmic membrane-bounded vesicle | 4.99E-05 |
|  | GOTERM_CC_3 | GO:0031988~membrane-bounded vesicle | 5.15E-05 |
|  | GOTERM_CC_4 | GO:0016023~cytoplasmic membrane-bounded vesicle | 5.48E-05 |
|  | GOTERM_BP_5 | GO:0043568~positive regulation of insulin-like growth factor receptor signaling pathway | 7.39E-05 |
|  | GOTERM_CC_3 | GO:0031410~cytoplasmic vesicle | 1.22E-04 |
|  | GOTERM_MF_2 | GO:0042277~peptide binding | 1.22E-04 |
|  | GOTERM_CC_5 | GO:0031410~cytoplasmic vesicle | 1.47E-04 |
|  | GOTERM_CC_4 | GO:0031410~cytoplasmic vesicle | 1.61E-04 |
|  | GOTERM_CC_2 | GO:0031982~vesicle | 1.62E-04 |
|  | GOTERM_BP_1 | GO:0051179~localization | 2.02E-04 |
|  | GOTERM_BP_5 | GO:0030252~growth hormone secretion | 2.06E-04 |
|  | GOTERM_BP_2 | GO:0033036~macromolecule localization | 2.85E-04 |
|  | GOTERM_BP_5 | GO:0043567~regulation of insulin-like growth factor receptor signaling pathway | 3.30E-04 |
|  | GOTERM_CC_5 | GO:0030659~cytoplasmic vesicle membrane | 3.83E-04 |
|  | GOTERM_CC_4 | GO:0030659~cytoplasmic vesicle membrane | 4.00E-04 |
|  | GOTERM_CC_3 | GO:0012506~vesicle membrane | 4.69E-04 |
|  | GOTERM_MF_5 | GO:0005509~calcium ion binding | 4.74E-04 |
|  | GOTERM_CC_5 | GO:0012506~vesicle membrane | 5.23E-04 |
|  | GOTERM_CC_4 | GO:0012506~vesicle membrane | 5.47E-04 |
|  | GOTERM_BP_4 | GO:0040014~regulation of multicellular organism growth | 5.56E-04 |
|  | GOTERM_BP_4 | GO:0006869~lipid transport | 5.65E-04 |
|  | GOTERM_BP_3 | GO:0040014~regulation of multicellular organism growth | 5.68E-04 |
|  | GOTERM_BP_3 | GO:0006869~lipid transport | 5.82E-04 |
|  | GOTERM_BP_4 | GO:0046883~regulation of hormone secretion | 7.01E-04 |
|  | GOTERM_BP_3 | GO:0010876~lipid localization | 7.84E-04 |
|  | GOTERM_BP_5 | GO:0046883~regulation of hormone secretion | 7.86E-04 |
|  | GOTERM_CC_3 | GO:0044433~cytoplasmic vesicle part | 0.001046 |
|  | GOTERM_BP_4 | GO:0046903~secretion | 0.001098 |
|  | GOTERM_BP_2 | GO:0045927~positive regulation of growth | 0.001133 |
|  | GOTERM_BP_3 | GO:0046903~secretion | 0.001137 |
|  | GOTERM_CC_5 | GO:0044433~cytoplasmic vesicle part | 0.001164 |
|  | GOTERM_CC_4 | GO:0044433~cytoplasmic vesicle part | 0.001216 |
|  | GOTERM_BP_4 | GO:0045927~positive regulation of growth | 0.001227 |
|  | GOTERM_BP_3 | GO:0045927~positive regulation of growth | 0.001253 |
|  | GOTERM_CC_2 | GO:0009986~cell surface | 0.00142 |
|  | GOTERM_CC_3 | GO:0009986~cell surface | 0.001424 |
|  | GOTERM_BP_2 | GO:0032940~secretion by cell | 0.001915 |
|  | GOTERM_BP_4 | GO:0051046~regulation of secretion | 0.001936 |
|  | GOTERM_BP_4 | GO:0032940~secretion by cell | 0.002117 |
|  | GOTERM_CC_5 | GO:0005783~endoplasmic reticulum | 0.00212 |
|  | GOTERM_BP_3 | GO:0032940~secretion by cell | 0.002175 |
|  | GOTERM_BP_5 | GO:0051046~regulation of secretion | 0.002232 |
|  | GOTERM_CC_4 | GO:0005783~endoplasmic reticulum | 0.002299 |
|  | GOTERM_CC_2 | GO:0012505~endomembrane system | 0.002386 |
|  | GOTERM_CC_3 | GO:0012505~endomembrane system | 0.002396 |
|  | GOTERM_BP_5 | GO:0032940~secretion by cell | 0.002439 |
|  | GOTERM_BP_2 | GO:0045184~establishment of protein localization | 0.003095 |
|  | GOTERM_BP_3 | GO:0042592~homeostatic process | 0.003298 |
|  | GOTERM_BP_4 | GO:0015031~protein transport | 0.003415 |
|  | GOTERM_BP_2 | GO:0051240~positive regulation of multicellular organismal process | 0.003471 |
|  | GOTERM_BP_3 | GO:0015031~protein transport | 0.003576 |
|  | GOTERM_BP_4 | GO:0045184~establishment of protein localization | 0.003593 |
|  | GOTERM_BP_3 | GO:0045184~establishment of protein localization | 0.003762 |
|  | GOTERM_BP_4 | GO:0051240~positive regulation of multicellular organismal process | 0.00383 |
|  | GOTERM_BP_3 | GO:0051240~positive regulation of multicellular organismal process | 0.003933 |
|  | GOTERM_BP_4 | GO:0060341~regulation of cellular localization | 0.004058 |
|  | GOTERM_BP_4 | GO:0046887~positive regulation of hormone secretion | 0.004082 |
|  | GOTERM_BP_3 | GO:0046887~positive regulation of hormone secretion | 0.004137 |
|  | GOTERM_BP_3 | GO:0060341~regulation of cellular localization | 0.004167 |
|  | GOTERM_BP_5 | GO:0015031~protein transport | 0.004222 |
|  | GOTERM_BP_2 | GO:0042221~response to chemical stimulus | 0.004352 |
|  | GOTERM_BP_5 | GO:0046887~positive regulation of hormone secretion | 0.004411 |
|  | GOTERM_BP_4 | GO:0030072~peptide hormone secretion | 0.004777 |
|  | GOTERM_MF_3 | GO:0042923~neuropeptide binding | 0.005069 |
|  | GOTERM_BP_5 | GO:0030072~peptide hormone secretion | 0.005161 |
|  | GOTERM_BP_4 | GO:0002790~peptide secretion | 0.005269 |
|  | GOTERM_BP_2 | GO:0051649~establishment of localization in cell | 0.005447 |
|  | GOTERM_BP_5 | GO:0002790~peptide secretion | 0.005692 |
|  | GOTERM_CC_1 | GO:0044421~extracellular region part | 0.00597 |
|  | GOTERM_BP_5 | GO:0006641~triglyceride metabolic process | 0.006246 |
|  | GOTERM_BP_4 | GO:0046879~hormone secretion | 0.006319 |
|  | GOTERM_BP_3 | GO:0046879~hormone secretion | 0.006403 |
|  | GOTERM_BP_3 | GO:0051649~establishment of localization in cell | 0.006585 |
|  | GOTERM_BP_5 | GO:0046879~hormone secretion | 0.006825 |
|  | GOTERM_BP_5 | GO:0002791~regulation of peptide secretion | 0.007123 |
|  | GOTERM_CC_2 | GO:0044421~extracellular region part | 0.007371 |
|  | GOTERM_BP_4 | GO:0009914~hormone transport | 0.007457 |
|  | GOTERM_BP_4 | GO:0006639~acylglycerol metabolic process | 0.007457 |
|  | GOTERM_BP_3 | GO:0010817~regulation of hormone levels | 0.007532 |
|  | GOTERM_BP_3 | GO:0009914~hormone transport | 0.007555 |
|  | GOTERM_BP_4 | GO:0006638~neutral lipid metabolic process | 0.007755 |
|  | GOTERM_BP_3 | GO:0008104~protein localization | 0.007923 |
|  | GOTERM_BP_5 | GO:0006639~acylglycerol metabolic process | 0.008051 |
|  | GOTERM_BP_3 | GO:0006662~glycerol ether metabolic process | 0.008164 |
|  | GOTERM_BP_2 | GO:0018904~organic ether metabolic process | 0.008221 |
|  | GOTERM_BP_5 | GO:0006638~neutral lipid metabolic process | 0.008372 |
|  | GOTERM_MF_2 | GO:0008047~enzyme activator activity | 0.008532 |
|  | GOTERM_BP_2 | GO:0051641~cellular localization | 0.008612 |
|  | GOTERM_BP_5 | GO:0055088~lipid homeostasis | 0.008699 |
|  | GOTERM_BP_4 | GO:0015833~peptide transport | 0.008999 |
|  | GOTERM_BP_3 | GO:0015833~peptide transport | 0.009117 |
|  | GOTERM_MF_1 | GO:0005488~binding | 0.009397 |
|  | GOTERM_CC_3 | GO:0005886~plasma membrane | 0.010332 |
|  | GOTERM_BP_2 | GO:0065008~regulation of biological quality | 0.010579 |
|  | GOTERM_BP_4 | GO:0048878~chemical homeostasis | 0.010766 |
|  | GOTERM_BP_3 | GO:0010033~response to organic substance | 0.011366 |
|  | GOTERM_CC_3 | GO:0005737~cytoplasm | 0.012375 |
|  | GOTERM_BP_5 | GO:0006457~protein folding | 0.012661 |
|  | GOTERM_CC_2 | GO:0000267~cell fraction | 0.013837 |
|  | GOTERM_CC_3 | GO:0000267~cell fraction | 0.013888 |
|  | GOTERM_CC_5 | GO:0005624~membrane fraction | 0.014049 |
|  | GOTERM_CC_5 | GO:0030662~coated vesicle membrane | 0.014237 |
|  | GOTERM_CC_3 | GO:0005626~insoluble fraction | 0.014536 |
|  | GOTERM_CC_2 | GO:0031090~organelle membrane | 0.014705 |
|  | GOTERM_CC_3 | GO:0031090~organelle membrane | 0.014759 |
|  | GOTERM_CC_4 | GO:0005624~membrane fraction | 0.014842 |
|  | GOTERM_CC_4 | GO:0005886~plasma membrane | 0.015408 |
|  | GOTERM_CC_5 | GO:0000323~lytic vacuole | 0.015445 |
|  | GOTERM_BP_4 | GO:0060124~positive regulation of growth hormone secretion | 0.015838 |
|  | GOTERM_BP_5 | GO:0060124~positive regulation of growth hormone secretion | 0.016485 |
|  | GOTERM_CC_5 | GO:0005788~endoplasmic reticulum lumen | 0.016934 |
|  | GOTERM_CC_4 | GO:0005788~endoplasmic reticulum lumen | 0.017292 |
|  | GOTERM_CC_4 | GO:0005626~insoluble fraction | 0.01749 |
|  | GOTERM_CC_5 | GO:0005737~cytoplasm | 0.017811 |
|  | GOTERM_CC_4 | GO:0031090~organelle membrane | 0.018151 |
|  | GOTERM_BP_2 | GO:0032879~regulation of localization | 0.019338 |
|  | GOTERM_BP_2 | GO:0003001~generation of a signal involved in cell-cell signaling | 0.020228 |
|  | GOTERM_BP_4 | GO:0003001~generation of a signal involved in cell-cell signaling | 0.021328 |
|  | GOTERM_BP_5 | GO:0060123~regulation of growth hormone secretion | 0.021921 |
|  | GOTERM_BP_3 | GO:0032879~regulation of localization | 0.022149 |
|  | GOTERM_CC_4 | GO:0005737~cytoplasm | 0.0227 |
|  | GOTERM_BP_4 | GO:0044269~glycerol ether catabolic process | 0.023665 |
|  | GOTERM_BP_3 | GO:0032094~response to food | 0.023817 |
|  | GOTERM_BP_3 | GO:0021984~adenohypophysis development | 0.023817 |
|  | GOTERM_CC_5 | GO:0005773~vacuole | 0.024607 |
|  | GOTERM_BP_5 | GO:0046464~acylglycerol catabolic process | 0.024629 |
|  | GOTERM_BP_5 | GO:0046461~neutral lipid catabolic process | 0.024629 |
|  | GOTERM_BP_5 | GO:0021984~adenohypophysis development | 0.024629 |
|  | GOTERM_BP_5 | GO:0032094~response to food | 0.024629 |
|  | GOTERM_CC_5 | GO:0030132~clathrin coat of coated pit | 0.024682 |
|  | GOTERM_CC_4 | GO:0030132~clathrin coat of coated pit | 0.024948 |
|  | GOTERM_CC_4 | GO:0005773~vacuole | 0.025339 |
|  | GOTERM_CC_3 | GO:0044444~cytoplasmic part | 0.025719 |
|  | GOTERM_MF_2 | GO:0005515~protein binding | 0.026195 |
|  | GOTERM_BP_4 | GO:0042953~lipoprotein transport | 0.026261 |
|  | GOTERM_BP_5 | GO:0019932~second-messenger-mediated signaling | 0.026703 |
|  | GOTERM_BP_5 | GO:0042953~lipoprotein transport | 0.027328 |
|  | GOTERM_BP_4 | GO:0051049~regulation of transport | 0.027366 |
|  | GOTERM_BP_3 | GO:0051049~regulation of transport | 0.028008 |
|  | GOTERM_MF_5 | GO:0050750~low-density lipoprotein receptor binding | 0.029439 |
|  | GOTERM_BP_5 | GO:0007186~G-protein coupled receptor protein signaling pathway | 0.031405 |
|  | GOTERM_CC_5 | GO:0030130~clathrin coat of trans-Golgi network vesicle | 0.031971 |
|  | GOTERM_CC_4 | GO:0030130~clathrin coat of trans-Golgi network vesicle | 0.032314 |
|  | GOTERM_BP_5 | GO:0046503~glycerolipid catabolic process | 0.032706 |
|  | GOTERM_CC_5 | GO:0044444~cytoplasmic part | 0.033653 |
|  | GOTERM_BP_4 | GO:0051047~positive regulation of secretion | 0.033801 |
|  | GOTERM_BP_3 | GO:0051047~positive regulation of secretion | 0.034221 |
|  | GOTERM_CC_5 | GO:0012510~trans-Golgi network transport vesicle membrane | 0.034389 |
|  | GOTERM_MF_4 | GO:0005179~hormone activity | 0.034586 |
|  | GOTERM_CC_5 | GO:0048471~perinuclear region of cytoplasm | 0.03462 |
|  | GOTERM_CC_4 | GO:0048471~perinuclear region of cytoplasm | 0.035622 |
|  | GOTERM_MF_3 | GO:0051082~unfolded protein binding | 0.03595 |
|  | GOTERM_BP_5 | GO:0051047~positive regulation of secretion | 0.036355 |
|  | GOTERM_CC_4 | GO:0044444~cytoplasmic part | 0.0386 |
|  | GOTERM_MF_4 | GO:0070325~lipoprotein receptor binding | 0.040028 |
|  | GOTERM_BP_5 | GO:0030810~positive regulation of nucleotide biosynthetic process | 0.040719 |
|  | GOTERM_BP_5 | GO:0045981~positive regulation of nucleotide metabolic process | 0.040719 |
|  | GOTERM_BP_4 | GO:0034381~lipoprotein particle clearance | 0.041695 |
|  | GOTERM_BP_4 | GO:0007242~intracellular signaling cascade | 0.044241 |
|  | GOTERM_CC_5 | GO:0030125~clathrin vesicle coat | 0.04639 |
| 6248 | GOTERM_CC_2 | GO:0044421~extracellular region part | 2.44E-05 |
|  | GOTERM_CC_1 | GO:0044421~extracellular region part | 2.49E-05 |
|  | GOTERM_CC_2 | GO:0031012~extracellular matrix | 3.04E-04 |
|  | GOTERM_CC_3 | GO:0031012~extracellular matrix | 3.05E-04 |
|  | GOTERM_CC_1 | GO:0005576~extracellular region | 3.48E-04 |
|  | GOTERM_BP_4 | GO:0008015~blood circulation | 3.86E-04 |
|  | GOTERM_BP_3 | GO:0003013~circulatory system process | 4.16E-04 |
|  | GOTERM_MF_5 | GO:0008528~peptide receptor activity, G-protein coupled | 6.01E-04 |
|  | GOTERM_MF_4 | GO:0001653~peptide receptor activity | 6.49E-04 |
|  | GOTERM_MF_4 | GO:0008528~peptide receptor activity, G-protein coupled | 6.49E-04 |
|  | GOTERM_MF_3 | GO:0001653~peptide receptor activity | 6.64E-04 |
|  | GOTERM_MF_3 | GO:0017046~peptide hormone binding | 7.65E-04 |
|  | GOTERM_BP_3 | GO:0008217~regulation of blood pressure | 9.13E-04 |
|  | GOTERM_BP_5 | GO:0008217~regulation of blood pressure | 9.34E-04 |
|  | GOTERM_CC_3 | GO:0005578~proteinaceous extracellular matrix | 0.002263 |
|  | GOTERM_CC_4 | GO:0005578~proteinaceous extracellular matrix | 0.002274 |
|  | GOTERM_MF_2 | GO:0005515~protein binding | 0.002294 |
|  | GOTERM_MF_2 | GO:0042562~hormone binding | 0.003059 |
|  | GOTERM_BP_2 | GO:0043062~extracellular structure organization | 0.003346 |
|  | GOTERM_MF_4 | GO:0004784~superoxide dismutase activity | 0.00458 |
|  | GOTERM_MF_3 | GO:0016721~oxidoreductase activity, acting on superoxide radicals as acceptor | 0.004591 |
|  | GOTERM_MF_2 | GO:0042277~peptide binding | 0.00466 |
|  | GOTERM_CC_2 | GO:0005615~extracellular space | 0.006313 |
|  | GOTERM_CC_3 | GO:0005615~extracellular space | 0.006332 |
|  | GOTERM_CC_5 | GO:0005604~basement membrane | 0.007374 |
|  | GOTERM_CC_3 | GO:0005604~basement membrane | 0.00831 |
|  | GOTERM_CC_4 | GO:0005604~basement membrane | 0.008353 |
|  | GOTERM_MF_4 | GO:0004383~guanylate cyclase activity | 0.01217 |
|  | GOTERM_MF_3 | GO:0004383~guanylate cyclase activity | 0.012197 |
|  | GOTERM_BP_4 | GO:0042127~regulation of cell proliferation | 0.014694 |
|  | GOTERM_BP_3 | GO:0042127~regulation of cell proliferation | 0.015896 |
|  | GOTERM_BP_3 | GO:0030198~extracellular matrix organization | 0.017024 |
|  | GOTERM_CC_2 | GO:0044420~extracellular matrix part | 0.017978 |
|  | GOTERM_CC_3 | GO:0044420~extracellular matrix part | 0.018003 |
|  | GOTERM_CC_4 | GO:0044420~extracellular matrix part | 0.018096 |
|  | GOTERM_BP_1 | GO:0009987~cellular process | 0.025866 |
|  | GOTERM_BP_5 | GO:0046068~cGMP metabolic process | 0.027005 |
|  | GOTERM_BP_4 | GO:0008285~negative regulation of cell proliferation | 0.030037 |
|  | GOTERM_BP_3 | GO:0008285~negative regulation of cell proliferation | 0.031498 |
|  | GOTERM_BP_5 | GO:0008285~negative regulation of cell proliferation | 0.032202 |
|  | GOTERM_MF_3 | GO:0016849~phosphorus-oxygen lyase activity | 0.033203 |
|  | GOTERM_BP_1 | GO:0044085~cellular component biogenesis | 0.034117 |
|  | GOTERM_MF_2 | GO:0009975~cyclase activity | 0.038103 |
|  | GOTERM_BP_4 | GO:0045429~positive regulation of nitric oxide biosynthetic process | 0.039133 |
|  | GOTERM_BP_5 | GO:0045429~positive regulation of nitric oxide biosynthetic process | 0.040244 |
|  | GOTERM_BP_4 | GO:0006801~superoxide metabolic process | 0.046418 |
| 6320 | GOTERM_BP_4 | GO:0042135~neurotransmitter catabolic process | 1.34E-04 |
|  | GOTERM_BP_5 | GO:0042135~neurotransmitter catabolic process | 1.42E-04 |
|  | GOTERM_CC_1 | GO:0045202~synapse | 4.84E-04 |
|  | GOTERM_BP_4 | GO:0019226~transmission of nerve impulse | 5.34E-04 |
|  | GOTERM_BP_4 | GO:0042133~neurotransmitter metabolic process | 8.44E-04 |
|  | GOTERM_BP_3 | GO:0042133~neurotransmitter metabolic process | 8.73E-04 |
|  | GOTERM_BP_3 | GO:0050877~neurological system process | 0.001953 |
|  | GOTERM_BP_2 | GO:0006066~alcohol metabolic process | 0.002032 |
|  | GOTERM_BP_3 | GO:0042439~ethanolamine and derivative metabolic process | 0.002089 |
|  | GOTERM_BP_5 | GO:0042439~ethanolamine and derivative metabolic process | 0.002141 |
|  | GOTERM_BP_4 | GO:0007268~synaptic transmission | 0.002589 |
|  | GOTERM_BP_5 | GO:0007268~synaptic transmission | 0.002864 |
|  | GOTERM_CC_3 | GO:0005578~proteinaceous extracellular matrix | 0.003363 |
|  | GOTERM_CC_4 | GO:0005578~proteinaceous extracellular matrix | 0.003432 |
|  | GOTERM_BP_5 | GO:0006581~acetylcholine catabolic process | 0.004064 |
|  | GOTERM_CC_2 | GO:0031012~extracellular matrix | 0.004387 |
|  | GOTERM_CC_3 | GO:0031012~extracellular matrix | 0.004398 |
|  | GOTERM_BP_4 | GO:0008291~acetylcholine metabolic process | 0.00591 |
|  | GOTERM_BP_5 | GO:0008291~acetylcholine metabolic process | 0.00609 |
|  | GOTERM_BP_2 | GO:0007154~cell communication | 0.006163 |
|  | GOTERM_BP_3 | GO:0007267~cell-cell signaling | 0.006175 |
|  | GOTERM_MF_4 | GO:0043237~laminin-1 binding | 0.006865 |
|  | GOTERM_MF_5 | GO:0004104~cholinesterase activity | 0.006914 |
|  | GOTERM_BP_1 | GO:0032501~multicellular organismal process | 0.007431 |
|  | GOTERM_BP_3 | GO:0001505~regulation of neurotransmitter levels | 0.007903 |
|  | GOTERM_BP_5 | GO:0001505~regulation of neurotransmitter levels | 0.008098 |
|  | GOTERM_CC_1 | GO:0044421~extracellular region part | 0.0081 |
|  | GOTERM_CC_2 | GO:0044421~extracellular region part | 0.00975 |
|  | GOTERM_BP_2 | GO:0065008~regulation of biological quality | 0.01048 |
|  | GOTERM_BP_2 | GO:0003008~system process | 0.011967 |
|  | GOTERM_BP_4 | GO:0060341~regulation of cellular localization | 0.012447 |
|  | GOTERM_BP_4 | GO:0010648~negative regulation of cell communication | 0.012447 |
|  | GOTERM_BP_3 | GO:0010648~negative regulation of cell communication | 0.013034 |
|  | GOTERM_BP_3 | GO:0060341~regulation of cellular localization | 0.013034 |
|  | GOTERM_BP_5 | GO:0010648~negative regulation of cell communication | 0.013446 |
|  | GOTERM_CC_5 | GO:0019898~extrinsic to membrane | 0.014488 |
|  | GOTERM_CC_3 | GO:0019898~extrinsic to membrane | 0.015184 |
|  | GOTERM_BP_1 | GO:0022610~biological adhesion | 0.015388 |
|  | GOTERM_CC_4 | GO:0019898~extrinsic to membrane | 0.015484 |
|  | GOTERM_BP_4 | GO:0006576~biogenic amine metabolic process | 0.015546 |
|  | GOTERM_BP_2 | GO:0007155~cell adhesion | 0.015767 |
|  | GOTERM_BP_5 | GO:0006576~biogenic amine metabolic process | 0.016432 |
|  | GOTERM_MF_2 | GO:0005515~protein binding | 0.017121 |
|  | GOTERM_BP_2 | GO:0007610~behavior | 0.017317 |
|  | GOTERM_CC_2 | GO:0044420~extracellular matrix part | 0.021953 |
|  | GOTERM_CC_3 | GO:0044420~extracellular matrix part | 0.021982 |
|  | GOTERM_CC_4 | GO:0044420~extracellular matrix part | 0.022258 |
|  | GOTERM_MF_3 | GO:0043236~laminin binding | 0.023357 |
|  | GOTERM_MF_3 | GO:0019899~enzyme binding | 0.024334 |
|  | GOTERM_MF_3 | GO:0001540~beta-amyloid binding | 0.027547 |
|  | GOTERM_BP_5 | GO:0007271~synaptic transmission, cholinergic | 0.028115 |
|  | GOTERM_BP_5 | GO:0042402~biogenic amine catabolic process | 0.030094 |
|  | GOTERM_CC_5 | GO:0005605~basal lamina | 0.032504 |
|  | GOTERM_CC_3 | GO:0005605~basal lamina | 0.032868 |
|  | GOTERM_CC_4 | GO:0005605~basal lamina | 0.033102 |
|  | GOTERM_BP_4 | GO:0042219~cellular amino acid derivative catabolic process | 0.036864 |
|  | GOTERM_BP_5 | GO:0042219~cellular amino acid derivative catabolic process | 0.037971 |
|  | GOTERM_BP_5 | GO:0050805~negative regulation of synaptic transmission | 0.041887 |
|  | GOTERM_BP_4 | GO:0006575~cellular amino acid derivative metabolic process | 0.042092 |
|  | GOTERM_BP_3 | GO:0009308~amine metabolic process | 0.044941 |
|  | GOTERM_BP_2 | GO:0009056~catabolic process | 0.047555 |
|  | GOTERM_BP_2 | GO:0043062~extracellular structure organization | 0.047709 |
|  | GOTERM_BP_4 | GO:0051970~negative regulation of transmission of nerve impulse | 0.048232 |
|  | GOTERM_BP_4 | GO:0010646~regulation of cell communication | 0.049385 |
|  | GOTERM_BP_5 | GO:0051970~negative regulation of transmission of nerve impulse | 0.049672 |
| 6404 | GOTERM_CC_4 | GO:0043189~H4/H2A histone acetyltransferase complex | 1.14E-06 |
|  | GOTERM_BP_4 | GO:0006350~transcription | 2.39E-06 |
|  | GOTERM_MF_1 | GO:0030528~transcription regulator activity | 6.93E-06 |
|  | GOTERM_CC_4 | GO:0005634~nucleus | 1.79E-05 |
|  | GOTERM_CC_5 | GO:0005634~nucleus | 2.30E-05 |
|  | GOTERM_CC_3 | GO:0000123~histone acetyltransferase complex | 4.20E-05 |
|  | GOTERM_CC_5 | GO:0000123~histone acetyltransferase complex | 4.99E-05 |
|  | GOTERM_BP_3 | GO:0009059~macromolecule biosynthetic process | 5.61E-05 |
|  | GOTERM_BP_3 | GO:0051171~regulation of nitrogen compound metabolic process | 5.77E-05 |
|  | GOTERM_BP_5 | GO:0045449~regulation of transcription | 5.98E-05 |
|  | GOTERM_BP_4 | GO:0034645~cellular macromolecule biosynthetic process | 6.60E-05 |
|  | GOTERM_BP_4 | GO:0019219~regulation of nucleobase, nucleoside, nucleotide and nucleic acid metabolic process | 6.65E-05 |
|  | GOTERM_BP_4 | GO:0010556~regulation of macromolecule biosynthetic process | 7.08E-05 |
|  | GOTERM_BP_4 | GO:0051171~regulation of nitrogen compound metabolic process | 7.33E-05 |
|  | GOTERM_BP_3 | GO:0006139~nucleobase, nucleoside, nucleotide and nucleic acid metabolic process | 7.81E-05 |
|  | GOTERM_BP_4 | GO:0010468~regulation of gene expression | 7.83E-05 |
|  | GOTERM_BP_2 | GO:0009058~biosynthetic process | 8.11E-05 |
|  | GOTERM_BP_3 | GO:0044249~cellular biosynthetic process | 8.76E-05 |
|  | GOTERM_BP_3 | GO:0009889~regulation of biosynthetic process | 9.35E-05 |
|  | GOTERM_BP_3 | GO:0010467~gene expression | 1.06E-04 |
|  | GOTERM_BP_4 | GO:0031326~regulation of cellular biosynthetic process | 1.11E-04 |
|  | GOTERM_BP_4 | GO:0009889~regulation of biosynthetic process | 1.19E-04 |
|  | GOTERM_BP_5 | GO:0019219~regulation of nucleobase, nucleoside, nucleotide and nucleic acid metabolic process | 1.42E-04 |
|  | GOTERM_BP_5 | GO:0010556~regulation of macromolecule biosynthetic process | 1.51E-04 |
|  | GOTERM_BP_5 | GO:0010468~regulation of gene expression | 1.67E-04 |
|  | GOTERM_BP_2 | GO:0006807~nitrogen compound metabolic process | 1.73E-04 |
|  | GOTERM_CC_5 | GO:0035267~NuA4 histone acetyltransferase complex | 1.82E-04 |
|  | GOTERM_BP_3 | GO:0034641~cellular nitrogen compound metabolic process | 1.86E-04 |
|  | GOTERM_BP_5 | GO:0031326~regulation of cellular biosynthetic process | 2.35E-04 |
|  | GOTERM_BP_3 | GO:0060255~regulation of macromolecule metabolic process | 2.60E-04 |
|  | GOTERM_BP_3 | GO:0080090~regulation of primary metabolic process | 2.89E-04 |
|  | GOTERM_BP_1 | GO:0065007~biological regulation | 3.23E-04 |
|  | GOTERM_BP_4 | GO:0060255~regulation of macromolecule metabolic process | 3.28E-04 |
|  | GOTERM_BP_4 | GO:0080090~regulation of primary metabolic process | 3.64E-04 |
|  | GOTERM_BP_3 | GO:0031323~regulation of cellular metabolic process | 4.99E-04 |
|  | GOTERM_BP_3 | GO:0044260~cellular macromolecule metabolic process | 5.42E-04 |
|  | GOTERM_BP_2 | GO:0019222~regulation of metabolic process | 5.49E-04 |
|  | GOTERM_BP_4 | GO:0031323~regulation of cellular metabolic process | 6.26E-04 |
|  | GOTERM_MF_3 | GO:0008134~transcription factor binding | 6.77E-04 |
|  | GOTERM_BP_3 | GO:0019222~regulation of metabolic process | 7.93E-04 |
|  | GOTERM_CC_1 | GO:0043226~organelle | 8.76E-04 |
|  | GOTERM_BP_5 | GO:0016481~negative regulation of transcription | 8.80E-04 |
|  | GOTERM_CC_4 | GO:0044451~nucleoplasm part | 9.36E-04 |
|  | GOTERM_BP_4 | GO:0010629~negative regulation of gene expression | 9.83E-04 |
|  | GOTERM_CC_5 | GO:0044451~nucleoplasm part | 0.001009 |
|  | GOTERM_BP_3 | GO:0051172~negative regulation of nitrogen compound metabolic process | 0.001017 |
|  | GOTERM_BP_4 | GO:0045934~negative regulation of nucleobase, nucleoside, nucleotide and nucleic acid metabolic process | 0.001055 |
|  | GOTERM_BP_5 | GO:0016568~chromatin modification | 0.001072 |
|  | GOTERM_BP_4 | GO:0051172~negative regulation of nitrogen compound metabolic process | 0.001121 |
|  | GOTERM_BP_2 | GO:0043170~macromolecule metabolic process | 0.001122 |
|  | GOTERM_MF_3 | GO:0016566~specific transcriptional repressor activity | 0.001228 |
|  | GOTERM_MF_2 | GO:0003712~transcription cofactor activity | 0.001255 |
|  | GOTERM_BP_5 | GO:0010629~negative regulation of gene expression | 0.001339 |
|  | GOTERM_MF_2 | GO:0003700~transcription factor activity | 0.001388 |
|  | GOTERM_BP_4 | GO:0010558~negative regulation of macromolecule biosynthetic process | 0.001418 |
|  | GOTERM_BP_5 | GO:0045934~negative regulation of nucleobase, nucleoside, nucleotide and nucleic acid metabolic process | 0.001437 |
|  | GOTERM_BP_5 | GO:0051172~negative regulation of nitrogen compound metabolic process | 0.001526 |
|  | GOTERM_BP_3 | GO:0009890~negative regulation of biosynthetic process | 0.001583 |
|  | GOTERM_BP_4 | GO:0031327~negative regulation of cellular biosynthetic process | 0.001586 |
|  | GOTERM_CC_2 | GO:0043229~intracellular organelle | 0.001647 |
|  | GOTERM_CC_3 | GO:0043229~intracellular organelle | 0.001666 |
|  | GOTERM_BP_4 | GO:0009890~negative regulation of biosynthetic process | 0.001742 |
|  | GOTERM_BP_2 | GO:0044237~cellular metabolic process | 0.001821 |
|  | GOTERM_BP_5 | GO:0010558~negative regulation of macromolecule biosynthetic process | 0.001926 |
|  | GOTERM_MF_4 | GO:0003712~transcription cofactor activity | 0.00193 |
|  | GOTERM_CC_3 | GO:0044428~nuclear part | 0.001948 |
|  | GOTERM_MF_2 | GO:0016563~transcription activator activity | 0.001965 |
|  | GOTERM_BP_5 | GO:0031327~negative regulation of cellular biosynthetic process | 0.002153 |
|  | GOTERM_BP_5 | GO:0009890~negative regulation of biosynthetic process | 0.002362 |
|  | GOTERM_MF_5 | GO:0003713~transcription coactivator activity | 0.002387 |
|  | GOTERM_MF_4 | GO:0003700~transcription factor activity | 0.002473 |
|  | GOTERM_CC_4 | GO:0044428~nuclear part | 0.002541 |
|  | GOTERM_BP_2 | GO:0050794~regulation of cellular process | 0.002645 |
|  | GOTERM_BP_4 | GO:0006325~chromatin organization | 0.002711 |
|  | GOTERM_CC_5 | GO:0044428~nuclear part | 0.002826 |
|  | GOTERM_CC_4 | GO:0031981~nuclear lumen | 0.003041 |
|  | GOTERM_BP_2 | GO:0044238~primary metabolic process | 0.003249 |
|  | GOTERM_CC_4 | GO:0043229~intracellular organelle | 0.003266 |
|  | GOTERM_CC_5 | GO:0031981~nuclear lumen | 0.003342 |
|  | GOTERM_MF_3 | GO:0003713~transcription coactivator activity | 0.00347 |
|  | GOTERM_BP_3 | GO:0050794~regulation of cellular process | 0.004256 |
|  | GOTERM_CC_5 | GO:0043229~intracellular organelle | 0.004272 |
|  | GOTERM_BP_3 | GO:0031324~negative regulation of cellular metabolic process | 0.004288 |
|  | GOTERM_MF_5 | GO:0035257~nuclear hormone receptor binding | 0.004356 |
|  | GOTERM_BP_2 | GO:0050789~regulation of biological process | 0.004616 |
|  | GOTERM_BP_3 | GO:0010605~negative regulation of macromolecule metabolic process | 0.004657 |
|  | GOTERM_BP_4 | GO:0031324~negative regulation of cellular metabolic process | 0.004704 |
|  | GOTERM_CC_2 | GO:0044424~intracellular part | 0.004843 |
|  | GOTERM_BP_4 | GO:0051252~regulation of RNA metabolic process | 0.004864 |
|  | GOTERM_CC_3 | GO:0044424~intracellular part | 0.004907 |
|  | GOTERM_MF_2 | GO:0005515~protein binding | 0.004912 |
|  | GOTERM_BP_4 | GO:0010605~negative regulation of macromolecule metabolic process | 0.005107 |
|  | GOTERM_BP_2 | GO:0009892~negative regulation of metabolic process | 0.005195 |
|  | GOTERM_BP_3 | GO:0009892~negative regulation of metabolic process | 0.006027 |
|  | GOTERM_BP_3 | GO:0051276~chromosome organization | 0.006112 |
|  | GOTERM_BP_5 | GO:0031324~negative regulation of cellular metabolic process | 0.006308 |
|  | GOTERM_CC_3 | GO:0043231~intracellular membrane-bounded organelle | 0.006406 |
|  | GOTERM_BP_4 | GO:0051254~positive regulation of RNA metabolic process | 0.006406 |
|  | GOTERM_CC_2 | GO:0043227~membrane-bounded organelle | 0.00642 |
|  | GOTERM_BP_5 | GO:0006355~regulation of transcription, DNA-dependent | 0.006508 |
|  | GOTERM_BP_4 | GO:0009892~negative regulation of metabolic process | 0.006603 |
|  | GOTERM_BP_5 | GO:0010605~negative regulation of macromolecule metabolic process | 0.00684 |
|  | GOTERM_CC_4 | GO:0005654~nucleoplasm | 0.007051 |
|  | GOTERM_BP_5 | GO:0051252~regulation of RNA metabolic process | 0.007462 |
|  | GOTERM_CC_5 | GO:0005654~nucleoplasm | 0.007556 |
|  | GOTERM_CC_3 | GO:0070013~intracellular organelle lumen | 0.007593 |
|  | GOTERM_CC_1 | GO:0031974~membrane-enclosed lumen | 0.00769 |
|  | GOTERM_MF_4 | GO:0051427~hormone receptor binding | 0.007819 |
|  | GOTERM_BP_5 | GO:0045893~positive regulation of transcription, DNA-dependent | 0.007923 |
|  | GOTERM_BP_5 | GO:0051254~positive regulation of RNA metabolic process | 0.008157 |
|  | GOTERM_MF_2 | GO:0016564~transcription repressor activity | 0.008357 |
|  | GOTERM_CC_2 | GO:0043233~organelle lumen | 0.008563 |
|  | GOTERM_CC_3 | GO:0043233~organelle lumen | 0.008597 |
|  | GOTERM_CC_2 | GO:0005622~intracellular | 0.008971 |
|  | GOTERM_CC_3 | GO:0005622~intracellular | 0.009087 |
|  | GOTERM_CC_4 | GO:0070013~intracellular organelle lumen | 0.009489 |
|  | GOTERM_CC_4 | GO:0044424~intracellular part | 0.010246 |
|  | GOTERM_CC_5 | GO:0070013~intracellular organelle lumen | 0.010374 |
|  | GOTERM_CC_4 | GO:0043231~intracellular membrane-bounded organelle | 0.010943 |
|  | GOTERM_BP_1 | GO:0008152~metabolic process | 0.010955 |
|  | GOTERM_MF_3 | GO:0003677~DNA binding | 0.011945 |
|  | GOTERM_BP_4 | GO:0010628~positive regulation of gene expression | 0.012315 |
|  | GOTERM_CC_5 | GO:0031430~M band | 0.013028 |
|  | GOTERM_CC_5 | GO:0043231~intracellular membrane-bounded organelle | 0.01353 |
|  | GOTERM_BP_5 | GO:0045941~positive regulation of transcription | 0.01408 |
|  | GOTERM_BP_2 | GO:0048522~positive regulation of cellular process | 0.015157 |
|  | GOTERM_BP_5 | GO:0010628~positive regulation of gene expression | 0.015562 |
|  | GOTERM_BP_4 | GO:0045935~positive regulation of nucleobase, nucleoside, nucleotide and nucleic acid metabolic process | 0.015679 |
|  | GOTERM_BP_3 | GO:0051173~positive regulation of nitrogen compound metabolic process | 0.016207 |
|  | GOTERM_CC_2 | GO:0043234~protein complex | 0.016932 |
|  | GOTERM_BP_4 | GO:0051173~positive regulation of nitrogen compound metabolic process | 0.017426 |
|  | GOTERM_BP_3 | GO:0048522~positive regulation of cellular process | 0.018199 |
|  | GOTERM_BP_4 | GO:0010557~positive regulation of macromolecule biosynthetic process | 0.018344 |
|  | GOTERM_BP_4 | GO:0051253~negative regulation of RNA metabolic process | 0.018956 |
|  | GOTERM_BP_1 | GO:0009987~cellular process | 0.019051 |
|  | GOTERM_BP_5 | GO:0045935~positive regulation of nucleobase, nucleoside, nucleotide and nucleic acid metabolic process | 0.019749 |
|  | GOTERM_BP_4 | GO:0048522~positive regulation of cellular process | 0.020348 |
|  | GOTERM_BP_3 | GO:0009891~positive regulation of biosynthetic process | 0.020883 |
|  | GOTERM_BP_4 | GO:0031328~positive regulation of cellular biosynthetic process | 0.021383 |
|  | GOTERM_BP_5 | GO:0045892~negative regulation of transcription, DNA-dependent | 0.021759 |
|  | GOTERM_BP_5 | GO:0051173~positive regulation of nitrogen compound metabolic process | 0.021916 |
|  | GOTERM_BP_4 | GO:0009891~positive regulation of biosynthetic process | 0.022427 |
|  | GOTERM_BP_1 | GO:0016043~cellular component organization | 0.022459 |
|  | GOTERM_BP_5 | GO:0051253~negative regulation of RNA metabolic process | 0.022731 |
|  | GOTERM_CC_5 | GO:0031672~A band | 0.023049 |
|  | GOTERM_BP_5 | GO:0010557~positive regulation of macromolecule biosynthetic process | 0.023053 |
|  | GOTERM_BP_1 | GO:0022414~reproductive process | 0.024553 |
|  | GOTERM_BP_2 | GO:0048518~positive regulation of biological process | 0.024827 |
|  | GOTERM_BP_1 | GO:0000003~reproduction | 0.025083 |
|  | GOTERM_BP_2 | GO:0022414~reproductive process | 0.025166 |
|  | GOTERM_BP_5 | GO:0031328~positive regulation of cellular biosynthetic process | 0.026811 |
|  | GOTERM_BP_5 | GO:0009891~positive regulation of biosynthetic process | 0.028099 |
|  | GOTERM_BP_3 | GO:0048518~positive regulation of biological process | 0.029603 |
|  | GOTERM_BP_2 | GO:0048523~negative regulation of cellular process | 0.031583 |
|  | GOTERM_BP_3 | GO:0048523~negative regulation of cellular process | 0.036793 |
|  | GOTERM_BP_4 | GO:0048523~negative regulation of cellular process | 0.040383 |
|  | GOTERM_BP_3 | GO:0010604~positive regulation of macromolecule metabolic process | 0.040987 |
|  | GOTERM_CC_1 | GO:0032991~macromolecular complex | 0.041061 |
|  | GOTERM_MF_2 | GO:0003702~RNA polymerase II transcription factor activity | 0.042191 |
|  | GOTERM_BP_4 | GO:0010604~positive regulation of macromolecule metabolic process | 0.043857 |
|  | GOTERM_BP_2 | GO:0006996~organelle organization | 0.043993 |
|  | GOTERM_BP_3 | GO:0031325~positive regulation of cellular metabolic process | 0.044517 |
|  | GOTERM_BP_2 | GO:0009893~positive regulation of metabolic process | 0.045977 |
|  | GOTERM_BP_2 | GO:0048519~negative regulation of biological process | 0.04602 |
|  | GOTERM_BP_4 | GO:0031325~positive regulation of cellular metabolic process | 0.04761 |
| 6410 | GOTERM_BP_5 | GO:0051345~positive regulation of hydrolase activity | 0.002109 |
|  | GOTERM_BP_5 | GO:0006919~activation of caspase activity | 0.002771 |
|  | GOTERM_MF_3 | GO:0045569~TRAIL binding | 0.00291 |
|  | GOTERM_CC_1 | GO:0044422~organelle part | 0.00301 |
|  | GOTERM_CC_2 | GO:0044446~intracellular organelle part | 0.004203 |
|  | GOTERM_CC_3 | GO:0044446~intracellular organelle part | 0.004233 |
|  | GOTERM_CC_2 | GO:0044422~organelle part | 0.004467 |
|  | GOTERM_BP_4 | GO:0043085~positive regulation of catalytic activity | 0.005797 |
|  | GOTERM_CC_4 | GO:0044446~intracellular organelle part | 0.006342 |
|  | GOTERM_BP_5 | GO:0052547~regulation of peptidase activity | 0.006883 |
|  | GOTERM_CC_5 | GO:0044446~intracellular organelle part | 0.007446 |
|  | GOTERM_BP_3 | GO:0044093~positive regulation of molecular function | 0.009867 |
|  | GOTERM_BP_4 | GO:0051336~regulation of hydrolase activity | 0.011861 |
|  | GOTERM_CC_3 | GO:0005737~cytoplasm | 0.023052 |
|  | GOTERM_MF_3 | GO:0008656~caspase activator activity | 0.023054 |
|  | GOTERM_MF_5 | GO:0008656~caspase activator activity | 0.023849 |
|  | GOTERM_CC_1 | GO:0043226~organelle | 0.025826 |
|  | GOTERM_CC_3 | GO:0030530~heterogeneous nuclear ribonucleoprotein complex | 0.028545 |
|  | GOTERM_CC_3 | GO:0044428~nuclear part | 0.028809 |
|  | GOTERM_CC_4 | GO:0030530~heterogeneous nuclear ribonucleoprotein complex | 0.029726 |
|  | GOTERM_CC_5 | GO:0030530~heterogeneous nuclear ribonucleoprotein complex | 0.030216 |
|  | GOTERM_MF_3 | GO:0016504~peptidase activator activity | 0.032981 |
|  | GOTERM_BP_3 | GO:0050790~regulation of catalytic activity | 0.033475 |
|  | GOTERM_CC_4 | GO:0044428~nuclear part | 0.03515 |
|  | GOTERM_CC_5 | GO:0016604~nuclear body | 0.03649 |
|  | GOTERM_CC_4 | GO:0005737~cytoplasm | 0.036783 |
|  | GOTERM_CC_5 | GO:0044428~nuclear part | 0.038045 |
|  | GOTERM_MF_2 | GO:0000166~nucleotide binding | 0.038454 |
|  | GOTERM_CC_2 | GO:0012505~endomembrane system | 0.041392 |
|  | GOTERM_CC_3 | GO:0012505~endomembrane system | 0.041485 |
|  | GOTERM_MF_2 | GO:0043028~caspase regulator activity | 0.042192 |
|  | GOTERM_CC_2 | GO:0043229~intracellular organelle | 0.042319 |
|  | GOTERM_CC_3 | GO:0043229~intracellular organelle | 0.042707 |
|  | GOTERM_CC_5 | GO:0005737~cytoplasm | 0.044161 |
|  | GOTERM_BP_4 | GO:0006796~phosphate metabolic process | 0.046987 |
|  | GOTERM_BP_5 | GO:0051347~positive regulation of transferase activity | 0.047303 |
|  | GOTERM_BP_3 | GO:0033554~cellular response to stress | 0.049496 |
| 6433 | GOTERM_BP_2 | GO:0006955~immune response | 1.59E-07 |
|  | GOTERM_MF_5 | GO:0016175~superoxide-generating NADPH oxidase activity | 5.24E-06 |
|  | GOTERM_CC_5 | GO:0043020~NADPH oxidase complex | 1.79E-05 |
|  | GOTERM_CC_4 | GO:0043020~NADPH oxidase complex | 1.89E-05 |
|  | GOTERM_CC_3 | GO:0043020~NADPH oxidase complex | 1.90E-05 |
|  | GOTERM_BP_1 | GO:0002376~immune system process | 3.24E-05 |
|  | GOTERM_MF_4 | GO:0050664~oxidoreductase activity, acting on NADH or NADPH, with oxygen as acceptor | 3.79E-05 |
|  | GOTERM_CC_5 | GO:0043231~intracellular membrane-bounded organelle | 4.03E-05 |
|  | GOTERM_MF_2 | GO:0005515~protein binding | 4.24E-05 |
|  | GOTERM_CC_1 | GO:0005576~extracellular region | 5.64E-05 |
|  | GOTERM_BP_3 | GO:0006800~oxygen and reactive oxygen species metabolic process | 6.33E-05 |
|  | GOTERM_BP_5 | GO:0042554~superoxide anion generation | 6.46E-05 |
|  | GOTERM_CC_1 | GO:0044421~extracellular region part | 7.20E-05 |
|  | GOTERM_CC_2 | GO:0044421~extracellular region part | 7.79E-05 |
|  | GOTERM_BP_2 | GO:0045730~respiratory burst | 8.34E-05 |
|  | GOTERM_CC_4 | GO:0043231~intracellular membrane-bounded organelle | 1.23E-04 |
|  | GOTERM_BP_1 | GO:0050896~response to stimulus | 1.74E-04 |
|  | GOTERM_CC_3 | GO:0043231~intracellular membrane-bounded organelle | 2.06E-04 |
|  | GOTERM_CC_2 | GO:0043227~membrane-bounded organelle | 2.07E-04 |
|  | GOTERM_BP_4 | GO:0006801~superoxide metabolic process | 4.50E-04 |
|  | GOTERM_BP_4 | GO:0042743~hydrogen peroxide metabolic process | 4.50E-04 |
|  | GOTERM_CC_5 | GO:0042612~MHC class I protein complex | 6.43E-04 |
|  | GOTERM_CC_4 | GO:0042612~MHC class I protein complex | 6.76E-04 |
|  | GOTERM_BP_4 | GO:0048523~negative regulation of cellular process | 7.04E-04 |
|  | GOTERM_MF_3 | GO:0016798~hydrolase activity, acting on glycosyl bonds | 7.48E-04 |
|  | GOTERM_CC_5 | GO:0043229~intracellular organelle | 8.48E-04 |
|  | GOTERM_BP_3 | GO:0048523~negative regulation of cellular process | 0.001238 |
|  | GOTERM_BP_4 | GO:0031667~response to nutrient levels | 0.001243 |
|  | GOTERM_BP_2 | GO:0009605~response to external stimulus | 0.00125 |
|  | GOTERM_BP_3 | GO:0048519~negative regulation of biological process | 0.001583 |
|  | GOTERM_BP_2 | GO:0006950~response to stress | 0.001817 |
|  | GOTERM_CC_4 | GO:0043229~intracellular organelle | 0.002256 |
|  | GOTERM_BP_2 | GO:0019882~antigen processing and presentation | 0.002463 |
|  | GOTERM_BP_3 | GO:0009991~response to extracellular stimulus | 0.00266 |
|  | GOTERM_CC_1 | GO:0043226~organelle | 0.003205 |
|  | GOTERM_CC_2 | GO:0043229~intracellular organelle | 0.003295 |
|  | GOTERM_CC_3 | GO:0043229~intracellular organelle | 0.003377 |
|  | GOTERM_CC_2 | GO:0005615~extracellular space | 0.003427 |
|  | GOTERM_CC_3 | GO:0005615~extracellular space | 0.003446 |
|  | GOTERM_CC_2 | GO:0043233~organelle lumen | 0.003448 |
|  | GOTERM_BP_2 | GO:0048523~negative regulation of cellular process | 0.003452 |
|  | GOTERM_CC_3 | GO:0043233~organelle lumen | 0.003478 |
|  | GOTERM_CC_1 | GO:0031974~membrane-enclosed lumen | 0.004024 |
|  | GOTERM_BP_2 | GO:0048519~negative regulation of biological process | 0.004499 |
|  | GOTERM_MF_2 | GO:0003823~antigen binding | 0.004755 |
|  | GOTERM_CC_5 | GO:0042611~MHC protein complex | 0.00506 |
|  | GOTERM_CC_4 | GO:0042611~MHC protein complex | 0.005307 |
|  | GOTERM_CC_3 | GO:0042611~MHC protein complex | 0.005341 |
|  | GOTERM_CC_5 | GO:0031410~cytoplasmic vesicle | 0.005621 |
|  | GOTERM_CC_2 | GO:0031012~extracellular matrix | 0.005667 |
|  | GOTERM_CC_3 | GO:0031012~extracellular matrix | 0.005689 |
|  | GOTERM_BP_4 | GO:0008285~negative regulation of cell proliferation | 0.006265 |
|  | GOTERM_CC_4 | GO:0031410~cytoplasmic vesicle | 0.006357 |
|  | GOTERM_CC_3 | GO:0031410~cytoplasmic vesicle | 0.006497 |
|  | GOTERM_BP_5 | GO:0008285~negative regulation of cell proliferation | 0.007341 |
|  | GOTERM_BP_3 | GO:0008285~negative regulation of cell proliferation | 0.007747 |
|  | GOTERM_CC_2 | GO:0031982~vesicle | 0.008621 |
|  | GOTERM_CC_5 | GO:0000323~lytic vacuole | 0.009376 |
|  | GOTERM_BP_4 | GO:0045087~innate immune response | 0.009866 |
|  | GOTERM_CC_5 | GO:0070013~intracellular organelle lumen | 0.009909 |
|  | GOTERM_BP_3 | GO:0045087~innate immune response | 0.011284 |
|  | GOTERM_BP_5 | GO:0007584~response to nutrient | 0.011487 |
|  | GOTERM_CC_5 | GO:0044459~plasma membrane part | 0.011553 |
|  | GOTERM_BP_3 | GO:0007584~response to nutrient | 0.011847 |
|  | GOTERM_CC_4 | GO:0070013~intracellular organelle lumen | 0.011966 |
|  | GOTERM_CC_5 | GO:0005887~integral to plasma membrane | 0.012434 |
|  | GOTERM_CC_3 | GO:0070013~intracellular organelle lumen | 0.012444 |
|  | GOTERM_BP_3 | GO:0000387~spliceosomal snRNP biogenesis | 0.013387 |
|  | GOTERM_CC_5 | GO:0005834~heterotrimeric G-protein complex | 0.013421 |
|  | GOTERM_BP_3 | GO:0006959~humoral immune response | 0.013609 |
|  | GOTERM_CC_3 | GO:0005834~heterotrimeric G-protein complex | 0.013919 |
|  | GOTERM_BP_4 | GO:0006954~inflammatory response | 0.01404 |
|  | GOTERM_CC_4 | GO:0044459~plasma membrane part | 0.014221 |
|  | GOTERM_MF_3 | GO:0016651~oxidoreductase activity, acting on NADH or NADPH | 0.014341 |
|  | GOTERM_CC_4 | GO:0005578~proteinaceous extracellular matrix | 0.014568 |
|  | GOTERM_CC_3 | GO:0005578~proteinaceous extracellular matrix | 0.014743 |
|  | GOTERM_CC_3 | GO:0044459~plasma membrane part | 0.014869 |
|  | GOTERM_CC_5 | GO:0031226~intrinsic to plasma membrane | 0.014912 |
|  | GOTERM_BP_3 | GO:0006952~defense response | 0.015307 |
|  | GOTERM_BP_2 | GO:0019748~secondary metabolic process | 0.016681 |
|  | GOTERM_CC_4 | GO:0031226~intrinsic to plasma membrane | 0.017224 |
|  | GOTERM_MF_3 | GO:0046983~protein dimerization activity | 0.01772 |
|  | GOTERM_MF_2 | GO:0048037~cofactor binding | 0.017776 |
|  | GOTERM_CC_5 | GO:0016023~cytoplasmic membrane-bounded vesicle | 0.018902 |
|  | GOTERM_CC_5 | GO:0005773~vacuole | 0.018937 |
|  | GOTERM_MF_1 | GO:0005488~binding | 0.019183 |
|  | GOTERM_CC_4 | GO:0005773~vacuole | 0.020245 |
|  | GOTERM_BP_4 | GO:0042127~regulation of cell proliferation | 0.020377 |
|  | GOTERM_CC_4 | GO:0016023~cytoplasmic membrane-bounded vesicle | 0.020767 |
|  | GOTERM_MF_4 | GO:0004553~hydrolase activity, hydrolyzing O-glycosyl compounds | 0.021251 |
|  | GOTERM_BP_2 | GO:0007586~digestion | 0.02418 |
|  | GOTERM_CC_5 | GO:0030141~secretory granule | 0.024294 |
|  | GOTERM_BP_4 | GO:0006769~nicotinamide metabolic process | 0.024472 |
|  | GOTERM_CC_3 | GO:0031988~membrane-bounded vesicle | 0.024969 |
|  | GOTERM_MF_3 | GO:0050662~coenzyme binding | 0.025034 |
|  | GOTERM_BP_3 | GO:0042127~regulation of cell proliferation | 0.026118 |
|  | GOTERM_BP_4 | GO:0042445~hormone metabolic process | 0.026609 |
|  | GOTERM_BP_3 | GO:0009820~alkaloid metabolic process | 0.027549 |
|  | GOTERM_CC_5 | GO:0044439~peroxisomal part | 0.028255 |
|  | GOTERM_CC_5 | GO:0044438~microbody part | 0.028255 |
|  | GOTERM_BP_5 | GO:0042594~response to starvation | 0.028373 |
|  | GOTERM_BP_5 | GO:0019362~pyridine nucleotide metabolic process | 0.028373 |
|  | GOTERM_BP_3 | GO:0042594~response to starvation | 0.02881 |
|  | GOTERM_CC_4 | GO:0044438~microbody part | 0.029157 |
|  | GOTERM_CC_4 | GO:0044439~peroxisomal part | 0.029157 |
|  | GOTERM_CC_3 | GO:0044438~microbody part | 0.029271 |
|  | GOTERM_BP_4 | GO:0031324~negative regulation of cellular metabolic process | 0.030025 |
|  | GOTERM_BP_2 | GO:0033036~macromolecule localization | 0.030318 |
|  | GOTERM_MF_5 | GO:0004448~isocitrate dehydrogenase activity | 0.03222 |
|  | GOTERM_MF_5 | GO:0004994~somatostatin receptor activity | 0.03222 |
|  | GOTERM_MF_3 | GO:0051082~unfolded protein binding | 0.033554 |
|  | GOTERM_BP_5 | GO:0002791~regulation of peptide secretion | 0.033566 |
|  | GOTERM_MF_3 | GO:0051287~NAD or NADH binding | 0.033576 |
|  | GOTERM_BP_3 | GO:0006730~one-carbon metabolic process | 0.033846 |
|  | GOTERM_BP_3 | GO:0002682~regulation of immune system process | 0.034759 |
|  | GOTERM_MF_4 | GO:0051287~NAD or NADH binding | 0.035123 |
|  | GOTERM_BP_5 | GO:0031324~negative regulation of cellular metabolic process | 0.035423 |
|  | GOTERM_BP_4 | GO:0045934~negative regulation of nucleobase, nucleoside, nucleotide and nucleic acid metabolic process | 0.035665 |
|  | GOTERM_BP_4 | GO:0048878~chemical homeostasis | 0.035665 |
|  | GOTERM_BP_2 | GO:0042445~hormone metabolic process | 0.035719 |
|  | GOTERM_BP_5 | GO:0006140~regulation of nucleotide metabolic process | 0.036184 |
|  | GOTERM_BP_5 | GO:0034384~high-density lipoprotein particle clearance | 0.037009 |
|  | GOTERM_CC_5 | GO:0019897~extrinsic to plasma membrane | 0.037298 |
|  | GOTERM_BP_3 | GO:0031324~negative regulation of cellular metabolic process | 0.037433 |
|  | GOTERM_BP_4 | GO:0051172~negative regulation of nitrogen compound metabolic process | 0.037953 |
|  | GOTERM_CC_4 | GO:0019897~extrinsic to plasma membrane | 0.03847 |
|  | GOTERM_CC_5 | GO:0000775~chromosome, centromeric region | 0.040124 |
|  | GOTERM_CC_1 | GO:0032991~macromolecular complex | 0.04018 |
|  | GOTERM_BP_4 | GO:0044255~cellular lipid metabolic process | 0.040338 |
|  | GOTERM_BP_4 | GO:0015031~protein transport | 0.040902 |
|  | GOTERM_BP_5 | GO:0045934~negative regulation of nucleobase, nucleoside, nucleotide and nucleic acid metabolic process | 0.040941 |
|  | GOTERM_BP_4 | GO:0050665~hydrogen peroxide biosynthetic process | 0.041751 |
|  | GOTERM_MF_4 | GO:0046982~protein heterodimerization activity | 0.041785 |
|  | GOTERM_CC_4 | GO:0000775~chromosome, centromeric region | 0.041868 |
|  | GOTERM_BP_5 | GO:0006733~oxidoreduction coenzyme metabolic process | 0.041987 |
|  | GOTERM_BP_4 | GO:0045184~establishment of protein localization | 0.042947 |
|  | GOTERM_BP_5 | GO:0050665~hydrogen peroxide biosynthetic process | 0.043044 |
|  | GOTERM_MF_4 | GO:0008035~high-density lipoprotein binding | 0.043189 |
|  | GOTERM_BP_5 | GO:0051172~negative regulation of nitrogen compound metabolic process | 0.043528 |
|  | GOTERM_CC_5 | GO:0044444~cytoplasmic part | 0.043955 |
|  | GOTERM_CC_4 | GO:0005625~soluble fraction | 0.045138 |
|  | GOTERM_BP_4 | GO:0043603~cellular amide metabolic process | 0.045412 |
|  | GOTERM_BP_3 | GO:0051172~negative regulation of nitrogen compound metabolic process | 0.045445 |
|  | GOTERM_CC_3 | GO:0005625~soluble fraction | 0.04553 |
|  | GOTERM_BP_5 | GO:0006605~protein targeting | 0.045819 |
|  | GOTERM_BP_4 | GO:0009892~negative regulation of metabolic process | 0.046299 |
|  | GOTERM_BP_5 | GO:0051350~negative regulation of lyase activity | 0.046463 |
|  | GOTERM_BP_5 | GO:0031280~negative regulation of cyclase activity | 0.046463 |
|  | GOTERM_CC_5 | GO:0005634~nucleus | 0.047457 |
|  | GOTERM_BP_4 | GO:0051702~interaction with symbiont | 0.047573 |
|  | GOTERM_BP_2 | GO:0002682~regulation of immune system process | 0.047641 |
|  | GOTERM_BP_3 | GO:0006091~generation of precursor metabolites and energy | 0.047876 |
|  | GOTERM_BP_5 | GO:0015031~protein transport | 0.04798 |
|  | GOTERM_BP_4 | GO:0010558~negative regulation of macromolecule biosynthetic process | 0.048076 |
|  | GOTERM_BP_3 | GO:0044255~cellular lipid metabolic process | 0.048233 |
|  | GOTERM_BP_3 | GO:0001935~endothelial cell proliferation | 0.049419 |
|  | GOTERM_BP_3 | GO:0051702~interaction with symbiont | 0.049419 |
|  | GOTERM_BP_3 | GO:0009611~response to wounding | 0.049875 |
| 6454 | GOTERM_MF_5 | GO:0015057~thrombin receptor activity | 0.002509 |
|  | GOTERM_MF_4 | GO:0015057~thrombin receptor activity | 0.00297 |
|  | GOTERM_CC_5 | GO:0005887~integral to plasma membrane | 0.01789 |
|  | GOTERM_CC_4 | GO:0031226~intrinsic to plasma membrane | 0.018252 |
|  | GOTERM_CC_5 | GO:0031226~intrinsic to plasma membrane | 0.019302 |
|  | GOTERM_CC_4 | GO:0005874~microtubule | 0.023676 |
|  | GOTERM_BP_1 | GO:0022414~reproductive process | 0.024195 |
|  | GOTERM_CC_5 | GO:0005874~microtubule | 0.024419 |
|  | GOTERM_BP_1 | GO:0000003~reproduction | 0.024615 |
|  | GOTERM_BP_2 | GO:0022414~reproductive process | 0.024681 |
|  | GOTERM_CC_3 | GO:0044459~plasma membrane part | 0.029169 |
|  | GOTERM_BP_3 | GO:0048232~male gamete generation | 0.029596 |
|  | GOTERM_BP_5 | GO:0007283~spermatogenesis | 0.029646 |
|  | GOTERM_BP_4 | GO:0048232~male gamete generation | 0.030806 |
|  | GOTERM_BP_4 | GO:0007283~spermatogenesis | 0.030806 |
|  | GOTERM_BP_5 | GO:0030193~regulation of blood coagulation | 0.031761 |
|  | GOTERM_BP_4 | GO:0030193~regulation of blood coagulation | 0.032317 |
|  | GOTERM_CC_4 | GO:0044459~plasma membrane part | 0.034268 |
|  | GOTERM_CC_2 | GO:0005930~axoneme | 0.034308 |
|  | GOTERM_CC_3 | GO:0005930~axoneme | 0.034333 |
|  | GOTERM_CC_4 | GO:0005930~axoneme | 0.035749 |
|  | GOTERM_BP_3 | GO:0050818~regulation of coagulation | 0.035953 |
|  | GOTERM_CC_5 | GO:0005930~axoneme | 0.036336 |
|  | GOTERM_CC_5 | GO:0044459~plasma membrane part | 0.036547 |
|  | GOTERM_BP_4 | GO:0050818~regulation of coagulation | 0.036729 |
|  | GOTERM_CC_3 | GO:0019861~flagellum | 0.038444 |
|  | GOTERM_CC_4 | GO:0019861~flagellum | 0.040027 |
|  | GOTERM_BP_2 | GO:0007276~gamete generation | 0.043786 |
|  | GOTERM_BP_3 | GO:0007276~gamete generation | 0.046663 |
