# Supplementary material for: Disease Gene Interaction Pathways: A Potential Framework for How Disease Genes Associate by Disease-Risk Modules
Source: PLoS One. 2011 Sep 6;6(9):e24495. doi: 10.1371/journal.pone.0024495 (PMC3167857; doi:10.1371/journal.pone.0024495)
Supplement: Table S2 — Common GO functions shared by interacting terms in the CAD disease gene interaction pathway. (DOC) [file pone.0024495.s005.doc]

**Table S2. Common GO functions shared by interacting terms in the CAD disease gene interaction pathway.**

| **Interacting terms** | **Common GO functions** |
| --- | --- |
| 638-3866 | GOTERM_CC_3 GO:0005622~intracellular; GOTERM_CC_2 GO:0005622~intracellular; GOTERM_CC_4 GO:0044424~intracellular part; GOTERM_CC_3 GO:0044424~intracellular part; GOTERM_CC_2 GO:0044424~intracellular part; GOTERM_CC_5 GO:0043229~intracellular organelle; GOTERM_CC_4 GO:0043229~intracellular organelle; GOTERM_CC_1 GO:0043226~organelle; GOTERM_CC_5 GO:0005634~nucleus; GOTERM_CC_4 GO:0005634~nucleus; GOTERM_CC_3 GO:0043229~intracellular organelle; GOTERM_CC_2 GO:0043229~intracellular organelle; GOTERM_MF_5 GO:0046914~transition metal ion binding; GOTERM_BP_3 GO:0010467~gene expression; GOTERM_BP_2 GO:0009058~biosynthetic process; GOTERM_BP_3 GO:0044249~cellular biosynthetic process; GOTERM_CC_5 GO:0043231~intracellular membrane-bounded organelle; GOTERM_CC_4 GO:0043231~intracellular membrane-bounded organelle; GOTERM_BP_1 GO:0009987~cellular process; GOTERM_BP_4 GO:0034645~cellular macromolecule biosynthetic process; GOTERM_BP_4 GO:0006350~transcription; GOTERM_MF_1 GO:0005488~binding; GOTERM_BP_3 GO:0009059~macromolecule biosynthetic process; GOTERM_CC_3 GO:0043231~intracellular membrane-bounded organelle; GOTERM_MF_2 GO:0003676~nucleic acid binding; GOTERM_CC_2 GO:0043227~membrane-bounded organelle; GOTERM_MF_2 GO:0005515~protein binding; GOTERM_BP_3 GO:0044260~cellular macromolecule metabolic process; GOTERM_BP_2 GO:0044237~cellular metabolic process; GOTERM_BP_3 GO:0034641~cellular nitrogen compound metabolic process; GOTERM_BP_1 GO:0008152~metabolic process; GOTERM_BP_3 GO:0006139~nucleobase, nucleoside, nucleotide and nucleic acid metabolic process; GOTERM_BP_5 GO:0010468~regulation of gene expression; GOTERM_MF_4 GO:0046872~metal ion binding |
| 638-4287 | GOTERM_MF_2 GO:0005515~protein binding |
| 638-4945 | GOTERM_MF_2 GO:0005515~protein binding; GOTERM_MF_4 GO:0046872~metal ion binding |
| 638-5331 | GOTERM_MF_2 GO:0005515~protein binding |
| 638-5414 | GOTERM_CC_3 GO:0005622~intracellular; GOTERM_CC_2 GO:0005622~intracellular; GOTERM_CC_4 GO:0044424~intracellular part; GOTERM_CC_3 GO:0044424~intracellular part; GOTERM_CC_2 GO:0044424~intracellular part; GOTERM_CC_5 GO:0043229~intracellular organelle; GOTERM_CC_4 GO:0043229~intracellular organelle; GOTERM_CC_1 GO:0043226~organelle; GOTERM_CC_5 GO:0005634~nucleus; GOTERM_CC_4 GO:0005634~nucleus; GOTERM_CC_3 GO:0043229~intracellular organelle; GOTERM_CC_2 GO:0043229~intracellular organelle; GOTERM_CC_5 GO:0043232~intracellular non-membrane-bounded organelle; GOTERM_CC_4 GO:0043232~intracellular non-membrane-bounded organelle; GOTERM_CC_5 GO:0043231~intracellular membrane-bounded organelle; GOTERM_CC_4 GO:0043231~intracellular membrane-bounded organelle; GOTERM_CC_3 GO:0043232~intracellular non-membrane-bounded organelle; GOTERM_CC_2 GO:0043228~non-membrane-bounded organelle; GOTERM_BP_1 GO:0009987~cellular process; GOTERM_CC_3 GO:0043231~intracellular membrane-bounded organelle; GOTERM_MF_2 GO:0003676~nucleic acid binding; GOTERM_CC_2 GO:0043227~membrane-bounded organelle; GOTERM_MF_2 GO:0005515~protein binding; GOTERM_MF_5 GO:0016462~pyrophosphatase activity; GOTERM_MF_4 GO:0016818~hydrolase activity, acting on acid anhydrides, in phosphorus-containing anhydrides; GOTERM_MF_3 GO:0016817~hydrolase activity, acting on acid anhydrides; GOTERM_BP_3 GO:0044260~cellular macromolecule metabolic process; GOTERM_CC_1 GO:0044422~organelle part; GOTERM_CC_5 GO:0044446~intracellular organelle part; GOTERM_CC_4 GO:0044446~intracellular organelle part; GOTERM_BP_2 GO:0044237~cellular metabolic process; GOTERM_BP_3 GO:0034641~cellular nitrogen compound metabolic process; GOTERM_BP_1 GO:0008152~metabolic process; GOTERM_CC_3 GO:0044446~intracellular organelle part; GOTERM_CC_2 GO:0044446~intracellular organelle part; GOTERM_CC_2 GO:0044422~organelle part; GOTERM_BP_3 GO:0006139~nucleobase, nucleoside, nucleotide and nucleic acid metabolic process |
| 638-2437 | GOTERM_CC_3 GO:0005622~intracellular; GOTERM_CC_2 GO:0005622~intracellular; GOTERM_CC_4 GO:0044424~intracellular part; GOTERM_CC_3 GO:0044424~intracellular part; GOTERM_CC_2 GO:0044424~intracellular part; GOTERM_CC_5 GO:0043229~intracellular organelle; GOTERM_CC_4 GO:0043229~intracellular organelle; GOTERM_CC_1 GO:0043226~organelle; GOTERM_CC_5 GO:0005634~nucleus; GOTERM_CC_4 GO:0005634~nucleus; GOTERM_CC_3 GO:0043229~intracellular organelle; GOTERM_CC_2 GO:0043229~intracellular organelle; GOTERM_MF_5 GO:0046914~transition metal ion binding; GOTERM_CC_5 GO:0043232~intracellular non-membrane-bounded organelle; GOTERM_CC_4 GO:0043232~intracellular non-membrane-bounded organelle; GOTERM_CC_5 GO:0043231~intracellular membrane-bounded organelle; GOTERM_CC_4 GO:0043231~intracellular membrane-bounded organelle; GOTERM_CC_3 GO:0043232~intracellular non-membrane-bounded organelle; GOTERM_CC_2 GO:0043228~non-membrane-bounded organelle; GOTERM_BP_4 GO:0006350~transcription; GOTERM_MF_1 GO:0005488~binding; GOTERM_CC_3 GO:0043231~intracellular membrane-bounded organelle; GOTERM_CC_2 GO:0043227~membrane-bounded organelle; GOTERM_CC_1 GO:0044422~organelle part; GOTERM_MF_4 GO:0046872~metal ion binding |
| 638-6171 | GOTERM_CC_3 GO:0005622~intracellular; GOTERM_CC_2 GO:0005622~intracellular; GOTERM_CC_4 GO:0044424~intracellular part; GOTERM_CC_3 GO:0044424~intracellular part; GOTERM_CC_2 GO:0044424~intracellular part; GOTERM_CC_5 GO:0043229~intracellular organelle; GOTERM_CC_4 GO:0043229~intracellular organelle; GOTERM_CC_1 GO:0043226~organelle; GOTERM_CC_5 GO:0005634~nucleus; GOTERM_CC_4 GO:0005634~nucleus; GOTERM_CC_3 GO:0043229~intracellular organelle; GOTERM_CC_2 GO:0043229~intracellular organelle; GOTERM_BP_3 GO:0010467~gene expression; GOTERM_BP_2 GO:0009058~biosynthetic process; GOTERM_BP_3 GO:0044249~cellular biosynthetic process; GOTERM_CC_5 GO:0043231~intracellular membrane-bounded organelle; GOTERM_CC_4 GO:0043231~intracellular membrane-bounded organelle; GOTERM_BP_4 GO:0034645~cellular macromolecule biosynthetic process; GOTERM_BP_4 GO:0006350~transcription; GOTERM_BP_3 GO:0009059~macromolecule biosynthetic process; GOTERM_CC_3 GO:0043231~intracellular membrane-bounded organelle; GOTERM_MF_2 GO:0003676~nucleic acid binding; GOTERM_CC_2 GO:0043227~membrane-bounded organelle; GOTERM_MF_2 GO:0005515~protein binding; GOTERM_BP_3 GO:0044260~cellular macromolecule metabolic process; GOTERM_BP_2 GO:0044237~cellular metabolic process; GOTERM_BP_3 GO:0034641~cellular nitrogen compound metabolic process; GOTERM_BP_1 GO:0008152~metabolic process; GOTERM_BP_3 GO:0006139~nucleobase, nucleoside, nucleotide and nucleic acid metabolic process; GOTERM_BP_5 GO:0010468~regulation of gene expression |
| 638-6126 | GOTERM_MF_2 GO:0005515~protein binding; GOTERM_MF_4 GO:0046872~metal ion binding |
| 638-6320 | GOTERM_MF_2 GO:0005515~protein binding |
| 638-5975 | GOTERM_CC_3 GO:0005622~intracellular; GOTERM_CC_2 GO:0005622~intracellular; GOTERM_CC_4 GO:0044424~intracellular part; GOTERM_CC_3 GO:0044424~intracellular part; GOTERM_CC_2 GO:0044424~intracellular part; GOTERM_MF_1 GO:0005488~binding |
| 638-4958 | GOTERM_MF_2 GO:0005515~protein binding |
| 638-6433 | GOTERM_CC_5 GO:0043229~intracellular organelle; GOTERM_CC_4 GO:0043229~intracellular organelle; GOTERM_CC_1 GO:0043226~organelle; GOTERM_CC_5 GO:0005634~nucleus; GOTERM_CC_3 GO:0043229~intracellular organelle; GOTERM_CC_2 GO:0043229~intracellular organelle; GOTERM_CC_5 GO:0043231~intracellular membrane-bounded organelle; GOTERM_CC_4 GO:0043231~intracellular membrane-bounded organelle; GOTERM_MF_1 GO:0005488~binding; GOTERM_CC_3 GO:0043231~intracellular membrane-bounded organelle; GOTERM_CC_2 GO:0043227~membrane-bounded organelle; GOTERM_MF_2 GO:0005515~protein binding |
| 638-6116 | GOTERM_BP_3 GO:0010467~gene expression; GOTERM_MF_1 GO:0005488~binding; GOTERM_MF_2 GO:0005515~protein binding; GOTERM_BP_3 GO:0044260~cellular macromolecule metabolic process |
| 3866-3903 | GOTERM_MF_3 GO:0004872~receptor activity; GOTERM_MF_1 GO:0060089~molecular transducer activity; GOTERM_MF_2 GO:0004871~signal transducer activity; GOTERM_BP_2 GO:0048522~positive regulation of cellular process; GOTERM_BP_3 GO:0048522~positive regulation of cellular process; GOTERM_BP_4 GO:0048522~positive regulation of cellular process; GOTERM_BP_3 GO:0010604~positive regulation of macromolecule metabolic process; GOTERM_BP_4 GO:0010604~positive regulation of macromolecule metabolic process; GOTERM_BP_2 GO:0048518~positive regulation of biological process; GOTERM_BP_2 GO:0009893~positive regulation of metabolic process; GOTERM_BP_3 GO:0080090~regulation of primary metabolic process; GOTERM_BP_4 GO:0010557~positive regulation of macromolecule biosynthetic process; GOTERM_BP_3 GO:0009893~positive regulation of metabolic process; GOTERM_BP_3 GO:0048518~positive regulation of biological process; GOTERM_BP_5 GO:0010604~positive regulation of macromolecule metabolic process; GOTERM_BP_4 GO:0080090~regulation of primary metabolic process; GOTERM_BP_4 GO:0009893~positive regulation of metabolic process; GOTERM_BP_3 GO:0009891~positive regulation of biosynthetic process; GOTERM_BP_4 GO:0031328~positive regulation of cellular biosynthetic process; GOTERM_BP_4 GO:0009891~positive regulation of biosynthetic process; GOTERM_BP_5 GO:0010557~positive regulation of macromolecule biosynthetic process; GOTERM_BP_3 GO:0031323~regulation of cellular metabolic process; GOTERM_BP_2 GO:0019222~regulation of metabolic process; GOTERM_BP_4 GO:0031323~regulation of cellular metabolic process; GOTERM_BP_5 GO:0031328~positive regulation of cellular biosynthetic process; GOTERM_BP_5 GO:0009891~positive regulation of biosynthetic process; GOTERM_BP_3 GO:0019222~regulation of metabolic process; GOTERM_BP_3 GO:0031325~positive regulation of cellular metabolic process; GOTERM_BP_4 GO:0031325~positive regulation of cellular metabolic process; GOTERM_BP_5 GO:0031325~positive regulation of cellular metabolic process; GOTERM_BP_2 GO:0042221~response to chemical stimulus; GOTERM_BP_2 GO:0048519~negative regulation of biological process; GOTERM_BP_3 GO:0048519~negative regulation of biological process; GOTERM_BP_2 GO:0048523~negative regulation of cellular process; GOTERM_BP_3 GO:0048523~negative regulation of cellular process; GOTERM_BP_4 GO:0048523~negative regulation of cellular process; GOTERM_BP_3 GO:0010033~response to organic substance; GOTERM_BP_3 GO:0007165~signal transduction; GOTERM_BP_4 GO:0007165~signal transduction; GOTERM_BP_3 GO:0009725~response to hormone stimulus; GOTERM_BP_2 GO:0050794~regulation of cellular process; GOTERM_BP_2 GO:0009719~response to endogenous stimulus; GOTERM_BP_3 GO:0050794~regulation of cellular process; GOTERM_BP_3 GO:0048513~organ development; GOTERM_BP_2 GO:0050789~regulation of biological process; GOTERM_BP_4 GO:0019216~regulation of lipid metabolic process; GOTERM_BP_4 GO:0048513~organ development; GOTERM_BP_5 GO:0019216~regulation of lipid metabolic process; GOTERM_BP_1 GO:0065007~biological regulation; GOTERM_BP_4 GO:0010565~regulation of cellular ketone metabolic process; GOTERM_BP_5 GO:0010565~regulation of cellular ketone metabolic process; GOTERM_BP_4 GO:0007242~intracellular signaling cascade; GOTERM_BP_5 GO:0007242~intracellular signaling cascade; GOTERM_BP_4 GO:0045923~positive regulation of fatty acid metabolic process; GOTERM_BP_5 GO:0032868~response to insulin stimulus; GOTERM_BP_5 GO:0045923~positive regulation of fatty acid metabolic process; GOTERM_MF_2 GO:0005515~protein binding; GOTERM_BP_1 GO:0050896~response to stimulus; GOTERM_BP_4 GO:0043434~response to peptide hormone stimulus; GOTERM_BP_3 GO:0045834~positive regulation of lipid metabolic process; GOTERM_BP_4 GO:0045834~positive regulation of lipid metabolic process; GOTERM_BP_4 GO:0048545~response to steroid hormone stimulus; GOTERM_BP_5 GO:0045834~positive regulation of lipid metabolic process; GOTERM_BP_3 GO:0010942~positive regulation of cell death; GOTERM_BP_4 GO:0043068~positive regulation of programmed cell death; GOTERM_BP_4 GO:0010942~positive regulation of cell death; GOTERM_BP_5 GO:0019217~regulation of fatty acid metabolic process; GOTERM_BP_3 GO:0010941~regulation of cell death; GOTERM_BP_4 GO:0043067~regulation of programmed cell death; GOTERM_BP_4 GO:0010941~regulation of cell death; GOTERM_BP_5 GO:0042981~regulation of apoptosis; GOTERM_BP_5 GO:0043067~regulation of programmed cell death; GOTERM_MF_3 GO:0005102~receptor binding; GOTERM_BP_5 GO:0043627~response to estrogen stimulus; GOTERM_BP_4 GO:0046321~positive regulation of fatty acid oxidation; GOTERM_BP_5 GO:0046321~positive regulation of fatty acid oxidation; GOTERM_BP_3 GO:0032870~cellular response to hormone stimulus; GOTERM_BP_4 GO:0032870~cellular response to hormone stimulus; GOTERM_BP_3 GO:0042127~regulation of cell proliferation; GOTERM_BP_2 GO:0051716~cellular response to stimulus; GOTERM_BP_4 GO:0042127~regulation of cell proliferation; GOTERM_BP_2 GO:0065009~regulation of molecular function; GOTERM_BP_4 GO:0046320~regulation of fatty acid oxidation; GOTERM_BP_5 GO:0046320~regulation of fatty acid oxidation; GOTERM_BP_4 GO:0006109~regulation of carbohydrate metabolic process |
| 3866-5033 | GOTERM_MF_5 GO:0003707~steroid hormone receptor activity; GOTERM_MF_4 GO:0004879~ligand-dependent nuclear receptor activity; GOTERM_MF_4 GO:0043565~sequence-specific DNA binding; GOTERM_MF_2 GO:0003700~transcription factor activity; GOTERM_MF_4 GO:0003700~transcription factor activity; GOTERM_MF_1 GO:0030528~transcription regulator activity; GOTERM_MF_2 GO:0016563~transcription activator activity; GOTERM_BP_4 GO:0051252~regulation of RNA metabolic process; GOTERM_BP_5 GO:0006355~regulation of transcription, DNA-dependent; GOTERM_BP_5 GO:0051252~regulation of RNA metabolic process; GOTERM_BP_4 GO:0006350~transcription; GOTERM_MF_3 GO:0003677~DNA binding; GOTERM_BP_4 GO:0010628~positive regulation of gene expression; GOTERM_BP_5 GO:0010628~positive regulation of gene expression; GOTERM_BP_4 GO:0051254~positive regulation of RNA metabolic process; GOTERM_BP_5 GO:0045893~positive regulation of transcription, DNA-dependent; GOTERM_BP_5 GO:0051254~positive regulation of RNA metabolic process; GOTERM_BP_3 GO:0009059~macromolecule biosynthetic process; GOTERM_BP_3 GO:0051171~regulation of nitrogen compound metabolic process; GOTERM_BP_5 GO:0045449~regulation of transcription; GOTERM_BP_4 GO:0034645~cellular macromolecule biosynthetic process; GOTERM_BP_4 GO:0019219~regulation of nucleobase, nucleoside, nucleotide and nucleic acid metabolic process; GOTERM_BP_4 GO:0010556~regulation of macromolecule biosynthetic process; GOTERM_BP_5 GO:0030522~intracellular receptor-mediated signaling pathway; GOTERM_BP_4 GO:0051171~regulation of nitrogen compound metabolic process; GOTERM_BP_4 GO:0010468~regulation of gene expression; GOTERM_MF_2 GO:0003676~nucleic acid binding; GOTERM_BP_3 GO:0009889~regulation of biosynthetic process; GOTERM_BP_2 GO:0048522~positive regulation of cellular process; GOTERM_BP_3 GO:0010467~gene expression; GOTERM_BP_4 GO:0031326~regulation of cellular biosynthetic process; GOTERM_BP_4 GO:0009889~regulation of biosynthetic process; GOTERM_BP_3 GO:0048522~positive regulation of cellular process; GOTERM_BP_5 GO:0019219~regulation of nucleobase, nucleoside, nucleotide and nucleic acid metabolic process; GOTERM_BP_5 GO:0010556~regulation of macromolecule biosynthetic process; GOTERM_BP_4 GO:0048522~positive regulation of cellular process; GOTERM_BP_5 GO:0010468~regulation of gene expression; GOTERM_BP_5 GO:0045941~positive regulation of transcription; GOTERM_BP_3 GO:0010604~positive regulation of macromolecule metabolic process; GOTERM_BP_4 GO:0045935~positive regulation of nucleobase, nucleoside, nucleotide and nucleic acid metabolic process; GOTERM_BP_4 GO:0010604~positive regulation of macromolecule metabolic process; GOTERM_BP_3 GO:0051173~positive regulation of nitrogen compound metabolic process; GOTERM_BP_5 GO:0031326~regulation of cellular biosynthetic process; GOTERM_BP_2 GO:0048518~positive regulation of biological process; GOTERM_BP_2 GO:0009893~positive regulation of metabolic process; GOTERM_BP_3 GO:0060255~regulation of macromolecule metabolic process; GOTERM_BP_4 GO:0051173~positive regulation of nitrogen compound metabolic process; GOTERM_BP_3 GO:0080090~regulation of primary metabolic process; GOTERM_BP_4 GO:0010557~positive regulation of macromolecule biosynthetic process; GOTERM_BP_3 GO:0009893~positive regulation of metabolic process; GOTERM_BP_3 GO:0048518~positive regulation of biological process; GOTERM_BP_4 GO:0060255~regulation of macromolecule metabolic process; GOTERM_BP_5 GO:0045935~positive regulation of nucleobase, nucleoside, nucleotide and nucleic acid metabolic process; GOTERM_BP_5 GO:0010604~positive regulation of macromolecule metabolic process; GOTERM_BP_4 GO:0080090~regulation of primary metabolic process; GOTERM_BP_4 GO:0009893~positive regulation of metabolic process; GOTERM_BP_3 GO:0009891~positive regulation of biosynthetic process; GOTERM_BP_4 GO:0031328~positive regulation of cellular biosynthetic process; GOTERM_BP_5 GO:0051173~positive regulation of nitrogen compound metabolic process; GOTERM_BP_3 GO:0006139~nucleobase, nucleoside, nucleotide and nucleic acid metabolic process; GOTERM_BP_5 GO:0016481~negative regulation of transcription; GOTERM_BP_2 GO:0009058~biosynthetic process; GOTERM_BP_4 GO:0009891~positive regulation of biosynthetic process; GOTERM_BP_5 GO:0010557~positive regulation of macromolecule biosynthetic process; GOTERM_BP_3 GO:0044249~cellular biosynthetic process; GOTERM_BP_3 GO:0031323~regulation of cellular metabolic process; GOTERM_BP_4 GO:0010629~negative regulation of gene expression; GOTERM_BP_3 GO:0051172~negative regulation of nitrogen compound metabolic process; GOTERM_BP_2 GO:0019222~regulation of metabolic process; GOTERM_BP_4 GO:0045934~negative regulation of nucleobase, nucleoside, nucleotide and nucleic acid metabolic process; GOTERM_MF_3 GO:0008134~transcription factor binding; GOTERM_BP_4 GO:0051172~negative regulation of nitrogen compound metabolic process; GOTERM_BP_4 GO:0031323~regulation of cellular metabolic process; GOTERM_BP_5 GO:0031328~positive regulation of cellular biosynthetic process; GOTERM_BP_5 GO:0009891~positive regulation of biosynthetic process; GOTERM_BP_5 GO:0010629~negative regulation of gene expression; GOTERM_BP_3 GO:0019222~regulation of metabolic process; GOTERM_BP_4 GO:0010558~negative regulation of macromolecule biosynthetic process; GOTERM_BP_5 GO:0045934~negative regulation of nucleobase, nucleoside, nucleotide and nucleic acid metabolic process; GOTERM_BP_2 GO:0006807~nitrogen compound metabolic process; GOTERM_BP_5 GO:0051172~negative regulation of nitrogen compound metabolic process; GOTERM_BP_3 GO:0034641~cellular nitrogen compound metabolic process; GOTERM_BP_3 GO:0009890~negative regulation of biosynthetic process; GOTERM_BP_4 GO:0031327~negative regulation of cellular biosynthetic process; GOTERM_BP_4 GO:0009890~negative regulation of biosynthetic process; GOTERM_BP_5 GO:0010558~negative regulation of macromolecule biosynthetic process; GOTERM_BP_5 GO:0031327~negative regulation of cellular biosynthetic process; GOTERM_BP_5 GO:0009890~negative regulation of biosynthetic process; GOTERM_BP_3 GO:0031325~positive regulation of cellular metabolic process; GOTERM_BP_4 GO:0031325~positive regulation of cellular metabolic process; GOTERM_BP_5 GO:0031325~positive regulation of cellular metabolic process; GOTERM_BP_3 GO:0031324~negative regulation of cellular metabolic process; GOTERM_BP_3 GO:0010605~negative regulation of macromolecule metabolic process; GOTERM_BP_4 GO:0031324~negative regulation of cellular metabolic process; GOTERM_BP_4 GO:0010605~negative regulation of macromolecule metabolic process; GOTERM_BP_2 GO:0009892~negative regulation of metabolic process; GOTERM_BP_3 GO:0009892~negative regulation of metabolic process; GOTERM_BP_5 GO:0031324~negative regulation of cellular metabolic process; GOTERM_BP_4 GO:0009892~negative regulation of metabolic process; GOTERM_BP_5 GO:0010605~negative regulation of macromolecule metabolic process; GOTERM_CC_4 GO:0005634~nucleus; GOTERM_CC_5 GO:0005634~nucleus; GOTERM_BP_2 GO:0048519~negative regulation of biological process; GOTERM_BP_3 GO:0044260~cellular macromolecule metabolic process; GOTERM_BP_3 GO:0048519~negative regulation of biological process; GOTERM_BP_2 GO:0043170~macromolecule metabolic process; GOTERM_BP_2 GO:0048523~negative regulation of cellular process; GOTERM_BP_3 GO:0048523~negative regulation of cellular process; GOTERM_BP_4 GO:0048523~negative regulation of cellular process; GOTERM_BP_3 GO:0007165~signal transduction; GOTERM_MF_2 GO:0003712~transcription cofactor activity; GOTERM_BP_4 GO:0007165~signal transduction; GOTERM_BP_2 GO:0044237~cellular metabolic process; GOTERM_BP_4 GO:0051253~negative regulation of RNA metabolic process; GOTERM_BP_2 GO:0050794~regulation of cellular process; GOTERM_BP_5 GO:0045892~negative regulation of transcription, DNA-dependent; GOTERM_BP_5 GO:0051253~negative regulation of RNA metabolic process; GOTERM_MF_3 GO:0003713~transcription coactivator activity; GOTERM_MF_4 GO:0051427~hormone receptor binding; GOTERM_BP_2 GO:0044238~primary metabolic process; GOTERM_MF_5 GO:0035257~nuclear hormone receptor binding; GOTERM_MF_4 GO:0003712~transcription cofactor activity; GOTERM_BP_3 GO:0050794~regulation of cellular process; GOTERM_BP_3 GO:0048513~organ development; GOTERM_BP_2 GO:0050789~regulation of biological process; GOTERM_BP_4 GO:0048513~organ development; GOTERM_CC_3 GO:0043231~intracellular membrane-bounded organelle; GOTERM_CC_2 GO:0043227~membrane-bounded organelle; GOTERM_MF_5 GO:0003713~transcription coactivator activity; GOTERM_BP_1 GO:0065007~biological regulation; GOTERM_CC_4 GO:0043231~intracellular membrane-bounded organelle; GOTERM_BP_1 GO:0008152~metabolic process; GOTERM_CC_5 GO:0043231~intracellular membrane-bounded organelle; GOTERM_CC_1 GO:0043226~organelle; GOTERM_MF_5 GO:0010843~promoter binding; GOTERM_CC_2 GO:0043229~intracellular organelle; GOTERM_CC_3 GO:0043229~intracellular organelle; GOTERM_BP_3 GO:0048731~system development; GOTERM_BP_2 GO:0048856~anatomical structure development; GOTERM_CC_4 GO:0043229~intracellular organelle; GOTERM_CC_5 GO:0043229~intracellular organelle; GOTERM_MF_2 GO:0016564~transcription repressor activity; GOTERM_MF_2 GO:0005515~protein binding; GOTERM_BP_2 GO:0007275~multicellular organismal development; GOTERM_BP_1 GO:0032502~developmental process; GOTERM_MF_3 GO:0003704~specific RNA polymerase II transcription factor activity; GOTERM_CC_2 GO:0044424~intracellular part; GOTERM_CC_3 GO:0044424~intracellular part; GOTERM_CC_2 GO:0005622~intracellular; GOTERM_CC_3 GO:0005622~intracellular; GOTERM_CC_4 GO:0044424~intracellular part; GOTERM_BP_1 GO:0009987~cellular process; GOTERM_BP_4 GO:0007517~muscle organ development; GOTERM_MF_2 GO:0003702~RNA polymerase II transcription factor activity; GOTERM_BP_5 GO:0007517~muscle organ development |
| 3866-5414 | GOTERM_MF_3 GO:0003677~DNA binding; GOTERM_MF_2 GO:0003676~nucleic acid binding; GOTERM_BP_2 GO:0048522~positive regulation of cellular process; GOTERM_BP_3 GO:0048522~positive regulation of cellular process; GOTERM_BP_4 GO:0048522~positive regulation of cellular process; GOTERM_BP_4 GO:0045935~positive regulation of nucleobase, nucleoside, nucleotide and nucleic acid metabolic process; GOTERM_BP_3 GO:0051173~positive regulation of nitrogen compound metabolic process; GOTERM_BP_2 GO:0048518~positive regulation of biological process; GOTERM_BP_4 GO:0051173~positive regulation of nitrogen compound metabolic process; GOTERM_BP_3 GO:0048518~positive regulation of biological process; GOTERM_BP_5 GO:0045935~positive regulation of nucleobase, nucleoside, nucleotide and nucleic acid metabolic process; GOTERM_BP_5 GO:0051173~positive regulation of nitrogen compound metabolic process; GOTERM_BP_3 GO:0006139~nucleobase, nucleoside, nucleotide and nucleic acid metabolic process; GOTERM_BP_2 GO:0006807~nitrogen compound metabolic process; GOTERM_BP_3 GO:0034641~cellular nitrogen compound metabolic process; GOTERM_CC_4 GO:0005634~nucleus; GOTERM_CC_5 GO:0005634~nucleus; GOTERM_BP_3 GO:0044260~cellular macromolecule metabolic process; GOTERM_BP_2 GO:0043170~macromolecule metabolic process; GOTERM_BP_2 GO:0044237~cellular metabolic process; GOTERM_BP_2 GO:0044238~primary metabolic process; GOTERM_CC_3 GO:0043231~intracellular membrane-bounded organelle; GOTERM_CC_2 GO:0043227~membrane-bounded organelle; GOTERM_CC_4 GO:0043231~intracellular membrane-bounded organelle; GOTERM_BP_1 GO:0008152~metabolic process; GOTERM_CC_5 GO:0043231~intracellular membrane-bounded organelle; GOTERM_CC_1 GO:0043226~organelle; GOTERM_CC_2 GO:0043229~intracellular organelle; GOTERM_CC_3 GO:0043229~intracellular organelle; GOTERM_CC_4 GO:0043229~intracellular organelle; GOTERM_CC_5 GO:0043229~intracellular organelle; GOTERM_MF_2 GO:0005515~protein binding; GOTERM_BP_1 GO:0050896~response to stimulus; GOTERM_CC_2 GO:0044424~intracellular part; GOTERM_CC_3 GO:0044424~intracellular part; GOTERM_CC_2 GO:0005622~intracellular; GOTERM_CC_3 GO:0005622~intracellular; GOTERM_CC_4 GO:0044424~intracellular part; GOTERM_BP_1 GO:0009987~cellular process; GOTERM_MF_5 GO:0003690~double-stranded DNA binding; GOTERM_BP_2 GO:0051716~cellular response to stimulus; GOTERM_MF_4 GO:0043566~structure-specific DNA binding; GOTERM_BP_2 GO:0065008~regulation of biological quality |
| 3866-4944 | GOTERM_BP_2 GO:0048522~positive regulation of cellular process; GOTERM_BP_3 GO:0048522~positive regulation of cellular process; GOTERM_BP_4 GO:0048522~positive regulation of cellular process; GOTERM_BP_3 GO:0010604~positive regulation of macromolecule metabolic process; GOTERM_BP_4 GO:0010604~positive regulation of macromolecule metabolic process; GOTERM_BP_2 GO:0048518~positive regulation of biological process; GOTERM_BP_2 GO:0009893~positive regulation of metabolic process; GOTERM_BP_3 GO:0060255~regulation of macromolecule metabolic process; GOTERM_BP_3 GO:0080090~regulation of primary metabolic process; GOTERM_BP_3 GO:0009893~positive regulation of metabolic process; GOTERM_BP_3 GO:0048518~positive regulation of biological process; GOTERM_BP_4 GO:0060255~regulation of macromolecule metabolic process; GOTERM_BP_5 GO:0010604~positive regulation of macromolecule metabolic process; GOTERM_BP_4 GO:0009893~positive regulation of metabolic process; GOTERM_BP_3 GO:0031325~positive regulation of cellular metabolic process; GOTERM_BP_4 GO:0031325~positive regulation of cellular metabolic process; GOTERM_BP_5 GO:0031325~positive regulation of cellular metabolic process; GOTERM_BP_2 GO:0042221~response to chemical stimulus; GOTERM_BP_2 GO:0048519~negative regulation of biological process; GOTERM_BP_3 GO:0048519~negative regulation of biological process; GOTERM_BP_3 GO:0010033~response to organic substance; GOTERM_BP_1 GO:0050896~response to stimulus; GOTERM_BP_2 GO:0009605~response to external stimulus; GOTERM_MF_3 GO:0005102~receptor binding; GOTERM_BP_2 GO:0065008~regulation of biological quality |
| 3866-5982 | GOTERM_MF_2 GO:0003700~transcription factor activity; GOTERM_MF_4 GO:0003700~transcription factor activity; GOTERM_MF_1 GO:0030528~transcription regulator activity; GOTERM_BP_4 GO:0006350~transcription; GOTERM_MF_3 GO:0003677~DNA binding; GOTERM_BP_4 GO:0010556~regulation of macromolecule biosynthetic process; GOTERM_BP_2 GO:0048522~positive regulation of cellular process; GOTERM_BP_3 GO:0048522~positive regulation of cellular process; GOTERM_BP_5 GO:0010556~regulation of macromolecule biosynthetic process; GOTERM_BP_4 GO:0048522~positive regulation of cellular process; GOTERM_BP_2 GO:0048518~positive regulation of biological process; GOTERM_BP_3 GO:0060255~regulation of macromolecule metabolic process; GOTERM_BP_3 GO:0080090~regulation of primary metabolic process; GOTERM_BP_3 GO:0048518~positive regulation of biological process; GOTERM_BP_4 GO:0060255~regulation of macromolecule metabolic process; GOTERM_BP_4 GO:0080090~regulation of primary metabolic process; GOTERM_BP_3 GO:0031323~regulation of cellular metabolic process; GOTERM_BP_2 GO:0019222~regulation of metabolic process; GOTERM_BP_4 GO:0031323~regulation of cellular metabolic process; GOTERM_BP_3 GO:0019222~regulation of metabolic process; GOTERM_BP_3 GO:0031324~negative regulation of cellular metabolic process; GOTERM_BP_3 GO:0010605~negative regulation of macromolecule metabolic process; GOTERM_BP_4 GO:0031324~negative regulation of cellular metabolic process; GOTERM_BP_4 GO:0010605~negative regulation of macromolecule metabolic process; GOTERM_BP_2 GO:0009892~negative regulation of metabolic process; GOTERM_BP_3 GO:0009892~negative regulation of metabolic process; GOTERM_BP_5 GO:0031324~negative regulation of cellular metabolic process; GOTERM_BP_4 GO:0009892~negative regulation of metabolic process; GOTERM_BP_5 GO:0010605~negative regulation of macromolecule metabolic process; GOTERM_BP_2 GO:0050794~regulation of cellular process; GOTERM_BP_3 GO:0050794~regulation of cellular process; GOTERM_BP_3 GO:0048513~organ development; GOTERM_BP_2 GO:0050789~regulation of biological process; GOTERM_BP_4 GO:0048513~organ development; GOTERM_BP_1 GO:0065007~biological regulation; GOTERM_BP_4 GO:0032774~RNA biosynthetic process; GOTERM_BP_5 GO:0006351~transcription, DNA-dependent; GOTERM_BP_5 GO:0032774~RNA biosynthetic process; GOTERM_CC_1 GO:0043226~organelle; GOTERM_BP_3 GO:0009888~tissue development; GOTERM_CC_2 GO:0043229~intracellular organelle; GOTERM_CC_3 GO:0043229~intracellular organelle; GOTERM_BP_4 GO:0009888~tissue development; GOTERM_BP_3 GO:0048731~system development; GOTERM_BP_5 GO:0009888~tissue development; GOTERM_BP_2 GO:0048856~anatomical structure development; GOTERM_CC_4 GO:0043229~intracellular organelle; GOTERM_CC_5 GO:0043229~intracellular organelle; GOTERM_MF_2 GO:0005515~protein binding; GOTERM_BP_2 GO:0007275~multicellular organismal development; GOTERM_BP_1 GO:0032502~developmental process; GOTERM_BP_1 GO:0032501~multicellular organismal process; GOTERM_BP_3 GO:0042127~regulation of cell proliferation; GOTERM_BP_4 GO:0042127~regulation of cell proliferation; GOTERM_MF_1 GO:0005488~binding |
| 3866-5978 | GOTERM_BP_2 GO:0006807~nitrogen compound metabolic process; GOTERM_BP_3 GO:0034641~cellular nitrogen compound metabolic process; GOTERM_CC_4 GO:0005634~nucleus; GOTERM_CC_5 GO:0005634~nucleus; GOTERM_CC_3 GO:0043231~intracellular membrane-bounded organelle; GOTERM_CC_2 GO:0043227~membrane-bounded organelle; GOTERM_CC_4 GO:0043231~intracellular membrane-bounded organelle; GOTERM_CC_5 GO:0043231~intracellular membrane-bounded organelle; GOTERM_CC_1 GO:0043226~organelle; GOTERM_CC_2 GO:0043229~intracellular organelle; GOTERM_CC_3 GO:0043229~intracellular organelle; GOTERM_CC_4 GO:0043229~intracellular organelle; GOTERM_CC_5 GO:0043229~intracellular organelle; GOTERM_CC_2 GO:0044424~intracellular part; GOTERM_CC_3 GO:0044424~intracellular part; GOTERM_CC_2 GO:0005622~intracellular; GOTERM_CC_3 GO:0005622~intracellular; GOTERM_CC_4 GO:0044424~intracellular part |
| 3866-5949 | GOTERM_MF_3 GO:0004872~receptor activity; GOTERM_MF_1 GO:0060089~molecular transducer activity; GOTERM_MF_2 GO:0004871~signal transducer activity; GOTERM_BP_4 GO:0007242~intracellular signaling cascade; GOTERM_BP_5 GO:0007242~intracellular signaling cascade; GOTERM_MF_2 GO:0005515~protein binding; GOTERM_MF_1 GO:0005488~binding |
| 3866-2437 | GOTERM_BP_4 GO:0006350~transcription; GOTERM_MF_5 GO:0046914~transition metal ion binding; GOTERM_MF_2 GO:0043167~ion binding; GOTERM_MF_3 GO:0043169~cation binding; GOTERM_CC_4 GO:0005634~nucleus; GOTERM_MF_4 GO:0046872~metal ion binding; GOTERM_CC_5 GO:0005634~nucleus; GOTERM_CC_3 GO:0043231~intracellular membrane-bounded organelle; GOTERM_CC_2 GO:0043227~membrane-bounded organelle; GOTERM_CC_4 GO:0043231~intracellular membrane-bounded organelle; GOTERM_CC_5 GO:0043231~intracellular membrane-bounded organelle; GOTERM_CC_1 GO:0043226~organelle; GOTERM_CC_2 GO:0043229~intracellular organelle; GOTERM_CC_3 GO:0043229~intracellular organelle; GOTERM_CC_4 GO:0043229~intracellular organelle; GOTERM_CC_5 GO:0043229~intracellular organelle; GOTERM_CC_2 GO:0044424~intracellular part; GOTERM_CC_3 GO:0044424~intracellular part; GOTERM_CC_2 GO:0005622~intracellular; GOTERM_CC_3 GO:0005622~intracellular; GOTERM_CC_4 GO:0044424~intracellular part; GOTERM_MF_1 GO:0005488~binding |
| 3866-5115 | GOTERM_MF_4 GO:0043565~sequence-specific DNA binding; GOTERM_MF_1 GO:0030528~transcription regulator activity; GOTERM_MF_2 GO:0016563~transcription activator activity; GOTERM_BP_4 GO:0051252~regulation of RNA metabolic process; GOTERM_BP_5 GO:0006355~regulation of transcription, DNA-dependent; GOTERM_BP_5 GO:0051252~regulation of RNA metabolic process; GOTERM_BP_4 GO:0006350~transcription; GOTERM_MF_3 GO:0003677~DNA binding; GOTERM_BP_4 GO:0010628~positive regulation of gene expression; GOTERM_BP_5 GO:0010628~positive regulation of gene expression; GOTERM_BP_4 GO:0051254~positive regulation of RNA metabolic process; GOTERM_BP_5 GO:0045893~positive regulation of transcription, DNA-dependent; GOTERM_BP_5 GO:0051254~positive regulation of RNA metabolic process; GOTERM_BP_3 GO:0009059~macromolecule biosynthetic process; GOTERM_BP_3 GO:0051171~regulation of nitrogen compound metabolic process; GOTERM_BP_5 GO:0045449~regulation of transcription; GOTERM_BP_4 GO:0034645~cellular macromolecule biosynthetic process; GOTERM_BP_4 GO:0019219~regulation of nucleobase, nucleoside, nucleotide and nucleic acid metabolic process; GOTERM_BP_4 GO:0010556~regulation of macromolecule biosynthetic process; GOTERM_BP_4 GO:0051171~regulation of nitrogen compound metabolic process; GOTERM_BP_4 GO:0010468~regulation of gene expression; GOTERM_MF_2 GO:0003676~nucleic acid binding; GOTERM_BP_3 GO:0009889~regulation of biosynthetic process; GOTERM_BP_3 GO:0010467~gene expression; GOTERM_BP_4 GO:0031326~regulation of cellular biosynthetic process; GOTERM_BP_4 GO:0009889~regulation of biosynthetic process; GOTERM_BP_5 GO:0019219~regulation of nucleobase, nucleoside, nucleotide and nucleic acid metabolic process; GOTERM_BP_5 GO:0010556~regulation of macromolecule biosynthetic process; GOTERM_BP_5 GO:0010468~regulation of gene expression; GOTERM_BP_5 GO:0045941~positive regulation of transcription; GOTERM_BP_3 GO:0010604~positive regulation of macromolecule metabolic process; GOTERM_BP_4 GO:0045935~positive regulation of nucleobase, nucleoside, nucleotide and nucleic acid metabolic process; GOTERM_BP_4 GO:0010604~positive regulation of macromolecule metabolic process; GOTERM_BP_3 GO:0051173~positive regulation of nitrogen compound metabolic process; GOTERM_BP_5 GO:0031326~regulation of cellular biosynthetic process; GOTERM_BP_2 GO:0009893~positive regulation of metabolic process; GOTERM_BP_3 GO:0060255~regulation of macromolecule metabolic process; GOTERM_BP_4 GO:0051173~positive regulation of nitrogen compound metabolic process; GOTERM_BP_3 GO:0080090~regulation of primary metabolic process; GOTERM_BP_4 GO:0010557~positive regulation of macromolecule biosynthetic process; GOTERM_BP_4 GO:0060255~regulation of macromolecule metabolic process; GOTERM_BP_5 GO:0045935~positive regulation of nucleobase, nucleoside, nucleotide and nucleic acid metabolic process; GOTERM_BP_5 GO:0010604~positive regulation of macromolecule metabolic process; GOTERM_BP_4 GO:0080090~regulation of primary metabolic process; GOTERM_BP_3 GO:0009891~positive regulation of biosynthetic process; GOTERM_BP_4 GO:0031328~positive regulation of cellular biosynthetic process; GOTERM_BP_5 GO:0051173~positive regulation of nitrogen compound metabolic process; GOTERM_BP_3 GO:0006139~nucleobase, nucleoside, nucleotide and nucleic acid metabolic process; GOTERM_BP_2 GO:0009058~biosynthetic process; GOTERM_BP_4 GO:0009891~positive regulation of biosynthetic process; GOTERM_BP_5 GO:0010557~positive regulation of macromolecule biosynthetic process; GOTERM_BP_3 GO:0044249~cellular biosynthetic process; GOTERM_BP_3 GO:0031323~regulation of cellular metabolic process; GOTERM_BP_2 GO:0019222~regulation of metabolic process; GOTERM_BP_4 GO:0031323~regulation of cellular metabolic process; GOTERM_BP_5 GO:0031328~positive regulation of cellular biosynthetic process; GOTERM_BP_5 GO:0009891~positive regulation of biosynthetic process; GOTERM_BP_3 GO:0019222~regulation of metabolic process; GOTERM_BP_2 GO:0006807~nitrogen compound metabolic process; GOTERM_BP_3 GO:0034641~cellular nitrogen compound metabolic process; GOTERM_BP_3 GO:0031325~positive regulation of cellular metabolic process; GOTERM_BP_4 GO:0031325~positive regulation of cellular metabolic process; GOTERM_CC_4 GO:0005634~nucleus; GOTERM_CC_5 GO:0005634~nucleus; GOTERM_BP_3 GO:0044260~cellular macromolecule metabolic process; GOTERM_BP_2 GO:0043170~macromolecule metabolic process; GOTERM_BP_2 GO:0044237~cellular metabolic process; GOTERM_BP_2 GO:0044238~primary metabolic process; GOTERM_CC_3 GO:0043231~intracellular membrane-bounded organelle; GOTERM_CC_2 GO:0043227~membrane-bounded organelle; GOTERM_BP_1 GO:0065007~biological regulation; GOTERM_CC_4 GO:0043231~intracellular membrane-bounded organelle; GOTERM_BP_1 GO:0008152~metabolic process; GOTERM_CC_5 GO:0043231~intracellular membrane-bounded organelle; GOTERM_CC_1 GO:0043226~organelle; GOTERM_CC_2 GO:0043229~intracellular organelle; GOTERM_CC_3 GO:0043229~intracellular organelle; GOTERM_CC_4 GO:0043229~intracellular organelle; GOTERM_CC_5 GO:0043229~intracellular organelle; GOTERM_BP_1 GO:0009987~cellular process |
| 3866-6171 | GOTERM_MF_2 GO:0003700~transcription factor activity; GOTERM_MF_4 GO:0003700~transcription factor activity; GOTERM_MF_1 GO:0030528~transcription regulator activity; GOTERM_BP_4 GO:0051252~regulation of RNA metabolic process; GOTERM_BP_5 GO:0006355~regulation of transcription, DNA-dependent; GOTERM_BP_5 GO:0051252~regulation of RNA metabolic process; GOTERM_BP_4 GO:0006350~transcription; GOTERM_MF_3 GO:0003677~DNA binding; GOTERM_BP_3 GO:0009059~macromolecule biosynthetic process; GOTERM_BP_3 GO:0051171~regulation of nitrogen compound metabolic process; GOTERM_BP_5 GO:0045449~regulation of transcription; GOTERM_BP_4 GO:0034645~cellular macromolecule biosynthetic process; GOTERM_BP_4 GO:0019219~regulation of nucleobase, nucleoside, nucleotide and nucleic acid metabolic process; GOTERM_BP_4 GO:0010556~regulation of macromolecule biosynthetic process; GOTERM_BP_4 GO:0051171~regulation of nitrogen compound metabolic process; GOTERM_BP_4 GO:0010468~regulation of gene expression; GOTERM_MF_2 GO:0003676~nucleic acid binding; GOTERM_BP_3 GO:0009889~regulation of biosynthetic process; GOTERM_BP_3 GO:0010467~gene expression; GOTERM_BP_4 GO:0031326~regulation of cellular biosynthetic process; GOTERM_BP_4 GO:0009889~regulation of biosynthetic process; GOTERM_BP_5 GO:0019219~regulation of nucleobase, nucleoside, nucleotide and nucleic acid metabolic process; GOTERM_BP_5 GO:0010556~regulation of macromolecule biosynthetic process; GOTERM_BP_5 GO:0010468~regulation of gene expression; GOTERM_BP_5 GO:0031326~regulation of cellular biosynthetic process; GOTERM_BP_3 GO:0060255~regulation of macromolecule metabolic process; GOTERM_BP_3 GO:0080090~regulation of primary metabolic process; GOTERM_BP_4 GO:0060255~regulation of macromolecule metabolic process; GOTERM_BP_4 GO:0080090~regulation of primary metabolic process; GOTERM_BP_3 GO:0006139~nucleobase, nucleoside, nucleotide and nucleic acid metabolic process; GOTERM_BP_5 GO:0016481~negative regulation of transcription; GOTERM_BP_2 GO:0009058~biosynthetic process; GOTERM_BP_3 GO:0044249~cellular biosynthetic process; GOTERM_BP_3 GO:0031323~regulation of cellular metabolic process; GOTERM_BP_4 GO:0010629~negative regulation of gene expression; GOTERM_BP_3 GO:0051172~negative regulation of nitrogen compound metabolic process; GOTERM_BP_2 GO:0019222~regulation of metabolic process; GOTERM_BP_4 GO:0045934~negative regulation of nucleobase, nucleoside, nucleotide and nucleic acid metabolic process; GOTERM_MF_3 GO:0008134~transcription factor binding; GOTERM_BP_4 GO:0051172~negative regulation of nitrogen compound metabolic process; GOTERM_BP_4 GO:0031323~regulation of cellular metabolic process; GOTERM_BP_5 GO:0010629~negative regulation of gene expression; GOTERM_BP_3 GO:0019222~regulation of metabolic process; GOTERM_BP_4 GO:0010558~negative regulation of macromolecule biosynthetic process; GOTERM_BP_5 GO:0045934~negative regulation of nucleobase, nucleoside, nucleotide and nucleic acid metabolic process; GOTERM_BP_2 GO:0006807~nitrogen compound metabolic process; GOTERM_BP_5 GO:0051172~negative regulation of nitrogen compound metabolic process; GOTERM_BP_3 GO:0034641~cellular nitrogen compound metabolic process; GOTERM_BP_3 GO:0009890~negative regulation of biosynthetic process; GOTERM_BP_4 GO:0031327~negative regulation of cellular biosynthetic process; GOTERM_BP_4 GO:0009890~negative regulation of biosynthetic process; GOTERM_BP_5 GO:0010558~negative regulation of macromolecule biosynthetic process; GOTERM_BP_5 GO:0031327~negative regulation of cellular biosynthetic process; GOTERM_BP_5 GO:0009890~negative regulation of biosynthetic process; GOTERM_BP_3 GO:0031324~negative regulation of cellular metabolic process; GOTERM_BP_3 GO:0010605~negative regulation of macromolecule metabolic process; GOTERM_BP_4 GO:0031324~negative regulation of cellular metabolic process; GOTERM_BP_4 GO:0010605~negative regulation of macromolecule metabolic process; GOTERM_BP_2 GO:0009892~negative regulation of metabolic process; GOTERM_BP_3 GO:0009892~negative regulation of metabolic process; GOTERM_BP_5 GO:0031324~negative regulation of cellular metabolic process; GOTERM_BP_4 GO:0009892~negative regulation of metabolic process; GOTERM_BP_5 GO:0010605~negative regulation of macromolecule metabolic process; GOTERM_CC_4 GO:0005634~nucleus; GOTERM_CC_5 GO:0005634~nucleus; GOTERM_BP_2 GO:0048519~negative regulation of biological process; GOTERM_BP_3 GO:0044260~cellular macromolecule metabolic process; GOTERM_BP_3 GO:0048519~negative regulation of biological process; GOTERM_BP_2 GO:0043170~macromolecule metabolic process; GOTERM_BP_2 GO:0048523~negative regulation of cellular process; GOTERM_BP_3 GO:0048523~negative regulation of cellular process; GOTERM_BP_4 GO:0048523~negative regulation of cellular process; GOTERM_BP_2 GO:0044237~cellular metabolic process; GOTERM_BP_4 GO:0051253~negative regulation of RNA metabolic process; GOTERM_BP_2 GO:0050794~regulation of cellular process; GOTERM_BP_5 GO:0045892~negative regulation of transcription, DNA-dependent; GOTERM_BP_5 GO:0051253~negative regulation of RNA metabolic process; GOTERM_BP_2 GO:0044238~primary metabolic process; GOTERM_BP_3 GO:0050794~regulation of cellular process; GOTERM_BP_3 GO:0048513~organ development; GOTERM_BP_2 GO:0050789~regulation of biological process; GOTERM_BP_4 GO:0048513~organ development; GOTERM_CC_3 GO:0043231~intracellular membrane-bounded organelle; GOTERM_CC_2 GO:0043227~membrane-bounded organelle; GOTERM_BP_1 GO:0065007~biological regulation; GOTERM_CC_4 GO:0043231~intracellular membrane-bounded organelle; GOTERM_BP_1 GO:0008152~metabolic process; GOTERM_CC_5 GO:0043231~intracellular membrane-bounded organelle; GOTERM_CC_1 GO:0043226~organelle; GOTERM_CC_2 GO:0043229~intracellular organelle; GOTERM_CC_3 GO:0043229~intracellular organelle; GOTERM_BP_3 GO:0048731~system development; GOTERM_BP_2 GO:0048856~anatomical structure development; GOTERM_CC_4 GO:0043229~intracellular organelle; GOTERM_CC_5 GO:0043229~intracellular organelle; GOTERM_MF_2 GO:0016564~transcription repressor activity; GOTERM_MF_2 GO:0005515~protein binding; GOTERM_BP_2 GO:0007275~multicellular organismal development; GOTERM_BP_1 GO:0032502~developmental process; GOTERM_CC_2 GO:0044424~intracellular part; GOTERM_CC_3 GO:0044424~intracellular part; GOTERM_CC_2 GO:0005622~intracellular; GOTERM_CC_3 GO:0005622~intracellular; GOTERM_CC_4 GO:0044424~intracellular part; GOTERM_BP_1 GO:0032501~multicellular organismal process; GOTERM_MF_4 GO:0043566~structure-specific DNA binding; GOTERM_BP_4 GO:0007507~heart development; GOTERM_BP_5 GO:0007507~heart development |
| 3866-5975 | GOTERM_MF_4 GO:0043565~sequence-specific DNA binding; GOTERM_MF_4 GO:0003700~transcription factor activity; GOTERM_BP_3 GO:0048513~organ development; GOTERM_BP_4 GO:0048513~organ development; GOTERM_BP_3 GO:0048731~system development; GOTERM_BP_2 GO:0048856~anatomical structure development; GOTERM_BP_2 GO:0007275~multicellular organismal development; GOTERM_BP_1 GO:0032502~developmental process; GOTERM_CC_2 GO:0044424~intracellular part; GOTERM_CC_3 GO:0044424~intracellular part; GOTERM_CC_2 GO:0005622~intracellular; GOTERM_CC_3 GO:0005622~intracellular; GOTERM_CC_4 GO:0044424~intracellular part; GOTERM_BP_2 GO:0009653~anatomical structure morphogenesis; GOTERM_BP_3 GO:0009653~anatomical structure morphogenesis; GOTERM_BP_1 GO:0032501~multicellular organismal process; GOTERM_MF_1 GO:0005488~binding |
| 3866-4958 | GOTERM_BP_4 GO:0051254~positive regulation of RNA metabolic process; GOTERM_BP_5 GO:0045893~positive regulation of transcription, DNA-dependent; GOTERM_BP_5 GO:0051254~positive regulation of RNA metabolic process; GOTERM_BP_3 GO:0009889~regulation of biosynthetic process; GOTERM_BP_2 GO:0048522~positive regulation of cellular process; GOTERM_BP_4 GO:0031326~regulation of cellular biosynthetic process; GOTERM_BP_4 GO:0009889~regulation of biosynthetic process; GOTERM_BP_3 GO:0048522~positive regulation of cellular process; GOTERM_BP_4 GO:0048522~positive regulation of cellular process; GOTERM_BP_3 GO:0010604~positive regulation of macromolecule metabolic process; GOTERM_BP_4 GO:0045935~positive regulation of nucleobase, nucleoside, nucleotide and nucleic acid metabolic process; GOTERM_BP_4 GO:0010604~positive regulation of macromolecule metabolic process; GOTERM_BP_3 GO:0051173~positive regulation of nitrogen compound metabolic process; GOTERM_BP_5 GO:0031326~regulation of cellular biosynthetic process; GOTERM_BP_2 GO:0048518~positive regulation of biological process; GOTERM_BP_2 GO:0009893~positive regulation of metabolic process; GOTERM_BP_3 GO:0060255~regulation of macromolecule metabolic process; GOTERM_BP_4 GO:0051173~positive regulation of nitrogen compound metabolic process; GOTERM_BP_3 GO:0080090~regulation of primary metabolic process; GOTERM_BP_4 GO:0010557~positive regulation of macromolecule biosynthetic process; GOTERM_BP_3 GO:0009893~positive regulation of metabolic process; GOTERM_BP_3 GO:0048518~positive regulation of biological process; GOTERM_BP_4 GO:0060255~regulation of macromolecule metabolic process; GOTERM_BP_5 GO:0045935~positive regulation of nucleobase, nucleoside, nucleotide and nucleic acid metabolic process; GOTERM_BP_5 GO:0010604~positive regulation of macromolecule metabolic process; GOTERM_BP_4 GO:0080090~regulation of primary metabolic process; GOTERM_BP_4 GO:0009893~positive regulation of metabolic process; GOTERM_BP_3 GO:0009891~positive regulation of biosynthetic process; GOTERM_BP_4 GO:0031328~positive regulation of cellular biosynthetic process; GOTERM_BP_5 GO:0051173~positive regulation of nitrogen compound metabolic process; GOTERM_BP_4 GO:0009891~positive regulation of biosynthetic process; GOTERM_BP_5 GO:0010557~positive regulation of macromolecule biosynthetic process; GOTERM_BP_3 GO:0031323~regulation of cellular metabolic process; GOTERM_BP_2 GO:0019222~regulation of metabolic process; GOTERM_BP_4 GO:0031323~regulation of cellular metabolic process; GOTERM_BP_5 GO:0031328~positive regulation of cellular biosynthetic process; GOTERM_BP_5 GO:0009891~positive regulation of biosynthetic process; GOTERM_BP_3 GO:0019222~regulation of metabolic process; GOTERM_BP_3 GO:0031325~positive regulation of cellular metabolic process; GOTERM_BP_4 GO:0031325~positive regulation of cellular metabolic process; GOTERM_BP_5 GO:0031325~positive regulation of cellular metabolic process; GOTERM_BP_2 GO:0048519~negative regulation of biological process; GOTERM_BP_3 GO:0048519~negative regulation of biological process; GOTERM_BP_2 GO:0048523~negative regulation of cellular process; GOTERM_BP_3 GO:0048523~negative regulation of cellular process; GOTERM_BP_4 GO:0048523~negative regulation of cellular process; GOTERM_BP_3 GO:0007165~signal transduction; GOTERM_BP_4 GO:0007165~signal transduction; GOTERM_BP_2 GO:0050794~regulation of cellular process; GOTERM_BP_3 GO:0050794~regulation of cellular process; GOTERM_BP_3 GO:0048513~organ development; GOTERM_BP_2 GO:0050789~regulation of biological process; GOTERM_BP_4 GO:0048513~organ development; GOTERM_BP_1 GO:0065007~biological regulation; GOTERM_BP_4 GO:0007242~intracellular signaling cascade; GOTERM_BP_3 GO:0048731~system development; GOTERM_BP_2 GO:0048856~anatomical structure development; GOTERM_MF_2 GO:0005515~protein binding; GOTERM_BP_2 GO:0007275~multicellular organismal development; GOTERM_BP_1 GO:0032502~developmental process; GOTERM_BP_3 GO:0010941~regulation of cell death; GOTERM_BP_3 GO:0045595~regulation of cell differentiation; GOTERM_BP_4 GO:0043067~regulation of programmed cell death; GOTERM_BP_4 GO:0010941~regulation of cell death; GOTERM_BP_4 GO:0045595~regulation of cell differentiation; GOTERM_BP_5 GO:0042981~regulation of apoptosis; GOTERM_BP_5 GO:0043067~regulation of programmed cell death; GOTERM_MF_3 GO:0005102~receptor binding; GOTERM_BP_2 GO:0050793~regulation of developmental process; GOTERM_BP_3 GO:0050793~regulation of developmental process; GOTERM_BP_1 GO:0032501~multicellular organismal process; GOTERM_BP_3 GO:0042127~regulation of cell proliferation; GOTERM_BP_4 GO:0042127~regulation of cell proliferation; GOTERM_BP_4 GO:0007517~muscle organ development; GOTERM_BP_3 GO:0045597~positive regulation of cell differentiation; GOTERM_BP_5 GO:0007517~muscle organ development; GOTERM_BP_4 GO:0045597~positive regulation of cell differentiation; GOTERM_BP_5 GO:0045597~positive regulation of cell differentiation; GOTERM_BP_2 GO:0051094~positive regulation of developmental process; GOTERM_BP_3 GO:0051094~positive regulation of developmental process; GOTERM_BP_4 GO:0051094~positive regulation of developmental process |
| 3866-6404 | GOTERM_MF_2 GO:0003700~transcription factor activity; GOTERM_MF_4 GO:0003700~transcription factor activity; GOTERM_MF_1 GO:0030528~transcription regulator activity; GOTERM_MF_2 GO:0016563~transcription activator activity; GOTERM_BP_4 GO:0051252~regulation of RNA metabolic process; GOTERM_BP_5 GO:0006355~regulation of transcription, DNA-dependent; GOTERM_BP_5 GO:0051252~regulation of RNA metabolic process; GOTERM_BP_4 GO:0006350~transcription; GOTERM_MF_3 GO:0003677~DNA binding; GOTERM_BP_4 GO:0010628~positive regulation of gene expression; GOTERM_BP_5 GO:0010628~positive regulation of gene expression; GOTERM_BP_4 GO:0051254~positive regulation of RNA metabolic process; GOTERM_BP_5 GO:0045893~positive regulation of transcription, DNA-dependent; GOTERM_BP_5 GO:0051254~positive regulation of RNA metabolic process; GOTERM_BP_3 GO:0009059~macromolecule biosynthetic process; GOTERM_BP_3 GO:0051171~regulation of nitrogen compound metabolic process; GOTERM_BP_5 GO:0045449~regulation of transcription; GOTERM_BP_4 GO:0034645~cellular macromolecule biosynthetic process; GOTERM_BP_4 GO:0019219~regulation of nucleobase, nucleoside, nucleotide and nucleic acid metabolic process; GOTERM_BP_4 GO:0010556~regulation of macromolecule biosynthetic process; GOTERM_BP_4 GO:0051171~regulation of nitrogen compound metabolic process; GOTERM_BP_4 GO:0010468~regulation of gene expression; GOTERM_BP_3 GO:0009889~regulation of biosynthetic process; GOTERM_BP_2 GO:0048522~positive regulation of cellular process; GOTERM_BP_3 GO:0010467~gene expression; GOTERM_BP_4 GO:0031326~regulation of cellular biosynthetic process; GOTERM_BP_4 GO:0009889~regulation of biosynthetic process; GOTERM_BP_3 GO:0048522~positive regulation of cellular process; GOTERM_BP_5 GO:0019219~regulation of nucleobase, nucleoside, nucleotide and nucleic acid metabolic process; GOTERM_BP_5 GO:0010556~regulation of macromolecule biosynthetic process; GOTERM_BP_4 GO:0048522~positive regulation of cellular process; GOTERM_BP_5 GO:0010468~regulation of gene expression; GOTERM_BP_5 GO:0045941~positive regulation of transcription; GOTERM_BP_3 GO:0010604~positive regulation of macromolecule metabolic process; GOTERM_BP_4 GO:0045935~positive regulation of nucleobase, nucleoside, nucleotide and nucleic acid metabolic process; GOTERM_BP_4 GO:0010604~positive regulation of macromolecule metabolic process; GOTERM_BP_3 GO:0051173~positive regulation of nitrogen compound metabolic process; GOTERM_BP_5 GO:0031326~regulation of cellular biosynthetic process; GOTERM_BP_2 GO:0048518~positive regulation of biological process; GOTERM_BP_2 GO:0009893~positive regulation of metabolic process; GOTERM_BP_3 GO:0060255~regulation of macromolecule metabolic process; GOTERM_BP_4 GO:0051173~positive regulation of nitrogen compound metabolic process; GOTERM_BP_3 GO:0080090~regulation of primary metabolic process; GOTERM_BP_4 GO:0010557~positive regulation of macromolecule biosynthetic process; GOTERM_BP_3 GO:0048518~positive regulation of biological process; GOTERM_BP_4 GO:0060255~regulation of macromolecule metabolic process; GOTERM_BP_5 GO:0045935~positive regulation of nucleobase, nucleoside, nucleotide and nucleic acid metabolic process; GOTERM_BP_4 GO:0080090~regulation of primary metabolic process; GOTERM_BP_3 GO:0009891~positive regulation of biosynthetic process; GOTERM_BP_4 GO:0031328~positive regulation of cellular biosynthetic process; GOTERM_BP_5 GO:0051173~positive regulation of nitrogen compound metabolic process; GOTERM_BP_3 GO:0006139~nucleobase, nucleoside, nucleotide and nucleic acid metabolic process; GOTERM_BP_5 GO:0016481~negative regulation of transcription; GOTERM_BP_2 GO:0009058~biosynthetic process; GOTERM_BP_4 GO:0009891~positive regulation of biosynthetic process; GOTERM_BP_5 GO:0010557~positive regulation of macromolecule biosynthetic process; GOTERM_BP_3 GO:0044249~cellular biosynthetic process; GOTERM_BP_3 GO:0031323~regulation of cellular metabolic process; GOTERM_BP_4 GO:0010629~negative regulation of gene expression; GOTERM_BP_3 GO:0051172~negative regulation of nitrogen compound metabolic process; GOTERM_BP_2 GO:0019222~regulation of metabolic process; GOTERM_BP_4 GO:0045934~negative regulation of nucleobase, nucleoside, nucleotide and nucleic acid metabolic process; GOTERM_MF_3 GO:0008134~transcription factor binding; GOTERM_BP_4 GO:0051172~negative regulation of nitrogen compound metabolic process; GOTERM_BP_4 GO:0031323~regulation of cellular metabolic process; GOTERM_BP_5 GO:0031328~positive regulation of cellular biosynthetic process; GOTERM_BP_5 GO:0009891~positive regulation of biosynthetic process; GOTERM_BP_5 GO:0010629~negative regulation of gene expression; GOTERM_BP_3 GO:0019222~regulation of metabolic process; GOTERM_BP_4 GO:0010558~negative regulation of macromolecule biosynthetic process; GOTERM_BP_5 GO:0045934~negative regulation of nucleobase, nucleoside, nucleotide and nucleic acid metabolic process; GOTERM_BP_2 GO:0006807~nitrogen compound metabolic process; GOTERM_BP_5 GO:0051172~negative regulation of nitrogen compound metabolic process; GOTERM_BP_3 GO:0034641~cellular nitrogen compound metabolic process; GOTERM_BP_3 GO:0009890~negative regulation of biosynthetic process; GOTERM_BP_4 GO:0031327~negative regulation of cellular biosynthetic process; GOTERM_BP_4 GO:0009890~negative regulation of biosynthetic process; GOTERM_BP_5 GO:0010558~negative regulation of macromolecule biosynthetic process; GOTERM_BP_5 GO:0031327~negative regulation of cellular biosynthetic process; GOTERM_BP_5 GO:0009890~negative regulation of biosynthetic process; GOTERM_BP_3 GO:0031325~positive regulation of cellular metabolic process; GOTERM_BP_4 GO:0031325~positive regulation of cellular metabolic process; GOTERM_BP_3 GO:0031324~negative regulation of cellular metabolic process; GOTERM_BP_3 GO:0010605~negative regulation of macromolecule metabolic process; GOTERM_BP_4 GO:0031324~negative regulation of cellular metabolic process; GOTERM_BP_4 GO:0010605~negative regulation of macromolecule metabolic process; GOTERM_BP_2 GO:0009892~negative regulation of metabolic process; GOTERM_BP_3 GO:0009892~negative regulation of metabolic process; GOTERM_BP_5 GO:0031324~negative regulation of cellular metabolic process; GOTERM_BP_4 GO:0009892~negative regulation of metabolic process; GOTERM_BP_5 GO:0010605~negative regulation of macromolecule metabolic process; GOTERM_CC_4 GO:0005634~nucleus; GOTERM_CC_5 GO:0005634~nucleus; GOTERM_BP_2 GO:0048519~negative regulation of biological process; GOTERM_BP_3 GO:0044260~cellular macromolecule metabolic process; GOTERM_BP_2 GO:0043170~macromolecule metabolic process; GOTERM_BP_2 GO:0048523~negative regulation of cellular process; GOTERM_BP_3 GO:0048523~negative regulation of cellular process; GOTERM_BP_4 GO:0048523~negative regulation of cellular process; GOTERM_MF_2 GO:0003712~transcription cofactor activity; GOTERM_BP_2 GO:0044237~cellular metabolic process; GOTERM_BP_4 GO:0051253~negative regulation of RNA metabolic process; GOTERM_BP_2 GO:0050794~regulation of cellular process; GOTERM_BP_5 GO:0045892~negative regulation of transcription, DNA-dependent; GOTERM_BP_5 GO:0051253~negative regulation of RNA metabolic process; GOTERM_MF_3 GO:0003713~transcription coactivator activity; GOTERM_MF_4 GO:0051427~hormone receptor binding; GOTERM_BP_2 GO:0044238~primary metabolic process; GOTERM_MF_5 GO:0035257~nuclear hormone receptor binding; GOTERM_MF_4 GO:0003712~transcription cofactor activity; GOTERM_BP_3 GO:0050794~regulation of cellular process; GOTERM_BP_2 GO:0050789~regulation of biological process; GOTERM_CC_3 GO:0043231~intracellular membrane-bounded organelle; GOTERM_CC_2 GO:0043227~membrane-bounded organelle; GOTERM_MF_5 GO:0003713~transcription coactivator activity; GOTERM_BP_1 GO:0065007~biological regulation; GOTERM_CC_4 GO:0043231~intracellular membrane-bounded organelle; GOTERM_BP_1 GO:0008152~metabolic process; GOTERM_CC_5 GO:0043231~intracellular membrane-bounded organelle; GOTERM_CC_1 GO:0043226~organelle; GOTERM_CC_2 GO:0043229~intracellular organelle; GOTERM_CC_3 GO:0043229~intracellular organelle; GOTERM_CC_4 GO:0043229~intracellular organelle; GOTERM_CC_5 GO:0043229~intracellular organelle; GOTERM_MF_2 GO:0016564~transcription repressor activity; GOTERM_MF_2 GO:0005515~protein binding; GOTERM_CC_2 GO:0044424~intracellular part; GOTERM_CC_3 GO:0044424~intracellular part; GOTERM_CC_2 GO:0005622~intracellular; GOTERM_CC_3 GO:0005622~intracellular; GOTERM_CC_4 GO:0044424~intracellular part; GOTERM_BP_1 GO:0009987~cellular process; GOTERM_MF_2 GO:0003702~RNA polymerase II transcription factor activity |
| 3866-6433 | GOTERM_BP_3 GO:0051172~negative regulation of nitrogen compound metabolic process; GOTERM_BP_4 GO:0045934~negative regulation of nucleobase, nucleoside, nucleotide and nucleic acid metabolic process; GOTERM_BP_4 GO:0051172~negative regulation of nitrogen compound metabolic process; GOTERM_BP_4 GO:0010558~negative regulation of macromolecule biosynthetic process; GOTERM_BP_5 GO:0045934~negative regulation of nucleobase, nucleoside, nucleotide and nucleic acid metabolic process; GOTERM_BP_5 GO:0051172~negative regulation of nitrogen compound metabolic process; GOTERM_BP_3 GO:0031324~negative regulation of cellular metabolic process; GOTERM_BP_4 GO:0031324~negative regulation of cellular metabolic process; GOTERM_BP_5 GO:0031324~negative regulation of cellular metabolic process; GOTERM_BP_4 GO:0009892~negative regulation of metabolic process; GOTERM_CC_5 GO:0005634~nucleus; GOTERM_BP_2 GO:0048519~negative regulation of biological process; GOTERM_BP_3 GO:0048519~negative regulation of biological process; GOTERM_BP_2 GO:0048523~negative regulation of cellular process; GOTERM_BP_3 GO:0048523~negative regulation of cellular process; GOTERM_BP_4 GO:0048523~negative regulation of cellular process; GOTERM_BP_4 GO:0031667~response to nutrient levels; GOTERM_BP_3 GO:0009991~response to extracellular stimulus; GOTERM_CC_3 GO:0043231~intracellular membrane-bounded organelle; GOTERM_CC_2 GO:0043227~membrane-bounded organelle; GOTERM_BP_3 GO:0007584~response to nutrient; GOTERM_CC_4 GO:0043231~intracellular membrane-bounded organelle; GOTERM_BP_5 GO:0007584~response to nutrient; GOTERM_CC_5 GO:0043231~intracellular membrane-bounded organelle; GOTERM_CC_1 GO:0043226~organelle; GOTERM_CC_2 GO:0043229~intracellular organelle; GOTERM_CC_3 GO:0043229~intracellular organelle; GOTERM_CC_4 GO:0043229~intracellular organelle; GOTERM_CC_5 GO:0043229~intracellular organelle; GOTERM_MF_2 GO:0005515~protein binding; GOTERM_BP_1 GO:0050896~response to stimulus; GOTERM_BP_3 GO:0008285~negative regulation of cell proliferation; GOTERM_BP_4 GO:0008285~negative regulation of cell proliferation; GOTERM_BP_5 GO:0008285~negative regulation of cell proliferation; GOTERM_BP_2 GO:0009605~response to external stimulus; GOTERM_BP_3 GO:0042127~regulation of cell proliferation; GOTERM_BP_4 GO:0042127~regulation of cell proliferation; GOTERM_MF_1 GO:0005488~binding |
| 3866-6116 | GOTERM_MF_4 GO:0043565~sequence-specific DNA binding; GOTERM_MF_2 GO:0003700~transcription factor activity; GOTERM_MF_3 GO:0004872~receptor activity; GOTERM_MF_1 GO:0060089~molecular transducer activity; GOTERM_MF_2 GO:0004871~signal transducer activity; GOTERM_BP_4 GO:0010628~positive regulation of gene expression; GOTERM_BP_5 GO:0010628~positive regulation of gene expression; GOTERM_BP_4 GO:0051254~positive regulation of RNA metabolic process; GOTERM_BP_5 GO:0045893~positive regulation of transcription, DNA-dependent; GOTERM_BP_5 GO:0051254~positive regulation of RNA metabolic process; GOTERM_BP_5 GO:0045449~regulation of transcription; GOTERM_BP_3 GO:0010467~gene expression; GOTERM_BP_5 GO:0045941~positive regulation of transcription; GOTERM_BP_4 GO:0045935~positive regulation of nucleobase, nucleoside, nucleotide and nucleic acid metabolic process; GOTERM_BP_3 GO:0051173~positive regulation of nitrogen compound metabolic process; GOTERM_BP_3 GO:0060255~regulation of macromolecule metabolic process; GOTERM_BP_4 GO:0051173~positive regulation of nitrogen compound metabolic process; GOTERM_BP_3 GO:0080090~regulation of primary metabolic process; GOTERM_BP_4 GO:0010557~positive regulation of macromolecule biosynthetic process; GOTERM_BP_4 GO:0060255~regulation of macromolecule metabolic process; GOTERM_BP_5 GO:0045935~positive regulation of nucleobase, nucleoside, nucleotide and nucleic acid metabolic process; GOTERM_BP_4 GO:0080090~regulation of primary metabolic process; GOTERM_BP_3 GO:0009891~positive regulation of biosynthetic process; GOTERM_BP_4 GO:0031328~positive regulation of cellular biosynthetic process; GOTERM_BP_5 GO:0051173~positive regulation of nitrogen compound metabolic process; GOTERM_BP_4 GO:0009891~positive regulation of biosynthetic process; GOTERM_BP_5 GO:0010557~positive regulation of macromolecule biosynthetic process; GOTERM_BP_5 GO:0031328~positive regulation of cellular biosynthetic process; GOTERM_BP_5 GO:0009891~positive regulation of biosynthetic process; GOTERM_BP_3 GO:0044260~cellular macromolecule metabolic process; GOTERM_MF_2 GO:0005515~protein binding; GOTERM_BP_1 GO:0050896~response to stimulus; GOTERM_MF_1 GO:0005488~binding |
| 3866-6410 | GOTERM_CC_1 GO:0043226~organelle; GOTERM_CC_2 GO:0043229~intracellular organelle; GOTERM_CC_3 GO:0043229~intracellular organelle |
| 3903-5414 | GOTERM_BP_2 GO:0048522~positive regulation of cellular process; GOTERM_BP_3 GO:0048522~positive regulation of cellular process; GOTERM_BP_4 GO:0048522~positive regulation of cellular process; GOTERM_BP_2 GO:0051716~cellular response to stimulus; GOTERM_BP_2 GO:0048518~positive regulation of biological process; GOTERM_BP_3 GO:0048518~positive regulation of biological process; GOTERM_MF_2 GO:0005515~protein binding; GOTERM_BP_1 GO:0016043~cellular component organization; GOTERM_BP_1 GO:0050896~response to stimulus |
| 3903-4944 | GOTERM_MF_3 GO:0005102~receptor binding; GOTERM_BP_2 GO:0009893~positive regulation of metabolic process; GOTERM_BP_3 GO:0009893~positive regulation of metabolic process; GOTERM_BP_4 GO:0009893~positive regulation of metabolic process; GOTERM_BP_4 GO:0030335~positive regulation of cell migration; GOTERM_BP_2 GO:0040017~positive regulation of locomotion; GOTERM_BP_5 GO:0030335~positive regulation of cell migration; GOTERM_BP_3 GO:0051272~positive regulation of cell motion; GOTERM_BP_3 GO:0040017~positive regulation of locomotion; GOTERM_BP_4 GO:0051272~positive regulation of cell motion; GOTERM_BP_4 GO:0040017~positive regulation of locomotion; GOTERM_BP_5 GO:0051272~positive regulation of cell motion; GOTERM_MF_3 GO:0019838~growth factor binding; GOTERM_BP_2 GO:0048522~positive regulation of cellular process; GOTERM_BP_3 GO:0010033~response to organic substance; GOTERM_BP_3 GO:0048522~positive regulation of cellular process; GOTERM_BP_4 GO:0048522~positive regulation of cellular process; GOTERM_BP_2 GO:0048518~positive regulation of biological process; GOTERM_BP_3 GO:0030334~regulation of cell migration; GOTERM_BP_3 GO:0048518~positive regulation of biological process; GOTERM_BP_2 GO:0048519~negative regulation of biological process; GOTERM_MF_4 GO:0004896~cytokine receptor activity; GOTERM_BP_3 GO:0048519~negative regulation of biological process; GOTERM_MF_5 GO:0004896~cytokine receptor activity; GOTERM_BP_2 GO:0042221~response to chemical stimulus; GOTERM_CC_3 GO:0043235~receptor complex; GOTERM_BP_3 GO:0010604~positive regulation of macromolecule metabolic process; GOTERM_BP_4 GO:0010604~positive regulation of macromolecule metabolic process; GOTERM_BP_3 GO:0031325~positive regulation of cellular metabolic process; GOTERM_MF_3 GO:0019955~cytokine binding; GOTERM_BP_4 GO:0031325~positive regulation of cellular metabolic process; GOTERM_BP_5 GO:0010604~positive regulation of macromolecule metabolic process; GOTERM_BP_5 GO:0031325~positive regulation of cellular metabolic process; GOTERM_BP_3 GO:0080090~regulation of primary metabolic process; GOTERM_BP_1 GO:0050896~response to stimulus |
| 3903-5982 | GOTERM_BP_4 GO:0051338~regulation of transferase activity; GOTERM_BP_5 GO:0043549~regulation of kinase activity; GOTERM_BP_4 GO:0051174~regulation of phosphorus metabolic process; GOTERM_BP_5 GO:0051174~regulation of phosphorus metabolic process; GOTERM_BP_5 GO:0019220~regulation of phosphate metabolic process; GOTERM_BP_2 GO:0048522~positive regulation of cellular process; GOTERM_BP_3 GO:0048522~positive regulation of cellular process; GOTERM_BP_4 GO:0048522~positive regulation of cellular process; GOTERM_BP_2 GO:0048518~positive regulation of biological process; GOTERM_BP_3 GO:0048518~positive regulation of biological process; GOTERM_BP_2 GO:0050794~regulation of cellular process; GOTERM_MF_2 GO:0005515~protein binding; GOTERM_BP_3 GO:0050794~regulation of cellular process; GOTERM_BP_2 GO:0050789~regulation of biological process; GOTERM_BP_1 GO:0065007~biological regulation; GOTERM_BP_3 GO:0031323~regulation of cellular metabolic process; GOTERM_BP_2 GO:0019222~regulation of metabolic process; GOTERM_BP_4 GO:0031323~regulation of cellular metabolic process; GOTERM_BP_3 GO:0019222~regulation of metabolic process; GOTERM_BP_3 GO:0042127~regulation of cell proliferation; GOTERM_BP_4 GO:0042127~regulation of cell proliferation; GOTERM_BP_3 GO:0080090~regulation of primary metabolic process; GOTERM_BP_4 GO:0080090~regulation of primary metabolic process; GOTERM_BP_3 GO:0048513~organ development; GOTERM_BP_4 GO:0048513~organ development |
| 3903-4224 | GOTERM_BP_3 GO:0007165~signal transduction; GOTERM_BP_4 GO:0007165~signal transduction; GOTERM_BP_4 GO:0007166~cell surface receptor linked signal transduction; GOTERM_BP_5 GO:0007166~cell surface receptor linked signal transduction; GOTERM_MF_1 GO:0060089~molecular transducer activity; GOTERM_MF_2 GO:0004871~signal transducer activity; GOTERM_BP_2 GO:0040008~regulation of growth; GOTERM_MF_3 GO:0004872~receptor activity; GOTERM_MF_4 GO:0004888~transmembrane receptor activity; GOTERM_MF_2 GO:0042277~peptide binding; GOTERM_BP_3 GO:0042127~regulation of cell proliferation; GOTERM_BP_4 GO:0042127~regulation of cell proliferation; GOTERM_CC_5 GO:0005887~integral to plasma membrane; GOTERM_CC_5 GO:0031226~intrinsic to plasma membrane |
| 3903-2305 | GOTERM_MF_3 GO:0019955~cytokine binding; GOTERM_MF_2 GO:0042277~peptide binding |
| 3903-5822 | GOTERM_BP_4 GO:0007166~cell surface receptor linked signal transduction; GOTERM_BP_5 GO:0007166~cell surface receptor linked signal transduction; GOTERM_MF_1 GO:0060089~molecular transducer activity; GOTERM_MF_2 GO:0004871~signal transducer activity; GOTERM_BP_3 GO:0031323~regulation of cellular metabolic process; GOTERM_CC_4 GO:0005886~plasma membrane; GOTERM_BP_2 GO:0019222~regulation of metabolic process; GOTERM_BP_4 GO:0031323~regulation of cellular metabolic process; GOTERM_BP_3 GO:0019222~regulation of metabolic process; GOTERM_CC_3 GO:0005886~plasma membrane; GOTERM_CC_5 GO:0031226~intrinsic to plasma membrane |
| 3903-5931 | GOTERM_BP_4 GO:0007166~cell surface receptor linked signal transduction; GOTERM_BP_4 GO:0051174~regulation of phosphorus metabolic process; GOTERM_BP_3 GO:0050790~regulation of catalytic activity; GOTERM_BP_5 GO:0051174~regulation of phosphorus metabolic process; GOTERM_BP_5 GO:0019220~regulation of phosphate metabolic process; GOTERM_BP_4 GO:0043085~positive regulation of catalytic activity; GOTERM_BP_5 GO:0007166~cell surface receptor linked signal transduction; GOTERM_BP_2 GO:0065009~regulation of molecular function; GOTERM_BP_3 GO:0044093~positive regulation of molecular function; GOTERM_BP_2 GO:0048518~positive regulation of biological process; GOTERM_BP_3 GO:0048518~positive regulation of biological process; GOTERM_BP_2 GO:0048519~negative regulation of biological process; GOTERM_CC_4 GO:0005886~plasma membrane; GOTERM_BP_3 GO:0048519~negative regulation of biological process; GOTERM_CC_3 GO:0005886~plasma membrane; GOTERM_BP_2 GO:0048523~negative regulation of cellular process; GOTERM_BP_3 GO:0048523~negative regulation of cellular process; GOTERM_BP_4 GO:0048523~negative regulation of cellular process; GOTERM_BP_2 GO:0032879~regulation of localization; GOTERM_BP_3 GO:0032879~regulation of localization; GOTERM_CC_2 GO:0016020~membrane; GOTERM_CC_3 GO:0016020~membrane; GOTERM_CC_5 GO:0005901~caveola; GOTERM_CC_5 GO:0005887~integral to plasma membrane; GOTERM_CC_5 GO:0031226~intrinsic to plasma membrane; GOTERM_CC_4 GO:0005901~caveola; GOTERM_BP_3 GO:0051049~regulation of transport; GOTERM_BP_4 GO:0051049~regulation of transport |
| 4287-4945 | GOTERM_CC_1 GO:0044421~extracellular region part; GOTERM_CC_2 GO:0044421~extracellular region part; GOTERM_CC_1 GO:0005576~extracellular region; GOTERM_CC_2 GO:0005615~extracellular space; GOTERM_CC_3 GO:0005615~extracellular space; GOTERM_BP_3 GO:0009653~anatomical structure morphogenesis; GOTERM_MF_5 GO:0005509~calcium ion binding; GOTERM_BP_2 GO:0009653~anatomical structure morphogenesis; GOTERM_BP_2 GO:0048856~anatomical structure development; GOTERM_BP_1 GO:0032501~multicellular organismal process; GOTERM_BP_1 GO:0032502~developmental process; GOTERM_BP_1 GO:0022610~biological adhesion; GOTERM_BP_2 GO:0007155~cell adhesion; GOTERM_BP_2 GO:0007275~multicellular organismal development; GOTERM_BP_3 GO:0048731~system development; GOTERM_BP_2 GO:0065008~regulation of biological quality; GOTERM_BP_3 GO:0006928~cell motion; GOTERM_BP_2 GO:0006928~cell motion; GOTERM_BP_4 GO:0019538~protein metabolic process; GOTERM_BP_3 GO:0019538~protein metabolic process; GOTERM_BP_1 GO:0016043~cellular component organization; GOTERM_MF_2 GO:0005515~protein binding; GOTERM_CC_5 GO:0016023~cytoplasmic membrane-bounded vesicle |
| 4287-5219 | GOTERM_CC_2 GO:0031012~extracellular matrix; GOTERM_CC_3 GO:0031012~extracellular matrix; GOTERM_CC_1 GO:0044421~extracellular region part; GOTERM_CC_4 GO:0005578~proteinaceous extracellular matrix; GOTERM_CC_2 GO:0044421~extracellular region part; GOTERM_CC_3 GO:0005578~proteinaceous extracellular matrix; GOTERM_CC_4 GO:0044420~extracellular matrix part; GOTERM_CC_2 GO:0044420~extracellular matrix part; GOTERM_CC_3 GO:0044420~extracellular matrix part; GOTERM_CC_1 GO:0005576~extracellular region; GOTERM_CC_5 GO:0005604~basement membrane; GOTERM_CC_5 GO:0005581~collagen; GOTERM_CC_4 GO:0005604~basement membrane; GOTERM_CC_3 GO:0005604~basement membrane; GOTERM_MF_1 GO:0005198~structural molecule activity; GOTERM_CC_2 GO:0005615~extracellular space; GOTERM_CC_3 GO:0005615~extracellular space; GOTERM_MF_5 GO:0004175~endopeptidase activity; GOTERM_BP_4 GO:0030155~regulation of cell adhesion; GOTERM_MF_5 GO:0004252~serine-type endopeptidase activity; GOTERM_BP_3 GO:0030155~regulation of cell adhesion; GOTERM_MF_5 GO:0008236~serine-type peptidase activity; GOTERM_BP_3 GO:0051239~regulation of multicellular organismal process; GOTERM_BP_2 GO:0051239~regulation of multicellular organismal process; GOTERM_MF_3 GO:0008233~peptidase activity; GOTERM_MF_4 GO:0070011~peptidase activity, acting on L-amino acid peptides; GOTERM_MF_3 GO:0017171~serine hydrolase activity; GOTERM_MF_4 GO:0008236~serine-type peptidase activity; GOTERM_BP_4 GO:0042060~wound healing; GOTERM_BP_4 GO:0048513~organ development; GOTERM_BP_3 GO:0048513~organ development; GOTERM_BP_2 GO:0048856~anatomical structure development; GOTERM_BP_4 GO:0032101~regulation of response to external stimulus; GOTERM_BP_4 GO:0030195~negative regulation of blood coagulation; GOTERM_BP_1 GO:0032501~multicellular organismal process; GOTERM_BP_3 GO:0032101~regulation of response to external stimulus; GOTERM_BP_5 GO:0030195~negative regulation of blood coagulation; GOTERM_BP_4 GO:0050819~negative regulation of coagulation; GOTERM_BP_3 GO:0050819~negative regulation of coagulation; GOTERM_BP_5 GO:0050819~negative regulation of coagulation; GOTERM_BP_1 GO:0032502~developmental process; GOTERM_BP_1 GO:0022610~biological adhesion; GOTERM_BP_2 GO:0007155~cell adhesion; GOTERM_BP_2 GO:0007275~multicellular organismal development; GOTERM_BP_3 GO:0009611~response to wounding; GOTERM_BP_3 GO:0048731~system development; GOTERM_BP_4 GO:0080134~regulation of response to stress; GOTERM_BP_3 GO:0080134~regulation of response to stress; GOTERM_BP_4 GO:0009888~tissue development; GOTERM_BP_3 GO:0009888~tissue development; GOTERM_BP_3 GO:0050793~regulation of developmental process; GOTERM_BP_2 GO:0050793~regulation of developmental process; GOTERM_BP_3 GO:0048519~negative regulation of biological process; GOTERM_BP_5 GO:0009888~tissue development; GOTERM_BP_4 GO:0051241~negative regulation of multicellular organismal process; GOTERM_BP_2 GO:0048519~negative regulation of biological process; GOTERM_BP_3 GO:0051241~negative regulation of multicellular organismal process; GOTERM_BP_2 GO:0051241~negative regulation of multicellular organismal process; GOTERM_BP_5 GO:0042730~fibrinolysis; GOTERM_BP_4 GO:0008544~epidermis development; GOTERM_BP_5 GO:0008544~epidermis development; GOTERM_BP_4 GO:0007398~ectoderm development; GOTERM_MF_2 GO:0005515~protein binding; GOTERM_BP_5 GO:0007398~ectoderm development; GOTERM_MF_5 GO:0008201~heparin binding; GOTERM_CC_5 GO:0043256~laminin complex; GOTERM_CC_4 GO:0043256~laminin complex; GOTERM_CC_3 GO:0043256~laminin complex; GOTERM_CC_5 GO:0005605~basal lamina; GOTERM_MF_4 GO:0005539~glycosaminoglycan binding; GOTERM_CC_5 GO:0030141~secretory granule; GOTERM_CC_4 GO:0005605~basal lamina; GOTERM_MF_3 GO:0030247~polysaccharide binding; GOTERM_CC_3 GO:0005605~basal lamina; GOTERM_MF_2 GO:0001871~pattern binding; GOTERM_CC_5 GO:0016023~cytoplasmic membrane-bounded vesicle |
| 4287-3853 | GOTERM_CC_2 GO:0031012~extracellular matrix; GOTERM_CC_3 GO:0031012~extracellular matrix; GOTERM_CC_1 GO:0044421~extracellular region part; GOTERM_CC_4 GO:0005578~proteinaceous extracellular matrix; GOTERM_CC_2 GO:0044421~extracellular region part; GOTERM_CC_3 GO:0005578~proteinaceous extracellular matrix; GOTERM_CC_1 GO:0005576~extracellular region; GOTERM_CC_2 GO:0005615~extracellular space; GOTERM_CC_3 GO:0005615~extracellular space; GOTERM_MF_5 GO:0004175~endopeptidase activity; GOTERM_BP_3 GO:0030198~extracellular matrix organization; GOTERM_MF_3 GO:0008233~peptidase activity; GOTERM_BP_2 GO:0043062~extracellular structure organization; GOTERM_MF_4 GO:0070011~peptidase activity, acting on L-amino acid peptides; GOTERM_MF_5 GO:0005509~calcium ion binding; GOTERM_BP_1 GO:0032501~multicellular organismal process; GOTERM_BP_4 GO:0032963~collagen metabolic process; GOTERM_BP_4 GO:0030199~collagen fibril organization; GOTERM_BP_3 GO:0044259~multicellular organismal macromolecule metabolic process; GOTERM_BP_1 GO:0022610~biological adhesion; GOTERM_BP_2 GO:0007155~cell adhesion; GOTERM_BP_2 GO:0044236~multicellular organismal metabolic process; GOTERM_BP_5 GO:0006508~proteolysis; GOTERM_BP_4 GO:0019538~protein metabolic process; GOTERM_BP_3 GO:0019538~protein metabolic process; GOTERM_MF_2 GO:0016787~hydrolase activity; GOTERM_MF_4 GO:0005539~glycosaminoglycan binding; GOTERM_MF_3 GO:0030247~polysaccharide binding; GOTERM_BP_4 GO:0030574~collagen catabolic process; GOTERM_MF_2 GO:0001871~pattern binding; GOTERM_MF_5 GO:0008237~metallopeptidase activity; GOTERM_BP_5 GO:0030574~collagen catabolic process; GOTERM_BP_3 GO:0044243~multicellular organismal catabolic process |
| 4287-4393 | GOTERM_MF_3 GO:0008233~peptidase activity; GOTERM_MF_5 GO:0005509~calcium ion binding; GOTERM_BP_2 GO:0009605~response to external stimulus; GOTERM_BP_4 GO:0016477~cell migration; GOTERM_BP_4 GO:0032101~regulation of response to external stimulus; GOTERM_BP_3 GO:0016477~cell migration; GOTERM_BP_3 GO:0032101~regulation of response to external stimulus; GOTERM_BP_5 GO:0016477~cell migration; GOTERM_BP_4 GO:0048870~cell motility; GOTERM_BP_3 GO:0048870~cell motility; GOTERM_BP_2 GO:0048870~cell motility; GOTERM_BP_2 GO:0051674~localization of cell; GOTERM_BP_1 GO:0022610~biological adhesion; GOTERM_BP_2 GO:0007155~cell adhesion; GOTERM_BP_3 GO:0009611~response to wounding; GOTERM_BP_5 GO:0006508~proteolysis; GOTERM_BP_1 GO:0040011~locomotion; GOTERM_BP_3 GO:0006928~cell motion; GOTERM_BP_2 GO:0006928~cell motion; GOTERM_MF_2 GO:0005515~protein binding; GOTERM_MF_5 GO:0008201~heparin binding; GOTERM_BP_5 GO:0016485~protein processing; GOTERM_BP_4 GO:0051604~protein maturation; GOTERM_BP_5 GO:0051604~protein maturation; GOTERM_MF_4 GO:0005539~glycosaminoglycan binding; GOTERM_CC_5 GO:0030141~secretory granule; GOTERM_MF_3 GO:0030247~polysaccharide binding; GOTERM_MF_2 GO:0001871~pattern binding; GOTERM_CC_5 GO:0016023~cytoplasmic membrane-bounded vesicle |
| 4287-4944 | GOTERM_CC_1 GO:0044421~extracellular region part; GOTERM_CC_2 GO:0044421~extracellular region part; GOTERM_CC_1 GO:0005576~extracellular region; GOTERM_CC_2 GO:0005615~extracellular space; GOTERM_CC_3 GO:0005615~extracellular space; GOTERM_BP_3 GO:0051239~regulation of multicellular organismal process; GOTERM_BP_2 GO:0051239~regulation of multicellular organismal process; GOTERM_BP_4 GO:0030193~regulation of blood coagulation; GOTERM_BP_5 GO:0030193~regulation of blood coagulation; GOTERM_BP_4 GO:0050818~regulation of coagulation; GOTERM_BP_3 GO:0050818~regulation of coagulation; GOTERM_BP_4 GO:0042060~wound healing; GOTERM_BP_4 GO:0007596~blood coagulation; GOTERM_BP_3 GO:0007596~blood coagulation; GOTERM_BP_2 GO:0050817~coagulation; GOTERM_MF_3 GO:0019838~growth factor binding; GOTERM_BP_4 GO:0007599~hemostasis; GOTERM_BP_5 GO:0007596~blood coagulation; GOTERM_BP_3 GO:0007599~hemostasis; GOTERM_BP_2 GO:0009605~response to external stimulus; GOTERM_BP_3 GO:0050878~regulation of body fluid levels; GOTERM_BP_2 GO:0050878~regulation of body fluid levels; GOTERM_BP_4 GO:0032101~regulation of response to external stimulus; GOTERM_BP_4 GO:0030195~negative regulation of blood coagulation; GOTERM_BP_3 GO:0032101~regulation of response to external stimulus; GOTERM_BP_5 GO:0030195~negative regulation of blood coagulation; GOTERM_BP_4 GO:0050819~negative regulation of coagulation; GOTERM_BP_3 GO:0050819~negative regulation of coagulation; GOTERM_BP_5 GO:0050819~negative regulation of coagulation; GOTERM_BP_2 GO:0006950~response to stress; GOTERM_BP_3 GO:0009611~response to wounding; GOTERM_BP_4 GO:0080134~regulation of response to stress; GOTERM_BP_3 GO:0080134~regulation of response to stress; GOTERM_BP_3 GO:0048583~regulation of response to stimulus; GOTERM_BP_2 GO:0048583~regulation of response to stimulus; GOTERM_BP_2 GO:0065008~regulation of biological quality; GOTERM_BP_3 GO:0048519~negative regulation of biological process; GOTERM_BP_4 GO:0051241~negative regulation of multicellular organismal process; GOTERM_BP_2 GO:0048519~negative regulation of biological process; GOTERM_BP_3 GO:0051241~negative regulation of multicellular organismal process; GOTERM_BP_2 GO:0051241~negative regulation of multicellular organismal process; GOTERM_BP_1 GO:0050896~response to stimulus; GOTERM_BP_3 GO:0048518~positive regulation of biological process; GOTERM_BP_2 GO:0048518~positive regulation of biological process; GOTERM_BP_3 GO:0030334~regulation of cell migration; GOTERM_BP_4 GO:0048522~positive regulation of cellular process; GOTERM_BP_3 GO:0048522~positive regulation of cellular process; GOTERM_BP_2 GO:0048522~positive regulation of cellular process; GOTERM_BP_2 GO:0042221~response to chemical stimulus; GOTERM_BP_3 GO:0010033~response to organic substance; GOTERM_BP_4 GO:0051240~positive regulation of multicellular organismal process; GOTERM_BP_3 GO:0051240~positive regulation of multicellular organismal process; GOTERM_BP_2 GO:0051240~positive regulation of multicellular organismal process; GOTERM_BP_5 GO:0002526~acute inflammatory response; GOTERM_BP_4 GO:0006954~inflammatory response; GOTERM_MF_3 GO:0005102~receptor binding; GOTERM_BP_3 GO:0006952~defense response |
| 4287-2437 |  |
| 4287-3895 | GOTERM_CC_1 GO:0044421~extracellular region part; GOTERM_CC_2 GO:0044421~extracellular region part; GOTERM_CC_1 GO:0005576~extracellular region; GOTERM_CC_2 GO:0005615~extracellular space; GOTERM_CC_3 GO:0005615~extracellular space; GOTERM_MF_5 GO:0004175~endopeptidase activity; GOTERM_MF_5 GO:0004252~serine-type endopeptidase activity; GOTERM_MF_5 GO:0008236~serine-type peptidase activity; GOTERM_MF_3 GO:0008233~peptidase activity; GOTERM_MF_4 GO:0070011~peptidase activity, acting on L-amino acid peptides; GOTERM_BP_4 GO:0030193~regulation of blood coagulation; GOTERM_BP_5 GO:0030193~regulation of blood coagulation; GOTERM_MF_3 GO:0017171~serine hydrolase activity; GOTERM_MF_4 GO:0008236~serine-type peptidase activity; GOTERM_BP_4 GO:0042060~wound healing; GOTERM_BP_4 GO:0007596~blood coagulation; GOTERM_BP_3 GO:0007596~blood coagulation; GOTERM_BP_2 GO:0050817~coagulation; GOTERM_BP_4 GO:0007599~hemostasis; GOTERM_BP_5 GO:0007596~blood coagulation; GOTERM_BP_3 GO:0007599~hemostasis; GOTERM_BP_2 GO:0009605~response to external stimulus; GOTERM_BP_3 GO:0050878~regulation of body fluid levels; GOTERM_BP_2 GO:0050878~regulation of body fluid levels; GOTERM_BP_4 GO:0030195~negative regulation of blood coagulation; GOTERM_BP_1 GO:0032501~multicellular organismal process; GOTERM_BP_5 GO:0030195~negative regulation of blood coagulation; GOTERM_BP_4 GO:0050819~negative regulation of coagulation; GOTERM_BP_3 GO:0050819~negative regulation of coagulation; GOTERM_BP_5 GO:0050819~negative regulation of coagulation; GOTERM_BP_2 GO:0006950~response to stress; GOTERM_BP_1 GO:0022610~biological adhesion; GOTERM_BP_2 GO:0007155~cell adhesion; GOTERM_BP_3 GO:0009611~response to wounding; GOTERM_BP_5 GO:0006508~proteolysis; GOTERM_BP_2 GO:0065008~regulation of biological quality; GOTERM_BP_1 GO:0050896~response to stimulus; GOTERM_MF_3 GO:0005102~receptor binding; GOTERM_MF_4 GO:0005539~glycosaminoglycan binding; GOTERM_CC_5 GO:0030141~secretory granule; GOTERM_BP_1 GO:0065007~biological regulation; GOTERM_MF_3 GO:0030247~polysaccharide binding; GOTERM_MF_2 GO:0001871~pattern binding; GOTERM_CC_5 GO:0016023~cytoplasmic membrane-bounded vesicle |
| 4287-4878 | GOTERM_CC_2 GO:0031012~extracellular matrix; GOTERM_CC_3 GO:0031012~extracellular matrix; GOTERM_CC_1 GO:0044421~extracellular region part; GOTERM_CC_4 GO:0005578~proteinaceous extracellular matrix; GOTERM_CC_2 GO:0044421~extracellular region part; GOTERM_CC_3 GO:0005578~proteinaceous extracellular matrix; GOTERM_CC_1 GO:0005576~extracellular region; GOTERM_CC_2 GO:0005615~extracellular space; GOTERM_CC_3 GO:0005615~extracellular space; GOTERM_MF_5 GO:0004175~endopeptidase activity; GOTERM_MF_5 GO:0004252~serine-type endopeptidase activity; GOTERM_MF_5 GO:0008236~serine-type peptidase activity; GOTERM_MF_3 GO:0008233~peptidase activity; GOTERM_MF_4 GO:0070011~peptidase activity, acting on L-amino acid peptides; GOTERM_MF_5 GO:0005509~calcium ion binding; GOTERM_MF_3 GO:0017171~serine hydrolase activity; GOTERM_MF_4 GO:0008236~serine-type peptidase activity; GOTERM_BP_4 GO:0042060~wound healing; GOTERM_BP_4 GO:0007596~blood coagulation; GOTERM_BP_3 GO:0007596~blood coagulation; GOTERM_BP_2 GO:0050817~coagulation; GOTERM_BP_4 GO:0007599~hemostasis; GOTERM_BP_5 GO:0007596~blood coagulation; GOTERM_BP_3 GO:0007599~hemostasis; GOTERM_BP_2 GO:0009605~response to external stimulus; GOTERM_BP_3 GO:0050878~regulation of body fluid levels; GOTERM_BP_2 GO:0050878~regulation of body fluid levels; GOTERM_BP_4 GO:0032101~regulation of response to external stimulus; GOTERM_BP_1 GO:0032501~multicellular organismal process; GOTERM_BP_3 GO:0032101~regulation of response to external stimulus; GOTERM_BP_4 GO:0032963~collagen metabolic process; GOTERM_BP_2 GO:0006950~response to stress; GOTERM_BP_3 GO:0044259~multicellular organismal macromolecule metabolic process; GOTERM_BP_3 GO:0009611~response to wounding; GOTERM_BP_2 GO:0044236~multicellular organismal metabolic process; GOTERM_BP_5 GO:0006508~proteolysis; GOTERM_BP_1 GO:0040011~locomotion; GOTERM_BP_4 GO:0080134~regulation of response to stress; GOTERM_BP_3 GO:0080134~regulation of response to stress; GOTERM_BP_3 GO:0048583~regulation of response to stimulus; GOTERM_BP_2 GO:0048583~regulation of response to stimulus; GOTERM_BP_2 GO:0065008~regulation of biological quality; GOTERM_BP_1 GO:0050896~response to stimulus; GOTERM_BP_2 GO:0042221~response to chemical stimulus; GOTERM_MF_5 GO:0008201~heparin binding; GOTERM_BP_5 GO:0002526~acute inflammatory response; GOTERM_BP_4 GO:0006954~inflammatory response; GOTERM_BP_3 GO:0006952~defense response; GOTERM_MF_2 GO:0016787~hydrolase activity; GOTERM_MF_4 GO:0005539~glycosaminoglycan binding; GOTERM_CC_5 GO:0030141~secretory granule; GOTERM_BP_1 GO:0065007~biological regulation; GOTERM_MF_3 GO:0030247~polysaccharide binding; GOTERM_BP_4 GO:0030574~collagen catabolic process; GOTERM_MF_2 GO:0001871~pattern binding; GOTERM_BP_5 GO:0030574~collagen catabolic process; GOTERM_BP_3 GO:0044243~multicellular organismal catabolic process; GOTERM_CC_5 GO:0016023~cytoplasmic membrane-bounded vesicle |
| 4287-5316 | GOTERM_CC_1 GO:0044421~extracellular region part; GOTERM_CC_2 GO:0044421~extracellular region part; GOTERM_CC_1 GO:0005576~extracellular region; GOTERM_CC_2 GO:0005615~extracellular space; GOTERM_CC_3 GO:0005615~extracellular space; GOTERM_MF_5 GO:0004175~endopeptidase activity; GOTERM_MF_3 GO:0008233~peptidase activity; GOTERM_MF_4 GO:0070011~peptidase activity, acting on L-amino acid peptides; GOTERM_BP_5 GO:0006508~proteolysis; GOTERM_BP_4 GO:0019538~protein metabolic process; GOTERM_BP_3 GO:0019538~protein metabolic process; GOTERM_MF_2 GO:0016787~hydrolase activity; GOTERM_MF_5 GO:0008237~metallopeptidase activity |
| 4287-5011 | GOTERM_CC_2 GO:0031012~extracellular matrix; GOTERM_CC_3 GO:0031012~extracellular matrix; GOTERM_CC_4 GO:0005578~proteinaceous extracellular matrix; GOTERM_CC_3 GO:0005578~proteinaceous extracellular matrix; GOTERM_BP_3 GO:0009653~anatomical structure morphogenesis; GOTERM_BP_2 GO:0009653~anatomical structure morphogenesis; GOTERM_BP_4 GO:0048513~organ development; GOTERM_BP_3 GO:0048513~organ development; GOTERM_BP_2 GO:0048856~anatomical structure development; GOTERM_BP_4 GO:0016477~cell migration; GOTERM_BP_3 GO:0016477~cell migration; GOTERM_BP_1 GO:0032501~multicellular organismal process; GOTERM_BP_5 GO:0016477~cell migration; GOTERM_BP_4 GO:0048870~cell motility; GOTERM_BP_3 GO:0048870~cell motility; GOTERM_BP_2 GO:0048870~cell motility; GOTERM_BP_2 GO:0051674~localization of cell; GOTERM_BP_1 GO:0032502~developmental process; GOTERM_BP_2 GO:0007275~multicellular organismal development; GOTERM_BP_1 GO:0040011~locomotion; GOTERM_BP_3 GO:0006928~cell motion; GOTERM_BP_2 GO:0006928~cell motion; GOTERM_BP_1 GO:0016043~cellular component organization; GOTERM_MF_2 GO:0005515~protein binding; GOTERM_BP_4 GO:0048598~embryonic morphogenesis; GOTERM_BP_2 GO:0050789~regulation of biological process; GOTERM_BP_3 GO:0048598~embryonic morphogenesis; GOTERM_BP_3 GO:0009790~embryonic development; GOTERM_BP_2 GO:0009790~embryonic development; GOTERM_BP_1 GO:0065007~biological regulation |
| 4287-5699 | GOTERM_CC_1 GO:0005576~extracellular region; GOTERM_CC_2 GO:0005615~extracellular space; GOTERM_CC_3 GO:0005615~extracellular space; GOTERM_MF_5 GO:0005509~calcium ion binding; GOTERM_BP_2 GO:0006950~response to stress; GOTERM_BP_3 GO:0009611~response to wounding; GOTERM_BP_1 GO:0050896~response to stimulus; GOTERM_MF_2 GO:0005515~protein binding; GOTERM_BP_5 GO:0002526~acute inflammatory response; GOTERM_BP_4 GO:0006954~inflammatory response; GOTERM_BP_3 GO:0032879~regulation of localization; GOTERM_BP_2 GO:0032879~regulation of localization; GOTERM_BP_3 GO:0006952~defense response |
| 4287-6320 | GOTERM_CC_2 GO:0031012~extracellular matrix; GOTERM_CC_3 GO:0031012~extracellular matrix; GOTERM_CC_1 GO:0044421~extracellular region part; GOTERM_CC_4 GO:0005578~proteinaceous extracellular matrix; GOTERM_CC_2 GO:0044421~extracellular region part; GOTERM_CC_3 GO:0005578~proteinaceous extracellular matrix; GOTERM_CC_4 GO:0044420~extracellular matrix part; GOTERM_CC_2 GO:0044420~extracellular matrix part; GOTERM_CC_3 GO:0044420~extracellular matrix part; GOTERM_BP_2 GO:0043062~extracellular structure organization; GOTERM_BP_1 GO:0032501~multicellular organismal process; GOTERM_BP_1 GO:0022610~biological adhesion; GOTERM_BP_2 GO:0007155~cell adhesion; GOTERM_BP_2 GO:0065008~regulation of biological quality; GOTERM_MF_2 GO:0005515~protein binding; GOTERM_CC_5 GO:0005605~basal lamina; GOTERM_CC_4 GO:0005605~basal lamina; GOTERM_CC_3 GO:0005605~basal lamina |
| 4287-4958 | GOTERM_CC_1 GO:0044421~extracellular region part; GOTERM_CC_2 GO:0044421~extracellular region part; GOTERM_CC_1 GO:0005576~extracellular region; GOTERM_CC_2 GO:0005615~extracellular space; GOTERM_CC_3 GO:0005615~extracellular space; GOTERM_BP_3 GO:0051239~regulation of multicellular organismal process; GOTERM_BP_2 GO:0051239~regulation of multicellular organismal process; GOTERM_BP_4 GO:0048513~organ development; GOTERM_BP_3 GO:0048513~organ development; GOTERM_BP_2 GO:0048856~anatomical structure development; GOTERM_BP_4 GO:0032101~regulation of response to external stimulus; GOTERM_BP_1 GO:0032501~multicellular organismal process; GOTERM_BP_3 GO:0032101~regulation of response to external stimulus; GOTERM_BP_1 GO:0032502~developmental process; GOTERM_BP_2 GO:0007275~multicellular organismal development; GOTERM_BP_3 GO:0048731~system development; GOTERM_BP_4 GO:0080134~regulation of response to stress; GOTERM_BP_3 GO:0080134~regulation of response to stress; GOTERM_BP_3 GO:0048583~regulation of response to stimulus; GOTERM_BP_3 GO:0050793~regulation of developmental process; GOTERM_BP_2 GO:0048583~regulation of response to stimulus; GOTERM_BP_2 GO:0050793~regulation of developmental process; GOTERM_BP_3 GO:0048519~negative regulation of biological process; GOTERM_BP_2 GO:0048519~negative regulation of biological process; GOTERM_BP_3 GO:0048518~positive regulation of biological process; GOTERM_BP_2 GO:0048518~positive regulation of biological process; GOTERM_BP_4 GO:0048522~positive regulation of cellular process; GOTERM_BP_3 GO:0048522~positive regulation of cellular process; GOTERM_BP_2 GO:0048522~positive regulation of cellular process; GOTERM_MF_2 GO:0005515~protein binding; GOTERM_BP_4 GO:0042127~regulation of cell proliferation; GOTERM_BP_3 GO:0042127~regulation of cell proliferation; GOTERM_BP_2 GO:0050789~regulation of biological process; GOTERM_MF_3 GO:0005102~receptor binding; GOTERM_BP_3 GO:0032879~regulation of localization; GOTERM_BP_2 GO:0032879~regulation of localization; GOTERM_BP_1 GO:0065007~biological regulation |
| 4287-3847 | GOTERM_CC_1 GO:0005576~extracellular region; GOTERM_BP_4 GO:0042060~wound healing; GOTERM_BP_4 GO:0007596~blood coagulation; GOTERM_BP_3 GO:0007596~blood coagulation; GOTERM_BP_2 GO:0050817~coagulation; GOTERM_BP_4 GO:0007599~hemostasis; GOTERM_BP_5 GO:0007596~blood coagulation; GOTERM_BP_3 GO:0007599~hemostasis; GOTERM_BP_3 GO:0050878~regulation of body fluid levels; GOTERM_BP_2 GO:0050878~regulation of body fluid levels; GOTERM_BP_1 GO:0022610~biological adhesion; GOTERM_BP_2 GO:0007155~cell adhesion; GOTERM_BP_3 GO:0009611~response to wounding; GOTERM_BP_1 GO:0040011~locomotion; GOTERM_BP_2 GO:0065008~regulation of biological quality; GOTERM_MF_4 GO:0005178~integrin binding; GOTERM_MF_3 GO:0032403~protein complex binding; GOTERM_CC_4 GO:0005577~fibrinogen complex; GOTERM_CC_2 GO:0005577~fibrinogen complex; GOTERM_CC_3 GO:0005577~fibrinogen complex; GOTERM_MF_3 GO:0005102~receptor binding; GOTERM_CC_5 GO:0030141~secretory granule; GOTERM_CC_5 GO:0016023~cytoplasmic membrane-bounded vesicle |
| 4287-2305 | GOTERM_BP_3 GO:0009611~response to wounding; GOTERM_BP_1 GO:0040011~locomotion; GOTERM_BP_4 GO:0006954~inflammatory response; GOTERM_BP_3 GO:0006952~defense response |
| 4287-6248 | GOTERM_CC_2 GO:0031012~extracellular matrix; GOTERM_CC_3 GO:0031012~extracellular matrix; GOTERM_CC_1 GO:0044421~extracellular region part; GOTERM_CC_4 GO:0005578~proteinaceous extracellular matrix; GOTERM_CC_2 GO:0044421~extracellular region part; GOTERM_CC_3 GO:0005578~proteinaceous extracellular matrix; GOTERM_CC_4 GO:0044420~extracellular matrix part; GOTERM_CC_2 GO:0044420~extracellular matrix part; GOTERM_CC_3 GO:0044420~extracellular matrix part; GOTERM_CC_1 GO:0005576~extracellular region; GOTERM_CC_5 GO:0005604~basement membrane; GOTERM_CC_4 GO:0005604~basement membrane; GOTERM_CC_3 GO:0005604~basement membrane; GOTERM_CC_2 GO:0005615~extracellular space; GOTERM_CC_3 GO:0005615~extracellular space; GOTERM_BP_3 GO:0030198~extracellular matrix organization; GOTERM_BP_2 GO:0043062~extracellular structure organization; GOTERM_MF_2 GO:0005515~protein binding; GOTERM_BP_4 GO:0042127~regulation of cell proliferation; GOTERM_BP_3 GO:0042127~regulation of cell proliferation |
| 4287-5822 | GOTERM_BP_3 GO:0009653~anatomical structure morphogenesis; GOTERM_BP_2 GO:0009653~anatomical structure morphogenesis; GOTERM_BP_4 GO:0032101~regulation of response to external stimulus; GOTERM_BP_3 GO:0032101~regulation of response to external stimulus; GOTERM_BP_1 GO:0022610~biological adhesion; GOTERM_BP_2 GO:0007155~cell adhesion |
| 4287-6404 | GOTERM_BP_2 GO:0048519~negative regulation of biological process; GOTERM_BP_3 GO:0048518~positive regulation of biological process; GOTERM_BP_2 GO:0048518~positive regulation of biological process; GOTERM_BP_1 GO:0016043~cellular component organization; GOTERM_BP_4 GO:0048522~positive regulation of cellular process; GOTERM_BP_3 GO:0048522~positive regulation of cellular process; GOTERM_BP_2 GO:0048522~positive regulation of cellular process; GOTERM_MF_2 GO:0005515~protein binding; GOTERM_BP_2 GO:0050789~regulation of biological process; GOTERM_BP_1 GO:0065007~biological regulation |
| 4287-4305 | GOTERM_CC_1 GO:0005576~extracellular region; GOTERM_BP_4 GO:0030155~regulation of cell adhesion; GOTERM_BP_3 GO:0030155~regulation of cell adhesion; GOTERM_BP_4 GO:0001944~vasculature development; GOTERM_BP_5 GO:0001568~blood vessel development; GOTERM_BP_5 GO:0001944~vasculature development; GOTERM_BP_3 GO:0009653~anatomical structure morphogenesis; GOTERM_BP_2 GO:0043062~extracellular structure organization; GOTERM_MF_5 GO:0005509~calcium ion binding; GOTERM_BP_2 GO:0009653~anatomical structure morphogenesis; GOTERM_MF_3 GO:0019838~growth factor binding; GOTERM_BP_4 GO:0010810~regulation of cell-substrate adhesion; GOTERM_BP_5 GO:0010810~regulation of cell-substrate adhesion; GOTERM_BP_4 GO:0048513~organ development; GOTERM_BP_3 GO:0048513~organ development; GOTERM_BP_2 GO:0048856~anatomical structure development; GOTERM_BP_1 GO:0032502~developmental process; GOTERM_BP_1 GO:0022610~biological adhesion; GOTERM_BP_2 GO:0007155~cell adhesion; GOTERM_BP_2 GO:0007275~multicellular organismal development; GOTERM_BP_3 GO:0048731~system development; GOTERM_BP_1 GO:0040011~locomotion; GOTERM_BP_3 GO:0050793~regulation of developmental process; GOTERM_BP_2 GO:0050793~regulation of developmental process; GOTERM_MF_4 GO:0005178~integrin binding; GOTERM_BP_3 GO:0040012~regulation of locomotion; GOTERM_BP_2 GO:0040012~regulation of locomotion; GOTERM_BP_4 GO:0007160~cell-matrix adhesion; GOTERM_BP_3 GO:0031589~cell-substrate adhesion; GOTERM_BP_4 GO:0010811~positive regulation of cell-substrate adhesion; GOTERM_MF_2 GO:0050840~extracellular matrix binding; GOTERM_BP_5 GO:0010811~positive regulation of cell-substrate adhesion; GOTERM_BP_3 GO:0048518~positive regulation of biological process; GOTERM_BP_2 GO:0048518~positive regulation of biological process; GOTERM_BP_4 GO:0030334~regulation of cell migration; GOTERM_BP_4 GO:0048646~anatomical structure formation involved in morphogenesis; GOTERM_BP_3 GO:0030334~regulation of cell migration; GOTERM_BP_4 GO:0048522~positive regulation of cellular process; GOTERM_BP_3 GO:0048646~anatomical structure formation involved in morphogenesis; GOTERM_BP_2 GO:0048646~anatomical structure formation involved in morphogenesis; GOTERM_BP_5 GO:0030334~regulation of cell migration; GOTERM_BP_3 GO:0048522~positive regulation of cellular process; GOTERM_BP_2 GO:0048522~positive regulation of cellular process; GOTERM_BP_4 GO:0051270~regulation of cell motion; GOTERM_BP_4 GO:0045785~positive regulation of cell adhesion; GOTERM_BP_3 GO:0051270~regulation of cell motion; GOTERM_MF_3 GO:0032403~protein complex binding; GOTERM_BP_3 GO:0045785~positive regulation of cell adhesion; GOTERM_MF_2 GO:0005515~protein binding; GOTERM_BP_5 GO:0045785~positive regulation of cell adhesion; GOTERM_BP_4 GO:0048514~blood vessel morphogenesis; GOTERM_BP_5 GO:0048514~blood vessel morphogenesis; GOTERM_BP_4 GO:0042127~regulation of cell proliferation; GOTERM_BP_3 GO:0042127~regulation of cell proliferation; GOTERM_BP_2 GO:0050789~regulation of biological process; GOTERM_MF_3 GO:0005102~receptor binding; GOTERM_BP_3 GO:0032879~regulation of localization; GOTERM_BP_2 GO:0032879~regulation of localization; GOTERM_BP_4 GO:0001525~angiogenesis; GOTERM_CC_5 GO:0030141~secretory granule; GOTERM_BP_3 GO:0001525~angiogenesis; GOTERM_BP_1 GO:0065007~biological regulation; GOTERM_BP_5 GO:0001525~angiogenesis; GOTERM_BP_4 GO:0007044~cell-substrate junction assembly |
| 4287-6433 | GOTERM_CC_2 GO:0031012~extracellular matrix; GOTERM_CC_3 GO:0031012~extracellular matrix; GOTERM_CC_1 GO:0044421~extracellular region part; GOTERM_CC_4 GO:0005578~proteinaceous extracellular matrix; GOTERM_CC_2 GO:0044421~extracellular region part; GOTERM_CC_3 GO:0005578~proteinaceous extracellular matrix; GOTERM_CC_1 GO:0005576~extracellular region; GOTERM_CC_2 GO:0005615~extracellular space; GOTERM_CC_3 GO:0005615~extracellular space; GOTERM_BP_2 GO:0009605~response to external stimulus; GOTERM_BP_2 GO:0006950~response to stress; GOTERM_BP_3 GO:0009611~response to wounding; GOTERM_BP_3 GO:0048519~negative regulation of biological process; GOTERM_BP_2 GO:0048519~negative regulation of biological process; GOTERM_BP_1 GO:0050896~response to stimulus; GOTERM_MF_2 GO:0005515~protein binding; GOTERM_BP_4 GO:0042127~regulation of cell proliferation; GOTERM_BP_3 GO:0042127~regulation of cell proliferation; GOTERM_BP_4 GO:0006954~inflammatory response; GOTERM_BP_3 GO:0006952~defense response; GOTERM_CC_5 GO:0030141~secretory granule; GOTERM_CC_5 GO:0016023~cytoplasmic membrane-bounded vesicle |
| 4287-6454 | GOTERM_BP_4 GO:0030193~regulation of blood coagulation; GOTERM_BP_5 GO:0030193~regulation of blood coagulation; GOTERM_BP_4 GO:0050818~regulation of coagulation; GOTERM_BP_3 GO:0050818~regulation of coagulation |
| 4287-6410 | GOTERM_MF_3 GO:0016504~peptidase activator activity |
| 4945-5219 | GOTERM_CC_1 GO:0005576~extracellular region; GOTERM_BP_4 GO:0007399~nervous system development; GOTERM_CC_2 GO:0005615~extracellular space; GOTERM_CC_3 GO:0005615~extracellular space; GOTERM_BP_1 GO:0032501~multicellular organismal process; GOTERM_CC_1 GO:0044421~extracellular region part; GOTERM_BP_2 GO:0007275~multicellular organismal development; GOTERM_CC_2 GO:0044421~extracellular region part; GOTERM_BP_1 GO:0032502~developmental process; GOTERM_CC_3 GO:0031988~membrane-bounded vesicle; GOTERM_CC_4 GO:0016023~cytoplasmic membrane-bounded vesicle; GOTERM_CC_5 GO:0016023~cytoplasmic membrane-bounded vesicle; GOTERM_BP_3 GO:0048731~system development; GOTERM_CC_2 GO:0031982~vesicle; GOTERM_CC_5 GO:0031410~cytoplasmic vesicle; GOTERM_BP_2 GO:0048856~anatomical structure development; GOTERM_MF_2 GO:0005515~protein binding; GOTERM_BP_1 GO:0051704~multi-organism process; GOTERM_BP_1 GO:0022610~biological adhesion; GOTERM_BP_2 GO:0007155~cell adhesion |
| 4945-4393 | GOTERM_MF_5 GO:0005509~calcium ion binding; GOTERM_BP_3 GO:0050790~regulation of catalytic activity; GOTERM_BP_2 GO:0065009~regulation of molecular function; GOTERM_CC_3 GO:0044459~plasma membrane part; GOTERM_CC_4 GO:0044459~plasma membrane part; GOTERM_CC_3 GO:0005886~plasma membrane; GOTERM_CC_5 GO:0044459~plasma membrane part; GOTERM_CC_4 GO:0005886~plasma membrane; GOTERM_CC_3 GO:0031988~membrane-bounded vesicle; GOTERM_CC_4 GO:0016023~cytoplasmic membrane-bounded vesicle; GOTERM_CC_5 GO:0016023~cytoplasmic membrane-bounded vesicle; GOTERM_BP_4 GO:0043085~positive regulation of catalytic activity; GOTERM_CC_3 GO:0031410~cytoplasmic vesicle; GOTERM_BP_3 GO:0044093~positive regulation of molecular function; GOTERM_CC_4 GO:0031410~cytoplasmic vesicle; GOTERM_CC_2 GO:0031982~vesicle; GOTERM_CC_5 GO:0031410~cytoplasmic vesicle; GOTERM_CC_3 GO:0005626~insoluble fraction; GOTERM_CC_4 GO:0005624~membrane fraction; GOTERM_CC_5 GO:0005624~membrane fraction; GOTERM_CC_4 GO:0005626~insoluble fraction; GOTERM_CC_2 GO:0000267~cell fraction; GOTERM_CC_3 GO:0000267~cell fraction; GOTERM_CC_3 GO:0031224~intrinsic to membrane; GOTERM_CC_4 GO:0016021~integral to membrane; GOTERM_CC_5 GO:0016021~integral to membrane; GOTERM_CC_4 GO:0031224~intrinsic to membrane; GOTERM_CC_5 GO:0031224~intrinsic to membrane; GOTERM_CC_5 GO:0005887~integral to plasma membrane; GOTERM_MF_2 GO:0005515~protein binding; GOTERM_CC_4 GO:0031226~intrinsic to plasma membrane; GOTERM_CC_5 GO:0031226~intrinsic to plasma membrane; GOTERM_BP_2 GO:0006928~cell motion; GOTERM_BP_3 GO:0006928~cell motion; GOTERM_CC_2 GO:0044425~membrane part; GOTERM_CC_3 GO:0044425~membrane part; GOTERM_CC_4 GO:0044425~membrane part; GOTERM_BP_1 GO:0022610~biological adhesion; GOTERM_BP_2 GO:0007155~cell adhesion; GOTERM_CC_2 GO:0016020~membrane; GOTERM_CC_3 GO:0016020~membrane |
| 4945-4944 | GOTERM_CC_1 GO:0005576~extracellular region; GOTERM_CC_2 GO:0005615~extracellular space; GOTERM_CC_3 GO:0005615~extracellular space; GOTERM_CC_1 GO:0044421~extracellular region part; GOTERM_CC_2 GO:0044421~extracellular region part; GOTERM_BP_2 GO:0065008~regulation of biological quality |
| 4945-5982 | GOTERM_BP_5 GO:0043549~regulation of kinase activity; GOTERM_BP_4 GO:0051338~regulation of transferase activity; GOTERM_BP_5 GO:0019220~regulation of phosphate metabolic process; GOTERM_BP_5 GO:0051174~regulation of phosphorus metabolic process; GOTERM_BP_4 GO:0051174~regulation of phosphorus metabolic process; GOTERM_BP_1 GO:0032501~multicellular organismal process; GOTERM_BP_2 GO:0007275~multicellular organismal development; GOTERM_BP_1 GO:0032502~developmental process; GOTERM_BP_3 GO:0048731~system development; GOTERM_BP_2 GO:0048856~anatomical structure development; GOTERM_MF_2 GO:0005515~protein binding |
| 4945-2437 | GOTERM_MF_2 GO:0043167~ion binding; GOTERM_MF_3 GO:0043169~cation binding; GOTERM_MF_4 GO:0046872~metal ion binding |
| 4945-3895 | GOTERM_CC_1 GO:0005576~extracellular region; GOTERM_CC_2 GO:0005615~extracellular space; GOTERM_CC_3 GO:0005615~extracellular space; GOTERM_BP_1 GO:0032501~multicellular organismal process; GOTERM_CC_1 GO:0044421~extracellular region part; GOTERM_CC_2 GO:0044421~extracellular region part; GOTERM_CC_3 GO:0005886~plasma membrane; GOTERM_CC_4 GO:0005886~plasma membrane; GOTERM_CC_3 GO:0031988~membrane-bounded vesicle; GOTERM_CC_4 GO:0016023~cytoplasmic membrane-bounded vesicle; GOTERM_CC_5 GO:0016023~cytoplasmic membrane-bounded vesicle; GOTERM_CC_3 GO:0031410~cytoplasmic vesicle; GOTERM_CC_4 GO:0031410~cytoplasmic vesicle; GOTERM_CC_2 GO:0031982~vesicle; GOTERM_CC_5 GO:0031410~cytoplasmic vesicle; GOTERM_BP_2 GO:0065008~regulation of biological quality; GOTERM_BP_1 GO:0022610~biological adhesion; GOTERM_BP_2 GO:0007155~cell adhesion |
| 4945-4878 | GOTERM_MF_5 GO:0005509~calcium ion binding; GOTERM_CC_1 GO:0005576~extracellular region; GOTERM_CC_2 GO:0005615~extracellular space; GOTERM_CC_3 GO:0005615~extracellular space; GOTERM_BP_1 GO:0032501~multicellular organismal process; GOTERM_CC_1 GO:0044421~extracellular region part; GOTERM_CC_2 GO:0044421~extracellular region part; GOTERM_CC_3 GO:0031988~membrane-bounded vesicle; GOTERM_CC_4 GO:0016023~cytoplasmic membrane-bounded vesicle; GOTERM_CC_5 GO:0016023~cytoplasmic membrane-bounded vesicle; GOTERM_CC_3 GO:0031410~cytoplasmic vesicle; GOTERM_CC_4 GO:0031410~cytoplasmic vesicle; GOTERM_CC_2 GO:0031982~vesicle; GOTERM_CC_5 GO:0031410~cytoplasmic vesicle; GOTERM_BP_2 GO:0007610~behavior; GOTERM_BP_2 GO:0065008~regulation of biological quality; GOTERM_BP_1 GO:0051704~multi-organism process |
| 4945-5011 | GOTERM_BP_5 GO:0006897~endocytosis; GOTERM_BP_3 GO:0006897~endocytosis; GOTERM_BP_3 GO:0010324~membrane invagination; GOTERM_BP_4 GO:0006897~endocytosis; GOTERM_BP_2 GO:0016044~membrane organization; GOTERM_BP_4 GO:0007399~nervous system development; GOTERM_BP_1 GO:0016043~cellular component organization; GOTERM_BP_1 GO:0032501~multicellular organismal process; GOTERM_BP_5 GO:0007420~brain development; GOTERM_BP_4 GO:0007420~brain development; GOTERM_BP_2 GO:0007275~multicellular organismal development; GOTERM_BP_1 GO:0032502~developmental process; GOTERM_BP_5 GO:0007417~central nervous system development; GOTERM_BP_4 GO:0007417~central nervous system development; GOTERM_BP_2 GO:0048856~anatomical structure development; GOTERM_MF_3 GO:0004872~receptor activity; GOTERM_CC_5 GO:0016021~integral to membrane; GOTERM_MF_1 GO:0060089~molecular transducer activity; GOTERM_MF_2 GO:0004871~signal transducer activity; GOTERM_CC_5 GO:0031224~intrinsic to membrane; GOTERM_MF_2 GO:0005515~protein binding; GOTERM_BP_2 GO:0009653~anatomical structure morphogenesis; GOTERM_BP_3 GO:0009653~anatomical structure morphogenesis; GOTERM_BP_2 GO:0006928~cell motion; GOTERM_BP_3 GO:0006928~cell motion; GOTERM_CC_2 GO:0044425~membrane part; GOTERM_CC_3 GO:0044425~membrane part; GOTERM_CC_4 GO:0044425~membrane part |
| 4945-6126 | GOTERM_MF_2 GO:0043167~ion binding; GOTERM_BP_1 GO:0016043~cellular component organization; GOTERM_MF_3 GO:0043169~cation binding; GOTERM_MF_4 GO:0046872~metal ion binding; GOTERM_MF_2 GO:0005515~protein binding; GOTERM_CC_2 GO:0032994~protein-lipid complex; GOTERM_CC_2 GO:0034358~plasma lipoprotein particle; GOTERM_CC_3 GO:0034358~plasma lipoprotein particle; GOTERM_CC_4 GO:0034358~plasma lipoprotein particle |
| 4945-6240 | GOTERM_MF_5 GO:0005509~calcium ion binding; GOTERM_CC_2 GO:0012505~endomembrane system; GOTERM_CC_3 GO:0012505~endomembrane system; GOTERM_CC_1 GO:0044421~extracellular region part; GOTERM_CC_2 GO:0044421~extracellular region part; GOTERM_BP_1 GO:0051179~localization; GOTERM_CC_3 GO:0005886~plasma membrane; GOTERM_CC_4 GO:0005886~plasma membrane; GOTERM_CC_3 GO:0031988~membrane-bounded vesicle; GOTERM_CC_4 GO:0016023~cytoplasmic membrane-bounded vesicle; GOTERM_CC_5 GO:0016023~cytoplasmic membrane-bounded vesicle; GOTERM_CC_3 GO:0031410~cytoplasmic vesicle; GOTERM_CC_4 GO:0031410~cytoplasmic vesicle; GOTERM_CC_2 GO:0031982~vesicle; GOTERM_CC_5 GO:0031410~cytoplasmic vesicle; GOTERM_BP_2 GO:0006810~transport; GOTERM_BP_1 GO:0051234~establishment of localization; GOTERM_BP_2 GO:0051234~establishment of localization; GOTERM_BP_3 GO:0006810~transport; GOTERM_CC_3 GO:0005626~insoluble fraction; GOTERM_CC_4 GO:0005624~membrane fraction; GOTERM_CC_5 GO:0005624~membrane fraction; GOTERM_CC_4 GO:0005626~insoluble fraction; GOTERM_BP_4 GO:0034381~lipoprotein particle clearance; GOTERM_CC_2 GO:0000267~cell fraction; GOTERM_CC_3 GO:0000267~cell fraction; GOTERM_MF_2 GO:0005515~protein binding; GOTERM_BP_2 GO:0065008~regulation of biological quality |
| 4945-6320 | GOTERM_BP_1 GO:0032501~multicellular organismal process; GOTERM_CC_1 GO:0044421~extracellular region part; GOTERM_CC_2 GO:0044421~extracellular region part; GOTERM_MF_2 GO:0005515~protein binding; GOTERM_BP_2 GO:0007610~behavior; GOTERM_BP_2 GO:0065008~regulation of biological quality; GOTERM_BP_1 GO:0022610~biological adhesion; GOTERM_BP_2 GO:0007155~cell adhesion |
| 4945-4958 | GOTERM_CC_1 GO:0005576~extracellular region; GOTERM_BP_5 GO:0019220~regulation of phosphate metabolic process; GOTERM_BP_5 GO:0051174~regulation of phosphorus metabolic process; GOTERM_BP_4 GO:0051174~regulation of phosphorus metabolic process; GOTERM_BP_4 GO:0007399~nervous system development; GOTERM_CC_2 GO:0005615~extracellular space; GOTERM_CC_3 GO:0005615~extracellular space; GOTERM_BP_1 GO:0032501~multicellular organismal process; GOTERM_CC_1 GO:0044421~extracellular region part; GOTERM_BP_2 GO:0007275~multicellular organismal development; GOTERM_CC_2 GO:0044421~extracellular region part; GOTERM_BP_1 GO:0032502~developmental process; GOTERM_BP_3 GO:0048731~system development; GOTERM_BP_2 GO:0048856~anatomical structure development; GOTERM_MF_2 GO:0005515~protein binding |
| 4945-6433 | GOTERM_CC_1 GO:0005576~extracellular region; GOTERM_CC_2 GO:0005615~extracellular space; GOTERM_CC_3 GO:0005615~extracellular space; GOTERM_CC_1 GO:0044421~extracellular region part; GOTERM_CC_2 GO:0044421~extracellular region part; GOTERM_CC_3 GO:0044459~plasma membrane part; GOTERM_CC_4 GO:0044459~plasma membrane part; GOTERM_CC_5 GO:0044459~plasma membrane part; GOTERM_CC_3 GO:0031988~membrane-bounded vesicle; GOTERM_CC_4 GO:0016023~cytoplasmic membrane-bounded vesicle; GOTERM_CC_5 GO:0016023~cytoplasmic membrane-bounded vesicle; GOTERM_CC_3 GO:0031410~cytoplasmic vesicle; GOTERM_CC_4 GO:0031410~cytoplasmic vesicle; GOTERM_CC_2 GO:0031982~vesicle; GOTERM_CC_5 GO:0031410~cytoplasmic vesicle; GOTERM_CC_5 GO:0005887~integral to plasma membrane; GOTERM_MF_2 GO:0005515~protein binding; GOTERM_CC_4 GO:0031226~intrinsic to plasma membrane; GOTERM_CC_5 GO:0031226~intrinsic to plasma membrane |
| 4945-6410 | GOTERM_BP_5 GO:0051347~positive regulation of transferase activity; GOTERM_CC_2 GO:0012505~endomembrane system; GOTERM_CC_3 GO:0012505~endomembrane system; GOTERM_BP_3 GO:0050790~regulation of catalytic activity; GOTERM_BP_4 GO:0043085~positive regulation of catalytic activity; GOTERM_BP_3 GO:0044093~positive regulation of molecular function |
| 5219-3853 | GOTERM_CC_1 GO:0005576~extracellular region; GOTERM_CC_4 GO:0005578~proteinaceous extracellular matrix; GOTERM_CC_2 GO:0044421~extracellular region part; GOTERM_CC_3 GO:0005578~proteinaceous extracellular matrix; GOTERM_CC_2 GO:0031012~extracellular matrix; GOTERM_CC_3 GO:0031012~extracellular matrix; GOTERM_CC_1 GO:0044421~extracellular region part; GOTERM_BP_1 GO:0022610~biological adhesion; GOTERM_BP_2 GO:0007155~cell adhesion; GOTERM_MF_4 GO:0005539~glycosaminoglycan binding; GOTERM_MF_2 GO:0001871~pattern binding; GOTERM_MF_3 GO:0030247~polysaccharide binding; GOTERM_MF_2 GO:0030246~carbohydrate binding; GOTERM_MF_5 GO:0004175~endopeptidase activity; GOTERM_MF_4 GO:0070011~peptidase activity, acting on L-amino acid peptides; GOTERM_BP_1 GO:0032501~multicellular organismal process; GOTERM_CC_2 GO:0005615~extracellular space; GOTERM_CC_3 GO:0005615~extracellular space; GOTERM_MF_3 GO:0008233~peptidase activity |
| 5219-5316 | GOTERM_CC_1 GO:0005576~extracellular region; GOTERM_CC_2 GO:0044421~extracellular region part; GOTERM_CC_1 GO:0044421~extracellular region part; GOTERM_MF_5 GO:0004175~endopeptidase activity; GOTERM_MF_4 GO:0070011~peptidase activity, acting on L-amino acid peptides; GOTERM_CC_2 GO:0005615~extracellular space; GOTERM_CC_3 GO:0005615~extracellular space; GOTERM_MF_3 GO:0008233~peptidase activity |
| 5219-6240 | GOTERM_CC_2 GO:0044421~extracellular region part; GOTERM_CC_1 GO:0044421~extracellular region part; GOTERM_CC_5 GO:0044433~cytoplasmic vesicle part; GOTERM_CC_4 GO:0044433~cytoplasmic vesicle part; GOTERM_CC_3 GO:0044433~cytoplasmic vesicle part; GOTERM_CC_3 GO:0031988~membrane-bounded vesicle; GOTERM_CC_2 GO:0031982~vesicle; GOTERM_CC_5 GO:0016023~cytoplasmic membrane-bounded vesicle; GOTERM_MF_2 GO:0005515~protein binding; GOTERM_CC_5 GO:0031410~cytoplasmic vesicle; GOTERM_CC_4 GO:0016023~cytoplasmic membrane-bounded vesicle |
| 5219-6320 | GOTERM_CC_4 GO:0005578~proteinaceous extracellular matrix; GOTERM_CC_2 GO:0044421~extracellular region part; GOTERM_CC_3 GO:0005578~proteinaceous extracellular matrix; GOTERM_CC_2 GO:0031012~extracellular matrix; GOTERM_CC_3 GO:0031012~extracellular matrix; GOTERM_CC_1 GO:0044421~extracellular region part; GOTERM_CC_4 GO:0044420~extracellular matrix part; GOTERM_CC_2 GO:0044420~extracellular matrix part; GOTERM_CC_3 GO:0044420~extracellular matrix part; GOTERM_BP_1 GO:0022610~biological adhesion; GOTERM_BP_2 GO:0007155~cell adhesion; GOTERM_BP_1 GO:0032501~multicellular organismal process; GOTERM_CC_5 GO:0005605~basal lamina; GOTERM_CC_4 GO:0005605~basal lamina; GOTERM_CC_3 GO:0005605~basal lamina; GOTERM_MF_2 GO:0005515~protein binding |
| 5219-4305 | GOTERM_CC_1 GO:0005576~extracellular region; GOTERM_BP_3 GO:0048731~system development; GOTERM_BP_2 GO:0048856~anatomical structure development; GOTERM_BP_1 GO:0022610~biological adhesion; GOTERM_BP_2 GO:0007155~cell adhesion; GOTERM_BP_2 GO:0007275~multicellular organismal development; GOTERM_BP_1 GO:0032502~developmental process; GOTERM_BP_4 GO:0048513~organ development; GOTERM_BP_3 GO:0048513~organ development; GOTERM_CC_5 GO:0030141~secretory granule; GOTERM_CC_5 GO:0044433~cytoplasmic vesicle part; GOTERM_CC_4 GO:0044433~cytoplasmic vesicle part; GOTERM_CC_3 GO:0044433~cytoplasmic vesicle part; GOTERM_BP_3 GO:0050793~regulation of developmental process; GOTERM_BP_2 GO:0050793~regulation of developmental process; GOTERM_BP_4 GO:0030155~regulation of cell adhesion; GOTERM_BP_3 GO:0030155~regulation of cell adhesion; GOTERM_MF_2 GO:0005515~protein binding |
| 5219-6433 | GOTERM_CC_1 GO:0005576~extracellular region; GOTERM_CC_4 GO:0005578~proteinaceous extracellular matrix; GOTERM_CC_2 GO:0044421~extracellular region part; GOTERM_CC_3 GO:0005578~proteinaceous extracellular matrix; GOTERM_CC_2 GO:0031012~extracellular matrix; GOTERM_CC_3 GO:0031012~extracellular matrix; GOTERM_CC_1 GO:0044421~extracellular region part; GOTERM_CC_5 GO:0030141~secretory granule; GOTERM_CC_2 GO:0005615~extracellular space; GOTERM_CC_3 GO:0005615~extracellular space; GOTERM_CC_3 GO:0031988~membrane-bounded vesicle; GOTERM_BP_3 GO:0009611~response to wounding; GOTERM_CC_2 GO:0031982~vesicle; GOTERM_CC_5 GO:0016023~cytoplasmic membrane-bounded vesicle; GOTERM_MF_2 GO:0005515~protein binding; GOTERM_BP_3 GO:0048519~negative regulation of biological process; GOTERM_CC_5 GO:0031410~cytoplasmic vesicle; GOTERM_BP_2 GO:0048519~negative regulation of biological process; GOTERM_CC_4 GO:0016023~cytoplasmic membrane-bounded vesicle |
| 5219-6454 |  |
| 5033-5331 | GOTERM_BP_2 GO:0048522~positive regulation of cellular process; GOTERM_BP_3 GO:0048522~positive regulation of cellular process; GOTERM_BP_4 GO:0048522~positive regulation of cellular process; GOTERM_BP_2 GO:0048518~positive regulation of biological process; GOTERM_BP_1 GO:0065007~biological regulation; GOTERM_BP_3 GO:0048518~positive regulation of biological process; GOTERM_MF_2 GO:0005515~protein binding |
| 5033-5982 | GOTERM_MF_1 GO:0030528~transcription regulator activity; GOTERM_BP_4 GO:0006350~transcription; GOTERM_BP_4 GO:0010556~regulation of macromolecule biosynthetic process; GOTERM_BP_5 GO:0010556~regulation of macromolecule biosynthetic process; GOTERM_MF_3 GO:0003677~DNA binding; GOTERM_BP_3 GO:0060255~regulation of macromolecule metabolic process; GOTERM_BP_3 GO:0080090~regulation of primary metabolic process; GOTERM_BP_4 GO:0060255~regulation of macromolecule metabolic process; GOTERM_BP_4 GO:0080090~regulation of primary metabolic process; GOTERM_BP_3 GO:0031323~regulation of cellular metabolic process; GOTERM_BP_2 GO:0019222~regulation of metabolic process; GOTERM_BP_4 GO:0031323~regulation of cellular metabolic process; GOTERM_BP_3 GO:0019222~regulation of metabolic process; GOTERM_MF_2 GO:0003700~transcription factor activity; GOTERM_MF_4 GO:0003700~transcription factor activity; GOTERM_BP_2 GO:0050794~regulation of cellular process; GOTERM_BP_2 GO:0048522~positive regulation of cellular process; GOTERM_BP_3 GO:0050794~regulation of cellular process; GOTERM_BP_3 GO:0048522~positive regulation of cellular process; GOTERM_BP_2 GO:0050789~regulation of biological process; GOTERM_BP_4 GO:0048522~positive regulation of cellular process; GOTERM_BP_2 GO:0048518~positive regulation of biological process; GOTERM_BP_1 GO:0065007~biological regulation; GOTERM_BP_3 GO:0048518~positive regulation of biological process; GOTERM_CC_1 GO:0043226~organelle; GOTERM_CC_2 GO:0043229~intracellular organelle; GOTERM_CC_3 GO:0043229~intracellular organelle; GOTERM_CC_4 GO:0043229~intracellular organelle; GOTERM_CC_5 GO:0043229~intracellular organelle; GOTERM_BP_3 GO:0010605~negative regulation of macromolecule metabolic process; GOTERM_BP_4 GO:0010605~negative regulation of macromolecule metabolic process; GOTERM_BP_2 GO:0009892~negative regulation of metabolic process; GOTERM_BP_3 GO:0009892~negative regulation of metabolic process; GOTERM_BP_4 GO:0009892~negative regulation of metabolic process; GOTERM_BP_5 GO:0010605~negative regulation of macromolecule metabolic process; GOTERM_BP_3 GO:0048513~organ development; GOTERM_BP_4 GO:0048513~organ development; GOTERM_BP_3 GO:0031324~negative regulation of cellular metabolic process; GOTERM_BP_4 GO:0031324~negative regulation of cellular metabolic process; GOTERM_BP_3 GO:0048731~system development; GOTERM_BP_1 GO:0032502~developmental process; GOTERM_BP_5 GO:0031324~negative regulation of cellular metabolic process; GOTERM_BP_2 GO:0048856~anatomical structure development; GOTERM_MF_2 GO:0005515~protein binding; GOTERM_BP_2 GO:0007275~multicellular organismal development |
| 5033-6171 | GOTERM_MF_1 GO:0030528~transcription regulator activity; GOTERM_BP_4 GO:0006350~transcription; GOTERM_BP_3 GO:0009059~macromolecule biosynthetic process; GOTERM_BP_3 GO:0051171~regulation of nitrogen compound metabolic process; GOTERM_BP_5 GO:0045449~regulation of transcription; GOTERM_BP_4 GO:0034645~cellular macromolecule biosynthetic process; GOTERM_BP_4 GO:0019219~regulation of nucleobase, nucleoside, nucleotide and nucleic acid metabolic process; GOTERM_BP_4 GO:0010556~regulation of macromolecule biosynthetic process; GOTERM_BP_4 GO:0051171~regulation of nitrogen compound metabolic process; GOTERM_BP_4 GO:0010468~regulation of gene expression; GOTERM_BP_3 GO:0009889~regulation of biosynthetic process; GOTERM_BP_3 GO:0010467~gene expression; GOTERM_BP_4 GO:0031326~regulation of cellular biosynthetic process; GOTERM_BP_4 GO:0009889~regulation of biosynthetic process; GOTERM_BP_5 GO:0019219~regulation of nucleobase, nucleoside, nucleotide and nucleic acid metabolic process; GOTERM_BP_5 GO:0010556~regulation of macromolecule biosynthetic process; GOTERM_BP_5 GO:0010468~regulation of gene expression; GOTERM_MF_3 GO:0003677~DNA binding; GOTERM_BP_5 GO:0031326~regulation of cellular biosynthetic process; GOTERM_BP_4 GO:0051252~regulation of RNA metabolic process; GOTERM_BP_3 GO:0060255~regulation of macromolecule metabolic process; GOTERM_BP_3 GO:0080090~regulation of primary metabolic process; GOTERM_BP_4 GO:0060255~regulation of macromolecule metabolic process; GOTERM_BP_5 GO:0006355~regulation of transcription, DNA-dependent; GOTERM_BP_4 GO:0080090~regulation of primary metabolic process; GOTERM_BP_3 GO:0006139~nucleobase, nucleoside, nucleotide and nucleic acid metabolic process; GOTERM_BP_2 GO:0009058~biosynthetic process; GOTERM_BP_5 GO:0051252~regulation of RNA metabolic process; GOTERM_BP_3 GO:0044249~cellular biosynthetic process; GOTERM_BP_3 GO:0031323~regulation of cellular metabolic process; GOTERM_BP_2 GO:0019222~regulation of metabolic process; GOTERM_BP_4 GO:0031323~regulation of cellular metabolic process; GOTERM_BP_3 GO:0019222~regulation of metabolic process; GOTERM_BP_2 GO:0006807~nitrogen compound metabolic process; GOTERM_MF_2 GO:0003700~transcription factor activity; GOTERM_BP_3 GO:0034641~cellular nitrogen compound metabolic process; GOTERM_MF_2 GO:0003676~nucleic acid binding; GOTERM_MF_4 GO:0003700~transcription factor activity; GOTERM_CC_4 GO:0005634~nucleus; GOTERM_CC_5 GO:0005634~nucleus; GOTERM_BP_3 GO:0044260~cellular macromolecule metabolic process; GOTERM_MF_3 GO:0008134~transcription factor binding; GOTERM_BP_2 GO:0043170~macromolecule metabolic process; GOTERM_BP_2 GO:0044237~cellular metabolic process; GOTERM_BP_2 GO:0050794~regulation of cellular process; GOTERM_BP_2 GO:0044238~primary metabolic process; GOTERM_BP_3 GO:0050794~regulation of cellular process; GOTERM_BP_2 GO:0050789~regulation of biological process; GOTERM_CC_3 GO:0043231~intracellular membrane-bounded organelle; GOTERM_CC_2 GO:0043227~membrane-bounded organelle; GOTERM_BP_1 GO:0065007~biological regulation; GOTERM_CC_4 GO:0043231~intracellular membrane-bounded organelle; GOTERM_BP_1 GO:0008152~metabolic process; GOTERM_CC_5 GO:0043231~intracellular membrane-bounded organelle; GOTERM_CC_1 GO:0043226~organelle; GOTERM_CC_2 GO:0043229~intracellular organelle; GOTERM_CC_3 GO:0043229~intracellular organelle; GOTERM_BP_4 GO:0051253~negative regulation of RNA metabolic process; GOTERM_CC_4 GO:0043229~intracellular organelle; GOTERM_BP_5 GO:0045892~negative regulation of transcription, DNA-dependent; GOTERM_BP_5 GO:0051253~negative regulation of RNA metabolic process; GOTERM_CC_5 GO:0043229~intracellular organelle; GOTERM_BP_3 GO:0010605~negative regulation of macromolecule metabolic process; GOTERM_BP_4 GO:0010605~negative regulation of macromolecule metabolic process; GOTERM_BP_2 GO:0009892~negative regulation of metabolic process; GOTERM_BP_3 GO:0009892~negative regulation of metabolic process; GOTERM_BP_4 GO:0009892~negative regulation of metabolic process; GOTERM_BP_5 GO:0010605~negative regulation of macromolecule metabolic process; GOTERM_BP_3 GO:0048513~organ development; GOTERM_BP_4 GO:0048513~organ development; GOTERM_BP_5 GO:0016481~negative regulation of transcription; GOTERM_BP_4 GO:0010629~negative regulation of gene expression; GOTERM_BP_3 GO:0051172~negative regulation of nitrogen compound metabolic process; GOTERM_BP_4 GO:0045934~negative regulation of nucleobase, nucleoside, nucleotide and nucleic acid metabolic process; GOTERM_BP_4 GO:0051172~negative regulation of nitrogen compound metabolic process; GOTERM_BP_5 GO:0010629~negative regulation of gene expression; GOTERM_BP_4 GO:0010558~negative regulation of macromolecule biosynthetic process; GOTERM_BP_5 GO:0045934~negative regulation of nucleobase, nucleoside, nucleotide and nucleic acid metabolic process; GOTERM_BP_5 GO:0051172~negative regulation of nitrogen compound metabolic process; GOTERM_BP_3 GO:0009890~negative regulation of biosynthetic process; GOTERM_BP_4 GO:0031327~negative regulation of cellular biosynthetic process; GOTERM_BP_4 GO:0009890~negative regulation of biosynthetic process; GOTERM_BP_5 GO:0010558~negative regulation of macromolecule biosynthetic process; GOTERM_BP_5 GO:0031327~negative regulation of cellular biosynthetic process; GOTERM_CC_2 GO:0044424~intracellular part; GOTERM_BP_5 GO:0009890~negative regulation of biosynthetic process; GOTERM_CC_3 GO:0044424~intracellular part; GOTERM_BP_2 GO:0048523~negative regulation of cellular process; GOTERM_BP_3 GO:0030154~cell differentiation; GOTERM_BP_2 GO:0048869~cellular developmental process; GOTERM_BP_3 GO:0048523~negative regulation of cellular process; GOTERM_BP_3 GO:0031324~negative regulation of cellular metabolic process; GOTERM_CC_2 GO:0005622~intracellular; GOTERM_CC_3 GO:0005622~intracellular; GOTERM_BP_4 GO:0048523~negative regulation of cellular process; GOTERM_BP_4 GO:0031324~negative regulation of cellular metabolic process; GOTERM_CC_4 GO:0044424~intracellular part; GOTERM_BP_3 GO:0048731~system development; GOTERM_BP_2 GO:0048519~negative regulation of biological process; GOTERM_BP_1 GO:0032502~developmental process; GOTERM_BP_5 GO:0031324~negative regulation of cellular metabolic process; GOTERM_BP_3 GO:0048519~negative regulation of biological process; GOTERM_BP_2 GO:0048856~anatomical structure development; GOTERM_MF_2 GO:0005515~protein binding; GOTERM_BP_2 GO:0007275~multicellular organismal development; GOTERM_MF_2 GO:0016564~transcription repressor activity |
| 5033-5975 | GOTERM_MF_4 GO:0003700~transcription factor activity; GOTERM_MF_4 GO:0043565~sequence-specific DNA binding; GOTERM_BP_3 GO:0048513~organ development; GOTERM_BP_4 GO:0048513~organ development; GOTERM_CC_2 GO:0044424~intracellular part; GOTERM_CC_3 GO:0044424~intracellular part; GOTERM_CC_2 GO:0005622~intracellular; GOTERM_CC_3 GO:0005622~intracellular; GOTERM_CC_4 GO:0044424~intracellular part; GOTERM_BP_3 GO:0048731~system development; GOTERM_BP_1 GO:0032502~developmental process; GOTERM_BP_2 GO:0048856~anatomical structure development; GOTERM_BP_2 GO:0007275~multicellular organismal development |
| 5033-6404 | GOTERM_MF_1 GO:0030528~transcription regulator activity; GOTERM_BP_4 GO:0006350~transcription; GOTERM_BP_3 GO:0009059~macromolecule biosynthetic process; GOTERM_BP_3 GO:0051171~regulation of nitrogen compound metabolic process; GOTERM_BP_5 GO:0045449~regulation of transcription; GOTERM_BP_4 GO:0034645~cellular macromolecule biosynthetic process; GOTERM_BP_4 GO:0019219~regulation of nucleobase, nucleoside, nucleotide and nucleic acid metabolic process; GOTERM_BP_4 GO:0010556~regulation of macromolecule biosynthetic process; GOTERM_BP_4 GO:0051171~regulation of nitrogen compound metabolic process; GOTERM_BP_4 GO:0010468~regulation of gene expression; GOTERM_BP_3 GO:0009889~regulation of biosynthetic process; GOTERM_BP_3 GO:0010467~gene expression; GOTERM_BP_4 GO:0031326~regulation of cellular biosynthetic process; GOTERM_BP_4 GO:0009889~regulation of biosynthetic process; GOTERM_BP_5 GO:0019219~regulation of nucleobase, nucleoside, nucleotide and nucleic acid metabolic process; GOTERM_BP_5 GO:0010556~regulation of macromolecule biosynthetic process; GOTERM_BP_5 GO:0010468~regulation of gene expression; GOTERM_MF_3 GO:0003677~DNA binding; GOTERM_BP_4 GO:0051254~positive regulation of RNA metabolic process; GOTERM_BP_5 GO:0031326~regulation of cellular biosynthetic process; GOTERM_BP_4 GO:0051252~regulation of RNA metabolic process; GOTERM_BP_3 GO:0060255~regulation of macromolecule metabolic process; GOTERM_BP_3 GO:0080090~regulation of primary metabolic process; GOTERM_BP_5 GO:0045893~positive regulation of transcription, DNA-dependent; GOTERM_BP_4 GO:0060255~regulation of macromolecule metabolic process; GOTERM_BP_5 GO:0051254~positive regulation of RNA metabolic process; GOTERM_BP_5 GO:0006355~regulation of transcription, DNA-dependent; GOTERM_BP_4 GO:0080090~regulation of primary metabolic process; GOTERM_BP_3 GO:0006139~nucleobase, nucleoside, nucleotide and nucleic acid metabolic process; GOTERM_BP_2 GO:0009058~biosynthetic process; GOTERM_BP_5 GO:0051252~regulation of RNA metabolic process; GOTERM_BP_3 GO:0044249~cellular biosynthetic process; GOTERM_BP_3 GO:0031323~regulation of cellular metabolic process; GOTERM_BP_2 GO:0019222~regulation of metabolic process; GOTERM_BP_4 GO:0031323~regulation of cellular metabolic process; GOTERM_BP_4 GO:0010628~positive regulation of gene expression; GOTERM_BP_3 GO:0019222~regulation of metabolic process; GOTERM_BP_2 GO:0006807~nitrogen compound metabolic process; GOTERM_MF_2 GO:0003700~transcription factor activity; GOTERM_BP_5 GO:0045941~positive regulation of transcription; GOTERM_BP_3 GO:0034641~cellular nitrogen compound metabolic process; GOTERM_BP_5 GO:0010628~positive regulation of gene expression; GOTERM_BP_4 GO:0045935~positive regulation of nucleobase, nucleoside, nucleotide and nucleic acid metabolic process; GOTERM_BP_3 GO:0051173~positive regulation of nitrogen compound metabolic process; GOTERM_BP_4 GO:0051173~positive regulation of nitrogen compound metabolic process; GOTERM_BP_4 GO:0010557~positive regulation of macromolecule biosynthetic process; GOTERM_BP_5 GO:0045935~positive regulation of nucleobase, nucleoside, nucleotide and nucleic acid metabolic process; GOTERM_BP_3 GO:0009891~positive regulation of biosynthetic process; GOTERM_BP_4 GO:0031328~positive regulation of cellular biosynthetic process; GOTERM_BP_5 GO:0051173~positive regulation of nitrogen compound metabolic process; GOTERM_BP_4 GO:0009891~positive regulation of biosynthetic process; GOTERM_BP_5 GO:0010557~positive regulation of macromolecule biosynthetic process; GOTERM_BP_5 GO:0031328~positive regulation of cellular biosynthetic process; GOTERM_BP_5 GO:0009891~positive regulation of biosynthetic process; GOTERM_MF_4 GO:0003700~transcription factor activity; GOTERM_BP_3 GO:0010604~positive regulation of macromolecule metabolic process; GOTERM_BP_4 GO:0010604~positive regulation of macromolecule metabolic process; GOTERM_BP_3 GO:0031325~positive regulation of cellular metabolic process; GOTERM_BP_2 GO:0009893~positive regulation of metabolic process; GOTERM_MF_2 GO:0003702~RNA polymerase II transcription factor activity; GOTERM_BP_4 GO:0031325~positive regulation of cellular metabolic process; GOTERM_CC_4 GO:0005634~nucleus; GOTERM_CC_5 GO:0005634~nucleus; GOTERM_BP_3 GO:0044260~cellular macromolecule metabolic process; GOTERM_MF_3 GO:0008134~transcription factor binding; GOTERM_MF_2 GO:0003712~transcription cofactor activity; GOTERM_BP_2 GO:0043170~macromolecule metabolic process; GOTERM_MF_2 GO:0016563~transcription activator activity; GOTERM_MF_3 GO:0003713~transcription coactivator activity; GOTERM_MF_5 GO:0003713~transcription coactivator activity; GOTERM_MF_4 GO:0003712~transcription cofactor activity; GOTERM_BP_2 GO:0044237~cellular metabolic process; GOTERM_BP_2 GO:0050794~regulation of cellular process; GOTERM_BP_2 GO:0044238~primary metabolic process; GOTERM_BP_2 GO:0048522~positive regulation of cellular process; GOTERM_BP_3 GO:0050794~regulation of cellular process; GOTERM_BP_3 GO:0048522~positive regulation of cellular process; GOTERM_BP_2 GO:0050789~regulation of biological process; GOTERM_BP_4 GO:0048522~positive regulation of cellular process; GOTERM_CC_3 GO:0043231~intracellular membrane-bounded organelle; GOTERM_CC_2 GO:0043227~membrane-bounded organelle; GOTERM_BP_2 GO:0048518~positive regulation of biological process; GOTERM_BP_1 GO:0065007~biological regulation; GOTERM_BP_3 GO:0048518~positive regulation of biological process; GOTERM_CC_4 GO:0043231~intracellular membrane-bounded organelle; GOTERM_BP_1 GO:0008152~metabolic process; GOTERM_CC_5 GO:0043231~intracellular membrane-bounded organelle; GOTERM_CC_1 GO:0043226~organelle; GOTERM_CC_2 GO:0043229~intracellular organelle; GOTERM_CC_3 GO:0043229~intracellular organelle; GOTERM_BP_4 GO:0051253~negative regulation of RNA metabolic process; GOTERM_CC_4 GO:0043229~intracellular organelle; GOTERM_BP_5 GO:0045892~negative regulation of transcription, DNA-dependent; GOTERM_BP_5 GO:0051253~negative regulation of RNA metabolic process; GOTERM_CC_5 GO:0043229~intracellular organelle; GOTERM_BP_3 GO:0010605~negative regulation of macromolecule metabolic process; GOTERM_BP_4 GO:0010605~negative regulation of macromolecule metabolic process; GOTERM_BP_2 GO:0009892~negative regulation of metabolic process; GOTERM_BP_3 GO:0009892~negative regulation of metabolic process; GOTERM_BP_4 GO:0009892~negative regulation of metabolic process; GOTERM_BP_5 GO:0010605~negative regulation of macromolecule metabolic process; GOTERM_BP_5 GO:0016481~negative regulation of transcription; GOTERM_BP_4 GO:0010629~negative regulation of gene expression; GOTERM_BP_3 GO:0051172~negative regulation of nitrogen compound metabolic process; GOTERM_BP_4 GO:0045934~negative regulation of nucleobase, nucleoside, nucleotide and nucleic acid metabolic process; GOTERM_BP_4 GO:0051172~negative regulation of nitrogen compound metabolic process; GOTERM_BP_5 GO:0010629~negative regulation of gene expression; GOTERM_BP_4 GO:0010558~negative regulation of macromolecule biosynthetic process; GOTERM_BP_5 GO:0045934~negative regulation of nucleobase, nucleoside, nucleotide and nucleic acid metabolic process; GOTERM_BP_5 GO:0051172~negative regulation of nitrogen compound metabolic process; GOTERM_BP_3 GO:0009890~negative regulation of biosynthetic process; GOTERM_BP_4 GO:0031327~negative regulation of cellular biosynthetic process; GOTERM_BP_4 GO:0009890~negative regulation of biosynthetic process; GOTERM_BP_5 GO:0010558~negative regulation of macromolecule biosynthetic process; GOTERM_MF_5 GO:0035257~nuclear hormone receptor binding; GOTERM_BP_5 GO:0031327~negative regulation of cellular biosynthetic process; GOTERM_CC_2 GO:0044424~intracellular part; GOTERM_BP_5 GO:0009890~negative regulation of biosynthetic process; GOTERM_CC_3 GO:0044424~intracellular part; GOTERM_BP_2 GO:0048523~negative regulation of cellular process; GOTERM_MF_4 GO:0051427~hormone receptor binding; GOTERM_BP_3 GO:0048523~negative regulation of cellular process; GOTERM_BP_3 GO:0031324~negative regulation of cellular metabolic process; GOTERM_CC_2 GO:0005622~intracellular; GOTERM_CC_3 GO:0005622~intracellular; GOTERM_BP_4 GO:0048523~negative regulation of cellular process; GOTERM_BP_4 GO:0031324~negative regulation of cellular metabolic process; GOTERM_CC_4 GO:0044424~intracellular part; GOTERM_BP_2 GO:0048519~negative regulation of biological process; GOTERM_BP_5 GO:0031324~negative regulation of cellular metabolic process; GOTERM_MF_2 GO:0005515~protein binding; GOTERM_BP_1 GO:0009987~cellular process; GOTERM_MF_2 GO:0016564~transcription repressor activity |
| 5033-6116 | GOTERM_BP_5 GO:0045449~regulation of transcription; GOTERM_BP_3 GO:0010467~gene expression; GOTERM_BP_4 GO:0051254~positive regulation of RNA metabolic process; GOTERM_BP_3 GO:0060255~regulation of macromolecule metabolic process; GOTERM_BP_3 GO:0080090~regulation of primary metabolic process; GOTERM_BP_5 GO:0045893~positive regulation of transcription, DNA-dependent; GOTERM_BP_4 GO:0060255~regulation of macromolecule metabolic process; GOTERM_BP_5 GO:0051254~positive regulation of RNA metabolic process; GOTERM_BP_4 GO:0080090~regulation of primary metabolic process; GOTERM_BP_4 GO:0010628~positive regulation of gene expression; GOTERM_MF_2 GO:0003700~transcription factor activity; GOTERM_BP_5 GO:0045941~positive regulation of transcription; GOTERM_BP_5 GO:0010628~positive regulation of gene expression; GOTERM_BP_4 GO:0045935~positive regulation of nucleobase, nucleoside, nucleotide and nucleic acid metabolic process; GOTERM_BP_3 GO:0051173~positive regulation of nitrogen compound metabolic process; GOTERM_BP_4 GO:0051173~positive regulation of nitrogen compound metabolic process; GOTERM_BP_4 GO:0010557~positive regulation of macromolecule biosynthetic process; GOTERM_BP_5 GO:0045935~positive regulation of nucleobase, nucleoside, nucleotide and nucleic acid metabolic process; GOTERM_BP_3 GO:0009891~positive regulation of biosynthetic process; GOTERM_BP_4 GO:0031328~positive regulation of cellular biosynthetic process; GOTERM_BP_5 GO:0051173~positive regulation of nitrogen compound metabolic process; GOTERM_BP_4 GO:0009891~positive regulation of biosynthetic process; GOTERM_BP_5 GO:0010557~positive regulation of macromolecule biosynthetic process; GOTERM_BP_5 GO:0031328~positive regulation of cellular biosynthetic process; GOTERM_BP_5 GO:0009891~positive regulation of biosynthetic process; GOTERM_MF_4 GO:0043565~sequence-specific DNA binding; GOTERM_BP_3 GO:0044260~cellular macromolecule metabolic process; GOTERM_BP_4 GO:0007399~nervous system development; GOTERM_MF_2 GO:0005515~protein binding; GOTERM_BP_5 GO:0048699~generation of neurons; GOTERM_BP_4 GO:0022008~neurogenesis; GOTERM_BP_5 GO:0022008~neurogenesis |
| 5033-6410 | GOTERM_CC_1 GO:0043226~organelle; GOTERM_CC_2 GO:0043229~intracellular organelle; GOTERM_CC_3 GO:0043229~intracellular organelle |
| 3853-4393 | GOTERM_MF_2 GO:0001871~pattern binding; GOTERM_BP_5 GO:0006508~proteolysis; GOTERM_MF_4 GO:0005539~glycosaminoglycan binding; GOTERM_MF_3 GO:0008233~peptidase activity; GOTERM_MF_3 GO:0030247~polysaccharide binding; GOTERM_MF_2 GO:0030246~carbohydrate binding; GOTERM_MF_5 GO:0005509~calcium ion binding; GOTERM_BP_1 GO:0022610~biological adhesion; GOTERM_BP_2 GO:0007155~cell adhesion; GOTERM_MF_3 GO:0005529~sugar binding |
| 3853-3895 | GOTERM_CC_1 GO:0044421~extracellular region part; GOTERM_CC_2 GO:0044421~extracellular region part; GOTERM_CC_1 GO:0005576~extracellular region; GOTERM_MF_5 GO:0004175~endopeptidase activity; GOTERM_MF_4 GO:0070011~peptidase activity, acting on L-amino acid peptides; GOTERM_MF_2 GO:0001871~pattern binding; GOTERM_BP_5 GO:0006508~proteolysis; GOTERM_MF_4 GO:0005539~glycosaminoglycan binding; GOTERM_MF_3 GO:0008233~peptidase activity; GOTERM_MF_3 GO:0030247~polysaccharide binding; GOTERM_CC_2 GO:0005615~extracellular space; GOTERM_CC_3 GO:0005615~extracellular space; GOTERM_BP_1 GO:0022610~biological adhesion; GOTERM_BP_2 GO:0007155~cell adhesion; GOTERM_BP_1 GO:0032501~multicellular organismal process |
| 3853-4878 | GOTERM_CC_3 GO:0005578~proteinaceous extracellular matrix; GOTERM_CC_4 GO:0005578~proteinaceous extracellular matrix; GOTERM_CC_2 GO:0031012~extracellular matrix; GOTERM_CC_3 GO:0031012~extracellular matrix; GOTERM_CC_1 GO:0044421~extracellular region part; GOTERM_CC_2 GO:0044421~extracellular region part; GOTERM_CC_1 GO:0005576~extracellular region; GOTERM_BP_5 GO:0030574~collagen catabolic process; GOTERM_BP_4 GO:0030574~collagen catabolic process; GOTERM_BP_3 GO:0044243~multicellular organismal catabolic process; GOTERM_BP_4 GO:0032963~collagen metabolic process; GOTERM_BP_3 GO:0044259~multicellular organismal macromolecule metabolic process; GOTERM_BP_2 GO:0044236~multicellular organismal metabolic process; GOTERM_MF_5 GO:0004175~endopeptidase activity; GOTERM_MF_4 GO:0070011~peptidase activity, acting on L-amino acid peptides; GOTERM_MF_2 GO:0001871~pattern binding; GOTERM_BP_5 GO:0006508~proteolysis; GOTERM_MF_4 GO:0005539~glycosaminoglycan binding; GOTERM_MF_3 GO:0008233~peptidase activity; GOTERM_MF_3 GO:0030247~polysaccharide binding; GOTERM_MF_2 GO:0030246~carbohydrate binding; GOTERM_MF_5 GO:0005509~calcium ion binding; GOTERM_CC_2 GO:0005615~extracellular space; GOTERM_CC_3 GO:0005615~extracellular space; GOTERM_BP_1 GO:0032501~multicellular organismal process; GOTERM_MF_2 GO:0016787~hydrolase activity |
| 5331-4393 | GOTERM_CC_2 GO:0009986~cell surface; GOTERM_CC_3 GO:0009986~cell surface; GOTERM_BP_4 GO:0043085~positive regulation of catalytic activity; GOTERM_BP_3 GO:0044093~positive regulation of molecular function; GOTERM_MF_3 GO:0008233~peptidase activity; GOTERM_MF_2 GO:0005515~protein binding; GOTERM_BP_3 GO:0050790~regulation of catalytic activity |
| 5331-4842 |  |
| 5331-2437 |  |
| 5331-4878 | GOTERM_BP_2 GO:0048583~regulation of response to stimulus; GOTERM_BP_3 GO:0048583~regulation of response to stimulus; GOTERM_CC_1 GO:0005576~extracellular region; GOTERM_BP_2 GO:0065008~regulation of biological quality; GOTERM_CC_2 GO:0005615~extracellular space; GOTERM_CC_3 GO:0005615~extracellular space; GOTERM_MF_5 GO:0004175~endopeptidase activity; GOTERM_BP_1 GO:0065007~biological regulation; GOTERM_MF_3 GO:0008233~peptidase activity; GOTERM_MF_4 GO:0070011~peptidase activity, acting on L-amino acid peptides; GOTERM_CC_1 GO:0044421~extracellular region part; GOTERM_CC_2 GO:0044421~extracellular region part |
| 5331-6248 | GOTERM_CC_1 GO:0005576~extracellular region; GOTERM_CC_2 GO:0005615~extracellular space; GOTERM_CC_3 GO:0005615~extracellular space; GOTERM_MF_2 GO:0005515~protein binding; GOTERM_CC_1 GO:0044421~extracellular region part; GOTERM_CC_2 GO:0044421~extracellular region part; GOTERM_BP_3 GO:0042127~regulation of cell proliferation; GOTERM_BP_4 GO:0042127~regulation of cell proliferation |
| 5331-6454 |  |
| 5414-5978 | GOTERM_BP_5 GO:0006260~DNA replication; GOTERM_CC_4 GO:0005634~nucleus; GOTERM_BP_2 GO:0006807~nitrogen compound metabolic process; GOTERM_BP_3 GO:0034641~cellular nitrogen compound metabolic process; GOTERM_CC_5 GO:0005634~nucleus; GOTERM_CC_3 GO:0043231~intracellular membrane-bounded organelle; GOTERM_CC_2 GO:0043227~membrane-bounded organelle; GOTERM_CC_4 GO:0043231~intracellular membrane-bounded organelle; GOTERM_CC_5 GO:0043231~intracellular membrane-bounded organelle; GOTERM_CC_1 GO:0043226~organelle; GOTERM_CC_2 GO:0043229~intracellular organelle; GOTERM_CC_3 GO:0043229~intracellular organelle; GOTERM_CC_4 GO:0043229~intracellular organelle; GOTERM_CC_5 GO:0043229~intracellular organelle; GOTERM_CC_2 GO:0044424~intracellular part; GOTERM_CC_3 GO:0044424~intracellular part; GOTERM_CC_2 GO:0005622~intracellular; GOTERM_CC_3 GO:0005622~intracellular; GOTERM_CC_4 GO:0044424~intracellular part |
| 5414-5115 | GOTERM_CC_3 GO:0044428~nuclear part; GOTERM_CC_4 GO:0044428~nuclear part; GOTERM_CC_5 GO:0044428~nuclear part; GOTERM_CC_4 GO:0005654~nucleoplasm; GOTERM_CC_5 GO:0005654~nucleoplasm; GOTERM_MF_3 GO:0003677~DNA binding; GOTERM_BP_3 GO:0006139~nucleobase, nucleoside, nucleotide and nucleic acid metabolic process; GOTERM_CC_4 GO:0005634~nucleus; GOTERM_BP_2 GO:0006807~nitrogen compound metabolic process; GOTERM_BP_3 GO:0034641~cellular nitrogen compound metabolic process; GOTERM_CC_5 GO:0005634~nucleus; GOTERM_CC_4 GO:0031981~nuclear lumen; GOTERM_MF_2 GO:0003676~nucleic acid binding; GOTERM_CC_5 GO:0031981~nuclear lumen; GOTERM_CC_3 GO:0070013~intracellular organelle lumen; GOTERM_CC_1 GO:0031974~membrane-enclosed lumen; GOTERM_CC_2 GO:0043233~organelle lumen; GOTERM_CC_3 GO:0043233~organelle lumen; GOTERM_CC_4 GO:0070013~intracellular organelle lumen; GOTERM_CC_5 GO:0070013~intracellular organelle lumen; GOTERM_BP_3 GO:0044260~cellular macromolecule metabolic process; GOTERM_BP_2 GO:0043170~macromolecule metabolic process; GOTERM_CC_3 GO:0043231~intracellular membrane-bounded organelle; GOTERM_CC_2 GO:0043227~membrane-bounded organelle; GOTERM_CC_4 GO:0043231~intracellular membrane-bounded organelle; GOTERM_CC_5 GO:0043231~intracellular membrane-bounded organelle; GOTERM_CC_1 GO:0043226~organelle; GOTERM_BP_2 GO:0044237~cellular metabolic process; GOTERM_CC_2 GO:0043229~intracellular organelle; GOTERM_CC_3 GO:0043229~intracellular organelle; GOTERM_BP_2 GO:0044238~primary metabolic process; GOTERM_CC_4 GO:0043229~intracellular organelle; GOTERM_CC_5 GO:0043229~intracellular organelle; GOTERM_BP_1 GO:0008152~metabolic process; GOTERM_CC_4 GO:0044451~nucleoplasm part; GOTERM_CC_5 GO:0044451~nucleoplasm part; GOTERM_BP_1 GO:0009987~cellular process; GOTERM_BP_4 GO:0045935~positive regulation of nucleobase, nucleoside, nucleotide and nucleic acid metabolic process; GOTERM_BP_3 GO:0051173~positive regulation of nitrogen compound metabolic process; GOTERM_BP_4 GO:0051173~positive regulation of nitrogen compound metabolic process; GOTERM_BP_5 GO:0045935~positive regulation of nucleobase, nucleoside, nucleotide and nucleic acid metabolic process; GOTERM_BP_5 GO:0051173~positive regulation of nitrogen compound metabolic process |
| 5414-5699 | GOTERM_BP_2 GO:0006950~response to stress; GOTERM_BP_1 GO:0050896~response to stimulus; GOTERM_BP_1 GO:0002376~immune system process; GOTERM_MF_2 GO:0005515~protein binding |
| 5414-6240 | GOTERM_BP_3 GO:0042592~homeostatic process; GOTERM_BP_2 GO:0065008~regulation of biological quality; GOTERM_MF_2 GO:0005515~protein binding |
| 5414-5975 | GOTERM_CC_2 GO:0044424~intracellular part; GOTERM_CC_3 GO:0044424~intracellular part; GOTERM_CC_2 GO:0005622~intracellular; GOTERM_CC_3 GO:0005622~intracellular; GOTERM_CC_4 GO:0044424~intracellular part; GOTERM_MF_3 GO:0046983~protein dimerization activity |
| 4393-4944 | GOTERM_CC_2 GO:0009986~cell surface; GOTERM_CC_3 GO:0009986~cell surface; GOTERM_CC_3 GO:0043235~receptor complex; GOTERM_BP_3 GO:0032101~regulation of response to external stimulus; GOTERM_BP_4 GO:0032101~regulation of response to external stimulus; GOTERM_BP_2 GO:0009605~response to external stimulus; GOTERM_BP_3 GO:0009611~response to wounding |
| 4393-5982 | GOTERM_MF_2 GO:0005515~protein binding; GOTERM_BP_2 GO:0009056~catabolic process |
| 4393-3895 | GOTERM_BP_1 GO:0022610~biological adhesion; GOTERM_BP_2 GO:0007155~cell adhesion; GOTERM_CC_3 GO:0005886~plasma membrane; GOTERM_CC_4 GO:0005886~plasma membrane; GOTERM_CC_5 GO:0031410~cytoplasmic vesicle; GOTERM_CC_3 GO:0031410~cytoplasmic vesicle; GOTERM_CC_4 GO:0031410~cytoplasmic vesicle; GOTERM_CC_2 GO:0031982~vesicle; GOTERM_MF_4 GO:0005539~glycosaminoglycan binding; GOTERM_CC_5 GO:0016023~cytoplasmic membrane-bounded vesicle; GOTERM_CC_3 GO:0031988~membrane-bounded vesicle; GOTERM_CC_4 GO:0016023~cytoplasmic membrane-bounded vesicle; GOTERM_MF_3 GO:0030247~polysaccharide binding; GOTERM_MF_2 GO:0001871~pattern binding; GOTERM_CC_5 GO:0030141~secretory granule; GOTERM_CC_3 GO:0044433~cytoplasmic vesicle part; GOTERM_CC_5 GO:0044433~cytoplasmic vesicle part; GOTERM_CC_4 GO:0044433~cytoplasmic vesicle part; GOTERM_BP_5 GO:0006508~proteolysis; GOTERM_MF_3 GO:0008233~peptidase activity; GOTERM_CC_5 GO:0030667~secretory granule membrane; GOTERM_BP_2 GO:0009605~response to external stimulus; GOTERM_BP_3 GO:0009611~response to wounding; GOTERM_MF_3 GO:0001948~glycoprotein binding |
| 4393-3847 | GOTERM_CC_5 GO:0005887~integral to plasma membrane; GOTERM_CC_5 GO:0031226~intrinsic to plasma membrane; GOTERM_CC_4 GO:0031226~intrinsic to plasma membrane; GOTERM_CC_2 GO:0009986~cell surface; GOTERM_CC_3 GO:0009986~cell surface; GOTERM_BP_1 GO:0022610~biological adhesion; GOTERM_BP_2 GO:0007155~cell adhesion; GOTERM_CC_5 GO:0044459~plasma membrane part; GOTERM_CC_3 GO:0044459~plasma membrane part; GOTERM_CC_4 GO:0044459~plasma membrane part; GOTERM_BP_3 GO:0016337~cell-cell adhesion; GOTERM_CC_3 GO:0005886~plasma membrane; GOTERM_CC_4 GO:0005886~plasma membrane; GOTERM_BP_4 GO:0007159~leukocyte adhesion; GOTERM_CC_5 GO:0031224~intrinsic to membrane; GOTERM_CC_3 GO:0009897~external side of plasma membrane; GOTERM_CC_5 GO:0009897~external side of plasma membrane; GOTERM_CC_3 GO:0031224~intrinsic to membrane; GOTERM_CC_4 GO:0009897~external side of plasma membrane; GOTERM_CC_4 GO:0031224~intrinsic to membrane; GOTERM_CC_5 GO:0031410~cytoplasmic vesicle; GOTERM_CC_3 GO:0031410~cytoplasmic vesicle; GOTERM_CC_4 GO:0031410~cytoplasmic vesicle; GOTERM_CC_2 GO:0031982~vesicle; GOTERM_CC_5 GO:0016021~integral to membrane; GOTERM_CC_4 GO:0016021~integral to membrane; GOTERM_CC_5 GO:0016023~cytoplasmic membrane-bounded vesicle; GOTERM_CC_3 GO:0031988~membrane-bounded vesicle; GOTERM_CC_4 GO:0016023~cytoplasmic membrane-bounded vesicle; GOTERM_CC_2 GO:0044425~membrane part; GOTERM_CC_3 GO:0044425~membrane part; GOTERM_CC_4 GO:0044425~membrane part; GOTERM_CC_5 GO:0030141~secretory granule; GOTERM_CC_3 GO:0044433~cytoplasmic vesicle part; GOTERM_CC_5 GO:0044433~cytoplasmic vesicle part; GOTERM_CC_4 GO:0044433~cytoplasmic vesicle part; GOTERM_CC_2 GO:0016020~membrane; GOTERM_CC_3 GO:0016020~membrane; GOTERM_BP_1 GO:0040011~locomotion; GOTERM_BP_4 GO:0007157~heterophilic cell adhesion; GOTERM_BP_3 GO:0009611~response to wounding |
| 4393-5822 | GOTERM_CC_5 GO:0031226~intrinsic to plasma membrane; GOTERM_CC_4 GO:0031226~intrinsic to plasma membrane; GOTERM_BP_1 GO:0022610~biological adhesion; GOTERM_BP_2 GO:0007155~cell adhesion; GOTERM_CC_5 GO:0044459~plasma membrane part; GOTERM_CC_3 GO:0044459~plasma membrane part; GOTERM_CC_4 GO:0044459~plasma membrane part; GOTERM_BP_3 GO:0016337~cell-cell adhesion; GOTERM_CC_3 GO:0005886~plasma membrane; GOTERM_CC_4 GO:0005886~plasma membrane; GOTERM_BP_4 GO:0007159~leukocyte adhesion; GOTERM_BP_5 GO:0007166~cell surface receptor linked signal transduction; GOTERM_BP_4 GO:0007166~cell surface receptor linked signal transduction; GOTERM_CC_5 GO:0005624~membrane fraction; GOTERM_CC_3 GO:0005626~insoluble fraction; GOTERM_CC_4 GO:0005624~membrane fraction; GOTERM_BP_3 GO:0032101~regulation of response to external stimulus; GOTERM_BP_4 GO:0032101~regulation of response to external stimulus; GOTERM_CC_4 GO:0005626~insoluble fraction; GOTERM_BP_4 GO:0007157~heterophilic cell adhesion; GOTERM_CC_2 GO:0000267~cell fraction; GOTERM_CC_3 GO:0000267~cell fraction |
| 4393-4305 | GOTERM_CC_5 GO:0005887~integral to plasma membrane; GOTERM_CC_5 GO:0031226~intrinsic to plasma membrane; GOTERM_CC_4 GO:0031226~intrinsic to plasma membrane; GOTERM_CC_5 GO:0008305~integrin complex; GOTERM_CC_4 GO:0008305~integrin complex; GOTERM_CC_2 GO:0009986~cell surface; GOTERM_CC_3 GO:0009986~cell surface; GOTERM_BP_1 GO:0022610~biological adhesion; GOTERM_BP_2 GO:0007155~cell adhesion; GOTERM_CC_5 GO:0044459~plasma membrane part; GOTERM_CC_3 GO:0044459~plasma membrane part; GOTERM_CC_4 GO:0044459~plasma membrane part; GOTERM_BP_5 GO:0007229~integrin-mediated signaling pathway; GOTERM_CC_3 GO:0005886~plasma membrane; GOTERM_CC_4 GO:0005886~plasma membrane; GOTERM_CC_3 GO:0043235~receptor complex; GOTERM_CC_5 GO:0031224~intrinsic to membrane; GOTERM_CC_3 GO:0009897~external side of plasma membrane; GOTERM_CC_5 GO:0009897~external side of plasma membrane; GOTERM_CC_3 GO:0031224~intrinsic to membrane; GOTERM_CC_4 GO:0009897~external side of plasma membrane; GOTERM_CC_4 GO:0031224~intrinsic to membrane; GOTERM_CC_5 GO:0016021~integral to membrane; GOTERM_CC_4 GO:0016021~integral to membrane; GOTERM_CC_2 GO:0044425~membrane part; GOTERM_CC_3 GO:0044425~membrane part; GOTERM_CC_4 GO:0044425~membrane part; GOTERM_CC_5 GO:0030141~secretory granule; GOTERM_CC_3 GO:0044433~cytoplasmic vesicle part; GOTERM_CC_5 GO:0044433~cytoplasmic vesicle part; GOTERM_CC_4 GO:0044433~cytoplasmic vesicle part; GOTERM_BP_5 GO:0007166~cell surface receptor linked signal transduction; GOTERM_CC_2 GO:0016020~membrane; GOTERM_CC_3 GO:0016020~membrane; GOTERM_BP_4 GO:0007166~cell surface receptor linked signal transduction; GOTERM_MF_2 GO:0005515~protein binding; GOTERM_BP_1 GO:0040011~locomotion; GOTERM_MF_5 GO:0005509~calcium ion binding |
| 4944-5982 | GOTERM_MF_5 GO:0005138~interleukin-6 receptor binding; GOTERM_BP_2 GO:0048522~positive regulation of cellular process; GOTERM_BP_3 GO:0048522~positive regulation of cellular process; GOTERM_BP_4 GO:0048522~positive regulation of cellular process; GOTERM_BP_2 GO:0048518~positive regulation of biological process; GOTERM_BP_3 GO:0048518~positive regulation of biological process; GOTERM_BP_3 GO:0060255~regulation of macromolecule metabolic process; GOTERM_BP_3 GO:0080090~regulation of primary metabolic process; GOTERM_BP_4 GO:0060255~regulation of macromolecule metabolic process |
| 4944-3895 | GOTERM_BP_2 GO:0009605~response to external stimulus; GOTERM_BP_3 GO:0009611~response to wounding; GOTERM_CC_1 GO:0005576~extracellular region; GOTERM_BP_2 GO:0050817~coagulation; GOTERM_BP_3 GO:0007596~blood coagulation; GOTERM_CC_2 GO:0005615~extracellular space; GOTERM_CC_3 GO:0005615~extracellular space; GOTERM_BP_4 GO:0007596~blood coagulation; GOTERM_BP_3 GO:0007599~hemostasis; GOTERM_BP_4 GO:0007599~hemostasis; GOTERM_BP_5 GO:0007596~blood coagulation; GOTERM_BP_2 GO:0050878~regulation of body fluid levels; GOTERM_BP_3 GO:0050878~regulation of body fluid levels; GOTERM_CC_1 GO:0044421~extracellular region part; GOTERM_CC_2 GO:0044421~extracellular region part; GOTERM_BP_4 GO:0042060~wound healing; GOTERM_BP_1 GO:0050896~response to stimulus; GOTERM_BP_2 GO:0065008~regulation of biological quality; GOTERM_BP_2 GO:0006950~response to stress; GOTERM_BP_4 GO:0030195~negative regulation of blood coagulation; GOTERM_BP_5 GO:0030195~negative regulation of blood coagulation; GOTERM_BP_3 GO:0050819~negative regulation of coagulation; GOTERM_BP_4 GO:0050819~negative regulation of coagulation; GOTERM_BP_5 GO:0050819~negative regulation of coagulation; GOTERM_BP_4 GO:0030193~regulation of blood coagulation; GOTERM_BP_5 GO:0030193~regulation of blood coagulation; GOTERM_MF_3 GO:0005102~receptor binding |
| 4944-5316 | GOTERM_CC_1 GO:0005576~extracellular region; GOTERM_CC_2 GO:0005615~extracellular space; GOTERM_CC_3 GO:0005615~extracellular space; GOTERM_CC_1 GO:0044421~extracellular region part; GOTERM_CC_2 GO:0044421~extracellular region part; GOTERM_BP_4 GO:0050727~regulation of inflammatory response; GOTERM_BP_5 GO:0050727~regulation of inflammatory response |
| 4944-4958 | GOTERM_BP_3 GO:0032101~regulation of response to external stimulus; GOTERM_BP_4 GO:0032101~regulation of response to external stimulus; GOTERM_BP_2 GO:0048583~regulation of response to stimulus; GOTERM_BP_3 GO:0048583~regulation of response to stimulus; GOTERM_CC_1 GO:0005576~extracellular region; GOTERM_BP_4 GO:0051246~regulation of protein metabolic process; GOTERM_BP_5 GO:0051246~regulation of protein metabolic process; GOTERM_CC_2 GO:0005615~extracellular space; GOTERM_CC_3 GO:0005615~extracellular space; GOTERM_CC_1 GO:0044421~extracellular region part; GOTERM_CC_2 GO:0044421~extracellular region part; GOTERM_BP_2 GO:0051239~regulation of multicellular organismal process; GOTERM_BP_3 GO:0051239~regulation of multicellular organismal process; GOTERM_BP_4 GO:0032270~positive regulation of cellular protein metabolic process; GOTERM_BP_4 GO:0051247~positive regulation of protein metabolic process; GOTERM_BP_5 GO:0032270~positive regulation of cellular protein metabolic process; GOTERM_BP_5 GO:0051247~positive regulation of protein metabolic process; GOTERM_BP_3 GO:0080134~regulation of response to stress; GOTERM_BP_4 GO:0080134~regulation of response to stress; GOTERM_BP_4 GO:0032268~regulation of cellular protein metabolic process; GOTERM_BP_2 GO:0048519~negative regulation of biological process; GOTERM_BP_2 GO:0048522~positive regulation of cellular process; GOTERM_BP_5 GO:0032268~regulation of cellular protein metabolic process; GOTERM_BP_3 GO:0048519~negative regulation of biological process; GOTERM_BP_3 GO:0048522~positive regulation of cellular process; GOTERM_BP_4 GO:0048522~positive regulation of cellular process; GOTERM_BP_2 GO:0048518~positive regulation of biological process; GOTERM_BP_3 GO:0048518~positive regulation of biological process; GOTERM_MF_5 GO:0004896~cytokine receptor activity; GOTERM_MF_4 GO:0004896~cytokine receptor activity; GOTERM_BP_3 GO:0032103~positive regulation of response to external stimulus; GOTERM_BP_4 GO:0032103~positive regulation of response to external stimulus; GOTERM_BP_5 GO:0032103~positive regulation of response to external stimulus; GOTERM_BP_3 GO:0010604~positive regulation of macromolecule metabolic process; GOTERM_BP_5 GO:0019221~cytokine-mediated signaling pathway; GOTERM_BP_4 GO:0010604~positive regulation of macromolecule metabolic process; GOTERM_BP_3 GO:0031325~positive regulation of cellular metabolic process; GOTERM_BP_2 GO:0009893~positive regulation of metabolic process; GOTERM_BP_4 GO:0031325~positive regulation of cellular metabolic process; GOTERM_MF_3 GO:0005102~receptor binding; GOTERM_BP_3 GO:0009893~positive regulation of metabolic process; GOTERM_BP_5 GO:0010604~positive regulation of macromolecule metabolic process; GOTERM_BP_4 GO:0009893~positive regulation of metabolic process; GOTERM_BP_5 GO:0031325~positive regulation of cellular metabolic process; GOTERM_BP_4 GO:0010562~positive regulation of phosphorus metabolic process; GOTERM_BP_5 GO:0010562~positive regulation of phosphorus metabolic process; GOTERM_BP_5 GO:0045937~positive regulation of phosphate metabolic process; GOTERM_MF_3 GO:0019955~cytokine binding; GOTERM_BP_4 GO:0031347~regulation of defense response; GOTERM_BP_5 GO:0031347~regulation of defense response; GOTERM_BP_3 GO:0060255~regulation of macromolecule metabolic process; GOTERM_BP_3 GO:0080090~regulation of primary metabolic process; GOTERM_BP_4 GO:0060255~regulation of macromolecule metabolic process |
| 5982-6171 | GOTERM_MF_2 GO:0005515~protein binding; GOTERM_BP_3 GO:0048513~organ development; GOTERM_BP_4 GO:0048513~organ development; GOTERM_BP_3 GO:0048731~system development; GOTERM_BP_2 GO:0048856~anatomical structure development; GOTERM_BP_2 GO:0007275~multicellular organismal development; GOTERM_MF_4 GO:0003700~transcription factor activity; GOTERM_MF_2 GO:0003700~transcription factor activity; GOTERM_CC_3 GO:0044428~nuclear part; GOTERM_BP_1 GO:0032502~developmental process; GOTERM_MF_3 GO:0003677~DNA binding; GOTERM_BP_2 GO:0019222~regulation of metabolic process; GOTERM_BP_3 GO:0019222~regulation of metabolic process; GOTERM_BP_2 GO:0050789~regulation of biological process; GOTERM_CC_1 GO:0043226~organelle; GOTERM_BP_3 GO:0050794~regulation of cellular process; GOTERM_BP_2 GO:0050794~regulation of cellular process; GOTERM_BP_3 GO:0031323~regulation of cellular metabolic process; GOTERM_BP_4 GO:0031323~regulation of cellular metabolic process; GOTERM_MF_4 GO:0017069~snRNA binding; GOTERM_BP_3 GO:0060255~regulation of macromolecule metabolic process; GOTERM_BP_1 GO:0065007~biological regulation; GOTERM_BP_3 GO:0080090~regulation of primary metabolic process; GOTERM_CC_2 GO:0043229~intracellular organelle; GOTERM_CC_3 GO:0043229~intracellular organelle; GOTERM_CC_4 GO:0043229~intracellular organelle; GOTERM_BP_4 GO:0060255~regulation of macromolecule metabolic process; GOTERM_BP_4 GO:0080090~regulation of primary metabolic process; GOTERM_BP_3 GO:0031324~negative regulation of cellular metabolic process; GOTERM_CC_5 GO:0043229~intracellular organelle; GOTERM_BP_4 GO:0006350~transcription; GOTERM_BP_3 GO:0010605~negative regulation of macromolecule metabolic process; GOTERM_BP_4 GO:0031324~negative regulation of cellular metabolic process; GOTERM_BP_5 GO:0031324~negative regulation of cellular metabolic process; GOTERM_BP_4 GO:0010605~negative regulation of macromolecule metabolic process; GOTERM_BP_5 GO:0010605~negative regulation of macromolecule metabolic process; GOTERM_BP_1 GO:0032501~multicellular organismal process; GOTERM_BP_2 GO:0009892~negative regulation of metabolic process; GOTERM_BP_3 GO:0009892~negative regulation of metabolic process; GOTERM_BP_4 GO:0010556~regulation of macromolecule biosynthetic process; GOTERM_BP_5 GO:0010556~regulation of macromolecule biosynthetic process; GOTERM_MF_1 GO:0030528~transcription regulator activity; GOTERM_BP_4 GO:0009892~negative regulation of metabolic process |
| 5982-5975 | GOTERM_BP_3 GO:0048513~organ development; GOTERM_BP_4 GO:0048513~organ development; GOTERM_BP_3 GO:0048731~system development; GOTERM_BP_2 GO:0048856~anatomical structure development; GOTERM_BP_2 GO:0007275~multicellular organismal development; GOTERM_MF_4 GO:0003700~transcription factor activity; GOTERM_BP_1 GO:0032502~developmental process; GOTERM_MF_1 GO:0005488~binding; GOTERM_BP_1 GO:0032501~multicellular organismal process |
| 5982-6404 | GOTERM_MF_2 GO:0005515~protein binding; GOTERM_CC_2 GO:0043234~protein complex; GOTERM_MF_4 GO:0003700~transcription factor activity; GOTERM_MF_2 GO:0003700~transcription factor activity; GOTERM_CC_3 GO:0044428~nuclear part; GOTERM_CC_4 GO:0044428~nuclear part; GOTERM_CC_5 GO:0044428~nuclear part; GOTERM_MF_3 GO:0003677~DNA binding; GOTERM_BP_2 GO:0019222~regulation of metabolic process; GOTERM_BP_3 GO:0019222~regulation of metabolic process; GOTERM_CC_1 GO:0032991~macromolecular complex; GOTERM_BP_2 GO:0050789~regulation of biological process; GOTERM_CC_4 GO:0005654~nucleoplasm; GOTERM_CC_1 GO:0043226~organelle; GOTERM_CC_4 GO:0031981~nuclear lumen; GOTERM_CC_3 GO:0070013~intracellular organelle lumen; GOTERM_CC_1 GO:0031974~membrane-enclosed lumen; GOTERM_CC_5 GO:0005654~nucleoplasm; GOTERM_CC_4 GO:0070013~intracellular organelle lumen; GOTERM_CC_5 GO:0031981~nuclear lumen; GOTERM_BP_3 GO:0050794~regulation of cellular process; GOTERM_BP_2 GO:0050794~regulation of cellular process; GOTERM_BP_3 GO:0031323~regulation of cellular metabolic process; GOTERM_CC_2 GO:0043233~organelle lumen; GOTERM_CC_3 GO:0043233~organelle lumen; GOTERM_CC_5 GO:0070013~intracellular organelle lumen; GOTERM_BP_4 GO:0031323~regulation of cellular metabolic process; GOTERM_BP_3 GO:0060255~regulation of macromolecule metabolic process; GOTERM_BP_1 GO:0065007~biological regulation; GOTERM_BP_3 GO:0080090~regulation of primary metabolic process; GOTERM_CC_2 GO:0043229~intracellular organelle; GOTERM_BP_2 GO:0048518~positive regulation of biological process; GOTERM_CC_3 GO:0043229~intracellular organelle; GOTERM_BP_3 GO:0048518~positive regulation of biological process; GOTERM_CC_4 GO:0043229~intracellular organelle; GOTERM_BP_4 GO:0060255~regulation of macromolecule metabolic process; GOTERM_BP_4 GO:0080090~regulation of primary metabolic process; GOTERM_BP_3 GO:0031324~negative regulation of cellular metabolic process; GOTERM_CC_5 GO:0043229~intracellular organelle; GOTERM_CC_4 GO:0044451~nucleoplasm part; GOTERM_BP_2 GO:0048522~positive regulation of cellular process; GOTERM_BP_4 GO:0006350~transcription; GOTERM_BP_3 GO:0010605~negative regulation of macromolecule metabolic process; GOTERM_BP_3 GO:0048522~positive regulation of cellular process; GOTERM_BP_4 GO:0031324~negative regulation of cellular metabolic process; GOTERM_CC_5 GO:0044451~nucleoplasm part; GOTERM_BP_5 GO:0031324~negative regulation of cellular metabolic process; GOTERM_BP_4 GO:0010605~negative regulation of macromolecule metabolic process; GOTERM_BP_5 GO:0010605~negative regulation of macromolecule metabolic process; GOTERM_BP_4 GO:0048522~positive regulation of cellular process; GOTERM_BP_2 GO:0009892~negative regulation of metabolic process; GOTERM_BP_3 GO:0009892~negative regulation of metabolic process; GOTERM_BP_4 GO:0010556~regulation of macromolecule biosynthetic process; GOTERM_BP_5 GO:0010556~regulation of macromolecule biosynthetic process; GOTERM_MF_1 GO:0030528~transcription regulator activity; GOTERM_BP_4 GO:0009892~negative regulation of metabolic process |
| 5978-5949 |  |
| 5978-6121 | GOTERM_CC_2 GO:0044424~intracellular part; GOTERM_CC_3 GO:0044424~intracellular part; GOTERM_CC_3 GO:0043231~intracellular membrane-bounded organelle; GOTERM_CC_2 GO:0043227~membrane-bounded organelle; GOTERM_CC_2 GO:0005622~intracellular; GOTERM_CC_3 GO:0005622~intracellular; GOTERM_CC_4 GO:0044424~intracellular part; GOTERM_CC_4 GO:0043231~intracellular membrane-bounded organelle; GOTERM_CC_5 GO:0043231~intracellular membrane-bounded organelle; GOTERM_CC_1 GO:0043226~organelle; GOTERM_CC_2 GO:0043229~intracellular organelle; GOTERM_CC_3 GO:0043229~intracellular organelle; GOTERM_CC_4 GO:0043229~intracellular organelle; GOTERM_CC_4 GO:0005634~nucleus; GOTERM_CC_5 GO:0043229~intracellular organelle; GOTERM_CC_5 GO:0005634~nucleus |
| 5978-6126 |  |
| 5978-5975 | GOTERM_CC_2 GO:0044424~intracellular part; GOTERM_CC_3 GO:0044424~intracellular part; GOTERM_CC_2 GO:0005622~intracellular; GOTERM_CC_3 GO:0005622~intracellular; GOTERM_CC_4 GO:0044424~intracellular part |
| 5978-6404 | GOTERM_CC_2 GO:0044424~intracellular part; GOTERM_CC_3 GO:0044424~intracellular part; GOTERM_CC_3 GO:0043231~intracellular membrane-bounded organelle; GOTERM_CC_2 GO:0043227~membrane-bounded organelle; GOTERM_CC_2 GO:0005622~intracellular; GOTERM_CC_3 GO:0005622~intracellular; GOTERM_CC_4 GO:0044424~intracellular part; GOTERM_CC_4 GO:0043231~intracellular membrane-bounded organelle; GOTERM_CC_5 GO:0043231~intracellular membrane-bounded organelle; GOTERM_CC_1 GO:0043226~organelle; GOTERM_CC_2 GO:0043229~intracellular organelle; GOTERM_CC_3 GO:0043229~intracellular organelle; GOTERM_CC_4 GO:0043229~intracellular organelle; GOTERM_BP_2 GO:0006807~nitrogen compound metabolic process; GOTERM_BP_3 GO:0034641~cellular nitrogen compound metabolic process; GOTERM_CC_4 GO:0005634~nucleus; GOTERM_CC_5 GO:0043229~intracellular organelle; GOTERM_CC_5 GO:0005634~nucleus |
| 5949-2437 | GOTERM_MF_1 GO:0005488~binding |
| 5949-6171 | GOTERM_MF_2 GO:0005515~protein binding |
| 5949-6126 | GOTERM_MF_2 GO:0005515~protein binding |
| 5949-5699 | GOTERM_CC_4 GO:0044459~plasma membrane part; GOTERM_CC_5 GO:0044459~plasma membrane part; GOTERM_CC_3 GO:0044459~plasma membrane part; GOTERM_CC_4 GO:0005886~plasma membrane; GOTERM_CC_3 GO:0005886~plasma membrane; GOTERM_MF_2 GO:0005515~protein binding; GOTERM_CC_5 GO:0005887~integral to plasma membrane; GOTERM_CC_4 GO:0031226~intrinsic to plasma membrane; GOTERM_MF_3 GO:0004872~receptor activity; GOTERM_CC_5 GO:0031226~intrinsic to plasma membrane; GOTERM_MF_3 GO:0019904~protein domain specific binding; GOTERM_CC_4 GO:0005624~membrane fraction; GOTERM_CC_5 GO:0005624~membrane fraction; GOTERM_CC_4 GO:0005626~insoluble fraction; GOTERM_CC_3 GO:0005626~insoluble fraction |
| 5949-5975 | GOTERM_MF_1 GO:0005488~binding |
| 5949-6248 | GOTERM_MF_2 GO:0005515~protein binding |
| 5949-5931 | GOTERM_CC_4 GO:0044459~plasma membrane part; GOTERM_CC_5 GO:0044459~plasma membrane part; GOTERM_CC_3 GO:0044459~plasma membrane part; GOTERM_CC_4 GO:0005886~plasma membrane; GOTERM_CC_3 GO:0005886~plasma membrane; GOTERM_CC_5 GO:0005887~integral to plasma membrane; GOTERM_CC_4 GO:0031226~intrinsic to plasma membrane; GOTERM_CC_2 GO:0016020~membrane; GOTERM_CC_3 GO:0016020~membrane; GOTERM_CC_5 GO:0031226~intrinsic to plasma membrane; GOTERM_BP_5 GO:0007268~synaptic transmission; GOTERM_BP_4 GO:0007268~synaptic transmission |
| 5949-6433 | GOTERM_CC_4 GO:0044459~plasma membrane part; GOTERM_CC_5 GO:0044459~plasma membrane part; GOTERM_CC_3 GO:0044459~plasma membrane part; GOTERM_MF_2 GO:0005515~protein binding; GOTERM_CC_5 GO:0005887~integral to plasma membrane; GOTERM_CC_4 GO:0031226~intrinsic to plasma membrane; GOTERM_CC_5 GO:0031226~intrinsic to plasma membrane; GOTERM_BP_5 GO:0015031~protein transport; GOTERM_BP_4 GO:0015031~protein transport; GOTERM_BP_4 GO:0045184~establishment of protein localization; GOTERM_BP_2 GO:0033036~macromolecule localization; GOTERM_MF_1 GO:0005488~binding |
| 4842-5975 | GOTERM_CC_3 GO:0005833~hemoglobin complex; GOTERM_CC_5 GO:0005833~hemoglobin complex; GOTERM_CC_4 GO:0005829~cytosol |
| 4842-5931 | GOTERM_CC_3 GO:0044459~plasma membrane part; GOTERM_CC_4 GO:0044459~plasma membrane part; GOTERM_CC_5 GO:0044459~plasma membrane part; GOTERM_MF_4 GO:0016709~oxidoreductase activity, acting on paired donors, with incorporation or reduction of molecular oxygen, NADH or NADPH as one donor, and incorporation of one atom of oxygen; GOTERM_CC_2 GO:0016020~membrane; GOTERM_CC_3 GO:0016020~membrane; GOTERM_CC_5 GO:0005887~integral to plasma membrane; GOTERM_CC_4 GO:0031226~intrinsic to plasma membrane; GOTERM_CC_5 GO:0031226~intrinsic to plasma membrane; GOTERM_CC_3 GO:0005886~plasma membrane; GOTERM_CC_4 GO:0005886~plasma membrane; GOTERM_BP_4 GO:0001666~response to hypoxia; GOTERM_BP_3 GO:0001666~response to hypoxia; GOTERM_BP_3 GO:0070482~response to oxygen levels |
| 6121-2437 | GOTERM_CC_4 GO:0005634~nucleus; GOTERM_CC_5 GO:0005634~nucleus; GOTERM_CC_2 GO:0044424~intracellular part; GOTERM_CC_3 GO:0044424~intracellular part; GOTERM_CC_2 GO:0005622~intracellular; GOTERM_CC_3 GO:0005622~intracellular; GOTERM_CC_1 GO:0043226~organelle; GOTERM_CC_4 GO:0044424~intracellular part; GOTERM_CC_3 GO:0043231~intracellular membrane-bounded organelle; GOTERM_CC_2 GO:0043227~membrane-bounded organelle; GOTERM_CC_3 GO:0044428~nuclear part; GOTERM_CC_2 GO:0043229~intracellular organelle; GOTERM_CC_3 GO:0043229~intracellular organelle; GOTERM_CC_4 GO:0044428~nuclear part; GOTERM_CC_5 GO:0044428~nuclear part; GOTERM_CC_4 GO:0043231~intracellular membrane-bounded organelle; GOTERM_CC_1 GO:0044422~organelle part; GOTERM_CC_5 GO:0043231~intracellular membrane-bounded organelle; GOTERM_CC_4 GO:0043229~intracellular organelle; GOTERM_CC_5 GO:0043229~intracellular organelle; GOTERM_CC_2 GO:0043228~non-membrane-bounded organelle; GOTERM_CC_3 GO:0043232~intracellular non-membrane-bounded organelle; GOTERM_CC_4 GO:0043232~intracellular non-membrane-bounded organelle; GOTERM_CC_5 GO:0043232~intracellular non-membrane-bounded organelle; GOTERM_MF_1 GO:0005488~binding |
| 6121-6171 | GOTERM_CC_4 GO:0005634~nucleus; GOTERM_CC_5 GO:0005634~nucleus; GOTERM_MF_2 GO:0005515~protein binding; GOTERM_CC_2 GO:0044424~intracellular part; GOTERM_CC_3 GO:0044424~intracellular part; GOTERM_CC_2 GO:0005622~intracellular; GOTERM_CC_3 GO:0005622~intracellular; GOTERM_CC_1 GO:0043226~organelle; GOTERM_CC_4 GO:0044424~intracellular part; GOTERM_CC_3 GO:0043231~intracellular membrane-bounded organelle; GOTERM_CC_2 GO:0043227~membrane-bounded organelle; GOTERM_CC_3 GO:0044428~nuclear part; GOTERM_CC_2 GO:0043229~intracellular organelle; GOTERM_CC_3 GO:0043229~intracellular organelle; GOTERM_CC_4 GO:0043231~intracellular membrane-bounded organelle; GOTERM_CC_5 GO:0043231~intracellular membrane-bounded organelle; GOTERM_CC_4 GO:0043229~intracellular organelle; GOTERM_CC_5 GO:0043229~intracellular organelle |
| 6121-6126 | GOTERM_MF_2 GO:0005515~protein binding; GOTERM_BP_2 GO:0043933~macromolecular complex subunit organization; GOTERM_BP_1 GO:0016043~cellular component organization |
| 6121-5699 | GOTERM_MF_2 GO:0005515~protein binding; GOTERM_BP_2 GO:0006810~transport; GOTERM_BP_1 GO:0051234~establishment of localization; GOTERM_BP_2 GO:0051234~establishment of localization; GOTERM_BP_3 GO:0006810~transport; GOTERM_BP_1 GO:0051179~localization |
| 6121-6404 | GOTERM_CC_4 GO:0005634~nucleus; GOTERM_CC_5 GO:0005634~nucleus; GOTERM_MF_2 GO:0005515~protein binding; GOTERM_CC_2 GO:0044424~intracellular part; GOTERM_CC_3 GO:0044424~intracellular part; GOTERM_CC_2 GO:0005622~intracellular; GOTERM_CC_3 GO:0005622~intracellular; GOTERM_CC_1 GO:0043226~organelle; GOTERM_CC_4 GO:0044424~intracellular part; GOTERM_CC_3 GO:0043231~intracellular membrane-bounded organelle; GOTERM_CC_2 GO:0043227~membrane-bounded organelle; GOTERM_CC_3 GO:0044428~nuclear part; GOTERM_CC_2 GO:0043229~intracellular organelle; GOTERM_CC_3 GO:0043229~intracellular organelle; GOTERM_CC_4 GO:0044428~nuclear part; GOTERM_CC_5 GO:0044428~nuclear part; GOTERM_CC_4 GO:0043231~intracellular membrane-bounded organelle; GOTERM_CC_5 GO:0043231~intracellular membrane-bounded organelle; GOTERM_CC_4 GO:0043229~intracellular organelle; GOTERM_CC_5 GO:0043229~intracellular organelle; GOTERM_BP_1 GO:0016043~cellular component organization; GOTERM_BP_1 GO:0009987~cellular process; GOTERM_CC_1 GO:0032991~macromolecular complex |
| 6121-6433 | GOTERM_BP_4 GO:0015031~protein transport; GOTERM_BP_4 GO:0045184~establishment of protein localization; GOTERM_BP_5 GO:0015031~protein transport; GOTERM_BP_2 GO:0033036~macromolecule localization; GOTERM_CC_5 GO:0005634~nucleus; GOTERM_MF_2 GO:0005515~protein binding; GOTERM_BP_5 GO:0006605~protein targeting; GOTERM_CC_1 GO:0043226~organelle; GOTERM_CC_3 GO:0043231~intracellular membrane-bounded organelle; GOTERM_CC_2 GO:0043227~membrane-bounded organelle; GOTERM_CC_2 GO:0043229~intracellular organelle; GOTERM_CC_3 GO:0043229~intracellular organelle; GOTERM_CC_4 GO:0043231~intracellular membrane-bounded organelle; GOTERM_CC_5 GO:0043231~intracellular membrane-bounded organelle; GOTERM_CC_4 GO:0043229~intracellular organelle; GOTERM_CC_5 GO:0043229~intracellular organelle; GOTERM_MF_1 GO:0005488~binding; GOTERM_CC_1 GO:0032991~macromolecular complex |
| 6121-6410 | GOTERM_CC_2 GO:0012505~endomembrane system; GOTERM_CC_3 GO:0012505~endomembrane system; GOTERM_CC_1 GO:0043226~organelle; GOTERM_BP_5 GO:0051347~positive regulation of transferase activity; GOTERM_CC_3 GO:0044428~nuclear part; GOTERM_CC_3 GO:0005737~cytoplasm; GOTERM_CC_2 GO:0043229~intracellular organelle; GOTERM_CC_3 GO:0043229~intracellular organelle; GOTERM_CC_4 GO:0044428~nuclear part; GOTERM_CC_5 GO:0044428~nuclear part; GOTERM_CC_1 GO:0044422~organelle part; GOTERM_CC_4 GO:0005737~cytoplasm; GOTERM_BP_4 GO:0043085~positive regulation of catalytic activity; GOTERM_CC_5 GO:0005737~cytoplasm; GOTERM_CC_2 GO:0044446~intracellular organelle part; GOTERM_CC_3 GO:0044446~intracellular organelle part; GOTERM_CC_2 GO:0044422~organelle part; GOTERM_BP_3 GO:0044093~positive regulation of molecular function; GOTERM_CC_4 GO:0044446~intracellular organelle part; GOTERM_BP_3 GO:0050790~regulation of catalytic activity; GOTERM_CC_5 GO:0044446~intracellular organelle part |
| 2437-6171 | GOTERM_CC_4 GO:0005634~nucleus; GOTERM_CC_5 GO:0005634~nucleus; GOTERM_CC_2 GO:0005622~intracellular; GOTERM_CC_3 GO:0005622~intracellular; GOTERM_CC_1 GO:0043226~organelle; GOTERM_CC_4 GO:0043229~intracellular organelle; GOTERM_CC_2 GO:0043229~intracellular organelle; GOTERM_CC_3 GO:0043229~intracellular organelle; GOTERM_CC_4 GO:0044424~intracellular part; GOTERM_CC_5 GO:0043229~intracellular organelle; GOTERM_CC_2 GO:0044424~intracellular part; GOTERM_CC_3 GO:0044424~intracellular part; GOTERM_CC_3 GO:0044428~nuclear part; GOTERM_CC_4 GO:0043231~intracellular membrane-bounded organelle; GOTERM_CC_3 GO:0043231~intracellular membrane-bounded organelle; GOTERM_CC_2 GO:0043227~membrane-bounded organelle; GOTERM_CC_5 GO:0043231~intracellular membrane-bounded organelle; GOTERM_BP_4 GO:0006350~transcription |
| 2437-5699 |  |
| 2437-5975 | GOTERM_MF_1 GO:0005488~binding; GOTERM_CC_2 GO:0005622~intracellular; GOTERM_CC_3 GO:0005622~intracellular; GOTERM_CC_4 GO:0044424~intracellular part; GOTERM_CC_2 GO:0044424~intracellular part; GOTERM_CC_3 GO:0044424~intracellular part |
| 2437-6433 | GOTERM_MF_1 GO:0005488~binding; GOTERM_CC_5 GO:0005634~nucleus; GOTERM_CC_3 GO:0070013~intracellular organelle lumen; GOTERM_CC_4 GO:0070013~intracellular organelle lumen; GOTERM_CC_5 GO:0070013~intracellular organelle lumen; GOTERM_CC_1 GO:0031974~membrane-enclosed lumen; GOTERM_CC_2 GO:0043233~organelle lumen; GOTERM_CC_3 GO:0043233~organelle lumen; GOTERM_CC_1 GO:0043226~organelle; GOTERM_CC_4 GO:0043229~intracellular organelle; GOTERM_CC_2 GO:0043229~intracellular organelle; GOTERM_CC_3 GO:0043229~intracellular organelle; GOTERM_CC_5 GO:0043229~intracellular organelle; GOTERM_CC_4 GO:0043231~intracellular membrane-bounded organelle; GOTERM_CC_3 GO:0043231~intracellular membrane-bounded organelle; GOTERM_CC_2 GO:0043227~membrane-bounded organelle; GOTERM_CC_5 GO:0043231~intracellular membrane-bounded organelle |
| 2437-6454 |  |
| 5115-3895 | GOTERM_BP_2 GO:0050878~regulation of body fluid levels; GOTERM_BP_3 GO:0050878~regulation of body fluid levels; GOTERM_BP_1 GO:0065007~biological regulation |
| 6171-3847 |  |
| 6171-4078 | GOTERM_CC_2 GO:0044424~intracellular part; GOTERM_CC_3 GO:0044424~intracellular part |
| 3895-4878 | GOTERM_BP_5 GO:0007596~blood coagulation; GOTERM_BP_4 GO:0007596~blood coagulation; GOTERM_BP_2 GO:0050817~coagulation; GOTERM_BP_3 GO:0007596~blood coagulation; GOTERM_BP_4 GO:0007599~hemostasis; GOTERM_BP_3 GO:0007599~hemostasis; GOTERM_BP_2 GO:0050878~regulation of body fluid levels; GOTERM_BP_3 GO:0050878~regulation of body fluid levels; GOTERM_BP_4 GO:0042060~wound healing; GOTERM_CC_5 GO:0030141~secretory granule; GOTERM_CC_1 GO:0005576~extracellular region; GOTERM_BP_3 GO:0009611~response to wounding; GOTERM_BP_2 GO:0009605~response to external stimulus; GOTERM_CC_5 GO:0044433~cytoplasmic vesicle part; GOTERM_CC_5 GO:0016023~cytoplasmic membrane-bounded vesicle; GOTERM_CC_4 GO:0044433~cytoplasmic vesicle part; GOTERM_CC_5 GO:0031410~cytoplasmic vesicle; GOTERM_CC_3 GO:0044433~cytoplasmic vesicle part; GOTERM_CC_4 GO:0016023~cytoplasmic membrane-bounded vesicle; GOTERM_BP_2 GO:0065008~regulation of biological quality; GOTERM_CC_2 GO:0044421~extracellular region part; GOTERM_CC_3 GO:0031988~membrane-bounded vesicle; GOTERM_CC_4 GO:0031410~cytoplasmic vesicle; GOTERM_CC_3 GO:0031410~cytoplasmic vesicle; GOTERM_CC_2 GO:0031982~vesicle; GOTERM_CC_1 GO:0044421~extracellular region part; GOTERM_BP_1 GO:0032501~multicellular organismal process; GOTERM_MF_5 GO:0004175~endopeptidase activity; GOTERM_MF_5 GO:0004252~serine-type endopeptidase activity; GOTERM_MF_5 GO:0008236~serine-type peptidase activity; GOTERM_BP_2 GO:0006950~response to stress; GOTERM_MF_2 GO:0001871~pattern binding; GOTERM_MF_3 GO:0030247~polysaccharide binding; GOTERM_BP_1 GO:0050896~response to stimulus; GOTERM_MF_4 GO:0005539~glycosaminoglycan binding; GOTERM_MF_3 GO:0017171~serine hydrolase activity; GOTERM_CC_2 GO:0005615~extracellular space; GOTERM_CC_3 GO:0005615~extracellular space; GOTERM_MF_4 GO:0008236~serine-type peptidase activity; GOTERM_BP_1 GO:0065007~biological regulation; GOTERM_MF_3 GO:0008233~peptidase activity; GOTERM_BP_5 GO:0006508~proteolysis; GOTERM_MF_4 GO:0070011~peptidase activity, acting on L-amino acid peptides |
| 3895-6116 | GOTERM_BP_1 GO:0050896~response to stimulus |
| 4878-6240 | GOTERM_CC_2 GO:0044421~extracellular region part; GOTERM_CC_1 GO:0044421~extracellular region part; GOTERM_BP_2 GO:0065008~regulation of biological quality; GOTERM_CC_5 GO:0044433~cytoplasmic vesicle part; GOTERM_BP_2 GO:0042221~response to chemical stimulus; GOTERM_CC_4 GO:0044433~cytoplasmic vesicle part; GOTERM_MF_5 GO:0005509~calcium ion binding; GOTERM_CC_3 GO:0044433~cytoplasmic vesicle part; GOTERM_CC_5 GO:0016023~cytoplasmic membrane-bounded vesicle; GOTERM_CC_5 GO:0031410~cytoplasmic vesicle; GOTERM_CC_4 GO:0016023~cytoplasmic membrane-bounded vesicle; GOTERM_CC_4 GO:0031410~cytoplasmic vesicle; GOTERM_CC_3 GO:0031988~membrane-bounded vesicle; GOTERM_CC_5 GO:0005737~cytoplasm; GOTERM_CC_3 GO:0031410~cytoplasmic vesicle; GOTERM_CC_2 GO:0031982~vesicle |
| 4878-3847 | GOTERM_CC_1 GO:0005576~extracellular region; GOTERM_BP_3 GO:0009611~response to wounding; GOTERM_BP_2 GO:0050817~coagulation; GOTERM_BP_3 GO:0007596~blood coagulation; GOTERM_BP_4 GO:0007596~blood coagulation; GOTERM_BP_3 GO:0007599~hemostasis; GOTERM_BP_4 GO:0007599~hemostasis; GOTERM_BP_5 GO:0007596~blood coagulation; GOTERM_BP_2 GO:0050878~regulation of body fluid levels; GOTERM_BP_3 GO:0050878~regulation of body fluid levels; GOTERM_BP_4 GO:0042060~wound healing; GOTERM_BP_2 GO:0065008~regulation of biological quality; GOTERM_CC_5 GO:0031093~platelet alpha granule lumen; GOTERM_CC_5 GO:0060205~cytoplasmic membrane-bounded vesicle lumen; GOTERM_CC_4 GO:0060205~cytoplasmic membrane-bounded vesicle lumen; GOTERM_CC_4 GO:0031983~vesicle lumen; GOTERM_CC_3 GO:0031983~vesicle lumen; GOTERM_CC_5 GO:0030141~secretory granule; GOTERM_CC_5 GO:0044433~cytoplasmic vesicle part; GOTERM_CC_4 GO:0044433~cytoplasmic vesicle part; GOTERM_CC_3 GO:0044433~cytoplasmic vesicle part; GOTERM_BP_1 GO:0040011~locomotion; GOTERM_CC_5 GO:0016023~cytoplasmic membrane-bounded vesicle; GOTERM_CC_5 GO:0031410~cytoplasmic vesicle; GOTERM_CC_4 GO:0016023~cytoplasmic membrane-bounded vesicle; GOTERM_CC_4 GO:0031410~cytoplasmic vesicle; GOTERM_CC_3 GO:0031988~membrane-bounded vesicle; GOTERM_CC_3 GO:0031410~cytoplasmic vesicle; GOTERM_CC_2 GO:0031982~vesicle; GOTERM_BP_1 GO:0051704~multi-organism process |
| 4878-2305 | GOTERM_BP_3 GO:0009611~response to wounding; GOTERM_BP_4 GO:0006954~inflammatory response; GOTERM_BP_3 GO:0006952~defense response; GOTERM_BP_2 GO:0042330~taxis; GOTERM_BP_3 GO:0006935~chemotaxis; GOTERM_BP_3 GO:0042330~taxis; GOTERM_BP_4 GO:0042330~taxis; GOTERM_BP_4 GO:0006935~chemotaxis; GOTERM_BP_5 GO:0006935~chemotaxis; GOTERM_BP_1 GO:0040011~locomotion; GOTERM_BP_3 GO:0007626~locomotory behavior; GOTERM_BP_2 GO:0006955~immune response; GOTERM_BP_2 GO:0007610~behavior; GOTERM_BP_1 GO:0051704~multi-organism process |
| 4878-2923 | GOTERM_MF_5 GO:0042379~chemokine receptor binding; GOTERM_MF_4 GO:0001664~G-protein-coupled receptor binding; GOTERM_BP_2 GO:0042330~taxis; GOTERM_BP_3 GO:0006935~chemotaxis; GOTERM_BP_3 GO:0042330~taxis; GOTERM_BP_4 GO:0042330~taxis; GOTERM_BP_4 GO:0006935~chemotaxis; GOTERM_BP_5 GO:0006935~chemotaxis; GOTERM_BP_1 GO:0040011~locomotion; GOTERM_BP_3 GO:0007626~locomotory behavior; GOTERM_BP_2 GO:0006955~immune response; GOTERM_BP_2 GO:0007610~behavior |
| 5316-4224 | GOTERM_CC_5 GO:0005887~integral to plasma membrane; GOTERM_CC_5 GO:0031226~intrinsic to plasma membrane; GOTERM_BP_5 GO:0003044~regulation of systemic arterial blood pressure mediated by a chemical signal; GOTERM_BP_4 GO:0019229~regulation of vasoconstriction; GOTERM_BP_4 GO:0003073~regulation of systemic arterial blood pressure; GOTERM_BP_5 GO:0019229~regulation of vasoconstriction; GOTERM_CC_4 GO:0031226~intrinsic to plasma membrane; GOTERM_BP_4 GO:0050727~regulation of inflammatory response; GOTERM_BP_5 GO:0050727~regulation of inflammatory response |
| 5316-6320 | GOTERM_CC_1 GO:0044421~extracellular region part; GOTERM_CC_2 GO:0044421~extracellular region part |
| 5316-3847 | GOTERM_CC_1 GO:0005576~extracellular region; GOTERM_CC_5 GO:0005887~integral to plasma membrane; GOTERM_CC_5 GO:0031226~intrinsic to plasma membrane; GOTERM_CC_4 GO:0031226~intrinsic to plasma membrane |
| 5316-3622 | GOTERM_BP_2 GO:0007586~digestion; GOTERM_CC_1 GO:0005576~extracellular region; GOTERM_BP_3 GO:0007631~feeding behavior |
| 6126-5931 |  |
| 6126-6433 | GOTERM_MF_2 GO:0005515~protein binding |
| 4224-5240 | GOTERM_BP_3 GO:0003013~circulatory system process; GOTERM_BP_4 GO:0008015~blood circulation; GOTERM_BP_5 GO:0003044~regulation of systemic arterial blood pressure mediated by a chemical signal; GOTERM_BP_4 GO:0003073~regulation of systemic arterial blood pressure; GOTERM_CC_5 GO:0005887~integral to plasma membrane; GOTERM_CC_4 GO:0031226~intrinsic to plasma membrane; GOTERM_CC_5 GO:0031226~intrinsic to plasma membrane; GOTERM_MF_5 GO:0004930~G-protein coupled receptor activity; GOTERM_BP_5 GO:0007186~G-protein coupled receptor protein signaling pathway; GOTERM_MF_4 GO:0004888~transmembrane receptor activity; GOTERM_MF_3 GO:0001653~peptide receptor activity; GOTERM_MF_4 GO:0001653~peptide receptor activity; GOTERM_MF_4 GO:0008528~peptide receptor activity, G-protein coupled; GOTERM_MF_3 GO:0004872~receptor activity; GOTERM_BP_4 GO:0007166~cell surface receptor linked signal transduction; GOTERM_CC_3 GO:0044459~plasma membrane part; GOTERM_MF_1 GO:0060089~molecular transducer activity; GOTERM_CC_4 GO:0044459~plasma membrane part; GOTERM_MF_5 GO:0008528~peptide receptor activity, G-protein coupled; GOTERM_BP_5 GO:0007166~cell surface receptor linked signal transduction; GOTERM_CC_5 GO:0044459~plasma membrane part; GOTERM_MF_2 GO:0004871~signal transducer activity; GOTERM_MF_2 GO:0042277~peptide binding; GOTERM_BP_5 GO:0019932~second-messenger-mediated signaling; GOTERM_BP_3 GO:0007165~signal transduction; GOTERM_BP_4 GO:0007165~signal transduction |
| 4224-5975 | GOTERM_MF_3 GO:0046983~protein dimerization activity |
| 4224-6248 | GOTERM_BP_3 GO:0008217~regulation of blood pressure; GOTERM_BP_5 GO:0008217~regulation of blood pressure; GOTERM_BP_3 GO:0003013~circulatory system process; GOTERM_BP_4 GO:0008015~blood circulation; GOTERM_BP_3 GO:0042127~regulation of cell proliferation; GOTERM_BP_4 GO:0042127~regulation of cell proliferation; GOTERM_MF_3 GO:0001653~peptide receptor activity; GOTERM_MF_4 GO:0001653~peptide receptor activity; GOTERM_MF_4 GO:0008528~peptide receptor activity, G-protein coupled; GOTERM_MF_5 GO:0008528~peptide receptor activity, G-protein coupled; GOTERM_MF_2 GO:0042277~peptide binding |
| 4224-5931 | GOTERM_BP_4 GO:0003018~vascular process in circulatory system; GOTERM_BP_3 GO:0003013~circulatory system process; GOTERM_BP_4 GO:0008015~blood circulation; GOTERM_BP_3 GO:0044057~regulation of system process; GOTERM_BP_4 GO:0044057~regulation of system process; GOTERM_BP_4 GO:0042312~regulation of vasodilation; GOTERM_BP_5 GO:0042312~regulation of vasodilation; GOTERM_CC_5 GO:0005887~integral to plasma membrane; GOTERM_CC_4 GO:0031226~intrinsic to plasma membrane; GOTERM_CC_5 GO:0031226~intrinsic to plasma membrane; GOTERM_BP_3 GO:0035150~regulation of tube size; GOTERM_BP_4 GO:0050880~regulation of blood vessel size; GOTERM_BP_5 GO:0007186~G-protein coupled receptor protein signaling pathway; GOTERM_BP_5 GO:0050880~regulation of blood vessel size; GOTERM_BP_2 GO:0003008~system process; GOTERM_BP_4 GO:0007166~cell surface receptor linked signal transduction; GOTERM_CC_3 GO:0044459~plasma membrane part; GOTERM_CC_4 GO:0044459~plasma membrane part; GOTERM_BP_5 GO:0007166~cell surface receptor linked signal transduction; GOTERM_CC_5 GO:0044459~plasma membrane part; GOTERM_BP_5 GO:0019932~second-messenger-mediated signaling; GOTERM_BP_5 GO:0030003~cellular cation homeostasis |
| 5240-5975 |  |
| 5240-5931 | GOTERM_BP_5 GO:0019932~second-messenger-mediated signaling; GOTERM_BP_5 GO:0007186~G-protein coupled receptor protein signaling pathway; GOTERM_BP_4 GO:0007166~cell surface receptor linked signal transduction; GOTERM_BP_5 GO:0007166~cell surface receptor linked signal transduction; GOTERM_CC_5 GO:0005887~integral to plasma membrane; GOTERM_CC_4 GO:0031226~intrinsic to plasma membrane; GOTERM_CC_5 GO:0031226~intrinsic to plasma membrane; GOTERM_CC_3 GO:0005886~plasma membrane; GOTERM_CC_4 GO:0005886~plasma membrane; GOTERM_CC_3 GO:0044459~plasma membrane part; GOTERM_CC_4 GO:0044459~plasma membrane part; GOTERM_CC_5 GO:0044459~plasma membrane part; GOTERM_BP_3 GO:0050790~regulation of catalytic activity; GOTERM_BP_4 GO:0043085~positive regulation of catalytic activity; GOTERM_BP_3 GO:0044093~positive regulation of molecular function; GOTERM_BP_2 GO:0065009~regulation of molecular function; GOTERM_CC_2 GO:0016020~membrane; GOTERM_CC_3 GO:0016020~membrane; GOTERM_BP_4 GO:0031279~regulation of cyclase activity; GOTERM_BP_4 GO:0051339~regulation of lyase activity; GOTERM_BP_4 GO:0051336~regulation of hydrolase activity; GOTERM_BP_5 GO:0030808~regulation of nucleotide biosynthetic process; GOTERM_BP_5 GO:0006140~regulation of nucleotide metabolic process; GOTERM_BP_3 GO:0003013~circulatory system process; GOTERM_BP_4 GO:0008015~blood circulation |
| 5240-6433 | GOTERM_CC_5 GO:0005887~integral to plasma membrane; GOTERM_CC_4 GO:0031226~intrinsic to plasma membrane; GOTERM_CC_5 GO:0031226~intrinsic to plasma membrane; GOTERM_CC_3 GO:0044459~plasma membrane part; GOTERM_CC_4 GO:0044459~plasma membrane part; GOTERM_CC_5 GO:0044459~plasma membrane part; GOTERM_BP_4 GO:0006954~inflammatory response; GOTERM_BP_2 GO:0009605~response to external stimulus; GOTERM_BP_3 GO:0009611~response to wounding; GOTERM_BP_3 GO:0006952~defense response; GOTERM_MF_5 GO:0004994~somatostatin receptor activity; GOTERM_BP_1 GO:0050896~response to stimulus; GOTERM_BP_5 GO:0006140~regulation of nucleotide metabolic process; GOTERM_BP_2 GO:0006950~response to stress |
| 5699-6240 | GOTERM_CC_4 GO:0005886~plasma membrane; GOTERM_BP_3 GO:0006810~transport; GOTERM_BP_1 GO:0051179~localization; GOTERM_CC_3 GO:0005886~plasma membrane; GOTERM_CC_2 GO:0000267~cell fraction; GOTERM_CC_3 GO:0000267~cell fraction; GOTERM_CC_4 GO:0005624~membrane fraction; GOTERM_CC_5 GO:0005624~membrane fraction; GOTERM_CC_4 GO:0005626~insoluble fraction; GOTERM_BP_2 GO:0006810~transport; GOTERM_BP_1 GO:0051234~establishment of localization; GOTERM_BP_2 GO:0051234~establishment of localization; GOTERM_CC_3 GO:0005626~insoluble fraction; GOTERM_MF_5 GO:0005509~calcium ion binding; GOTERM_MF_2 GO:0005515~protein binding; GOTERM_BP_3 GO:0032879~regulation of localization; GOTERM_BP_2 GO:0032879~regulation of localization |
| 5699-6320 | GOTERM_MF_2 GO:0005515~protein binding |
| 5699-4305 | GOTERM_CC_4 GO:0005886~plasma membrane; GOTERM_CC_4 GO:0044459~plasma membrane part; GOTERM_CC_5 GO:0044459~plasma membrane part; GOTERM_CC_5 GO:0005887~integral to plasma membrane; GOTERM_CC_4 GO:0031226~intrinsic to plasma membrane; GOTERM_CC_5 GO:0031226~intrinsic to plasma membrane; GOTERM_CC_3 GO:0044459~plasma membrane part; GOTERM_CC_3 GO:0005886~plasma membrane; GOTERM_MF_5 GO:0005509~calcium ion binding; GOTERM_MF_2 GO:0005515~protein binding; GOTERM_BP_5 GO:0015674~di-, tri-valent inorganic cation transport; GOTERM_BP_3 GO:0032879~regulation of localization; GOTERM_BP_2 GO:0032879~regulation of localization; GOTERM_CC_1 GO:0005576~extracellular region; GOTERM_MF_3 GO:0004872~receptor activity |
| 6240-6433 | GOTERM_CC_5 GO:0016023~cytoplasmic membrane-bounded vesicle; GOTERM_CC_3 GO:0031988~membrane-bounded vesicle; GOTERM_CC_4 GO:0016023~cytoplasmic membrane-bounded vesicle; GOTERM_CC_3 GO:0031410~cytoplasmic vesicle; GOTERM_CC_5 GO:0031410~cytoplasmic vesicle; GOTERM_CC_4 GO:0031410~cytoplasmic vesicle; GOTERM_CC_2 GO:0031982~vesicle; GOTERM_BP_2 GO:0033036~macromolecule localization; GOTERM_BP_4 GO:0015031~protein transport; GOTERM_BP_4 GO:0045184~establishment of protein localization; GOTERM_BP_5 GO:0015031~protein transport; GOTERM_CC_1 GO:0044421~extracellular region part; GOTERM_BP_5 GO:0002791~regulation of peptide secretion; GOTERM_CC_2 GO:0044421~extracellular region part; GOTERM_MF_1 GO:0005488~binding; GOTERM_BP_4 GO:0048878~chemical homeostasis; GOTERM_CC_5 GO:0000323~lytic vacuole; GOTERM_CC_5 GO:0005773~vacuole; GOTERM_CC_4 GO:0005773~vacuole; GOTERM_MF_2 GO:0005515~protein binding; GOTERM_CC_5 GO:0044444~cytoplasmic part; GOTERM_MF_3 GO:0051082~unfolded protein binding |
| 6320-6433 | GOTERM_CC_3 GO:0005578~proteinaceous extracellular matrix; GOTERM_CC_4 GO:0005578~proteinaceous extracellular matrix; GOTERM_CC_2 GO:0031012~extracellular matrix; GOTERM_CC_3 GO:0031012~extracellular matrix; GOTERM_CC_1 GO:0044421~extracellular region part; GOTERM_CC_2 GO:0044421~extracellular region part; GOTERM_MF_2 GO:0005515~protein binding |
| 5975-6433 | GOTERM_MF_3 GO:0046983~protein dimerization activity; GOTERM_MF_1 GO:0005488~binding |
| 4958-6433 | GOTERM_CC_2 GO:0005615~extracellular space; GOTERM_CC_3 GO:0005615~extracellular space; GOTERM_BP_3 GO:0042127~regulation of cell proliferation; GOTERM_BP_4 GO:0042127~regulation of cell proliferation; GOTERM_CC_1 GO:0044421~extracellular region part; GOTERM_CC_2 GO:0044421~extracellular region part; GOTERM_CC_1 GO:0005576~extracellular region; GOTERM_BP_2 GO:0048523~negative regulation of cellular process; GOTERM_BP_3 GO:0048523~negative regulation of cellular process; GOTERM_MF_2 GO:0005515~protein binding; GOTERM_BP_4 GO:0048523~negative regulation of cellular process; GOTERM_BP_2 GO:0048519~negative regulation of biological process; GOTERM_BP_3 GO:0048519~negative regulation of biological process; GOTERM_BP_1 GO:0002376~immune system process; GOTERM_BP_2 GO:0002682~regulation of immune system process; GOTERM_BP_3 GO:0002682~regulation of immune system process |
| 3847-6433 | GOTERM_MF_2 GO:0003823~antigen binding; GOTERM_CC_3 GO:0044459~plasma membrane part; GOTERM_CC_4 GO:0044459~plasma membrane part; GOTERM_CC_5 GO:0044459~plasma membrane part; GOTERM_CC_5 GO:0005887~integral to plasma membrane; GOTERM_CC_4 GO:0031226~intrinsic to plasma membrane; GOTERM_CC_5 GO:0031226~intrinsic to plasma membrane; GOTERM_CC_1 GO:0005576~extracellular region; GOTERM_BP_2 GO:0019882~antigen processing and presentation; GOTERM_CC_5 GO:0030141~secretory granule; GOTERM_CC_3 GO:0031988~membrane-bounded vesicle; GOTERM_CC_4 GO:0016023~cytoplasmic membrane-bounded vesicle; GOTERM_CC_5 GO:0016023~cytoplasmic membrane-bounded vesicle; GOTERM_BP_3 GO:0009611~response to wounding; GOTERM_CC_3 GO:0031410~cytoplasmic vesicle; GOTERM_CC_4 GO:0031410~cytoplasmic vesicle; GOTERM_CC_2 GO:0031982~vesicle; GOTERM_CC_5 GO:0031410~cytoplasmic vesicle |
| 2305-2923 | GOTERM_BP_2 GO:0042330~taxis; GOTERM_BP_3 GO:0006935~chemotaxis; GOTERM_BP_3 GO:0042330~taxis; GOTERM_BP_4 GO:0006935~chemotaxis; GOTERM_BP_4 GO:0042330~taxis; GOTERM_BP_5 GO:0006935~chemotaxis; GOTERM_BP_3 GO:0007626~locomotory behavior; GOTERM_BP_5 GO:0030003~cellular cation homeostasis; GOTERM_BP_3 GO:0055082~cellular chemical homeostasis; GOTERM_BP_4 GO:0006873~cellular ion homeostasis; GOTERM_BP_1 GO:0040011~locomotion; GOTERM_BP_5 GO:0055082~cellular chemical homeostasis; GOTERM_BP_5 GO:0050801~ion homeostasis; GOTERM_BP_2 GO:0019725~cellular homeostasis; GOTERM_BP_2 GO:0007610~behavior; GOTERM_BP_4 GO:0019725~cellular homeostasis; GOTERM_BP_4 GO:0048878~chemical homeostasis; GOTERM_BP_2 GO:0006955~immune response |
| 2305-6433 | GOTERM_BP_4 GO:0006954~inflammatory response; GOTERM_BP_4 GO:0048878~chemical homeostasis; GOTERM_BP_3 GO:0009611~response to wounding; GOTERM_BP_3 GO:0006952~defense response; GOTERM_BP_2 GO:0006955~immune response |
| 6248-6433 | GOTERM_CC_2 GO:0044421~extracellular region part; GOTERM_CC_1 GO:0044421~extracellular region part; GOTERM_CC_2 GO:0031012~extracellular matrix; GOTERM_CC_3 GO:0031012~extracellular matrix; GOTERM_CC_1 GO:0005576~extracellular region; GOTERM_CC_3 GO:0005578~proteinaceous extracellular matrix; GOTERM_CC_4 GO:0005578~proteinaceous extracellular matrix; GOTERM_MF_2 GO:0005515~protein binding; GOTERM_CC_2 GO:0005615~extracellular space; GOTERM_CC_3 GO:0005615~extracellular space; GOTERM_BP_4 GO:0042127~regulation of cell proliferation; GOTERM_BP_3 GO:0042127~regulation of cell proliferation; GOTERM_BP_4 GO:0008285~negative regulation of cell proliferation; GOTERM_BP_3 GO:0008285~negative regulation of cell proliferation; GOTERM_BP_5 GO:0008285~negative regulation of cell proliferation; GOTERM_BP_4 GO:0006801~superoxide metabolic process |
| 6248-6454 |  |
| 6248-6116 | GOTERM_MF_2 GO:0005515~protein binding |
| 5822-6116 | GOTERM_BP_3 GO:0060255~regulation of macromolecule metabolic process; GOTERM_MF_1 GO:0060089~molecular transducer activity; GOTERM_BP_4 GO:0060255~regulation of macromolecule metabolic process; GOTERM_MF_2 GO:0004871~signal transducer activity |
| 5822-6410 |  |
| 6404-6433 | GOTERM_CC_5 GO:0005634~nucleus; GOTERM_CC_1 GO:0043226~organelle; GOTERM_BP_3 GO:0051172~negative regulation of nitrogen compound metabolic process; GOTERM_BP_4 GO:0045934~negative regulation of nucleobase, nucleoside, nucleotide and nucleic acid metabolic process; GOTERM_BP_4 GO:0051172~negative regulation of nitrogen compound metabolic process; GOTERM_BP_4 GO:0010558~negative regulation of macromolecule biosynthetic process; GOTERM_BP_5 GO:0045934~negative regulation of nucleobase, nucleoside, nucleotide and nucleic acid metabolic process; GOTERM_BP_5 GO:0051172~negative regulation of nitrogen compound metabolic process; GOTERM_CC_2 GO:0043229~intracellular organelle; GOTERM_CC_3 GO:0043229~intracellular organelle; GOTERM_CC_4 GO:0043229~intracellular organelle; GOTERM_CC_5 GO:0043229~intracellular organelle; GOTERM_BP_3 GO:0031324~negative regulation of cellular metabolic process; GOTERM_BP_4 GO:0031324~negative regulation of cellular metabolic process; GOTERM_MF_2 GO:0005515~protein binding; GOTERM_BP_5 GO:0031324~negative regulation of cellular metabolic process; GOTERM_CC_3 GO:0043231~intracellular membrane-bounded organelle; GOTERM_CC_2 GO:0043227~membrane-bounded organelle; GOTERM_BP_4 GO:0009892~negative regulation of metabolic process; GOTERM_CC_3 GO:0070013~intracellular organelle lumen; GOTERM_CC_1 GO:0031974~membrane-enclosed lumen; GOTERM_CC_2 GO:0043233~organelle lumen; GOTERM_CC_3 GO:0043233~organelle lumen; GOTERM_CC_4 GO:0070013~intracellular organelle lumen; GOTERM_CC_5 GO:0070013~intracellular organelle lumen; GOTERM_CC_4 GO:0043231~intracellular membrane-bounded organelle; GOTERM_CC_5 GO:0043231~intracellular membrane-bounded organelle; GOTERM_BP_2 GO:0048523~negative regulation of cellular process; GOTERM_BP_3 GO:0048523~negative regulation of cellular process; GOTERM_BP_4 GO:0048523~negative regulation of cellular process; GOTERM_CC_1 GO:0032991~macromolecular complex; GOTERM_BP_2 GO:0048519~negative regulation of biological process |
| 6404-4078 | GOTERM_CC_2 GO:0044424~intracellular part; GOTERM_CC_3 GO:0044424~intracellular part |
| 4305-6454 | GOTERM_CC_5 GO:0005887~integral to plasma membrane; GOTERM_CC_4 GO:0031226~intrinsic to plasma membrane; GOTERM_CC_5 GO:0031226~intrinsic to plasma membrane; GOTERM_CC_4 GO:0044459~plasma membrane part; GOTERM_CC_5 GO:0044459~plasma membrane part; GOTERM_CC_3 GO:0044459~plasma membrane part |
| 6433-6454 | GOTERM_CC_5 GO:0044459~plasma membrane part; GOTERM_CC_5 GO:0005887~integral to plasma membrane; GOTERM_CC_4 GO:0044459~plasma membrane part; GOTERM_CC_3 GO:0044459~plasma membrane part; GOTERM_CC_5 GO:0031226~intrinsic to plasma membrane; GOTERM_CC_4 GO:0031226~intrinsic to plasma membrane |
| 6454-6116 |  |
