# Supplementary material for: Disease Gene Interaction Pathways: A Potential Framework for How Disease Genes Associate by Disease-Risk Modules
Source: PLoS One. 2011 Sep 6;6(9):e24495. doi: 10.1371/journal.pone.0024495 (PMC3167857; doi:10.1371/journal.pone.0024495)
Supplement: Table S3 — PubMed ID in which KEGG pathways enriched have been proved to be correlated with CAD. (DOC) [file pone.0024495.s006.doc]

**Table S3. PubMed ID in which KEGG pathways enriched have been proved to be correlated with CAD.**

| **pathway** | **PubMed ID associated with CAD** |
| --- | --- |
| ABC transporters | PMID: 15777535 PMID: 15053163 PMID: 12679197 PMID: 12359125 PMID: 12137403 |
| Acute myeloid leukemia | PMID: 18772122 |
| Adherens junction |  |
| Adipocytokine signaling pathway |  |
| Aldosterone-regulated sodium reabsorption |  |
| Alzheimer's disease | PMID: 20214536 PMID: 20184726 PMID: 20178050 PMID: 20088810 PMID: 20050287 PMID: 20042103 PMID: 19826563 PMID: 19673684 PMID: 19673450 PMID: 19639021 PMID: 19590498 PMID: 19498075 PMID: 19381858 PMID: 19281826 PMID: 19196369 PMID: 19096162 PMID: 19091445 PMID: 19079672 PMID: 18987747 PMID: 18626887 PMID: 18585852 PMID: 18537039 PMID: 18487142 PMID: 18457539 PMID: 18358084 PMID: 18348729 PMID: 18334739 PMID: 18204247 PMID: 18198422 PMID: 18179501 PMID: 18069348 PMID: 18046875 PMID: 17990970 PMID: 17711428 PMID: 17594534 PMID: 17504224 PMID: 17490685 PMID: 17473899 PMID: 17430249 PMID: 17374166 PMID: 17239370 PMID: 17227056 PMID: 17217095 PMID: 17200729 PMID: 17175070 PMID: 17168664 PMID: 17137520 PMID: 17116649 PMID: 17022108 PMID: 16965549 PMID: 16945213 PMID: 16892270 PMID: 16846553 PMID: 16842191 PMID: 16786033 PMID: 16752360 PMID: 16702784 PMID: 16680037 PMID: 16676611 PMID: 16621646 PMID: 16600299 PMID: 16596262 PMID: 16484840 PMID: 16453055 PMID: 16399888 PMID: 16385401 PMID: 16337092 PMID: 16327258 PMID: 16194042 PMID: 16124393 PMID: 16116114 PMID: 16108925 PMID: 16051686 PMID: 16028714 PMID: 15935563 PMID: 15899673 PMID: 15814164 PMID: 15718045 PMID: 15665398 PMID: 15630634 PMID: 15591750 PMID: 15582912 PMID: 15573849 PMID: 15567406 PMID: 15505371 PMID: 15448586 PMID: 15370197 PMID: 15181020 PMID: 15138623 PMID: 14746413 PMID: 14698449 PMID: 14662106 PMID: 14530190 PMID: 14518174 PMID: 12927758 PMID: 12898587 PMID: 12873034 PMID: 12816555 PMID: 12799433 PMID: 12732794 PMID: 12714262 PMID: 12684095 PMID: 12670623 PMID: 12649361 PMID: 12618290 PMID: 12587416 PMID: 12521230 PMID: 12515903 PMID: 12492654 PMID: 12480756 PMID: 12480731 PMID: 12480099 PMID: 12474023 PMID: 12473166 PMID: 12402342 PMID: 12212783 PMID: 12188398 PMID: 12126795 PMID: 12074828 PMID: 12019344 PMID: 12021416 PMID: 11882522 PMID: 11844650 PMID: 11834456 PMID: 11830193 PMID: 11828885 PMID: 11795362 PMID: 11803456 PMID: 11732264 PMID: 11716989 PMID: 11657425 PMID: 11501342 PMID: 11454010 PMID: 11440749 PMID: 11359359 PMID: 11296469 PMID: 11280031 PMID: 11256794 PMID: 11201186 PMID: 11133172 PMID: 11089820 PMID: 10961416 PMID: 10955124 PMID: 10936882 PMID: 10867217 PMID: 10842712 PMID: 10818499 PMID: 10774934 PMID: 10738542 PMID: 10661610 PMID: 10642431 PMID: 10614714 PMID: 10588960 PMID: 10448520 PMID: 10430423 PMID: 10416034 PMID: 9973659 PMID: 9848802 PMID: 9549718 PMID: 9536999 PMID: 9381032 PMID: 9329686 PMID: 9403125 PMID: 9229184 PMID: 9222173 PMID: 9129718 PMID: 9397382 PMID: 8996828 PMID: 8809005 PMID: 8879956 PMID: 8791246 PMID: 8867586 PMID: 8744411 PMID: 8530010 PMID: 7595642 PMID: 7740560 PMID: 7664194 PMID: 7481373 PMID: 7772071 PMID: 12319855 PMID: 7765749 PMID: 7918303 PMID: 7879640 PMID: 8503257 PMID: 8440995 PMID: 1704106 PMID: 3197048 PMID: 3619988 PMID: 3539962 |
| Amino sugar and nucleotide sugar metabolism | PMID: 11150394 |
| Aminoacyl-tRNA biosynthesis |  |
| Antigen processing and presentation |  |
| Apoptosis | PMID: 20395596 PMID: 20375694 PMID: 20211468 PMID: 20162343 PMID: 20139667 PMID: 20103334 PMID: 20102890 PMID: 20034655 PMID: 20031476 PMID: 20015493 PMID: 20009389 PMID: 19944316 PMID: 19861349 PMID: 19804343 PMID: 19789393 PMID: 19770641 PMID: 19762786 PMID: 19762778 PMID: 19751413 PMID: 19750006 PMID: 19726822 PMID: 19707922 PMID: 19706371 PMID: 19694500 PMID: 19690392 PMID: 19685158 PMID: 19667981 PMID: 19590843 PMID: 19533303 PMID: 19506320 PMID: 19505454 PMID: 19502560 PMID: 19461053 PMID: 19454488 PMID: 19441061 PMID: 19398656 PMID: 19343290 PMID: 19333379 PMID: 19275271 PMID: 19204406 PMID: 19193454 PMID: 19132996 PMID: 19132213 PMID: 19103817 PMID: 19099985 PMID: 19087127 PMID: 19068010 PMID: 19059213 PMID: 19056397 PMID: 19026018 PMID: 19020458 PMID: 19020338 PMID: 19010346 PMID: 19007931 PMID: 18923250 PMID: 18847304 PMID: 18827461 PMID: 18802021 PMID: 18802018 PMID: 18781471 PMID: 18761720 PMID: 18758183 PMID: 18724960 PMID: 18705697 PMID: 18664030 PMID: 18660453 PMID: 18645343 PMID: 18645246 PMID: 18634988 PMID: 18625049 PMID: 18582628 PMID: 18495127 PMID: 18474347 PMID: 18473868 PMID: 18420941 PMID: 18418425 PMID: 18409173 PMID: 18389485 PMID: 18386601 PMID: 18350717 PMID: 18344027 PMID: 18327085 PMID: 18311803 PMID: 18301873 PMID: 18298949 PMID: 18286426 PMID: 18281817 PMID: 18273488 PMID: 18256747 PMID: 18243052 PMID: 18235500 PMID: 18220582 PMID: 18208353 PMID: 18192848 PMID: 18180097 PMID: 18178561 PMID: 18154960 PMID: 18097624 PMID: 18083403 PMID: 18068708 PMID: 18066098 PMID: 18054314 PMID: 18004652 PMID: 18004118 PMID: 18001320 PMID: 17994273 PMID: 17979794 PMID: 17971205 PMID: 17967822 PMID: 17885419 PMID: 17876797 PMID: 17805085 PMID: 17709641 PMID: 17698735 PMID: 17640543 PMID: 17626327 PMID: 17604327 PMID: 17591646 PMID: 17584062 PMID: 17582591 PMID: 17558240 PMID: 17541821 PMID: 17534423 PMID: 17513374 PMID: 17498055 PMID: 17496367 PMID: 17487248 PMID: 17460198 PMID: 17453672 PMID: 17420351 PMID: 17391216 PMID: 17372667 PMID: 17338746 PMID: 17332488 PMID: 17313769 PMID: 17299913 PMID: 17275277 PMID: 17255533 PMID: 17220179 PMID: 17175040 PMID: 17172932 PMID: 17172803 PMID: 17152923 PMID: 17140552 PMID: 17136687 PMID: 17126678 PMID: 17123106 PMID: 16997625 PMID: 16995309 PMID: 16972538 PMID: 16960505 PMID: 16960105 PMID: 16954559 PMID: 16949468 PMID: 19804196 PMID: 16935694 PMID: 16907655 PMID: 16905964 PMID: 16905125 PMID: 16868702 PMID: 16846604 PMID: 16831207 PMID: 16816452 PMID: 16807648 PMID: 16805218 PMID: 16796160 PMID: 16767300 PMID: 16760868 PMID: 16759640 PMID: 16759520 PMID: 16678847 PMID: 16678804 PMID: 16631517 PMID: 16613180 PMID: 16613176 PMID: 16564529 PMID: 16555682 PMID: 16531984 PMID: 16517248 PMID: 16498517 PMID: 16466728 PMID: 16458149 PMID: 16298262 PMID: 16297799 PMID: 16294826 PMID: 16288714 PMID: 16259786 PMID: 16239600 PMID: 16218879 PMID: 16211207 PMID: 16170677 PMID: 16081267 PMID: 16032783 PMID: 16019609 PMID: 16008155 PMID: 16005051 PMID: 15982609 PMID: 15976317 PMID: 15952913 PMID: 15910870 PMID: 15888032 PMID: 15878008 PMID: 15867794 PMID: 15853632 PMID: 15842352 PMID: 15840864 PMID: 15831806 PMID: 15824087 PMID: 15823794 PMID: 15823277 PMID: 15820197 PMID: 15818089 PMID: 15808757 PMID: 15775191 PMID: 15772688 PMID: 15753227 PMID: 15735404 PMID: 15723685 PMID: 15716792 PMID: 15711199 PMID: 15695426 PMID: 15666476 PMID: 15649656 PMID: 15648182 PMID: 15638783 PMID: 15629664 PMID: 15596027 PMID: 15591005 PMID: 15589739 PMID: 15561331 PMID: 15531776 PMID: 15493951 PMID: 15457400 PMID: 15364863 PMID: 15364862 PMID: 15364800 PMID: 15361793 PMID: 15325020 PMID: 15322703 PMID: 15319365 PMID: 15301727 PMID: 15284284 PMID: 15280201 PMID: 15277326 PMID: 15242971 PMID: 15231505 PMID: 15224028 PMID: 15223025 PMID: 15217794 PMID: 15205608 PMID: 15167223 PMID: 15166782 PMID: 15151487 PMID: 15142859 PMID: 15090261 PMID: 15078140 PMID: 15067574 PMID: 15033813 PMID: 15027449 PMID: 15024703 PMID: 15006420 PMID: 14984432 PMID: 14977533 PMID: 14976136 PMID: 14761784 PMID: 14752031 PMID: 14735458 PMID: 14735044 PMID: 14732197 PMID: 14728000 PMID: 14727945 PMID: 14726373 PMID: 14724337 PMID: 14719566 PMID: 14691039 PMID: 14683657 PMID: 14683650 PMID: 14660654 PMID: 14654064 PMID: 14626194 PMID: 14615291 PMID: 14568225 PMID: 14555784 PMID: 14537103 PMID: 14507572 PMID: 12967330 PMID: 12957608 PMID: 12951604 PMID: 12939212 PMID: 12930259 PMID: 12930163 PMID: 12907943 PMID: 12907417 PMID: 12902549 PMID: 12881477 PMID: 12858127 PMID: 12843690 PMID: 12822196 PMID: 12808483 PMID: 12808360 PMID: 12796750 PMID: 12791514 PMID: 12748253 PMID: 12737064 PMID: 12730697 PMID: 12684095 PMID: 12655276 PMID: 12629330 PMID: 12616810 PMID: 12601880 PMID: 12593499 PMID: 12530513 PMID: 12502909 PMID: 12410519 PMID: 12401536 PMID: 12393934 PMID: 12361836 PMID: 12352017 PMID: 12352014 PMID: 12226479 PMID: 12205049 PMID: 12170032 PMID: 12148909 PMID: 12127376 PMID: 12115596 PMID: 12093880 PMID: 12074967 PMID: 12051460 PMID: 12020497 PMID: 12002259 PMID: 12001845 PMID: 11997428 PMID: 11967121 PMID: 11924703 PMID: 11917196 PMID: 11910304 PMID: 11887550 PMID: 11884278 PMID: 11882312 PMID: 11882051 PMID: 11868908 PMID: 11844871 PMID: 11796243 PMID: 11811329 PMID: 11790697 PMID: 11790271 PMID: 11787806 PMID: 11728960 PMID: 11717151 PMID: 11714653 PMID: 11691504 PMID: 11641234 PMID: 11602504 PMID: 11584167 PMID: 11551494 PMID: 11522678 PMID: 11499717 PMID: 11491202 PMID: 11479936 PMID: 11453575 PMID: 11451747 PMID: 11420155 PMID: 11407740 PMID: 11400570 PMID: 11327332 PMID: 11299235 PMID: 11281300 PMID: 11276917 PMID: 11251126 PMID: 11215532 PMID: 11176010 PMID: 11174871 PMID: 11170257 PMID: 10751558 PMID: 11152216 PMID: 11151786 PMID: 11139364 PMID: 11060730 PMID: 10976784 PMID: 10961416 PMID: 10910059 PMID: 10865844 PMID: 10860844 PMID: 10829247 PMID: 10774934 PMID: 10728394 PMID: 10694520 PMID: 10683356 PMID: 10638219 PMID: 10630765 PMID: 10623660 PMID: 10599541 PMID: 10567307 PMID: 10502209 PMID: 10483047 PMID: 10422461 PMID: 10421970 PMID: 10412643 PMID: 10403535 PMID: 10384826 PMID: 10376200 PMID: 10363980 PMID: 10353478 PMID: 10350677 PMID: 10341848 PMID: 10200946 PMID: 10077455 PMID: 10030383 PMID: 9888861 PMID: 9864601 PMID: 9717052 PMID: 9710689 PMID: 9546379 PMID: 9504148 PMID: 9504147 PMID: 9503173 PMID: 9480584 PMID: 9457444 PMID: 9457443 PMID: 9457442 PMID: 9457441 PMID: 9375940 PMID: 9342556 PMID: 9417747 PMID: 9192761 PMID: 9199270 PMID: 9137215 PMID: 9237032 PMID: 9429840 PMID: 9387108 PMID: 8968671 PMID: 8928549 PMID: 8642787 PMID: 7639326 PMID: 7758173 |
| Arachidonic acid metabolism | PMID: 20093140 PMID: 19689400 PMID: 19534694 PMID: 16759817 PMID: 12566962 PMID: 12444802 PMID: 12357134 PMID: 11208362 PMID: 9556499 PMID: 8790031 PMID: 8063366 PMID: 8065610 PMID: 3276185 PMID: 3248116 PMID: 3552319 PMID: 3160477 PMID: 6230918 |
| Arginine and proline metabolism | PMID: 19357637 |
| Arrhythmogenic right ventricular cardiomyopathy (ARVC) | PMID: 19214409 PMID: 19097213 PMID: 17533523 PMID: 17274894 PMID: 16645365 PMID: 15241541 PMID: 14663615 |
| Axon guidance |  |
| B cell receptor signaling pathway |  |
| Basal cell carcinoma | PMID: 19578363 PMID: 10940116 PMID: 1858158 |
| Biosynthesis of unsaturated fatty acids | PMID: 20219966 PMID: 19996015 PMID: 19946232 PMID: 19917448 PMID: 19916363 PMID: 19852883 PMID: 19852881 PMID: 19850308 PMID: 19732603 PMID: 19689400 PMID: 19648503 PMID: 19631352 PMID: 19628666 PMID: 19628194 PMID: 19508722 PMID: 19506932 PMID: 19506338 PMID: 19491533 PMID: 19447387 PMID: 19424218 PMID: 19424216 PMID: 19422375 PMID: 19422139 PMID: 19410579 PMID: 19399016 PMID: 19397693 PMID: 19390588 PMID: 19351291 PMID: 19345947 PMID: 19282639 PMID: 19272447 PMID: 19263263 PMID: 19248856 PMID: 19244379 PMID: 19241310 PMID: 19223688 PMID: 19217512 PMID: 19215971 PMID: 19196081 PMID: 19185299 PMID: 19150063 PMID: 19130089 PMID: 19080732 PMID: 19046748 PMID: 19040594 PMID: 19009217 PMID: 18989535 PMID: 18952714 PMID: 18951772 PMID: 18937898 PMID: 18937892 PMID: 18849551 PMID: 18842780 PMID: 18831918 PMID: 18812674 PMID: 18804984 PMID: 18802021 PMID: 18795577 PMID: 18769836 PMID: 18703045 PMID: 18662803 PMID: 18657307 PMID: 18645486 PMID: 18638594 PMID: 18625049 PMID: 18611440 PMID: 18575786 PMID: 18560524 PMID: 18552508 PMID: 18541601 PMID: 18536782 PMID: 18520610 PMID: 18507534 PMID: 18505546 PMID: 18296340 PMID: 18296320 PMID: 18499236 PMID: 18492846 PMID: 18469350 PMID: 18436228 PMID: 18431525 PMID: 18390905 PMID: 18377789 PMID: 18348729 PMID: 18280595 PMID: 18277607 PMID: 18246409 PMID: 18220672 PMID: 18184673 PMID: 18160071 PMID: 18077475 PMID: 18068523 PMID: 18065577 PMID: 18064329 PMID: 18059206 PMID: 18054937 PMID: 18051228 PMID: 18004111 PMID: 17991651 PMID: 17963772 PMID: 17876199 PMID: 17876190 PMID: 17927900 PMID: 17921408 PMID: 17914635 PMID: 17904342 PMID: 17869078 PMID: 17853322 PMID: 17700222 PMID: 17693694 PMID: 17690622 PMID: 17668458 PMID: 17663734 PMID: 17628847 PMID: 17612056 PMID: 17597662 PMID: 17569885 PMID: 17569678 PMID: 17555951 PMID: 17513374 PMID: 17507020 PMID: 17470694 PMID: 17466485 PMID: 17461697 PMID: 17456993 PMID: 17456471 PMID: 17441383 PMID: 17437143 PMID: 17327871 PMID: 17268422 PMID: 17257774 PMID: 17250811 PMID: 17237316 PMID: 17229894 PMID: 17207282 PMID: 17176247 PMID: 17174609 PMID: 17168664 PMID: 17167286 PMID: 17167284 PMID: 17167283 PMID: 17145556 PMID: 17086091 PMID: 17073704 PMID: 17058434 PMID: 17045070 PMID: 16997625 PMID: 16987874 PMID: 16979604 PMID: 16919516 PMID: 16919513 PMID: 16919512 PMID: 16905596 PMID: 16902597 PMID: 16892270 PMID: 16890573 PMID: 16888437 PMID: 16865092 PMID: 16843720 PMID: 16839864 PMID: 16824612 PMID: 16805758 PMID: 16801473 PMID: 16772452 PMID: 16770944 PMID: 16759817 PMID: 16741195 PMID: 16713753 PMID: 16713393 PMID: 16713389 PMID: 16702043 PMID: 16635110 PMID: 16634535 PMID: 16579978 PMID: 16571859 PMID: 16563364 PMID: 16531984 PMID: 16517503 PMID: 16514067 PMID: 16512956 PMID: 16488419 PMID: 16477807 PMID: 16476615 PMID: 16472797 PMID: 16458317 PMID: 16425789 PMID: 16400036 PMID: 16359994 PMID: 16329464 PMID: 16322351 PMID: 16275179 PMID: 16272805 PMID: 16256769 PMID: 16238116 PMID: 16216720 PMID: 16190133 PMID: 16154495 PMID: 16140305 PMID: 16131150 PMID: 16039290 PMID: 16028714 PMID: 16019008 PMID: 16018822 PMID: 16013362 PMID: 16005051 PMID: 15996671 PMID: 15975582 PMID: 15956822 PMID: 15945135 PMID: 15932660 PMID: 15910870 PMID: 15894964 PMID: 15886672 PMID: 15886380 PMID: 15871857 PMID: 15871849 PMID: 15840256 PMID: 15817699 PMID: 15780831 PMID: 15777535 PMID: 15766276 PMID: 15765889 PMID: 15761214 PMID: 15741053 PMID: 15721894 PMID: 15701462 PMID: 15694947 PMID: 15687142 PMID: 15678256 PMID: 15638820 PMID: 15630029 PMID: 15624283 PMID: 15618206 PMID: 15613707 PMID: 15583721 PMID: 15545514 PMID: 15539625 PMID: 15497766 PMID: 15485593 PMID: 15485592 PMID: 15485589 PMID: 15475832 PMID: 15466657 PMID: 15466638 PMID: 15455656 PMID: 15367173 PMID: 15330276 PMID: 15321802 PMID: 15313105 PMID: 15309461 PMID: 15258898 PMID: 15250255 PMID: 15217794 PMID: 15201617 PMID: 15189133 PMID: 15173404 PMID: 15168866 PMID: 15159035 PMID: 15151487 PMID: 15135261 PMID: 15115150 PMID: 15114276 PMID: 15093994 PMID: 15074010 PMID: 15052493 PMID: 15043986 PMID: 15030794 PMID: 15020512 PMID: 15001601 PMID: 14985171 PMID: 14767868 PMID: 14757688 PMID: 14748760 PMID: 14717057 PMID: 14717056 PMID: 14686661 PMID: 14659769 PMID: 14638540 PMID: 14634861 PMID: 14624957 PMID: 14600078 PMID: 14526661 PMID: 14520026 PMID: 12951363 PMID: 12924178 PMID: 12915330 PMID: 12888880 PMID: 12871402 PMID: 12855934 PMID: 12831960 PMID: 12780345 PMID: 12741431 PMID: 12737709 PMID: 12706135 PMID: 12702924 PMID: 12679222 PMID: 12645358 PMID: 12640509 PMID: 12600850 PMID: 12566962 PMID: 12566135 PMID: 12558058 PMID: 12544660 PMID: 12480795 PMID: 12476930 PMID: 12467937 PMID: 12449140 PMID: 12436761 PMID: 12410168 PMID: 12392964 PMID: 12371132 PMID: 12357135 PMID: 12227720 PMID: 12227101 PMID: 12207834 PMID: 12205049 PMID: 12204809 PMID: 12204798 PMID: 12090904 PMID: 12062374 PMID: 12052487 PMID: 11983840 PMID: 11979514 PMID: 11964125 PMID: 11960511 PMID: 11907637 PMID: 11900720 PMID: 11884287 PMID: 11844650 PMID: 11838331 PMID: 11827357 PMID: 11809774 PMID: 11801864 PMID: 11800413 PMID: 11742886 PMID: 11739012 PMID: 11708310 PMID: 11703959 PMID: 11695251 PMID: 11600487 PMID: 11582840 PMID: 11575709 PMID: 11533270 PMID: 11527668 PMID: 11522678 PMID: 11487306 PMID: 11483633 PMID: 11477165 PMID: 11465346 PMID: 11451720 PMID: 11397700 PMID: 11368292 PMID: 11367649 PMID: 11347136 PMID: 11333842 PMID: 11327332 PMID: 11306529 PMID: 11248083 PMID: 11242460 PMID: 11242446 PMID: 11217160 PMID: 11204580 PMID: 11208362 PMID: 11176197 PMID: 11171288 PMID: 11168298 PMID: 11165551 PMID: 11153776 PMID: 11150405 PMID: 11139933 PMID: 11133172 PMID: 11108899 PMID: 11085829 PMID: 11071065 PMID: 11004608 PMID: 11003013 PMID: 10974057 PMID: 10958817 PMID: 10956277 PMID: 10951510 PMID: 10946040 PMID: 10946007 PMID: 10884297 PMID: 10852422 PMID: 10842898 PMID: 10831526 PMID: 10813375 PMID: 10766373 PMID: 10700443 PMID: 10694780 PMID: 10629436 PMID: 10617977 PMID: 10617967 PMID: 10521370 PMID: 10517280 PMID: 10511296 PMID: 10508209 PMID: 10479232 PMID: 10471132 PMID: 10419173 PMID: 10400167 PMID: 10397685 PMID: 10395621 PMID: 10385604 PMID: 10381298 PMID: 10379026 PMID: 10377074 PMID: 10370875 PMID: 10353467 PMID: 10329979 PMID: 10218735 PMID: 10195943 PMID: 10195933 PMID: 10048598 PMID: 9950258 PMID: 9891844 PMID: 9885775 PMID: 9833077 PMID: 9800499 PMID: 9763864 PMID: 9758203 PMID: 9707524 PMID: 9701685 PMID: 9654398 PMID: 9641692 PMID: 9566646 PMID: 9534853 PMID: 9531913 PMID: 9527242 PMID: 9504146 PMID: 9478045 PMID: 9437192 PMID: 9409237 PMID: 9411311 PMID: 9409315 PMID: 9360638 PMID: 9322583 PMID: 9322581 PMID: 9202059 PMID: 9247889 PMID: 9194537 PMID: 9219083 PMID: 9175175 PMID: 9160173 PMID: 9135961 PMID: 9237021 PMID: 9229200 PMID: 9164704 PMID: 9051723 PMID: 9051202 PMID: 9601814 PMID: 9436526 PMID: 9038989 PMID: 8974213 PMID: 8993942 PMID: 8970764 PMID: 8910153 PMID: 8879440 PMID: 8822972 PMID: 8931120 PMID: 8896288 PMID: 8829088 PMID: 8759943 PMID: 8674891 PMID: 8679686 PMID: 8865141 PMID: 8735779 PMID: 8615347 PMID: 8739249 PMID: 8814965 PMID: 8613621 PMID: 8729141 PMID: 8650428 PMID: 8613538 PMID: 8926047 PMID: 8850177 PMID: 8567056 PMID: 8552614 PMID: 8979167 PMID: 8869357 PMID: 7489230 PMID: 8573981 PMID: 7677772 PMID: 8546752 PMID: 7635967 PMID: 7582995 PMID: 12049995 PMID: 8668647 PMID: 7598068 PMID: 7792898 PMID: 7773728 PMID: 7773725 PMID: 7648006 PMID: 7644560 PMID: 7479524 PMID: 7715350 PMID: 7702027 PMID: 7554275 PMID: 7850950 PMID: 7740525 PMID: 7840069 PMID: 7839038 PMID: 8685239 PMID: 7889892 PMID: 8052156 PMID: 8044917 PMID: 12436512 PMID: 8026041 PMID: 8006280 PMID: 7939368 PMID: 7911870 PMID: 8017464 PMID: 8201271 PMID: 8181117 PMID: 8179036 PMID: 7915035 PMID: 8024656 PMID: 8018102 PMID: 8109545 PMID: 8180744 PMID: 8191431 PMID: 7526576 PMID: 8165608 PMID: 8150445 PMID: 8268279 PMID: 8155941 PMID: 8140252 PMID: 7691912 PMID: 8480678 PMID: 8473644 PMID: 8348568 PMID: 8261211 PMID: 8094827 PMID: 8458587 PMID: 8458316 PMID: 8385810 PMID: 8461061 PMID: 8425302 PMID: 8502934 PMID: 8394402 PMID: 8084118 PMID: 1361345 PMID: 1290166 PMID: 1289094 PMID: 1510005 PMID: 1433870 PMID: 1395197 PMID: 1285701 PMID: 1506600 PMID: 1328748 PMID: 1632870 PMID: 1417409 PMID: 1411254 PMID: 1588945 PMID: 1588937 PMID: 1315789 PMID: 1539506 PMID: 1385908 PMID: 1632299 PMID: 1632287 PMID: 1449823 PMID: 1345319 PMID: 1294273 PMID: 1282975 PMID: 1959184 PMID: 1773702 PMID: 1683609 PMID: 1683608 PMID: 1745658 PMID: 1959473 PMID: 1867523 PMID: 2043019 PMID: 2040090 PMID: 2007712 PMID: 1831755 PMID: 1831753 PMID: 1824165 PMID: 1998738 PMID: 2064637 PMID: 1844445 PMID: 1776338 PMID: 2288764 PMID: 2088802 PMID: 2088801 PMID: 1965841 PMID: 2293451 PMID: 2290264 PMID: 2079610 PMID: 2255135 PMID: 2142635 PMID: 2086017 PMID: 2273617 PMID: 2117082 PMID: 2399269 PMID: 2321506 PMID: 2112389 PMID: 2108576 PMID: 2288513 PMID: 2139247 PMID: 2137044 PMID: 2621777 PMID: 2533414 PMID: 2679949 PMID: 2615160 PMID: 2512411 PMID: 2765262 PMID: 2542914 PMID: 2736000 PMID: 2564887 PMID: 2557813 PMID: 2525784 PMID: 2807954 PMID: 2730548 PMID: 2650696 PMID: 2650689 PMID: 2536273 PMID: 2506314 PMID: 3185285 PMID: 3231661 PMID: 3200117 PMID: 3054198 PMID: 3367809 PMID: 3289863 PMID: 3365124 PMID: 3276185 PMID: 3248116 PMID: 3332709 PMID: 3314460 PMID: 3655160 PMID: 3322532 PMID: 3301249 PMID: 2958954 PMID: 3552319 PMID: 3578111 PMID: 3552954 PMID: 3308356 PMID: 3035353 PMID: 2837698 PMID: 2952824 PMID: 3300984 PMID: 3111691 PMID: 3094541 PMID: 3748103 PMID: 3556124 PMID: 3530946 PMID: 3529901 PMID: 2945211 PMID: 3018642 PMID: 3016061 PMID: 3087455 PMID: 3705551 PMID: 2867579 PMID: 2878721 PMID: 3903564 PMID: 2866439 PMID: 3859999 PMID: 3903733 PMID: 3158183 PMID: 3989392 PMID: 4050552 PMID: 3907292 PMID: 3160477 PMID: 2983646 PMID: 2936148 PMID: 6499557 PMID: 6391133 PMID: 6488509 PMID: 6239599 PMID: 6237807 PMID: 6146032 PMID: 6230918 PMID: 6528704 PMID: 6364710 PMID: 6151892 PMID: 6147300 PMID: 6418338 PMID: 6344604 PMID: 6341645 PMID: 6833660 PMID: 6339591 PMID: 6839220 PMID: 6301667 PMID: 6681709 PMID: 6415673 PMID: 6221546 PMID: 6763197 PMID: 6216027 PMID: 6753382 PMID: 6212161 PMID: 6753154 PMID: 7040169 PMID: 7101234 PMID: 6289429 PMID: 7064831 PMID: 7200779 PMID: 7184495 PMID: 7161140 PMID: 6808619 PMID: 6758512 PMID: 6298152 PMID: 7032286 PMID: 7290132 PMID: 7036228 PMID: 7282515 PMID: 6455912 PMID: 7023225 PMID: 6110816 PMID: 6257988 PMID: 7020866 PMID: 7004161 PMID: 7465462 PMID: 7465459 PMID: 6244016 PMID: 6101858 PMID: 7423053 PMID: 7372200 PMID: 6935944 PMID: 495536 PMID: 479483 PMID: 87859 PMID: 373280 PMID: 725852 PMID: 630315 PMID: 349533 PMID: 742286 PMID: 848431 PMID: 858463 PMID: 1208984 PMID: 4852757 PMID: 4600584 PMID: 4588183 PMID: 4131581 PMID: 4754673 PMID: 4574831 PMID: 4598067 PMID: 4582913 PMID: 4116551 PMID: 4679714 PMID: 4556706 |
| Bladder cancer | PMID: 19498075 PMID: 19210552 PMID: 16951539 PMID: 16562715 PMID: 8612012 PMID: 8035710 PMID: 8208183 PMID: 1428484 PMID: 1750134 PMID: 2849478 PMID: 3823802 PMID: 6720695 |
| Calcium signaling pathway |  |
| Cardiac muscle contraction | PMID: 10422459 |
| Cell adhesion molecules (CAMs) | PMID: 16095049 PMID: 14967815 PMID: 12448198 PMID: 12020626 PMID: 10699426 PMID: 10412822 |
| Cell cycle | PMID: 20078384 PMID: 20070102 PMID: 19667240 PMID: 19502560 PMID: 19059213 PMID: 18927507 PMID: 18838155 PMID: 18719369 PMID: 18436806 PMID: 18390905 PMID: 18298949 PMID: 18243794 PMID: 18156140 PMID: 18004118 PMID: 17297752 PMID: 16895790 PMID: 19804184 PMID: 16617118 PMID: 16564529 PMID: 16555682 PMID: 16498512 PMID: 16288760 PMID: 16115478 PMID: 15665795 PMID: 15629664 PMID: 15481997 PMID: 15320856 PMID: 15320515 PMID: 15231505 PMID: 15221349 PMID: 15205608 PMID: 15180582 PMID: 15163286 PMID: 15054589 PMID: 14727979 PMID: 14726409 PMID: 14678872 PMID: 14615024 PMID: 14593697 PMID: 14501029 PMID: 12942161 PMID: 12847566 PMID: 12742492 PMID: 12624599 PMID: 12522126 PMID: 12482833 PMID: 12324707 PMID: 12151851 PMID: 12148909 PMID: 12131023 PMID: 12109864 PMID: 12048257 PMID: 12039437 PMID: 12002259 PMID: 11795828 PMID: 11686669 PMID: 11514367 PMID: 11201022 PMID: 11139796 PMID: 11082394 PMID: 11044429 PMID: 10974414 PMID: 10865827 PMID: 10809377 PMID: 10683356 PMID: 10571534 PMID: 10406694 PMID: 10363980 PMID: 10205288 PMID: 9827474 PMID: 9826574 PMID: 9647865 PMID: 9546379 PMID: 9375940 PMID: 9256282 PMID: 7947601 PMID: 8301982 PMID: 3057291 |
| Chemokine signaling pathway |  |
| Chronic myeloid leukemia | PMID: 12893021 PMID: 12436292 |
| Citrate cycle (TCA cycle) | PMID: 18626887 PMID: 11753387 PMID: 8023775 |
| Colorectal cancer | PMID: 20335629 PMID: 19857096 PMID: 19809413 PMID: 19707368 PMID: 19640966 PMID: 19468079 PMID: 19460658 PMID: 19384117 PMID: 19321569 PMID: 19258560 PMID: 19237618 PMID: 19050901 PMID: 19007655 PMID: 19000846 PMID: 18833143 PMID: 18788234 PMID: 18533043 PMID: 18504222 PMID: 18396599 PMID: 18348708 PMID: 18319414 PMID: 18252376 PMID: 18179501 PMID: 18053789 PMID: 17972534 PMID: 17882666 PMID: 17573984 PMID: 17347166 PMID: 17186167 PMID: 17060812 PMID: 16949142 PMID: 16886920 PMID: 16855537 PMID: 16750963 PMID: 16388273 PMID: 16262965 PMID: 15882164 PMID: 15817610 PMID: 15811887 PMID: 15249239 PMID: 15231013 PMID: 15167223 PMID: 15134159 PMID: 15118656 PMID: 15082697 PMID: 14685710 PMID: 14626652 PMID: 14624210 PMID: 12943477 PMID: 12819476 PMID: 12711997 PMID: 12695385 PMID: 12674437 PMID: 12664738 PMID: 12561598 PMID: 12557810 PMID: 12354487 PMID: 12186605 PMID: 12117397 PMID: 12076884 PMID: 12033527 PMID: 11408761 PMID: 11349515 PMID: 11304472 PMID: 11245405 PMID: 11103100 PMID: 11035446 PMID: 10646399 PMID: 10334659 PMID: 10199684 PMID: 9796631 PMID: 9761783 PMID: 9753019 PMID: 9492970 PMID: 9484426 PMID: 9283847 PMID: 9246838 PMID: 7634982 PMID: 7954294 PMID: 1406836 PMID: 1503913 PMID: 1592529 PMID: 1463976 PMID: 2114200 PMID: 3938834 PMID: 7102582 |
| Complement and coagulation cascades | PMID: 17332889 |
| Cytokine-cytokine receptor interaction |  |
| Cytosolic DNA-sensing pathway |  |
| Dilated cardiomyopathy | PMID: 20339977 PMID: 20199784 PMID: 20198391 PMID: 20186041 PMID: 20179174 PMID: 20129288 PMID: 20089485 PMID: 19950031 PMID: 19944560 PMID: 19854581 PMID: 19828226 PMID: 19808593 PMID: 19808587 PMID: 19783174 PMID: 19746241 PMID: 19734366 PMID: 19649680 PMID: 19639398 PMID: 19636323 PMID: 19604708 PMID: 19587062 PMID: 19552673 PMID: 19546096 PMID: 19514417 PMID: 19472864 PMID: 19448117 PMID: 19421837 PMID: 19417864 PMID: 19398083 PMID: 19396409 PMID: 19370326 PMID: 19340357 PMID: 19330818 PMID: 19250080 PMID: 19245969 PMID: 19245968 PMID: 19210213 PMID: 19166400 PMID: 19108909 PMID: 19085806 PMID: 19039552 PMID: 19026858 PMID: 19022000 PMID: 18928484 PMID: 18842162 PMID: 18820780 PMID: 18813769 PMID: 18803575 PMID: 18781471 PMID: 18774012 PMID: 18763005 PMID: 18721528 PMID: 18686210 PMID: 18663296 PMID: 18649821 PMID: 18628775 PMID: 18612489 PMID: 18580063 PMID: 18579481 PMID: 18539042 PMID: 18538192 PMID: 18537059 PMID: 18431525 PMID: 18418235 PMID: 18388033 PMID: 18385120 PMID: 18381865 PMID: 18370550 PMID: 18342686 PMID: 18322666 PMID: 18312905 PMID: 18306724 PMID: 18300519 PMID: 18286426 PMID: 18279769 PMID: 18279400 PMID: 18243370 PMID: 18191240 PMID: 18181953 PMID: 18172038 PMID: 19708444 PMID: 17966446 PMID: 17965495 PMID: 17938613 PMID: 17938040 PMID: 17926899 PMID: 17922122 PMID: 17911073 PMID: 17891435 PMID: 17885521 PMID: 17882369 PMID: 17875959 PMID: 17851087 PMID: 17765611 PMID: 17725758 PMID: 17721178 PMID: 17685188 PMID: 17653517 PMID: 17643880 PMID: 17635409 PMID: 17622376 PMID: 17597032 PMID: 17587592 PMID: 17579251 PMID: 17519866 PMID: 17512362 PMID: 17512361 PMID: 17493477 PMID: 17478458 PMID: 17478157 PMID: 17476783 PMID: 17447196 PMID: 17435813 PMID: 17399820 PMID: 17395321 PMID: 17373337 PMID: 17342631 PMID: 17339570 PMID: 17338743 PMID: 17288765 PMID: 17274894 PMID: 17262771 PMID: 17261407 PMID: 17217374 PMID: 17203440 PMID: 17187505 PMID: 17096077 PMID: 17027277 PMID: 17027099 PMID: 16987628 PMID: 16979011 PMID: 16964715 PMID: 16943890 PMID: 16939836 PMID: 16846419 PMID: 16816436 PMID: 16816434 PMID: 16788329 PMID: 16760230 PMID: 16755860 PMID: 16753336 PMID: 16739395 PMID: 16714771 PMID: 16714611 PMID: 16703221 PMID: 16673855 PMID: 16635611 PMID: 16626545 PMID: 16616020 PMID: 16585666 PMID: 16507617 PMID: 16456237 PMID: 16444925 PMID: 16418801 PMID: 16418253 PMID: 16401987 PMID: 16392237 PMID: 16387626 PMID: 16362731 PMID: 16353443 PMID: 16344225 PMID: 16312078 PMID: 16305631 PMID: 16301340 PMID: 16297777 PMID: 16288714 PMID: 16283135 PMID: 16274778 PMID: 16270223 PMID: 16255752 PMID: 16250184 PMID: 16216854 PMID: 16169330 PMID: 16160899 PMID: 16138880 PMID: 16103242 PMID: 16084277 PMID: 16013516 PMID: 15999470 PMID: 15917275 PMID: 15896609 PMID: 15888841 PMID: 15875511 PMID: 15847029 PMID: 15840906 PMID: 15824870 PMID: 15773420 PMID: 15755465 PMID: 15747157 PMID: 15734620 PMID: 15724661 PMID: 15724418 PMID: 15724139 PMID: 15701463 PMID: 15701462 PMID: 15701461 PMID: 15682796 PMID: 15654193 PMID: 15653387 PMID: 15642568 PMID: 15642290 PMID: 15621576 PMID: 15610259 PMID: 15553353 PMID: 15547652 PMID: 15519267 PMID: 15518629 PMID: 15487277 PMID: 15477114 PMID: 15474696 PMID: 15462399 PMID: 15461721 PMID: 15459471 PMID: 15368805 PMID: 15366427 PMID: 15358027 PMID: 15344685 PMID: 15329269 PMID: 15324610 PMID: 15311867 PMID: 15305960 PMID: 15302010 PMID: 15285780 PMID: 15254003 PMID: 15243848 PMID: 15238827 PMID: 15238823 PMID: 15219501 PMID: 15217598 PMID: 15215807 PMID: 15193822 PMID: 15185922 PMID: 15173778 PMID: 15159038 PMID: 15135700 PMID: 15120806 PMID: 15120249 PMID: 15093997 PMID: 15093994 PMID: 15084369 PMID: 15080581 PMID: 15068139 PMID: 15054924 PMID: 15024298 PMID: 15002071 PMID: 14996481 PMID: 14963612 PMID: 14768491 PMID: 14768490 PMID: 14764166 PMID: 14754423 PMID: 14750751 PMID: 14740179 PMID: 14699692 PMID: 14692298 PMID: 14666002 PMID: 14659774 PMID: 14631136 PMID: 14626736 PMID: 14625178 PMID: 14609607 PMID: 14602526 PMID: 14596664 PMID: 14566295 PMID: 14564332 PMID: 12975411 PMID: 12975406 PMID: 12939221 PMID: 12928018 PMID: 12914775 PMID: 12908069 PMID: 12891196 PMID: 12888880 PMID: 12875710 PMID: 12870530 PMID: 12821550 PMID: 12808483 PMID: 12798818 PMID: 12798578 PMID: 12741076 PMID: 12732279 PMID: 12713679 PMID: 12710322 PMID: 12687807 PMID: 12669991 PMID: 12638321 PMID: 12632908 PMID: 12590266 PMID: 12589583 PMID: 12570954 PMID: 12556677 PMID: 12555494 PMID: 12532541 PMID: 12516240 PMID: 12494058 PMID: 12487632 PMID: 12477325 PMID: 12473555 PMID: 12463096 PMID: 12445534 PMID: 12440520 PMID: 12438831 PMID: 12397571 PMID: 12390712 PMID: 12381651 PMID: 12231086 PMID: 12228791 PMID: 12221062 PMID: 12219695 PMID: 12210635 PMID: 12135243 PMID: 12132290 PMID: 12103265 PMID: 12102259 PMID: 12101796 PMID: 12086208 PMID: 12045384 PMID: 12031727 PMID: 12028602 PMID: 12025385 PMID: 12025384 PMID: 12025382 PMID: 11923809 PMID: 11858111 PMID: 11812665 PMID: 11812662 PMID: 11807744 PMID: 11797156 PMID: 11780428 PMID: 11777084 PMID: 11748102 PMID: 11741364 PMID: 11731693 PMID: 11721723 PMID: 11696268 PMID: 11689722 PMID: 11686913 PMID: 11685168 PMID: 11602813 PMID: 11593633 PMID: 11559683 PMID: 11521129 PMID: 11512689 PMID: 11491204 PMID: 11486251 PMID: 11467456 PMID: 11444506 PMID: 11431676 PMID: 11426756 PMID: 11391955 PMID: 11391040 PMID: 11377350 PMID: 11349745 PMID: 11338455 PMID: 11334658 PMID: 11333048 PMID: 11315607 PMID: 11265710 PMID: 11257767 PMID: 11214269 PMID: 11210005 PMID: 11196071 PMID: 11174343 PMID: 11144091 PMID: 11139962 PMID: 11084116 PMID: 11084103 PMID: 11077687 PMID: 11070757 PMID: 11068713 PMID: 11056772 PMID: 11025895 PMID: 11021272 PMID: 11008081 PMID: 10989746 PMID: 10983679 PMID: 10967584 PMID: 10967272 PMID: 10962124 PMID: 10953555 PMID: 10948781 PMID: 10935670 PMID: 10921786 PMID: 10909222 PMID: 10894901 PMID: 10875031 PMID: 10859992 PMID: 10838246 PMID: 10832561 PMID: 10830523 PMID: 10809200 PMID: 10806016 PMID: 10736279 PMID: 10728413 PMID: 10728394 PMID: 10630765 PMID: 11102996 PMID: 10614147 PMID: 10563156 PMID: 10568670 PMID: 10567307 PMID: 10551705 PMID: 10549834 PMID: 10543313 PMID: 10536679 PMID: 10523882 PMID: 10506392 PMID: 10464508 PMID: 10462467 PMID: 10456194 PMID: 10452346 PMID: 10452302 PMID: 10451233 PMID: 10449695 PMID: 10440148 PMID: 10426839 PMID: 10424207 PMID: 10423663 PMID: 10420902 PMID: 10420871 PMID: 10413749 PMID: 10410811 PMID: 10404349 PMID: 10377308 PMID: 10374351 PMID: 10348957 PMID: 10348551 PMID: 10230469 PMID: 10226898 PMID: 10220102 PMID: 10209002 PMID: 10208057 PMID: 10101550 PMID: 10077512 PMID: 10065024 PMID: 9990633 PMID: 9919772 PMID: 9892325 PMID: 9891947 PMID: 9891814 PMID: 9880221 PMID: 9861588 PMID: 9853179 PMID: 9842409 PMID: 9834862 PMID: 9833127 PMID: 9825363 PMID: 9822874 PMID: 9809922 PMID: 9786493 PMID: 9778329 PMID: 9771014 PMID: 9761087 PMID: 9740481 PMID: 9740478 PMID: 9731695 PMID: 9727680 PMID: 9711886 PMID: 9665032 PMID: 9642933 PMID: 9594533 PMID: 9578350 PMID: 9515022 PMID: 9570428 PMID: 9566065 PMID: 9514458 PMID: 9505952 PMID: 9488209 PMID: 9456191 PMID: 9445262 PMID: 9410774 PMID: 9358513 PMID: 9352972 PMID: 9313628 PMID: 9256850 PMID: 9226290 PMID: 11174962 PMID: 9259727 PMID: 9234054 PMID: 9183622 PMID: 9296828 PMID: 9290571 PMID: 9164884 PMID: 9152657 PMID: 9137215 PMID: 9129847 PMID: 9152354 PMID: 9254126 PMID: 9106431 PMID: 19495677 PMID: 9125676 PMID: 9139344 PMID: 10352469 PMID: 9489129 PMID: 9476539 PMID: 9336066 PMID: 9202849 PMID: 9121977 PMID: 8981204 PMID: 8960434 PMID: 9239870 PMID: 9064205 PMID: 9064204 PMID: 9019638 PMID: 10785734 PMID: 8922258 PMID: 8914701 PMID: 8803447 PMID: 8646991 PMID: 8796365 PMID: 8774633 PMID: 8737107 PMID: 8723595 PMID: 8610701 PMID: 8674103 PMID: 8814971 PMID: 8668603 PMID: 8634168 PMID: 8785402 PMID: 8734177 PMID: 8645475 PMID: 8855424 PMID: 8819711 PMID: 8554020 PMID: 8553030 PMID: 8881857 PMID: 8554365 PMID: 8552521 PMID: 8552518 PMID: 7482651 PMID: 7502570 PMID: 7648661 PMID: 7545768 PMID: 7502496 PMID: 8523436 PMID: 7674149 PMID: 7631612 PMID: 7618621 PMID: 7571768 PMID: 7595125 PMID: 7653086 PMID: 7788033 PMID: 10150403 PMID: 7731882 PMID: 7696026 PMID: 8751254 PMID: 8588380 PMID: 7863713 PMID: 7724391 PMID: 7724390 PMID: 7860011 PMID: 7722349 PMID: 7713106 PMID: 7713099 PMID: 7977122 PMID: 7930257 PMID: 7865511 PMID: 7797212 PMID: 7930227 PMID: 8062418 PMID: 7786119 PMID: 8000618 PMID: 7995268 PMID: 7988601 PMID: 7971396 PMID: 8020325 PMID: 7956986 PMID: 8205656 PMID: 8184813 PMID: 7730876 PMID: 8116990 PMID: 8147306 PMID: 8106700 PMID: 8113538 PMID: 8049092 PMID: 7842264 PMID: 8306847 PMID: 8150936 PMID: 8134287 PMID: 8127811 PMID: 8281642 PMID: 8149190 PMID: 8067013 PMID: 7856285 PMID: 7523060 PMID: 8131760 PMID: 10146527 PMID: 8305244 PMID: 8234776 PMID: 8163103 PMID: 8122866 PMID: 8413390 PMID: 8213549 PMID: 8213548 PMID: 8415326 PMID: 8294137 PMID: 8286132 PMID: 8213510 PMID: 8362719 PMID: 8233996 PMID: 7922220 PMID: 8394643 PMID: 8269315 PMID: 8517381 PMID: 8336663 PMID: 8269297 PMID: 8480587 PMID: 8328177 PMID: 7685883 PMID: 8462136 PMID: 8185728 PMID: 8458353 PMID: 8438714 PMID: 8472386 PMID: 8427136 PMID: 7680456 PMID: 8455337 PMID: 8442375 PMID: 8418631 PMID: 8417548 PMID: 8417063 PMID: 8417050 PMID: 8309177 PMID: 8298322 PMID: 8261482 PMID: 8231681 PMID: 8212708 PMID: 8145420 PMID: 8046594 PMID: 8009043 PMID: 1492439 PMID: 1451253 PMID: 1430681 PMID: 1359212 PMID: 1478217 PMID: 1442504 PMID: 1442493 PMID: 1401613 PMID: 1395210 PMID: 1325108 PMID: 1529176 PMID: 1402168 PMID: 1509128 PMID: 1487882 PMID: 1418109 PMID: 1341146 PMID: 1523653 PMID: 1598871 PMID: 1639337 PMID: 1352191 PMID: 1618198 PMID: 1615730 PMID: 1615729 PMID: 1615727 PMID: 1564223 PMID: 1640665 PMID: 1595301 PMID: 1588519 PMID: 1556585 PMID: 1405233 PMID: 1375371 PMID: 1571168 PMID: 1611179 PMID: 1536115 PMID: 1423423 PMID: 1405223 PMID: 1372727 PMID: 1311139 PMID: 1736616 PMID: 1527934 PMID: 1618239 PMID: 1523558 PMID: 1482310 PMID: 1326177 PMID: 1960307 PMID: 1816433 PMID: 1764590 PMID: 1726180 PMID: 1687118 PMID: 1957143 PMID: 1943242 PMID: 1753016 PMID: 1683146 PMID: 1683143 PMID: 1839090 PMID: 1870649 PMID: 1877457 PMID: 1836511 PMID: 1824197 PMID: 1916622 PMID: 1861916 PMID: 1858676 PMID: 1791707 PMID: 1832984 PMID: 1711061 PMID: 1676239 PMID: 1886476 PMID: 1769026 PMID: 2021115 PMID: 2049889 PMID: 1872007 PMID: 1854772 PMID: 1854762 PMID: 1901438 PMID: 2065687 PMID: 2036061 PMID: 1856903 PMID: 1672482 PMID: 1894120 PMID: 1711740 PMID: 1704826 PMID: 1706841 PMID: 1991893 PMID: 2015167 PMID: 2007168 PMID: 1985381 PMID: 1960969 PMID: 1947816 PMID: 1878080 PMID: 1843520 PMID: 1843519 PMID: 1833894 PMID: 1711971 PMID: 2244566 PMID: 2097387 PMID: 2097386 PMID: 2095127 PMID: 2089158 PMID: 1704610 PMID: 2104430 PMID: 2272703 PMID: 2226286 PMID: 2152244 PMID: 2087037 PMID: 2121015 PMID: 2220581 PMID: 2171312 PMID: 2095438 PMID: 2247980 PMID: 2290080 PMID: 2284756 PMID: 2261291 PMID: 2218208 PMID: 2145109 PMID: 2360521 PMID: 2360495 PMID: 2232469 PMID: 2232465 PMID: 2172613 PMID: 2191578 PMID: 2188768 PMID: 2160775 PMID: 1974100 PMID: 2144067 PMID: 2330870 PMID: 2188895 PMID: 2316465 PMID: 2312033 PMID: 1690167 PMID: 2296893 PMID: 11527116 PMID: 11527109 PMID: 2406361 PMID: 2368762 PMID: 2344226 PMID: 2339587 PMID: 2274878 PMID: 2274876 PMID: 2084916 PMID: 2688983 PMID: 2623916 PMID: 2598430 PMID: 2596579 PMID: 2572666 PMID: 2519797 PMID: 2615165 PMID: 2615143 PMID: 2782251 PMID: 2672767 PMID: 2768720 PMID: 2573326 PMID: 2800665 PMID: 2756871 PMID: 2754136 PMID: 2811044 PMID: 2693881 PMID: 2662734 PMID: 2627436 PMID: 2597609 PMID: 2698711 PMID: 2541334 PMID: 2705827 PMID: 2659182 PMID: 2648793 PMID: 2754912 PMID: 2705634 PMID: 2927794 PMID: 2919561 PMID: 2733332 PMID: 2465681 PMID: 2650869 PMID: 2646761 PMID: 2702015 PMID: 2652897 PMID: 2642367 PMID: 2534047 PMID: 2530974 PMID: 2521415 PMID: 2511682 PMID: 2480485 PMID: 3195428 PMID: 3230783 PMID: 3183060 PMID: 2463585 PMID: 2463576 PMID: 3177199 PMID: 2852276 PMID: 2970786 PMID: 3249281 PMID: 3044639 PMID: 3392336 PMID: 2456168 PMID: 3405780 PMID: 3287888 PMID: 3265217 PMID: 3136064 PMID: 3284737 PMID: 3163814 PMID: 3392876 PMID: 3354409 PMID: 3376474 PMID: 3356077 PMID: 3043926 PMID: 3223643 PMID: 3071100 PMID: 3071099 PMID: 2975926 PMID: 3326293 PMID: 3680786 PMID: 3673909 PMID: 3437666 PMID: 3665320 PMID: 3622270 PMID: 2956872 PMID: 3673160 PMID: 3655598 PMID: 3496001 PMID: 3594942 PMID: 3569924 PMID: 3825947 PMID: 3609034 PMID: 3548306 PMID: 3807720 PMID: 3493962 PMID: 3629487 PMID: 3622558 PMID: 3437485 PMID: 3324528 PMID: 3302186 PMID: 2959263 PMID: 2949575 PMID: 3776843 PMID: 2437339 PMID: 2876635 PMID: 3766598 PMID: 3751930 PMID: 3751863 PMID: 3019608 PMID: 3717032 PMID: 3521251 PMID: 2941533 PMID: 3082612 PMID: 3953392 PMID: 3948367 PMID: 3487721 PMID: 3953354 PMID: 3948548 PMID: 3705684 PMID: 3962311 PMID: 3946167 PMID: 3712036 PMID: 3798351 PMID: 3791405 PMID: 3738787 PMID: 3715729 PMID: 3099488 PMID: 3094316 PMID: 2947566 PMID: 4089581 PMID: 2932264 PMID: 4050704 PMID: 2417570 PMID: 4028536 PMID: 4025171 PMID: 4014014 PMID: 3894187 PMID: 3889105 PMID: 3874172 PMID: 2991541 PMID: 4032740 PMID: 3888631 PMID: 3973293 PMID: 3157304 PMID: 3981788 PMID: 4038571 PMID: 3922139 PMID: 3884232 PMID: 6526845 PMID: 6241903 PMID: 6240236 PMID: 6508193 PMID: 6486005 PMID: 6481009 PMID: 6475782 PMID: 6475779 PMID: 6475774 PMID: 6147879 PMID: 6492577 PMID: 6483626 PMID: 6743422 PMID: 6745290 PMID: 6731302 PMID: 6431932 PMID: 6711427 PMID: 6489021 PMID: 6702627 PMID: 6695789 PMID: 6690559 PMID: 6524455 PMID: 6523407 PMID: 6523406 PMID: 6650417 PMID: 6650406 PMID: 6360836 PMID: 6314688 PMID: 6620943 PMID: 6681931 PMID: 6889474 PMID: 7464235 |
| DNA replication | PMID: 18004118 PMID: 14764618 PMID: 12845685 PMID: 12048257 PMID: 9544743 PMID: 9552510 PMID: 1371432 |
| Drug metabolism | PMID: 19514965 PMID: 19463375 PMID: 18850180 PMID: 18805405 PMID: 16276236 PMID: 15833898 PMID: 19804164 PMID: 12938250 PMID: 12525679 PMID: 11416429 PMID: 11304891 PMID: 9241897 PMID: 8577194 PMID: 7852860 PMID: 8305778 PMID: 3437726 |
| ECM-receptor interaction |  |
| Endocytosis | PMID: 20023241 PMID: 19644050 PMID: 19635789 PMID: 18716843 PMID: 18704423 PMID: 18662803 PMID: 18212280 PMID: 17513374 PMID: 15378205 PMID: 15308601 PMID: 12553167 PMID: 12456719 PMID: 12434421 PMID: 11701468 PMID: 10338498 PMID: 10209655 PMID: 9192751 PMID: 8800494 PMID: 8767493 PMID: 1947935 PMID: 1825665 |
| Epithelial cell signaling in Helicobacter pylori infection |  |
| Ether lipid metabolism |  |
| Fc epsilon RI signaling pathway |  |
| Fc gamma R-mediated phagocytosis |  |
| Focal adhesion | PMID: 18802021 PMID: 17496332 PMID: 17306468 PMID: 14660654 |
| Fructose and mannose metabolism |  |
| Galactose metabolism |  |
| Gap junction | PMID: 18651385 PMID: 17318613 PMID: 17250811 PMID: 16646596 PMID: 15751661 PMID: 11744143 PMID: 9495300 PMID: 7627716 |
| Glutathione metabolism | PMID: 18062993 PMID: 8937270 |
| Glycerolipid metabolism |  |
| Glycerophospholipid metabolism |  |
| Glycolysis / Gluconeogenesis |  |
| Glyoxylate and dicarboxylate metabolism |  |
| GnRH signaling pathway |  |
| Hedgehog signaling pathway |  |
| Hematopoietic cell lineage |  |
| Homologous recombination |  |
| Huntington's disease | PMID: 12044443 PMID: 7765749 |
| Hypertrophic cardiomyopathy (HCM) | PMID: 20349138 PMID: 20193178 PMID: 20156645 PMID: 20132378 PMID: 20087240 PMID: 19891768 PMID: 19765841 PMID: 19527529 PMID: 19523780 PMID: 19100006 PMID: 18503222 PMID: 18384577 PMID: 18374998 PMID: 18243370 PMID: 17884073 PMID: 17653030 PMID: 17069906 PMID: 16506641 PMID: 16456237 PMID: 16442910 PMID: 16356568 PMID: 15860394 PMID: 15241541 PMID: 14967727 PMID: 14581405 PMID: 14557696 PMID: 11499819 PMID: 11214269 PMID: 10987611 PMID: 10888207 PMID: 9783650 PMID: 9416892 PMID: 9397588 PMID: 8934365 PMID: 7614488 PMID: 8131760 PMID: 8277603 PMID: 8336412 PMID: 8455337 PMID: 1837210 PMID: 1925099 PMID: 1772691 PMID: 1841923 PMID: 2615173 PMID: 3405780 PMID: 2977783 PMID: 3310637 PMID: 3622558 PMID: 3433879 PMID: 3704879 PMID: 6540479 PMID: 7175221 PMID: 7040581 PMID: 6459467 PMID: 7193139 PMID: 7190882 |
| Inositol phosphate metabolism |  |
| Insulin signaling pathway | PMID: 17437648 |
| Jak-STAT signaling pathway |  |
| Leukocyte transendothelial migration |  |
| Linoleic acid metabolism |  |
| Long-term depression |  |
| Lysine degradation |  |
| Lysosome | PMID: 18716843 PMID: 12591106 PMID: 7853716 PMID: 3199660 |
| MAPK signaling pathway | PMID: 15027449 |
| Melanogenesis |  |
| Metabolism of xenobiotics by cytochrome P450 | PMID: 18805405 PMID: 16824612 PMID: 10641128 |
| Methane metabolism |  |
| Mismatch repair |  |
| mTOR signaling pathway | PMID: 19375241 PMID: 19020099 |
| Natural killer cell mediated cytotoxicity |  |
| Neuroactive ligand-receptor interaction |  |
| Neurotrophin signaling pathway |  |
| N-Glycan biosynthesis |  |
| NOD-like receptor signaling pathway |  |
| Non-homologous end-joining |  |
| Non-small cell lung cancer | PMID: 19556815 PMID: 18650173 PMID: 18583144 PMID: 17505444 PMID: 17410071 PMID: 17239299 PMID: 16096702 PMID: 14711777 PMID: 12754038 PMID: 10369954 PMID: 9007127 PMID: 8086174 PMID: 8384493 PMID: 1328070 PMID: 2549825 |
| Notch signaling pathway | PMID: 18802018 |
| Nucleotide excision repair |  |
| One carbon pool by folate |  |
| Other glycan degradation | PMID: 19840568 PMID: 18416219 PMID: 17396063 PMID: 17070102 PMID: 17044374 PMID: 16321690 PMID: 16008544 PMID: 15928243 PMID: 15708700 PMID: 15320782 PMID: 15306215 PMID: 15287678 PMID: 15287677 PMID: 15206126 PMID: 15205701 PMID: 15142969 PMID: 14699687 PMID: 14663598 PMID: 14663597 PMID: 14505482 PMID: 12887720 PMID: 12828193 PMID: 12644346 PMID: 12618263 PMID: 12595841 PMID: 12553181 PMID: 11858183 PMID: 11827681 PMID: 11554775 PMID: 11397720 PMID: 11273849 PMID: 11254896 PMID: 11192307 PMID: 10929741 PMID: 10924726 PMID: 10877197 PMID: 10745576 PMID: 10611843 PMID: 10505540 PMID: 10471427 PMID: 10404734 PMID: 10372241 PMID: 10341391 PMID: 10198911 PMID: 9821816 PMID: 9711933 PMID: 9597405 PMID: 9409221 PMID: 9081692 PMID: 8841743 PMID: 8860712 PMID: 7585300 PMID: 8521759 PMID: 7627700 PMID: 8000623 PMID: 8199183 PMID: 8074804 PMID: 8199741 PMID: 8088176 PMID: 1424043 PMID: 1414897 PMID: 1418090 PMID: 1616906 PMID: 1313951 PMID: 1733381 PMID: 1929512 PMID: 2045376 PMID: 1794159 PMID: 2102083 PMID: 3229016 PMID: 2997433 PMID: 3993622 PMID: 6472153 PMID: 7175378 PMID: 7138283 PMID: 89993 PMID: 195760 PMID: 179743 PMID: 1208984 |
| Oxidative phosphorylation | PMID: 19488738 PMID: 19035162 PMID: 18001320 PMID: 17456993 PMID: 17378771 PMID: 16266403 PMID: 15496306 PMID: 12852781 PMID: 8950240 PMID: 1383759 PMID: 1890710 PMID: 1104262 PMID: 4636277 |
| Pancreatic cancer | PMID: 15724433 PMID: 15630853 PMID: 8529768 PMID: 7556816 PMID: 7781849 |
| Parkinson's disease | PMID: 20184681 PMID: 19888711 PMID: 19864173 PMID: 19715385 PMID: 19633703 PMID: 19381858 PMID: 18626887 PMID: 18457539 PMID: 17512599 PMID: 17420306 PMID: 17254441 PMID: 17239370 PMID: 16337092 PMID: 16078956 PMID: 16037918 PMID: 16027555 PMID: 15868996 PMID: 15814870 PMID: 14671778 PMID: 12533089 PMID: 12531940 PMID: 12375058 PMID: 12224783 PMID: 12044443 PMID: 11133172 PMID: 10842712 PMID: 10668414 PMID: 10634137 PMID: 10221467 PMID: 10086482 PMID: 10078894 PMID: 9633876 PMID: 9116179 PMID: 7481373 PMID: 8030397 PMID: 1418928 PMID: 1595102 PMID: 7384406 PMID: 1009713 |
| Pathways in cancer | PMID: 20188216 PMID: 19955787 PMID: 18537694 PMID: 18511896 PMID: 18508910 PMID: 18180097 PMID: 18030589 PMID: 17923573 PMID: 17600565 PMID: 17454207 PMID: 17257774 PMID: 16820735 PMID: 16716120 PMID: 16515479 PMID: 16194042 PMID: 16115539 PMID: 16043020 PMID: 16005051 PMID: 15968396 PMID: 15499992 PMID: 15364116 PMID: 15078140 PMID: 15077723 PMID: 12480795 PMID: 11813979 PMID: 11795383 PMID: 11747658 PMID: 11170257 PMID: 10842899 PMID: 10030691 PMID: 9855704 PMID: 9457441 PMID: 341410 |
| Pentose and glucuronate interconversions |  |
| Pentose phosphate pathway | PMID: 3248116 |
| Phenylalanine metabolism |  |
| Phosphatidylinositol signaling system |  |
| PPAR signaling pathway |  |
| Primary immunodeficiency | PMID: 18978467 PMID: 17098478 PMID: 9848383 |
| Prion diseases |  |
| Prostate cancer | PMID: 20377474 PMID: 20153946 PMID: 20141678 PMID: 20051565 PMID: 19996060 PMID: 19950034 PMID: 19913184 PMID: 19737788 PMID: 19706860 PMID: 19515794 PMID: 19233413 PMID: 19138808 PMID: 19047297 PMID: 19025432 PMID: 18947517 PMID: 18719369 PMID: 18520031 PMID: 18398147 PMID: 18243498 PMID: 17761128 PMID: 17619718 PMID: 17494461 PMID: 17411824 PMID: 17006736 PMID: 16983113 PMID: 16943535 PMID: 16915855 PMID: 16551206 PMID: 16323982 PMID: 16275241 PMID: 15937017 PMID: 15899311 PMID: 15820970 PMID: 15709885 PMID: 15648182 PMID: 15636671 PMID: 15630288 PMID: 15610866 PMID: 15570041 PMID: 15500418 PMID: 15486745 PMID: 15167319 PMID: 15146084 PMID: 15123400 PMID: 15123399 PMID: 15051847 PMID: 12667885 PMID: 12544298 PMID: 12424336 PMID: 12204553 PMID: 12076884 PMID: 12069675 PMID: 11937435 PMID: 11851107 PMID: 11476836 PMID: 11423676 PMID: 11164156 PMID: 11139004 PMID: 11018402 PMID: 10878703 PMID: 10439169 PMID: 10100412 PMID: 10069722 PMID: 9796631 PMID: 9734939 PMID: 9665108 PMID: 9521358 PMID: 9495697 PMID: 9351365 PMID: 9105984 PMID: 8627972 PMID: 8222655 PMID: 2173856 PMID: 3046303 PMID: 4062120 |
| Proteasome | PMID: 20226836 PMID: 19624571 PMID: 19282875 PMID: 19165168 PMID: 19094431 PMID: 18276919 PMID: 18157711 PMID: 17971205 PMID: 17823377 PMID: 17264164 PMID: 16254127 PMID: 16019609 PMID: 14732197 PMID: 12958191 PMID: 11397693 |
| Purine metabolism | PMID: 18585721 PMID: 16300459 PMID: 10212666 |
| Pyrimidine metabolism |  |
| Pyruvate metabolism |  |
| Regulation of actin cytoskeleton | PMID: 19307690 |
| Renal cell carcinoma | PMID: 19825494 PMID: 19513859 PMID: 19474115 PMID: 19017005 PMID: 18838713 PMID: 18436521 PMID: 18083403 PMID: 15610866 PMID: 15293584 PMID: 15006056 PMID: 11574248 PMID: 10619596 PMID: 9691592 PMID: 7618739 PMID: 1607254 PMID: 1922558 PMID: 2338330 PMID: 3491592 |
| Renin-angiotensin system | PMID: 20384386 PMID: 20359530 PMID: 20220527 PMID: 20198391 PMID: 20090429 PMID: 19922882 PMID: 19450721 PMID: 19878370 PMID: 19861349 PMID: 19811239 PMID: 19779116 PMID: 19732605 PMID: 19719332 PMID: 19707983 PMID: 19689616 PMID: 19669395 PMID: 19649581 PMID: 19648058 PMID: 19491619 PMID: 19475778 PMID: 19390704 PMID: 19390543 PMID: 19327134 PMID: 19301781 PMID: 19229817 PMID: 19144653 PMID: 19114589 PMID: 19098298 PMID: 19082699 PMID: 18996825 PMID: 18937921 PMID: 18929228 PMID: 18846354 PMID: 18832826 PMID: 18827909 PMID: 18794623 PMID: 18787943 PMID: 18724960 PMID: 18638617 PMID: 18637188 PMID: 18600300 PMID: 18595216 PMID: 18466420 PMID: 18449380 PMID: 18454336 PMID: 18413498 PMID: 18413308 PMID: 18409173 PMID: 18398395 PMID: 18344376 PMID: 18319594 PMID: 18317916 PMID: 18294050 PMID: 18288179 PMID: 18174783 PMID: 18230954 PMID: 18220697 PMID: 18220488 PMID: 18214672 PMID: 18200812 PMID: 19281913 PMID: 18095912 PMID: 18082520 PMID: 18062896 PMID: 18048949 PMID: 17969376 PMID: 19804278 PMID: 17954286 PMID: 17950785 PMID: 17903684 PMID: 17897016 PMID: 17846284 PMID: 17666197 PMID: 17761193 PMID: 17704609 PMID: 17697055 PMID: 17640474 PMID: 17606856 PMID: 17601392 PMID: 17596405 PMID: 17592090 PMID: 17579251 PMID: 17521710 PMID: 17486303 PMID: 17461301 PMID: 17429448 PMID: 17429286 PMID: 17414657 PMID: 17395678 PMID: 17327458 PMID: 17285438 PMID: 17253471 PMID: 17173262 PMID: 17131847 PMID: 17118372 PMID: 17064808 PMID: 17053529 PMID: 17013240 PMID: 17003740 PMID: 16949011 PMID: 16915040 PMID: 16845252 PMID: 16825329 PMID: 16820589 PMID: 16818827 PMID: 16702489 PMID: 16672147 PMID: 16672144 PMID: 16596809 PMID: 16572049 PMID: 16568131 PMID: 16563944 PMID: 16553505 PMID: 16464173 PMID: 16405197 PMID: 16397519 PMID: 16331093 PMID: 16306429 PMID: 16298258 PMID: 16271939 PMID: 16262560 PMID: 16250855 PMID: 16248996 PMID: 16218881 PMID: 16162605 PMID: 16146835 PMID: 16125051 PMID: 16125049 PMID: 16093452 PMID: 16088850 PMID: 16045904 PMID: 15971071 PMID: 15963208 PMID: 15907144 PMID: 15868362 PMID: 15858940 PMID: 15853700 PMID: 15834542 PMID: 15732660 PMID: 15699449 PMID: 15694745 PMID: 15676177 PMID: 15670768 PMID: 15667649 PMID: 15615794 PMID: 15580058 PMID: 15563875 PMID: 15556057 PMID: 15543356 PMID: 15540418 PMID: 15531767 PMID: 15529618 PMID: 15511408 PMID: 15505113 PMID: 15500433 PMID: 15470293 PMID: 15386943 PMID: 15326080 PMID: 15320838 PMID: 15320825 PMID: 15302839 PMID: 15261932 PMID: 15252776 PMID: 15222246 PMID: 15195836 PMID: 15193960 PMID: 15171419 PMID: 15135254 PMID: 15125489 PMID: 15016346 PMID: 14746140 PMID: 14717335 PMID: 14706665 PMID: 14689366 PMID: 14661009 PMID: 14608518 PMID: 14605589 PMID: 14602526 PMID: 14524646 PMID: 14502296 PMID: 14498755 PMID: 12975417 PMID: 12971828 PMID: 12957882 PMID: 12952843 PMID: 12938141 PMID: 12923384 PMID: 12862295 PMID: 12818730 PMID: 12796065 PMID: 12735065 PMID: 12734958 PMID: 12728597 PMID: 12724058 PMID: 12641882 PMID: 12634888 PMID: 12545338 PMID: 12426214 PMID: 12419176 PMID: 12401536 PMID: 12352014 PMID: 12228849 PMID: 12143941 PMID: 12133028 PMID: 12124994 PMID: 12090908 PMID: 11975906 PMID: 11967726 PMID: 11899582 PMID: 11875587 PMID: 11861038 PMID: 11852019 PMID: 11849656 PMID: 11840363 PMID: 11835907 PMID: 11814137 PMID: 11811371 PMID: 11800413 PMID: 11759948 PMID: 11728286 PMID: 11709400 PMID: 11696688 PMID: 11694212 PMID: 11675831 PMID: 11595564 PMID: 11575041 PMID: 11566927 PMID: 11554775 PMID: 11479463 PMID: 11454847 PMID: 11413801 PMID: 11392476 PMID: 11392475 PMID: 11349731 PMID: 11346218 PMID: 11345362 PMID: 11330506 PMID: 11317462 PMID: 11263608 PMID: 11177655 PMID: 11250978 PMID: 11243310 PMID: 11213892 PMID: 11198556 PMID: 11171786 PMID: 11134677 PMID: 11082147 PMID: 11077221 PMID: 10997795 PMID: 10981173 PMID: 10981170 PMID: 10961416 PMID: 10952419 PMID: 10889131 PMID: 10879452 PMID: 10867998 PMID: 10862638 PMID: 10852853 PMID: 10811595 PMID: 10794598 PMID: 10739375 PMID: 10736756 PMID: 10736279 PMID: 10731400 PMID: 10685727 PMID: 10652909 PMID: 10639000 PMID: 11116112 PMID: 10618582 PMID: 10545620 PMID: 10499558 PMID: 10422461 PMID: 10386272 PMID: 10365179 PMID: 10097233 PMID: 10093713 PMID: 9930381 PMID: 9894619 PMID: 9894621 PMID: 9861522 PMID: 9857879 PMID: 9833171 PMID: 9806472 PMID: 9796838 PMID: 9796837 PMID: 9773126 PMID: 9736438 PMID: 9719016 PMID: 9651738 PMID: 9649922 PMID: 9640343 PMID: 9605599 PMID: 9405164 PMID: 9426022 PMID: 9431478 PMID: 9313622 PMID: 9313606 PMID: 9235795 PMID: 9127618 PMID: 9127615 PMID: 9127614 PMID: 9105785 PMID: 9060882 PMID: 8994414 PMID: 9429844 PMID: 9397288 PMID: 9020385 PMID: 9003497 PMID: 8989733 PMID: 9115957 PMID: 9115954 PMID: 9120665 PMID: 8949314 PMID: 8925576 PMID: 8693718 PMID: 8548410 PMID: 8582695 PMID: 8583483 PMID: 7648682 PMID: 7783537 PMID: 8565712 PMID: 7697839 PMID: 7655713 PMID: 7850980 PMID: 7977014 PMID: 7713099 PMID: 7700002 PMID: 7955173 PMID: 7826551 PMID: 7987030 PMID: 7946805 PMID: 7827369 PMID: 7910095 PMID: 7856285 PMID: 7523057 PMID: 8485836 PMID: 8488853 PMID: 8498976 PMID: 7903590 PMID: 1414921 PMID: 1414920 PMID: 1414888 PMID: 1476764 PMID: 1395215 PMID: 1553691 PMID: 1440254 PMID: 1381795 PMID: 2263922 PMID: 2148785 PMID: 2118301 PMID: 2196774 PMID: 2365484 PMID: 2194807 PMID: 2250582 PMID: 2688986 PMID: 2661197 PMID: 2483242 PMID: 3314460 PMID: 2437401 PMID: 2441194 PMID: 6385698 PMID: 6148814 PMID: 6099735 PMID: 6085369 PMID: 6847008 PMID: 6185446 |
| Retinol metabolism |  |
| Ribosome |  |
| RIG-I-like receptor signaling pathway |  |
| RNA polymerase | PMID: 18309103 PMID: 10075593 |
| Small cell lung cancer | PMID: 19556815 PMID: 18650173 PMID: 18583144 PMID: 17505444 PMID: 17410071 PMID: 17239299 PMID: 17168436 PMID: 16096702 PMID: 14711777 PMID: 12754038 PMID: 10369954 PMID: 9007127 PMID: 9423097 PMID: 8086174 PMID: 8384493 PMID: 1328070 PMID: 1661205 PMID: 2549825 |
| Sphingolipid metabolism | PMID: 19784582 |
| Spliceosome | PMID: 16329997 |
| Steroid hormone biosynthesis | PMID: 3773997 |
| Systemic lupus erythematosus | PMID: 20396352 PMID: 20391484 PMID: 20335276 PMID: 20236499 PMID: 20202592 PMID: 20147475 PMID: 20132070 PMID: 20118159 PMID: 20082538 PMID: 20031679 PMID: 19955047 PMID: 19933722 PMID: 19833754 PMID: 19790130 PMID: 19762397 PMID: 19728017 PMID: 19691927 PMID: 19644909 PMID: 19578104 PMID: 19561227 PMID: 19531751 PMID: 19502228 PMID: 19500102 PMID: 19333947 PMID: 19318388 PMID: 19286698 PMID: 19276299 PMID: 19221828 PMID: 19208561 PMID: 19205552 PMID: 19196569 PMID: 19105418 PMID: 19086148 PMID: 19026147 PMID: 18976721 PMID: 18973750 PMID: 18942303 PMID: 18926165 PMID: 18852221 PMID: 18793004 PMID: 18785313 PMID: 18774002 PMID: 18719369 PMID: 18700910 PMID: 18665150 PMID: 18665148 PMID: 18662507 PMID: 18647585 PMID: 18646350 PMID: 18634156 PMID: 18625800 PMID: 18607179 PMID: 18576352 PMID: 18568176 PMID: 18528966 PMID: 18484694 PMID: 19936286 PMID: 18443545 PMID: 18391674 PMID: 18344922 PMID: 18323517 PMID: 18300571 PMID: 18260168 PMID: 18240259 PMID: 18240193 PMID: 18060447 PMID: 17959072 PMID: 17948705 PMID: 17900266 PMID: 17869565 PMID: 17762456 PMID: 17762454 PMID: 17728367 PMID: 17693444 PMID: 17650559 PMID: 17620509 PMID: 17576733 PMID: 17530717 PMID: 17454934 PMID: 17439938 PMID: 17439937 PMID: 17432112 PMID: 17426779 PMID: 17401301 PMID: 17315608 PMID: 17201275 PMID: 17183620 PMID: 17169159 PMID: 17117488 PMID: 17080910 PMID: 17043468 PMID: 17041400 PMID: 16980722 PMID: 16932628 PMID: 16909327 PMID: 16868975 PMID: 16864946 PMID: 16802358 PMID: 16782558 PMID: 16775899 PMID: 16729301 PMID: 16720898 PMID: 16565850 PMID: 16431336 PMID: 16395749 PMID: 16353073 PMID: 16344496 PMID: 16265708 PMID: 16234277 PMID: 16218467 PMID: 16175933 PMID: 16150405 PMID: 16119705 PMID: 16046220 PMID: 16021278 PMID: 15968605 PMID: 15922149 PMID: 15909739 PMID: 15909083 PMID: 15868616 PMID: 15854941 PMID: 15757967 PMID: 15693087 PMID: 15571929 PMID: 15462488 PMID: 15462483 PMID: 15384713 PMID: 15383063 PMID: 15370721 PMID: 15351310 PMID: 15316307 PMID: 15262847 PMID: 15247980 PMID: 15230141 PMID: 15150430 PMID: 15088296 PMID: 15074010 PMID: 15001976 PMID: 14995002 PMID: 14976854 PMID: 14769525 PMID: 14765038 PMID: 14726373 PMID: 14681506 PMID: 14681501 PMID: 14659847 PMID: 14613279 PMID: 14613278 PMID: 14583569 PMID: 13130471 PMID: 12960475 PMID: 12922957 PMID: 12858154 PMID: 12768666 PMID: 12734962 PMID: 12659991 PMID: 12645857 PMID: 12638899 PMID: 12612393 PMID: 12563682 PMID: 12468886 PMID: 12399879 PMID: 12364626 PMID: 12362850 PMID: 12094392 PMID: 11877022 PMID: 11842823 PMID: 11809000 PMID: 11727842 PMID: 11665973 PMID: 11600739 PMID: 11517743 PMID: 11404827 PMID: 11036832 PMID: 10943870 PMID: 10813315 PMID: 10805484 PMID: 10805483 PMID: 10805481 PMID: 10784507 PMID: 10778663 PMID: 10555892 PMID: 10544836 PMID: 10529129 PMID: 10089998 PMID: 10078014 PMID: 9974430 PMID: 9706424 PMID: 9706281 PMID: 9458619 PMID: 19078188 PMID: 9150122 PMID: 9740846 PMID: 9302660 PMID: 9256312 PMID: 8986854 PMID: 8888164 PMID: 8736620 PMID: 8634882 PMID: 9546909 PMID: 8554365 PMID: 7562794 PMID: 7791180 PMID: 7744099 PMID: 7723359 PMID: 7718428 PMID: 7647604 PMID: 7970032 PMID: 7951302 PMID: 8154514 PMID: 8159079 PMID: 8231039 PMID: 8339438 PMID: 8465737 PMID: 1442853 PMID: 1464868 PMID: 1522805 PMID: 1589650 PMID: 1735266 PMID: 1540039 PMID: 1750231 PMID: 1864145 PMID: 2214602 PMID: 2816986 PMID: 2526671 PMID: 3386889 PMID: 3425688 PMID: 3661587 PMID: 3577764 PMID: 3782887 PMID: 2436999 PMID: 4025948 PMID: 4016410 PMID: 4011845 PMID: 6465176 PMID: 6711679 PMID: 6977269 PMID: 7259828 PMID: 32614 PMID: 5070117 |
| T cell receptor signaling pathway |  |
| Taste transduction |  |
| Taurine and hypotaurine metabolism |  |
| Terpenoid backbone biosynthesis |  |
| TGF-beta signaling pathway |  |
| Thyroid cancer | PMID: 20014213 PMID: 15696350 PMID: 10646653 PMID: 8644032 PMID: 8378535 |
| Tight junction |  |
| Toll-like receptor signaling pathway | PMID: 18508689 |
| Tryptophan metabolism |  |
| Type I diabetes mellitus | PMID: 16340402 PMID: 15110601 PMID: 12433006 PMID: 10761866 PMID: 9686919 PMID: 7839327 PMID: 1442576 PMID: 1608214 PMID: 1805419 PMID: 3275682 PMID: 6521202 PMID: 6523369 |
| Type II diabetes mellitus | PMID: 20202592 PMID: 19876063 PMID: 19822102 PMID: 19685167 PMID: 19460605 PMID: 19395096 PMID: 19232230 PMID: 19067997 PMID: 18854748 PMID: 18678294 PMID: 18626311 PMID: 18273039 PMID: 18040744 PMID: 17721698 PMID: 17711711 PMID: 17704557 PMID: 17689519 PMID: 17577676 PMID: 17513208 PMID: 17318769 PMID: 17162282 PMID: 16606865 PMID: 16304314 PMID: 16236932 PMID: 16009332 PMID: 15941146 PMID: 15715433 PMID: 15669552 PMID: 15618250 PMID: 15211337 PMID: 15193683 PMID: 12444312 PMID: 12440233 PMID: 12067487 PMID: 11917198 PMID: 11893294 PMID: 11428709 PMID: 11386855 PMID: 19667562 PMID: 11374173 PMID: 11307863 PMID: 11285041 PMID: 11284486 PMID: 11256776 PMID: 11179243 PMID: 10548135 PMID: 9847683 PMID: 9274899 PMID: 9160809 PMID: 9158162 PMID: 9264821 PMID: 8993938 PMID: 8902158 PMID: 11416394 PMID: 8645373 PMID: 7573090 PMID: 7659888 PMID: 7800207 PMID: 8495624 PMID: 8477149 PMID: 1313951 PMID: 1531244 PMID: 1579896 PMID: 1284137 PMID: 1749445 PMID: 1802391 PMID: 1772997 PMID: 1950378 PMID: 2087939 PMID: 2595586 PMID: 4042800 PMID: 6521202 PMID: 6523369 PMID: 6240029 |
| Tyrosine metabolism | PMID: 10488288 PMID: 2073665 |
| Ubiquitin mediated proteolysis |  |
| Valine, leucine and isoleucine biosynthesis | PMID: 19357637 PMID: 11079831 PMID: 8497108 |
| Vascular smooth muscle contraction | PMID: 14707008 PMID: 12542447 PMID: 8477149 PMID: 7053296 |
| Vibrio cholerae infection |  |
| Wnt signaling pathway | PMID: 17622581 PMID: 17332414 |
