# Supplementary material for: Disease Gene Interaction Pathways: A Potential Framework for How Disease Genes Associate by Disease-Risk Modules
Source: PLoS One. 2011 Sep 6;6(9):e24495. doi: 10.1371/journal.pone.0024495 (PMC3167857; doi:10.1371/journal.pone.0024495)
Supplement: Table S4 — PubMed ID in which gene pairs between interacting disease-risk terms have been proved to be correlated with CAD. (DOC) [file pone.0024495.s007.doc]

**Table S4. PubMed ID in which gene pairs between interacting disease-risk terms have been proved to be correlated with CAD.**

| **interacting disease risk terms pairs** | **gene pairs between disease risk terms** | **PumMed ID that support the relationship between disease risk term pairs** |
| --- | --- | --- |
| 638 & 3866 | *PLA2G7* & *PPARA* | PMID: 19948975 |
|  | *MTR* & *AR* | PMID: 18785313 |
|  | *SHE* & *AR* | PMID: 19086148 |
|  | *MTHFR* & *AR* | PMID: 18785313 |
|  | *MTHFR* & *PPARG* | PMID: 17999084 |
| 638 & 4287 | *MTHFR* & *PLAT* | PMID: 16248996 PMID: 11963567 PMID: 11507973 |
|  | *MTHFR* & *F2* | PMID: 17683517 |
| 638 & 4945 | *MVK* & *LDLR* | PMID: 18193043 |
|  | *MTHFR* & *LRP1* | PMID: 15121769 |
|  | *CTA-216E10.6* & *APP* | PMID: 11587454 |
| 638 & 5331 | *RPE* & *HP* | PMID: 19420333 |
|  | *TAGLN* & *TNF* | PMID: 14560935 |
| 638 & 5414 |  |  |
| 638 & 2437 | *PLA2G7* & *PON1* | PMID: 19948975 |
|  | *MTHFR* & *PON1* | PMID: 19634497 PMID: 19280995 PMID: 19219535 PMID: 17683517 PMID: 16077191 |
| 638 & 6171 | *PPIC* & *HR* | PMID: 10440909 |
|  | *RPE* & *HR* | PMID: 16612447 PMID: 11775864 PMID: 8872291 PMID: 8235125 |
|  | *ALOX5AP* & *HR* | PMID: 18775537 |
|  | *AR*C & *HR* | PMID: 19812059 PMID: 18754967 PMID: 17295325 |
|  | *KY* & *HR* | PMID: 18166191 |
|  | *KIF6* & *HR* | PMID: 18222354 |
|  | *SHE* & *HR* | PMID: 19213377 |
|  | *MTHFR* & *HR* | PMID: 15301885 PMID: 12927690 PMID: 10982535 |
|  | *CTA-216E10.6* & *HR* | PMID: 20202518 PMID: 19269915 PMID: 18691213 PMID: 17385719 PMID: 16625106 |
| 638 & 6126 | *ABCB1* & *CETP* | PMID: 19558216 |
|  | *MVK* & *LPL* | PMID: 18193043 |
|  | *MVK* & *CETP* | PMID: 18193043 |
|  | *KY* & *CETP* | PMID: 15882786 |
|  | *MTHFR* & *LPL* | PMID: 18704761 PMID: 17403027 |
|  | *MTHFR* & *CETP* | PMID: 17403027 PMID: 16248996 |
| 638 & 6320 | *GPX1* & *BCHE* | PMID: 17275003 |
| 638 & 5975 | *AIRE* & *ACE* | PMID: 7965277 PMID: 8076659 |
|  | *KY* & *ACE* | PMID: 17011901 |
|  | *SHE* & *ACE* | PMID: 11109414 |
|  | *MTHFR* & *ACE* | PMID: 19634497 PMID: 19280995 PMID: 19043368 PMID: 17683517 PMID: 17403027 PMID: 16928730 PMID: 16248996 PMID: 15595935 PMID: 15151261 PMID: 15121769 PMID: 12833177 PMID: 12186157 PMID: 11963567 PMID: 11507973 PMID: 9105559 |
|  | SMS & *ACE* | PMID: 18291721 |
| 638 & 4958 | *MVK* & *APOE* | PMID: 18193043 |
|  | *GPX1* & *APOE* | PMID: 17255533 |
|  | *KY* & *LIF* | PMID: 8960523 |
|  | *SHE* & *APOE* | PMID: 15191354 |
|  | *MTHFR* & *APOE* | PMID: 19148342 PMID: 18704761 PMID: 17683517 PMID: 17403027 PMID: 15284679 PMID: 15121769 PMID: 9568733 |
|  | LSR & *APOE* | PMID: 18374151 |
| 638 & 6433 | *RPE* & *DBP* | PMID: 11775864 PMID: 8872291 |
|  | *RPE* & *SCARB1* | PMID: 19420333 |
|  | *MVK* & *APOA1* | PMID: 18193043 |
|  | *DDAH2* & *CYBA* | PMID: 16705470 |
|  | *MTHFR* & *APOA1* | PMID: 11963567 PMID: 11507973 |
|  | *TMEM57* & *CTCF* | PMID: 19060911 |
| 638 & 6116 | *PLA2G7* & *IL1R2* | PMID: 19948975 |
| 3866 & 3903 | *PPARA* & *INSR* | PMID: 19948975 |
| 3866 & 5033 | *ESR1* & *ESR2* | PMID: 19938352 PMID: 16099331 |
|  | *PPARA* & *ESR2* | PMID: 19948975 |
|  | *PPARA* & *SREBF2* | PMID: 16763159 |
| 3866 & 5414 |  |  |
| 3866 & 4944 |  |  |
| 3866 & 5982 | *AR* & *IL6* | PMID: 19877084 |
| 3866 & 5978 | *AR* & *CBS* | PMID: 18785313 |
| 3866 & 5949 | *PPARA* & *ABCA1* | PMID: 18219093 PMID: 17608096 PMID: 16763159 |
| 3866 & 2437 | *AR* & *PON1* | PMID: 17900266 PMID: 17473052 PMID: 16729301 PMID: 16683402 |
|  | *PPARA* & *PON1* | PMID: 19948975 PMID: 18219093 |
| 3866 & 5115 |  |  |
| 3866 & 6171 | *AR* & *HR* | PMID: 19344498 PMID: 17305646 PMID: 16877533 PMID: 15797659 PMID: 15751097 PMID: 15693010 PMID: 12804926 PMID: 7482735 PMID: 8154998 PMID: 3094316 PMID: 7195240 |
|  | *ESR1* & *HR* | PMID: 20153472 |
|  | *PPARA* & *PPARD* | PMID: 15128052 |
|  | *PPARG* & *PPARD* | PMID: 15128052 |
|  | *PPARG* & *HR* | PMID: 18561518 |
|  | RXRB & *PPARD* | PMID: 19935834 |
| 3866 & 5975 | *AR* & *ACE* | PMID: 19825824 PMID: 11684837 PMID: 9826315 PMID: 7712932 |
|  | *VDR* & *ACE* | PMID: 11716989 |
|  | *PPARA* & *ACE* | PMID: 18787507 |
| 3866 & 4958 | *AR* & *APOE* | PMID: 17947799 PMID: 15451791 |
|  | *PPARA* & *APOE* | PMID: 18219093 PMID: 16763159 PMID: 16043164 |
| 3866 & 6404 |  |  |
| 3866 & 6433 | *AR* & *PSD* | PMID: 1453779 PMID: 1515514 |
|  | *AR* & *APOA1* | PMID: 17473052 |
|  | *PPARA* & *APOA1* | PMID: 16763159 |
| 3866 & 6116 | *PPARA* & *IL1R2* | PMID: 19948975 |
| 3866 & 6410 |  |  |
| 3903 & 5414 |  |  |
| 3903 & 4944 |  |  |
| 3903 & 5982 | *IRS1* & *IL6* | PMID: 20112301 |
|  | *IRS2* & *IL6* | PMID: 20112301 |
| 3903 & 4224 | *INSR* & *AGTR1* | PMID: 19948975 |
| 3903 & 2305 |  |  |
| 3903 & 5822 |  |  |
| 3903 & 5931 | *IRS1* & *NOS3* | PMID: 18436806 |
| 4287 & 4945 | *PLAT* & *LDLR* | PMID: 18162663 PMID: 16957178 |
|  | *F2* & *LDLR* | PMID: 14557872 |
|  | *THBS1* & *LDLR* | PMID: 14557872 |
|  | *MMP2* & *LDLR* | PMID: 19940766 |
| 4287 & 5219 | *COL3A1* & *TIMP3* | PMID: 15905966 |
|  | *PLG* & *SERPINE1* | PMID: 16706973 |
|  | *F2* & *SERPINE1* | PMID: 17683517 |
| 4287 & 3853 | *MMP2* & *MMP1* | PMID: 18035073 PMID: 17893005 PMID: 16122719 |
|  | *MMP2* & *MMP7* | PMID: 16122719 |
|  | *MMP2* & *MMP3* | PMID: 19576586 PMID: 18035073 PMID: 16122719 |
|  | *MMP2* & *MMP9* | PMID: 18035073 PMID: 16122719 PMID: 12454321 |
|  | *MMP1*3 & *MMP1* | PMID: 16122719 |
|  | *MMP1*3 & *MMP7* | PMID: 16122719 |
|  | *MMP1*3 & *MMP3* | PMID: 16122719 |
|  | *MMP1*3 & *MMP9* | PMID: 16122719 |
| 4287 & 4393 | *F2* & *SELP* | PMID: 14557872 |
|  | *THBS1* & *SELP* | PMID: 14557872 |
| 4287 & 4944 | *F2* & *F12* | PMID: 19415820 |
| 4287 & 2437 | *COL3A1* & *PDCD10* | PMID: 15905966 |
|  | *F2* & *PON1* | PMID: 17683517 PMID: 17185369 |
| 4287 & 3895 | *MMP2* & *MMP1*2 | PMID: 16122719 |
|  | *MMP1*3 & *MMP1*2 | PMID: 16122719 |
| 4287 & 4878 | *PLG* & *LPA* | PMID: 18612205 |
|  | *PLAT* & *CCL2* | PMID: 16329464 |
|  | *F2* & *F5* | PMID: 19415820 PMID: 17683517 |
|  | *F2* & *F7* | PMID: 19415820 |
|  | *F2* & *CCL2* | PMID: 19628666 |
|  | *MMP2* & *MMP1*0 | PMID: 18035073 |
| 4287 & 5316 | *PLAT* & *AGT* | PMID: 19779330 PMID: 11963567 PMID: 11507973 |
|  | *F2* & *AGT* | PMID: 17683517 |
|  | *F2* & *PRCP* | PMID: 14557872 |
|  | *THBS1* & *PRCP* | PMID: 14557872 |
| 4287 & 5011 | *COL1A1* & *LRP5* | PMID: 15811432 |
| 4287 & 5699 | *PLAT* & *CRP* | PMID: 16329464 |
|  | *F2* & *CRP* | PMID: 18638594 PMID: 17991651 PMID: 17145556 PMID: 12611642 |
|  | *MMP2* & *CRP* | PMID: 18346322 PMID: 18035073 PMID: 12454321 |
| 4287 & 6320 |  |  |
| 4287 & 4958 | *F2* & *APOE* | PMID: 17901562 PMID: 17683517 PMID: 16028714 PMID: 11809774 |
|  | *MMP2* & *APOE* | PMID: 19762778 |
| 4287 & 3847 |  |  |
| 4287 & 2305 |  |  |
| 4287 & 6248 | *MMP2* & *CAMP* | PMID: 17893005 |
| 4287 & 5822 | *F2* & *SELE* | PMID: 14557872 |
|  | *THBS1* & *SELE* | PMID: 14557872 |
| 4287 & 6404 |  |  |
| 4287 & 4305 | *THBS1* & *VEGFC* | PMID: 18035072 |
| 4287 & 6433 | *PLAT* & *APOA1* | PMID: 11963567 PMID: 11507973 |
| 4287 & 6454 |  |  |
| 4287 & 6410 | *THBS1* & *HIF1A* | PMID: 18035072 |
| 4945 & 5219 | *LRP1* & *THBS2* | PMID: 15121769 |
|  | *LDLR* & *LCN2* | PMID: 16254208 |
|  | *LDLR* & *CD14* | PMID: 18067591 |
| 4945 & 4393 | *LRP1* & *SELP* | PMID: 15121769 |
|  | *LDLR* & *SELP* | PMID: 14557872 |
|  | *LDLR* & *HR*G | PMID: 10699424 PMID: 10633291 |
| 4945 & 4944 |  |  |
| 4945 & 5982 | *LDLR* & *IL6* | PMID: 14557872 |
| 4945 & 2437 | *LDLR* & *PON1* | PMID: 11230170 |
| 4945 & 3895 | *APP* & *VWF* | PMID: 15691624 |
|  | *LRP1* & *GP1BA* | PMID: 15121769 |
|  | *LDLR* & *APOH* | PMID: 12740481 |
| 4945 & 4878 | *LRP1* & *F7* | PMID: 15121769 |
|  | *LRP1* & *FGB* | PMID: 15121769 |
|  | *LDLR* & *LPA* | PMID: 9691807 PMID: 2203216 |
|  | *LDLR* & *F7* | PMID: 19336475 |
|  | *LDLR* & *FGB* | PMID: 16776623 |
|  | *LDLR* & *CCL2* | PMID: 16880330 |
| 4945 & 5011 |  |  |
| 4945 & 6126 | *LDLR* & *LPL* | PMID: 19336475 PMID: 18193043 PMID: 16776623 PMID: 16477380 PMID: 11230170 PMID: 9799092 |
|  | *LDLR* & *CETP* | PMID: 19336475 PMID: 18193043 PMID: 9691807 PMID: 9683614 PMID: 2203216 |
| 4945 & 6240 | *LDLR* & *LIPC* | PMID: 18193043 PMID: 14557872 PMID: 9878681 |
|  | *LDLR* & *MTTP* | PMID: 18369455 |
|  | *LDLR* & *APOB* | PMID: 20217239 PMID: 20019594 PMID: 19827648 PMID: 19336475 PMID: 19319977 PMID: 19041195 PMID: 18566665 PMID: 18279815 PMID: 18193043 PMID: 17964958 PMID: 17955342 PMID: 17765246 PMID: 17380167 PMID: 17277381 PMID: 17274457 PMID: 17142622 PMID: 17094996 PMID: 16477380 PMID: 16389549 PMID: 16159606 PMID: 16015283 PMID: 15523646 PMID: 15321838 PMID: 15321837 PMID: 15030301 PMID: 12730697 PMID: 12705331 PMID: 12421096 PMID: 11230170 PMID: 10669643 PMID: 10208479 PMID: 9878681 PMID: 9799092 PMID: 9691807 PMID: 9409302 PMID: 8645375 PMID: 2203216 |
|  | *LDLR* & *TG* | PMID: 20217239 PMID: 19766896 PMID: 16477380 PMID: 15144588 |
|  | V*LDLR* & *APOB* | PMID: 8879439 |
|  | V*LDLR* & *TG* | PMID: 8879439 |
| 4945 & 6320 |  |  |
| 4945 & 4958 | *LRP1* & *APOE* | PMID: 20360257 PMID: 15121769 |
|  | *LDLR* & *APOE* | PMID: 20360257 PMID: 20197419 PMID: 19336475 PMID: 18566665 PMID: 18193043 PMID: 18067591 PMID: 17894024 PMID: 17174298 PMID: 16954559 PMID: 16678832 PMID: 16514080 PMID: 16254208 PMID: 16015283 PMID: 15946668 PMID: 15804943 PMID: 15780204 PMID: 15144588 PMID: 15031124 PMID: 14642405 PMID: 12969990 PMID: 12626663 PMID: 12475897 PMID: 11592107 PMID: 11308177 PMID: 11230170 PMID: 10208479 PMID: 9878681 PMID: 9799092 PMID: 9691807 PMID: 9409302 PMID: 8645375 PMID: 2203216 |
|  | V*LDLR* & *APOE* | PMID: 8879439 |
| 4945 & 6433 | *LDLR* & *APOA1* | PMID: 18193043 PMID: 17924833 PMID: 11230170 PMID: 9878681 |
| 4945 & 6410 |  |  |
| 5219 & 3853 |  |  |
| 5219 & 5316 | *HGF* & *AGT* | PMID: 12124994 |
|  | *SERPINE1* & *AGT* | PMID: 17683517 PMID: 12124994 |
| 5219 & 6240 | *THBS2* & *TG* | PMID: 19631562 |
|  | *SERPINE1* & *HSF1* | PMID: 17259369 |
|  | *SERPINE1* & *APOB* | PMID: 17403027 |
| 5219 & 6320 |  |  |
| 5219 & 4305 | *CD14* & *CD36* | PMID: 19751271 PMID: 18067591 PMID: 17458880 |
| 5219 & 6433 |  |  |
| 5219 & 6454 | *CD14* & *CD47* | PMID: 17458880 |
| 5033 & 5331 | *SREBF1* & *TNF* | PMID: 14560935 |
|  | *RELA* & *TNF* | PMID: 18178561 |
| 5033 & 5982 |  |  |
| 5033 & 6171 |  |  |
| 5033 & 5975 |  |  |
| 5033 & 6404 |  |  |
| 5033 & 6116 | *ESR2* & *IL1R2* | PMID: 19948975 |
| 5033 & 6410 | *RELA* & *TNFRSF10A* | PMID: 18178561 |
| 3853 & 4393 |  |  |
| 3853 & 3895 | *MMP1* & *MMP1*2 | PMID: 16122719 |
|  | *MMP7* & *MMP1*2 | PMID: 16122719 |
|  | *MMP3* & *MMP1*2 | PMID: 16122719 |
|  | *MMP9* & *MMP1*2 | PMID: 16122719 |
|  | *TFPI* & *VWF* | PMID: 19171469 PMID: 15023872 |
| 3853 & 4878 | *MMP1* & *MMP1*0 | PMID: 18035073 |
|  | *MMP3* & *MMP1*0 | PMID: 18035073 |
|  | *MMP9* & *MMP1*0 | PMID: 18035073 |
| 5331 & 4393 |  |  |
| 5331 & 4842 |  |  |
| 5331 & 2437 | *TNF* & *PON1* | PMID: 17001213 |
| 5331 & 4878 | *CSF1* & *CCL2* | PMID: 17374712 |
|  | *TNF* & *CCL2* | PMID: 18619594 PMID: 17592553 PMID: 17388104 PMID: 16678847 PMID: 16297799 PMID: 16244112 PMID: 15876998 PMID: 15552272 PMID: 15364863 PMID: 15219635 PMID: 15136050 PMID: 14581396 PMID: 12923954 PMID: 12720581 PMID: 12237173 PMID: 12204776 PMID: 10569653 |
| 5331 & 6248 | *TNF* & *IL1B* | PMID: 20112301 |
|  | *TNF* & *CAMP* | PMID: 15753227 PMID: 12873549 PMID: 10982546 PMID: 10027806 |
| 5331 & 6454 |  |  |
| 5414 & 5978 |  |  |
| 5414 & 5115 |  |  |
| 5414 & 5699 |  |  |
| 5414 & 6240 |  |  |
| 5414 & 5975 |  |  |
| 4393 & 4944 |  |  |
| 4393 & 5982 | *SELP* & *IL6* | PMID: 14557872 |
| 4393 & 3895 | *SELP* & *GP1BA* | PMID: 15121769 |
| 4393 & 3847 |  |  |
| 4393 & 5822 | *SELP* & *SELE* | PMID: 14557872 |
| 4393 & 4305 | *HR*G & *CYR61* | PMID: 16155362 |
| 4944 & 5982 |  |  |
| 4944 & 3895 |  |  |
| 4944 & 5316 |  |  |
| 4944 & 4958 | *IL6*R & *APOE* | PMID: 19567438 |
| 5982 & 6171 | *IL6* & *HR* | PMID: 19349293 PMID: 18556001 |
|  | *AGA* & *HR* | PMID: 19213377 |
| 5982 & 5975 | *IL6* & *ACE* | PMID: 17823365 |
| 5982 & 6404 |  |  |
| 5978 & 5949 |  |  |
| 5978 & 6121 |  |  |
| 5978 & 6126 |  |  |
| 5978 & 5975 | *CBS* & *ACE* | PMID: 12186157 |
| 5978 & 6404 |  |  |
| 5949 & 2437 | *ABCA1* & *PON1* | PMID: 18219093 |
| 5949 & 6171 | *ABCA1* & *HR* | PMID: 16230498 |
| 5949 & 6126 | *ABCA1* & *LPL* | PMID: 19099922 PMID: 18193043 PMID: 16763159 PMID: 16086925 PMID: 15657615 PMID: 15135251 PMID: 11434189 |
|  | *ABCA1* & *CETP* | PMID: 19099922 PMID: 18193043 PMID: 16763159 PMID: 16596814 PMID: 16086925 PMID: 15767853 PMID: 12840658 PMID: 11434189 |
| 5949 & 5699 |  |  |
| 5949 & 5975 |  |  |
| 5949 & 6248 |  |  |
| 5949 & 5931 |  |  |
| 5949 & 6433 | *ABCA1* & *APOA1* | PMID: 20075335 PMID: 19644050 PMID: 19304576 PMID: 18193043 PMID: 17113061 PMID: 16763159 PMID: 15657615 PMID: 15177121 PMID: 14962947 PMID: 12841490 PMID: 12840658 PMID: 12709788 PMID: 11434189 PMID: 11349008 |
|  | *ABCA1* & *SCARB1* | PMID: 20075335 PMID: 19644050 PMID: 16596814 PMID: 15891392 PMID: 12840658 |
| 4842 & 5975 | *SCNN1A* & *ACE* | PMID: 15699455 |
|  | *SCD* & *ACE* | PMID: 18003665 PMID: 16480770 PMID: 15021998 PMID: 10459474 |
|  | *CYP1A2* & *COMT* | PMID: 19999796 |
| 4842 & 5931 | *SCNN1A* & *NOS3* | PMID: 15699455 |
| 6121 & 2437 |  |  |
| 6121 & 6171 |  |  |
| 6121 & 6126 |  |  |
| 6121 & 5699 | *RPS7* & *CRP* | PMID: 17592552 |
| 6121 & 6404 |  |  |
| 6121 & 6433 |  |  |
| 6121 & 6410 |  |  |
| 2437 & 6171 | *PON1* & *HR* | PMID: 18164014 PMID: 14636952 |
| 2437 & 5699 | *PON1* & *CRP* | PMID: 19269283 PMID: 16140307 PMID: 12957728 |
| 2437 & 5975 | *PON1* & *CPM* | PMID: 11230170 |
|  | *PON1* & *ACE* | PMID: 19634497 PMID: 19579917 PMID: 19280995 PMID: 17683517 |
| 2437 & 6433 | *PON1* & *APOA1* | PMID: 19201174 PMID: 17543199 PMID: 17525004 PMID: 17473052 PMID: 16319130 PMID: 11230170 |
|  | *PON1* & *SCARB1* | PMID: 15681296 |
| 2437 & 6454 |  |  |
| 5115 & 3895 | *ADAMTS13* & *VWF* | PMID: 20135067 PMID: 19969076 PMID: 19439298 PMID: 19085767 PMID: 18603284 PMID: 12670342 PMID: 11672586 |
| 6171 & 3847 | *HR* & *FGG* | PMID: 11007996 |
| 6171 & 4078 | *HR* & *MPO* | PMID: 19761988 |
| 3895 & 4878 | *GP1BA* & *F7* | PMID: 15121769 |
|  | *GP1BA* & *FGB* | PMID: 15121769 |
|  | *VWF* & *CCL2* | PMID: 16102437 |
| 3895 & 6116 |  |  |
| 4878 & 6240 | *LPA* & *APOB* | PMID: 19880117 PMID: 16572113 PMID: 15690311 PMID: 11730824 PMID: 10883517 PMID: 10412822 PMID: 9691807 PMID: 9108785 PMID: 8728325 PMID: 7981178 PMID: 8241092 PMID: 2203216 |
|  | *LPA* & *TG* | PMID: 18046597 PMID: 15460421 PMID: 12370857 PMID: 12364554 PMID: 12204809 PMID: 8017467 |
|  | *F7* & *APOB* | PMID: 19336475 |
| 4878 & 3847 | *FGB* & FGA | PMID: 20167083 |
|  | *FGB* & *FGG* | PMID: 20167083 |
|  | *CCL2* & *ICAM1* | PMID: 19506932 |
|  | *CCL2* & *ICAM3* | PMID: 17263916 |
| 4878 & 2305 | *CCL2* & *CXCR4* | PMID: 12093767 |
|  | *CCL2* & *CCR5* | PMID: 17426779 PMID: 16055130 PMID: 12618265 PMID: 11500196 |
| 4878 & 2923 | *CCL2* & *CCR2* | PMID: 20181074 PMID: 19506371 PMID: 18617279 PMID: 17426779 PMID: 16445900 PMID: 16055130 PMID: 12870487 PMID: 12618265 PMID: 12411463 PMID: 12239249 PMID: 11755917 PMID: 11500196 |
| 5316 & 4224 | *AGT* & *ADRB2* | PMID: 15699455 |
|  | *AGT* & *AGTR1* | PMID: 19779330 |
| 5316 & 6320 |  |  |
| 5316 & 3847 |  |  |
| 5316 & 3622 |  |  |
| 6126 & 5931 | *LPL* & *NOS3* | PMID: 17403027 |
|  | *CETP* & *NOS3* | PMID: 19691831 PMID: 17403027 |
| 6126 & 6433 | *LPL* & *DBP* | PMID: 15253101 |
|  | *LPL* & *APOA1* | PMID: 18415697 PMID: 18193043 PMID: 16938803 PMID: 16763159 PMID: 15657615 PMID: 11434189 PMID: 11230170 PMID: 10889803 |
|  | *CETP* & *LCT* | PMID: 14531818 |
|  | *CETP* & *APOA1* | PMID: 18193043 PMID: 17266548 PMID: 16763159 PMID: 12840658 PMID: 11730826 PMID: 11434189 PMID: 10889803 PMID: 10523010 |
|  | *CETP* & *SCARB1* | PMID: 16596814 PMID: 16542392 PMID: 14719972 PMID: 12840658 PMID: 11575708 PMID: 11111095 |
| 4224 & 5240 | *ADRB2* & *ADRB3* | PMID: 15917856 |
|  | *AGTR1* & *ADRB3* | PMID: 15193960 |
| 4224 & 5975 | *ADRB2* & *ACE* | PMID: 15699455 |
|  | *AGTR1* & *ACE* | PMID: 18787507 PMID: 12975417 PMID: 11696688 PMID: 9456365 |
| 4224 & 6248 |  |  |
| 4224 & 5931 | *ADRB2* & *NOS3* | PMID: 15699455 |
| 5240 & 5975 |  |  |
| 5240 & 5931 |  |  |
| 5240 & 6433 |  |  |
| 5699 & 6240 | *MX1* & *MTTP* | PMID: 18369455 |
|  | *CRP* & *APOB* | PMID: 20223535 PMID: 20019460 PMID: 19959168 PMID: 19800772 PMID: 19642911 PMID: 19573556 PMID: 19476111 PMID: 19424216 PMID: 19336475 PMID: 19269283 PMID: 17825806 PMID: 16963013 PMID: 15571826 PMID: 15557708 PMID: 14979683 PMID: 14968563 PMID: 9241743 |
|  | *CRP* & *TG* | PMID: 20229495 PMID: 20134372 PMID: 20095516 PMID: 20084446 PMID: 20002542 PMID: 19959168 PMID: 19953954 PMID: 19938052 PMID: 19728847 PMID: 19642911 PMID: 19606245 PMID: 19593954 PMID: 19593950 PMID: 19576019 PMID: 19548440 PMID: 19476111 PMID: 19284399 PMID: 19254210 PMID: 19228823 PMID: 19187587 PMID: 19167589 PMID: 18677173 PMID: 18396656 PMID: 18260956 PMID: 18260902 PMID: 17485889 PMID: 17433279 PMID: 17066589 PMID: 16787648 PMID: 16472797 PMID: 16171015 PMID: 16113803 PMID: 16047464 PMID: 15840256 PMID: 15777554 PMID: 15653117 PMID: 15557708 PMID: 15526541 PMID: 15505088 PMID: 15366636 PMID: 15313947 PMID: 14979683 PMID: 14579918 PMID: 12839659 PMID: 12773138 PMID: 10780315 |
| 5699 & 6320 |  |  |
| 5699 & 4305 |  |  |
| 6240 & 6433 | *LIPC* & *APOA1* | PMID: 18193043 PMID: 15657615 PMID: 9878681 |
|  | *LIPC* & *SCARB1* | PMID: 16397139 |
|  | *APOB* & *MVD* | PMID: 18609106 |
|  | *APOB* & *APOA1* | PMID: 19648057 PMID: 19476111 PMID: 18801202 PMID: 18640459 PMID: 18476573 PMID: 18356785 PMID: 18333887 PMID: 18193043 PMID: 18066136 PMID: 17985658 PMID: 17588826 PMID: 17543199 PMID: 17525004 PMID: 17319473 PMID: 16763159 PMID: 16682089 PMID: 16487434 PMID: 16208299 PMID: 16195894 PMID: 15815801 PMID: 15364185 PMID: 15250255 PMID: 14675566 PMID: 12778627 PMID: 12709788 PMID: 11902853 PMID: 11434189 PMID: 11367649 PMID: 11230170 PMID: 11122500 PMID: 10889803 PMID: 10523010 PMID: 9974428 PMID: 9878681 PMID: 9365344 PMID: 9241743 PMID: 7500552 PMID: 7671890 PMID: 7569454 PMID: 7695175 PMID: 8354314 PMID: 1289018 PMID: 1562163 PMID: 2395263 PMID: 3608115 |
|  | *APOB* & *SCARB1* | PMID: 11575708 |
|  | *APOB* & *SST* | PMID: 11500189 |
|  | *TG* & *DBP* | PMID: 20146671 PMID: 19209619 PMID: 18269824 PMID: 18206038 PMID: 18045723 PMID: 16213608 PMID: 15730804 PMID: 15223224 PMID: 12860491 PMID: 12406036 PMID: 12355867 PMID: 12225719 PMID: 11194020 PMID: 10830174 PMID: 10331294 PMID: 9764445 PMID: 9591740 PMID: 8729141 PMID: 7780864 PMID: 7807497 PMID: 7805525 PMID: 2383317 PMID: 2737688 |
|  | *TG* & *ALB* | PMID: 18688083 |
|  | *TG* & *APOA1* | PMID: 19732897 PMID: 19476111 PMID: 18801202 PMID: 18415697 PMID: 17588826 PMID: 16763159 PMID: 16208299 PMID: 16125709 PMID: 15657615 PMID: 15460421 PMID: 15250255 PMID: 14675566 PMID: 11367649 PMID: 1562163 |
|  | *TG* & *SST* | PMID: 18655280 PMID: 15715433 PMID: 12052247 PMID: 11500189 |
|  | *TG* & *SAC* | PMID: 2879769 |
| 6320 & 6433 |  |  |
| 5975 & 6433 | *CPM* & *APOA1* | PMID: 11230170 |
|  | *ACE* & *CYBA* | PMID: 15193812 PMID: 10488959 |
|  | *ACE* & *DBP* | PMID: 20334448 PMID: 18269824 PMID: 16220750 PMID: 12297007 PMID: 12117083 PMID: 11840224 PMID: 10652909 PMID: 9591757 PMID: 2514265 |
|  | *ACE* & *APOA1* | PMID: 11963567 PMID: 11507973 |
| 4958 & 6433 | *APOE* & *HFE* | PMID: 12850485 PMID: 12714262 |
|  | *APOE* & *APOA1* | PMID: 19644050 PMID: 18398670 PMID: 18193043 PMID: 18063807 PMID: 17991756 PMID: 17985658 PMID: 16763159 PMID: 15657615 PMID: 15177121 PMID: 12023827 PMID: 11434189 PMID: 11230170 PMID: 11055796 PMID: 10889803 PMID: 9974428 PMID: 9878681 PMID: 8354314 |
|  | *APOE* & *SCARB1* | PMID: 19644050 PMID: 18054357 PMID: 16397139 PMID: 15967843 PMID: 15692099 PMID: 11861414 |
| 3847 & 6433 | *ICAM1* & *CYBA* | PMID: 19564823 |
| 2305 & 2923 | *CXCR4* & *CCR2* | PMID: 16456237 |
|  | *CCR5* & *CCR2* | PMID: 20182805 PMID: 17426779 PMID: 17383752 PMID: 16480760 PMID: 16055130 PMID: 15017357 PMID: 14578618 PMID: 12742282 PMID: 12618265 PMID: 11500196 |
| 2305 & 6433 |  |  |
| 6248 & 6433 | SOD3 & *CYBA* | PMID: 16705470 |
|  | *IL1B* & *MVD* | PMID: 19142372 |
|  | *CAMP* & *MVD* | PMID: 10027806 |
| 6248 & 6454 |  |  |
| 6248 & 6116 |  |  |
| 5822 & 6116 | *SELE* & *IL1R1* | PMID: 14557872 |
| 5822 & 6410 |  |  |
| 6404 & 6433 |  |  |
| 6404 & 4078 |  |  |
| 4305 & 6454 | *CD36* & *CD47* | PMID: 17458880 |
| 6433 & 6454 |  |  |
| 6454 & 6116 |  |  |
