# Supplementary material for: Disease Gene Interaction Pathways: A Potential Framework for How Disease Genes Associate by Disease-Risk Modules
Source: PLoS One. 2011 Sep 6;6(9):e24495. doi: 10.1371/journal.pone.0024495 (PMC3167857; doi:10.1371/journal.pone.0024495)
Supplement: Table S5 — PubMed ID in which KEGG pathways enriched have been proved to be correlated with HT. (DOC) [file pone.0024495.s008.doc]

**Table S5. PubMed ID in which KEGG pathways enriched have been proved to be correlated with HT.**

| **pathway** | **Pubmed ID associated with HT** |
| --- | --- |
| ABC transporters | PMID: 21118087 PMID: 20222053 PMID: 14678230 |
| Acute myeloid leukemia | PMID: 20699277 PMID: 19674789 PMID: 19264722 PMID: 17668866 PMID: 17094046 PMID: 16617323 PMID: 16332390 PMID: 15861341 PMID: 15710268 PMID: 15459012 PMID: 15160962 PMID: 11685098 PMID: 10064182 PMID: 9219799 PMID: 8689355 PMID: 7595745 PMID: 7549797 PMID: 7872398 |
| Adherens junction | PMID: 20175114 PMID: 12053015 |
| Adipocytokine signaling pathway | PMID: 17615379 PMID: 17402563 |
| Aldosterone-regulated sodium reabsorption | PMID: 16116042 PMID: 12468577 |
| Allograft rejection | PMID: 20941744 PMID: 20688351 PMID: 20649510 PMID: 20130772 PMID: 20013274 PMID: 19958327 PMID: 19807050 PMID: 19782585 PMID: 19770719 PMID: 19623017 PMID: 19519358 PMID: 19370615 PMID: 19134091 PMID: 19129260 PMID: 19091785 PMID: 19060534 PMID: 19023530 PMID: 18835781 PMID: 18828116 PMID: 18710663 PMID: 18671680 PMID: 18405244 PMID: 18396011 PMID: 18078388 PMID: 18220719 PMID: 18202507 PMID: 18190712 PMID: 18087711 PMID: 18063119 PMID: 17982037 PMID: 17874514 PMID: 17850443 PMID: 17442224 PMID: 17299656 PMID: 17137409 PMID: 16899521 PMID: 16638355 PMID: 16408127 PMID: 16162211 PMID: 16075952 PMID: 15989680 PMID: 15954891 PMID: 15919458 PMID: 15892197 PMID: 15888709 PMID: 15703421 PMID: 15691225 PMID: 15669207 PMID: 15621081 PMID: 15569301 PMID: 15385638 PMID: 15378803 PMID: 15344956 PMID: 15304499 PMID: 15052212 PMID: 15041343 PMID: 15041303 PMID: 14702541 PMID: 14692809 PMID: 14593182 PMID: 14523805 PMID: 14508357 PMID: 12829899 PMID: 12799196 PMID: 12790696 PMID: 12756387 PMID: 12750764 PMID: 12694071 PMID: 12614289 PMID: 12490790 PMID: 12221903 PMID: 11805539 PMID: 11805185 PMID: 11801887 PMID: 11743851 PMID: 11590850 PMID: 11579301 PMID: 11558679 PMID: 11461948 PMID: 11420061 PMID: 11413896 PMID: 11310347 PMID: 11266841 PMID: 10755558 PMID: 11074219 PMID: 10995970 PMID: 10977786 PMID: 10859717 PMID: 10855741 PMID: 10782729 PMID: 10720632 PMID: 10711524 PMID: 10676738 PMID: 10620545 PMID: 10589950 PMID: 10589699 PMID: 10499377 PMID: 10454772 PMID: 10410574 PMID: 10343253 PMID: 10196038 PMID: 10092995 PMID: 10051050 PMID: 9934810 PMID: 9844127 PMID: 9760814 PMID: 9736138 PMID: 9734624 PMID: 9684074 PMID: 9588587 PMID: 9531939 PMID: 9531679 PMID: 9477210 PMID: 9407426 PMID: 9323275 PMID: 9298043 PMID: 9292559 PMID: 18020502 PMID: 9257256 PMID: 9158023 PMID: 9183823 PMID: 9138457 PMID: 8956848 PMID: 8625664 PMID: 8545866 PMID: 8588226 PMID: 8587280 PMID: 8719451 PMID: 8589282 PMID: 7655850 PMID: 7743805 PMID: 8587392 PMID: 8572388 PMID: 7539270 PMID: 7882637 PMID: 8088774 PMID: 8079974 PMID: 7997722 PMID: 7916934 PMID: 8147620 PMID: 8193289 PMID: 8109582 PMID: 11271341 PMID: 8179808 PMID: 10148400 PMID: 8425695 PMID: 8232106 PMID: 1477317 PMID: 1412763 PMID: 1405664 PMID: 1434917 PMID: 1410862 PMID: 1733091 PMID: 1728711 PMID: 1449829 PMID: 1748236 PMID: 1873018 PMID: 2058832 PMID: 1842547 PMID: 1723464 PMID: 2234717 PMID: 2400654 PMID: 2319369 PMID: 2361019 PMID: 3291321 PMID: 3287917 PMID: 3540834 PMID: 3528537 PMID: 3511315 PMID: 3916515 PMID: 3925760 PMID: 3886231 PMID: 6402622 PMID: 87551 PMID: 782401 PMID: 1108314 PMID: 1094575 PMID: 1095976 PMID: 1105556 PMID: 4601075 PMID: 4919685 PMID: 4944225 PMID: 4301781 |
| Alzheimer's disease | PMID: 21196259 PMID: 21188026 PMID: 21174483 PMID: 21170472 PMID: 21150913 PMID: 21143566 PMID: 21130746 PMID: 21129453 PMID: 21091952 PMID: 21094551 PMID: 21046450 PMID: 21044775 PMID: 21035950 PMID: 20977327 PMID: 20974195 PMID: 20970452 PMID: 20961666 PMID: 20955427 PMID: 20942592 PMID: 20932588 PMID: 20858957 PMID: 20855090 PMID: 20855089 PMID: 20854625 PMID: 20850134 PMID: 20840680 PMID: 20833237 PMID: 20817608 PMID: 20809662 PMID: 20798963 PMID: 20798750 PMID: 20739259 PMID: 20739254 PMID: 20736966 PMID: 20733494 PMID: 20731763 PMID: 20727007 PMID: 20709332 PMID: 20693632 PMID: 20639850 PMID: 20636450 PMID: 20627092 PMID: 20626335 PMID: 20605643 PMID: 20594386 PMID: 20590524 PMID: 20570489 PMID: 20567962 PMID: 20564847 PMID: 20487085 PMID: 20457955 PMID: 20448820 PMID: 20446934 PMID: 20444434 PMID: 20442827 PMID: 20413898 PMID: 20413875 PMID: 20404173 PMID: 20398148 PMID: 20398117 PMID: 20370718 PMID: 20359316 PMID: 20351074 PMID: 20303720 PMID: 20215857 PMID: 20214536 PMID: 20206067 PMID: 20189499 PMID: 20182039 PMID: 20182036 PMID: 20182031 PMID: 20182022 PMID: 20182021 PMID: 20182020 PMID: 20182015 PMID: 20164563 PMID: 20151005 PMID: 20121957 PMID: 20101718 PMID: 20056337 PMID: 20042103 PMID: 20041812 PMID: 20016220 PMID: 19948187 PMID: 19937043 PMID: 19936102 PMID: 19927134 PMID: 19922631 PMID: 19889176 PMID: 19879019 PMID: 19852002 PMID: 19844106 PMID: 19838624 PMID: 19835657 PMID: 19826563 PMID: 19821318 PMID: 19814842 PMID: 19807851 PMID: 19801534 PMID: 19783933 PMID: 19782074 PMID: 19776726 PMID: 19769454 PMID: 19742390 PMID: 19720479 PMID: 19683583 PMID: 19673450 PMID: 19650554 PMID: 19648749 PMID: 19648748 PMID: 19639021 PMID: 19619852 PMID: 19602329 PMID: 19597134 PMID: 19587815 PMID: 19587225 PMID: 19585956 PMID: 19585955 PMID: 19585947 PMID: 19579168 PMID: 21179532 PMID: 19521084 PMID: 19515691 PMID: 19507297 PMID: 19501711 PMID: 19491047 PMID: 19490292 PMID: 19488656 PMID: 19485934 PMID: 19484836 PMID: 19477197 PMID: 19433651 PMID: 19426579 PMID: 19402773 PMID: 19392962 PMID: 19375085 PMID: 19353221 PMID: 19328637 PMID: 19322689 PMID: 19281826 PMID: 19280172 PMID: 19268974 PMID: 19268394 PMID: 19264327 PMID: 19250756 PMID: 19246908 PMID: 19237536 PMID: 19228583 PMID: 19199876 PMID: 19196369 PMID: 19182465 PMID: 19175921 PMID: 19155623 PMID: 19145983 PMID: 19118811 PMID: 19079672 PMID: 19068544 PMID: 19063999 PMID: 19036496 PMID: 19023204 PMID: 19021021 PMID: 18997997 PMID: 18953718 PMID: 18945929 PMID: 18937113 PMID: 18922150 PMID: 18819689 PMID: 18763714 PMID: 18755980 PMID: 18753719 PMID: 18727557 PMID: 18707012 PMID: 18677860 PMID: 18675769 PMID: 18667359 PMID: 18629259 PMID: 18622365 PMID: 18603354 PMID: 18590347 PMID: 18561512 PMID: 18544003 PMID: 18522829 PMID: 18509646 PMID: 18499878 PMID: 18477946 PMID: 18466323 PMID: 18457635 PMID: 18457531 PMID: 18416873 PMID: 18385968 PMID: 18378224 PMID: 18363935 PMID: 18353692 PMID: 18348729 PMID: 18327871 PMID: 18321211 PMID: 18299540 PMID: 18289451 PMID: 18288934 PMID: 18276961 PMID: 18276960 PMID: 18247277 PMID: 18221232 PMID: 18221107 PMID: 18197729 PMID: 18189240 PMID: 18177501 PMID: 18166127 PMID: 18165854 PMID: 18165852 PMID: 18090879 PMID: 18070867 PMID: 18049445 PMID: 18046875 PMID: 18042993 PMID: 17971655 PMID: 18024186 PMID: 17997330 PMID: 17986333 PMID: 17981707 PMID: 17980978 PMID: 17971391 PMID: 17967779 PMID: 17942832 PMID: 17911359 PMID: 17904641 PMID: 19595960 PMID: 17890445 PMID: 17878957 PMID: 17827796 PMID: 17804352 PMID: 17711428 PMID: 17707846 PMID: 17696890 PMID: 17673335 PMID: 17670781 PMID: 17661953 PMID: 17659187 PMID: 17635634 PMID: 17611646 PMID: 17608877 PMID: 17602948 PMID: 17593948 PMID: 17553655 PMID: 17551353 PMID: 17514358 PMID: 17506226 PMID: 17490685 PMID: 17483667 PMID: 17483665 PMID: 17483664 PMID: 17470472 PMID: 17430249 PMID: 17430239 PMID: 17420963 PMID: 17420320 PMID: 19595977 PMID: 19595969 PMID: 17377202 PMID: 17375621 PMID: 17362841 PMID: 17350797 PMID: 17328914 PMID: 17319096 PMID: 17317458 PMID: 17254747 PMID: 17226743 PMID: 17210813 PMID: 17192484 PMID: 17168664 PMID: 17157543 PMID: 17110895 PMID: 17085361 PMID: 17052048 PMID: 17030648 PMID: 17022108 PMID: 17005070 PMID: 16978905 PMID: 16969326 PMID: 16969323 PMID: 16966548 PMID: 16957572 PMID: 16945216 PMID: 16945215 PMID: 16945213 PMID: 16945211 PMID: 16945210 PMID: 16938240 PMID: 16937930 PMID: 16932542 PMID: 16920207 PMID: 16918818 PMID: 16909012 PMID: 16902279 PMID: 16892270 PMID: 16867029 PMID: 16850709 PMID: 16846553 PMID: 16842191 PMID: 16828234 PMID: 16821256 PMID: 19595882 PMID: 16807483 PMID: 16786033 PMID: 16732691 PMID: 16702784 PMID: 16690992 PMID: 16651821 PMID: 16645275 PMID: 16635444 PMID: 16631882 PMID: 16625595 PMID: 16600299 PMID: 16556964 PMID: 16556518 PMID: 16527211 PMID: 16492072 PMID: 16484570 PMID: 16453055 PMID: 16408458 PMID: 16398169 PMID: 16398165 PMID: 16396227 PMID: 16386204 PMID: 16361026 PMID: 16361024 PMID: 16332384 PMID: 16327258 PMID: 16308484 PMID: 16300856 PMID: 16279269 PMID: 16276708 PMID: 16246041 PMID: 16236384 PMID: 16212686 PMID: 16194042 PMID: 16191240 PMID: 16191232 PMID: 16191231 PMID: 16191216 PMID: 16156481 PMID: 16116114 PMID: 16097385 PMID: 16050427 PMID: 16046380 PMID: 16033691 PMID: 16022573 PMID: 16021654 PMID: 15948662 PMID: 15942449 PMID: 15931374 PMID: 15920713 PMID: 15911141 PMID: 15884451 PMID: 15876979 PMID: 15874906 PMID: 15832031 PMID: 15814164 PMID: 15779680 PMID: 15721392 PMID: 15717981 PMID: 15717343 PMID: 15716519 PMID: 15684237 PMID: 15665405 PMID: 15645984 PMID: 15639310 PMID: 15635486 PMID: 15632477 PMID: 15591750 PMID: 15555488 PMID: 15537519 PMID: 15526312 PMID: 15515734 PMID: 15378248 PMID: 15358445 PMID: 15341687 PMID: 15324637 PMID: 15314113 PMID: 15286457 PMID: 15280798 PMID: 15258430 PMID: 15258208 PMID: 15224429 PMID: 15207973 PMID: 15200203 PMID: 15195690 PMID: 15191699 PMID: 15181020 PMID: 15176482 PMID: 15142650 PMID: 15141356 PMID: 15137325 PMID: 15095704 PMID: 15087585 PMID: 15066094 PMID: 15036563 PMID: 15034486 PMID: 15017355 PMID: 15000416 PMID: 14998175 PMID: 14996614 PMID: 14962623 PMID: 14746413 PMID: 14746246 PMID: 14739574 PMID: 14739544 PMID: 14710447 PMID: 14710446 PMID: 14702006 PMID: 14686563 PMID: 14676044 PMID: 14566623 PMID: 14560037 PMID: 14558983 PMID: 14558486 PMID: 14530190 PMID: 14522243 PMID: 14517614 PMID: 14503023 PMID: 14503019 PMID: 14503010 PMID: 14499288 PMID: 13677244 PMID: 12939421 PMID: 12937626 PMID: 12905081 PMID: 12895683 PMID: 12890066 PMID: 12849147 PMID: 12847569 PMID: 12808409 PMID: 12806897 PMID: 12801387 PMID: 12801386 PMID: 12795490 PMID: 12783912 PMID: 12749695 PMID: 12738320 PMID: 12736535 PMID: 12732794 PMID: 12728599 PMID: 12720610 PMID: 12716786 PMID: 12670623 PMID: 12670605 PMID: 12670054 PMID: 12657430 PMID: 12613149 PMID: 12570760 PMID: 12510954 PMID: 12505543 PMID: 12500258 PMID: 12480786 PMID: 12480734 PMID: 12480732 PMID: 12480731 PMID: 12480728 PMID: 12480441 PMID: 12441015 PMID: 12420649 PMID: 12404343 PMID: 12385592 PMID: 12366626 PMID: 12359980 PMID: 12244388 PMID: 12218642 PMID: 12212789 PMID: 12196314 PMID: 12147948 PMID: 12145456 PMID: 12140732 PMID: 12121011 PMID: 12099922 PMID: 12090556 PMID: 12090053 PMID: 12056919 PMID: 11954672 PMID: 11938501 PMID: 11925778 PMID: 11923249 PMID: 11921109 PMID: 11914555 PMID: 11890488 PMID: 11822101 PMID: 11779702 PMID: 11774221 PMID: 11761720 PMID: 11732264 PMID: 11702029 PMID: 11694979 PMID: 11676291 PMID: 11527486 PMID: 11490547 PMID: 11455131 PMID: 11442305 PMID: 11442298 PMID: 11432121 PMID: 11425934 PMID: 11425281 PMID: 11408299 PMID: 11397365 PMID: 11387503 PMID: 11378246 PMID: 11376469 PMID: 11351136 PMID: 11340256 PMID: 11271369 PMID: 11237312 PMID: 11148235 PMID: 11133172 PMID: 11096223 PMID: 11095154 PMID: 11089820 PMID: 11074159 PMID: 11058418 PMID: 11044873 PMID: 11018343 PMID: 10981741 PMID: 10978049 PMID: 10961416 PMID: 10939786 PMID: 10936882 PMID: 10923424 PMID: 10867217 PMID: 10867208 PMID: 10864134 PMID: 10844727 PMID: 10835442 PMID: 10822241 PMID: 10821138 PMID: 10819286 PMID: 10818547 PMID: 10818532 PMID: 10818505 PMID: 10818502 PMID: 10818491 PMID: 10812863 PMID: 10795724 PMID: 10794849 PMID: 10794848 PMID: 10771134 PMID: 10751108 PMID: 10738848 PMID: 10675799 PMID: 10672233 PMID: 10668705 PMID: 10654093 PMID: 10654378 PMID: 10637940 PMID: 10634269 PMID: 10537955 PMID: 10528361 PMID: 10486655 PMID: 10479748 PMID: 10449113 PMID: 10423114 PMID: 10420061 PMID: 10400067 PMID: 10334572 PMID: 10229302 PMID: 10227624 PMID: 10203126 PMID: 10203117 PMID: 10193912 PMID: 10093576 PMID: 10025786 PMID: 9988589 PMID: 9988290 PMID: 9973659 PMID: 9894287 PMID: 9853706 PMID: 9825724 PMID: 9781519 PMID: 9777922 PMID: 9740603 PMID: 9733229 PMID: 9716239 PMID: 9679207 PMID: 9606029 PMID: 9595968 PMID: 9549718 PMID: 9513031 PMID: 9510621 PMID: 9499598 PMID: 9329686 PMID: 9329681 PMID: 9329023 PMID: 9344405 PMID: 9339696 PMID: 9109802 PMID: 9380254 PMID: 9152727 PMID: 9065545 PMID: 9052903 PMID: 9024796 PMID: 9452785 PMID: 9161470 PMID: 8961673 PMID: 8843880 PMID: 9138399 PMID: 8809005 PMID: 8708297 PMID: 8831259 PMID: 8791246 PMID: 8773818 PMID: 8649554 PMID: 8609748 PMID: 8576514 PMID: 8561165 PMID: 14653448 PMID: 9063907 PMID: 8538823 PMID: 7473847 PMID: 7595642 PMID: 7619027 PMID: 8586291 PMID: 8543901 PMID: 7477666 PMID: 7765749 PMID: 7993923 PMID: 8087168 PMID: 8027633 PMID: 8129637 PMID: 8129197 PMID: 9719668 PMID: 8290099 PMID: 8253393 PMID: 8342875 PMID: 8503257 PMID: 8463769 PMID: 8475231 PMID: 8437692 PMID: 8418119 PMID: 8299673 PMID: 8272177 PMID: 1436202 PMID: 1430852 PMID: 1524516 PMID: 1599993 PMID: 1734748 PMID: 1528370 PMID: 1353650 PMID: 1841609 PMID: 1961401 PMID: 1953397 PMID: 1929892 PMID: 1891088 PMID: 1868414 PMID: 1866768 PMID: 2039381 PMID: 2006654 PMID: 2818256 PMID: 2760643 PMID: 2729334 PMID: 2736596 PMID: 2712531 PMID: 2651569 PMID: 3066459 PMID: 3339230 PMID: 3197048 PMID: 3288893 PMID: 3279483 PMID: 3496763 PMID: 3800720 PMID: 2439247 PMID: 3761519 PMID: 3785532 PMID: 3717434 PMID: 2872473 PMID: 4003595 PMID: 6539433 PMID: 6626840 PMID: 6823631 PMID: 7020434 PMID: 845610 PMID: 961375 PMID: 4426320 PMID: 4801351 PMID: 4112384 |
| Amino sugar and nucleotide sugar metabolism | PMID: 16645728 |
| Aminoacyl-tRNA biosynthesis | PMID: 7591336 |
| Amyotrophic lateral sclerosis (ALS) | PMID: 18286388 PMID: 16614031 PMID: 12385592 PMID: 11257787 PMID: 10828653 PMID: 8902322 PMID: 8543951 PMID: 3614653 |
| Antigen processing and presentation |  |
| Apoptosis | PMID: 21241794 PMID: 21235893 PMID: 21234356 PMID: 21228576 PMID: 21221781 PMID: 21210757 PMID: 21200363 PMID: 21199786 PMID: 21195054 PMID: 21193590 PMID: 21185376 PMID: 21180298 PMID: 21171923 PMID: 21160482 PMID: 21150127 PMID: 21136477 PMID: 21136300 PMID: 21122203 PMID: 21121369 PMID: 21116108 PMID: 21113490 PMID: 21112119 PMID: 21093722 PMID: 21088216 PMID: 21074972 PMID: 21057104 PMID: 21056597 PMID: 21053206 PMID: 21052012 PMID: 21037114 PMID: 20981034 PMID: 20980738 PMID: 20975036 PMID: 20975034 PMID: 20942592 PMID: 20933604 PMID: 20932656 PMID: 20923679 PMID: 20921950 PMID: 20889845 PMID: 20881610 PMID: 20863775 PMID: 20849327 PMID: 20833959 PMID: 20822476 PMID: 20813180 PMID: 20811331 PMID: 20734021 PMID: 20729036 PMID: 20725711 PMID: 20723731 PMID: 20708678 PMID: 20705925 PMID: 20703435 PMID: 20702857 PMID: 20688155 PMID: 20680403 PMID: 20675960 PMID: 20668454 PMID: 20660817 PMID: 20660518 PMID: 20650957 PMID: 20637184 PMID: 20634940 PMID: 20633610 PMID: 20590836 PMID: 20619278 PMID: 20608051 PMID: 20606107 PMID: 20603655 PMID: 20600153 PMID: 20591971 PMID: 20588062 PMID: 20585109 PMID: 20584172 PMID: 20577898 PMID: 20570807 PMID: 20552556 PMID: 20551590 PMID: 20546781 PMID: 20537355 PMID: 20522580 PMID: 20531983 PMID: 20531215 PMID: 20529999 PMID: 20523267 PMID: 20521441 PMID: 20520578 PMID: 20516761 PMID: 20514524 PMID: 20502140 PMID: 20491649 PMID: 20489655 PMID: 20472592 PMID: 20471212 PMID: 20463652 PMID: 20460401 PMID: 20457203 PMID: 20451279 PMID: 20443776 PMID: 20438756 PMID: 20430947 PMID: 20427266 PMID: 20427168 PMID: 20421515 PMID: 20410916 PMID: 20386756 PMID: 20406798 PMID: 20401843 PMID: 20399520 PMID: 20398479 PMID: 20385969 PMID: 20382855 PMID: 20351341 PMID: 20305782 PMID: 20298396 PMID: 20237455 PMID: 20216170 PMID: 20229524 PMID: 20224526 PMID: 20217871 PMID: 20213196 PMID: 20212261 PMID: 20210931 PMID: 20206649 PMID: 20206401 PMID: 20204747 PMID: 20204744 PMID: 20204736 PMID: 20204730 PMID: 20203299 PMID: 20195855 PMID: 20186129 PMID: 20178504 PMID: 20177000 PMID: 20169897 PMID: 20160044 PMID: 20150559 PMID: 20145415 PMID: 20142000 PMID: 20137497 PMID: 20135646 PMID: 20132409 PMID: 20117135 PMID: 20110409 PMID: 20105174 PMID: 20103667 PMID: 20075332 PMID: 20074922 PMID: 20073161 PMID: 20065153 PMID: 20051910 PMID: 20051854 PMID: 20051385 PMID: 20041812 PMID: 20034524 PMID: 20032843 PMID: 20032120 PMID: 20029634 PMID: 20029534 PMID: 20026055 PMID: 20018952 PMID: 20015035 PMID: 20008031 PMID: 19965662 PMID: 19953877 PMID: 19953399 PMID: 19949425 PMID: 19941836 PMID: 19937369 PMID: 19937043 PMID: 19927348 PMID: 19923886 PMID: 19919985 PMID: 19916846 PMID: 19915982 PMID: 19911467 PMID: 19910640 PMID: 19910005 PMID: 19906946 PMID: 19906783 PMID: 19905982 PMID: 19895749 PMID: 19895710 PMID: 19884466 PMID: 19863766 PMID: 19861349 PMID: 19853589 PMID: 19841294 PMID: 19841288 PMID: 19835652 PMID: 19834384 PMID: 19833459 PMID: 19825167 PMID: 19821832 PMID: 19820199 PMID: 19817870 PMID: 19811364 PMID: 19811360 PMID: 19807685 PMID: 19782107 PMID: 19776560 PMID: 19765568 PMID: 19758727 PMID: 19746421 PMID: 19744046 PMID: 19738161 PMID: 19737862 PMID: 19729440 PMID: 19718604 PMID: 19713449 PMID: 19706371 PMID: 19703674 PMID: 19697799 PMID: 19684610 PMID: 19684120 PMID: 19679640 PMID: 19673631 PMID: 19668263 PMID: 19666835 PMID: 19653229 PMID: 19647003 PMID: 19644200 PMID: 19625373 PMID: 19620519 PMID: 19619526 PMID: 19617400 PMID: 19617336 PMID: 19555853 PMID: 19581797 PMID: 19581502 PMID: 19574692 PMID: 19567157 PMID: 19566840 PMID: 19566746 PMID: 19562677 PMID: 19535947 PMID: 19543386 PMID: 19531939 PMID: 19526390 PMID: 19523699 PMID: 19506538 PMID: 19485926 PMID: 19473124 PMID: 19464477 PMID: 19458122 PMID: 19458120 PMID: 19442097 PMID: 19439736 PMID: 19435785 PMID: 19429773 PMID: 19427678 PMID: 19427584 PMID: 19427496 PMID: 19423951 PMID: 19417955 PMID: 19414648 PMID: 19413999 PMID: 19412697 PMID: 19398656 PMID: 19386984 PMID: 19383602 PMID: 19381013 PMID: 19380417 PMID: 19376400 PMID: 19372634 PMID: 19365015 PMID: 19355910 PMID: 19349318 PMID: 19345526 PMID: 19343290 PMID: 19340666 PMID: 19330911 PMID: 19330905 PMID: 19330083 PMID: 19325075 PMID: 19324947 PMID: 19323621 PMID: 19321739 PMID: 19320535 PMID: 19308045 PMID: 19298536 PMID: 19289568 PMID: 19289456 PMID: 19288153 PMID: 19270180 PMID: 19268530 PMID: 19265784 PMID: 19262492 PMID: 19251722 PMID: 19237446 PMID: 19236153 PMID: 19234343 PMID: 19228077 PMID: 19221208 PMID: 19208360 PMID: 19207270 PMID: 19204184 PMID: 19204178 PMID: 19182492 PMID: 21186609 PMID: 19172389 PMID: 19171978 PMID: 19155791 PMID: 19155788 PMID: 19151695 PMID: 19148610 PMID: 19148291 PMID: 19145821 PMID: 19139606 PMID: 19139264 PMID: 19132996 PMID: 19120131 PMID: 20469678 PMID: 20210098 PMID: 19114643 PMID: 19111553 PMID: 19109969 PMID: 19104057 PMID: 19102871 PMID: 19087667 PMID: 19085803 PMID: 19077682 PMID: 19075656 PMID: 19074475 PMID: 19068010 PMID: 19065997 PMID: 19064852 PMID: 19060130 PMID: 19051724 PMID: 19041334 PMID: 20804105 PMID: 19027602 PMID: 19027145 PMID: 19026018 PMID: 19011668 PMID: 19010896 PMID: 19010346 PMID: 19008804 PMID: 19007669 PMID: 19005302 PMID: 18992279 PMID: 18991571 PMID: 18989704 PMID: 18983459 PMID: 18978196 PMID: 18971427 PMID: 18955663 PMID: 18939363 PMID: 18938725 PMID: 18931051 PMID: 18924487 PMID: 18923387 PMID: 18849542 PMID: 18848926 PMID: 18844104 PMID: 18836920 PMID: 18823550 PMID: 18814088 PMID: 18813182 PMID: 18793114 PMID: 18787714 PMID: 18787517 PMID: 18783313 PMID: 18776064 PMID: 18768400 PMID: 18762556 PMID: 18759859 PMID: 18724960 PMID: 18723761 PMID: 18701465 PMID: 18695148 PMID: 18688015 PMID: 18678790 PMID: 18676690 PMID: 18676676 PMID: 18667463 PMID: 18653232 PMID: 18650792 PMID: 18650487 PMID: 18645050 PMID: 18641182 PMID: 18600314 PMID: 18596905 PMID: 18593227 PMID: 18587055 PMID: 18587045 PMID: 18582719 PMID: 18556624 PMID: 18552156 PMID: 18551017 PMID: 18542928 PMID: 18542120 PMID: 18540612 PMID: 18534913 PMID: 18516627 PMID: 18498720 PMID: 18494000 PMID: 18481270 PMID: 18480747 PMID: 18476793 PMID: 18464051 PMID: 18459466 PMID: 18459124 PMID: 18445624 PMID: 18436805 PMID: 18434301 PMID: 18420966 PMID: 18418439 PMID: 18413786 PMID: 18400414 PMID: 18398704 PMID: 18398344 PMID: 18386601 PMID: 18372234 PMID: 18357915 PMID: 18336234 PMID: 18327085 PMID: 18319309 PMID: 18318163 PMID: 18315495 PMID: 18303084 PMID: 18301873 PMID: 18282695 PMID: 18276926 PMID: 18227481 PMID: 18269754 PMID: 18265484 PMID: 18260912 PMID: 18258847 PMID: 18256363 PMID: 18253094 PMID: 18078390 PMID: 18250361 PMID: 18247327 PMID: 18246055 PMID: 18241929 PMID: 18226850 PMID: 18220772 PMID: 18219296 PMID: 18218830 PMID: 18211807 PMID: 18204248 PMID: 18202349 PMID: 18195174 PMID: 18192217 PMID: 18184111 PMID: 18182103 PMID: 18180403 PMID: 19804272 PMID: 18091526 PMID: 18084317 PMID: 18083891 PMID: 18083403 PMID: 18081207 PMID: 18079409 PMID: 18071063 PMID: 18070548 PMID: 18070547 PMID: 18057217 PMID: 18049311 PMID: 21136660 PMID: 18045820 PMID: 18033815 PMID: 18029156 PMID: 18025171 PMID: 18007089 PMID: 18006491 PMID: 17997040 PMID: 17993597 PMID: 17982966 PMID: 17977949 PMID: 17977916 PMID: 17975100 PMID: 17973309 PMID: 17971205 PMID: 17968307 PMID: 17938379 PMID: 17934515 PMID: 17932321 PMID: 17932313 PMID: 17924279 PMID: 17923589 PMID: 17923213 PMID: 17921808 PMID: 17920518 PMID: 17903843 PMID: 17901689 PMID: 17900330 PMID: 17885560 PMID: 17881463 PMID: 17878288 PMID: 17870236 PMID: 17870114 PMID: 17869258 PMID: 17868638 PMID: 17853317 PMID: 17826763 PMID: 17825849 PMID: 17786052 PMID: 17786025 PMID: 17785964 PMID: 17784886 PMID: 17766279 PMID: 17761111 PMID: 17727843 PMID: 17720166 PMID: 17714733 PMID: 17692012 PMID: 17698735 PMID: 17698584 PMID: 17695700 PMID: 17693442 PMID: 17684962 PMID: 17679833 PMID: 17675370 PMID: 17666920 PMID: 17652367 PMID: 17646759 PMID: 17646574 PMID: 17642135 PMID: 17638876 PMID: 17622738 PMID: 17611646 PMID: 17609290 PMID: 17601794 PMID: 17596340 PMID: 17593948 PMID: 17592516 PMID: 17583356 PMID: 17577098 PMID: 17570945 PMID: 17567571 PMID: 17564028 PMID: 17563544 PMID: 17556883 PMID: 17554970 PMID: 17538008 PMID: 17535738 PMID: 17533199 PMID: 17533012 PMID: 17531161 PMID: 17526595 PMID: 17525251 PMID: 17506493 PMID: 17505622 PMID: 17502186 PMID: 17501693 PMID: 17498056 PMID: 17496367 PMID: 17494630 PMID: 17491205 PMID: 17479183 PMID: 17469331 PMID: 17468705 PMID: 17459952 PMID: 17456758 PMID: 17439412 PMID: 17434478 PMID: 17430643 PMID: 17429340 PMID: 17414669 PMID: 17413127 PMID: 17402563 PMID: 17392853 PMID: 17392319 PMID: 17391216 PMID: 17355088 PMID: 17347486 PMID: 17344894 PMID: 17341625 PMID: 17338926 PMID: 17337597 PMID: 17337126 PMID: 17332488 PMID: 17327050 PMID: 17325658 PMID: 17322420 PMID: 17316158 PMID: 17314083 PMID: 17313787 PMID: 17309952 PMID: 17306711 PMID: 17305584 PMID: 17303202 PMID: 17292046 PMID: 17290492 PMID: 17289831 PMID: 17287428 PMID: 17283249 PMID: 17280880 PMID: 17275583 PMID: 17272823 PMID: 17272402 PMID: 17267549 PMID: 17259069 PMID: 17258719 PMID: 17257271 PMID: 17242991 PMID: 17241886 PMID: 17230612 PMID: 17226020 PMID: 17224475 PMID: 17222789 PMID: 17220956 PMID: 17220028 PMID: 17218601 PMID: 17215444 PMID: 17210842 PMID: 17199211 PMID: 17181659 PMID: 17175463 PMID: 17173503 PMID: 17169499 PMID: 17169354 PMID: 17159082 PMID: 17143192 PMID: 17142350 PMID: 17137212 PMID: 17136686 PMID: 17127075 PMID: 17123106 PMID: 17122582 PMID: 17122447 PMID: 17108707 PMID: 17102939 PMID: 17098760 PMID: 17097823 PMID: 17090407 PMID: 17085423 PMID: 17084706 PMID: 17077282 PMID: 17074304 PMID: 17073607 PMID: 17066325 PMID: 17065373 PMID: 17060874 PMID: 17057830 PMID: 17051236 PMID: 17030903 PMID: 17023878 PMID: 17017920 PMID: 17015250 PMID: 16996066 PMID: 16978905 PMID: 16973905 PMID: 16972538 PMID: 16971980 PMID: 16963253 PMID: 16959961 PMID: 16951131 PMID: 16946081 PMID: 16940561 PMID: 16940231 PMID: 16935777 PMID: 16921121 PMID: 16914427 PMID: 16908774 PMID: 16908624 PMID: 16900013 PMID: 16897865 PMID: 16896805 PMID: 16890922 PMID: 16877524 PMID: 16870458 PMID: 16868559 PMID: 16867874 PMID: 16857764 PMID: 16837795 PMID: 16828723 PMID: 16825605 PMID: 16822508 PMID: 16818501 PMID: 16816138 PMID: 16815889 PMID: 16805218 PMID: 16800727 PMID: 16796160 PMID: 16786111 PMID: 16778983 PMID: 16778006 PMID: 16771662 PMID: 16765337 PMID: 16762988 PMID: 16755209 PMID: 16755196 PMID: 16753786 PMID: 16738532 PMID: 16735463 PMID: 16735095 PMID: 16724944 PMID: 16724932 PMID: 16716756 PMID: 16699275 PMID: 16699260 PMID: 16698853 PMID: 16697056 PMID: 16689363 PMID: 16688118 PMID: 16685597 PMID: 16685380 PMID: 16675585 PMID: 16673649 PMID: 16672320 PMID: 16636193 PMID: 16636191 PMID: 16633987 PMID: 16631164 PMID: 16617095 PMID: 16603723 PMID: 16601575 PMID: 16597688 PMID: 16585965 PMID: 16554918 PMID: 16554418 PMID: 16541023 PMID: 16537870 PMID: 16534320 PMID: 16534002 PMID: 16531988 PMID: 16528750 PMID: 16523421 PMID: 16501604 PMID: 16501242 PMID: 16497808 PMID: 16491056 PMID: 16485256 PMID: 16484628 PMID: 16484570 PMID: 16467659 PMID: 16458203 PMID: 16456109 PMID: 16440585 PMID: 16440149 PMID: 16437745 PMID: 16434570 PMID: 16431960 PMID: 16423283 PMID: 16415490 PMID: 16415374 PMID: 16380515 PMID: 16373861 PMID: 16371224 PMID: 16368652 PMID: 16364997 PMID: 16361357 PMID: 16357305 PMID: 16356795 PMID: 16350786 PMID: 16344369 PMID: 16339963 PMID: 16339840 PMID: 16319344 PMID: 16316322 PMID: 16309576 PMID: 16309229 PMID: 16308484 PMID: 16302997 PMID: 16291522 PMID: 16273412 PMID: 16263938 PMID: 16258272 PMID: 16257070 PMID: 16248829 PMID: 16239600 PMID: 16239589 PMID: 16226132 PMID: 16223783 PMID: 16223043 PMID: 16221212 PMID: 16218486 PMID: 16199478 PMID: 16186060 PMID: 16178788 PMID: 16173947 PMID: 16166241 PMID: 16164628 PMID: 16151651 PMID: 16142693 PMID: 16129952 PMID: 16128280 PMID: 16126273 PMID: 16125050 PMID: 16123331 PMID: 16121311 PMID: 16118392 PMID: 16115034 PMID: 16113463 PMID: 16104842 PMID: 16102048 PMID: 16100039 PMID: 16098091 PMID: 16075725 PMID: 16075407 PMID: 16060848 PMID: 16059798 PMID: 16054890 PMID: 16054866 PMID: 16046310 PMID: 16043172 PMID: 16037565 PMID: 16035650 PMID: 16027270 PMID: 16024013 PMID: 16020484 PMID: 16005610 PMID: 15998704 PMID: 15996271 PMID: 15996002 PMID: 15995138 PMID: 15984907 PMID: 15979050 PMID: 15976312 PMID: 15969209 PMID: 15964095 PMID: 15955132 PMID: 15931388 PMID: 15931382 PMID: 15927895 PMID: 15925276 PMID: 15922255 PMID: 15919782 PMID: 15911745 PMID: 15897232 PMID: 15894827 PMID: 15890798 PMID: 15890797 PMID: 15889280 PMID: 15886352 PMID: 15884660 PMID: 15883498 PMID: 15880269 PMID: 15878795 PMID: 15878794 PMID: 15877958 PMID: 15867141 PMID: 15863619 PMID: 21171340 PMID: 15845886 PMID: 15845210 PMID: 15838489 PMID: 15837836 PMID: 15824197 PMID: 15824196 PMID: 15823277 PMID: 15817798 PMID: 15812766 PMID: 15803113 PMID: 15800411 PMID: 15798538 PMID: 15790956 PMID: 15790570 PMID: 15781756 PMID: 15775190 PMID: 15772095 PMID: 15771551 PMID: 15769854 PMID: 15769308 PMID: 15759048 PMID: 15755871 PMID: 15755024 PMID: 15748529 PMID: 15732000 PMID: 15728787 PMID: 15723095 PMID: 15719257 PMID: 15718424 PMID: 15718270 PMID: 15716683 PMID: 15703702 PMID: 15698430 PMID: 15695426 PMID: 15687129 PMID: 15673306 PMID: 15669369 PMID: 15665405 PMID: 15653716 PMID: 15650305 PMID: 15641470 PMID: 15640279 PMID: 15629664 PMID: 15603432 PMID: 15601749 PMID: 15595927 PMID: 15578657 PMID: 15569301 PMID: 15561331 PMID: 15559224 PMID: 15545511 PMID: 15539463 PMID: 15539106 PMID: 15534078 PMID: 15506383 PMID: 15492992 PMID: 15492481 PMID: 15475499 PMID: 15469393 PMID: 15466266 PMID: 15465369 PMID: 15451437 PMID: 15384194 PMID: 15382615 PMID: 15375007 PMID: 15371167 PMID: 15345697 PMID: 15340658 PMID: 15338229 PMID: 19780248 PMID: 15333333 PMID: 15330733 PMID: 15320852 PMID: 15320833 PMID: 15320511 PMID: 15313118 PMID: 15304499 PMID: 15302785 PMID: 15301727 PMID: 15284284 PMID: 15277326 PMID: 15276015 PMID: 15273829 PMID: 15257859 PMID: 15255232 PMID: 15252775 PMID: 15249178 PMID: 15246028 PMID: 15232622 PMID: 15222251 PMID: 15213632 PMID: 15201556 PMID: 15200422 PMID: 15200251 PMID: 15195685 PMID: 15193221 PMID: 15192576 PMID: 15188402 PMID: 15187132 PMID: 15183103 PMID: 15178645 PMID: 15173597 PMID: 15172670 PMID: 15171368 PMID: 15171336 PMID: 15167436 PMID: 15148457 PMID: 15147828 PMID: 15142859 PMID: 15140747 PMID: 15128503 PMID: 15127885 PMID: 15123578 PMID: 15117910 PMID: 15115333 PMID: 15098086 PMID: 15096473 PMID: 15095481 PMID: 15090261 PMID: 15087290 PMID: 15073487 PMID: 15069561 PMID: 15069378 PMID: 15064713 PMID: 15051933 PMID: 15037594 PMID: 15036563 PMID: 15033850 PMID: 15030288 PMID: 15027449 PMID: 14988519 PMID: 14981066 PMID: 14977533 PMID: 14975589 PMID: 14971574 PMID: 14967844 PMID: 14967838 PMID: 14967835 PMID: 14967829 PMID: 14871401 PMID: 14871060 PMID: 14871052 PMID: 14766767 PMID: 14764919 PMID: 14764147 PMID: 14750147 PMID: 14745571 PMID: 14736733 PMID: 14732786 PMID: 14728862 PMID: 14727993 PMID: 14724354 PMID: 14717385 PMID: 14715186 PMID: 14704913 PMID: 14698996 PMID: 14695338 PMID: 14678698 PMID: 14675038 PMID: 14675034 PMID: 14670839 PMID: 14662648 PMID: 14659811 PMID: 14656699 PMID: 14645134 PMID: 14640931 PMID: 14645111 PMID: 14621599 PMID: 14621104 PMID: 14608940 PMID: 14581295 PMID: 14551246 PMID: 14523805 PMID: 14521925 PMID: 14514645 PMID: 14503927 PMID: 14499856 PMID: 13680276 PMID: 12969124 PMID: 12966667 PMID: 12963647 PMID: 12960676 PMID: 12957878 PMID: 12939515 PMID: 12904575 PMID: 12899336 PMID: 12881480 PMID: 12874107 PMID: 12874095 PMID: 12869365 PMID: 12843686 PMID: 12842818 PMID: 12828857 PMID: 12808483 PMID: 12804596 PMID: 12799307 PMID: 12789027 PMID: 12787408 PMID: 12750121 PMID: 12748555 PMID: 12740218 PMID: 12738800 PMID: 12728683 PMID: 12722039 PMID: 12714563 PMID: 12699073 PMID: 12690034 PMID: 12681244 PMID: 12686738 PMID: 12684765 PMID: 12684706 PMID: 12684506 PMID: 12683421 PMID: 12679323 PMID: 12677190 PMID: 12668584 PMID: 12654712 PMID: 12642506 PMID: 12642016 PMID: 12634314 PMID: 12632608 PMID: 12625816 PMID: 12623880 PMID: 12623481 PMID: 12618239 PMID: 12600884 PMID: 12583607 PMID: 12575293 PMID: 12570806 PMID: 12570788 PMID: 12566067 PMID: 12536482 PMID: 12525995 PMID: 12524467 PMID: 12519090 PMID: 12502909 PMID: 12488510 PMID: 12487919 PMID: 12484517 PMID: 12475805 PMID: 12466046 PMID: 12466044 PMID: 12464684 PMID: 12452333 PMID: 12446203 PMID: 12444202 PMID: 12443711 PMID: 12442352 PMID: 12440782 PMID: 12429110 PMID: 12422546 PMID: 12411972 PMID: 12411458 PMID: 12411096 PMID: 12401536 PMID: 12401248 PMID: 12397679 PMID: 12393934 PMID: 12378819 PMID: 12370493 PMID: 12365859 PMID: 12358155 PMID: 12352014 PMID: 12296852 PMID: 12224048 PMID: 12205919 PMID: 12219172 PMID: 12215472 PMID: 12215428 PMID: 12193662 PMID: 12187097 PMID: 12182967 PMID: 12180853 PMID: 12163130 PMID: 12152103 PMID: 12135323 PMID: 12133298 PMID: 12120413 PMID: 12083487 PMID: 12047042 PMID: 12034664 PMID: 12027563 PMID: 12025960 PMID: 12021538 PMID: 12011661 PMID: 12011650 PMID: 12011583 PMID: 12004244 PMID: 11997075 PMID: 11961112 PMID: 11959625 PMID: 11940365 PMID: 11937560 PMID: 11936844 PMID: 11930691 PMID: 11924754 PMID: 11923249 PMID: 11916613 PMID: 11916363 PMID: 11914746 PMID: 11910304 PMID: 11891510 PMID: 11884276 PMID: 11880294 PMID: 11869174 PMID: 11868908 PMID: 11865097 PMID: 11844871 PMID: 11842228 PMID: 11836163 PMID: 11827997 PMID: 11818026 PMID: 11817654 PMID: 11809907 PMID: 11809715 PMID: 11806806 PMID: 11799084 PMID: 11799082 PMID: 11790924 PMID: 11788782 PMID: 11788406 PMID: 11787813 PMID: 11786155 PMID: 11786083 PMID: 11776172 PMID: 11772940 PMID: 11754958 PMID: 11751726 PMID: 11751725 PMID: 11751722 PMID: 11751707 PMID: 11751706 PMID: 11743883 PMID: 11730557 PMID: 11728960 PMID: 11727758 PMID: 11726734 PMID: 11711513 PMID: 11701629 PMID: 11696435 PMID: 11693558 PMID: 11692472 PMID: 11687842 PMID: 11641234 PMID: 11601340 PMID: 11598137 PMID: 11593108 PMID: 11590839 PMID: 11590837 PMID: 11582628 PMID: 11566936 PMID: 11566935 PMID: 11564975 PMID: 11561148 PMID: 11546656 PMID: 11527386 PMID: 11523425 PMID: 11509485 PMID: 11504780 PMID: 11493536 PMID: 11478033 PMID: 11473637 PMID: 11470015 PMID: 11470012 PMID: 11463759 PMID: 11460568 PMID: 11459340 PMID: 11433830 PMID: 11409658 PMID: 11412743 PMID: 11410492 PMID: 11408383 PMID: 11399895 PMID: 11399641 PMID: 11381292 PMID: 11380827 PMID: 11379792 PMID: 11369834 PMID: 11358951 PMID: 11331262 PMID: 11321839 PMID: 11309529 PMID: 11299309 PMID: 11289502 PMID: 11284547 PMID: 11282056 PMID: 11278731 PMID: 11275023 PMID: 11274739 PMID: 11269510 PMID: 11266827 PMID: 11261820 PMID: 11259528 PMID: 11259391 PMID: 11254533 PMID: 11246972 PMID: 11244682 PMID: 11241129 PMID: 11230370 PMID: 11230338 PMID: 11230311 PMID: 11223164 PMID: 11200813 PMID: 11193480 PMID: 11191362 PMID: 11176262 PMID: 11168948 PMID: 11168946 PMID: 11162777 PMID: 11160146 PMID: 11159195 PMID: 11156958 PMID: 11152759 PMID: 11139485 PMID: 11139830 PMID: 11139087 PMID: 11135076 PMID: 11131277 PMID: 11130838 PMID: 11104784 PMID: 11085282 PMID: 11082155 PMID: 11065229 PMID: 11055983 PMID: 11044221 PMID: 11038804 PMID: 11026979 PMID: 11009576 PMID: 11008165 PMID: 10993849 PMID: 10986432 PMID: 10976827 PMID: 10976051 PMID: 10972681 PMID: 10961416 PMID: 10946849 PMID: 10930186 PMID: 10926866 PMID: 10926769 PMID: 10926548 PMID: 10919577 PMID: 10914632 PMID: 10911636 PMID: 10904021 PMID: 10904018 PMID: 10904017 PMID: 10894795 PMID: 10889464 PMID: 10873149 PMID: 10859529 PMID: 10856260 PMID: 10849306 PMID: 10843941 PMID: 10838810 PMID: 10835689 PMID: 10828756 PMID: 10826007 PMID: 10821433 PMID: 10821351 PMID: 10818066 PMID: 10818065 PMID: 10807584 PMID: 10792606 PMID: 10792274 PMID: 10783154 PMID: 10775071 PMID: 10756925 PMID: 10746817 PMID: 10728395 PMID: 10726976 PMID: 10726718 PMID: 10711738 PMID: 10710817 PMID: 10685061 PMID: 10679512 PMID: 10670473 PMID: 10658018 PMID: 10642312 PMID: 10642304 PMID: 10642270 PMID: 11116126 PMID: 11110769 PMID: 11104740 PMID: 10625574 PMID: 10619858 PMID: 10619573 PMID: 10614145 PMID: 10609689 PMID: 10608484 PMID: 10599559 PMID: 10588195 PMID: 10588088 PMID: 10564091 PMID: 10551895 PMID: 10526901 PMID: 10523336 PMID: 10523335 PMID: 10518388 PMID: 10516396 PMID: 10490095 PMID: 10487483 PMID: 10480475 PMID: 10468528 PMID: 10456200 PMID: 10454446 PMID: 10454440 PMID: 10446995 PMID: 10422461 PMID: 10373214 PMID: 10364709 PMID: 10350674 PMID: 10334572 PMID: 10326685 PMID: 10330036 PMID: 10326717 PMID: 10224225 PMID: 10221347 PMID: 10216440 PMID: 10082507 PMID: 10079015 PMID: 10076916 PMID: 10075388 PMID: 9972329 PMID: 9931097 PMID: 9922372 PMID: 9891592 PMID: 9888876 PMID: 9887983 PMID: 9884429 PMID: 9864601 PMID: 9839281 PMID: 9823788 PMID: 9822143 PMID: 9806660 PMID: 9804303 PMID: 9785475 PMID: 9752897 PMID: 9748760 PMID: 9742261 PMID: 9719148 PMID: 9719055 PMID: 9717053 PMID: 9715819 PMID: 9707500 PMID: 9702295 PMID: 9676650 PMID: 9665366 PMID: 9647892 PMID: 9593871 PMID: 9583357 PMID: 9576088 PMID: 9554559 PMID: 9549226 PMID: 9541273 PMID: 9530177 PMID: 9528285 PMID: 9490660 PMID: 9503101 PMID: 9431850 PMID: 9457442 PMID: 9453351 PMID: 9453296 PMID: 9445349 PMID: 9412541 PMID: 9409811 PMID: 9375940 PMID: 9403598 PMID: 9396461 PMID: 9395125 PMID: 9369276 PMID: 9369251 PMID: 9356598 PMID: 9323100 PMID: 9323012 PMID: 9315566 PMID: 9314417 PMID: 9176300 PMID: 9149671 PMID: 9136906 PMID: 9166987 PMID: 9094991 PMID: 9146986 PMID: 9146979 PMID: 9052895 PMID: 9087915 PMID: 9476548 PMID: 9039125 PMID: 9120691 PMID: 9007722 PMID: 8943951 PMID: 8896663 PMID: 8899552 PMID: 8757150 PMID: 8967027 PMID: 8766157 PMID: 8946072 PMID: 8613247 PMID: 8780000 PMID: 10388004 PMID: 8988460 PMID: 8720083 PMID: 8682057 PMID: 7558225 PMID: 7743153 PMID: 7913956 PMID: 7909858 PMID: 7747162 PMID: 8370649 PMID: 1513115 |
| Arachidonic acid metabolism | PMID: 20797450 PMID: 20694143 PMID: 20622163 PMID: 20093140 PMID: 19485927 PMID: 19440924 PMID: 19077685 PMID: 19047578 PMID: 18768398 PMID: 18378855 PMID: 18219097 PMID: 18067589 PMID: 17450683 PMID: 16915013 PMID: 16487260 PMID: 16316349 PMID: 16207400 PMID: 16199435 PMID: 15994032 PMID: 15780761 PMID: 15238791 PMID: 14691199 PMID: 14638916 PMID: 14637180 PMID: 14629650 PMID: 12566962 PMID: 12357134 PMID: 12215466 PMID: 12185963 PMID: 11737606 PMID: 11727407 PMID: 11566953 PMID: 11208362 PMID: 11040244 PMID: 11001943 PMID: 10620200 PMID: 10320629 PMID: 10211585 PMID: 10070137 PMID: 9730827 PMID: 9688677 PMID: 9556499 PMID: 9495265 PMID: 9322988 PMID: 9128202 PMID: 9056695 PMID: 9022553 PMID: 9425643 PMID: 8946652 PMID: 8790031 PMID: 8856265 PMID: 8307634 PMID: 8065610 PMID: 8112447 PMID: 8371169 PMID: 8357995 PMID: 1639455 PMID: 1509159 PMID: 1777588 PMID: 1937691 PMID: 1797924 PMID: 2045172 PMID: 1903902 PMID: 2395329 PMID: 2124406 PMID: 2141003 PMID: 2406318 PMID: 2286824 PMID: 2118711 PMID: 2573371 PMID: 2514650 PMID: 2527469 PMID: 2624757 PMID: 2504817 PMID: 2647108 PMID: 2774404 PMID: 3060428 PMID: 3178481 PMID: 3136669 PMID: 2968672 PMID: 3386243 PMID: 3133889 PMID: 3105370 PMID: 3923843 PMID: 2987123 PMID: 3923251 PMID: 6362546 PMID: 6354535 PMID: 6337423 PMID: 7051860 PMID: 7036731 PMID: 7380683 |
| Arginine and proline metabolism | PMID: 20823711 PMID: 20506010 PMID: 19801451 PMID: 19728040 PMID: 19633445 PMID: 19491403 PMID: 19360123 PMID: 19126665 PMID: 19033012 PMID: 18955661 PMID: 18835922 PMID: 18657187 PMID: 18437360 PMID: 18047624 PMID: 17824802 PMID: 17645639 PMID: 17470726 PMID: 17379008 PMID: 17192295 PMID: 17009099 PMID: 16710350 PMID: 16669787 PMID: 16531806 PMID: 16220007 PMID: 16197366 PMID: 15994432 PMID: 15948896 PMID: 15566389 PMID: 15290337 PMID: 15215814 PMID: 15145922 PMID: 14646364 PMID: 14569001 PMID: 12900428 PMID: 12714875 PMID: 12623960 PMID: 12411476 PMID: 12079874 PMID: 12077487 PMID: 12047040 PMID: 11956573 PMID: 11951116 PMID: 11881129 PMID: 11566904 PMID: 11564976 PMID: 11360627 PMID: 11212967 PMID: 11171663 PMID: 10994755 PMID: 10805405 PMID: 10722795 PMID: 10625224 PMID: 10585456 PMID: 10564706 PMID: 9987649 PMID: 9933759 PMID: 9928759 PMID: 9890305 PMID: 9441842 PMID: 9344638 PMID: 9680296 PMID: 8957040 PMID: 8869001 PMID: 9085355 PMID: 8818205 PMID: 8566127 PMID: 8586803 PMID: 7554759 PMID: 8565028 PMID: 7643519 PMID: 7611481 PMID: 7723354 PMID: 8000447 PMID: 8143302 PMID: 8274625 PMID: 8293769 PMID: 8495554 PMID: 8389325 PMID: 8343226 PMID: 8272217 PMID: 1338079 PMID: 1737647 PMID: 1887930 PMID: 1900513 PMID: 1972412 PMID: 2698964 PMID: 2825944 PMID: 2887673 PMID: 3519238 PMID: 3902342 PMID: 2859221 PMID: 6311736 PMID: 6337509 PMID: 6292084 PMID: 90271 |
| Arrhythmogenic right ventricular cardiomyopathy (ARVC) | PMID: 19214409 PMID: 18522797 PMID: 17064792 |
| Asthma | PMID: 21244684 PMID: 21239387 PMID: 21228877 PMID: 21219601 PMID: 21209446 PMID: 21194659 PMID: 21188255 PMID: 21180303 PMID: 21168814 PMID: 21165527 PMID: 21146349 PMID: 21142936 PMID: 21141063 PMID: 21139050 PMID: 21128938 PMID: 21128528 PMID: 21121205 PMID: 21092218 PMID: 21089181 PMID: 21085040 PMID: 21084071 PMID: 21076915 PMID: 21074553 PMID: 21062639 PMID: 21061110 PMID: 21050910 PMID: 21046521 PMID: 21045419 PMID: 21033494 PMID: 21030373 PMID: 20979928 PMID: 20977421 PMID: 20975725 PMID: 20975529 PMID: 20930909 PMID: 20929469 PMID: 20925457 PMID: 20923801 PMID: 20880427 PMID: 20873263 PMID: 20865673 PMID: 20857434 PMID: 20851926 PMID: 20849369 PMID: 20836340 PMID: 20833255 PMID: 20832903 PMID: 20823351 PMID: 20822990 PMID: 20819708 PMID: 20812490 PMID: 20810049 PMID: 20808667 PMID: 20801416 PMID: 20798160 PMID: 20704548 PMID: 20700554 PMID: 20694180 PMID: 20693905 PMID: 20689127 PMID: 20684656 PMID: 20680482 PMID: 20675031 PMID: 20666724 PMID: 20649076 PMID: 20646317 PMID: 20630807 PMID: 20627736 PMID: 20156741 PMID: 20614187 PMID: 20604676 PMID: 20598688 PMID: 20587104 PMID: 20584522 PMID: 20575236 PMID: 20556682 PMID: 20554500 PMID: 20544049 PMID: 20538198 PMID: 20516306 PMID: 20509891 PMID: 20504017 PMID: 20499695 PMID: 20497559 PMID: 20490283 PMID: 20464615 PMID: 20465035 PMID: 20458132 PMID: 20448538 PMID: 20414074 PMID: 20413242 PMID: 20405656 PMID: 20393884 PMID: 20380359 PMID: 20367774 PMID: 20356986 PMID: 20356408 PMID: 20350462 PMID: 20350267 PMID: 20339873 PMID: 20309776 PMID: 20230970 PMID: 20228001 PMID: 20226955 PMID: 20223919 PMID: 20219319 PMID: 20184717 PMID: 20177448 PMID: 20171533 PMID: 20164795 PMID: 20164501 PMID: 20158961 PMID: 20156213 PMID: 20154078 PMID: 20148609 PMID: 20143177 PMID: 20142575 PMID: 20134401 PMID: 20128135 PMID: 20099251 PMID: 20091554 PMID: 20091486 PMID: 20083045 PMID: 20079280 PMID: 20075855 PMID: 20073549 PMID: 20052862 PMID: 20043505 PMID: 20039157 PMID: 20035097 PMID: 20011602 PMID: 20009304 PMID: 20007497 PMID: 20003336 PMID: 19995140 PMID: 19966960 PMID: 19961985 PMID: 19953986 PMID: 19952160 PMID: 19947862 PMID: 19942797 PMID: 19940092 PMID: 19938725 PMID: 19933375 PMID: 19887811 PMID: 19878492 PMID: 19852550 PMID: 19851300 PMID: 19849760 PMID: 19847732 PMID: 19815673 PMID: 19798059 PMID: 19784520 PMID: 19780368 PMID: 19769012 PMID: 19765672 PMID: 19765426 PMID: 19748869 PMID: 19740085 PMID: 19738642 PMID: 19731150 PMID: 19730395 PMID: 19710438 PMID: 19696388 PMID: 19690384 PMID: 19689355 PMID: 19687219 PMID: 19672159 PMID: 19670966 PMID: 19656444 PMID: 19648129 PMID: 19642736 PMID: 19642439 PMID: 19641087 PMID: 19638367 PMID: 19635314 PMID: 19629026 PMID: 19620024 PMID: 19616137 PMID: 19593155 PMID: 19588042 PMID: 19587225 PMID: 19568869 PMID: 19564096 PMID: 19562288 PMID: 19560371 PMID: 19555610 PMID: 19546098 PMID: 19537230 PMID: 19531529 PMID: 19514298 PMID: 19508395 PMID: 19496364 PMID: 19486197 PMID: 19484681 PMID: 19484665 PMID: 19480377 PMID: 19472059 PMID: 19462613 PMID: 19449146 PMID: 19445271 PMID: 19442583 PMID: 19440499 PMID: 19426604 PMID: 19423314 PMID: 19422376 PMID: 19421709 PMID: 19400687 PMID: 19393838 PMID: 19390491 PMID: 19387519 PMID: 19384123 PMID: 19368569 PMID: 19365604 PMID: 19352305 PMID: 19336951 PMID: 19336439 PMID: 19331277 PMID: 19318680 PMID: 19317361 PMID: 19301933 PMID: 19297247 PMID: 19289392 PMID: 19278479 PMID: 19269877 PMID: 19263156 PMID: 19259552 PMID: 19243828 PMID: 19243624 PMID: 19229984 PMID: 19226283 PMID: 19222603 PMID: 19221969 PMID: 19204381 PMID: 19199210 PMID: 19197264 PMID: 19196365 PMID: 19193494 PMID: 19188657 PMID: 19168697 PMID: 19164428 PMID: 19160774 PMID: 19156751 PMID: 19155195 PMID: 19145832 PMID: 19130149 PMID: 19124369 PMID: 19119794 PMID: 21119973 PMID: 19112110 PMID: 19111150 PMID: 19104962 PMID: 19096513 PMID: 19064241 PMID: 19058239 PMID: 19055665 PMID: 19052938 PMID: 18987275 PMID: 19048005 PMID: 19036221 PMID: 19035231 PMID: 19029235 PMID: 19019517 PMID: 19011675 PMID: 19010061 PMID: 18998210 PMID: 18992253 PMID: 18991648 PMID: 18987584 PMID: 18972274 PMID: 18951067 PMID: 18843931 PMID: 18840374 PMID: 18838067 PMID: 18831745 PMID: 18830522 PMID: 18822854 PMID: 18819771 PMID: 18811605 PMID: 18805479 PMID: 18801798 PMID: 18798723 PMID: 18794683 PMID: 18774423 PMID: 18754258 PMID: 18728177 PMID: 18716689 PMID: 18713510 PMID: 18712093 PMID: 18704464 PMID: 18690353 PMID: 18678844 PMID: 18669791 PMID: 18651193 PMID: 18642124 PMID: 18637981 PMID: 18637769 PMID: 18611969 PMID: 18602496 PMID: 18501314 PMID: 18582390 PMID: 18579210 PMID: 18568054 PMID: 18564027 PMID: 18559086 PMID: 18528598 PMID: 18514583 PMID: 18494753 PMID: 18475096 PMID: 18468486 PMID: 18467528 PMID: 18454794 PMID: 18450658 PMID: 18440968 PMID: 18437360 PMID: 18420311 PMID: 18416079 PMID: 18408658 PMID: 18405959 PMID: 18403881 PMID: 18398631 PMID: 19999485 PMID: 18369671 PMID: 18368806 PMID: 18361259 PMID: 18341861 PMID: 18338965 PMID: 18328650 PMID: 18320579 PMID: 18297432 PMID: 18292674 PMID: 18292218 PMID: 18279472 PMID: 18219771 PMID: 18202916 PMID: 18202907 PMID: 18198791 PMID: 18198081 PMID: 18193180 PMID: 18188012 PMID: 18183329 PMID: 19823661 PMID: 19675731 PMID: 19450069 PMID: 18157997 PMID: 18097862 PMID: 18095969 PMID: 18082006 PMID: 18075488 PMID: 18070293 PMID: 18026809 PMID: 18048798 PMID: 18038580 PMID: 18034851 PMID: 18030180 PMID: 18022133 PMID: 18019794 PMID: 18018626 PMID: 18005055 PMID: 18001162 PMID: 17987508 PMID: 17982012 PMID: 17972534 PMID: 17921462 PMID: 17954865 PMID: 17926985 PMID: 17925629 PMID: 17916102 PMID: 17906365 PMID: 17904981 PMID: 17904940 PMID: 17890477 PMID: 17883922 PMID: 17848560 PMID: 17786807 PMID: 17786138 PMID: 17784724 PMID: 17766180 PMID: 17764971 PMID: 17760976 PMID: 17721642 PMID: 17708488 PMID: 17705158 PMID: 17697321 PMID: 17682236 PMID: 17680298 PMID: 17544021 PMID: 17671038 PMID: 17646029 PMID: 17620460 PMID: 17616645 PMID: 17610018 PMID: 19804018 PMID: 17590189 PMID: 17584003 PMID: 17582782 PMID: 17578536 PMID: 17570084 PMID: 17568517 PMID: 17567238 PMID: 17563978 PMID: 17557479 PMID: 17550669 PMID: 17546500 PMID: 17545672 PMID: 17534741 PMID: 17524092 PMID: 17514526 PMID: 17514358 PMID: 17495793 PMID: 17495374 PMID: 17492234 PMID: 17483561 PMID: 17474948 PMID: 17472762 PMID: 17470017 PMID: 17459784 PMID: 17450422 PMID: 17446057 PMID: 17444181 PMID: 17442118 PMID: 17435266 PMID: 17430765 PMID: 17407107 PMID: 17406553 PMID: 17406550 PMID: 17400877 PMID: 17392129 PMID: 17388112 PMID: 17386905 PMID: 17385469 PMID: 17384899 PMID: 17370439 PMID: 17366357 PMID: 17365199 PMID: 17359568 PMID: 17353636 PMID: 17350086 PMID: 17337804 PMID: 17329699 PMID: 17317702 PMID: 17306270 PMID: 17298337 PMID: 17294879 PMID: 17286919 PMID: 17284698 PMID: 17283305 PMID: 17273558 PMID: 17272783 PMID: 17264322 PMID: 17244672 PMID: 17242451 PMID: 17205090 PMID: 17202967 PMID: 17199843 PMID: 17198758 PMID: 19075874 PMID: 17188453 PMID: 17172692 PMID: 17166573 PMID: 17163314 PMID: 19385836 PMID: 17132349 PMID: 17127971 PMID: 17099334 PMID: 17083971 PMID: 17078421 PMID: 17064234 PMID: 17064232 PMID: 17059740 PMID: 17059600 PMID: 17058529 PMID: 17045272 PMID: 17044926 PMID: 17015204 PMID: 17011898 PMID: 16998092 PMID: 16963533 PMID: 16961747 PMID: 16937916 PMID: 16937765 PMID: 16926936 PMID: 16926453 PMID: 16925332 PMID: 16925217 PMID: 16924164 PMID: 16921779 PMID: 16912249 PMID: 16906279 PMID: 16897116 PMID: 16893334 PMID: 16888289 PMID: 16888146 PMID: 16883084 PMID: 16875149 PMID: 16874722 PMID: 16870965 PMID: 16846553 PMID: 16846397 PMID: 16842858 PMID: 16842696 PMID: 16839482 PMID: 16830504 PMID: 16827957 PMID: 16822303 PMID: 16818501 PMID: 16816059 PMID: 16809245 PMID: 16809243 PMID: 16800199 PMID: 16790320 PMID: 16779465 PMID: 16775906 PMID: 16774877 PMID: 16750832 PMID: 16739367 PMID: 16737666 PMID: 16728650 PMID: 16716097 PMID: 16714488 PMID: 16713785 PMID: 16648391 PMID: 16643702 PMID: 16636683 PMID: 16617001 PMID: 16615674 PMID: 16613348 PMID: 16598992 PMID: 16582677 PMID: 16582132 PMID: 16580288 PMID: 16570707 PMID: 16563080 PMID: 16550711 PMID: 16548624 PMID: 16545267 PMID: 16539787 PMID: 16534298 PMID: 16532166 PMID: 16528016 PMID: 16496290 PMID: 16474262 PMID: 16458289 PMID: 16451303 PMID: 16450748 PMID: 16449782 PMID: 16445708 PMID: 16442916 PMID: 16440857 PMID: 16434223 PMID: 16429272 PMID: 16425926 PMID: 16416887 PMID: 16416807 PMID: 16409014 PMID: 16403941 PMID: 16392399 PMID: 16389020 PMID: 16379054 PMID: 16373194 PMID: 16372367 PMID: 16339291 PMID: 16335644 PMID: 16324036 PMID: 16318973 PMID: 16296793 PMID: 16296575 PMID: 16281689 PMID: 16279269 PMID: 16277741 PMID: 16265563 PMID: 16259723 PMID: 16259511 PMID: 16257231 PMID: 16247529 PMID: 16236966 PMID: 16231773 PMID: 16231770 PMID: 16226198 PMID: 16218226 PMID: 16208311 PMID: 16207641 PMID: 16190176 PMID: 16188400 PMID: 16142389 PMID: 16141109 PMID: 16140618 PMID: 16135175 PMID: 16128540 PMID: 16120741 PMID: 16114227 PMID: 16111439 PMID: 16053643 PMID: 16052980 PMID: 16028566 PMID: 16005610 PMID: 15989409 PMID: 15983239 PMID: 15974215 PMID: 15953132 PMID: 15946454 PMID: 15935198 PMID: 15928401 PMID: 15926759 PMID: 15911456 PMID: 15893131 PMID: 15889563 PMID: 15888843 PMID: 15881965 PMID: 15881095 PMID: 15880960 PMID: 15847005 PMID: 15826467 PMID: 15816505 PMID: 15814164 PMID: 15810441 PMID: 15804132 PMID: 15794198 PMID: 15784059 PMID: 15782448 PMID: 15753484 PMID: 15750178 PMID: 15747907 PMID: 15747574 PMID: 15743882 PMID: 15737978 PMID: 15732714 PMID: 15724924 PMID: 15724895 PMID: 15717482 PMID: 15704699 PMID: 15701486 PMID: 15687512 PMID: 15682958 PMID: 15673950 PMID: 15671633 PMID: 15659480 PMID: 15655881 PMID: 15655528 PMID: 15649101 PMID: 15648399 PMID: 15639490 PMID: 15636311 PMID: 15635486 PMID: 15629304 PMID: 15624788 PMID: 15624195 PMID: 15617881 PMID: 15611893 PMID: 15608802 PMID: 15603272 PMID: 15597357 PMID: 15579081 PMID: 15576897 PMID: 15568267 PMID: 15552241 PMID: 15546394 PMID: 15536492 PMID: 15529942 PMID: 15525454 PMID: 15520110 PMID: 15518884 PMID: 15507333 PMID: 15487387 PMID: 15487110 PMID: 15480107 PMID: 15471207 PMID: 15465987 PMID: 21218585 PMID: 15362955 PMID: 15353323 PMID: 15349514 PMID: 15346935 PMID: 15330878 PMID: 15329968 PMID: 15328327 PMID: 15321395 PMID: 15314468 PMID: 15302634 PMID: 15300966 PMID: 15283229 PMID: 15266510 PMID: 15256480 PMID: 15233312 PMID: 15208581 PMID: 15204789 PMID: 15202030 PMID: 15193367 PMID: 15192624 PMID: 15162259 PMID: 15152765 PMID: 15134516 PMID: 15131088 PMID: 15125428 PMID: 15114772 PMID: 15114276 PMID: 15113718 PMID: 15106507 PMID: 15103311 PMID: 15087211 PMID: 15080082 PMID: 15058158 PMID: 15025889 PMID: 14996097 PMID: 14987333 PMID: 14977544 PMID: 14764810 PMID: 14748897 PMID: 14733762 PMID: 14727379 PMID: 14724718 PMID: 14720074 PMID: 14718331 PMID: 14689111 PMID: 14681260 PMID: 14665811 PMID: 14660612 PMID: 14655768 PMID: 14652111 PMID: 14647562 PMID: 14647973 PMID: 14634861 PMID: 14630383 PMID: 14601956 PMID: 14601942 PMID: 14596356 PMID: 14594343 PMID: 14592322 PMID: 14581654 PMID: 14578971 PMID: 14532367 PMID: 14528027 PMID: 14513541 PMID: 14513527 PMID: 14506307 PMID: 13679007 PMID: 12971492 PMID: 12968097 PMID: 12966799 PMID: 12940593 PMID: 12940322 PMID: 12937626 PMID: 12934874 PMID: 12882854 PMID: 12870235 PMID: 12869771 PMID: 12869295 PMID: 12867391 PMID: 12865722 PMID: 12860749 PMID: 12858133 PMID: 12854627 PMID: 12831656 PMID: 12808765 PMID: 12808278 PMID: 12796572 PMID: 12765320 PMID: 12765319 PMID: 12749763 PMID: 12742804 PMID: 12741352 PMID: 12735662 PMID: 12726866 PMID: 12717323 PMID: 12716786 PMID: 12712039 PMID: 12685233 PMID: 12676466 PMID: 12674586 PMID: 12661439 PMID: 12638407 PMID: 12633801 PMID: 12611269 PMID: 12601535 PMID: 12592248 PMID: 12592057 PMID: 12585752 PMID: 12579511 PMID: 12577845 PMID: 12570760 PMID: 12559191 PMID: 12555048 PMID: 12535498 PMID: 12527626 PMID: 12527352 PMID: 12527343 PMID: 12519582 PMID: 12517512 PMID: 12511719 PMID: 12503980 PMID: 12491775 PMID: 12490643 PMID: 12482208 PMID: 12471841 PMID: 12466048 PMID: 12463055 PMID: 12452751 PMID: 12444870 PMID: 12448939 PMID: 12448387 PMID: 12448269 PMID: 12421219 PMID: 12416945 PMID: 12416087 PMID: 12396636 PMID: 12395147 PMID: 12392119 PMID: 12391520 PMID: 12370004 PMID: 12353595 PMID: 12353390 PMID: 12242177 PMID: 12241531 PMID: 12240844 PMID: 12232928 PMID: 12229254 PMID: 12227118 PMID: 12210638 PMID: 12199681 PMID: 12171558 PMID: 12162864 PMID: 12113643 PMID: 12090053 PMID: 12084001 PMID: 12077613 PMID: 12076486 PMID: 12070556 PMID: 12069502 PMID: 12066970 PMID: 12063644 PMID: 12048404 PMID: 12030637 PMID: 12030626 PMID: 12003770 PMID: 12003159 PMID: 12001885 PMID: 11996210 PMID: 11980126 PMID: 11967714 PMID: 11967432 PMID: 11966453 PMID: 11965822 PMID: 19475216 PMID: 11910609 PMID: 11898315 PMID: 11894847 PMID: 11889333 PMID: 11887620 PMID: 11879559 PMID: 11869646 PMID: 11863258 PMID: 11862064 PMID: 11845767 PMID: 11843995 PMID: 11842287 PMID: 11838870 PMID: 11822540 PMID: 11816664 PMID: 11811855 PMID: 11796456 PMID: 11788092 PMID: 11787411 PMID: 11783250 PMID: 11780716 PMID: 11769437 PMID: 11763246 PMID: 11758894 PMID: 11740851 PMID: 11731716 PMID: 11729389 PMID: 11702579 PMID: 11676126 PMID: 11642684 PMID: 11593152 PMID: 11585189 PMID: 11584939 PMID: 11563816 PMID: 11535263 PMID: 11533422 PMID: 11529288 PMID: 11515813 PMID: 11486469 PMID: 11468136 PMID: 11434792 PMID: 11457745 PMID: 11453346 PMID: 11433630 PMID: 11422957 PMID: 11416614 PMID: 11406056 PMID: 11405609 PMID: 11395153 PMID: 11392264 PMID: 11379468 PMID: 11372012 PMID: 11364696 PMID: 11355718 PMID: 11345692 PMID: 11344633 PMID: 11338156 PMID: 11335183 PMID: 11322094 PMID: 11320213 PMID: 11315078 PMID: 11281235 PMID: 11279875 PMID: 11246585 PMID: 11223326 PMID: 11184893 PMID: 11183415 PMID: 11208627 PMID: 11174486 PMID: 11171680 PMID: 11165553 PMID: 11139004 PMID: 11137489 PMID: 11136265 PMID: 11122298 PMID: 11101175 PMID: 11093300 PMID: 11080708 PMID: 11080007 PMID: 11079911 PMID: 11079856 PMID: 11054247 PMID: 11051735 PMID: 11049148 PMID: 11044544 PMID: 11040602 PMID: 11031335 PMID: 11015800 PMID: 11008001 PMID: 11001172 PMID: 10977443 PMID: 10976856 PMID: 10975434 PMID: 10967865 PMID: 10949880 PMID: 10945376 PMID: 10940788 PMID: 10938756 PMID: 10910412 PMID: 10892385 PMID: 10885082 PMID: 10873149 PMID: 10871321 PMID: 10845442 PMID: 10796542 PMID: 10786347 PMID: 10780271 PMID: 10776373 PMID: 10771982 PMID: 10758080 PMID: 10746738 PMID: 10741778 PMID: 10730452 PMID: 10730026 PMID: 10730840 PMID: 10727669 PMID: 10721395 PMID: 10707918 PMID: 10691060 PMID: 10687208 PMID: 10684210 PMID: 10672135 PMID: 10669710 PMID: 10658301 PMID: 10641045 PMID: 10634992 PMID: 10632281 PMID: 10618941 PMID: 10606977 PMID: 10597094 PMID: 10565623 PMID: 10550745 PMID: 10549233 PMID: 10539224 PMID: 10538452 PMID: 10537955 PMID: 10537868 PMID: 10528279 PMID: 10496643 PMID: 10492750 PMID: 10479465 PMID: 10471019 PMID: 10467822 PMID: 10467627 PMID: 10466907 PMID: 10460748 PMID: 10460745 PMID: 10444954 PMID: 10442091 PMID: 10439581 PMID: 10436870 PMID: 10414557 PMID: 10394644 PMID: 10385358 PMID: 10384665 PMID: 10363142 PMID: 10361479 PMID: 10326988 PMID: 10326936 PMID: 10322699 PMID: 10321860 PMID: 10234766 PMID: 10217666 PMID: 10212856 PMID: 10211540 PMID: 10195079 PMID: 10193531 PMID: 10191559 PMID: 10097242 PMID: 10087910 PMID: 10081393 PMID: 10079647 PMID: 10078166 PMID: 10070673 PMID: 10065341 PMID: 10065336 PMID: 9988276 PMID: 9924639 PMID: 9918746 PMID: 9917180 PMID: 9917078 PMID: 9892818 PMID: 9844886 PMID: 9842078 PMID: 9825082 PMID: 9817732 PMID: 9813738 PMID: 9792347 PMID: 9769998 PMID: 9748883 PMID: 9748792 PMID: 9731696 PMID: 9722579 PMID: 9722370 PMID: 9721785 PMID: 9711442 PMID: 9711043 PMID: 9706414 PMID: 9689690 PMID: 9680494 PMID: 9673832 PMID: 9671899 PMID: 9660321 PMID: 9650340 PMID: 9649093 PMID: 9643314 PMID: 9624315 PMID: 9623692 PMID: 9615296 PMID: 9564659 PMID: 9604586 PMID: 9542008 PMID: 9580925 PMID: 9569445 PMID: 9555175 PMID: 9551698 PMID: 9539009 PMID: 9532537 PMID: 9511016 PMID: 9504293 PMID: 9494802 PMID: 9474368 PMID: 9472211 PMID: 9465849 PMID: 9430989 PMID: 9450840 PMID: 9413700 PMID: 9375944 PMID: 9400700 PMID: 9392980 PMID: 9390920 PMID: 9307092 PMID: 9567402 PMID: 9431757 PMID: 9247850 PMID: 9424823 PMID: 9340027 PMID: 9333988 PMID: 9228910 PMID: 9212760 PMID: 9200624 PMID: 9220922 PMID: 10184796 PMID: 9218185 PMID: 9196329 PMID: 9174496 PMID: 9263668 PMID: 9245208 PMID: 9161656 PMID: 9143858 PMID: 9192541 PMID: 9264658 PMID: 10174892 PMID: 9127645 PMID: 10868186 PMID: 9565725 PMID: 9340042 PMID: 9263758 PMID: 9183782 PMID: 9139510 PMID: 9137044 PMID: 9104737 PMID: 9083713 PMID: 9056046 PMID: 9054904 PMID: 9046223 PMID: 9283340 PMID: 8997046 PMID: 8968445 PMID: 8948666 PMID: 8992674 PMID: 9363193 PMID: 8874457 PMID: 8865786 PMID: 8841195 PMID: 8883524 PMID: 8870879 PMID: 9251427 PMID: 8938500 PMID: 8921463 PMID: 8709661 PMID: 10159579 PMID: 8928424 PMID: 8684756 PMID: 8646146 PMID: 8856111 PMID: 8762744 PMID: 8966157 PMID: 8880948 PMID: 8666864 PMID: 8622277 PMID: 9012180 PMID: 8948884 PMID: 8876755 PMID: 8805803 PMID: 8739884 PMID: 8632507 PMID: 8693216 PMID: 8681060 PMID: 8553311 PMID: 9363086 PMID: 8903898 PMID: 8692160 PMID: 8573991 PMID: 7481316 PMID: 8762487 PMID: 8529590 PMID: 7555128 PMID: 8928631 PMID: 8846718 PMID: 7583080 PMID: 7494742 PMID: 7487830 PMID: 7609259 PMID: 8576591 PMID: 7667488 PMID: 7672896 PMID: 7661094 PMID: 7473537 PMID: 7569498 PMID: 7780531 PMID: 7697248 PMID: 7754314 PMID: 7724948 PMID: 7701490 PMID: 7624274 PMID: 7554410 PMID: 10136809 PMID: 7886371 PMID: 7862403 PMID: 9584792 PMID: 8779100 PMID: 8676357 PMID: 8591533 PMID: 8577113 PMID: 8521087 PMID: 7874464 PMID: 7813294 PMID: 7800328 PMID: 7770803 PMID: 7759168 PMID: 7990241 PMID: 7659919 PMID: 8000695 PMID: 7836621 PMID: 7916004 PMID: 7836103 PMID: 7826819 PMID: 7811786 PMID: 7995270 PMID: 7964339 PMID: 7948845 PMID: 8089876 PMID: 8020306 PMID: 7936357 PMID: 7923744 PMID: 7918816 PMID: 7819730 PMID: 7919552 PMID: 7852243 PMID: 7937607 PMID: 8004835 PMID: 7854988 PMID: 8166149 PMID: 7516861 PMID: 18415455 PMID: 8017040 PMID: 8301774 PMID: 8009483 PMID: 8177860 PMID: 8164243 PMID: 7508323 PMID: 8131179 PMID: 8298346 PMID: 8201828 PMID: 8179974 PMID: 8092784 PMID: 8092781 PMID: 7911649 PMID: 7900058 PMID: 7800957 PMID: 7533226 PMID: 8158343 PMID: 8120506 PMID: 8109229 PMID: 8293764 PMID: 8288071 PMID: 8112452 PMID: 8404181 PMID: 8039818 PMID: 8362695 PMID: 8211298 PMID: 8038862 PMID: 10146924 PMID: 8366566 PMID: 8393579 PMID: 8373314 PMID: 8276499 PMID: 8105598 PMID: 8494195 PMID: 8353422 PMID: 8098790 PMID: 8501991 PMID: 8498428 PMID: 8405636 PMID: 8209475 PMID: 8508344 PMID: 8378653 PMID: 8487929 PMID: 8482511 PMID: 8452906 PMID: 8387049 PMID: 8465532 PMID: 8428511 PMID: 8355554 PMID: 8272476 PMID: 8211799 PMID: 8094084 PMID: 8059397 PMID: 1460937 PMID: 1491150 PMID: 1360775 PMID: 1464896 PMID: 1519635 PMID: 1509607 PMID: 1382018 PMID: 1511110 PMID: 1599349 PMID: 1419243 PMID: 1319811 PMID: 1348344 PMID: 1609268 PMID: 1573238 PMID: 1560165 PMID: 1535127 PMID: 1573346 PMID: 1569731 PMID: 1568843 PMID: 1374320 PMID: 1350235 PMID: 1535954 PMID: 1346201 PMID: 1632246 PMID: 1617511 PMID: 1604029 PMID: 1514305 PMID: 1462882 PMID: 1363672 PMID: 1348373 PMID: 1313266 PMID: 1298833 PMID: 1283428 PMID: 1725154 PMID: 1797323 PMID: 1669606 PMID: 1957073 PMID: 1928973 PMID: 1837707 PMID: 1742826 PMID: 1839919 PMID: 1792481 PMID: 1946860 PMID: 1838781 PMID: 1876799 PMID: 2041508 PMID: 2042683 PMID: 1886531 PMID: 1839420 PMID: 2020218 PMID: 1929175 PMID: 2014434 PMID: 2009000 PMID: 1855576 PMID: 1671192 PMID: 1986288 PMID: 1916615 PMID: 1844555 PMID: 1844423 PMID: 1805415 PMID: 1792813 PMID: 1686977 PMID: 1365389 PMID: 2272077 PMID: 2240820 PMID: 2274568 PMID: 2209137 PMID: 1977116 PMID: 2262253 PMID: 2255278 PMID: 2223429 PMID: 2120771 PMID: 2376093 PMID: 1699664 PMID: 2388184 PMID: 2198911 PMID: 2189098 PMID: 2275174 PMID: 2235103 PMID: 2183868 PMID: 2301091 PMID: 11527141 PMID: 2368003 PMID: 2293752 PMID: 2274322 PMID: 2176522 PMID: 2095310 PMID: 2075319 PMID: 1691400 PMID: 1688983 PMID: 2689066 PMID: 2575174 PMID: 2696137 PMID: 2625958 PMID: 2508951 PMID: 2801484 PMID: 2571293 PMID: 2789974 PMID: 2682132 PMID: 2764016 PMID: 2569823 PMID: 2762946 PMID: 2756975 PMID: 2670511 PMID: 2663536 PMID: 2743542 PMID: 2675379 PMID: 10293579 PMID: 2537709 PMID: 2524306 PMID: 2713039 PMID: 2712281 PMID: 2645630 PMID: 2913777 PMID: 2772358 PMID: 2772247 PMID: 2740930 PMID: 2740929 PMID: 2695911 PMID: 2678323 PMID: 2562825 PMID: 2520392 PMID: 2483571 PMID: 3075287 PMID: 3059177 PMID: 3190143 PMID: 3171009 PMID: 3205192 PMID: 3063110 PMID: 3048940 PMID: 2900659 PMID: 3272553 PMID: 3202400 PMID: 2850403 PMID: 3293726 PMID: 3140230 PMID: 3226975 PMID: 3365073 PMID: 3171120 PMID: 2835029 PMID: 3393124 PMID: 2908432 PMID: 2963522 PMID: 2827838 PMID: 3414089 PMID: 3410657 PMID: 3388279 PMID: 3276988 PMID: 3210118 PMID: 3207233 PMID: 3162631 PMID: 3154666 PMID: 3075403 PMID: 3072451 PMID: 3067409 PMID: 3042360 PMID: 2975610 PMID: 2975609 PMID: 2905817 PMID: 2831595 PMID: 2972478 PMID: 3658275 PMID: 3434324 PMID: 3632373 PMID: 3310776 PMID: 3669560 PMID: 3624700 PMID: 2980908 PMID: 3306268 PMID: 3578924 PMID: 2908417 PMID: 2837699 PMID: 3549116 PMID: 3135819 PMID: 3312597 PMID: 2433927 PMID: 3603390 PMID: 3603388 PMID: 3578944 PMID: 3563907 PMID: 3454388 PMID: 3333283 PMID: 3113825 PMID: 2455149 PMID: 2454370 PMID: 3295816 PMID: 3825053 PMID: 3022582 PMID: 3534631 PMID: 3762158 PMID: 3752178 PMID: 3750125 PMID: 3537902 PMID: 3784478 PMID: 3092700 PMID: 3525084 PMID: 2943654 PMID: 2873109 PMID: 3704872 PMID: 3538396 PMID: 3515982 PMID: 3961966 PMID: 3946713 PMID: 3825366 PMID: 3538781 PMID: 3317340 PMID: 3317334 PMID: 3099590 PMID: 3089849 PMID: 2878401 PMID: 2872771 PMID: 2868819 PMID: 2439803 PMID: 2427862 PMID: 2427852 PMID: 2427851 PMID: 2427837 PMID: 2427836 PMID: 2427835 PMID: 3905285 PMID: 2866069 PMID: 3853209 PMID: 4053724 PMID: 2872604 PMID: 2868082 PMID: 2931840 PMID: 3899468 PMID: 3928056 PMID: 3853652 PMID: 3898359 PMID: 4013994 PMID: 2862980 PMID: 3840215 PMID: 2864061 PMID: 2864058 PMID: 3900735 PMID: 2859777 PMID: 3923251 PMID: 2988221 PMID: 3989775 PMID: 3836298 PMID: 3836297 PMID: 12280585 PMID: 3969544 PMID: 4002165 PMID: 3904331 PMID: 3891370 PMID: 2989986 PMID: 2866859 PMID: 2420157 PMID: 6152089 PMID: 6507943 PMID: 6150479 PMID: 6517234 PMID: 6744571 PMID: 6482022 PMID: 6380352 PMID: 6088618 PMID: 6375615 PMID: 6145093 PMID: 6375888 PMID: 6510320 PMID: 6377247 PMID: 6741154 PMID: 6732146 PMID: 6704008 PMID: 6366453 PMID: 6232756 PMID: 6141720 PMID: 6524189 PMID: 6515699 PMID: 6364879 PMID: 6363358 PMID: 6151891 PMID: 6084132 PMID: 6685194 PMID: 6139010 PMID: 6652572 PMID: 6359750 PMID: 6884104 PMID: 6615706 PMID: 6310529 PMID: 6135714 PMID: 6860529 PMID: 6865186 PMID: 6865182 PMID: 6305618 PMID: 6129953 PMID: 6868938 PMID: 6844370 PMID: 6622629 PMID: 6552920 PMID: 6218610 PMID: 7154635 PMID: 6126725 PMID: 7147886 PMID: 7134688 PMID: 7122444 PMID: 7113145 PMID: 6212087 PMID: 7146936 PMID: 7140799 PMID: 7101191 PMID: 7090530 PMID: 7087606 PMID: 7075644 PMID: 6958494 PMID: 6896920 PMID: 6977078 PMID: 6797777 PMID: 7301197 PMID: 7307505 PMID: 7023221 PMID: 7025452 PMID: 7021826 PMID: 6452067 PMID: 6111068 PMID: 7269538 PMID: 7256601 PMID: 7256075 PMID: 7032947 PMID: 6971975 PMID: 6935712 PMID: 6108617 PMID: 7444697 PMID: 6969987 PMID: 7416357 PMID: 7471827 PMID: 6771626 PMID: 7402591 PMID: 7381884 PMID: 6994164 PMID: 6256830 PMID: 7382366 PMID: 7355894 PMID: 7355327 PMID: 7374433 PMID: 542264 PMID: 574785 PMID: 464779 PMID: 440931 PMID: 378050 PMID: 38096 PMID: 310707 PMID: 758809 PMID: 550177 PMID: 424862 PMID: 374041 PMID: 225832 PMID: 310196 PMID: 31260 PMID: 707790 PMID: 659609 PMID: 387967 PMID: 350512 PMID: 25708 PMID: 651889 PMID: 351429 PMID: 209529 PMID: 306038 PMID: 648102 PMID: 351350 PMID: 351103 PMID: 414904 PMID: 725770 PMID: 724265 PMID: 680553 PMID: 356445 PMID: 203996 PMID: 604968 PMID: 337071 PMID: 269508 PMID: 562303 PMID: 334309 PMID: 896074 PMID: 334677 PMID: 576722 PMID: 557763 PMID: 343894 PMID: 594895 PMID: 592224 PMID: 577484 PMID: 264317 PMID: 12653 PMID: 1020411 PMID: 825056 PMID: 10730 PMID: 1013666 PMID: 951414 PMID: 1267503 PMID: 7858 PMID: 1252670 PMID: 1263159 PMID: 1263158 PMID: 1007853 PMID: 1001000 PMID: 964029 PMID: 787954 PMID: 135327 PMID: 1228169 PMID: 1216986 PMID: 1229122 PMID: 1081290 PMID: 1173907 PMID: 1156110 PMID: 812443 PMID: 808693 PMID: 1162018 PMID: 1153360 PMID: 1124859 PMID: 1124452 PMID: 1054448 PMID: 164200 PMID: 1110293 PMID: 1231281 PMID: 1198087 PMID: 1119239 PMID: 4373633 PMID: 4612231 PMID: 4414820 PMID: 4372582 PMID: 4606956 PMID: 4408728 PMID: 4609171 PMID: 4855055 PMID: 4842014 PMID: 4832681 PMID: 4838744 PMID: 4611742 PMID: 4606415 PMID: 4428383 PMID: 4282614 PMID: 4152167 PMID: 4595462 PMID: 4147920 PMID: 4126267 PMID: 4793909 PMID: 4711321 PMID: 4736191 PMID: 4144731 PMID: 4682991 PMID: 4790030 PMID: 4684067 PMID: 4581867 PMID: 4573887 PMID: 4647927 PMID: 4656089 PMID: 4342159 PMID: 4658132 PMID: 4403192 PMID: 5077685 PMID: 4677707 PMID: 5025095 PMID: 5044836 PMID: 4143623 PMID: 5118166 PMID: 5122466 PMID: 5093474 PMID: 5565912 PMID: 4937200 PMID: 5118265 PMID: 5560835 PMID: 5556102 PMID: 5108966 PMID: 5576107 PMID: 5103269 PMID: 5548969 PMID: 5154098 PMID: 4394708 PMID: 4097320 PMID: 4248929 PMID: 5315844 PMID: 5426713 PMID: 4316441 PMID: 4194133 PMID: 4916980 PMID: 5439543 PMID: 5468000 PMID: 4915309 PMID: 5452151 PMID: 4392557 PMID: 5520674 PMID: 5418347 PMID: 5313859 PMID: 5395591 PMID: 5377764 PMID: 5367893 PMID: 5346102 PMID: 4390395 PMID: 4900319 PMID: 5409036 PMID: 4306559 PMID: 4897341 PMID: 5807904 PMID: 4901729 PMID: 5785246 PMID: 4892569 PMID: 4989002 PMID: 5767155 PMID: 5375201 PMID: 5373248 PMID: 5354538 PMID: 5353428 PMID: 5716076 PMID: 5753012 PMID: 5304705 PMID: 4240912 PMID: 4879607 PMID: 5693584 PMID: 5703957 PMID: 4231820 PMID: 5646400 PMID: 4870738 PMID: 5653026 PMID: 5689221 PMID: 5668948 PMID: 5694842 PMID: 5662430 PMID: 5736988 PMID: 5728390 PMID: 6061296 PMID: 4873607 PMID: 4294925 PMID: 4227081 PMID: 4962446 PMID: 6028602 PMID: 6027074 PMID: 6072719 PMID: 6024246 PMID: 5618529 PMID: 6049287 PMID: 5232351 PMID: 4381226 PMID: 6070516 PMID: 6030788 PMID: 4300996 PMID: 5971528 PMID: 4231308 PMID: 5991397 PMID: 5962914 PMID: 5908675 PMID: 5952245 PMID: 5897572 PMID: 14282155 PMID: 14270996 PMID: 14266172 PMID: 14269697 PMID: 14213174 PMID: 15446184 PMID: 14231571 PMID: 14169416 PMID: 14201940 PMID: 14158485 PMID: 14104975 PMID: 14225764 PMID: 14173505 PMID: 14142448 PMID: 14136309 PMID: 14172754 PMID: 14144851 PMID: 14164799 PMID: 14162719 PMID: 14152172 PMID: 14132731 PMID: 14116771 PMID: 14102161 PMID: 14105482 PMID: 14093373 PMID: 14112034 PMID: 14090841 PMID: 14083502 PMID: 14120118 PMID: 14069145 PMID: 14076650 PMID: 14066337 PMID: 14132445 PMID: 14469409 PMID: 14458703 PMID: 13876615 PMID: 13760047 PMID: 13657596 PMID: 13580900 PMID: 13414067 PMID: 13442519 PMID: 13301164 PMID: 13210790 PMID: 13161430 PMID: 13176021 PMID: 13115153 PMID: 13112377 PMID: 13009503 PMID: 14945990 PMID: 14899075 PMID: 14876580 PMID: 15418028 PMID: 15409018 PMID: 15398441 PMID: 18885390 PMID: 20243226 |
| Autoimmune thyroid disease | PMID: 20845662 PMID: 20573783 PMID: 20375216 PMID: 20009390 PMID: 19474524 PMID: 18719369 PMID: 18218316 PMID: 16984944 PMID: 15813612 PMID: 15340865 PMID: 12524672 PMID: 12426269 PMID: 9837829 PMID: 9103950 PMID: 9075605 |
| Axon guidance | PMID: 20926682 PMID: 16540569 |
| B cell receptor signaling pathway |  |
| Basal cell carcinoma | PMID: 19751881 PMID: 19590411 PMID: 18347458 PMID: 17618177 PMID: 17057750 PMID: 16792769 PMID: 11578854 PMID: 10597976 PMID: 10449940 PMID: 1759057 PMID: 1891132 PMID: 2369568 PMID: 2806096 PMID: 3887060 PMID: 6572862 PMID: 223536 PMID: 972342 PMID: 5102629 PMID: 13995586 |
| Biosynthesis of unsaturated fatty acids | PMID: 21051399 PMID: 21041702 PMID: 20956731 PMID: 20943934 PMID: 20888525 PMID: 20861077 PMID: 20852048 PMID: 20846129 PMID: 20844610 PMID: 20837888 PMID: 20837886 PMID: 20833959 PMID: 20819225 PMID: 20691134 PMID: 20688085 PMID: 20674858 PMID: 20660124 PMID: 20659230 PMID: 20645173 PMID: 20628829 PMID: 20622163 PMID: 20622039 PMID: 20619278 PMID: 20610532 PMID: 20590572 PMID: 20583254 PMID: 20578283 PMID: 20561700 PMID: 20555328 PMID: 20546237 PMID: 20522807 PMID: 20520615 PMID: 20514986 PMID: 20512996 PMID: 20501636 PMID: 20499250 PMID: 20463040 PMID: 20460121 PMID: 20431527 PMID: 20418081 PMID: 20416876 PMID: 20415621 PMID: 20357747 PMID: 20357746 PMID: 20357745 PMID: 20379046 PMID: 20375903 PMID: 20364702 PMID: 20347779 PMID: 20333529 PMID: 20307663 PMID: 20307216 PMID: 20298545 PMID: 20212458 PMID: 20206489 PMID: 20203687 PMID: 20202290 PMID: 20194308 PMID: 20194297 PMID: 20194296 PMID: 20186125 PMID: 20182021 PMID: 20179619 PMID: 20164834 PMID: 20157051 PMID: 20142564 PMID: 20139904 PMID: 20132021 PMID: 20128814 PMID: 20120158 PMID: 20118222 PMID: 20107832 PMID: 20107114 PMID: 20093140 PMID: 20091049 PMID: 20079425 PMID: 20065148 PMID: 20065147 PMID: 20058612 PMID: 20051913 PMID: 20050826 PMID: 20046414 PMID: 20042103 PMID: 20041816 PMID: 20041812 PMID: 20038752 PMID: 20026767 PMID: 20018822 PMID: 20007352 PMID: 20004361 PMID: 20003820 PMID: 19954007 PMID: 19940786 PMID: 19940265 PMID: 19937318 PMID: 19929032 PMID: 19921237 PMID: 19915063 PMID: 19894275 PMID: 19893499 PMID: 19893496 PMID: 19864304 PMID: 19857569 PMID: 19838762 PMID: 19826183 PMID: 19825380 PMID: 19822802 PMID: 19822801 PMID: 19809463 PMID: 19797218 PMID: 19795383 PMID: 19789547 PMID: 19786728 PMID: 19786647 PMID: 19786646 PMID: 19776718 PMID: 19776641 PMID: 19770404 PMID: 19761780 PMID: 19746421 PMID: 19718606 PMID: 19718605 PMID: 19718604 PMID: 19717790 PMID: 19705518 PMID: 19698199 PMID: 19687342 PMID: 19686729 PMID: 19682899 PMID: 19675180 PMID: 19672123 PMID: 19668263 PMID: 19665690 PMID: 19660790 PMID: 19656915 PMID: 19652084 PMID: 19646544 PMID: 19633817 PMID: 19617407 PMID: 19609299 PMID: 19597033 PMID: 19592461 PMID: 19592458 PMID: 19592221 PMID: 19555855 PMID: 19581832 PMID: 19579269 PMID: 19577709 PMID: 19571576 PMID: 19566838 PMID: 19566753 PMID: 19564543 PMID: 19553934 PMID: 19535947 PMID: 19539780 PMID: 19539681 PMID: 19531939 PMID: 19527795 PMID: 19516159 PMID: 19506338 PMID: 19502719 PMID: 19496702 PMID: 19491705 PMID: 19491530 PMID: 19485927 PMID: 19483747 PMID: 19483043 PMID: 19474762 PMID: 19464961 PMID: 19462497 PMID: 19458537 PMID: 19445816 PMID: 19440924 PMID: 19437028 PMID: 19433775 PMID: 19430191 PMID: 19428232 PMID: 19422139 PMID: 19416858 PMID: 19414643 PMID: 19411610 PMID: 19404315 PMID: 19403858 PMID: 19397224 PMID: 19382469 PMID: 19380610 PMID: 19360315 PMID: 19356270 PMID: 19331646 PMID: 19303975 PMID: 19299433 PMID: 19289321 PMID: 19285604 PMID: 19280705 PMID: 19279555 PMID: 19269635 PMID: 19251782 PMID: 19226708 PMID: 19226702 PMID: 19225144 PMID: 19208832 PMID: 19196081 PMID: 19193725 PMID: 19188660 PMID: 19159454 PMID: 19147999 PMID: 19145780 PMID: 19138979 PMID: 19133994 PMID: 19100733 PMID: 19096033 PMID: 19086228 PMID: 19083388 PMID: 19077685 PMID: 19075484 PMID: 19075100 PMID: 19073906 PMID: 19038618 PMID: 19057444 PMID: 19049669 PMID: 19047578 PMID: 19046748 PMID: 19033012 PMID: 19021758 PMID: 19015135 PMID: 19013235 PMID: 19011675 PMID: 19008712 PMID: 18983226 PMID: 18978038 PMID: 18971891 PMID: 18971427 PMID: 18957819 PMID: 18952718 PMID: 18927469 PMID: 18843091 PMID: 18842817 PMID: 18842497 PMID: 18829737 PMID: 18827466 PMID: 18824658 PMID: 18802217 PMID: 18820028 PMID: 18819643 PMID: 18818375 PMID: 18812232 PMID: 18793165 PMID: 18772366 PMID: 18772174 PMID: 18771663 PMID: 18760760 PMID: 18726563 PMID: 18704488 PMID: 18693174 PMID: 18685064 PMID: 18684890 PMID: 18665807 PMID: 18663155 PMID: 18663153 PMID: 18657627 PMID: 18641268 PMID: 18635926 PMID: 18633173 PMID: 18632796 PMID: 18614742 PMID: 18606907 PMID: 18606903 PMID: 18596730 PMID: 18589711 PMID: 18574459 PMID: 18574070 PMID: 18572197 PMID: 18555214 PMID: 18547479 PMID: 18541602 PMID: 18535668 PMID: 18505546 PMID: 18296334 PMID: 18499529 PMID: 18490519 PMID: 18487433 PMID: 18471910 PMID: 18467507 PMID: 18451496 PMID: 18450829 PMID: 18441279 PMID: 18437998 PMID: 18437120 PMID: 18432131 PMID: 18430418 PMID: 18430060 PMID: 18430051 PMID: 18425706 PMID: 18398704 PMID: 18391113 PMID: 18387796 PMID: 18387761 PMID: 18378855 PMID: 18378784 PMID: 18378224 PMID: 18373394 PMID: 18356699 PMID: 18348729 PMID: 18344620 PMID: 18312493 PMID: 18310512 PMID: 18303992 PMID: 18295521 PMID: 18285614 PMID: 18284813 PMID: 18282556 PMID: 18280595 PMID: 18278466 PMID: 18277607 PMID: 18276983 PMID: 18276980 PMID: 18270244 PMID: 18260957 PMID: 18259008 PMID: 18256365 PMID: 18254187 PMID: 18248310 PMID: 18227407 PMID: 18219097 PMID: 18215696 PMID: 18215487 PMID: 18202670 PMID: 18199587 PMID: 18195162 PMID: 18187376 PMID: 18180398 PMID: 18158339 PMID: 18158336 PMID: 18156442 PMID: 18156192 PMID: 18094217 PMID: 18094033 PMID: 18093985 PMID: 18086957 PMID: 18083771 PMID: 18079463 PMID: 18076476 PMID: 18076345 PMID: 18067589 PMID: 18059573 PMID: 18056786 PMID: 18055759 PMID: 17975478 PMID: 17975473 PMID: 18049313 PMID: 18047631 PMID: 18047621 PMID: 18032469 PMID: 18030055 PMID: 18028778 PMID: 17999638 PMID: 17998477 PMID: 17994357 PMID: 17993965 PMID: 17992638 PMID: 17984671 PMID: 17982012 PMID: 17975203 PMID: 17971762 PMID: 17971759 PMID: 17962608 PMID: 17960144 PMID: 17954158 PMID: 17947499 PMID: 17928647 PMID: 17909117 PMID: 17904526 PMID: 17898997 PMID: 17898498 PMID: 17867925 PMID: 17852735 PMID: 17803988 PMID: 17785633 PMID: 17766473 PMID: 17764757 PMID: 17710229 PMID: 17701920 PMID: 17699685 PMID: 17693763 PMID: 17693108 PMID: 17691954 PMID: 17679649 PMID: 17675416 PMID: 17669489 PMID: 17666915 PMID: 17653966 PMID: 17652367 PMID: 17644566 PMID: 17644349 PMID: 17641676 PMID: 17635857 PMID: 17635855 PMID: 17622960 PMID: 17613534 PMID: 17612651 PMID: 17609490 PMID: 17604232 PMID: 17597703 PMID: 17597662 PMID: 17592513 PMID: 17591864 PMID: 17586406 PMID: 17567934 PMID: 17565644 PMID: 17557920 PMID: 17556673 PMID: 17549724 PMID: 17548721 PMID: 17548717 PMID: 17539008 PMID: 17533419 PMID: 17522574 PMID: 17522117 PMID: 17510605 PMID: 17507118 PMID: 17501693 PMID: 17490960 PMID: 17489367 PMID: 17485012 PMID: 17481853 PMID: 17477024 PMID: 17473728 PMID: 17470727 PMID: 17470694 PMID: 17466497 PMID: 17462541 PMID: 17450683 PMID: 17444274 PMID: 17442729 PMID: 17438361 PMID: 17413112 PMID: 17410406 PMID: 17406062 PMID: 17404161 PMID: 17377208 PMID: 17376760 PMID: 17376184 PMID: 17373635 PMID: 17361113 PMID: 17351370 PMID: 17347480 PMID: 17346127 PMID: 17341693 PMID: 17341601 PMID: 17320855 PMID: 17308041 PMID: 17289783 PMID: 17283870 PMID: 17264019 PMID: 17258737 PMID: 17257398 PMID: 17254747 PMID: 17245361 PMID: 17244945 PMID: 17244722 PMID: 17237412 PMID: 17220472 PMID: 17212359 PMID: 17211729 PMID: 17211250 PMID: 17210834 PMID: 17207282 PMID: 17202660 PMID: 17200775 PMID: 17200690 PMID: 17200437 PMID: 17199843 PMID: 17199257 PMID: 17198914 PMID: 17191022 PMID: 17170520 PMID: 17168664 PMID: 17164144 PMID: 17161492 PMID: 17150330 PMID: 17150260 PMID: 17143195 PMID: 17142134 PMID: 17126841 PMID: 17112788 PMID: 17112342 PMID: 17085527 PMID: 17085343 PMID: 17079123 PMID: 17076623 PMID: 17070432 PMID: 17070431 PMID: 17062967 PMID: 17060503 PMID: 17059813 PMID: 17034315 PMID: 17034310 PMID: 17023265 PMID: 17022122 PMID: 17012356 PMID: 17008638 PMID: 16999229 PMID: 16988189 PMID: 16981533 PMID: 16979916 PMID: 16978602 PMID: 16971374 PMID: 16970786 PMID: 16964694 PMID: 16956336 PMID: 16949718 PMID: 16936121 PMID: 16934677 PMID: 16932737 PMID: 16922818 PMID: 16920809 PMID: 16915037 PMID: 16895538 PMID: 16895178 PMID: 16892270 PMID: 16879569 PMID: 16878433 PMID: 16865295 PMID: 16847149 PMID: 16831539 PMID: 16824612 PMID: 16801485 PMID: 16798820 PMID: 16788145 PMID: 16782881 PMID: 16781129 PMID: 16764555 PMID: 16723295 PMID: 16723294 PMID: 16716091 PMID: 16713366 PMID: 16713008 PMID: 16698586 PMID: 16691295 PMID: 16688824 PMID: 16687663 PMID: 16650752 PMID: 16647809 PMID: 16647629 PMID: 16636194 PMID: 16621593 PMID: 16620286 PMID: 16615363 PMID: 16614311 PMID: 16580574 PMID: 16565906 PMID: 16563285 PMID: 16546838 PMID: 16543501 PMID: 16533160 PMID: 16523888 PMID: 16508588 PMID: 16507859 PMID: 16502517 PMID: 16500520 PMID: 16497090 PMID: 16490839 PMID: 16487260 PMID: 16482624 PMID: 16458868 PMID: 16444875 PMID: 16441951 PMID: 16440602 PMID: 16432053 PMID: 16428339 PMID: 16423283 PMID: 16421287 PMID: 16417945 PMID: 16401762 PMID: 16396943 PMID: 16388195 PMID: 16380650 PMID: 16371062 PMID: 16365187 PMID: 16342341 PMID: 16339392 PMID: 16331106 PMID: 16331105 PMID: 16331104 PMID: 16331096 PMID: 16330315 PMID: 16326639 PMID: 16322563 PMID: 16320694 PMID: 16316349 PMID: 16315601 PMID: 16298908 PMID: 16280288 PMID: 16242948 PMID: 16239641 PMID: 16230521 PMID: 16230518 PMID: 16227347 PMID: 16212878 PMID: 16207400 PMID: 16194124 PMID: 20527402 PMID: 16187692 PMID: 16176445 PMID: 16172159 PMID: 16166567 PMID: 16165346 PMID: 16162167 PMID: 16158068 PMID: 16155101 PMID: 16154102 PMID: 16144988 PMID: 16141308 PMID: 16131150 PMID: 16129977 PMID: 16121317 PMID: 16120191 PMID: 16115034 PMID: 16113940 PMID: 16106039 PMID: 16103275 PMID: 16087784 PMID: 16082155 PMID: 16061731 PMID: 16055511 PMID: 16053986 PMID: 16049546 PMID: 16049544 PMID: 16043660 PMID: 16036356 PMID: 16027259 PMID: 16006484 PMID: 16006432 PMID: 16004949 PMID: 15996671 PMID: 15994858 PMID: 15963975 PMID: 15958720 PMID: 15958269 PMID: 15958004 PMID: 15956822 PMID: 15956109 PMID: 15956108 PMID: 15954907 PMID: 15941780 PMID: 15936618 PMID: 15925276 PMID: 15924778 PMID: 15924000 PMID: 15913657 PMID: 15905461 PMID: 15900020 PMID: 15897359 PMID: 15894164 PMID: 15890973 PMID: 15883498 PMID: 15883230 PMID: 15879329 PMID: 15874906 PMID: 15870159 PMID: 15845610 PMID: 15838363 PMID: 15822183 PMID: 15821037 PMID: 15821014 PMID: 15817699 PMID: 15803435 PMID: 15795515 PMID: 15792364 PMID: 15782913 PMID: 15780761 PMID: 15777779 PMID: 15775785 PMID: 15775781 PMID: 15774269 PMID: 15773226 PMID: 15769451 PMID: 15764265 PMID: 15762422 PMID: 15725144 PMID: 15723968 PMID: 15721894 PMID: 15716703 PMID: 15715923 PMID: 15700631 PMID: 15699464 PMID: 15699460 PMID: 15699458 PMID: 15699457 PMID: 15699443 PMID: 15699263 PMID: 15691620 PMID: 15684809 PMID: 15684702 PMID: 15684012 PMID: 15683714 PMID: 15683712 PMID: 15662232 PMID: 15659536 PMID: 15658280 PMID: 15650608 PMID: 15646031 PMID: 15640327 PMID: 15639490 PMID: 15639216 PMID: 15630035 PMID: 15626689 PMID: 15618071 PMID: 15613618 PMID: 15613254 PMID: 15611369 PMID: 15610241 PMID: 15604302 PMID: 15596580 PMID: 15589689 PMID: 15583721 PMID: 15576842 PMID: 15575233 PMID: 15570896 PMID: 15569856 PMID: 15569854 PMID: 15569407 PMID: 15556564 PMID: 15548142 PMID: 15539883 PMID: 15528040 PMID: 15526988 PMID: 15516370 PMID: 15514281 PMID: 15496799 PMID: 15496307 PMID: 15492475 PMID: 15492320 PMID: 15486030 PMID: 15472607 PMID: 15471399 PMID: 15466650 PMID: 15460166 PMID: 15458541 PMID: 15452025 PMID: 15451779 PMID: 15383399 PMID: 15381052 PMID: 15380919 PMID: 15377497 PMID: 15372104 PMID: 15372097 PMID: 15369681 PMID: 15367173 PMID: 15365189 PMID: 15363814 PMID: 15361865 PMID: 15358613 PMID: 15350319 PMID: 15345487 PMID: 15320828 PMID: 15320697 PMID: 15320511 PMID: 15314574 PMID: 15302794 PMID: 15297254 PMID: 15285118 PMID: 15283037 PMID: 15273422 PMID: 15249543 PMID: 15243302 PMID: 15234074 PMID: 15231519 PMID: 15226275 PMID: 15224029 PMID: 15218395 PMID: 15213069 PMID: 15212473 PMID: 15211441 PMID: 15191411 PMID: 15182267 PMID: 15180494 PMID: 15177944 PMID: 15177916 PMID: 15171375 PMID: 15166180 PMID: 15163595 PMID: 15139786 PMID: 15138244 PMID: 15134517 PMID: 15134516 PMID: 15120475 PMID: 15117817 PMID: 15115150 PMID: 15114276 PMID: 15107293 PMID: 15100364 PMID: 15090706 PMID: 15082033 PMID: 15081318 PMID: 15081313 PMID: 15072717 PMID: 15051643 PMID: 15037564 PMID: 15031263 PMID: 15030794 PMID: 15020294 PMID: 15007032 PMID: 15006938 PMID: 15001201 PMID: 14985177 PMID: 14977874 PMID: 14967842 PMID: 14967839 PMID: 14967602 PMID: 14961045 PMID: 14757776 PMID: 14757688 PMID: 14752236 PMID: 14751848 PMID: 14751660 PMID: 14749737 PMID: 14744634 PMID: 14742258 PMID: 14718367 PMID: 14718355 PMID: 14717925 PMID: 14716214 PMID: 14714586 PMID: 14714582 PMID: 14701708 PMID: 14700509 PMID: 14691199 PMID: 14691197 PMID: 14683695 PMID: 14678698 PMID: 14676683 PMID: 14670842 PMID: 14670812 PMID: 14668575 PMID: 14668567 PMID: 14668269 PMID: 14666016 PMID: 14662651 PMID: 14656953 PMID: 14656952 PMID: 14656807 PMID: 14654757 PMID: 14654753 PMID: 14652649 PMID: 14649387 PMID: 14637180 PMID: 14634861 PMID: 14629650 PMID: 14626496 PMID: 14626495 PMID: 14626493 PMID: 14624957 PMID: 14620923 PMID: 14615281 PMID: 14613921 PMID: 14597594 PMID: 14583341 PMID: 14583335 PMID: 14582024 PMID: 14580369 PMID: 14580367 PMID: 14578115 PMID: 14570673 PMID: 14566078 PMID: 14565964 PMID: 14521947 PMID: 14521506 PMID: 14519522 PMID: 14519419 PMID: 14517679 PMID: 14508237 PMID: 14506893 PMID: 14504257 PMID: 12975388 PMID: 12970114 PMID: 12967948 PMID: 12953017 PMID: 12953014 PMID: 12946946 PMID: 12939515 PMID: 12939236 PMID: 12939232 PMID: 12921385 PMID: 12919953 PMID: 12910720 PMID: 12901446 PMID: 12900435 PMID: 12894486 PMID: 12885795 PMID: 12878447 PMID: 12874096 PMID: 12874095 PMID: 12874094 PMID: 12874093 PMID: 12874087 PMID: 12873816 PMID: 12872047 PMID: 12872045 PMID: 12872044 PMID: 12821598 PMID: 12805077 PMID: 12788078 PMID: 12782642 PMID: 12781059 PMID: 12781058 PMID: 12773029 PMID: 12764908 PMID: 12755960 PMID: 12753306 PMID: 12745201 PMID: 12745192 PMID: 12738901 PMID: 12738505 PMID: 12716139 PMID: 12716137 PMID: 12714872 PMID: 12709568 PMID: 12702924 PMID: 12699909 PMID: 12696636 PMID: 12694812 PMID: 12681244 PMID: 12679169 PMID: 12669109 PMID: 12668590 PMID: 12664605 PMID: 12661920 PMID: 12658208 PMID: 12656651 PMID: 12654613 PMID: 12651642 PMID: 12647282 PMID: 12640255 PMID: 12631079 PMID: 12626356 PMID: 12623999 PMID: 12623992 PMID: 12623985 PMID: 12623982 PMID: 12623967 PMID: 12623938 PMID: 12615693 PMID: 12609817 PMID: 12605017 PMID: 12600888 PMID: 12598425 PMID: 12591008 PMID: 12579126 PMID: 12574096 PMID: 12570747 PMID: 12569272 PMID: 12566962 PMID: 12537256 PMID: 12531784 PMID: 12530633 PMID: 12525997 PMID: 12525995 PMID: 12510364 PMID: 12500202 PMID: 12492449 PMID: 12468584 PMID: 12468571 PMID: 12459486 PMID: 12456495 PMID: 12451327 PMID: 12451006 PMID: 12444204 PMID: 12444028 PMID: 12439866 PMID: 12434548 PMID: 12433654 PMID: 12431905 PMID: 12427646 PMID: 12412689 PMID: 12411476 PMID: 12411468 PMID: 12411466 PMID: 12410847 PMID: 12410168 PMID: 12401432 PMID: 12391520 PMID: 12391278 PMID: 12391273 PMID: 12388660 PMID: 12388340 PMID: 12354470 PMID: 12324236 PMID: 12243041 PMID: 12241530 PMID: 12235032 PMID: 12225948 PMID: 12219874 PMID: 12218315 PMID: 12218313 PMID: 12215466 PMID: 12208146 PMID: 12190041 PMID: 12185963 PMID: 12185008 PMID: 12184052 PMID: 12182967 PMID: 12181171 PMID: 12176110 PMID: 12175894 PMID: 12163344 PMID: 12160196 PMID: 12149501 PMID: 12144877 PMID: 12144869 PMID: 12135321 PMID: 12130981 PMID: 12120954 PMID: 12119234 PMID: 12105142 PMID: 12093889 PMID: 12088278 PMID: 12082102 PMID: 12072574 PMID: 12069931 PMID: 12065853 PMID: 12062720 PMID: 12060588 PMID: 12060522 PMID: 12049831 PMID: 12042029 PMID: 12019279 PMID: 12011667 PMID: 11993718 PMID: 11983295 PMID: 11979514 PMID: 11964369 PMID: 11960511 PMID: 11913222 PMID: 11910303 PMID: 11907186 PMID: 11903507 PMID: 11897770 PMID: 11897764 PMID: 11892994 PMID: 11891614 PMID: 11890898 PMID: 11887169 PMID: 11884287 PMID: 11882628 PMID: 11882623 PMID: 11882617 PMID: 11882612 PMID: 11882605 PMID: 11882577 PMID: 11880295 PMID: 11873835 PMID: 11859420 PMID: 11853230 PMID: 11837981 PMID: 11832453 PMID: 11814618 PMID: 11811373 PMID: 11808073 PMID: 11805541 PMID: 11804870 PMID: 11804866 PMID: 11799094 PMID: 11785065 PMID: 11773611 PMID: 11772398 PMID: 11765147 PMID: 11758971 PMID: 11751730 PMID: 11751709 PMID: 11744644 PMID: 11739239 PMID: 11735360 PMID: 11733614 PMID: 11728983 PMID: 11726622 PMID: 11724215 PMID: 11713147 PMID: 11712212 PMID: 11704540 PMID: 11704531 PMID: 11679404 PMID: 11675948 PMID: 11668076 PMID: 11641301 PMID: 11641299 PMID: 11641295 PMID: 11641290 PMID: 11606318 PMID: 11601330 PMID: 11597987 PMID: 11593105 PMID: 11593093 PMID: 11587993 PMID: 11584574 PMID: 11581303 PMID: 11570987 PMID: 11568780 PMID: 11566953 PMID: 11566950 PMID: 11553512 PMID: 11551140 PMID: 11545625 PMID: 11533280 PMID: 11516480 PMID: 11510754 PMID: 11509469 PMID: 11509444 PMID: 11509332 PMID: 11505055 PMID: 11498517 PMID: 11484065 PMID: 11483635 PMID: 11477200 PMID: 11471069 PMID: 11465660 PMID: 11465346 PMID: 11463755 PMID: 11453630 PMID: 11451037 PMID: 11445706 PMID: 11435744 PMID: 11434937 PMID: 11434650 PMID: 11433214 PMID: 11427614 PMID: 11427039 PMID: 11411771 PMID: 11408262 PMID: 11380805 PMID: 11393683 PMID: 11391985 PMID: 11390023 PMID: 11385296 PMID: 11384581 PMID: 11379794 PMID: 11379791 PMID: 11378531 PMID: 11375256 PMID: 11369175 PMID: 11367676 PMID: 11360634 PMID: 11360609 PMID: 11356791 PMID: 11355072 PMID: 11339690 PMID: 11334879 PMID: 11334556 PMID: 11320253 PMID: 11305695 PMID: 11304662 PMID: 11304522 PMID: 11304521 PMID: 11300653 PMID: 11294599 PMID: 11292182 PMID: 11285322 PMID: 11285308 PMID: 11280643 PMID: 11278979 PMID: 11274747 PMID: 11273841 PMID: 11263364 PMID: 11262599 PMID: 11249862 PMID: 11246814 PMID: 11246317 PMID: 11244311 PMID: 11244013 PMID: 11243964 PMID: 11243414 PMID: 11242460 PMID: 11239021 PMID: 11230365 PMID: 11230346 PMID: 11230334 PMID: 11230332 PMID: 11230326 PMID: 11230325 PMID: 11230310 PMID: 11230294 PMID: 11230290 PMID: 11224699 PMID: 11195057 PMID: 11192940 PMID: 11192936 PMID: 11192933 PMID: 11208936 PMID: 11208362 PMID: 11171663 PMID: 11171624 PMID: 11168444 PMID: 11166679 PMID: 11158948 PMID: 11156591 PMID: 11149279 PMID: 11136866 PMID: 11110881 PMID: 11133498 PMID: 11133172 PMID: 11133171 PMID: 11132611 PMID: 11132177 PMID: 11129225 PMID: 11126238 PMID: 11121807 PMID: 11104729 PMID: 11095155 PMID: 11090543 PMID: 11082156 PMID: 11082155 PMID: 11080183 PMID: 11078436 PMID: 11078180 PMID: 11076816 PMID: 11071299 PMID: 11065217 PMID: 11061346 PMID: 11049696 PMID: 11045975 PMID: 11040244 PMID: 11040234 PMID: 11034952 PMID: 11031209 PMID: 11029367 PMID: 11026622 PMID: 11026280 PMID: 11023940 PMID: 11022891 PMID: 11001943 PMID: 11001172 PMID: 10988259 PMID: 10965691 PMID: 10965193 PMID: 10954003 PMID: 10954001 PMID: 10948089 PMID: 10948078 PMID: 10944426 PMID: 10936484 PMID: 10930383 PMID: 10929431 PMID: 10919850 PMID: 10924071 PMID: 10924050 PMID: 10920715 PMID: 10920332 PMID: 10920204 PMID: 10913376 PMID: 10904019 PMID: 10901388 PMID: 10894784 PMID: 10892668 PMID: 10892661 PMID: 10891111 PMID: 10874469 PMID: 10873513 PMID: 10867862 PMID: 10859998 PMID: 10856282 PMID: 10856272 PMID: 10856269 PMID: 10854087 PMID: 10854076 PMID: 10854075 PMID: 10846615 PMID: 10842658 PMID: 10839922 PMID: 10836725 PMID: 10833791 PMID: 10821343 PMID: 10818069 PMID: 10803730 PMID: 10803488 PMID: 10781427 PMID: 10779386 PMID: 10775563 PMID: 10775129 PMID: 10776058 PMID: 10770325 PMID: 10744358 PMID: 10733442 PMID: 10726786 PMID: 10720882 PMID: 10720587 PMID: 10720586 PMID: 10712778 PMID: 10711867 PMID: 10710121 PMID: 10700516 PMID: 10696069 PMID: 10681399 PMID: 10679511 PMID: 10670690 PMID: 10655114 PMID: 10654518 PMID: 10642344 PMID: 10642331 PMID: 10642327 PMID: 10642320 PMID: 10642312 PMID: 10642306 PMID: 10642277 PMID: 10641705 PMID: 11120706 PMID: 11116101 PMID: 10620200 PMID: 10619586 PMID: 10617987 PMID: 10615427 PMID: 10614144 PMID: 10605951 PMID: 10604544 PMID: 10601699 PMID: 10601125 PMID: 10588688 PMID: 10586525 PMID: 10581155 PMID: 10579296 PMID: 10579269 PMID: 10553820 PMID: 10559135 PMID: 10551974 PMID: 10549860 PMID: 10539741 PMID: 10535387 PMID: 10526902 PMID: 10523395 PMID: 10523389 PMID: 10523372 PMID: 10520810 PMID: 10515440 PMID: 10512267 PMID: 10509552 PMID: 10502757 PMID: 10493105 PMID: 10489401 PMID: 10489109 PMID: 10484602 PMID: 10484046 PMID: 10479232 PMID: 10478273 PMID: 10476619 PMID: 10471607 PMID: 10466476 PMID: 10440208 PMID: 10436308 PMID: 10430735 PMID: 10428307 PMID: 10413062 PMID: 10411547 PMID: 10406821 PMID: 10403604 PMID: 10402073 PMID: 10385598 PMID: 10382236 PMID: 10381895 PMID: 10375769 PMID: 10373232 PMID: 10369379 PMID: 10364089 PMID: 10329875 PMID: 10323263 PMID: 10321793 PMID: 10320629 PMID: 10234942 PMID: 10233136 PMID: 10232500 PMID: 10226854 PMID: 10226768 PMID: 10218733 PMID: 10218731 PMID: 10213252 PMID: 10211585 PMID: 10211584 PMID: 10211583 PMID: 10193773 PMID: 10100097 PMID: 10093893 PMID: 10092995 PMID: 10092983 PMID: 10090346 PMID: 10090338 PMID: 10087011 PMID: 10082496 PMID: 10079175 PMID: 10079015 PMID: 10072716 PMID: 10070137 PMID: 10067789 PMID: 10030848 PMID: 10023867 PMID: 9950873 PMID: 9931155 PMID: 9931140 PMID: 9931139 PMID: 9931138 PMID: 9929405 PMID: 9927152 PMID: 9920344 PMID: 9916257 PMID: 9876989 PMID: 9876450 PMID: 9865590 PMID: 9863664 PMID: 9863193 PMID: 9861778 PMID: 9859722 PMID: 9856979 PMID: 9856964 PMID: 9855596 PMID: 9831829 PMID: 9825473 PMID: 9822642 PMID: 9821020 PMID: 9817705 PMID: 9797169 PMID: 9780725 PMID: 9772127 PMID: 9742857 PMID: 9741570 PMID: 9740611 PMID: 9731560 PMID: 9727610 PMID: 9711442 PMID: 9706665 PMID: 9700980 PMID: 9688753 PMID: 9688677 PMID: 9683041 PMID: 9674642 PMID: 9674634 PMID: 9673818 PMID: 9673428 PMID: 9661585 PMID: 9657352 PMID: 9648942 PMID: 9644719 PMID: 9636164 PMID: 9565256 PMID: 9623788 PMID: 9622285 PMID: 9622144 PMID: 9614643 PMID: 9606863 PMID: 9605813 PMID: 9542566 PMID: 9589380 PMID: 9590322 PMID: 9587840 PMID: 9585158 PMID: 9583194 PMID: 9581546 PMID: 9572571 PMID: 9566780 PMID: 9550591 PMID: 9544875 PMID: 9543640 PMID: 9535424 PMID: 9535414 PMID: 9530191 PMID: 9527508 PMID: 9524055 PMID: 9503427 PMID: 9498538 PMID: 9495274 PMID: 9495266 PMID: 9488222 PMID: 9471230 PMID: 9461234 PMID: 9461224 PMID: 9458854 PMID: 9431855 PMID: 9453310 PMID: 9453309 PMID: 9453306 PMID: 9453305 PMID: 9453304 PMID: 9453302 PMID: 9451469 PMID: 9440240 PMID: 9384242 PMID: 9435687 PMID: 9425912 PMID: 9423755 PMID: 9408223 PMID: 9408065 PMID: 9411311 PMID: 9410959 PMID: 9409562 PMID: 9403589 PMID: 9369254 PMID: 9362262 PMID: 9360180 PMID: 9350584 PMID: 9329766 PMID: 9306052 PMID: 9336397 PMID: 9336387 PMID: 9322988 PMID: 9307481 PMID: 9378247 PMID: 9281597 PMID: 9284401 PMID: 9280209 PMID: 9279254 PMID: 9277505 PMID: 9250696 PMID: 9217714 PMID: 9252029 PMID: 9268228 PMID: 9252547 PMID: 9236419 PMID: 9234829 PMID: 9233750 PMID: 9289376 PMID: 9230925 PMID: 9220270 PMID: 9195523 PMID: 9180635 PMID: 9174486 PMID: 9176341 PMID: 9176315 PMID: 9163651 PMID: 9160783 PMID: 9157942 PMID: 9113986 PMID: 10495783 PMID: 9210249 PMID: 9196521 PMID: 9167210 PMID: 9128202 PMID: 9125643 PMID: 9111529 PMID: 9107182 PMID: 9097077 PMID: 9095099 PMID: 10684065 PMID: 9073608 PMID: 9056695 PMID: 9056685 PMID: 9052898 PMID: 9052892 PMID: 9209710 PMID: 9124442 PMID: 9124424 PMID: 9051725 PMID: 9037329 PMID: 9750589 PMID: 9530433 PMID: 9479629 PMID: 9460845 PMID: 9416330 PMID: 9412077 PMID: 9257047 PMID: 9126880 PMID: 9089893 PMID: 9064474 PMID: 9039122 PMID: 9039121 PMID: 9039119 PMID: 9039112 PMID: 9039081 PMID: 9038972 PMID: 9028640 PMID: 8989192 PMID: 9014219 PMID: 8997290 PMID: 8986457 PMID: 8937452 PMID: 8981627 PMID: 8950034 PMID: 8946652 PMID: 8940291 PMID: 8937726 PMID: 8932993 PMID: 8930174 PMID: 8922563 PMID: 8917035 PMID: 8901820 PMID: 8893830 PMID: 8951997 PMID: 8936586 PMID: 8887285 PMID: 8843895 PMID: 8905421 PMID: 8872845 PMID: 8869407 PMID: 8794831 PMID: 8794819 PMID: 8781542 PMID: 8949372 PMID: 8770097 PMID: 8756014 PMID: 8707383 PMID: 8707377 PMID: 9201331 PMID: 8946069 PMID: 8829117 PMID: 8818928 PMID: 8675267 PMID: 8674891 PMID: 8670323 PMID: 8875764 PMID: 8829198 PMID: 8803631 PMID: 8800575 PMID: 8799531 PMID: 8743533 PMID: 8641744 PMID: 8641741 PMID: 8637442 PMID: 8963891 PMID: 8790929 PMID: 8767630 PMID: 8665778 PMID: 8633958 PMID: 8621209 PMID: 8613060 PMID: 8621760 PMID: 8860957 PMID: 8847877 PMID: 8738735 PMID: 8738072 PMID: 8732601 PMID: 8721386 PMID: 8964757 PMID: 8860111 PMID: 8860100 PMID: 8698441 PMID: 8613244 PMID: 8613215 PMID: 8925186 PMID: 8868072 PMID: 8848434 PMID: 8821826 PMID: 8808162 PMID: 8779837 PMID: 8728295 PMID: 8720417 PMID: 8711134 PMID: 8699134 PMID: 8623815 PMID: 8567056 PMID: 9157710 PMID: 8959802 PMID: 8933500 PMID: 8895963 PMID: 8871682 PMID: 8856265 PMID: 8851819 PMID: 8822232 PMID: 8777474 PMID: 8656653 PMID: 8642189 PMID: 8591888 PMID: 8591886 PMID: 8550317 PMID: 7486485 PMID: 9062551 PMID: 8998253 PMID: 8998251 PMID: 8966195 PMID: 8903651 PMID: 8821118 PMID: 8746951 PMID: 8664452 PMID: 7498987 PMID: 7498963 PMID: 7484888 PMID: 7484885 PMID: 7495221 PMID: 8596776 PMID: 8595527 PMID: 8581285 PMID: 8534627 PMID: 7591017 PMID: 7591016 PMID: 8845070 PMID: 8777575 PMID: 8777568 PMID: 8742618 PMID: 8582070 PMID: 8577778 PMID: 8573753 PMID: 7574058 PMID: 7558231 PMID: 7664438 PMID: 8570572 PMID: 8551489 PMID: 8541011 PMID: 8540946 PMID: 7668305 PMID: 7665980 PMID: 7568902 PMID: 7474516 PMID: 7544116 PMID: 8572878 PMID: 8570923 PMID: 7635531 PMID: 7585817 PMID: 7569536 PMID: 8534259 PMID: 8532605 PMID: 8524183 PMID: 7675824 PMID: 7675821 PMID: 7631831 PMID: 7594441 PMID: 7594435 PMID: 7582857 PMID: 7541937 PMID: 8529075 PMID: 7768568 PMID: 7675628 PMID: 7644560 PMID: 7611475 PMID: 7564329 PMID: 7546632 PMID: 7755079 PMID: 7737707 PMID: 7561014 PMID: 7776519 PMID: 7733312 PMID: 7721444 PMID: 7721392 PMID: 7698587 PMID: 7606651 PMID: 7495094 PMID: 9101251 PMID: 7881662 PMID: 7862371 PMID: 7777724 PMID: 7775859 PMID: 7774663 PMID: 7840069 PMID: 7752646 PMID: 7723248 PMID: 7539934 PMID: 9524655 PMID: 9072433 PMID: 9072404 PMID: 8641319 PMID: 8590251 PMID: 8582461 PMID: 8559476 PMID: 7879457 PMID: 7840337 PMID: 7759856 PMID: 7756098 PMID: 7725884 PMID: 7653078 PMID: 7626978 PMID: 7877612 PMID: 7989598 PMID: 7982662 PMID: 7982651 PMID: 7886025 PMID: 7702804 PMID: 7699653 PMID: 7699222 PMID: 7695874 PMID: 7532874 PMID: 7935657 PMID: 7977770 PMID: 7882905 PMID: 7868418 PMID: 7864999 PMID: 7848621 PMID: 7848331 PMID: 7620514 PMID: 7533848 PMID: 8088922 PMID: 7709199 PMID: 7857372 PMID: 8092306 PMID: 7836137 PMID: 7808838 PMID: 7528303 PMID: 7520443 PMID: 8067471 PMID: 8002544 PMID: 7825084 PMID: 7814848 PMID: 8020605 PMID: 8002047 PMID: 7994448 PMID: 7987719 PMID: 7970077 PMID: 7939368 PMID: 7735986 PMID: 8206638 PMID: 8206596 PMID: 8062510 PMID: 7930391 PMID: 7523781 PMID: 8175982 PMID: 8079443 PMID: 8066098 PMID: 8066094 PMID: 7945561 PMID: 7915035 PMID: 8184891 PMID: 8143049 PMID: 8022853 PMID: 8021907 PMID: 7735964 PMID: 7511568 PMID: 8199718 PMID: 8199712 PMID: 8193093 PMID: 8181217 PMID: 8166228 PMID: 8160707 PMID: 8128353 PMID: 8118653 PMID: 8066384 PMID: 8022064 PMID: 8011980 PMID: 8005841 PMID: 7912508 PMID: 7911801 PMID: 7513130 PMID: 8304827 PMID: 8293559 PMID: 8165724 PMID: 8157859 PMID: 8142566 PMID: 8141169 PMID: 8113009 PMID: 8021464 PMID: 8146236 PMID: 8280782 PMID: 8306543 PMID: 8282345 PMID: 8274625 PMID: 8152333 PMID: 8127025 PMID: 8048287 PMID: 7871774 PMID: 7856235 PMID: 7856218 PMID: 7817530 PMID: 7719289 PMID: 8121906 PMID: 8267432 PMID: 8244267 PMID: 8136091 PMID: 8133019 PMID: 8130119 PMID: 7510280 PMID: 8305640 PMID: 8293777 PMID: 8255635 PMID: 8239773 PMID: 8239151 PMID: 8222495 PMID: 8222105 PMID: 7509294 PMID: 8406662 PMID: 8261661 PMID: 8216015 PMID: 8042306 PMID: 7505593 PMID: 8403245 PMID: 8401415 PMID: 8378666 PMID: 8359804 PMID: 8353893 PMID: 8349328 PMID: 8231040 PMID: 8222534 PMID: 8217999 PMID: 7511313 PMID: 8396345 PMID: 8340155 PMID: 8262302 PMID: 8226502 PMID: 8219660 PMID: 8210758 PMID: 8356015 PMID: 8326295 PMID: 8211492 PMID: 8149697 PMID: 8505111 PMID: 8505098 PMID: 8505087 PMID: 8504749 PMID: 8396104 PMID: 8343228 PMID: 8343227 PMID: 8341725 PMID: 8180727 PMID: 7763887 PMID: 7685934 PMID: 7685006 PMID: 8507196 PMID: 8390526 PMID: 8335575 PMID: 8316758 PMID: 8311753 PMID: 8103335 PMID: 8479813 PMID: 8475962 PMID: 8473549 PMID: 8489125 PMID: 8094827 PMID: 8482311 PMID: 8478037 PMID: 8469678 PMID: 8460710 PMID: 8437781 PMID: 8395243 PMID: 8385532 PMID: 8358008 PMID: 8357995 PMID: 8042297 PMID: 8357359 PMID: 8095429 PMID: 8498970 PMID: 8446650 PMID: 8441226 PMID: 8384614 PMID: 8383447 PMID: 8382502 PMID: 8324835 PMID: 7681429 PMID: 8444700 PMID: 8444695 PMID: 8424451 PMID: 8424122 PMID: 8416417 PMID: 8402751 PMID: 8378844 PMID: 8229615 PMID: 8067253 PMID: 8062718 PMID: 7975402 PMID: 7905203 PMID: 1490969 PMID: 1293602 PMID: 1342789 PMID: 1475272 PMID: 1474095 PMID: 1474086 PMID: 1440713 PMID: 1428115 PMID: 1330923 PMID: 1307083 PMID: 1280952 PMID: 1476271 PMID: 1415742 PMID: 1415735 PMID: 1398887 PMID: 1335001 PMID: 1329556 PMID: 1329185 PMID: 1285867 PMID: 1280717 PMID: 1492398 PMID: 1457335 PMID: 1433869 PMID: 1421673 PMID: 1410527 PMID: 1396860 PMID: 1641773 PMID: 1639963 PMID: 1639455 PMID: 1526884 PMID: 1405324 PMID: 1328748 PMID: 1603118 PMID: 1488334 PMID: 1506397 PMID: 1383617 PMID: 1410518 PMID: 1534314 PMID: 1506726 PMID: 1393269 PMID: 1321638 PMID: 1320077 PMID: 1320071 PMID: 1622907 PMID: 1575347 PMID: 1567044 PMID: 1513001 PMID: 1504198 PMID: 1413641 PMID: 1324265 PMID: 1516634 PMID: 1635208 PMID: 1635183 PMID: 1592709 PMID: 1555866 PMID: 1555864 PMID: 1532833 PMID: 1521675 PMID: 1315648 PMID: 1639208 PMID: 1630030 PMID: 1575947 PMID: 1572577 PMID: 1286547 PMID: 1311763 PMID: 1542546 PMID: 1740628 PMID: 1735595 PMID: 1735587 PMID: 1563330 PMID: 1559913 PMID: 1545169 PMID: 1539713 PMID: 1539677 PMID: 1385908 PMID: 1328596 PMID: 1326677 PMID: 1313634 PMID: 1350991 PMID: 1727763 PMID: 1632315 PMID: 1619691 PMID: 1594324 PMID: 1557557 PMID: 1537723 PMID: 1509159 PMID: 1484261 PMID: 1462863 PMID: 1462859 PMID: 1458213 PMID: 1449835 PMID: 1413811 PMID: 1412450 PMID: 1412449 PMID: 1363407 PMID: 1349658 PMID: 1347260 PMID: 1307345 PMID: 1284561 PMID: 1282978 PMID: 1282966 PMID: 1282633 PMID: 1741521 PMID: 1837059 PMID: 1818979 PMID: 1818775 PMID: 1794206 PMID: 1777588 PMID: 1777456 PMID: 1743757 PMID: 1726178 PMID: 1683609 PMID: 1683608 PMID: 1668010 PMID: 1761141 PMID: 1934867 PMID: 1937679 PMID: 1839614 PMID: 1811280 PMID: 1807007 PMID: 1805199 PMID: 1775625 PMID: 1659228 PMID: 1936584 PMID: 1839873 PMID: 1747214 PMID: 1657496 PMID: 1657495 PMID: 1655653 PMID: 1888055 PMID: 1930860 PMID: 1908631 PMID: 1882820 PMID: 1834087 PMID: 1797924 PMID: 1753537 PMID: 1716064 PMID: 1657042 PMID: 1655330 PMID: 1653767 PMID: 1938712 PMID: 1937159 PMID: 1936664 PMID: 1930848 PMID: 1908864 PMID: 1885223 PMID: 1864958 PMID: 1831598 PMID: 1802432 PMID: 1657862 PMID: 1655882 PMID: 1934222 PMID: 1910640 PMID: 1871182 PMID: 1860709 PMID: 1858913 PMID: 1771100 PMID: 2045158 PMID: 1914243 PMID: 1912396 PMID: 1909312 PMID: 1886913 PMID: 1828131 PMID: 1711945 PMID: 1903902 PMID: 2065475 PMID: 2032803 PMID: 2029277 PMID: 1908580 PMID: 1881943 PMID: 1852125 PMID: 1852118 PMID: 1851689 PMID: 1712311 PMID: 2065689 PMID: 2019140 PMID: 2013478 PMID: 2012627 PMID: 1883628 PMID: 1826193 PMID: 1711633 PMID: 2062016 PMID: 2034476 PMID: 2000943 PMID: 1999361 PMID: 1999360 PMID: 1999357 PMID: 1857725 PMID: 1802195 PMID: 2063232 PMID: 2030809 PMID: 1899531 PMID: 1899491 PMID: 1849530 PMID: 1826997 PMID: 1824165 PMID: 1673799 PMID: 1987435 PMID: 2065462 PMID: 2053347 PMID: 2051884 PMID: 1996900 PMID: 1948744 PMID: 1940192 PMID: 1848945 PMID: 1823218 PMID: 1794953 PMID: 1794726 PMID: 1772685 PMID: 1725198 PMID: 1725017 PMID: 1666433 PMID: 2174433 PMID: 2277204 PMID: 2244565 PMID: 2173970 PMID: 2147175 PMID: 2147173 PMID: 2124641 PMID: 2124426 PMID: 2077541 PMID: 1964752 PMID: 1962803 PMID: 2177810 PMID: 2283006 PMID: 2272976 PMID: 2261156 PMID: 2171807 PMID: 2150554 PMID: 2133429 PMID: 2097166 PMID: 2291866 PMID: 2283638 PMID: 2281141 PMID: 2274570 PMID: 2274569 PMID: 2274568 PMID: 2225702 PMID: 2222942 PMID: 2221102 PMID: 2209041 PMID: 2174941 PMID: 2171364 PMID: 2124586 PMID: 2085853 PMID: 1979787 PMID: 2242327 PMID: 2232487 PMID: 2203682 PMID: 2174565 PMID: 2124406 PMID: 2120373 PMID: 2256275 PMID: 2219874 PMID: 2215089 PMID: 2198572 PMID: 2145699 PMID: 2115026 PMID: 2093954 PMID: 1980714 PMID: 2398267 PMID: 2394657 PMID: 2273596 PMID: 2124461 PMID: 2124460 PMID: 2114425 PMID: 2204238 PMID: 2200897 PMID: 2165993 PMID: 2163217 PMID: 2161793 PMID: 2143586 PMID: 2141003 PMID: 1974438 PMID: 1693376 PMID: 2399267 PMID: 2345673 PMID: 2341910 PMID: 2332238 PMID: 2232114 PMID: 2186636 PMID: 2124686 PMID: 2112669 PMID: 2110113 PMID: 2102719 PMID: 1970789 PMID: 2347783 PMID: 2343443 PMID: 2318970 PMID: 2156677 PMID: 2112129 PMID: 2112004 PMID: 2111678 PMID: 2109944 PMID: 2109798 PMID: 2108083 PMID: 1971818 PMID: 1969511 PMID: 2 |
| Bladder cancer | PMID: 21223815 PMID: 20732908 PMID: 20568111 PMID: 20501621 PMID: 20361139 PMID: 18950807 PMID: 18711411 PMID: 18459948 PMID: 20717483 PMID: 18162775 PMID: 18057892 PMID: 17445434 PMID: 16675585 PMID: 16309229 PMID: 15270419 PMID: 15133541 PMID: 12879645 PMID: 12527845 PMID: 12352416 PMID: 11144893 PMID: 10570093 PMID: 10479227 PMID: 8677560 PMID: 8658457 PMID: 8578986 PMID: 7889049 PMID: 8447299 PMID: 3185200 PMID: 3566300 PMID: 3747162 PMID: 3942969 PMID: 3851017 |
| Caffeine metabolism | PMID: 19999796 PMID: 17616786 PMID: 2680964 |
| Calcium signaling pathway | PMID: 19393642 PMID: 10411692 PMID: 9369284 |
| Cardiac muscle contraction | PMID: 19965931 PMID: 15715922 PMID: 10422459 PMID: 2000992 |
| Cell adhesion molecules (CAMs) | PMID: 16095049 PMID: 12448198 PMID: 8311068 |
| Chemokine signaling pathway |  |
| Chronic myeloid leukemia | PMID: 21211419 PMID: 21210124 PMID: 18986702 PMID: 17128687 PMID: 14614643 PMID: 12893021 PMID: 12884818 PMID: 12688317 PMID: 12134204 PMID: 11555513 PMID: 10986148 PMID: 9746766 PMID: 7858532 PMID: 8260157 PMID: 8347786 PMID: 2620893 |
| Citrate cycle (TCA cycle) | PMID: 19095954 PMID: 17954369 PMID: 15550523 PMID: 15141213 PMID: 15141197 PMID: 11707454 PMID: 8448994 PMID: 1544913 PMID: 3366475 PMID: 3100863 PMID: 7121668 PMID: 920353 PMID: 934001 PMID: 1031989 PMID: 1017410 PMID: 4564017 PMID: 4306132 PMID: 4234665 PMID: 14334170 PMID: 14044245 |
| Colorectal cancer | PMID: 21248449 PMID: 21243394 PMID: 21163073 PMID: 21146479 PMID: 21131552 PMID: 21097651 PMID: 21074408 PMID: 20958992 PMID: 20881153 PMID: 20868601 PMID: 20857527 PMID: 20828744 PMID: 20812490 PMID: 20799147 PMID: 20798560 PMID: 20733336 PMID: 20702138 PMID: 20680303 PMID: 20670865 PMID: 20661569 PMID: 20658253 PMID: 20567110 PMID: 20504361 PMID: 20460980 PMID: 20424611 PMID: 20406923 PMID: 20395126 PMID: 20380333 PMID: 20302723 PMID: 20186298 PMID: 20173137 PMID: 20167233 PMID: 20136906 PMID: 20123124 PMID: 19967487 PMID: 19942597 PMID: 19940466 PMID: 19940012 PMID: 19921473 PMID: 19835652 PMID: 19822514 PMID: 19793713 PMID: 19785749 PMID: 19774471 PMID: 19755965 PMID: 19726453 PMID: 19707368 PMID: 19643598 PMID: 19636011 PMID: 19626652 PMID: 19598023 PMID: 19591135 PMID: 19588372 PMID: 19528879 PMID: 19526201 PMID: 19513949 PMID: 19509565 PMID: 19508512 PMID: 19443817 PMID: 19406901 PMID: 19377787 PMID: 19333227 PMID: 19321964 PMID: 19321569 PMID: 19308410 PMID: 19261963 PMID: 19256771 PMID: 19235032 PMID: 19228749 PMID: 19184059 PMID: 19184020 PMID: 19151582 PMID: 20616890 PMID: 18983014 PMID: 18854571 PMID: 18842611 PMID: 18786559 PMID: 18785568 PMID: 18632490 PMID: 18625170 PMID: 18567598 PMID: 18512008 PMID: 18501075 PMID: 18498064 PMID: 18471041 PMID: 18317846 PMID: 18226850 PMID: 18069765 PMID: 17990351 PMID: 17972534 PMID: 17951483 PMID: 17947725 PMID: 17921195 PMID: 17878894 PMID: 17846007 PMID: 17684126 PMID: 17681780 PMID: 17629064 PMID: 17573328 PMID: 17565263 PMID: 17477810 PMID: 17442997 PMID: 17442373 PMID: 17381413 PMID: 17376054 PMID: 17375777 PMID: 17374619 PMID: 17286784 PMID: 17282972 PMID: 17218488 PMID: 17212999 PMID: 17164361 PMID: 17145522 PMID: 17106282 PMID: 17086869 PMID: 17075324 PMID: 17066486 PMID: 16961747 PMID: 16926943 PMID: 16923261 PMID: 16877536 PMID: 16873427 PMID: 16866078 PMID: 16849749 PMID: 16842197 PMID: 16790036 PMID: 16774493 PMID: 16764097 PMID: 16750963 PMID: 16728921 PMID: 16721800 PMID: 16601426 PMID: 16582132 PMID: 16546242 PMID: 16489301 PMID: 16387667 PMID: 16365764 PMID: 16301833 PMID: 16301832 PMID: 16291519 PMID: 16127229 PMID: 16050889 PMID: 16018743 PMID: 15934875 PMID: 15908660 PMID: 15843419 PMID: 15779071 PMID: 15738537 PMID: 15683477 PMID: 15655642 PMID: 15612152 PMID: 15552623 PMID: 15479482 PMID: 15479481 PMID: 15340658 PMID: 19780248 PMID: 15187215 PMID: 15178811 PMID: 15175435 PMID: 14532868 PMID: 12802793 PMID: 12655441 PMID: 12642982 PMID: 12561598 PMID: 12506171 PMID: 12428190 PMID: 12370158 PMID: 12366618 PMID: 11778127 PMID: 11349515 PMID: 11256879 PMID: 11196455 PMID: 11082143 PMID: 10965781 PMID: 10916814 PMID: 10914917 PMID: 10826013 PMID: 10741301 PMID: 10705704 PMID: 10674000 PMID: 10659641 PMID: 10465343 PMID: 10234343 PMID: 9732227 PMID: 9484426 PMID: 9107220 PMID: 8619207 PMID: 8625192 PMID: 2031492 PMID: 1868509 PMID: 2199362 PMID: 1976740 PMID: 2276560 PMID: 3390835 PMID: 3610539 PMID: 3807542 PMID: 3711252 |
| Complement and coagulation cascades |  |
| Cytokine-cytokine receptor interaction |  |
| Dilated cardiomyopathy | PMID: 21210757 PMID: 21174696 PMID: 21173513 PMID: 21160914 PMID: 21090529 PMID: 21047408 PMID: 20979862 PMID: 20977421 PMID: 20949226 PMID: 20945963 PMID: 20921936 PMID: 20876060 PMID: 20866004 PMID: 20863775 PMID: 20860173 PMID: 20845617 PMID: 20828585 PMID: 20736239 PMID: 20712239 PMID: 20679547 PMID: 20653703 PMID: 20640098 PMID: 20621179 PMID: 20537415 PMID: 20531215 PMID: 20527612 PMID: 20501410 PMID: 20458831 PMID: 20434045 PMID: 20374947 PMID: 20348225 PMID: 20331808 PMID: 20224752 PMID: 20198391 PMID: 20193969 PMID: 20178563 PMID: 20108498 PMID: 20093812 PMID: 20017903 PMID: 20008274 PMID: 19946241 PMID: 19938884 PMID: 19937007 PMID: 19934645 PMID: 19846039 PMID: 19809210 PMID: 19808329 PMID: 19796352 PMID: 19763046 PMID: 19738530 PMID: 19700049 PMID: 19667252 PMID: 19653009 PMID: 19627718 PMID: 19604574 PMID: 19603612 PMID: 19602777 PMID: 19573382 PMID: 19546096 PMID: 19545779 PMID: 19473337 PMID: 19472864 PMID: 19434333 PMID: 19427162 PMID: 19397801 PMID: 19394851 PMID: 19376400 PMID: 19370326 PMID: 19349318 PMID: 19328965 PMID: 19287809 PMID: 19276506 PMID: 19210213 PMID: 19207219 PMID: 19148372 PMID: 19142383 PMID: 19137795 PMID: 19125641 PMID: 19092322 PMID: 19085801 PMID: 19075541 PMID: 19039552 PMID: 19029467 PMID: 19001357 PMID: 18942531 PMID: 18842162 PMID: 18835781 PMID: 18813769 PMID: 18795069 PMID: 18788267 PMID: 18763005 PMID: 18752767 PMID: 18721528 PMID: 18713266 PMID: 18690380 PMID: 18686210 PMID: 18678314 PMID: 18678207 PMID: 18655459 PMID: 18651846 PMID: 18649821 PMID: 18633565 PMID: 18632171 PMID: 18607686 PMID: 18603902 PMID: 18592937 PMID: 18533584 PMID: 18504616 PMID: 18475217 PMID: 18471462 PMID: 18469010 PMID: 18467806 PMID: 18439181 PMID: 18433709 PMID: 18367009 PMID: 18358976 PMID: 18346349 PMID: 18344067 PMID: 18344061 PMID: 18342686 PMID: 18322666 PMID: 18300519 PMID: 18279769 PMID: 18279400 PMID: 18277575 PMID: 18256568 PMID: 18254468 PMID: 18176361 PMID: 18098343 PMID: 18098098 PMID: 18022092 PMID: 17940554 PMID: 17938769 PMID: 17926897 PMID: 17925629 PMID: 17682844 PMID: 17921333 PMID: 17896511 PMID: 17891435 PMID: 17891417 PMID: 17890693 PMID: 17889185 PMID: 17889116 PMID: 17885524 PMID: 17882369 PMID: 17721178 PMID: 17646730 PMID: 17643880 PMID: 17642136 PMID: 17622380 PMID: 17610345 PMID: 17592472 PMID: 17579251 PMID: 17578029 PMID: 17571398 PMID: 19205577 PMID: 17525381 PMID: 17498023 PMID: 17493477 PMID: 17492847 PMID: 17485520 PMID: 17469331 PMID: 17447196 PMID: 17427537 PMID: 17289480 PMID: 17242658 PMID: 17208320 PMID: 17203795 PMID: 17201269 PMID: 17171992 PMID: 17115967 PMID: 17102833 PMID: 17096077 PMID: 17091798 PMID: 17089245 PMID: 17088462 PMID: 17027099 PMID: 17023572 PMID: 16978158 PMID: 16949038 PMID: 16939832 PMID: 16908774 PMID: 16890305 PMID: 16883266 PMID: 16867874 PMID: 16858174 PMID: 16839424 PMID: 16816434 PMID: 16808984 PMID: 16808138 PMID: 16760201 PMID: 16723792 PMID: 16714771 PMID: 16703221 PMID: 16699305 PMID: 16678576 PMID: 16648482 PMID: 16645420 PMID: 16645383 PMID: 16645368 PMID: 16612070 PMID: 16585965 PMID: 16585666 PMID: 16569542 PMID: 16563104 PMID: 16538434 PMID: 16524176 PMID: 16472484 PMID: 16462722 PMID: 16446230 PMID: 16444925 PMID: 16436898 PMID: 16418253 PMID: 16386612 PMID: 16344368 PMID: 16326679 PMID: 16316398 PMID: 16285604 PMID: 16283028 PMID: 16255661 PMID: 16253730 PMID: 16242489 PMID: 16218427 PMID: 16215357 PMID: 19641683 PMID: 16189637 PMID: 16164878 PMID: 16091665 PMID: 16084340 PMID: 16084277 PMID: 16080792 PMID: 16026319 PMID: 15999470 PMID: 15998712 PMID: 15982486 PMID: 15947541 PMID: 15940440 PMID: 15909747 PMID: 15897732 PMID: 15896768 PMID: 15888841 PMID: 15866670 PMID: 15864294 PMID: 15864004 PMID: 15818120 PMID: 15773420 PMID: 15755465 PMID: 15736669 PMID: 15724418 PMID: 15724385 PMID: 15716981 PMID: 15710287 PMID: 15687116 PMID: 15660737 PMID: 15617639 PMID: 15607676 PMID: 15605834 PMID: 15591764 PMID: 15508792 PMID: 15492895 PMID: 15473480 PMID: 15449973 PMID: 15381653 PMID: 15378803 PMID: 15373989 PMID: 15346692 PMID: 15329269 PMID: 15306231 PMID: 15302010 PMID: 15302000 PMID: 15289469 PMID: 15231366 PMID: 15206485 PMID: 15200781 PMID: 15185463 PMID: 15146952 PMID: 15144333 PMID: 15086040 PMID: 15082279 PMID: 15078808 PMID: 15024368 PMID: 15018189 PMID: 14996479 PMID: 14984725 PMID: 14961954 PMID: 14871039 PMID: 14871037 PMID: 14755975 PMID: 14754423 PMID: 14750751 PMID: 14726055 PMID: 14699692 PMID: 14690422 PMID: 14642194 PMID: 14612845 PMID: 14577573 PMID: 14564332 PMID: 14533544 PMID: 14517556 PMID: 14504186 PMID: 14502297 PMID: 12918898 PMID: 12906166 PMID: 12879763 PMID: 12870530 PMID: 12870526 PMID: 12808483 PMID: 12792925 PMID: 12754029 PMID: 12743013 PMID: 12732279 PMID: 12720488 PMID: 12673964 PMID: 12624699 PMID: 12566118 PMID: 12556677 PMID: 12555494 PMID: 12555392 PMID: 12532541 PMID: 12516240 PMID: 12511534 PMID: 12507258 PMID: 12463096 PMID: 12461319 PMID: 12440520 PMID: 12437500 PMID: 12390712 PMID: 12383574 PMID: 12356340 PMID: 12221062 PMID: 12215275 PMID: 12208410 PMID: 12189287 PMID: 12177436 PMID: 12164087 PMID: 12163901 PMID: 12152522 PMID: 12118898 PMID: 12116803 PMID: 12105137 PMID: 12102213 PMID: 12101796 PMID: 12100504 PMID: 12070559 PMID: 12034159 PMID: 12032481 PMID: 11992024 PMID: 11988637 PMID: 11976781 PMID: 11968156 PMID: 11957798 PMID: 11885884 PMID: 11873750 PMID: 11858111 PMID: 11748268 PMID: 11743243 PMID: 11741555 PMID: 11721723 PMID: 11716328 PMID: 11715333 PMID: 11714886 PMID: 11711528 PMID: 11701629 PMID: 11696268 PMID: 11687986 PMID: 11686913 PMID: 11582624 PMID: 11559683 PMID: 11556162 PMID: 11528624 PMID: 11513912 PMID: 11478813 PMID: 11476749 PMID: 11461981 PMID: 11448809 PMID: 11435335 PMID: 11433813 PMID: 11431676 PMID: 11414601 PMID: 11386592 PMID: 11378003 PMID: 11378000 PMID: 11376626 PMID: 11370810 PMID: 11309531 PMID: 11309529 PMID: 11261335 PMID: 11246053 PMID: 11236523 PMID: 11206720 PMID: 11186995 PMID: 11174904 PMID: 11110429 PMID: 11078425 PMID: 11070757 PMID: 11068713 PMID: 11030472 PMID: 11006887 PMID: 10989746 PMID: 10983679 PMID: 10962762 PMID: 10962115 PMID: 10953835 PMID: 10944991 PMID: 10908024 PMID: 10894901 PMID: 10885317 PMID: 10859992 PMID: 10856392 PMID: 10832133 PMID: 10820962 PMID: 10783225 PMID: 10773239 PMID: 10729073 PMID: 10724045 PMID: 10721644 PMID: 10670797 PMID: 10614147 PMID: 10596717 PMID: 10587341 PMID: 10579734 PMID: 10573087 PMID: 10560143 PMID: 10551705 PMID: 10547093 PMID: 10546133 PMID: 10533665 PMID: 10503066 PMID: 10502594 PMID: 10490095 PMID: 10488455 PMID: 10484868 PMID: 10464508 PMID: 10454788 PMID: 10452302 PMID: 10420871 PMID: 10348539 PMID: 10347957 PMID: 10343080 PMID: 10335857 PMID: 10232447 PMID: 10230484 PMID: 10226904 PMID: 10226898 PMID: 10224210 PMID: 10220102 PMID: 10213279 PMID: 10204815 PMID: 10194660 PMID: 10099912 PMID: 10097300 PMID: 10086565 PMID: 10084376 PMID: 9880221 PMID: 9869999 PMID: 9861588 PMID: 9859148 PMID: 9853179 PMID: 9842409 PMID: 9838502 PMID: 9822143 PMID: 9809957 PMID: 9788827 PMID: 9771014 PMID: 9741515 PMID: 9727680 PMID: 9711886 PMID: 9665216 PMID: 9656083 PMID: 9642838 PMID: 9639381 PMID: 9617657 PMID: 9614664 PMID: 9609007 PMID: 9604963 PMID: 9595502 PMID: 9594370 PMID: 9587210 PMID: 9488948 PMID: 9488209 PMID: 9472994 PMID: 9458452 PMID: 9431448 PMID: 9348632 PMID: 9410910 PMID: 9410774 PMID: 9360074 PMID: 9359048 PMID: 9358513 PMID: 9327710 PMID: 9339958 PMID: 9286500 PMID: 9313597 PMID: 9285151 PMID: 9260976 PMID: 9256850 PMID: 9226290 PMID: 9358670 PMID: 9333722 PMID: 9259727 PMID: 9231820 PMID: 9444024 PMID: 9234054 PMID: 9202685 PMID: 9160786 PMID: 9154957 PMID: 9090671 PMID: 10895236 PMID: 9490376 PMID: 9333490 PMID: 9097483 PMID: 9057067 PMID: 10352469 PMID: 9489129 PMID: 9050817 PMID: 9239870 PMID: 9064204 PMID: 8953402 PMID: 8901758 PMID: 9072069 PMID: 8938701 PMID: 8806340 PMID: 8756879 PMID: 8752195 PMID: 8752190 PMID: 8964117 PMID: 8803443 PMID: 8798110 PMID: 8798109 PMID: 8645643 PMID: 8774633 PMID: 8763644 PMID: 8610701 PMID: 8614340 PMID: 8674106 PMID: 8650400 PMID: 8668603 PMID: 8785402 PMID: 8579020 PMID: 8865884 PMID: 8681317 PMID: 8645890 PMID: 7495217 PMID: 8598627 PMID: 8526603 PMID: 7475210 PMID: 8682229 PMID: 8682221 PMID: 8678701 PMID: 8522394 PMID: 8519705 PMID: 7656290 PMID: 7655849 PMID: 7653790 PMID: 7595857 PMID: 7786655 PMID: 7649665 PMID: 7646289 PMID: 7642066 PMID: 7774864 PMID: 7487315 PMID: 7832102 PMID: 7813265 PMID: 7760372 PMID: 7676759 PMID: 7857740 PMID: 7713099 PMID: 7842466 PMID: 7807681 PMID: 7815010 PMID: 7968889 PMID: 10147474 PMID: 7801669 PMID: 7786119 PMID: 8055742 PMID: 7995267 PMID: 7988601 PMID: 7971396 PMID: 8020188 PMID: 7956986 PMID: 7926384 PMID: 7918816 PMID: 8197986 PMID: 8184813 PMID: 8047317 PMID: 8046768 PMID: 7730876 PMID: 8210030 PMID: 8058185 PMID: 8207870 PMID: 8204795 PMID: 7842264 PMID: 8306847 PMID: 8173141 PMID: 8127811 PMID: 8205273 PMID: 8149190 PMID: 7811151 PMID: 7752828 PMID: 8146049 PMID: 8122748 PMID: 8312006 PMID: 8376702 PMID: 8362719 PMID: 8241219 PMID: 8233996 PMID: 7922220 PMID: 8218788 PMID: 8379245 PMID: 8351938 PMID: 8477526 PMID: 8470613 PMID: 8465357 PMID: 8370206 PMID: 8498805 PMID: 8452906 PMID: 8443905 PMID: 8365751 PMID: 8434171 PMID: 8418995 PMID: 8465654 PMID: 8432290 PMID: 8309177 PMID: 8246409 PMID: 8009036 PMID: 8009029 PMID: 7966956 PMID: 1334684 PMID: 1307087 PMID: 1300570 PMID: 1338502 PMID: 1395210 PMID: 1298568 PMID: 1634681 PMID: 1498138 PMID: 1515288 PMID: 1580444 PMID: 1625355 PMID: 1340721 PMID: 1618198 PMID: 1640665 PMID: 1588519 PMID: 1563851 PMID: 1532353 PMID: 1348727 PMID: 1311717 PMID: 1734552 PMID: 1527934 PMID: 1491580 PMID: 1630665 PMID: 1589606 PMID: 1535390 PMID: 1485979 PMID: 1438899 PMID: 1345107 PMID: 1342831 PMID: 1323259 PMID: 1291711 PMID: 1959197 PMID: 1817203 PMID: 1687118 PMID: 1683519 PMID: 1742825 PMID: 1955646 PMID: 1838741 PMID: 1807273 PMID: 1724233 PMID: 1926945 PMID: 1861916 PMID: 1832984 PMID: 10147590 PMID: 2045161 PMID: 1770747 PMID: 1854762 PMID: 2060902 PMID: 2046251 PMID: 2020139 PMID: 1878080 PMID: 1827978 PMID: 1808940 PMID: 1789050 PMID: 1743025 PMID: 1711971 PMID: 2133867 PMID: 2104430 PMID: 2226286 PMID: 2121813 PMID: 2148434 PMID: 2237043 PMID: 2234473 PMID: 2258679 PMID: 2232465 PMID: 2144067 PMID: 2391933 PMID: 2268858 PMID: 2188716 PMID: 2172581 PMID: 2316456 PMID: 2334884 PMID: 2139381 PMID: 11527116 PMID: 2344226 PMID: 2339587 PMID: 2293752 PMID: 2293351 PMID: 2274876 PMID: 2209022 PMID: 2098849 PMID: 2093753 PMID: 2091237 PMID: 2805308 PMID: 2614553 PMID: 2801484 PMID: 2815893 PMID: 2764296 PMID: 2799024 PMID: 2732298 PMID: 2595796 PMID: 2698711 PMID: 2674445 PMID: 2770093 PMID: 2659182 PMID: 2499991 PMID: 2705634 PMID: 2500909 PMID: 2924787 PMID: 2724763 PMID: 2523768 PMID: 2536513 PMID: 2800999 PMID: 2778643 PMID: 2716168 PMID: 2702015 PMID: 2698073 PMID: 2663011 PMID: 2651234 PMID: 2530617 PMID: 3244266 PMID: 3229865 PMID: 3074596 PMID: 3228815 PMID: 3195174 PMID: 2976854 PMID: 3143848 PMID: 3389301 PMID: 3051139 PMID: 3405780 PMID: 3249261 PMID: 2843018 PMID: 2967953 PMID: 3136723 PMID: 3392876 PMID: 3356077 PMID: 3279665 PMID: 3078539 PMID: 3075397 PMID: 2975926 PMID: 2963965 PMID: 2905877 PMID: 3692071 PMID: 3618497 PMID: 3552306 PMID: 3499525 PMID: 3448172 PMID: 2821938 PMID: 3298872 PMID: 3592558 PMID: 3429922 PMID: 3814452 PMID: 3807720 PMID: 3616940 PMID: 3540834 PMID: 3536106 PMID: 3437485 PMID: 3426808 PMID: 2963451 PMID: 3501442 PMID: 2946747 PMID: 3820525 PMID: 2949100 PMID: 3093511 PMID: 3777821 PMID: 3732300 PMID: 2940856 PMID: 3302169 PMID: 2940080 PMID: 3948548 PMID: 3940829 PMID: 3948054 PMID: 3804771 PMID: 3760615 PMID: 3527184 PMID: 3472440 PMID: 2424202 PMID: 2944971 PMID: 4063000 PMID: 3003306 PMID: 4050704 PMID: 3935443 PMID: 3842641 PMID: 2866154 PMID: 3161925 PMID: 4014050 PMID: 4068326 PMID: 4036245 PMID: 4024573 PMID: 4039410 PMID: 4039291 PMID: 2986216 PMID: 4038422 PMID: 2857050 PMID: 4063082 PMID: 4012627 PMID: 3916478 PMID: 3884232 PMID: 3843585 PMID: 2936013 PMID: 2579609 PMID: 6241903 PMID: 6491072 PMID: 6093193 PMID: 6540593 PMID: 6517641 PMID: 6440064 PMID: 6382439 PMID: 6733886 PMID: 6399213 PMID: 6542928 PMID: 6431932 PMID: 6505349 PMID: 6525781 PMID: 6727081 PMID: 6395426 PMID: 6395282 PMID: 6241133 PMID: 6152107 PMID: 6084317 PMID: 6226198 PMID: 6361715 PMID: 6218739 PMID: 6603083 PMID: 6444343 PMID: 704514 |
| Drug metabolism | PMID: 21217867 PMID: 20444930 PMID: 20095790 PMID: 19734738 PMID: 19514965 PMID: 19403240 PMID: 19263460 PMID: 19199043 PMID: 18850180 PMID: 18720470 PMID: 18706002 PMID: 18345632 PMID: 18332082 PMID: 17635183 PMID: 17428010 PMID: 17094780 PMID: 16933064 PMID: 16763014 PMID: 16345128 PMID: 16316654 PMID: 16314882 PMID: 16035690 PMID: 16003296 PMID: 15947543 PMID: 15499165 PMID: 15354618 PMID: 15289791 PMID: 15174896 PMID: 14646689 PMID: 12946533 PMID: 12871202 PMID: 12570747 PMID: 12397705 PMID: 12016901 PMID: 11549291 PMID: 11523062 PMID: 11416429 PMID: 11364696 PMID: 11098416 PMID: 11094595 PMID: 11014323 PMID: 10975434 PMID: 10901697 PMID: 10893308 PMID: 10874050 PMID: 10760839 PMID: 10736610 PMID: 10535697 PMID: 10479090 PMID: 10453104 PMID: 9535200 PMID: 9535199 PMID: 9421115 PMID: 8799873 PMID: 8739024 PMID: 8941039 PMID: 7674268 PMID: 8641322 PMID: 8318717 PMID: 1429978 PMID: 1542102 PMID: 1878825 PMID: 2193809 PMID: 2190414 PMID: 2729127 PMID: 2703529 PMID: 2717519 PMID: 3192922 PMID: 3293397 PMID: 3178920 PMID: 3178919 PMID: 3094379 PMID: 3770017 PMID: 4082637 PMID: 3975262 PMID: 2862811 PMID: 6716694 PMID: 6862153 PMID: 7271909 PMID: 7216134 PMID: 7426273 PMID: 685725 PMID: 4438402 |
| ECM-receptor interaction | PMID: 18177501 |
| Endocytosis | PMID: 21104725 PMID: 20855896 PMID: 20719977 PMID: 20675381 PMID: 20644520 PMID: 20525693 PMID: 20472592 PMID: 20354761 PMID: 20204744 PMID: 20083574 PMID: 20013072 PMID: 19726542 PMID: 19644050 PMID: 19276088 PMID: 19193726 PMID: 19158399 PMID: 19007435 PMID: 18695394 PMID: 18691017 PMID: 18663158 PMID: 18550644 PMID: 18547994 PMID: 18524856 PMID: 18508877 PMID: 18448595 PMID: 18370232 PMID: 18212280 PMID: 17989357 PMID: 17673464 PMID: 17614937 PMID: 17605762 PMID: 17537986 PMID: 17535738 PMID: 17499813 PMID: 17487823 PMID: 17428839 PMID: 17405938 PMID: 17381423 PMID: 17380208 PMID: 17341626 PMID: 17322640 PMID: 17125841 PMID: 17085050 PMID: 17061452 PMID: 16788137 PMID: 16527989 PMID: 16478976 PMID: 16477034 PMID: 16428287 PMID: 16236259 PMID: 16234332 PMID: 16174284 PMID: 16172428 PMID: 16150899 PMID: 16103266 PMID: 15998700 PMID: 15797645 PMID: 15768830 PMID: 15563605 PMID: 15528469 PMID: 15516997 PMID: 15467006 PMID: 15339974 PMID: 15090867 PMID: 15090857 PMID: 14608358 PMID: 12920402 PMID: 12876377 PMID: 12684839 PMID: 12623967 PMID: 12460120 PMID: 12419801 PMID: 12027218 PMID: 11943747 PMID: 11278712 PMID: 11159195 PMID: 11158220 PMID: 10720933 PMID: 10385007 PMID: 10318798 PMID: 9845918 PMID: 9325269 PMID: 9231819 PMID: 8952695 PMID: 8904561 PMID: 8807023 PMID: 8782833 PMID: 7593249 PMID: 7810703 PMID: 7510767 PMID: 8424455 PMID: 1848506 PMID: 2178342 PMID: 2211876 PMID: 2847156 PMID: 3826297 PMID: 6323093 PMID: 7159501 PMID: 360069 PMID: 1275059 |
| Endometrial cancer | PMID: 21224825 PMID: 21209347 PMID: 21191832 PMID: 21173649 PMID: 21152841 PMID: 21112078 PMID: 20937645 PMID: 20846065 PMID: 20799515 PMID: 20714146 PMID: 20674961 PMID: 20670865 PMID: 20354525 PMID: 20096921 PMID: 19410280 PMID: 19401534 PMID: 19383893 PMID: 19332387 PMID: 19004400 PMID: 18992861 PMID: 18682485 PMID: 18642563 PMID: 18439075 PMID: 18087838 PMID: 18071194 PMID: 18021979 PMID: 17912243 PMID: 17825884 PMID: 17823739 PMID: 17689651 PMID: 17623559 PMID: 17427503 PMID: 17224856 PMID: 17168500 PMID: 17168483 PMID: 17139988 PMID: 17045328 PMID: 17009635 PMID: 16963204 PMID: 16870694 PMID: 16639730 PMID: 16556476 PMID: 16378192 PMID: 15738027 PMID: 15703772 PMID: 15527901 PMID: 15497909 PMID: 15483212 PMID: 15340658 PMID: 15339771 PMID: 15304163 PMID: 15178898 PMID: 15163305 PMID: 15107259 PMID: 14979602 PMID: 14766262 PMID: 14690786 PMID: 12955892 PMID: 12916269 PMID: 12885450 PMID: 12821356 PMID: 12722379 PMID: 12704677 PMID: 12691310 PMID: 12640672 PMID: 12566908 PMID: 11883289 PMID: 11844363 PMID: 11604236 PMID: 11518897 PMID: 11476557 PMID: 11339920 PMID: 11242642 PMID: 11240663 PMID: 11235033 PMID: 11142531 PMID: 11065007 PMID: 10977477 PMID: 10858183 PMID: 10842912 PMID: 10789224 PMID: 10739499 PMID: 10730382 PMID: 10710204 PMID: 10601598 PMID: 10582403 PMID: 10535788 PMID: 10454461 PMID: 10392590 PMID: 10368519 PMID: 10352918 PMID: 10346659 PMID: 10225441 PMID: 10021299 PMID: 9917950 PMID: 9883070 PMID: 9695322 PMID: 9679207 PMID: 9607348 PMID: 9578282 PMID: 9454393 PMID: 9270408 PMID: 9499009 PMID: 9499008 PMID: 9117140 PMID: 8979202 PMID: 8869880 PMID: 8720976 PMID: 8634998 PMID: 7590470 PMID: 7732953 PMID: 7766340 PMID: 8747392 PMID: 7995766 PMID: 7668068 PMID: 8999698 PMID: 7959270 PMID: 9073770 PMID: 12287980 PMID: 8200846 PMID: 8194655 PMID: 16353609 PMID: 8092764 PMID: 7921803 PMID: 8120860 PMID: 8347781 PMID: 7686914 PMID: 12179509 PMID: 8460621 PMID: 8435805 PMID: 8281709 PMID: 1468592 PMID: 1437913 PMID: 1442985 PMID: 1605587 PMID: 1317637 PMID: 1596431 PMID: 1501996 PMID: 1469237 PMID: 1414073 PMID: 1815841 PMID: 1936493 PMID: 2031492 PMID: 2026352 PMID: 2010009 PMID: 1989919 PMID: 2227571 PMID: 2215269 PMID: 2170823 PMID: 2210309 PMID: 2270983 PMID: 2145895 PMID: 2696133 PMID: 2614174 PMID: 2695994 PMID: 2591436 PMID: 2909449 PMID: 2648543 PMID: 2644761 PMID: 2577197 PMID: 3225079 PMID: 3193014 PMID: 3283471 PMID: 3072880 PMID: 3311130 PMID: 3602806 PMID: 3102532 PMID: 3826217 PMID: 3559319 PMID: 3543907 PMID: 3497500 PMID: 3330733 PMID: 3314396 PMID: 3002600 PMID: 3518931 PMID: 3720786 PMID: 4086966 PMID: 4024548 PMID: 6383739 PMID: 6089441 PMID: 12313083 PMID: 6497587 PMID: 6371616 PMID: 6668673 PMID: 6556006 PMID: 6824255 PMID: 6822361 PMID: 6637403 PMID: 6130992 PMID: 12311809 PMID: 6214734 PMID: 7097914 PMID: 7039906 PMID: 7033575 PMID: 12263973 PMID: 12279153 PMID: 7022279 PMID: 7272975 PMID: 7249946 PMID: 7019794 PMID: 7246086 PMID: 6944429 PMID: 7448715 PMID: 7454090 PMID: 7202690 PMID: 7442552 PMID: 7445154 PMID: 7429139 PMID: 6992895 PMID: 6245580 PMID: 7413728 PMID: 12279276 PMID: 12309932 PMID: 443293 PMID: 218369 PMID: 371370 PMID: 210085 PMID: 359420 PMID: 333120 PMID: 197509 PMID: 866107 PMID: 866106 PMID: 870690 PMID: 323098 PMID: 1002092 PMID: 1028554 PMID: 12334809 PMID: 963173 PMID: 984096 PMID: 1258036 PMID: 1021008 PMID: 1203697 PMID: 1186789 PMID: 1189776 PMID: 4457398 PMID: 4600328 PMID: 4727679 PMID: 4777226 PMID: 12229384 PMID: 6016348 PMID: 14093620 |
| Epithelial cell signaling in Helicobacter pylori infection |  |
| ErbB signaling pathway |  |
| Fc epsilon RI signaling pathway |  |
| Fc gamma R-mediated phagocytosis |  |
| Focal adhesion | PMID: 21068519 PMID: 21048023 PMID: 20932596 PMID: 20805441 PMID: 20663583 PMID: 20019333 PMID: 19734358 PMID: 19276129 PMID: 19151249 PMID: 18981321 PMID: 18956652 PMID: 18955661 PMID: 18829614 PMID: 18486177 PMID: 18319309 PMID: 18195161 PMID: 18194601 PMID: 18182825 PMID: 18177501 PMID: 18075463 PMID: 18047620 PMID: 17913382 PMID: 17406055 PMID: 17369469 PMID: 17293683 PMID: 17188706 PMID: 16870827 PMID: 16707113 PMID: 16594906 PMID: 16574795 PMID: 16244766 PMID: 15894164 PMID: 15886352 PMID: 15719257 PMID: 15563577 PMID: 15203192 PMID: 14967842 PMID: 14613935 PMID: 14506307 PMID: 12732587 PMID: 12719447 PMID: 12676164 PMID: 12124218 PMID: 12111044 PMID: 11942577 PMID: 11882598 PMID: 11882594 PMID: 11847190 PMID: 11788392 PMID: 11600408 PMID: 11408396 PMID: 11262415 PMID: 11204444 PMID: 11208772 PMID: 11121380 PMID: 11062326 PMID: 10715259 PMID: 10642310 PMID: 10486664 PMID: 9931105 PMID: 9892142 PMID: 9886878 PMID: 9719064 PMID: 9596072 PMID: 9514420 PMID: 9453297 PMID: 9212588 PMID: 9130441 PMID: 9023046 PMID: 8575261 |
| Fructose and mannose metabolism | PMID: 19258383 PMID: 11707454 PMID: 14254528 |
| Galactose metabolism | PMID: 9564659 |
| Gap junction | PMID: 21040688 PMID: 20164205 PMID: 19686729 PMID: 19470887 PMID: 19411610 PMID: 18834327 PMID: 18831678 PMID: 18632992 PMID: 18543397 PMID: 18373396 PMID: 18373394 PMID: 17613133 PMID: 18045155 PMID: 17922338 PMID: 17882239 PMID: 17785925 PMID: 17416596 PMID: 17322640 PMID: 17255527 PMID: 17223729 PMID: 17146929 PMID: 16952587 PMID: 15605115 PMID: 15528934 PMID: 15094354 PMID: 12566216 PMID: 12521932 PMID: 12468583 PMID: 11856964 PMID: 11847193 PMID: 11422751 PMID: 10785509 PMID: 10775308 PMID: 10523388 PMID: 10397678 PMID: 9843730 PMID: 9689445 PMID: 9635160 PMID: 9433513 PMID: 8697713 PMID: 8586804 PMID: 7584912 PMID: 8222085 PMID: 8428777 PMID: 7078093 |
| Glioma | PMID: 21232201 PMID: 21214290 PMID: 21209769 PMID: 20921456 PMID: 20703889 PMID: 20665891 PMID: 20479404 PMID: 20173326 PMID: 19938667 PMID: 19920819 PMID: 19916846 PMID: 19772409 PMID: 19601816 PMID: 19537027 PMID: 19365997 PMID: 19309540 PMID: 19293489 PMID: 19007739 PMID: 18940042 PMID: 18838327 PMID: 18810924 PMID: 18715950 PMID: 18647179 PMID: 18639166 PMID: 18063929 PMID: 18044225 PMID: 18020927 PMID: 17301720 PMID: 17217202 PMID: 17134648 PMID: 17041828 PMID: 17019386 PMID: 16871745 PMID: 16776435 PMID: 16770809 PMID: 16724823 PMID: 16710995 PMID: 16585887 PMID: 16531197 PMID: 16495922 PMID: 16389565 PMID: 16376285 PMID: 16368514 PMID: 16364614 PMID: 16326274 PMID: 16156235 PMID: 16134300 PMID: 15995830 PMID: 15989760 PMID: 15921750 PMID: 15696424 PMID: 15277267 PMID: 15200149 PMID: 15178645 PMID: 15052773 PMID: 14978474 PMID: 14969747 PMID: 14743432 PMID: 14740261 PMID: 14500184 PMID: 12894278 PMID: 12778010 PMID: 12715191 PMID: 12672279 PMID: 12191496 PMID: 12173391 PMID: 11898512 PMID: 11720480 PMID: 11678349 PMID: 11560039 PMID: 11223164 PMID: 10965271 PMID: 10945815 PMID: 10874128 PMID: 10854981 PMID: 10730065 PMID: 10478374 PMID: 10428437 PMID: 10089996 PMID: 9802850 PMID: 9740044 PMID: 9698732 PMID: 9520084 PMID: 9018030 PMID: 8990430 PMID: 9091772 PMID: 8915311 PMID: 8903081 PMID: 8738401 PMID: 8552814 PMID: 7644408 PMID: 8751291 PMID: 8027795 PMID: 8064789 PMID: 8146534 PMID: 8351439 PMID: 8301376 PMID: 8485652 PMID: 1595037 PMID: 1570077 PMID: 1338925 PMID: 1319669 PMID: 1813265 PMID: 1910950 PMID: 1895165 PMID: 1901597 PMID: 2003744 PMID: 1749475 PMID: 1661856 PMID: 2076352 PMID: 2358853 PMID: 2334169 PMID: 1709458 PMID: 2569566 PMID: 2711819 PMID: 3244034 PMID: 3171170 PMID: 3267325 PMID: 2835913 PMID: 3596787 PMID: 3563857 PMID: 3819830 PMID: 3440874 PMID: 3463530 PMID: 3963852 PMID: 3762836 PMID: 4088398 PMID: 6334092 PMID: 6495954 PMID: 6738895 PMID: 6711220 PMID: 6371995 PMID: 6305729 PMID: 6881968 PMID: 6880492 PMID: 7110572 PMID: 6226696 PMID: 7199867 PMID: 7143083 PMID: 7233466 PMID: 7013370 PMID: 6269367 PMID: 6999845 PMID: 6256674 PMID: 227108 PMID: 531424 PMID: 230705 PMID: 100195 PMID: 892240 PMID: 1253798 PMID: 1015053 PMID: 1180012 PMID: 1111762 PMID: 1181810 PMID: 4455764 PMID: 4407719 PMID: 4765001 PMID: 5536667 PMID: 14316064 PMID: 14192306 PMID: 14371747 PMID: 14371500 |
| Glutathione metabolism | PMID: 21178975 PMID: 12411453 PMID: 8573205 PMID: 3243001 |
| Glycerolipid metabolism | PMID: 2767941 |
| Glycine, serine and threonine metabolism | PMID: 21060000 PMID: 20018872 PMID: 15200428 PMID: 2312039 |
| Glycolysis / Gluconeogenesis |  |
| Glyoxylate and dicarboxylate metabolism |  |
| GnRH signaling pathway |  |
| Graft-versus-host disease | PMID: 21178709 PMID: 21154984 PMID: 20961888 PMID: 20851864 PMID: 20735807 PMID: 20376502 PMID: 20181110 PMID: 20153836 PMID: 20067905 PMID: 19765466 PMID: 19660723 PMID: 19352305 PMID: 19349209 PMID: 19338041 PMID: 19318596 PMID: 19225223 PMID: 19203551 PMID: 19041058 PMID: 19264209 PMID: 18940687 PMID: 18688653 PMID: 18346325 PMID: 18215783 PMID: 18031144 PMID: 17850443 PMID: 17699273 PMID: 17342159 PMID: 17057649 PMID: 16513536 PMID: 16266922 PMID: 16138351 PMID: 15833186 PMID: 15806118 PMID: 15567546 PMID: 15531904 PMID: 15531898 PMID: 14739682 PMID: 14631624 PMID: 14605498 PMID: 12790696 PMID: 12544782 PMID: 11704797 PMID: 11545420 PMID: 11384585 PMID: 11100753 PMID: 10959906 PMID: 10918426 PMID: 10849532 PMID: 10792274 PMID: 10730553 PMID: 10516680 PMID: 10233877 PMID: 10064182 PMID: 9785975 PMID: 9746768 PMID: 9746766 PMID: 9720734 PMID: 9695669 PMID: 9466284 PMID: 9436547 PMID: 9240551 PMID: 9209738 PMID: 8896434 PMID: 8832009 PMID: 7491699 PMID: 7815808 PMID: 8301434 PMID: 8205090 PMID: 8110873 PMID: 8312344 PMID: 7693907 PMID: 8333042 PMID: 8438907 PMID: 7679172 PMID: 1440852 PMID: 1768975 PMID: 1871805 PMID: 2015407 PMID: 1848265 PMID: 1783253 PMID: 2402791 PMID: 2368150 PMID: 2193442 PMID: 2671437 PMID: 3046689 PMID: 3048498 PMID: 3058105 PMID: 3554661 PMID: 3332119 PMID: 3888312 PMID: 3882141 PMID: 3906282 PMID: 6342656 PMID: 6342655 PMID: 6807391 |
| Hedgehog signaling pathway | PMID: 17016550 |
| Hematopoietic cell lineage |  |
| Huntington's disease | PMID: 18728845 PMID: 18034588 PMID: 16968947 PMID: 12385592 PMID: 7765749 PMID: 7969213 PMID: 9719668 PMID: 2957021 |
| Hypertrophic cardiomyopathy (HCM) | PMID: 21163864 PMID: 21143986 PMID: 21092643 PMID: 20597231 PMID: 20594303 PMID: 20582898 PMID: 20433544 PMID: 20362415 PMID: 20331697 PMID: 20226627 PMID: 20156645 PMID: 20137368 PMID: 20047131 PMID: 19889657 PMID: 19838000 PMID: 19419401 PMID: 19181403 PMID: 19147047 PMID: 19096110 PMID: 18651846 PMID: 18513529 PMID: 18254468 PMID: 18177389 PMID: 18022114 PMID: 17667752 PMID: 17653030 PMID: 17651095 PMID: 17579564 PMID: 17411275 PMID: 17337597 PMID: 17063702 PMID: 16949038 PMID: 16808984 PMID: 16723792 PMID: 16506641 PMID: 16377293 PMID: 16307805 PMID: 16260052 PMID: 15959967 PMID: 15902928 PMID: 15832975 PMID: 15583080 PMID: 15243850 PMID: 15226630 PMID: 15220895 PMID: 15144333 PMID: 15118286 PMID: 15058760 PMID: 14621181 PMID: 14577573 PMID: 19641710 PMID: 12925456 PMID: 12452323 PMID: 12197596 PMID: 11869178 PMID: 11847170 PMID: 11786155 PMID: 11751703 PMID: 11469422 PMID: 10959455 PMID: 10948080 PMID: 10803446 PMID: 10636256 PMID: 10560530 PMID: 10362077 PMID: 10096034 PMID: 9892772 PMID: 9822439 PMID: 9783650 PMID: 9783493 PMID: 9697824 PMID: 9657474 PMID: 9601485 PMID: 9368664 PMID: 9283537 PMID: 9283227 PMID: 9261931 PMID: 9166987 PMID: 9023164 PMID: 8934365 PMID: 10830045 PMID: 8964117 PMID: 9062591 PMID: 8865685 PMID: 8727703 PMID: 7641357 PMID: 8820048 PMID: 7614493 PMID: 7861646 PMID: 7967013 PMID: 7995267 PMID: 7955572 PMID: 8020188 PMID: 8207870 PMID: 8309048 PMID: 8187113 PMID: 8174144 PMID: 8158529 PMID: 8336412 PMID: 8505866 PMID: 8416759 PMID: 1620998 PMID: 1533688 PMID: 1307583 PMID: 1920945 PMID: 1920958 PMID: 1824622 PMID: 1715467 PMID: 2388399 PMID: 2172581 PMID: 2146419 PMID: 2144601 PMID: 2338765 PMID: 2151776 PMID: 2137778 PMID: 2615173 PMID: 2533275 PMID: 3405780 PMID: 3249261 PMID: 2975702 PMID: 3552306 PMID: 3429921 PMID: 2953823 PMID: 3782885 PMID: 3722878 PMID: 3841886 PMID: 3831023 PMID: 4040319 PMID: 4093619 PMID: 4093618 PMID: 2936836 PMID: 2936835 PMID: 3158127 PMID: 3157012 PMID: 4067357 PMID: 6542928 PMID: 6543588 PMID: 6236268 PMID: 6678291 PMID: 6235295 PMID: 6687125 PMID: 6682019 PMID: 6222206 PMID: 6685744 PMID: 6304397 PMID: 6218739 PMID: 6892223 PMID: 6459467 PMID: 7201489 PMID: 7199148 PMID: 7190882 PMID: 571311 |
| Inositol phosphate metabolism | PMID: 9812839 PMID: 1334617 PMID: 2167204 |
| Insulin signaling pathway | PMID: 18855718 PMID: 18570670 PMID: 18193490 PMID: 17660951 PMID: 17646573 PMID: 16877964 PMID: 16389635 PMID: 15930959 PMID: 15657091 PMID: 15573143 PMID: 15504952 PMID: 12876303 PMID: 12837769 PMID: 12770608 PMID: 11884265 PMID: 11246877 PMID: 10535386 |
| Intestinal immune network for IgA production |  |
| Jak-STAT signaling pathway | PMID: 16534557 PMID: 16271517 PMID: 11799081 |
| Leukocyte transendothelial migration |  |
| Linoleic acid metabolism | PMID: 2150554 PMID: 4046319 |
| Long-term depression | PMID: 18704488 PMID: 10454212 |
| Long-term potentiation | PMID: 20870745 PMID: 20463749 PMID: 20351978 PMID: 19038294 PMID: 18957307 PMID: 18704488 PMID: 18649973 PMID: 18563301 PMID: 18386186 PMID: 17981707 PMID: 17653963 PMID: 17099064 PMID: 16280604 PMID: 16005635 PMID: 14732737 PMID: 14635713 PMID: 12504912 PMID: 11743138 PMID: 11448869 PMID: 10454212 PMID: 9704997 PMID: 7709342 |
| Lysine degradation |  |
| Lysosome | PMID: 21148428 PMID: 20865352 PMID: 19216915 PMID: 19193720 PMID: 18840681 PMID: 18391110 PMID: 18254660 PMID: 17485520 PMID: 16914343 PMID: 16368298 PMID: 16103266 PMID: 2233116 PMID: 2782425 |
| MAPK signaling pathway |  |
| Melanogenesis | PMID: 17318350 PMID: 15592791 PMID: 10771134 PMID: 9098273 |
| Melanoma | PMID: 21088057 PMID: 20924644 PMID: 20797563 PMID: 20808512 PMID: 20797650 PMID: 20615627 PMID: 20462842 PMID: 20449716 PMID: 20389300 PMID: 20375744 PMID: 20130803 PMID: 20103667 PMID: 20079924 PMID: 20029086 PMID: 19810102 PMID: 19775358 PMID: 19751881 PMID: 19711088 PMID: 19640634 PMID: 19637344 PMID: 19590411 PMID: 19527424 PMID: 19513949 PMID: 19508498 PMID: 19508146 PMID: 19138020 PMID: 19090009 PMID: 19069686 PMID: 18724134 PMID: 18664608 PMID: 18660744 PMID: 18604743 PMID: 18516765 PMID: 18411200 PMID: 18392776 PMID: 18381947 PMID: 19388463 PMID: 18158632 PMID: 17987726 PMID: 17958572 PMID: 17767073 PMID: 17534686 PMID: 17448200 PMID: 17401623 PMID: 17365838 PMID: 17245831 PMID: 17144512 PMID: 17094536 PMID: 17088199 PMID: 17048454 PMID: 17016167 PMID: 17013090 PMID: 16978213 PMID: 16935777 PMID: 16885809 PMID: 16769837 PMID: 16705506 PMID: 16610571 PMID: 16603232 PMID: 16503817 PMID: 16428145 PMID: 16202828 PMID: 16138923 PMID: 16050403 PMID: 15922119 PMID: 15692891 PMID: 15523573 PMID: 15468307 PMID: 15448036 PMID: 15208268 PMID: 15115440 PMID: 14990812 PMID: 14665785 PMID: 14663384 PMID: 12778010 PMID: 12688317 PMID: 12673380 PMID: 12640358 PMID: 12507088 PMID: 12382150 PMID: 12381213 PMID: 12223386 PMID: 12161209 PMID: 12132662 PMID: 11958893 PMID: 11830516 PMID: 11766463 PMID: 11398885 PMID: 11384576 PMID: 11371484 PMID: 11240479 PMID: 10755526 PMID: 11152107 PMID: 10980767 PMID: 10763944 PMID: 10597976 PMID: 10319983 PMID: 10209730 PMID: 9887493 PMID: 9794733 PMID: 9406719 PMID: 9097979 PMID: 9070132 PMID: 9104728 PMID: 8987375 PMID: 8956786 PMID: 8964212 PMID: 8811495 PMID: 8559202 PMID: 8988460 PMID: 8852008 PMID: 7601653 PMID: 7738295 PMID: 7885967 PMID: 7812786 PMID: 7804528 PMID: 8065581 PMID: 8167515 PMID: 8299179 PMID: 8235234 PMID: 1292783 PMID: 1423283 PMID: 1638163 PMID: 1603235 PMID: 1742743 PMID: 1745458 PMID: 1719559 PMID: 1997690 PMID: 2074068 PMID: 2331447 PMID: 2179799 PMID: 2607078 PMID: 2620401 PMID: 2629900 PMID: 2644761 PMID: 3402317 PMID: 3696399 PMID: 3625258 PMID: 3322333 PMID: 3504098 PMID: 3034007 PMID: 2880436 PMID: 3490028 PMID: 2415150 PMID: 3965856 PMID: 6683904 PMID: 12311809 PMID: 12263973 PMID: 7322491 PMID: 7016302 PMID: 7243211 PMID: 7003568 PMID: 7413728 PMID: 7224805 PMID: 476298 PMID: 777032 PMID: 5053697 PMID: 4932769 PMID: 5777180 PMID: 5742069 PMID: 5937985 PMID: 14098350 |
| Metabolism of xenobiotics by cytochrome P450 | PMID: 16985099 PMID: 16824612 PMID: 16314882 PMID: 15379059 PMID: 10098907 PMID: 8521087 |
| Methane metabolism |  |
| mTOR signaling pathway | PMID: 20206401 PMID: 19375241 |
| Natural killer cell mediated cytotoxicity |  |
| Neuroactive ligand-receptor interaction |  |
| Neurotrophin signaling pathway | PMID: 19748836 PMID: 19268530 |
| N-Glycan biosynthesis |  |
| NOD-like receptor signaling pathway |  |
| Non-small cell lung cancer | PMID: 21119733 PMID: 21107290 PMID: 21079521 PMID: 20967300 PMID: 20881641 PMID: 20715425 PMID: 20681440 PMID: 20650686 PMID: 20516450 PMID: 20460557 PMID: 20439196 PMID: 20427348 PMID: 20407031 PMID: 20358384 PMID: 20356758 PMID: 20234816 PMID: 20225906 PMID: 20085937 PMID: 20028752 PMID: 19940466 PMID: 19917841 PMID: 19793713 PMID: 19652055 PMID: 19597027 PMID: 19433684 PMID: 19349511 PMID: 19349493 PMID: 19332730 PMID: 19319108 PMID: 19308410 PMID: 20716427 PMID: 19289369 PMID: 19261963 PMID: 19228742 PMID: 19138979 PMID: 19091548 PMID: 19081729 PMID: 18977094 PMID: 18971808 PMID: 18936474 PMID: 18827612 PMID: 18772832 PMID: 18650173 PMID: 18520293 PMID: 18536787 PMID: 18449004 PMID: 18411700 PMID: 18398152 PMID: 18379357 PMID: 18157597 PMID: 18055759 PMID: 17898810 PMID: 17892508 PMID: 17826629 PMID: 17825686 PMID: 17762342 PMID: 17671153 PMID: 17602060 PMID: 17573328 PMID: 17571910 PMID: 17532379 PMID: 17409986 PMID: 17327260 PMID: 17267325 PMID: 17239287 PMID: 17212999 PMID: 17143257 PMID: 16932737 PMID: 16476542 PMID: 15867233 PMID: 15560691 PMID: 15366568 PMID: 15217970 PMID: 15178812 PMID: 12655441 PMID: 12377883 PMID: 11955658 PMID: 11892431 PMID: 10758383 PMID: 10731757 PMID: 10585066 PMID: 9593699 PMID: 9331136 PMID: 9507688 PMID: 7844610 PMID: 8032544 PMID: 2626991 PMID: 2986559 |
| Notch signaling pathway | PMID: 20204737 PMID: 19644017 |
| Nucleotide excision repair | PMID: 18711149 |
| One carbon pool by folate |  |
| Oocyte meiosis |  |
| Other glycan degradation | PMID: 20796156 PMID: 20590836 PMID: 20516397 PMID: 20509295 PMID: 20431264 PMID: 20102853 PMID: 20056895 PMID: 20027123 PMID: 20010244 PMID: 19730394 PMID: 19697998 PMID: 19641984 PMID: 19626619 PMID: 19542880 PMID: 19358182 PMID: 19284125 PMID: 19164256 PMID: 19151107 PMID: 19137265 PMID: 18983578 PMID: 18645357 PMID: 18609357 PMID: 18374418 PMID: 18309462 PMID: 18271277 PMID: 18087711 PMID: 18079460 PMID: 17925502 PMID: 17907891 PMID: 17878927 PMID: 17873594 PMID: 17663194 PMID: 17592072 PMID: 17386347 PMID: 17295896 PMID: 17215147 PMID: 17201729 PMID: 17172009 PMID: 17108707 PMID: 16822831 PMID: 16815490 PMID: 16636461 PMID: 16616240 PMID: 16546836 PMID: 16488477 PMID: 16443676 PMID: 16239345 PMID: 16125272 PMID: 16048251 PMID: 15870842 PMID: 15670668 PMID: 15610228 PMID: 15583217 PMID: 15489930 PMID: 15384194 PMID: 15383480 PMID: 15358274 PMID: 15301793 PMID: 15139786 PMID: 15119221 PMID: 14975589 PMID: 14699687 PMID: 14520627 PMID: 12937216 PMID: 12923368 PMID: 12766119 PMID: 12763606 PMID: 12296852 PMID: 12187097 PMID: 12182967 PMID: 12182502 PMID: 11984747 PMID: 11716368 PMID: 11574733 PMID: 11466245 PMID: 11432087 PMID: 11426367 PMID: 11339861 PMID: 11330554 PMID: 11236301 PMID: 11078567 PMID: 10932925 PMID: 10910439 PMID: 10837347 PMID: 10833096 PMID: 10607479 PMID: 10562627 PMID: 10460952 PMID: 10401011 PMID: 10334819 PMID: 9870465 PMID: 9865419 PMID: 9627625 PMID: 9400374 PMID: 9370388 PMID: 9322992 PMID: 9280208 PMID: 9355812 PMID: 9139937 PMID: 8941033 PMID: 9102854 PMID: 8762086 PMID: 8860097 PMID: 8706356 PMID: 8994550 PMID: 7585300 PMID: 7574058 PMID: 8521759 PMID: 7536714 PMID: 7623627 PMID: 7982651 PMID: 8079184 PMID: 8074804 PMID: 7735948 PMID: 8280641 PMID: 8376269 PMID: 8317791 PMID: 8449675 PMID: 1381510 PMID: 1562054 PMID: 1733381 PMID: 1600642 PMID: 1535403 PMID: 1520075 PMID: 2066367 PMID: 1924500 PMID: 1722587 PMID: 1822594 PMID: 1773530 PMID: 1719732 PMID: 2154114 PMID: 2509378 PMID: 2665972 PMID: 2745299 PMID: 2610054 PMID: 2463882 PMID: 2901268 PMID: 3318374 PMID: 3599647 PMID: 3822595 PMID: 3812352 PMID: 3540163 PMID: 2447438 PMID: 3023589 PMID: 3086612 PMID: 2418382 PMID: 4023926 PMID: 6702821 PMID: 6202633 PMID: 7165494 PMID: 6216880 PMID: 6462119 PMID: 7032096 PMID: 7260236 PMID: 7239776 PMID: 7213998 PMID: 7002425 PMID: 197665 PMID: 1016096 PMID: 803560 PMID: 1089012 PMID: 4858770 PMID: 4222823 |
| Oxidative phosphorylation | PMID: 21157304 PMID: 20725711 PMID: 20705925 PMID: 20585107 PMID: 20577032 PMID: 20448039 PMID: 20187241 PMID: 20110695 PMID: 19859074 PMID: 19762685 PMID: 19595436 PMID: 19488738 PMID: 19474762 PMID: 19249331 PMID: 19107126 PMID: 19106112 PMID: 19001357 PMID: 18819888 PMID: 18563753 PMID: 18487445 PMID: 18357915 PMID: 18227801 PMID: 18220945 PMID: 18089947 PMID: 17705793 PMID: 17378771 PMID: 17102090 PMID: 16230522 PMID: 16230521 PMID: 15990946 PMID: 15917810 PMID: 15769456 PMID: 15375007 PMID: 14654753 PMID: 12626438 PMID: 12595753 PMID: 12494018 PMID: 12044763 PMID: 11581303 PMID: 11372718 PMID: 11297287 PMID: 11254897 PMID: 11237098 PMID: 11081764 PMID: 11006086 PMID: 10488290 PMID: 10323480 PMID: 9822642 PMID: 9633918 PMID: 9605387 PMID: 9525068 PMID: 9192388 PMID: 9192055 PMID: 2202752 PMID: 3682579 PMID: 2947755 PMID: 2982227 PMID: 6600506 PMID: 6212269 PMID: 425466 PMID: 1031989 PMID: 1215639 PMID: 4216318 PMID: 4156526 PMID: 4239804 PMID: 5997430 PMID: 13813339 |
| Pancreatic cancer | PMID: 21196000 PMID: 20978506 PMID: 20803052 PMID: 20606091 PMID: 20130876 PMID: 20005970 PMID: 19934390 PMID: 19858399 PMID: 19734629 PMID: 19661352 PMID: 19491904 PMID: 19476877 PMID: 19308045 PMID: 18836620 PMID: 18795656 PMID: 18379729 PMID: 18234644 PMID: 18208781 PMID: 18055853 PMID: 17589923 PMID: 17261417 PMID: 17212999 PMID: 17020506 PMID: 16258101 PMID: 16191762 PMID: 15793888 PMID: 15703772 PMID: 12749245 PMID: 12584914 PMID: 11883385 PMID: 11272218 PMID: 10907780 PMID: 10541354 PMID: 10520930 PMID: 10479227 PMID: 10465343 PMID: 9494414 PMID: 8529768 PMID: 7556816 PMID: 7781849 PMID: 7507450 PMID: 2795950 PMID: 2770683 PMID: 3566522 PMID: 3747162 PMID: 3997986 PMID: 7064976 PMID: 7189169 |
| Parkinson's disease | PMID: 21248778 PMID: 21195698 PMID: 21070662 PMID: 20970452 PMID: 20881573 PMID: 20859904 PMID: 20818667 PMID: 20817229 PMID: 20682521 PMID: 20669249 PMID: 20648415 PMID: 20404676 PMID: 20347450 PMID: 20205144 PMID: 20187241 PMID: 20184681 PMID: 20099319 PMID: 20051133 PMID: 19852154 PMID: 19827594 PMID: 19826822 PMID: 19768815 PMID: 19761277 PMID: 19740484 PMID: 19727546 PMID: 19720479 PMID: 19682943 PMID: 19660871 PMID: 19633703 PMID: 19587225 PMID: 19556271 PMID: 19508591 PMID: 19491335 PMID: 19427584 PMID: 19418293 PMID: 19250756 PMID: 19232169 PMID: 19082526 PMID: 18954496 PMID: 18787645 PMID: 18779056 PMID: 18729534 PMID: 18717182 PMID: 18607207 PMID: 18591113 PMID: 18574545 PMID: 18509646 PMID: 18420158 PMID: 18416166 PMID: 18405780 PMID: 18398911 PMID: 18385627 PMID: 18379735 PMID: 18221232 PMID: 18166127 PMID: 17980978 PMID: 17890445 PMID: 17850194 PMID: 17825848 PMID: 17708488 PMID: 17696890 PMID: 17640385 PMID: 17548551 PMID: 17522612 PMID: 17514358 PMID: 17512599 PMID: 17466338 PMID: 17397638 PMID: 17346129 PMID: 17334582 PMID: 17245831 PMID: 17202967 PMID: 17120714 PMID: 17101846 PMID: 17101845 PMID: 17024577 PMID: 16978905 PMID: 16967348 PMID: 16962949 PMID: 16955434 PMID: 16913991 PMID: 16893345 PMID: 16872231 PMID: 16648603 PMID: 16640797 PMID: 16507475 PMID: 16438660 PMID: 19219779 PMID: 16362830 PMID: 16212686 PMID: 16154793 PMID: 16106242 PMID: 16078956 PMID: 16037918 PMID: 16015598 PMID: 15912760 PMID: 15814870 PMID: 15779680 PMID: 15719417 PMID: 15620163 PMID: 15560307 PMID: 15477584 PMID: 15200203 PMID: 15197700 PMID: 15098342 PMID: 15090877 PMID: 15088263 PMID: 15088256 PMID: 15061824 PMID: 14753053 PMID: 14609309 PMID: 14524248 PMID: 14509040 PMID: 13130338 PMID: 12933939 PMID: 12850398 PMID: 12835329 PMID: 12758062 PMID: 12730251 PMID: 12720610 PMID: 12685002 PMID: 12634289 PMID: 12618058 PMID: 12590680 PMID: 12409889 PMID: 12385592 PMID: 12380718 PMID: 12224783 PMID: 12199263 PMID: 12162864 PMID: 12010345 PMID: 11972141 PMID: 11921109 PMID: 11685243 PMID: 11588635 PMID: 11499649 PMID: 11493225 PMID: 11463772 PMID: 11359079 PMID: 11316833 PMID: 11240547 PMID: 11154095 PMID: 11152059 PMID: 11133172 PMID: 11058906 PMID: 10981248 PMID: 10971882 PMID: 10916719 PMID: 10923424 PMID: 10818491 PMID: 10771134 PMID: 10634237 PMID: 10385681 PMID: 10363142 PMID: 10099036 PMID: 9823747 PMID: 9814613 PMID: 9778597 PMID: 9745932 PMID: 9673855 PMID: 9666038 PMID: 9556982 PMID: 9513031 PMID: 9499598 PMID: 9425458 PMID: 9403584 PMID: 9347383 PMID: 9180670 PMID: 9138399 PMID: 8895995 PMID: 8695142 PMID: 8956068 PMID: 8848190 PMID: 8984127 PMID: 8543951 PMID: 7575219 PMID: 7494742 PMID: 7481414 PMID: 7706627 PMID: 7729088 PMID: 8051291 PMID: 7858960 PMID: 8192560 PMID: 8207874 PMID: 8030397 PMID: 8143425 PMID: 8190206 PMID: 8139589 PMID: 8295352 PMID: 8252079 PMID: 8170564 PMID: 8458995 PMID: 8440286 PMID: 8272177 PMID: 8160496 PMID: 1595102 PMID: 1676832 PMID: 1745583 PMID: 2582681 PMID: 2803843 PMID: 2779595 PMID: 3147824 PMID: 3614653 PMID: 3504266 PMID: 4058753 PMID: 6509009 PMID: 6151826 PMID: 6086842 PMID: 6743415 PMID: 6736976 PMID: 6124895 PMID: 7093827 PMID: 7437167 PMID: 7415721 PMID: 75248 PMID: 354939 PMID: 1254882 PMID: 1009713 PMID: 4836359 PMID: 4129257 PMID: 4756175 |
| Pathways in cancer | PMID: 21247755 PMID: 21167003 PMID: 21136477 PMID: 21125352 PMID: 21119733 PMID: 21112119 PMID: 21078774 PMID: 20956731 PMID: 20935521 PMID: 20890053 PMID: 20705669 PMID: 20692712 PMID: 20570489 PMID: 20567276 PMID: 20498617 PMID: 20498248 PMID: 20490765 PMID: 20457494 PMID: 20331603 PMID: 20188216 PMID: 20143001 PMID: 20103667 PMID: 20061206 PMID: 20050989 PMID: 20038798 PMID: 20007921 PMID: 20001639 PMID: 19923886 PMID: 19835652 PMID: 19819870 PMID: 19748906 PMID: 19726694 PMID: 19674789 PMID: 19574548 PMID: 19573754 PMID: 19546050 PMID: 19537230 PMID: 19483739 PMID: 19448118 PMID: 19445732 PMID: 19444937 PMID: 19421832 PMID: 19402058 PMID: 19377787 PMID: 19349511 PMID: 19308045 PMID: 19289642 PMID: 19289568 PMID: 19249331 PMID: 19226283 PMID: 19001524 PMID: 18991571 PMID: 18984063 PMID: 18838439 PMID: 18795291 PMID: 18762460 PMID: 18710609 PMID: 18691010 PMID: 18669461 PMID: 18537694 PMID: 18395683 PMID: 18425050 PMID: 18401209 PMID: 18379357 PMID: 18355879 PMID: 18347460 PMID: 18320579 PMID: 18303084 PMID: 18238817 PMID: 18188524 PMID: 18177501 PMID: 18055759 PMID: 18020927 PMID: 17990351 PMID: 17896998 PMID: 17846007 PMID: 17668425 PMID: 17655513 PMID: 17638514 PMID: 17582782 PMID: 17522716 PMID: 17496203 PMID: 17494630 PMID: 17467022 PMID: 17449411 PMID: 17429048 PMID: 17376054 PMID: 17305584 PMID: 17301720 PMID: 17286784 PMID: 17239287 PMID: 17217202 PMID: 17209299 PMID: 17172692 PMID: 17102114 PMID: 17064664 PMID: 16978905 PMID: 16888676 PMID: 16878254 PMID: 16874666 PMID: 16787199 PMID: 16685380 PMID: 16650752 PMID: 16648603 PMID: 16641887 PMID: 16554418 PMID: 16489081 PMID: 16460269 PMID: 16458203 PMID: 16391721 PMID: 16278524 PMID: 16199155 PMID: 16194042 PMID: 16141358 PMID: 16101380 PMID: 16087770 PMID: 15942449 PMID: 15934875 PMID: 15894165 PMID: 15878795 PMID: 15827348 PMID: 15781952 PMID: 15761536 PMID: 15733806 PMID: 15694686 PMID: 15605302 PMID: 15545507 PMID: 15479482 PMID: 15369681 PMID: 15340658 PMID: 19780248 PMID: 15327234 PMID: 15277267 PMID: 15232377 PMID: 15188402 PMID: 15089098 PMID: 15064713 PMID: 15032733 PMID: 14976858 PMID: 14645134 PMID: 14587290 PMID: 14522471 PMID: 14517614 PMID: 12802339 PMID: 12795413 PMID: 12700162 PMID: 12661057 PMID: 12496664 PMID: 12438170 PMID: 12389065 PMID: 12389024 PMID: 12381541 PMID: 12215428 PMID: 12181726 PMID: 12040180 PMID: 11981259 PMID: 11847191 PMID: 11710150 PMID: 11309459 PMID: 11153962 PMID: 11148408 PMID: 11143264 PMID: 11134110 PMID: 11008001 PMID: 10500423 PMID: 10465663 PMID: 10388118 PMID: 9773050 PMID: 9488208 PMID: 9225390 PMID: 9274697 PMID: 9130924 PMID: 8676754 PMID: 7843740 PMID: 7976599 PMID: 8403686 PMID: 8483816 PMID: 1914201 PMID: 2017287 PMID: 2326463 PMID: 3805884 PMID: 3741083 PMID: 6738895 PMID: 6728104 PMID: 6693915 PMID: 6604425 PMID: 6308773 PMID: 6625420 PMID: 7164690 PMID: 7139966 PMID: 7139973 PMID: 7286345 PMID: 7339773 PMID: 546940 PMID: 207843 PMID: 932797 PMID: 1033655 PMID: 4544532 |
| Pentose and glucuronate interconversions |  |
| Pentose phosphate pathway | PMID: 20711518 PMID: 18729006 PMID: 15998684 |
| Phenylalanine metabolism | PMID: 1659482 |
| Phosphatidylinositol signaling system |  |
| PPAR signaling pathway |  |
| Prion diseases | PMID: 18431471 PMID: 15533033 PMID: 11523425 PMID: 7795890 PMID: 8175163 PMID: 1736158 |
| Progesterone-mediated oocyte maturation |  |
| Prostate cancer | PMID: 21136477 PMID: 21094992 PMID: 21054791 PMID: 21053206 PMID: 20967300 PMID: 20881157 PMID: 20881153 PMID: 20880183 PMID: 20843883 PMID: 20806183 PMID: 20727557 PMID: 20722777 PMID: 20702155 PMID: 20665492 PMID: 20643440 PMID: 20630084 PMID: 20605342 PMID: 20598811 PMID: 20568407 PMID: 20518947 PMID: 20487583 PMID: 20465382 PMID: 20393104 PMID: 20229524 PMID: 20159824 PMID: 20159814 PMID: 20141064 PMID: 20087861 PMID: 20086117 PMID: 20085504 PMID: 20061271 PMID: 19963257 PMID: 19949849 PMID: 19900389 PMID: 19875340 PMID: 19816779 PMID: 19768259 PMID: 19755655 PMID: 19706860 PMID: 19628521 PMID: 19591135 PMID: 19513949 PMID: 19453871 PMID: 19444937 PMID: 19409038 PMID: 19357973 PMID: 19346819 PMID: 19342698 PMID: 19332205 PMID: 19303721 PMID: 19293491 PMID: 19284665 PMID: 19283254 PMID: 19233413 PMID: 19228742 PMID: 19143908 PMID: 19134207 PMID: 19097660 PMID: 19047297 PMID: 18947517 PMID: 18849542 PMID: 18813529 PMID: 18786559 PMID: 18719369 PMID: 18645193 PMID: 18607842 PMID: 18454794 PMID: 18411200 PMID: 18265484 PMID: 18248681 PMID: 18243498 PMID: 18172272 PMID: 20717483 PMID: 19347812 PMID: 18162775 PMID: 18158027 PMID: 18091052 PMID: 18050813 PMID: 18042326 PMID: 17974099 PMID: 17950470 PMID: 17870114 PMID: 17761128 PMID: 17651539 PMID: 17619718 PMID: 17596576 PMID: 17572966 PMID: 17443863 PMID: 17334977 PMID: 17311345 PMID: 17267325 PMID: 17265528 PMID: 17224072 PMID: 17189954 PMID: 17188084 PMID: 17164368 PMID: 17153888 PMID: 17051983 PMID: 17011475 PMID: 16952929 PMID: 16923261 PMID: 16921050 PMID: 16902521 PMID: 16752375 PMID: 16643633 PMID: 16601383 PMID: 16551206 PMID: 16546242 PMID: 16527580 PMID: 16520652 PMID: 16469020 PMID: 16448441 PMID: 16426683 PMID: 16365764 PMID: 16360430 PMID: 16356795 PMID: 16309229 PMID: 16296198 PMID: 16291033 PMID: 16288076 PMID: 16250187 PMID: 16243513 PMID: 16217993 PMID: 16213153 PMID: 16208599 PMID: 16158610 PMID: 16142693 PMID: 16101380 PMID: 16053667 PMID: 16043303 PMID: 16020484 PMID: 15925651 PMID: 15890585 PMID: 15889121 PMID: 15878795 PMID: 15794796 PMID: 15768829 PMID: 15711258 PMID: 15667872 PMID: 15636671 PMID: 15629591 PMID: 15351880 PMID: 15336602 PMID: 15184609 PMID: 15169797 PMID: 15167319 PMID: 15146084 PMID: 15126819 PMID: 15083641 PMID: 15032627 PMID: 14697424 PMID: 14673952 PMID: 14505996 PMID: 12869611 PMID: 12855072 PMID: 12789612 PMID: 12750762 PMID: 12746243 PMID: 12745042 PMID: 12738320 PMID: 12667885 PMID: 12640358 PMID: 12639654 PMID: 12602083 PMID: 12497091 PMID: 12487736 PMID: 12428190 PMID: 12393912 PMID: 12211423 PMID: 12208568 PMID: 12199681 PMID: 12010706 PMID: 11937435 PMID: 11809715 PMID: 11741129 PMID: 11709272 PMID: 11701443 PMID: 11520654 PMID: 11436202 PMID: 11205491 PMID: 11139004 PMID: 11020562 PMID: 11018402 PMID: 10878703 PMID: 10854956 PMID: 10812863 PMID: 10784636 PMID: 10750600 PMID: 10696282 PMID: 10682984 PMID: 10654020 PMID: 10616691 PMID: 10580775 PMID: 10430425 PMID: 10234343 PMID: 9951446 PMID: 9820831 PMID: 9644788 PMID: 9583357 PMID: 9571393 PMID: 9112487 PMID: 9092285 PMID: 9086344 PMID: 8932356 PMID: 8693656 PMID: 8627850 PMID: 8704029 PMID: 8608045 PMID: 8829580 PMID: 7583080 PMID: 7789356 PMID: 8009696 PMID: 8222655 PMID: 7686590 PMID: 8439695 PMID: 12345029 PMID: 1621259 PMID: 2626991 PMID: 3285485 PMID: 6475925 PMID: 6694291 |
| Proteasome | PMID: 20577843 PMID: 19597039 PMID: 19526842 PMID: 19458122 PMID: 19399470 PMID: 19237536 PMID: 19193720 PMID: 19165168 PMID: 19074476 PMID: 19264209 PMID: 19007435 PMID: 18464932 PMID: 18317593 PMID: 18259030 PMID: 18172057 PMID: 18157711 PMID: 18086945 PMID: 17971205 PMID: 17700069 PMID: 17681944 PMID: 17668425 PMID: 17646573 PMID: 17381423 PMID: 17314322 PMID: 17220356 PMID: 17142836 PMID: 16839860 PMID: 16782881 PMID: 16769238 PMID: 16338225 PMID: 16306379 PMID: 15716331 PMID: 15660576 PMID: 15638441 PMID: 15337735 PMID: 15320849 PMID: 15213858 PMID: 15208719 PMID: 15169797 PMID: 14625867 PMID: 14616239 PMID: 14499340 PMID: 12959638 PMID: 12437578 PMID: 11880311 PMID: 11509447 PMID: 11509443 PMID: 11380524 PMID: 11278712 PMID: 11001820 PMID: 10484544 PMID: 9812920 PMID: 9698041 PMID: 9683028 |
| Purine metabolism | PMID: 20706956 PMID: 20516647 PMID: 20484295 PMID: 20460346 PMID: 19513390 PMID: 19179314 PMID: 18585721 PMID: 18344415 PMID: 18326285 PMID: 16300459 PMID: 15991921 PMID: 15663643 PMID: 15250282 PMID: 15202488 PMID: 14694147 PMID: 12709672 PMID: 12494033 PMID: 12494026 PMID: 11899846 PMID: 11223861 PMID: 10622277 PMID: 10420458 PMID: 10212666 PMID: 9551574 PMID: 9098853 PMID: 9069456 PMID: 8779683 PMID: 8009701 PMID: 7940361 PMID: 8427538 PMID: 1595201 PMID: 3628142 PMID: 855751 PMID: 1233638 |
| Pyrimidine metabolism | PMID: 17065066 PMID: 12629751 PMID: 12629748 PMID: 11302742 PMID: 9598057 PMID: 7660979 PMID: 3339736 PMID: 6452240 PMID: 5474198 PMID: 13462992 |
| Pyruvate metabolism |  |
| Regulation of actin cytoskeleton | PMID: 21242007 PMID: 20948539 PMID: 20803696 PMID: 20038798 PMID: 19893316 PMID: 19783933 PMID: 19188659 PMID: 18829614 PMID: 18550613 PMID: 18540886 PMID: 17641403 PMID: 17244945 PMID: 17223073 PMID: 17215322 PMID: 17009727 PMID: 16940215 PMID: 16856934 PMID: 16707113 PMID: 16611050 PMID: 16428270 PMID: 16339838 PMID: 15982657 PMID: 15911746 PMID: 19804115 PMID: 15618548 PMID: 15068974 PMID: 14506264 PMID: 12963706 PMID: 12958025 PMID: 12791705 PMID: 12719447 PMID: 12354696 PMID: 12093770 PMID: 11752055 PMID: 10786995 PMID: 10700177 PMID: 10444512 PMID: 9473406 PMID: 8743491 PMID: 2145089 PMID: 6530043 |
| Renal cell carcinoma | PMID: 21223815 PMID: 21208033 PMID: 21195923 PMID: 21125981 PMID: 21119733 PMID: 21090521 PMID: 21078774 PMID: 21051905 PMID: 20956731 PMID: 20952033 PMID: 20944808 PMID: 20806321 PMID: 20800033 PMID: 20799147 PMID: 20733337 PMID: 20733093 PMID: 20733091 PMID: 20709657 PMID: 20698150 PMID: 20693825 PMID: 20679973 PMID: 20624928 PMID: 20603433 PMID: 20577603 PMID: 20571752 PMID: 20556510 PMID: 20458483 PMID: 20450499 PMID: 20448658 PMID: 20407031 PMID: 20401474 PMID: 20395930 PMID: 20390283 PMID: 20383873 PMID: 20368558 PMID: 20347628 PMID: 20225993 PMID: 20181452 PMID: 20173406 PMID: 20163987 PMID: 20163917 PMID: 20100962 PMID: 20082451 PMID: 20072832 PMID: 20061402 PMID: 20047954 PMID: 20039869 PMID: 20035985 PMID: 20028613 PMID: 20019017 PMID: 20012255 PMID: 20006922 PMID: 19920817 PMID: 19914102 PMID: 19914097 PMID: 19897852 PMID: 19875754 PMID: 19858082 PMID: 19815480 PMID: 19745694 PMID: 19736679 PMID: 19734629 PMID: 19671237 PMID: 19670913 PMID: 19652072 PMID: 19652060 PMID: 19640634 PMID: 19640391 PMID: 19629420 PMID: 19377420 PMID: 19620943 PMID: 19616230 PMID: 19596662 PMID: 19588863 PMID: 19583732 PMID: 19567357 PMID: 19549692 PMID: 19542988 PMID: 19542731 PMID: 19528803 PMID: 19513859 PMID: 19499966 PMID: 19496716 PMID: 19487381 PMID: 19474115 PMID: 19473641 PMID: 19462735 PMID: 19456244 PMID: 19403939 PMID: 19402058 PMID: 19381758 PMID: 19372677 PMID: 19332708 PMID: 19261963 PMID: 19261099 PMID: 19255117 PMID: 19224847 PMID: 19224836 PMID: 19220252 PMID: 19213666 PMID: 19211503 PMID: 19208735 PMID: 19203552 PMID: 19184271 PMID: 19177319 PMID: 19157688 PMID: 19147300 PMID: 19136449 PMID: 20689612 PMID: 19100388 PMID: 19098017 PMID: 19070809 PMID: 19037839 PMID: 19009036 PMID: 19002179 PMID: 18992612 PMID: 18985529 PMID: 18936475 PMID: 18838713 PMID: 18838439 PMID: 18836333 PMID: 18824436 PMID: 21086340 PMID: 18795291 PMID: 18786276 PMID: 18752081 PMID: 18751330 PMID: 18716419 PMID: 18711149 PMID: 18701146 PMID: 18690958 PMID: 18669461 PMID: 18664608 PMID: 18630628 PMID: 18520293 PMID: 18542784 PMID: 18509646 PMID: 18501078 PMID: 18468990 PMID: 18462356 PMID: 18419787 PMID: 18418013 PMID: 18407401 PMID: 18386829 PMID: 18366320 PMID: 18360643 PMID: 18313224 PMID: 18303730 PMID: 18257434 PMID: 18224688 PMID: 18221915 PMID: 18192256 PMID: 18190645 PMID: 18172185 PMID: 18083403 PMID: 18072582 PMID: 18048375 PMID: 18035517 PMID: 17992578 PMID: 17962173 PMID: 17959415 PMID: 17922902 PMID: 17631602 PMID: 17876014 PMID: 17868731 PMID: 17846009 PMID: 17825982 PMID: 17825686 PMID: 17786460 PMID: 17760755 PMID: 17657389 PMID: 17656615 PMID: 17655513 PMID: 17586755 PMID: 17586751 PMID: 17564106 PMID: 17557672 PMID: 17513325 PMID: 17487125 PMID: 17425203 PMID: 17406048 PMID: 17382145 PMID: 17374619 PMID: 17335301 PMID: 17332278 PMID: 17320693 PMID: 17303902 PMID: 17267332 PMID: 17255295 PMID: 17227905 PMID: 17225738 PMID: 17215530 PMID: 17212999 PMID: 17189398 PMID: 17164368 PMID: 17158772 PMID: 17146608 PMID: 17085101 PMID: 17045081 PMID: 16928804 PMID: 16910586 PMID: 16883080 PMID: 16807766 PMID: 16792769 PMID: 16705506 PMID: 16704739 PMID: 16687987 PMID: 16582132 PMID: 16548731 PMID: 16531988 PMID: 16503817 PMID: 16458203 PMID: 16364997 PMID: 16358925 PMID: 16314617 PMID: 16208141 PMID: 16201987 PMID: 16197791 PMID: 16189698 PMID: 16184476 PMID: 16171008 PMID: 16142569 PMID: 15999675 PMID: 15977598 PMID: 15945510 PMID: 15929109 PMID: 15918325 PMID: 15860967 PMID: 15812478 PMID: 15783117 PMID: 15763800 PMID: 15758708 PMID: 15673329 PMID: 15616634 PMID: 15562089 PMID: 15553610 PMID: 15477716 PMID: 15448036 PMID: 15448032 PMID: 15379932 PMID: 15349554 PMID: 15315568 PMID: 15293735 PMID: 15293584 PMID: 15283642 PMID: 15253816 PMID: 15212411 PMID: 15123404 PMID: 15042897 PMID: 15006056 PMID: 14990812 PMID: 14764117 PMID: 14696127 PMID: 14669275 PMID: 14665858 PMID: 14621200 PMID: 12890841 PMID: 12883184 PMID: 12778010 PMID: 12684190 PMID: 12683884 PMID: 12673380 PMID: 12655523 PMID: 12611132 PMID: 12507088 PMID: 12469060 PMID: 12385076 PMID: 12162879 PMID: 12081237 PMID: 12050492 PMID: 12031359 PMID: 12020111 PMID: 11959059 PMID: 11902306 PMID: 11847024 PMID: 11769880 PMID: 11769879 PMID: 19570254 PMID: 11752942 PMID: 11692436 PMID: 11688380 PMID: 11593967 PMID: 11579616 PMID: 11535554 PMID: 11505815 PMID: 11483638 PMID: 11458078 PMID: 11417754 PMID: 11332328 PMID: 11323315 PMID: 11256006 PMID: 11231367 PMID: 11221620 PMID: 10752223 PMID: 11144893 PMID: 11096143 PMID: 11063137 PMID: 11061907 PMID: 11058675 PMID: 11018393 PMID: 10999647 PMID: 10886077 PMID: 10897941 PMID: 10841199 PMID: 10768591 PMID: 10667044 PMID: 10619596 PMID: 10610416 PMID: 10605429 PMID: 10582436 PMID: 10503848 PMID: 10493838 PMID: 10473808 PMID: 10459788 PMID: 10449203 PMID: 10435866 PMID: 10417977 PMID: 10328597 PMID: 10327808 PMID: 10190526 PMID: 10189046 PMID: 10084241 PMID: 9737554 PMID: 9730699 PMID: 9719500 PMID: 9719172 PMID: 9681734 PMID: 9652770 PMID: 9650554 PMID: 9619363 PMID: 9589878 PMID: 9562582 PMID: 9392335 PMID: 9707992 PMID: 9218036 PMID: 9273066 PMID: 9312811 PMID: 9112487 PMID: 9098177 PMID: 9238616 PMID: 9041209 PMID: 9116091 PMID: 9075613 PMID: 9158203 PMID: 8973940 PMID: 8914392 PMID: 8881939 PMID: 8647639 PMID: 8616354 PMID: 7490136 PMID: 7591207 PMID: 7637246 PMID: 7655326 PMID: 7736400 PMID: 7829237 PMID: 8560896 PMID: 8559448 PMID: 7966745 PMID: 7817478 PMID: 7733689 PMID: 7977290 PMID: 7885967 PMID: 7868875 PMID: 7860172 PMID: 8080943 PMID: 7518188 PMID: 8280833 PMID: 8212435 PMID: 8345589 PMID: 8503741 PMID: 8446228 PMID: 1423283 PMID: 1420021 PMID: 1393433 PMID: 1638381 PMID: 1617119 PMID: 1617117 PMID: 1632325 PMID: 1581798 PMID: 1561964 PMID: 1553788 PMID: 1782854 PMID: 1997690 PMID: 1996567 PMID: 1927756 PMID: 2084009 PMID: 2254761 PMID: 2220787 PMID: 2380139 PMID: 2205501 PMID: 2659870 PMID: 2924376 PMID: 2812171 PMID: 2803041 PMID: 3063103 PMID: 3229930 PMID: 3066275 PMID: 3431076 PMID: 2444108 PMID: 3306861 PMID: 3573771 PMID: 3543776 PMID: 3661314 PMID: 3806480 PMID: 3536712 PMID: 3461197 PMID: 3959239 PMID: 3484412 PMID: 3084099 PMID: 3904123 PMID: 4044189 PMID: 3964874 PMID: 6542970 PMID: 6392777 PMID: 6463687 PMID: 6351990 PMID: 6827660 PMID: 7406357 PMID: 7430686 PMID: 6244734 PMID: 494466 PMID: 291810 PMID: 442378 PMID: 279841 PMID: 660755 PMID: 650776 PMID: 597363 PMID: 894817 PMID: 850316 PMID: 833981 PMID: 1205694 PMID: 125158 PMID: 1152163 PMID: 4817839 PMID: 14182444 |
| Renin-angiotensin system | PMID: 21249217 PMID: 21248783 PMID: 21245763 PMID: 21245762 PMID: 21242463 PMID: 21242461 PMID: 21239311 PMID: 21239267 PMID: 21239248 PMID: 21240870 PMID: 21234720 PMID: 21234717 PMID: 21228785 PMID: 21226275 PMID: 21226272 PMID: 21224025 PMID: 21215698 PMID: 21213120 PMID: 21208837 PMID: 21206009 PMID: 21198274 PMID: 21196334 PMID: 21195935 PMID: 21191002 PMID: 21189402 PMID: 21189401 PMID: 21188841 PMID: 21188840 PMID: 21188837 PMID: 21188835 PMID: 21188834 PMID: 21188833 PMID: 21188830 PMID: 21188829 PMID: 21188828 PMID: 21188827 PMID: 21188826 PMID: 21188825 PMID: 21188824 PMID: 21188821 PMID: 21188820 PMID: 21188819 PMID: 21188818 PMID: 21188817 PMID: 21188816 PMID: 21188815 PMID: 21188814 PMID: 21188813 PMID: 21188799 PMID: 21182294 PMID: 21180307 PMID: 21178362 PMID: 21178125 PMID: 21167778 PMID: 21164491 PMID: 21148352 PMID: 21148349 PMID: 21143170 PMID: 21143168 PMID: 21134030 PMID: 21132641 PMID: 21130977 PMID: 21130911 PMID: 21125352 PMID: 21125351 PMID: 21124341 PMID: 21116322 PMID: 21115616 PMID: 21115160 PMID: 21111930 PMID: 21108554 PMID: 21099686 PMID: 21098313 PMID: 21093722 PMID: 21091810 PMID: 21090936 PMID: 21089428 PMID: 21088669 PMID: 21084406 PMID: 21076147 PMID: 21071956 PMID: 21071955 PMID: 21071232 PMID: 21068087 PMID: 21066891 PMID: 21063203 PMID: 21059993 PMID: 21059964 PMID: 21056585 PMID: 21050477 PMID: 21049548 PMID: 21045790 PMID: 21045683 PMID: 21033494 PMID: 21029339 PMID: 20980550 PMID: 20978506 PMID: 20978179 PMID: 20975035 PMID: 20974581 PMID: 20968204 PMID: 20966920 PMID: 20959043 PMID: 20957132 PMID: 20956208 PMID: 20954461 PMID: 20953568 PMID: 20950382 PMID: 20948563 PMID: 20948562 PMID: 20944545 PMID: 20939795 PMID: 20937967 PMID: 20937450 PMID: 20937029 PMID: 20935013 PMID: 20933203 PMID: 20924335 PMID: 20923243 PMID: 20922678 PMID: 20921433 PMID: 20921428 PMID: 20920303 PMID: 20890053 PMID: 20880190 PMID: 20876366 PMID: 20864945 PMID: 20861827 PMID: 20860902 PMID: 20859542 PMID: 20859541 PMID: 20857707 PMID: 20855654 PMID: 20850300 PMID: 20829617 PMID: 20847569 PMID: 20837888 PMID: 20837886 PMID: 20837666 PMID: 20833959 PMID: 20833955 PMID: 20827517 PMID: 20821077 PMID: 20819950 PMID: 20817560 PMID: 20816596 PMID: 20816114 PMID: 20811292 PMID: 20809234 PMID: 20807467 PMID: 20798958 PMID: 20795939 PMID: 20735790 PMID: 20733571 PMID: 20733093 PMID: 20730071 PMID: 20728424 PMID: 20724490 PMID: 20723639 PMID: 20714908 PMID: 20707915 PMID: 20706197 PMID: 20705534 PMID: 20697985 PMID: 20696984 PMID: 20690889 PMID: 20689424 PMID: 20689271 PMID: 20689062 PMID: 20687997 PMID: 20685681 PMID: 20679729 PMID: 20679182 PMID: 20678593 PMID: 20675960 PMID: 20675957 PMID: 20668101 PMID: 20662730 PMID: 20653151 PMID: 20651156 PMID: 20646125 PMID: 20640944 PMID: 20634721 PMID: 20609697 PMID: 20626343 PMID: 20625317 PMID: 20625249 PMID: 20625082 PMID: 20625079 PMID: 20620720 PMID: 20615910 PMID: 20586896 PMID: 20614101 PMID: 20613937 PMID: 20606474 PMID: 20606106 PMID: 20605845 PMID: 20602809 PMID: 20602018 PMID: 20597335 PMID: 20597208 PMID: 20595797 PMID: 20590619 PMID: 20583976 PMID: 20582734 PMID: 20581787 PMID: 20581171 PMID: 20580725 PMID: 20574248 PMID: 20570385 PMID: 20567593 PMID: 20567239 PMID: 20558909 PMID: 20556668 PMID: 20554900 PMID: 20543085 PMID: 20542468 PMID: 20539228 PMID: 20538833 PMID: 20537141 PMID: 20532698 PMID: 20531950 PMID: 20531215 PMID: 20531060 PMID: 20530293 PMID: 20528639 PMID: 20528638 PMID: 20528630 PMID: 20524801 PMID: 20525211 PMID: 20524103 PMID: 20524096 PMID: 20519561 PMID: 20518950 PMID: 20516556 PMID: 20516397 PMID: 20516242 PMID: 20512446 PMID: 20511409 PMID: 20505677 PMID: 20503888 PMID: 20498247 PMID: 20494920 PMID: 20493224 PMID: 20491649 PMID: 20488824 PMID: 20488299 PMID: 20488190 PMID: 20487340 PMID: 20479579 PMID: 20467589 PMID: 20465998 PMID: 20463431 PMID: 20458827 PMID: 20455790 PMID: 20455049 PMID: 20448797 PMID: 20448074 PMID: 20447546 PMID: 20447545 PMID: 20444953 PMID: 20444553 PMID: 20443718 PMID: 20440453 PMID: 20440277 PMID: 20439821 PMID: 20439092 PMID: 20438446 PMID: 20430947 PMID: 20429803 PMID: 20429690 PMID: 20425064 PMID: 20424953 PMID: 20424943 PMID: 20424942 PMID: 20424397 PMID: 20422227 PMID: 20421514 PMID: 20420907 PMID: 20418269 PMID: 20415231 PMID: 20415227 PMID: 20415226 PMID: 20415212 PMID: 20413995 PMID: 20409690 PMID: 20408257 PMID: 20407632 PMID: 20399170 PMID: 20396757 PMID: 20392165 PMID: 20391294 PMID: 20384386 PMID: 20381510 PMID: 20380486 PMID: 20379190 PMID: 20375118 PMID: 20374548 PMID: 20370487 PMID: 20368751 PMID: 20368502 PMID: 20367559 PMID: 20363893 PMID: 20019475 PMID: 20019473 PMID: 20019472 PMID: 20019470 PMID: 20019467 PMID: 20360313 PMID: 20360012 PMID: 20359530 PMID: 20356704 PMID: 20351344 PMID: 20347950 PMID: 20346375 PMID: 20339374 PMID: 20329582 PMID: 20329413 PMID: 20302183 PMID: 20299339 PMID: 20238303 PMID: 20237455 PMID: 20236752 PMID: 20236614 PMID: 20231521 PMID: 20231519 PMID: 20229032 PMID: 20224837 PMID: 20224432 PMID: 20223792 PMID: 20220527 PMID: 20219873 PMID: 20219618 PMID: 20216091 PMID: 20216089 PMID: 20213806 PMID: 20212262 PMID: 20211330 PMID: 20210772 PMID: 20204775 PMID: 20203685 PMID: 20203626 PMID: 20203453 PMID: 20198391 PMID: 20194307 PMID: 20189597 PMID: 20182454 PMID: 20178811 PMID: 20170949 PMID: 20169056 PMID: 20166535 PMID: 20164205 PMID: 20164204 PMID: 20160435 PMID: 20160196 PMID: 20157876 PMID: 20156154 PMID: 20153824 PMID: 20150448 PMID: 20150004 PMID: 20149750 PMID: 20144071 PMID: 20141351 PMID: 20135646 PMID: 20134375 PMID: 20134323 PMID: 20133311 PMID: 20129455 PMID: 20129454 PMID: 20128937 PMID: 20102971 PMID: 20126350 PMID: 20123095 PMID: 20122388 PMID: 20118482 PMID: 20118410 PMID: 20113315 PMID: 20111009 PMID: 20110337 PMID: 20104936 PMID: 20104654 PMID: 20104189 PMID: 20103817 PMID: 20102922 PMID: 20102385 PMID: 20102285 PMID: 20099993 PMID: 20095078 PMID: 20093814 PMID: 19724161 PMID: 20091762 PMID: 20090429 PMID: 20088829 PMID: 20087247 PMID: 20083826 PMID: 20083810 PMID: 20083722 PMID: 20081590 PMID: 20077385 PMID: 20057897 PMID: 20055710 PMID: 20055702 PMID: 20053964 PMID: 20051852 PMID: 20046983 PMID: 20046162 PMID: 20042976 PMID: 20042665 PMID: 20042402 PMID: 20038757 PMID: 20038747 PMID: 20036896 PMID: 20030433 PMID: 20030022 PMID: 20027122 PMID: 20026758 PMID: 20026757 PMID: 20021530 PMID: 20019704 PMID: 20019651 PMID: 20016423 PMID: 20016143 PMID: 20015829 PMID: 20013646 PMID: 20012594 PMID: 20009302 PMID: 20007350 PMID: 19999462 PMID: 19930314 PMID: 19997575 PMID: 19996611 PMID: 19966196 PMID: 19965325 PMID: 19961928 PMID: 19958778 PMID: 19955853 PMID: 19954690 PMID: 19948673 PMID: 19946310 PMID: 19944075 PMID: 19942847 PMID: 19942846 PMID: 19942843 PMID: 19942842 PMID: 19940415 PMID: 19935744 PMID: 19933420 PMID: 19932924 PMID: 19930431 PMID: 19930420 PMID: 19927154 PMID: 19927008 PMID: 19926893 PMID: 19926873 PMID: 19924105 PMID: 19923380 PMID: 19923158 PMID: 19922631 PMID: 19921961 PMID: 19919394 PMID: 19917780 PMID: 19917337 PMID: 19917331 PMID: 19917330 PMID: 19915481 PMID: 19914267 PMID: 19911002 PMID: 19911001 PMID: 19904215 PMID: 19903660 PMID: 19901197 PMID: 19900020 PMID: 19900018 PMID: 19450721 PMID: 19450720 PMID: 19450719 PMID: 19893566 PMID: 19893496 PMID: 19893434 PMID: 19892060 PMID: 19888787 PMID: 19887503 PMID: 19886853 PMID: 19884815 PMID: 19882205 PMID: 19878370 PMID: 19864447 PMID: 19864304 PMID: 19863868 PMID: 19863867 PMID: 19863866 PMID: 19861353 PMID: 19861349 PMID: 19861347 PMID: 19853337 PMID: 19834426 PMID: 19851326 PMID: 19847140 PMID: 19846849 PMID: 19846570 PMID: 19843097 PMID: 19841283 PMID: 19837408 PMID: 19835466 PMID: 19827534 PMID: 19811239 PMID: 19825910 PMID: 19825333 PMID: 19822800 PMID: 19822797 PMID: 19822794 PMID: 19817935 PMID: 19817934 PMID: 19817870 PMID: 19816502 PMID: 19810392 PMID: 19809364 PMID: 19809363 PMID: 19808775 PMID: 19808322 PMID: 19805643 PMID: 19805638 PMID: 19805637 PMID: 19802341 PMID: 19799195 PMID: 19799192 PMID: 19797978 PMID: 19797291 PMID: 19794108 PMID: 19793932 PMID: 19785368 PMID: 19782074 PMID: 19779489 PMID: 19779116 PMID: 19774220 PMID: 19770680 PMID: 19768259 PMID: 19768253 PMID: 19763608 PMID: 19756054 PMID: 19749160 PMID: 19749146 PMID: 19748684 PMID: 19747516 PMID: 19747137 PMID: 19739495 PMID: 19738369 PMID: 19737455 PMID: 19737453 PMID: 19737449 PMID: 19735944 PMID: 19734358 PMID: 19733721 PMID: 19732605 PMID: 19727113 PMID: 19727007 PMID: 19723538 PMID: 19719332 PMID: 19715410 PMID: 19713966 PMID: 19707964 PMID: 19707182 PMID: 19707098 PMID: 19706425 PMID: 19706379 PMID: 19701732 PMID: 19701162 PMID: 19700568 PMID: 19698082 PMID: 19696226 PMID: 19690472 PMID: 19689616 PMID: 19689219 PMID: 19688100 PMID: 19687790 PMID: 19686729 PMID: 19684612 PMID: 19681973 PMID: 19673933 PMID: 19673673 PMID: 19672117 PMID: 19672111 PMID: 19671393 PMID: 19671390 PMID: 19671072 PMID: 19669119 PMID: 19668000 PMID: 19666350 PMID: 19662019 PMID: 19633447 PMID: 19652085 PMID: 19649581 PMID: 19648480 PMID: 19641984 PMID: 19641301 PMID: 19640903 PMID: 19640361 PMID: 19639770 PMID: 19623066 PMID: 19620512 PMID: 19619074 PMID: 19617416 PMID: 19617273 PMID: 19617271 PMID: 19609077 PMID: 19609049 PMID: 19605974 PMID: 19587554 PMID: 19587551 PMID: 19587549 PMID: 19602331 PMID: 19602327 PMID: 19602326 PMID: 19602324 PMID: 19601552 PMID: 19601551 PMID: 19601549 PMID: 19601548 PMID: 19601546 PMID: 19595728 PMID: 19593210 PMID: 19593209 PMID: 19593208 PMID: 19592334 PMID: 19590506 PMID: 19571168 PMID: 19584722 PMID: 19580809 PMID: 19578709 PMID: 19577521 PMID: 19574341 PMID: 19574340 PMID: 19572190 PMID: 19570132 PMID: 19570119 PMID: 19566838 PMID: 19564552 PMID: 19564544 PMID: 19557706 PMID: 19556972 PMID: 19555871 PMID: 19546583 PMID: 19546380 PMID: 19546373 PMID: 19543233 PMID: 19539879 PMID: 19538001 PMID: 19537590 PMID: 19537336 PMID: 19534021 PMID: 19508849 PMID: 19531557 PMID: 19527323 PMID: 19524176 PMID: 19523370 PMID: 19521419 PMID: 19521418 PMID: 19520788 PMID: 19516182 PMID: 19516179 PMID: 19516079 PMID: 19491621 PMID: 19491620 PMID: 19491616 PMID: 19491615 PMID: 19513415 PMID: 19509486 PMID: 19509012 PMID: 19505282 PMID: 19505276 PMID: 19503776 PMID: 19502258 PMID: 19502253 PMID: 19497593 PMID: 19497068 PMID: 19494815 PMID: 19494174 PMID: 19491705 PMID: 19490286 PMID: 19487712 PMID: 19484883 PMID: 19481431 PMID: 19479237 PMID: 19477166 PMID: 19476574 PMID: 19475781 PMID: 19475778 PMID: 19475773 PMID: 19474722 PMID: 19474391 PMID: 19469002 PMID: 19466520 PMID: 19466216 PMID: 19463422 PMID: 19460476 PMID: 19459520 PMID: 19455178 PMID: 19454904 PMID: 19451697 PMID: 19443512 PMID: 19442328 PMID: 19442326 PMID: 19436720 PMID: 19436679 PMID: 19436661 PMID: 19436655 PMID: 19433972 PMID: 19433774 PMID: 19432815 PMID: 19430333 PMID: 19428956 PMID: 19427502 PMID: 19427501 PMID: 19427500 PMID: 19427492 PMID: 19427491 PMID: 19424280 PMID: 19419949 PMID: 19417726 PMID: 19412131 PMID: 19412130 PMID: 19392925 PMID: 19390704 PMID: 19390543 PMID: 19373215 PMID: 19372194 PMID: 19364992 PMID: 19364630 PMID: 19364247 PMID: 19363847 PMID: 19361967 PMID: 19356005 PMID: 19348244 PMID: 19348235 PMID: 19348224 PMID: 19348223 PMID: 19348142 PMID: 19343087 PMID: 19340755 PMID: 19340531 PMID: 19339999 PMID: 19339998 PMID: 19339677 PMID: 19332659 PMID: 19330918 PMID: 19329197 PMID: 19327775 PMID: 19327134 PMID: 19325560 PMID: 19322187 PMID: 19319498 PMID: 19308341 PMID: 19307984 PMID: 19301781 PMID: 19298532 PMID: 19296328 PMID: 19296235 PMID: 19290794 PMID: 19289651 PMID: 19287817 PMID: 19287096 PMID: 19286752 PMID: 19278599 PMID: 19278598 PMID: 19278597 PMID: 19274051 PMID: 19272479 PMID: 19268675 PMID: 19267052 PMID: 19265246 PMID: 19264441 PMID: 19262496 PMID: 19262478 PMID: 19262221 PMID: 19261819 PMID: 19259805 PMID: 19255824 PMID: 19255358 PMID: 19253715 PMID: 19249905 PMID: 19249443 PMID: 19246717 PMID: 19246535 PMID: 19246475 PMID: 19245518 PMID: 19244589 PMID: 19244588 PMID: 19244403 PMID: 19244399 PMID: 19243623 PMID: 19240304 PMID: 19239592 PMID: 19238903 PMID: 19236440 PMID: 19233604 PMID: 19230172 PMID: 19229817 PMID: 19228756 PMID: 19227807 PMID: 19225200 PMID: 19223006 PMID: 19220742 PMID: 19219002 PMID: 19218503 PMID: 19218356 PMID: 19218092 PMID: 19216016 PMID: 19215237 PMID: 19211690 PMID: 19202919 PMID: 19199982 PMID: 19198728 PMID: 18456194 PMID: 19197138 PMID: 19195602 PMID: 19183745 PMID: 19182077 PMID: 19179817 PMID: 19178127 PMID: 19176688 PMID: 19171690 PMID: 19168534 PMID: 19166800 PMID: 19160528 PMID: 19158408 PMID: 19155791 PMID: 19155618 PMID: 19154322 PMID: 19148104 PMID: 19147462 PMID: 19146803 PMID: 19145207 PMID: 19144653 PMID: 19142999 PMID: 19139376 PMID: 19138313 PMID: 19138171 PMID: 19138169 PMID: 19131936 PMID: 19131933 PMID: 19129854 PMID: 19129738 PMID: 19128278 PMID: 19126666 PMID: 19126660 PMID: 19125897 PMID: 19124682 PMID: 19124678 PMID: 19124433 PMID: 19124424 PMID: 19124420 PMID: 19119484 PMID: 20948734 PMID: 20380117 PMID: 20131938 PMID: 20131937 PMID: 19893088 PMID: 19114890 PMID: 19114643 PMID: 19113795 PMID: 19109662 PMID: 19103986 PMID: 19101780 PMID: 19093698 PMID: 19092813 PMID: 19090344 PMID: 19085043 PMID: 19082699 PMID: 19077694 PMID: 19076077 PMID: 19075785 PMID: 19075095 PMID: 19073907 PMID: 19008539 PMID: 19066408 PMID: 19066001 PMID: 19060862 PMID: 19059574 PMID: 19056836 PMID: 19055985 PMID: 19054798 PMID: 19047581 PMID: 19047580 PMID: 19037531 PMID: 19034321 PMID: 19033634 PMID: 19033253 PMID: 19029505 PMID: 19029483 PMID: 19028917 PMID: 19021695 PMID: 19020509 PMID: 19018797 PMID: 19018688 PMID: 19015604 PMID: 19015602 PMID: 19015385 PMID: 19014923 PMID: 19014390 PMID: 19012063 PMID: 19010896 PMID: 19010879 PMID: 19001516 PMID: 18996825 PMID: 18984652 PMID: 18981322 PMID: 18981317 PMID: 18981308 PMID: 18979719 PMID: 18974652 PMID: 18971544 PMID: 18971542 PMID: 18959835 PMID: 18958502 PMID: 18958182 PMID: 18957817 PMID: 18957786 PMID: 18957387 PMID: 18955659 PMID: 18950556 PMID: 18949169 PMID: 18948960 PMID: 18940307 PMID: 18939903 PMID: 18939386 PMID: 18930783 PMID: 18929228 PMID: 18843231 PMID: 18923387 PMID: 18923367 PMID: 18855718 PMID: 18855637 PMID: 18855530 PMID: 18854744 PMID: 18853198 PMID: 18847324 PMID: 18847303 PMID: 18846932 PMID: 18845809 PMID: 18844766 PMID: 18838625 PMID: 18835922 PMID: 18832826 PMID: 18832257 PMID: 18832092 PMID: 18830250 PMID: 18827905 PMID: 18819340 PMID: 18816206 PMID: 18814762 PMID: 18813285 PMID: 18809790 PMID: 18807241 PMID: 18807206 PMID: 18806897 PMID: 18552271 PMID: 18800142 PMID: 18800139 PMID: 18795583 PMID: 18794817 PMID: 18792876 PMID: 18791745 PMID: 18788405 PMID: 18788403 PMID: 18788395 PMID: 18788393 PMID: 18787722 PMID: 18787523 PMID: 18787520 PMID: 18783618 PMID: 18782141 PMID: 18779439 PMID: 18779436 PMID: 18778332 PMID: 18776118 PMID: 18775902 PMID: 18775357 PMID: 18775122 PMID: 18775121 PMID: 18775120 PMID: 18775113 PMID: 18775112 PMID: 18773531 PMID: 18773126 PMID: 18772857 PMID: 18765097 PMID: 18765096 PMID: 18765095 PMID: 18765093 PMID: 18765089 PMID: 18765086 PMID: 18764720 PMID: 18760761 PMID: 18760759 PMID: 18760758 PMID: 18760757 PMID: 18756340 PMID: 18756260 PMID: 18729004 PMID: 18724960 PMID: 18722896 PMID: 18718746 PMID: 18711010 PMID: 18707969 PMID: 18704745 PMID: 18704622 PMID: 18703536 PMID: 18700558 PMID: 18700549 PMID: 18700548 PMID: 18700314 PMID: 18698331 PMID: 18698212 PMID: 18697992 PMID: 18695385 PMID: 18693632 PMID: 18692558 PMID: 18691475 PMID: 18689957 PMID: 18687466 PMID: 18685606 PMID: 18684890 PMID: 18683482 PMID: 18679784 PMID: 18679781 PMID: 18670907 PMID: 18666842 PMID: 18657531 PMID: 18653711 PMID: 18650793 PMID: 18650792 PMID: 18650598 PMID: 18638617 PMID: 18638616 PMID: 18638614 PMID: 18637188 PMID: 18634977 PMID: 18633762 PMID: 18633754 PMID: 18629353 PMID: 18580863 PMID: 18580862 PMID: 18545893 PMID: 18625163 PMID: 18625162 PMID: 18625117 PMID: 18622353 PMID: 18622246 PMID: 18622244 PMID: 18622238 PMID: 18615100 PMID: 18614817 PMID: 18614776 PMID: 18607150 PMID: 18607142 PMID: 18606911 PMID: 18604370 PMID: 18600300 PMID: 18600297 PMID: 18599677 PMID: 18473314 PMID: 18587241 PMID: 20409934 PMID: 18584583 PMID: 18583262 PMID: 18579674 PMID: 18579222 PMID: 18575070 PMID: 18572207 PMID: 18569908 PMID: 18568313 PMID: 18567711 PMID: 18567710 PMID: 18564334 PMID: 18563171 PMID: 18562020 PMID: 18560669 PMID: 18560212 PMID: 18557800 PMID: 18551014 PMID: 18551012 PMID: 18551010 PMID: 18550936 PMID: 18550934 PMID: 18548147 PMID: 18548089 PMID: 18544166 PMID: 18537759 PMID: 18535668 PMID: 18535538 PMID: 18535536 PMID: 18528800 PMID: 18520954 PMID: 18520721 PMID: 18519134 PMID: 18518883 PMID: 18516095 PMID: 18510491 PMID: 18509345 PMID: 18506359 PMID: 18395684 PMID: 18395678 PMID: 18395677 PMID: 18497480 PMID: 18497476 PMID: 18497458 PMID: 18496146 PMID: 18495795 PMID: 18494810 PMID: 18490805 PMID: 18489861 PMID: 18488444 PMID: 18480417 PMID: 18479899 PMID: 18475146 PMID: 18474827 PMID: 18474174 PMID: 18473295 PMID: 18471445 PMID: 18466419 PMID: 18465684 PMID: 18463318 PMID: 18449382 PMID: 18460596 PMID: 18456730 PMID: 18455003 PMID: 18454336 PMID: 18454253 PMID: 18453798 PMID: 18450829 PMID: 18449520 PMID: 18445755 PMID: 19804328 PMID: 18443751 PMID: 18443750 PMID: 18443571 PMID: 18443258 PMID: 18442360 PMID: 18440325 PMID: 18438549 PMID: 18437552 PMID: 18437334 PMID: 18437333 PMID: 18437332 PMID: 18437140 PMID: 18437139 PMID: 18430060 PMID: 18424632 PMID: 18420994 PMID: 18417117 PMID: 18416854 PMID: 18413498 PMID: 18413494 PMID: 18413492 PMID: 18410507 PMID: 18408475 PMID: 18404602 PMID: 18402537 PMID: 18401240 PMID: 18400868 PMID: 18398593 PMID: 18398395 PMID: 18398345 PMID: 18398335 PMID: 18398321 PMID: 18396000 PMID: 18391097 PMID: 18391092 PMID: 18385968 PMID: 18385669 PMID: 18384915 PMID: 18384844 PMID: 18383770 PMID: 18380058 PMID: 18378855 PMID: 18374681 PMID: 18374418 PMID: 18373194 PMID: 18372234 PMID: 18369742 PMID: 18367030 PMID: 18367023 PMID: 18367014 PMID: 18367013 PMID: 18367011 PMID: 18366863 PMID: 18364619 PMID: 18363646 PMID: 18362227 PMID: 18362226 PMID: 18360019 PMID: 18360010 PMID: 18359898 PMID: 18355768 PMID: 18354383 PMID: 18353869 PMID: 18351457 PMID: 18350781 PMID: 18350443 PMID: 18347668 PMID: 18347611 PMID: 18347448 PMID: 18347439 PMID: 18347230 PMID: 18347225 PMID: 18344631 PMID: 18344376 PMID: 18343241 PMID: 18340680 PMID: 18340340 PMID: 18337487 PMID: 18333378 PMID: 18332286 PMID: 18328909 PMID: 18328297 PMID: 18327992 PMID: 18327991 PMID: 18327094 PMID: 18327089 PMID: 18326972 PMID: 18319594 PMID: 18318741 PMID: 18317628 PMID: 18316950 PMID: 18311665 PMID: 18307835 PMID: 18307743 PMID: 18307734 PMID: 18307733 PMID: 18307732 PMID: 19804294 PMID: 18305548 PMID: 18304583 PMID: 18303933 PMID: 18301870 PMID: 18300871 PMID: 18300843 PMID: 18299203 PMID: 18294862 PMID: 18293857 PMID: 18292466 PMID: 18289603 PMID: 18288179 PMID: 18287671 PMID: 18281370 PMID: 18275765 PMID: 18273042 PMID: 18269189 PMID: 18268482 PMID: 18268147 PMID: 18268138 PMID: 18266774 PMID: 18265794 PMID: 18264915 PMID: 18264912 PMID: 18264628 PMID: 18260981 PMID: 18260912 PMID: 18260840 PMID: 18259034 PMID: 18259017 PMID: 18258853 PMID: 18256579 PMID: 18250549 PMID: 18250325 PMID: 18243793 PMID: 18241288 PMID: 18239819 PMID: 18239590 PMID: 18237679 PMID: 18236361 PMID: 18235083 PMID: 18174783 PMID: 18227408 PMID: 18223352 PMID: 18221088 PMID: 18221077 PMID: 18220718 PMID: 18220697 PMID: 18219303 PMID: 18219299 PMID: 18216141 PMID: 18214672 PMID: 18212268 PMID: 18212262 PMID: 18205625 PMID: 18205101 PMID: 18205097 PMID: 18202667 PMID: 18200816 PMID: 18200812 PMID: 18200809 PMID: 18200774 PMID: 18199587 PMID: 18198281 PMID: 18195162 PMID: 18195161 PMID: 18194430 PMID: 18192845 PMID: 18192841 PMID: 18192840 PMID: 18192836 PMID: 18192335 PMID: 18191300 PMID: 18187437 PMID: 18187280 PMID: 18182798 PMID: 18182795 PMID: 18093408 PMID: 18093407 PMID: 18177591 PMID: 18177590 PMID: 18177588 PMID: 18177587 PMID: 18177583 PMID: 18175939 PMID: 18175217 PMID: 18175062 PMID: 18174884 PMID: 18174880 PMID: 18174767 PMID: 18172061 PMID: 18172055 PMID: 20409880 PMID: 19337544 PMID: 19337534 PMID: 19281913 PMID: 18158357 PMID: 18154717 PMID: 18154154 PMID: 18095912 PMID: 18094340 PMID: 18094033 PMID: 18091747 PMID: 18090671 PMID: 18087711 PMID: 18083506 PMID: 18083251 PMID: 18081357 PMID: 18076344 PMID: 18073713 PMID: 18072386 PMID: 18063856 PMID: 18062896 PMID: 18057217 PMID: 18051227 PMID: 18048710 PMID: 18047925 PMID: 18047465 PMID: 18046666 PMID: 18045096 PMID: 18044458 PMID: 18041161 PMID: 18039983 PMID: 18037779 PMID: 18037772 PMID: 18035185 PMID: 18034588 PMID: 18031114 PMID: 17970613 PMID: 18022600 PMID: 17978603 PMID: 17978602 PMID: 17978601 PMID: 17903324 PMID: 18001264 PMID: 18000231 PMID: 17999872 PMID: 17999870 PMID: 17998473 PMID: 17993725 PMID: 17992634 PMID: 17990103 PMID: 17990006 PMID: 17987382 PMID: 17986358 PMID: 17985036 PMID: 17984673 PMID: 17984665 PMID: 17984617 PMID: 17984483 PMID: 17981707 PMID: 17981584 PMID: 17980562 PMID: 17979687 PMID: 17978595 PMID: 17978591 PMID: 17969382 PMID: 17969376 PMID: 17969367 PMID: 19151457 PMID: 17965276 PMID: 17961798 PMID: 17959024 PMID: 17955830 PMID: 17727384 PMID: 17954369 PMID: 17954368 PMID: 17952903 PMID: 17952638 PMID: 17951998 PMID: 17950785 PMID: 17948630 PMID: 17948626 PMID: 17948624 PMID: 17943080 PMID: 17934656 PMID: 17934238 PMID: 17933861 PMID: 17932768 PMID: 17931588 PMID: 17929419 PMID: 17928966 PMID: 17923585 PMID: 17909121 PMID: 17909118 PMID: 17909117 PMID: 17907104 PMID: 17907098 PMID: 17903697 PMID: 17903692 PMID: 17903684 PMID: 19124393 PMID: 17895315 PMID: 17890883 PMID: 17889157 PMID: 17885547 PMID: 17883284 PMID: 17880379 PMID: 17878751 PMID: 17878513 PMID: 17877002 PMID: 17873339 PMID: 17868789 PMID: 17868788 PMID: 17868787 PMID: 17867914 PMID: 17867913 PMID: 17855644 PMID: 17850576 PMID: 17846345 PMID: 17666198 PMID: 17666197 PMID: 17666196 PMID: 17666194 PMID: 17825849 PMID: 17824680 PMID: 17824679 PMID: 17803730 PMID: 17786085 PMID: 17786081 PMID: 17786072 PMID: 17785964 PMID: 17785932 PMID: 17785634 PMID: 17785631 PMID: 17785627 PMID: 17765139 PMID: 17762658 PMID: 19668475 PMID: 17729187 PMID: 17726104 PMID: 17724277 PMID: 17724274 PMID: 17724273 PMID: 17722475 PMID: 17709905 PMID: 17704608 PMID: 17703634 PMID: 17699210 PMID: 17691927 PMID: 17703435 PMID: 17703434 PMID: 17703126 PMID: 17702098 PMID: 17700383 PMID: 17699561 PMID: 17699327 PMID: 17693755 PMID: 17693754 PMID: 17692338 PMID: 17692319 PMID: 17691961 PMID: 17686381 PMID: 17686379 PMID: 17686378 PMID: 17684464 PMID: 17683282 PMID: 17683174 PMID: 17682668 PMID: 17679833 PMID: 17679832 PMID: 17679041 PMID: 17675902 PMID: 17674598 PMID: 17674332 PMID: 17673883 PMID: 17666909 PMID: 17665309 PMID: 17664394 PMID: 17663116 PMID: 17653023 PMID: 17650687 PMID: 17647140 PMID: 17647026 PMID: 17646572 PMID: 17646033 PMID: 17643780 PMID: 17642147 PMID: 17641732 PMID: 17640965 PMID: 17630947 PMID: 17630347 PMID: 17629353 PMID: 17627265 PMID: 17626124 PMID: 17625243 PMID: 17622758 PMID: 17622273 PMID: 17616746 PMID: 17614938 PMID: 17608790 PMID: 17608104 PMID: 17607758 PMID: 17607364 PMID: 17606856 PMID: 17605654 PMID: 17604232 PMID: 17601392 PMID: 19160879 PMID: 17597800 PMID: 17596529 PMID: 17595864 PMID: 17592030 PMID: 17587757 PMID: 17587748 PMID: 17586619 PMID: 17586614 PMID: 17586415 PMID: 17586303 PMID: 17586284 PMID: 17581211 PMID: 17579824 PMID: 17579251 PMID: 17578167 PMID: 17576854 PMID: 17576852 PMID: 17575433 PMID: 17573849 PMID: 17571367 PMID: 17570082 PMID: 17569300 PMID: 17566078 PMID: 17565879 PMID: 17563829 PMID: 17563548 PMID: 17563539 PMID: 17562974 PMID: 17556195 PMID: 17552412 PMID: 17548209 PMID: 17547838 PMID: 17546625 PMID: 17546276 PMID: 17541390 PMID: 17541389 PMID: 17541331 PMID: 17541325 PMID: 17541211 PMID: 17537837 PMID: 17534470 PMID: 17534249 PMID: 17533019 PMID: 17531930 PMID: 17522264 PMID: 17522061 PMID: 17521525 PMID: 17520398 PMID: 17519121 PMID: 17519002 PMID: 17506715 PMID: 17504232 PMID: 17495447 PMID: 17494996 PMID: 17493613 PMID: 17489076 PMID: 17488176 PMID: 17487823 PMID: 17487251 PMID: 17485595 PMID: 17477109 PMID: 17477024 PMID: 17475158 PMID: 17474500 PMID: 17470719 PMID: 17470717 PMID: 17464936 PMID: 17461301 PMID: 17460710 PMID: 17460378 PMID: 17460373 PMID: 17460367 PMID: 17460364 PMID: 17459533 PMID: 17458399 PMID: 17455554 PMID: 17453140 PMID: 17452509 PMID: 17452499 PMID: 17449556 PMID: 17446713 PMID: 17446534 PMID: 17444279 PMID: 17444274 PMID: 17442229 PMID: 17442227 PMID: 17442223 PMID: 17439418 PMID: 17439247 PMID: 17438952 PMID: 17438305 PMID: 17436021 PMID: 17429048 PMID: 17428896 PMID: 17425665 PMID: 17420664 PMID: 17418683 PMID: 17416265 PMID: 17414669 PMID: 17414657 PMID: 17414582 PMID: 17409317 PMID: 17407587 PMID: 17404186 PMID: 17404180 PMID: 17403166 PMID: 17402563 PMID: 17402291 PMID: 17401314 PMID: 17401312 PMID: 17401311 PMID: 17391169 PMID: 17389253 PMID: 17386874 PMID: 17386830 PMID: 17386349 PMID: 17384453 PMID: 17380887 PMID: 17379017 PMID: 17378373 PMID: 17377513 PMID: 17376010 PMID: 17372036 PMID: 17370850 PMID: 17369717 PMID: 17367659 PMID: 17367405 PMID: 17364596 PMID: 17363603 PMID: 17362841 PMID: 17362671 PMID: 17361526 PMID: 17360470 PMID: 17356844 PMID: 17356118 PMID: 17355830 PMID: 17355164 PMID: 17354656 PMID: 17346243 PMID: 17346127 PMID: 17343900 PMID: 17341529 PMID: 17337492 PMID: 17336417 PMID: 17334632 PMID: 17334527 PMID: 17333097 PMID: 17332676 PMID: 17332436 PMID: 17324742 PMID: 17324151 PMID: 17324119 PMID: 17323587 PMID: 17322575 PMID: 17321627 PMID: 17320999 PMID: 17320855 PMID: 17319472 PMID: 17319461 PMID: 17318789 PMID: 17315601 PMID: 17314035 PMID: 17307972 PMID: 17303661 PMID: 17303473 PMID: 17301824 PMID: 17301693 PMID: 17301622 PMID: 17299437 PMID: 17295666 PMID: 17294577 PMID: 17293688 PMID: 17293686 PMID: 17293678 PMID: 17291601 PMID: 17288698 PMID: 17287426 PMID: 17283869 PMID: 17277018 PMID: 17275289 PMID: 17269597 PMID: 17261642 PMID: 17260464 PMID: 17259738 PMID: 17257271 PMID: 17255528 PMID: 17254516 PMID: 17253471 PMID: 17251496 PMID: 17250640 PMID: 17245476 PMID: 17242300 PMID: 17229979 PMID: 17229674 PMID: 17227234 PMID: 17224474 PMID: 17220293 PMID: 17218415 PMID: 17217709 PMID: 17217708 PMID: 17217707 PMID: 17217099 PMID: 17215650 PMID: 17214277 PMID: 17213573 PMID: 17211247 PMID: 17211228 PMID: 17210476 PMID: 17209324 PMID: 17204493 PMID: 17202406 PMID: 17200690 PMID: 17199221 PMID: 17199213 PMID: 17196676 PMID: 17196575 PMID: 17192509 PMID: 17190732 PMID: 17186681 PMID: 17186680 PMID: 17185409 PMID: 17185142 PMID: 17185112 PMID: 17184180 PMID: 17183494 PMID: 17173262 PMID: 17173244 PMID: 17173239 PMID: 17170603 PMID: 17169295 PMID: 17168728 PMID: 17161777 PMID: 17161776 PMID: 17161748 PMID: 17161436 PMID: 17159081 PMID: 17147926 PMID: 17147925 PMID: 17143190 PMID: 17143170 PMID: 17143168 PMID: 17143167 PMID: 17140168 PMID: 17139806 PMID: 17139580 PMID: 17137404 PMID: 17135705 PMID: 17134615 PMID: 17131847 PMID: 17127253 PMID: 17125841 PMID: 17124396 PMID: 17122330 PMID: 17118124 PMID: 17116759 PMID: 17113991 PMID: 17098828 PMID: 17094057 PMID: 17094056 PMID: 17094050 PMID: 17094049 PMID: 17094048 PMID: 17091602 PMID: 17090678 PMID: 17089475 PMID: 17087862 PMID: 17086021 PMID: 17083268 PMID: 17083074 PMID: 17083073 PMID: 17083068 PMID: 17083065 PMID: 17083061 PMID: 17082352 PMID: 17081084 PMID: 17075219 PMID: 17075218 PMID: 17075213 PMID: 17075201 PMID: 17073832 PMID: 17073705 PMID: 17073613 PMID: 17070433 PMID: 17066056 PMID: 17065573 PMID: 17064808 PMID: 17061451 PMID: 17060513 PMID: 17060512 PMID: 17060507 PMID: 17058853 PMID: 17055947 PMID: 17053529 PMID: 17048209 PMID: 17048199 PMID: 17045222 PMID: 17044667 PMID: 17042963 PMID: 17041806 PMID: 17041716 PMID: 17037313 PMID: 17035613 PMID: 17031066 PMID: 17029631 PMID: 17028485 PMID: 17022602 PMID: 17022600 PMID: 17015773 PMID: 17014864 PMID: 17014556 PMID: 17013240 PMID: 17009099 PMID: 17008838 PMID: 17008835 PMID: 17008303 PMID: 17003835 PMID: 17003827 PMID: 17001498 PMID: 17000927 PMID: 16999659 PMID: 16999229 PMID: 16998266 PMID: 16989590 PMID: 16989399 PMID: 16987854 PMID: 16986231 PMID: 16981926 PMID: 16981561 PMID: 16981553 PMID: 16981518 PMID: 16981517 PMID: 16980380 PMID: 16980206 PMID: 16979787 PMID: 16978547 PMID: 16972730 PMID: 16969748 PMID: 16968989 PMID: 16963475 PMID: 16959581 PMID: 16954165 PMID: 16946584 PMID: 16946085 PMID: 19804189 PMID: 16940231 PMID: 16940229 PMID: 16940212 PMID: 16939543 PMID: 16937775 PMID: 16937606 PMID: 16937603 PMID: 16929142 PMID: 16922820 PMID: 16917022 PMID: 16915347 PMID: 16915123 PMID: 16915040 PMID: 16915019 PMID: 16915013 PMID: 16914960 PMID: 16914958 PMID: 16914423 PMID: 16914072 PMID: 16913091 PMID: 16910761 PMID: 16910760 PMID: 16909244 PMID: 16908757 PMID: 16900013 PMID: 16898573 PMID: 16897888 PMID: 16897885 PMID: 16897869 PMID: 16897868 PMID: 16897867 PMID: 16897852 PMID: 16897851 PMID: 16897849 PMID: 16896271 PMID: 16896185 PMID: 16895678 PMID: 16895219 PMID: 16895196 PMID: 16895195 PMID: 16895193 PMID: 16895184 PMID: 16895175 PMID: 16895172 PMID: 16895169 PMID: 16895167 PMID: 16895166 PMID: 16885412 PMID: 16885153 PMID: 16884660 PMID: 16883325 PMID: 16879778 PMID: 16878172 PMID: 16876684 PMID: 16870827 PMID: 16868049 PMID: 16864159 PMID: 16855517 PMID: 16843452 PMID: 16843085 PMID: 16843071 PMID: 16842210 PMID: 16842207 PMID: 16837795 PMID: 16837049 PMID: 16827595 PMID: 16825530 PMID: 16825422 PMID: 16825309 PMID: 16816138 PMID: 16810471 PMID: 16810469 PMID: 16810285 PMID: 16810078 PMID: 16802544 PMID: 16801480 PMID: 16799256 PMID: 16794495 PMID: 16794486 PMID: 16789486 PMID: 16789402 PMID: 16788142 PMID: 16787263 PMID: 16784934 PMID: 16783586 PMID: 16783273 PMID: 16781089 PMID: 16780186 PMID: 16778327 PMID: 16776746 PMID: 16774068 PMID: 16774008 PMID: 16773397 PMID: 16771242 PMID: 16769926 PMID: 16768130 PMID: 16767297 PMID: 16767097 PMID: 16766648 PMID: 16762293 PMID: 16760545 PMID: 16757126 PMID: 16755200 PMID: 16755197 PMID: 16755156 PMID: 16754793 PMID: 16749484 PMID: 16741139 PMID: 16732715 PMID: 16723864 PMID: 16719821 PMID: 16715648 PMID: 16714246 PMID: 16710353 PMID: 16709309 PMID: 16703216 PMID: 16702618 PMID: 16702489 PMID: 16700880 PMID: 16700858 PMID: 16687944 PMID: 16685579 PMID: 16685192 PMID: 16681991 PMID: 16673014 PMID: 16672313 PMID: 16672148 PMID: 16672147 PMID: 16672146 PMID: 16672145 PMID: 16672144 PMID: 16672142 PMID: 16669974 PMID: 16651849 PMID: 16651722 PMID: 16649442 PMID: 16649424 PMID: 16648368 PMID: 16647630 PMID: 19804091 PMID: 16645728 PMID: 16641928 PMID: 16640505 PMID: 16640172 PMID: 16640079 PMID: 16636899 PMID: 16635914 PMID: 16635753 PMID: 16629872 PMID: 16623947 PMID: 16616959 PMID: 16612258 PMID: 16612257 PMID: 16611677 PMID: 16610183 PMID: 16608130 PMID: 16607602 PMID: 16603700 PMID: 16601577 PMID: 16601575 PMID: 16601574 PMID: 16601562 PMID: 16601559 PMID: 16601558 PMID: 16601219 PMID: 16600165 PMID: 16600156 PMID: 16596809 PMID: 16585421 PMID: 16585419 PMID: 16585408 PMID: 16581295 PMID: 16580582 PMID: 16572194 PMID: 16568131 PMID: 16565869 PMID: 16565308 PMID: 16565257 PMID: 16565245 PMID: 16565243 PMID: 16565237 PMID: 16565234 PMID: 16565230 PMID: 16563947 PMID: 16563946 PMID: 16563944 PMID: 16557296 PMID: 16557218 PMID: 16554331 PMID: 16551292 PMID: 16548238 PMID: 16540557 PMID: 16529551 PMID: 16528251 PMID: 16528245 PMID: 16523000 PMID: 16521820 PMID: 16516603 PMID: 16514174 PMID: 16514079 PMID: 16513451 PMID: 16511505 PMID: 16509553 PMID: 16508589 PMID: 16508564 PMID: 16505206 PMID: 16503870 PMID: 16494512 PMID: 16489846 PMID: 16485730 PMID: 16482098 PMID: 16481885 PMID: 16481883 PMID: 16481882 PMID: 16476717 PMID: 16471175 PMID: 16471111 PMID: 16470482 PMID: 16470079 PMID: 16470078 PMID: 16468060 PMID: 16467655 PMID: 16464173 PMID: 16461186 PMID: 16461185 PMID: 16461184 PMID: 16461183 PMID: 16457788 PMID: 16455770 PMID: 16453095 PMID: 16452532 PMID: 16448322 PMID: 16446393 PMID: 16444921 PMID: 16444875 PMID: 16444043 PMID: 16438064 PMID: 16432054 PMID: 16427219 PMID: 16424367 PMID: 16422128 PMID: 16421476 PMID: 16416810 PMID: 16415374 PMID: 16413241 PMID: 16405197 PMID: 16403686 PMID: 16401765 PMID: 16398060 PMID: 16397519 PMID: 16396964 PMID: 16395261 PMID: 16394249 PMID: 16392324 PMID: 16392299 PMID: 16391708 PMID: 16391175 PMID: 16386657 PMID: 19789728 PMID: 16384834 PMID: 16384824 PMID: 16380540 PMID: 16378772 PMID: 16377440 PMID: 16374846 PMID: 16374423 PMID: 16372167 PMID: 16365193 PMID: 16365192 PMID: 16364838 PMID: 16356784 PMID: 16354940 PMID: 16352906 PMID: 16352854 PMID: 16352114 PMID: 16344374 PMID: 16341685 PMID: 16341051 PMID: 16338452 PMID: 16333380 PMID: 16331123 PMID: 16331096 PMID: 16331093 PMID: 16330897 PMID: 16330697 PMID: 16330466 PMID: 16327253 PMID: 16321616 PMID: 16316360 PMID: 16316354 PMID: 19667709 PMID: 16311217 PMID: 16310573 PMID: 16309575 PMID: 16306796 PMID: 16299626 PMID: 16298258 PMID: 16295047 PMID: 16292423 PMID: 16289140 PMID: 16289003 PMID: 16286578 PMID: 16286577 PMID: 16286575 PMID: 16286563 PMID: 16284232 PMID: 16281161 PMID: 16280650 PMID |
| Retinol metabolism | PMID: 18641182 PMID: 11130751 |
| Ribosome | PMID: 20219868 PMID: 19304949 PMID: 18478094 PMID: 17600544 PMID: 16982039 PMID: 15612671 PMID: 12738089 PMID: 12468564 PMID: 11978851 PMID: 10984626 PMID: 9357789 PMID: 8557490 PMID: 7987029 PMID: 3548424 |
| RIG-I-like receptor signaling pathway |  |
| RNA polymerase | PMID: 21061100 PMID: 20503137 PMID: 20175839 PMID: 20012923 PMID: 19918031 PMID: 19799786 PMID: 19696067 PMID: 19592490 PMID: 19487221 PMID: 19267666 PMID: 18286246 PMID: 17604287 PMID: 17601770 PMID: 17179700 PMID: 17117486 PMID: 17039313 PMID: 16798048 PMID: 16084222 PMID: 15577611 PMID: 15158315 PMID: 12718748 PMID: 11787881 PMID: 11465718 PMID: 10555916 PMID: 6177342 PMID: 6157390 |
| Small cell lung cancer | PMID: 21119733 PMID: 21107290 PMID: 21079521 PMID: 20967300 PMID: 20881641 PMID: 20715425 PMID: 20681440 PMID: 20650686 PMID: 20516450 PMID: 20460557 PMID: 20439196 PMID: 20427348 PMID: 20407031 PMID: 20389300 PMID: 20358384 PMID: 20356758 PMID: 20234816 PMID: 20225906 PMID: 20085937 PMID: 20028752 PMID: 19940466 PMID: 19917841 PMID: 19829770 PMID: 19793713 PMID: 19652055 PMID: 19597027 PMID: 19433684 PMID: 19349511 PMID: 19349493 PMID: 19332730 PMID: 19319108 PMID: 19308410 PMID: 20716427 PMID: 19289369 PMID: 19261963 PMID: 19228742 PMID: 19138979 PMID: 19091548 PMID: 19081729 PMID: 18977094 PMID: 18971808 PMID: 18936474 PMID: 18827612 PMID: 18772832 PMID: 18650173 PMID: 18520293 PMID: 18536787 PMID: 18481038 PMID: 18449004 PMID: 18411700 PMID: 18398152 PMID: 18379357 PMID: 18157597 PMID: 18055759 PMID: 17909358 PMID: 17898810 PMID: 17892508 PMID: 17826629 PMID: 17825686 PMID: 17762342 PMID: 17726596 PMID: 17710205 PMID: 17671153 PMID: 17602060 PMID: 17573328 PMID: 17571910 PMID: 17532379 PMID: 17409986 PMID: 17327260 PMID: 17267325 PMID: 17239287 PMID: 17212999 PMID: 17197191 PMID: 17168436 PMID: 17143257 PMID: 16932737 PMID: 16476542 PMID: 15867233 PMID: 15560691 PMID: 15510598 PMID: 15366568 PMID: 15217970 PMID: 15178812 PMID: 12655441 PMID: 12377883 PMID: 11955658 PMID: 11892431 PMID: 10808390 PMID: 10758383 PMID: 10731757 PMID: 10585066 PMID: 10408844 PMID: 9593699 PMID: 9560532 PMID: 9331136 PMID: 9155197 PMID: 9507688 PMID: 8622281 PMID: 7844610 PMID: 8032544 PMID: 8390589 PMID: 1661205 PMID: 2626991 PMID: 2986559 |
| SNARE interactions in vesicular transport |  |
| Sphingolipid metabolism | PMID: 21241794 PMID: 19784582 |
| Spliceosome | PMID: 18820179 PMID: 16723792 PMID: 15563821 |
| Starch and sucrose metabolism | PMID: 19932004 PMID: 18474346 PMID: 17616744 PMID: 17386347 PMID: 17215582 PMID: 16944307 PMID: 16582031 PMID: 15878173 PMID: 14513067 PMID: 11710758 PMID: 9781621 PMID: 9625092 PMID: 9550454 PMID: 9280208 PMID: 9313782 PMID: 8706356 PMID: 8556833 PMID: 7662222 PMID: 7936845 PMID: 8437990 PMID: 1873016 PMID: 2177455 PMID: 3465909 |
| Steroid hormone biosynthesis | PMID: 21193036 PMID: 16426683 PMID: 15134825 |
| Systemic lupus erythematosus | PMID: 21248669 PMID: 21243304 PMID: 21225673 PMID: 21225443 PMID: 21212140 PMID: 21199460 PMID: 21194884 PMID: 21188448 PMID: 21162701 PMID: 21158850 PMID: 21129825 PMID: 21125145 PMID: 21123325 PMID: 21092251 PMID: 21089445 PMID: 20975635 PMID: 20973224 PMID: 20965395 PMID: 20947541 PMID: 20920173 PMID: 20870275 PMID: 20861207 PMID: 20850284 PMID: 20830234 PMID: 20827107 PMID: 20824279 PMID: 20814813 PMID: 20813797 PMID: 20704603 PMID: 20704100 PMID: 20696988 PMID: 20688887 PMID: 20680313 PMID: 20676648 PMID: 20669554 PMID: 20668858 PMID: 20661045 PMID: 20658237 PMID: 20599346 PMID: 20597269 PMID: 20570357 PMID: 20535475 PMID: 20535471 PMID: 20532956 PMID: 20521398 PMID: 20511979 PMID: 20507945 PMID: 20505627 PMID: 20504294 PMID: 20470389 PMID: 20434405 PMID: 20415236 PMID: 20396352 PMID: 20375662 PMID: 20377061 PMID: 20370340 PMID: 20359616 PMID: 20305045 PMID: 20187361 PMID: 20179170 PMID: 20178809 PMID: 20137654 PMID: 20133169 PMID: 20131232 PMID: 20119723 PMID: 20118159 PMID: 20089610 PMID: 20080915 PMID: 20079323 PMID: 20031679 PMID: 20012052 PMID: 19999586 PMID: 19954316 PMID: 19953077 PMID: 19935226 PMID: 19933722 PMID: 19880553 PMID: 19876801 PMID: 19861316 PMID: 19851768 PMID: 19833754 PMID: 19830150 PMID: 19816395 PMID: 19790113 PMID: 19789875 PMID: 19770558 PMID: 19762406 PMID: 19762391 PMID: 19762378 PMID: 19762360 PMID: 19758234 PMID: 19755473 PMID: 19736477 PMID: 19727047 PMID: 19713697 PMID: 19710237 PMID: 19691927 PMID: 19685134 PMID: 19671698 PMID: 19663191 PMID: 19644959 PMID: 19634082 PMID: 19632675 PMID: 19622170 PMID: 19606253 PMID: 19591786 PMID: 19591779 PMID: 19591778 PMID: 19580662 PMID: 19574341 PMID: 19545416 PMID: 19487226 PMID: 19506538 PMID: 19504167 PMID: 19502274 PMID: 19464508 PMID: 19458907 PMID: 19447935 PMID: 19439988 PMID: 19433461 PMID: 19412195 PMID: 19389674 PMID: 19381639 PMID: 19348047 PMID: 19342741 PMID: 19245930 PMID: 19318394 PMID: 19300290 PMID: 19295413 PMID: 19286698 PMID: 19280931 PMID: 19280929 PMID: 19276302 PMID: 19248125 PMID: 19224175 PMID: 19213981 PMID: 19205787 PMID: 19205552 PMID: 19201571 PMID: 19193937 PMID: 19171112 PMID: 19168158 PMID: 19158408 PMID: 19128872 PMID: 19116963 PMID: 19109318 PMID: 19107019 PMID: 19099898 PMID: 19074176 PMID: 19048414 PMID: 19040956 PMID: 19037607 PMID: 19023530 PMID: 18971190 PMID: 18947376 PMID: 18939368 PMID: 18931333 PMID: 18852227 PMID: 18840001 PMID: 18835781 PMID: 18819771 PMID: 18818002 PMID: 18807776 PMID: 18805348 PMID: 18793002 PMID: 18778014 PMID: 18774002 PMID: 18723953 PMID: 18719369 PMID: 18700910 PMID: 18692942 PMID: 18688914 PMID: 18665148 PMID: 18661410 PMID: 18655477 PMID: 18646350 PMID: 18625655 PMID: 18625640 PMID: 18625636 PMID: 18625099 PMID: 18537653 PMID: 18497552 PMID: 18490408 PMID: 18484694 PMID: 18456233 PMID: 18438863 PMID: 18436843 PMID: 18413410 PMID: 18413408 PMID: 18413406 PMID: 18398936 PMID: 18391674 PMID: 18386608 PMID: 18375400 PMID: 18361152 PMID: 18328160 PMID: 18320479 PMID: 18320193 PMID: 18278839 PMID: 18272123 PMID: 18267233 PMID: 18254284 PMID: 18250139 PMID: 18250133 PMID: 18240255 PMID: 18240193 PMID: 18235540 PMID: 18221991 PMID: 18221986 PMID: 18221926 PMID: 18096469 PMID: 18216477 PMID: 18207065 PMID: 18202859 PMID: 18179109 PMID: 19555041 PMID: 19374227 PMID: 18092438 PMID: 18091363 PMID: 18084001 PMID: 18066960 PMID: 18063917 PMID: 18050188 PMID: 18042597 PMID: 18041412 PMID: 18034514 PMID: 17985403 PMID: 17971364 PMID: 17966597 PMID: 17952693 PMID: 17916985 PMID: 17903828 PMID: 17901988 PMID: 17895310 PMID: 17893967 PMID: 17891918 PMID: 17891746 PMID: 17883922 PMID: 17869565 PMID: 17803471 PMID: 17802940 PMID: 17786449 PMID: 17764055 PMID: 17762454 PMID: 17728367 PMID: 17728365 PMID: 17711550 PMID: 17699936 PMID: 17699810 PMID: 17696041 PMID: 17693444 PMID: 17683621 PMID: 17670849 PMID: 17670844 PMID: 17668644 PMID: 17667990 PMID: 17664231 PMID: 17662929 PMID: 17640941 PMID: 17635751 PMID: 17620509 PMID: 17576739 PMID: 17576395 PMID: 17573029 PMID: 17565204 PMID: 17564778 PMID: 17538567 PMID: 17516127 PMID: 17509958 PMID: 17499706 PMID: 17499704 PMID: 17483845 PMID: 17471833 PMID: 17468541 PMID: 17458826 PMID: 17439938 PMID: 17439935 PMID: 17439930 PMID: 17437162 PMID: 17432107 PMID: 17406149 PMID: 17395116 PMID: 17378131 PMID: 17376945 PMID: 17376507 PMID: 17363983 PMID: 17318274 PMID: 17283582 PMID: 17243522 PMID: 17226010 PMID: 17223657 PMID: 17218611 PMID: 17211989 PMID: 17201275 PMID: 17195352 PMID: 17159078 PMID: 17159077 PMID: 17149066 PMID: 17143701 PMID: 17143660 PMID: 17139666 PMID: 17133599 PMID: 17132694 PMID: 17130340 PMID: 17120597 PMID: 17120595 PMID: 17119864 PMID: 17107981 PMID: 17099331 PMID: 17090740 PMID: 17090724 PMID: 17057632 PMID: 17039125 PMID: 17036267 PMID: 17014025 PMID: 17014002 PMID: 17013432 PMID: 16984944 PMID: 16982954 PMID: 16971374 PMID: 16970686 PMID: 16960925 PMID: 16944153 PMID: 16941203 PMID: 16924690 PMID: 16919011 PMID: 16909327 PMID: 16897115 PMID: 16889573 PMID: 16889045 PMID: 16881437 PMID: 16881111 PMID: 16856445 PMID: 16840400 PMID: 16831323 PMID: 16830033 PMID: 16827638 PMID: 16819645 PMID: 19803968 PMID: 16804698 PMID: 16793845 PMID: 16765716 PMID: 16762599 PMID: 16762598 PMID: 16760882 PMID: 16731183 PMID: 16670811 PMID: 16670051 PMID: 16651321 PMID: 16634368 PMID: 16634365 PMID: 16633933 PMID: 16633932 PMID: 16604250 PMID: 16581485 PMID: 16575686 PMID: 16570203 PMID: 16527878 PMID: 16508972 PMID: 16496078 PMID: 16482923 PMID: 16449114 PMID: 16425581 PMID: 16425573 PMID: 16419000 PMID: 16417849 PMID: 16409858 PMID: 16392231 PMID: 16391888 PMID: 16357459 PMID: 16354896 PMID: 16344496 PMID: 16328762 PMID: 16328417 PMID: 16302681 PMID: 16296869 PMID: 16274063 PMID: 16273769 PMID: 16260574 PMID: 16260209 PMID: 16260053 PMID: 16247648 PMID: 16234277 PMID: 16218473 PMID: 16193200 PMID: 16175936 PMID: 16175928 PMID: 16142864 PMID: 16135580 PMID: 16130517 PMID: 16130514 PMID: 16119705 PMID: 16117425 PMID: 16088549 PMID: 16084319 PMID: 16076883 PMID: 16060085 PMID: 16046220 PMID: 16034629 PMID: 16014060 PMID: 15983635 PMID: 15976324 PMID: 15970846 PMID: 15957139 PMID: 15954358 PMID: 15951919 PMID: 15940546 PMID: 15934435 PMID: 15934071 PMID: 15912770 PMID: 15911740 PMID: 15910552 PMID: 15895902 PMID: 15895890 PMID: 15888846 PMID: 15883235 PMID: 15881095 PMID: 15861356 PMID: 15859940 PMID: 15844768 PMID: 15842204 PMID: 15819133 PMID: 15801072 PMID: 15793677 PMID: 15768147 PMID: 15757967 PMID: 15751820 PMID: 15751819 PMID: 15732283 PMID: 15730620 PMID: 15729533 PMID: 15691057 PMID: 15668428 PMID: 15651427 PMID: 15645748 PMID: 15639320 PMID: 15633767 PMID: 15609267 PMID: 15596638 PMID: 15593366 PMID: 15580982 PMID: 15516414 PMID: 15513684 PMID: 15507877 PMID: 15507776 PMID: 15507479 PMID: 15497014 PMID: 15479907 PMID: 15462490 PMID: 15366343 PMID: 15361944 PMID: 15359434 PMID: 15357113 PMID: 15356317 PMID: 15352421 PMID: 15340865 PMID: 15334471 PMID: 15334470 PMID: 15314498 PMID: 15305243 PMID: 15302727 PMID: 15284296 PMID: 15278715 PMID: 15262847 PMID: 15257557 PMID: 15247980 PMID: 15246475 PMID: 15212150 PMID: 15201645 PMID: 15182787 PMID: 15177971 PMID: 15150430 PMID: 15145488 PMID: 15124249 PMID: 15119551 PMID: 15111687 PMID: 15077876 PMID: 15077094 PMID: 15071130 PMID: 15064940 PMID: 15052490 PMID: 15045815 PMID: 15042555 PMID: 15040621 PMID: 15027585 PMID: 15024932 PMID: 15001976 PMID: 14995003 PMID: 14986087 PMID: 14734790 PMID: 14714915 PMID: 14712424 PMID: 14694281 PMID: 14692439 PMID: 14663384 PMID: 14653445 PMID: 14648152 PMID: 14634858 PMID: 14618123 PMID: 14613279 PMID: 14613278 PMID: 14605293 PMID: 14596430 PMID: 14583570 PMID: 14583569 PMID: 14582332 PMID: 14582024 PMID: 14579159 PMID: 14579140 PMID: 14570286 PMID: 14533998 PMID: 14530779 PMID: 14530532 PMID: 14499721 PMID: 12955708 PMID: 12952274 PMID: 12945726 PMID: 12945718 PMID: 12922957 PMID: 12921303 PMID: 12905646 PMID: 12879781 PMID: 12876633 PMID: 12856325 PMID: 12849001 PMID: 12847897 PMID: 12827401 PMID: 12781093 PMID: 12765469 PMID: 12734895 PMID: 12729058 PMID: 12729057 PMID: 12729052 PMID: 12718748 PMID: 12708380 PMID: 12696499 PMID: 12680018 PMID: 12687219 PMID: 12640304 PMID: 12638413 PMID: 12625109 PMID: 12624796 PMID: 12610807 PMID: 12607972 PMID: 12605779 PMID: 12605324 PMID: 12605313 PMID: 12601532 PMID: 12599514 PMID: 12563682 PMID: 12534331 PMID: 12525998 PMID: 12497750 PMID: 12492708 PMID: 12491066 PMID: 12487188 PMID: 12476949 PMID: 12475005 PMID: 12467320 PMID: 12452314 PMID: 12429536 PMID: 12413061 PMID: 12403257 PMID: 12402412 PMID: 12399879 PMID: 12386730 PMID: 12352299 PMID: 12229197 PMID: 12216930 PMID: 12164854 PMID: 12152910 PMID: 12148172 PMID: 12148170 PMID: 12139379 PMID: 12136894 PMID: 12111637 PMID: 12064856 PMID: 12061288 PMID: 12052329 PMID: 12048290 PMID: 12043890 PMID: 12043886 PMID: 12017882 PMID: 12011382 PMID: 11999886 PMID: 19810870 PMID: 11966956 PMID: 11958584 PMID: 11937761 PMID: 19644578 PMID: 11907289 PMID: 11877599 PMID: 11877597 PMID: 11876567 PMID: 11868596 PMID: 11863120 PMID: 11858777 PMID: 11854941 PMID: 11849397 PMID: 11842823 PMID: 11840446 PMID: 11838845 PMID: 11830435 PMID: 11814740 PMID: 11809000 PMID: 11808430 PMID: 11806222 PMID: 11794471 PMID: 11793134 PMID: 11787881 PMID: 11780682 PMID: 11766110 PMID: 11752020 PMID: 11732484 PMID: 11727842 PMID: 11723615 PMID: 11692947 PMID: 11676360 PMID: 11676333 PMID: 11605222 PMID: 11604598 PMID: 11580735 PMID: 11579530 PMID: 11578020 PMID: 11533799 PMID: 11526929 PMID: 11511754 PMID: 11510158 PMID: 11477481 PMID: 11474881 PMID: 11472438 PMID: 11455738 PMID: 11455521 PMID: 11434583 PMID: 11404827 PMID: 11350849 PMID: 11340149 PMID: 11333798 PMID: 11322370 PMID: 11315347 PMID: 11304506 PMID: 11302882 PMID: 11302880 PMID: 11287782 PMID: 11244736 PMID: 11229469 PMID: 11220068 PMID: 11224023 PMID: 11199921 PMID: 11198495 PMID: 11196749 PMID: 11174063 PMID: 11161132 PMID: 10755694 PMID: 11124280 PMID: 11114281 PMID: 11111547 PMID: 11110581 PMID: 11109618 PMID: 11071967 PMID: 11071585 PMID: 11068646 PMID: 11063380 PMID: 11060762 PMID: 11035709 PMID: 11035427 PMID: 11021562 PMID: 11020963 PMID: 10975143 PMID: 10920686 PMID: 19078472 PMID: 10878725 PMID: 10878435 PMID: 10876002 PMID: 10834868 PMID: 10805483 PMID: 10805481 PMID: 10801148 PMID: 10801140 PMID: 10798062 PMID: 10793041 PMID: 10782851 PMID: 10773904 PMID: 10770031 PMID: 10713649 PMID: 10684369 PMID: 10682060 PMID: 10669712 PMID: 10667651 PMID: 10645148 PMID: 10637898 PMID: 10631192 PMID: 10627904 PMID: 10577372 PMID: 10561047 PMID: 10561162 PMID: 10555892 PMID: 10555891 PMID: 10544836 PMID: 10544751 PMID: 10534545 PMID: 10529128 PMID: 10503653 PMID: 10501431 PMID: 10493670 PMID: 10483012 PMID: 10473368 PMID: 10464859 PMID: 10462267 PMID: 10443625 PMID: 10432956 PMID: 10430980 PMID: 10430971 PMID: 10418057 PMID: 10414256 PMID: 10413214 PMID: 10403262 PMID: 10402073 PMID: 10402066 PMID: 10391522 PMID: 10370384 PMID: 10358843 PMID: 10352648 PMID: 10352647 PMID: 10326988 PMID: 10323445 PMID: 10232552 PMID: 10220835 PMID: 10202612 PMID: 10193434 PMID: 10193372 PMID: 10099897 PMID: 10078004 PMID: 10074587 PMID: 10073601 PMID: 10063281 PMID: 9987957 PMID: 9952276 PMID: 9951019 PMID: 9934837 PMID: 9918271 PMID: 9917963 PMID: 9890686 PMID: 9890674 PMID: 9830789 PMID: 9814679 PMID: 9796854 PMID: 9779861 PMID: 9750089 PMID: 9736596 PMID: 9720368 PMID: 9712095 PMID: 9706442 PMID: 9706424 PMID: 9699029 PMID: 9674345 PMID: 9672994 PMID: 9659644 PMID: 9632069 PMID: 9617498 PMID: 9613868 PMID: 9604106 PMID: 9593350 PMID: 9588269 PMID: 9588267 PMID: 9583067 PMID: 9577149 PMID: 9567527 PMID: 9562836 PMID: 9555175 PMID: 9541097 PMID: 9509438 PMID: 9493146 PMID: 9462174 PMID: 9453280 PMID: 9375894 PMID: 9429786 PMID: 9407444 PMID: 9398137 PMID: 9391866 PMID: 9391702 PMID: 9352738 PMID: 9323952 PMID: 9296242 PMID: 19078188 PMID: 9291752 PMID: 9263149 PMID: 9219700 PMID: 9227170 PMID: 9285016 PMID: 9112946 PMID: 9093795 PMID: 9058654 PMID: 9031381 PMID: 9016889 PMID: 9532830 PMID: 9483749 PMID: 9310115 PMID: 9302663 PMID: 9256308 PMID: 9175022 PMID: 9104728 PMID: 9008597 PMID: 9001324 PMID: 8995990 PMID: 8986098 PMID: 9275599 PMID: 9033229 PMID: 9003342 PMID: 8918589 PMID: 19078080 PMID: 8949281 PMID: 9102854 PMID: 8984464 PMID: 8913095 PMID: 8809137 PMID: 8804321 PMID: 9122000 PMID: 8794779 PMID: 8774190 PMID: 8842791 PMID: 8829069 PMID: 8823703 PMID: 8670602 PMID: 19078053 PMID: 8837075 PMID: 8712081 PMID: 8710047 PMID: 8725704 PMID: 8766346 PMID: 8763009 PMID: 9546909 PMID: 8792794 PMID: 8771655 PMID: 8684543 PMID: 8652932 PMID: 8703243 PMID: 8568388 PMID: 8608694 PMID: 8583727 PMID: 8579980 PMID: 7561148 PMID: 19077995 PMID: 8594661 PMID: 8578310 PMID: 8575129 PMID: 8563092 PMID: 8563085 PMID: 7582730 PMID: 8528233 PMID: 7551496 PMID: 7628842 PMID: 7562756 PMID: 7562753 PMID: 7655497 PMID: 7655489 PMID: 7633801 PMID: 7794041 PMID: 7748226 PMID: 7655867 PMID: 7587931 PMID: 7795613 PMID: 7872181 PMID: 7783081 PMID: 7782096 PMID: 8577127 PMID: 7709101 PMID: 7699684 PMID: 7618642 PMID: 7882637 PMID: 7870631 PMID: 7859048 PMID: 7699653 PMID: 7980684 PMID: 7869310 PMID: 7837174 PMID: 7831173 PMID: 8000104 PMID: 7993705 PMID: 7955630 PMID: 7811198 PMID: 8046316 PMID: 17974390 PMID: 8072226 PMID: 8035915 PMID: 7923744 PMID: 8069733 PMID: 7955716 PMID: 7939722 PMID: 7826232 PMID: 8059155 PMID: 8029190 PMID: 8147933 PMID: 8048354 PMID: 8044294 PMID: 8153398 PMID: 7516663 PMID: 8192282 PMID: 8162644 PMID: 8140019 PMID: 8084448 PMID: 8041958 PMID: 7781485 PMID: 7747162 PMID: 7724979 PMID: 8252983 PMID: 8136819 PMID: 8271470 PMID: 8402006 PMID: 8305928 PMID: 8295180 PMID: 8375310 PMID: 17590721 PMID: 8398608 PMID: 8275582 PMID: 8252781 PMID: 8231039 PMID: 8371500 PMID: 8361108 PMID: 8339438 PMID: 8338012 PMID: 8235916 PMID: 8371224 PMID: 8369214 PMID: 8325084 PMID: 8281200 PMID: 8218776 PMID: 8369808 PMID: 8350313 PMID: 8180722 PMID: 8494020 PMID: 8389511 PMID: 8517839 PMID: 8511597 PMID: 8496859 PMID: 8465737 PMID: 8316910 PMID: 8478885 PMID: 8447299 PMID: 8519077 PMID: 15636851 PMID: 8481653 PMID: 8384335 PMID: 8341391 PMID: 8246413 PMID: 8105911 PMID: 1461605 PMID: 1294751 PMID: 1442853 PMID: 1442758 PMID: 1297616 PMID: 1285872 PMID: 1285864 PMID: 1522805 PMID: 1404170 PMID: 1404151 PMID: 1341157 PMID: 1518395 PMID: 1442938 PMID: 1616370 PMID: 1455841 PMID: 1404141 PMID: 1608520 PMID: 1352123 PMID: 1631263 PMID: 1567491 PMID: 1585678 PMID: 1578526 PMID: 1550484 PMID: 1734390 PMID: 1604419 PMID: 1604413 PMID: 1576312 PMID: 1624723 PMID: 1603984 PMID: 1579720 PMID: 1555788 PMID: 1540039 PMID: 1444009 PMID: 1795332 PMID: 1747706 PMID: 1951378 PMID: 1913015 PMID: 1751310 PMID: 1749941 PMID: 1745869 PMID: 1937263 PMID: 1790644 PMID: 1771315 PMID: 1948460 PMID: 1948101 PMID: 1925104 PMID: 2048605 PMID: 2048604 PMID: 1929111 PMID: 2057730 PMID: 2032314 PMID: 1924675 PMID: 1925798 PMID: 1914323 PMID: 2034923 PMID: 2004751 PMID: 2000747 PMID: 1996564 PMID: 1885501 PMID: 1996201 PMID: 2050166 PMID: 2031153 PMID: 1995502 PMID: 1956482 PMID: 1835119 PMID: 2149767 PMID: 2128987 PMID: 2125409 PMID: 2237945 PMID: 2175138 PMID: 2133426 PMID: 2079484 PMID: 2269815 PMID: 2123932 PMID: 2224230 PMID: 2089172 PMID: 2214602 PMID: 2379050 PMID: 2230413 PMID: 2095621 PMID: 2113189 PMID: 2351496 PMID: 2330902 PMID: 1696192 PMID: 2313852 PMID: 2391331 PMID: 2350905 PMID: 2267443 PMID: 2112354 PMID: 2107479 PMID: 2331746 PMID: 2335048 PMID: 2332868 PMID: 2185911 PMID: 2108619 PMID: 1696033 PMID: 2368766 PMID: 2340851 PMID: 2301491 PMID: 2285205 PMID: 2270763 PMID: 2256476 PMID: 2243578 PMID: 2243569 PMID: 2240951 PMID: 2240946 PMID: 2127871 PMID: 2082786 PMID: 2077823 PMID: 2624664 PMID: 2511998 PMID: 2811661 PMID: 2625744 PMID: 2814519 PMID: 2591683 PMID: 2574122 PMID: 2694209 PMID: 2634004 PMID: 2640052 PMID: 2506841 PMID: 2769664 PMID: 2664212 PMID: 2491496 PMID: 2486941 PMID: 2717992 PMID: 2671208 PMID: 2543224 PMID: 9301921 PMID: 2701871 PMID: 2494884 PMID: 2743718 PMID: 2694791 PMID: 2926131 PMID: 3241233 PMID: 2853767 PMID: 3208238 PMID: 3144942 PMID: 3059177 PMID: 3224745 PMID: 3251440 PMID: 3397172 PMID: 3180553 PMID: 3133431 PMID: 3041485 PMID: 3280489 PMID: 2899385 PMID: 2893204 PMID: 3388148 PMID: 3348494 PMID: 3285421 PMID: 3172103 PMID: 3145584 PMID: 2850687 PMID: 3681569 PMID: 3674094 PMID: 3334284 PMID: 3423442 PMID: 3659344 PMID: 3116955 PMID: 3306188 PMID: 3612648 PMID: 3438780 PMID: 3496026 PMID: 3599009 PMID: 3598554 PMID: 3103510 PMID: 3579390 PMID: 3827339 PMID: 3577764 PMID: 3494196 PMID: 3101233 PMID: 3559358 PMID: 3767464 PMID: 3575975 PMID: 3740078 PMID: 3091656 PMID: 3729170 PMID: 3717247 PMID: 3747209 PMID: 3729068 PMID: 3709258 PMID: 3524950 PMID: 2423937 PMID: 3724006 PMID: 3723506 PMID: 3088276 PMID: 3702274 PMID: 3945397 PMID: 3485117 PMID: 3484901 PMID: 2871187 PMID: 3961292 PMID: 3548543 PMID: 3472444 PMID: 3081011 PMID: 2940667 PMID: 2866708 PMID: 3907317 PMID: 3834612 PMID: 3902437 PMID: 4042885 PMID: 12267711 PMID: 4087261 PMID: 3902311 PMID: 3902310 PMID: 2412088 PMID: 4057200 PMID: 3875288 PMID: 12280147 PMID: 4029472 PMID: 4025948 PMID: 4013994 PMID: 3927857 PMID: 3924488 PMID: 3902299 PMID: 3895068 PMID: 3924927 PMID: 4033027 PMID: 4017323 PMID: 3893819 PMID: 3889818 PMID: 2860699 PMID: 4032410 PMID: 3985693 PMID: 3893171 PMID: 3918712 PMID: 15227037 PMID: 3982112 PMID: 3977383 PMID: 3158296 PMID: 2934817 PMID: 3964919 PMID: 3991565 PMID: 3981075 PMID: 3972386 PMID: 3916476 PMID: 3869724 PMID: 2420157 PMID: 6396008 PMID: 6334092 PMID: 6151881 PMID: 6496544 PMID: 6494072 PMID: 6333815 PMID: 6240359 PMID: 6507805 PMID: 6437349 PMID: 6335863 PMID: 6524114 PMID: 6432120 PMID: 6743365 PMID: 6377906 PMID: 6464752 PMID: 6374900 PMID: 6711679 PMID: 6703830 PMID: 6231317 PMID: 6198902 PMID: 6747212 PMID: 6713868 PMID: 6443758 PMID: 6398036 PMID: 6392913 PMID: 6372000 PMID: 6336293 PMID: 6229228 PMID: 6200671 PMID: 6092121 PMID: 6670604 PMID: 6640871 PMID: 6606705 PMID: 6416530 PMID: 6139567 PMID: 6656170 PMID: 6639692 PMID: 6412935 PMID: 6683809 PMID: 6681142 PMID: 6681141 PMID: 6887170 PMID: 6687175 PMID: 6829588 PMID: 6824532 PMID: 6137582 PMID: 6861845 PMID: 6674712 PMID: 6359336 PMID: 6343902 PMID: 6138472 PMID: 7188437 PMID: 7131712 PMID: 7102671 PMID: 7102664 PMID: 7046543 PMID: 7048254 PMID: 7102312 PMID: 7047041 PMID: 6214224 PMID: 6811188 PMID: 6749397 PMID: 6802356 PMID: 7183893 PMID: 7073229 PMID: 7048854 PMID: 6762095 PMID: 7032298 PMID: 6948918 PMID: 6458208 PMID: 7294510 PMID: 7338287 PMID: 7284050 PMID: 7025213 PMID: 6974554 PMID: 7242321 PMID: 6457374 PMID: 6119054 PMID: 6119052 PMID: 7327618 PMID: 6112936 PMID: 7282111 PMID: 6787090 PMID: 6970646 PMID: 7220279 PMID: 7329884 PMID: 7205989 PMID: 7025253 PMID: 6939056 PMID: 7305172 PMID: 7026486 PMID: 6758576 PMID: 7460402 PMID: 7206461 PMID: 7443421 PMID: 7433293 PMID: 7309597 PMID: 6968159 PMID: 6968614 PMID: 7002438 PMID: 7001292 PMID: 7382059 PMID: 7392705 PMID: 7432149 PMID: 7392379 PMID: 7368127 PMID: 7352785 PMID: 7256014 PMID: 6156358 PMID: 6156349 PMID: 6156345 PMID: 92663 PMID: 519434 PMID: 534238 PMID: 583290 PMID: 522235 PMID: 40121 PMID: 544546 PMID: 508975 PMID: 474602 PMID: 89119 PMID: 158872 PMID: 502409 PMID: 472799 PMID: 387573 PMID: 496185 PMID: 528479 PMID: 372870 PMID: 522935 PMID: 439109 PMID: 433252 PMID: 425997 PMID: 92157 PMID: 740571 PMID: 699653 PMID: 704382 PMID: 351308 PMID: 96037 PMID: 96756 PMID: 745633 PMID: 618932 PMID: 370560 PMID: 24154 PMID: 923317 PMID: 604410 PMID: 600598 PMID: 22129 PMID: 143893 PMID: 913002 PMID: 901973 PMID: 70861 PMID: 916237 PMID: 888141 PMID: 867009 PMID: 69300 PMID: 850811 PMID: 325635 PMID: 869275 PMID: 835580 PMID: 70887 PMID: 16356 PMID: 1028032 PMID: 988514 PMID: 796535 PMID: 1085678 PMID: 962443 PMID: 957598 PMID: 778618 PMID: 934532 PMID: 1276547 PMID: 943695 PMID: 772901 PMID: 1258821 PMID: 1246541 PMID: 1082750 PMID: 971885 PMID: 139084 PMID: 10134 PMID: 175438 PMID: 1214887 PMID: 134371 PMID: 1241263 PMID: 1190852 PMID: 1180978 PMID: 50622 PMID: 973 PMID: 50845 PMID: 50009 PMID: 241733 PMID: 1160186 PMID: 1123986 PMID: 1121994 PMID: 238186 PMID: 803853 PMID: 1115070 PMID: 1168295 PMID: 242119 PMID: 4460396 PMID: 4445503 PMID: 4142960 PMID: 4427204 PMID: 4597950 PMID: 4132372 PMID: 4830511 PMID: 4128966 PMID: 4583700 PMID: 4199150 PMID: 4755798 PMID: 4711347 PMID: 4696623 PMID: 4755003 PMID: 4147359 PMID: 4540388 PMID: 4780465 PMID: 4268632 PMID: 4345117 PMID: 4695164 PMID: 4581906 PMID: 4579028 PMID: 4154537 PMID: 4154432 PMID: 4651839 PMID: 4647128 PMID: 4569486 PMID: 4558925 PMID: 4561565 PMID: 4536922 PMID: 5052347 PMID: 4941069 PMID: 5150207 PMID: 5292991 PMID: 4399662 PMID: 4105025 PMID: 4105122 PMID: 5145076 PMID: 4397770 PMID: 4937335 PMID: 4118529 PMID: 4947941 PMID: 4395690 PMID: 4320121 PMID: 4247287 PMID: 4393768 PMID: 4108772 PMID: 4906113 PMID: 5410395 PMID: 5293512 PMID: 4398206 PMID: 5378243 PMID: 5797074 PMID: 5381388 PMID: 5345413 PMID: 4388556 PMID: 4332262 PMID: 5376531 PMID: 4238441 PMID: 5711657 PMID: 4182936 PMID: 4386252 PMID: 5661884 PMID: 4385956 PMID: 4869684 PMID: 5653607 PMID: 5687881 PMID: 4950805 PMID: 4174632 PMID: 4172505 PMID: 6057644 PMID: 5587420 PMID: 5599101 PMID: 6025850 PMID: 4865110 PMID: 4864895 PMID: 4959828 PMID: 4387767 PMID: 5594742 PMID: 4872127 PMID: 5928932 PMID: 5335275 PMID: 5321759 PMID: 14321237 PMID: 14242524 PMID: 14258348 PMID: 14212307 PMID: 14206255 PMID: 14193537 PMID: 14183490 PMID: 14162899 PMID: 14121290 PMID: 14176299 PMID: 14129517 PMID: 14217829 PMID: 14129926 PMID: 14203060 PMID: 14105024 PMID: 14102709 PMID: 14066190 PMID: 14051185 PMID: 14168386 PMID: 14095801 PMID: 14054588 PMID: 13953420 PMID: 13835831 PMID: 13618605 PMID: 14375421 |
|  |
| T cell receptor signaling pathway |  |
| Taste transduction | PMID: 3384467 |
| Terpenoid backbone biosynthesis |  |
| TGF-beta signaling pathway | PMID: 18583708 PMID: 16530037 |
| Thyroid cancer | PMID: 20967300 PMID: 20694064 PMID: 20472683 PMID: 20456972 PMID: 19856714 PMID: 19564535 PMID: 19349511 PMID: 19169490 PMID: 18987277 PMID: 18797435 PMID: 18646485 PMID: 18596272 PMID: 18541897 PMID: 16835280 PMID: 16828412 PMID: 16584519 PMID: 15771139 PMID: 15671774 PMID: 15513540 PMID: 15328326 PMID: 12441836 PMID: 12074506 PMID: 11130252 PMID: 10597976 PMID: 10454461 PMID: 8885814 PMID: 8822754 PMID: 8644032 PMID: 7970809 PMID: 7049681 PMID: 704557 PMID: 995598 PMID: 13913237 |
| Tight junction | PMID: 20802087 PMID: 20577123 PMID: 19949573 PMID: 19922391 PMID: 19842891 PMID: 19721377 PMID: 19094838 PMID: 18931049 PMID: 18840681 PMID: 18097471 PMID: 17690246 PMID: 17660022 PMID: 17420334 PMID: 17347984 PMID: 17255128 PMID: 17234953 PMID: 15907795 PMID: 15855658 PMID: 15854590 PMID: 15763998 PMID: 15637347 PMID: 15465913 PMID: 15070779 PMID: 14968742 PMID: 12698368 PMID: 12353135 PMID: 12053015 PMID: 11102579 PMID: 11063220 PMID: 9315370 PMID: 7510767 PMID: 4007987 PMID: 6334092 PMID: 636839 |
| Toll-like receptor signaling pathway |  |
| Tryptophan metabolism | PMID: 9516677 PMID: 1772753 PMID: 2829704 PMID: 791557 PMID: 12256711 |
| Type I diabetes mellitus | PMID: 20164848 PMID: 17493551 PMID: 16856705 PMID: 16848868 PMID: 16617731 PMID: 16340402 PMID: 16309480 PMID: 12768043 PMID: 12761421 PMID: 12680017 PMID: 11380077 PMID: 11151763 PMID: 11012901 PMID: 10937927 PMID: 10768096 PMID: 10761866 PMID: 10491759 PMID: 9950302 PMID: 9833937 PMID: 9748739 PMID: 9580167 PMID: 9527979 PMID: 9438777 PMID: 9082261 PMID: 9354852 PMID: 8767069 PMID: 7558219 PMID: 8560902 PMID: 7626986 PMID: 7839327 PMID: 7971856 PMID: 8273249 PMID: 8341335 PMID: 8341492 PMID: 8481551 PMID: 1436257 PMID: 1610979 PMID: 1514307 PMID: 1440323 PMID: 1314967 PMID: 1889846 PMID: 1888240 PMID: 2087208 PMID: 2245880 PMID: 2224980 PMID: 2186611 PMID: 2754382 PMID: 2529455 PMID: 3236757 PMID: 3227321 PMID: 3696399 PMID: 4039409 PMID: 3985037 |
| Type II diabetes mellitus | PMID: 21045250 PMID: 20880826 PMID: 20851291 PMID: 20822856 PMID: 20558843 PMID: 20521902 PMID: 20497980 PMID: 20485966 PMID: 20410917 PMID: 20400141 PMID: 20370673 PMID: 20037278 PMID: 20337636 PMID: 20164784 PMID: 19830030 PMID: 19690664 PMID: 19687357 PMID: 19662657 PMID: 19637764 PMID: 21141002 PMID: 19491987 PMID: 19460605 PMID: 19347033 PMID: 19322204 PMID: 19230052 PMID: 19208721 PMID: 19199921 PMID: 19149532 PMID: 19067997 PMID: 19030139 PMID: 18955282 PMID: 18936798 PMID: 18854748 PMID: 18753719 PMID: 18752660 PMID: 18627637 PMID: 18617965 PMID: 18510739 PMID: 18274353 PMID: 18273039 PMID: 18068832 PMID: 18059349 PMID: 18040744 PMID: 18035918 PMID: 17967715 PMID: 17879019 PMID: 19885145 PMID: 17513208 PMID: 17496526 PMID: 17435887 PMID: 17359485 PMID: 17318769 PMID: 17273153 PMID: 17044598 PMID: 17008856 PMID: 16673014 PMID: 16672921 PMID: 16647437 PMID: 16613007 PMID: 16606865 PMID: 16598198 PMID: 16526203 PMID: 16512787 PMID: 16464169 PMID: 16433936 PMID: 16304314 PMID: 16246041 PMID: 16236932 PMID: 16181390 PMID: 16118923 PMID: 16092653 PMID: 16084231 PMID: 16084229 PMID: 15992403 PMID: 15990558 PMID: 15956930 PMID: 15905888 PMID: 15901019 PMID: 15855877 PMID: 15772518 PMID: 15560275 PMID: 15513385 PMID: 15479958 PMID: 15389849 PMID: 15331208 PMID: 15192624 PMID: 15085237 PMID: 15083319 PMID: 15071488 PMID: 15051749 PMID: 15006938 PMID: 14985776 PMID: 14682153 PMID: 14679791 PMID: 14657618 PMID: 14577061 PMID: 12905620 PMID: 12830537 PMID: 12723193 PMID: 12715635 PMID: 12669563 PMID: 12559191 PMID: 12454518 PMID: 12440233 PMID: 12422546 PMID: 12369710 PMID: 12270765 PMID: 12126190 PMID: 12092742 PMID: 12085419 PMID: 12085417 PMID: 12072586 PMID: 11928586 PMID: 19475216 PMID: 11887194 PMID: 11853022 PMID: 11851201 PMID: 11845222 PMID: 11834137 PMID: 11798874 PMID: 11798646 PMID: 11787468 PMID: 11697754 PMID: 11642211 PMID: 11416592 PMID: 11386855 PMID: 19667562 PMID: 11356159 PMID: 11307863 PMID: 11285041 PMID: 11284486 PMID: 11207863 PMID: 11179243 PMID: 11110243 PMID: 11082217 PMID: 11040246 PMID: 10981093 PMID: 10836728 PMID: 10710947 PMID: 10692751 PMID: 10596965 PMID: 10548140 PMID: 10548135 PMID: 10423654 PMID: 10409902 PMID: 10352918 PMID: 10235521 PMID: 10232494 PMID: 10081124 PMID: 9988106 PMID: 9881813 PMID: 9847683 PMID: 9834387 PMID: 9833177 PMID: 9820056 PMID: 9801931 PMID: 9794557 PMID: 9794114 PMID: 9506190 PMID: 9438777 PMID: 9415940 PMID: 9294791 PMID: 9291199 PMID: 9274899 PMID: 9178143 PMID: 9134273 PMID: 9235072 PMID: 9135386 PMID: 9082275 PMID: 9082261 PMID: 9048349 PMID: 9434051 PMID: 9297274 PMID: 9036570 PMID: 8972894 PMID: 9019849 PMID: 11416394 PMID: 8877314 PMID: 8873490 PMID: 8685566 PMID: 8858204 PMID: 8842503 PMID: 8833556 PMID: 9050034 PMID: 8834376 PMID: 8587222 PMID: 7498975 PMID: 8573957 PMID: 7491450 PMID: 7558466 PMID: 7784742 PMID: 7811439 PMID: 8059778 PMID: 8122058 PMID: 8131178 PMID: 7898093 PMID: 7800207 PMID: 7519694 PMID: 8285181 PMID: 8246767 PMID: 8341494 PMID: 8398010 PMID: 8221739 PMID: 8503436 PMID: 8503434 PMID: 8482434 PMID: 8465818 PMID: 8477149 PMID: 8466715 PMID: 8023075 PMID: 1338384 PMID: 1439393 PMID: 1568757 PMID: 1580275 PMID: 1546485 PMID: 1378156 PMID: 1284137 PMID: 1742694 PMID: 1749445 PMID: 1914199 PMID: 1888240 PMID: 1877473 PMID: 1944944 PMID: 2021449 PMID: 1992657 PMID: 1772997 PMID: 1950378 PMID: 1824760 PMID: 1723469 PMID: 2090512 PMID: 2096209 PMID: 2076856 PMID: 16989076 PMID: 2207005 PMID: 2196040 PMID: 2077598 PMID: 2371785 PMID: 2302332 PMID: 1695314 PMID: 1693716 PMID: 2688413 PMID: 2587885 PMID: 2729268 PMID: 2646216 PMID: 2660616 PMID: 2913774 PMID: 2746999 PMID: 2693148 PMID: 2681326 PMID: 3177228 PMID: 3046353 PMID: 3402301 PMID: 3290928 PMID: 3360564 PMID: 3283727 PMID: 3048053 PMID: 3325392 PMID: 3608799 PMID: 3550310 PMID: 3830211 PMID: 3812254 PMID: 3530589 PMID: 3541977 PMID: 3712472 PMID: 3534866 PMID: 3836300 PMID: 6530115 PMID: 6500167 PMID: 6240029 PMID: 6320388 PMID: 6751740 |
| Tyrosine metabolism | PMID: 17513431 PMID: 1755159 PMID: 2566501 PMID: 3091928 |
| Ubiquitin mediated proteolysis | PMID: 11001820 |
| Valine, leucine and isoleucine biosynthesis | PMID: 11780353 PMID: 10462368 PMID: 9928759 PMID: 2312039 PMID: 291018 PMID: 595605 PMID: 5448482 |
| Vascular smooth muscle contraction | PMID: 21193589 PMID: 21124793 PMID: 20923405 PMID: 20921425 PMID: 20727219 PMID: 20546781 PMID: 20419111 PMID: 20041838 PMID: 19203272 PMID: 18971541 PMID: 17201405 PMID: 16713535 PMID: 16687663 PMID: 16523421 PMID: 16474208 PMID: 16339838 PMID: 16230885 PMID: 16053829 PMID: 15983238 PMID: 15215652 PMID: 15010899 PMID: 14707008 PMID: 14500337 PMID: 12900430 PMID: 12885796 PMID: 12791705 PMID: 12689814 PMID: 12668585 PMID: 12655118 PMID: 12623997 PMID: 12542447 PMID: 12530938 PMID: 11882584 PMID: 11882575 PMID: 11785064 PMID: 11487094 PMID: 11359365 PMID: 11116125 PMID: 10614146 PMID: 10501091 PMID: 10499160 PMID: 10383844 PMID: 9816709 PMID: 9682917 PMID: 9403593 PMID: 8675250 PMID: 8734349 PMID: 8769962 PMID: 8689271 PMID: 8834705 PMID: 8778153 PMID: 7490139 PMID: 7782936 PMID: 7762002 PMID: 7781861 PMID: 8206600 PMID: 7506699 PMID: 8263773 PMID: 8393598 PMID: 8477149 PMID: 8385958 PMID: 1315823 PMID: 1725896 PMID: 1661838 PMID: 1937688 PMID: 1770474 PMID: 1832873 PMID: 1879725 PMID: 2030808 PMID: 1893611 PMID: 2078315 PMID: 2503687 PMID: 2538392 PMID: 2719749 PMID: 2452568 PMID: 2467096 PMID: 2824362 PMID: 3544831 PMID: 3810763 PMID: 3530604 PMID: 2856752 PMID: 2982537 PMID: 6091449 PMID: 6723990 PMID: 6143260 PMID: 6103867 PMID: 1231771 PMID: 5506153 |
| VEGF signaling pathway | PMID: 21146124 PMID: 20197639 PMID: 19789317 PMID: 19773379 PMID: 16160062 PMID: 15893841 PMID: 14988842 |
| Vibrio cholerae infection |  |
| Viral myocarditis | PMID: 19214409 PMID: 18346349 PMID: 18070473 PMID: 17896511 PMID: 16524176 PMID: 14677036 PMID: 11826236 PMID: 11715333 PMID: 11309531 PMID: 9990524 PMID: 9539009 PMID: 9490376 PMID: 8376702 PMID: 2278609 PMID: 3403790 PMID: 3302169 PMID: 2935758 PMID: 448785 PMID: 842436 |
| Wnt signaling pathway | PMID: 20947863 PMID: 20177000 PMID: 17622581 PMID: 17332414 PMID: 17211444 PMID: 16387667 |
