# Supplementary material for: Disease Gene Interaction Pathways: A Potential Framework for How Disease Genes Associate by Disease-Risk Modules
Source: PLoS One. 2011 Sep 6;6(9):e24495. doi: 10.1371/journal.pone.0024495 (PMC3167857; doi:10.1371/journal.pone.0024495)
Supplement: Table S6 — PubMed ID in which gene pairs between interacting disease-risk terms have been proved to be correlated with HT. (DOC) [file pone.0024495.s009.doc]

**Table S6. PubMed ID in which gene pairs between interacting disease-risk terms have been proved to be correlated with HT.**

| **interacting disease risk terms pairs** | | **gene pairs between disease risk terms** | **PumMed ID that support the relationship between disease risk term pairs** |
| --- | --- | --- | --- |
|  | 6497 & 5797 |  |  |
|  | 6497 & 5600 |  |  |
|  | 6497 & 5931 | *NOSTRIN* & *NOS3* | PMID: 20082095 |
|  |  | *NOSIP* & *NOS3* | PMID: 20082095 |
|  | 6497 & 5406 |  |  |
|  | 6398 & 5040 |  |  |
|  | 6398 & 2941 | *PTEN* & *SRC* | PMID: 16973905 PMID: 11847190 |
|  | 6398 & 3426 |  |  |
|  | 6398 & 5928 |  |  |
|  | 6398 & 4832 | *ADRA1A* & *ADRA1B* | PMID: 18297111 PMID: 18246093 PMID: 17603545 PMID: 17384126 PMID: 16585965 PMID: 16585959 PMID: 15824464 |
|  | 6398 & 5934 |  |  |
|  | 6398 & 5704 |  |  |
|  | 5040 & 2936 |  |  |
|  | 5040 & 638 | *IKBKAP* & *PLA2G7* | PMID: 17137217 |
|  | 5040 & 6019 |  |  |
|  | 5040 & 5725 |  |  |
|  | 5040 & 5204 |  |  |
|  | 2936 & 638 |  |  |
|  | 2936 & 4945 |  |  |
|  | 2936 & 5571 |  |  |
|  | 2936 & 5327 |  |  |
|  | 2936 & 4950 |  |  |
|  | 2936 & 6125 |  |  |
|  | 2936 & 5497 | *HLA-DQA1* & *HLA-DRB1* | PMID: 12373032 |
|  | 2936 & 5442 |  |  |
|  | 2936 & 2880 |  |  |
|  | 2936 & 4294 |  |  |
|  | 638 & 2941 | *GPX1* & *FYN* | PMID: 17334644 |
|  |  | *KY* & *SRC* | PMID: 17634416 |
|  |  | *PI3* & *SRC* | PMID: 17003231 PMID: 11673836 PMID: 10916078 |
|  |  | *SHE* & *SRC* | PMID: 20969543 PMID: 17039106 PMID: 9654914 PMID: 7967179 |
|  | 638 & 3893 | *GPX1* & *COL1A2* | PMID: 17334644 |
|  |  | *MTHFR* & *COL1A1* | PMID: 15530908 |
|  | 638 & 4208 | *CNP* & *F2* | PMID: 10509361 |
|  |  | *INDO* & *F2* | PMID: 262078 |
|  |  | *RPE* & *MMP2* | PMID: 19580809 |
|  |  | *KY* & *MMP2* | PMID: 17848984 |
|  |  | *SHE* & *F2* | PMID: 2604661 |
|  | 638 & 4945 | *KY* & *LDLR* | PMID: 19252100 |
|  |  | *SHE* & *APP* | PMID: 12421219 |
|  |  | *MTHFR* & *LRP1* | PMID: 15121769 |
|  | 638 & 5571 | *TAGLN* & *ATF4* | PMID: 12963864 |
|  |  | *PLA2G7* & *CAST* | PMID: 17137217 |
|  |  | *SHE* & *CAST* | PMID: 20007085 PMID: 16830033 PMID: 8777975 PMID: 8302154 |
|  |  | *SHE* & *NKX2-1* | PMID: 18957494 |
|  |  | *SCG2* & *ATF4* | PMID: 12963864 |
|  |  | *ASNS* & *ATF4* | PMID: 12963864 |
|  | 638 & 5331 | *FASN* & *TNF* | PMID: 18700276 |
|  |  | *AIF1* & *TNF* | PMID: 20808962 |
|  |  | *GPX1* & *TNF* | PMID: 18600213 |
|  |  | *PI3* & *TNF* | PMID: 15755871 |
|  |  | *SHE* & *TNF* | PMID: 20346246 PMID: 19201996 PMID: 17151265 |
|  |  | *SHE* & *HP* | PMID: 12908798 |
|  |  | *MTHFR* & *TNF* | PMID: 20357201 PMID: 16369102 |
|  |  | *MTHFR* & *HP* | PMID: 16369102 PMID: 10869114 |
|  |  | *ALAS1* & *TNF* | PMID: 16181105 |
|  | 638 & 5928 | *GPX1* & *CAV1* | PMID: 17334644 |
|  | 638 & 5327 |  |  |
|  | 638 & 2437 | *CNP* & *NKX2-5* | PMID: 20433683 |
|  |  | *MTR* & *PON1* | PMID: 18635682 |
|  |  | *KIF6* & *PON1* | PMID: 21044781 |
|  |  | *SHE* & *PNKD* | PMID: 17420335 |
|  |  | *MTHFR* & *PON1* | PMID: 19280995 PMID: 19254215 PMID: 18635682 PMID: 16521945 PMID: 16077191 |
|  | 638 & 4950 | *SLC22A6* & *NR3C2* | PMID: 14596636 |
|  | 638 & 5832 | *CNP* & *NOS2A* | PMID: 10826560 |
|  | 638 & 6125 | *PLA2G7* & *LPL* | PMID: 17137217 |
|  |  | *MTHFR* & *LPL* | PMID: 19254215 PMID: 18704761 PMID: 16894468 PMID: 16369102 |
|  |  | *MTHFR* & *CETP* | PMID: 17785925 |
|  | 638 & 5920 | *INDO* & *REST* | PMID: 9314433 PMID: 8551489 PMID: 7731059 PMID: 7755454 PMID: 1415809 |
|  |  | *PGD* & *REST* | PMID: 9616851 |
|  |  | *RPE* & *REST* | PMID: 14703273 PMID: 8776008 |
|  |  | *ARC* & *REST* | PMID: 19907435 PMID: 15999473 PMID: 12095402 PMID: 1617414 |
|  |  | *KY* & *REST* | PMID: 20609789 PMID: 19449057 PMID: 10421088 PMID: 8379718 PMID: 2066653 |
|  |  | *CARS* & *REST* | PMID: 17394732 |
|  |  | *SHE* & *REST* | PMID: 21248622 PMID: 21240182 PMID: 20824546 PMID: 20526478 PMID: 20409335 PMID: 18853683 PMID: 18758141 PMID: 18432179 PMID: 18069146 PMID: 17557672 PMID: 19610566 PMID: 16849665 PMID: 16822446 PMID: 16673650 PMID: 16423214 PMID: 15586539 PMID: 15470587 PMID: 15295684 PMID: 15077852 PMID: 14501862 PMID: 12808278 PMID: 11996960 PMID: 11974533 PMID: 11913582 PMID: 10512094 PMID: 10454694 PMID: 9670518 PMID: 9497510 PMID: 8687355 PMID: 8840181 PMID: 8652921 PMID: 7817619 PMID: 8235114 PMID: 1330942 PMID: 2794602 PMID: 6411583 PMID: 182944 |
|  |  | *MTHFR* & *REST* | PMID: 19799197 PMID: 19013496 |
|  |  | *RRH* & *REST* |  |
|  |  | *CTA-216E10.6* & *REST* | PMID: 18262666 |
|  | 638 & 5442 | *INDO* & *AVP* | PMID: 17101575 PMID: 8166228 |
|  |  | *ARC* & *AVP* | PMID: 11257787 PMID: 9464185 PMID: 8641721 PMID: 6697213 |
|  |  | *SHE* & *IDE* | PMID: 9545829 |
|  | 638 & 5975 | *CNP* & *ACE* | PMID: 11591626 PMID: 11476746 PMID: 10340842 PMID: 9869508 PMID: 9793068 |
|  |  | *CNP* & *MEOX2* | PMID: 9039131 |
|  |  | *INDO* & *ACE* | PMID: 16202851 PMID: 9894375 PMID: 9361498 PMID: 9287263 PMID: 8941121 PMID: 2561022 PMID: 3033687 |
|  |  | *PGD* & *ACE* | PMID: 18276980 |
|  |  | *RPE* & *ACE* | PMID: 8814748 |
|  |  | *TNFSF4* & *COMT* | PMID: 17016617 |
|  |  | *AIRE* & *ACE* | PMID: 12940534 PMID: 12639174 PMID: 11835905 PMID: 10654389 PMID: 10455471 PMID: 8677863 PMID: 7674273 PMID: 7774515 PMID: 7965277 PMID: 8042078 PMID: 1302164 |
|  |  | *GPX1* & *ACE* | PMID: 21053180 |
|  |  | *ARC* & *ACE* | PMID: 1893643 |
|  |  | *PI3* & *ACE* | PMID: 18855718 |
|  |  | *LARS* & *ACE* | PMID: 16823583 |
|  |  | *KIF6* & *ACE* | PMID: 21044781 |
|  |  | *SLC22A6* & *ACE* | PMID: 14596636 |
|  |  | *FRK* & *ACE* | PMID: 18212275 |
|  |  | *SHE* & *ACE* | PMID: 20726206 PMID: 20587399 PMID: 20180980 PMID: 19531936 PMID: 18453163 PMID: 18405793 PMID: 18320317 PMID: 18081237 PMID: 17415337 PMID: 17384371 PMID: 17285209 PMID: 17039106 PMID: 16342054 PMID: 15964339 PMID: 15912333 PMID: 15219079 PMID: 15191027 PMID: 12703406 PMID: 9105169 PMID: 8594963 PMID: 7519541 PMID: 7919557 PMID: 8272709 PMID: 8385374 PMID: 1480244 |
|  |  | *MTHFR* & *COMT* | PMID: 17016617 |
|  |  | *MTHFR* & *ACE* | PMID: 20162297 PMID: 19882941 PMID: 19810824 PMID: 19776610 PMID: 19580662 PMID: 19280995 PMID: 19254215 PMID: 19076077 PMID: 18685811 PMID: 18200034 PMID: 17146542 PMID: 16443328 PMID: 16369102 PMID: 16081343 PMID: 15226090 PMID: 15151261 PMID: 15121769 PMID: 14638877 |
|  |  | *MCC* & *ACE* | PMID: 12047032 PMID: 10069682 |
|  |  | *CTA-216E10.6* & *ACE* | PMID: 11024215 |
|  |  | *FAU* & *ACE* | PMID: 12915047 |
|  |  | *SMS* & *ACE* | PMID: 1335803 |
|  | 638 & 5864 | *ABCB1* & *ADD1* | PMID: 21106941 |
|  |  | *PI3* & *CD34* | PMID: 18793110 |
|  |  | *SHE* & *HMMR* | PMID: 19825830 |
|  |  | *SHE* & *CD34* | PMID: 20610922 PMID: 19523797 PMID: 19287116 PMID: 18785134 PMID: 18720751 PMID: 14571436 |
|  | 638 & 2880 | *SHE* & *BMPR2* | PMID: 18792970 PMID: 17211315 |
|  | 638 & 4958 | *TNFSF4* & *APOE* | PMID: 17016617 |
|  |  | *KY* & *APOE* | PMID: 19252100 |
|  |  | *SHE* & *APOE* | PMID: 15181857 |
|  |  | *MTHFR* & *APOE* | PMID: 19853876 PMID: 19742390 PMID: 19254215 PMID: 18704761 PMID: 17016617 PMID: 16443328 PMID: 16194201 PMID: 15692115 PMID: 15121769 |
|  | 638 & 5441 | *PI3* & *SLC6A2* | PMID: 15662030 |
|  | 638 & 6361 | *KY* & *TERT* | PMID: 12646410 |
|  | 3754 & 2941 |  |  |
|  | 3754 & 5331 | *TNFRSF1A* & *TNF* | PMID: 18504118 PMID: 17048217 |
|  |  | *CD40* & *TNF* | PMID: 15630673 PMID: 10798271 |
|  |  | *TNFRSF1B* & *TNF* | PMID: 12878376 PMID: 11357933 PMID: 11315843 PMID: 10942422 |
|  | 3754 & 5928 |  |  |
|  | 3754 & 4950 |  |  |
|  | 3754 & 5689 |  |  |
|  | 3269 & 5571 |  |  |
|  | 3269 & 5062 | *ESRRG* & *PPARGC1A* | PMID: 18375192 |
|  | 3269 & 4950 |  |  |
|  | 3269 & 5442 |  |  |
|  | 3269 & 5975 | *VDR* & *ACE* | PMID: 20099993 PMID: 19891555 PMID: 15225806 |
|  |  | *RARA* & *ACE* | PMID: 9618073 |
|  |  | *PPARA* & *ACE* | PMID: 19254215 PMID: 19076077 PMID: 14671555 |
|  |  | *PPARG* & *ACE* | PMID: 20099993 |
|  | 2941 & 3351 | *SRC* & *IRS1* | PMID: 17646573 |
|  |  | *SHC1* & *IRS1* | PMID: 16877964 |
|  |  | *SHC1* & *IRS2* | PMID: 16877964 |
|  | 2941 & 4945 |  |  |
|  | 2941 & 4907 | *SRC* & *CD9* | PMID: 15158909 |
|  | 2941 & 4891 |  |  |
|  | 2941 & 4284 | *SRC* & *MAP2K1* | PMID: 12388158 |
|  |  | *SRC* & *MAP2K2* | PMID: 12388158 |
|  | 2941 & 5331 | *SRC* & *HP* | PMID: 20012923 PMID: 19531752 PMID: 17159723 |
|  |  | *SYK* & *TNF* | PMID: 14613935 |
|  | 2941 & 5707 | *FYN* & *ADRB1* | PMID: 17334644 |
|  | 2941 & 5923 | *SRC* & *EGF* | PMID: 20977469 PMID: 20852045 PMID: 18282556 PMID: 17911376 PMID: 17350025 PMID: 15814837 PMID: 14717925 PMID: 12676164 PMID: 11304462 PMID: 10916078 PMID: 10559135 |
|  |  | *GRB2* & *EGF* | PMID: 10559135 PMID: 9856979 |
|  |  | *FYN* & *EGF* | PMID: 12676164 |
|  | 2941 & 5928 | *SRC* & *CAV1* | PMID: 15768830 |
|  |  | *FYN* & *CAV1* | PMID: 17334644 |
|  | 2941 & 4445 | *SHC1* & *HMOX1* | PMID: 19041334 |
|  | 2941 & 5327 |  |  |
|  | 2941 & 6019 | *SRC* & *ADRA2A* | PMID: 18250367 |
|  | 2941 & 4950 |  |  |
|  | 2941 & 5832 |  |  |
|  | 2941 & 5833 | *SRC* & *CAT* | PMID: 10827015 |
|  | 2941 & 5797 |  |  |
|  | 2941 & 4224 |  |  |
|  | 2941 & 5934 | *SRC* & *POLI* | PMID: 16707113 |
|  | 2941 & 5475 |  |  |
|  | 2941 & 5864 | *FYN* & *ADD1* | PMID: 19838659 |
|  |  | *FYN* & *ADD2* | PMID: 19838659 |
|  | 2941 & 5689 |  |  |
|  | 2941 & 5931 | *SRC* & *NOS3* | PMID: 18981321 PMID: 14557279 |
|  |  | *LYN* & *NOS3* | PMID: 15712782 PMID: 15233974 |
|  | 2941 & 4550 | *SRC* & *TYK2* | PMID: 16141358 PMID: 9892142 PMID: 9212588 PMID: 9130441 |
|  | 2941 & 4294 | *SRC* & *TRPC6* | PMID: 16530601 |
|  | 2941 & 6361 | *SRC* & *TERT* | PMID: 19739668 PMID: 17283870 PMID: 12234822 PMID: 11566939 |
|  |  | *LCK* & *TERT* | PMID: 19739668 |
|  | 2941 & 5406 |  |  |
|  | 3351 & 4891 |  |  |
|  | 3351 & 4284 |  |  |
|  | 3351 & 5707 |  |  |
|  | 3351 & 5928 |  |  |
|  | 3351 & 5327 |  |  |
|  | 3351 & 4224 |  |  |
|  | 3351 & 5442 |  |  |
|  | 3351 & 5931 |  |  |
|  | 3351 & 4550 |  |  |
|  | 3351 & 6361 |  |  |
|  | 3893 & 4208 | *COL1A1* & *MMP2* | PMID: 20194304 |
|  |  | *COL3A1* & *MMP2* | PMID: 20194304 |
|  |  | *COL4A1* & *MMP2* | PMID: 20194304 |
|  | 3893 & 4945 | *FN1* & *APP* | PMID: 18723004 |
|  | 3893 & 4907 | *COL3A1* & *PECAM1* | PMID: 19424605 |
|  | 3893 & 4199 | *COL1A1* & *COL7A1* |  |
|  |  | *COL3A1* & *COL7A1* | PMID: 20194304 |
|  |  | *COL4A1* & *COL7A1* | PMID: 20194304 |
|  | 3893 & 2292 | *COL3A1* & *MMP3* | PMID: 19424605 |
|  |  | *COL3A1* & *MMP1* | PMID: 19424605 |
|  | 3893 & 4209 |  |  |
|  | 3893 & 5928 | *COL1A2* & *CAV1* | PMID: 17334644 |
|  |  | *FN1* & *CAV1* | PMID: 21075447 |
|  | 3893 & 3748 |  |  |
|  | 3893 & 4571 |  |  |
|  | 3893 & 5316 | *FBN1* & *AGT* | PMID: 20537417 |
|  | 3893 & 5365 |  |  |
|  | 3893 & 5934 | *COL1A1* & *COL5A3* | PMID: 20194304 PMID: 15054833 |
|  |  | *COL1A2* & *COL5A3* | PMID: 15054833 |
|  |  | *COL3A1* & *COL5A3* | PMID: 20194304 PMID: 15054833 |
|  |  | *FN1* & *COL5A3* | PMID: 15054833 |
|  |  | *COL4A1* & *COL5A3* | PMID: 20194304 |
|  | 3893 & 5920 | *COL1A1* & *COL6A2* | PMID: 15054833 |
|  |  | *COL1A2* & *COL6A2* | PMID: 15054833 |
|  |  | *COL2A1* & *COL6A2* | PMID: 15054833 |
|  |  | *COL3A1* & *COL6A2* | PMID: 15054833 |
|  |  | *FN1* & *COL6A2* | PMID: 15054833 |
|  | 3893 & 4958 | *COL3A1* & *APOE* | PMID: 19424605 |
|  | 3893 & 6248 | *COL1A1* & *ELN* | PMID: 15054833 |
|  |  | *COL1A2* & *ELN* | PMID: 15054833 |
|  |  | *COL2A1* & *ELN* | PMID: 15054833 |
|  |  | *COL3A1* & *ELN* | PMID: 15054833 PMID: 12798348 |
|  |  | *FN1* & *ELN* | PMID: 15054833 |
|  |  | *FBN1* & *ELN* | PMID: 19850904 PMID: 12798348 |
|  | 3893 & 4294 |  |  |
|  | 4208 & 4945 | *F2* & *LDLR* | PMID: 14557872 |
|  |  | *THBS1* & *LDLR* | PMID: 14557872 |
|  | 4208 & 4199 | *MMP2* & *THBS2* | PMID: 19958327 |
|  |  | *MMP2* & *TIMP2* | PMID: 18935914 PMID: 17901377 PMID: 15561512 |
|  |  | *MMP2* & *TIMP3* | PMID: 17901377 |
|  |  | *MMP2* & *COL7A1* | PMID: 20194304 |
|  |  | *MMP13* & *TIMP4* | PMID: 19233360 |
|  | 4208 & 2292 | *MMP2* & *MMP3* | PMID: 19886850 PMID: 17901377 PMID: 11053050 |
|  |  | *MMP2* & *MMP1* | PMID: 19886850 PMID: 17901377 PMID: 15561512 |
|  |  | *MMP2* & *MMP7* | PMID: 19958327 |
|  |  | *MMP14* & *MMP3* | PMID: 19710627 PMID: 11053050 |
|  | 4208 & 4209 | *F2* & *C3* | PMID: 8142566 PMID: 1683788 |
|  | 4208 & 5928 |  |  |
|  | 4208 & 5345 |  |  |
|  | 4208 & 2437 |  |  |
|  | 4208 & 5916 | *F2* & *ATP1A1* | PMID: 12884521 PMID: 11564973 |
|  |  | *MMP2* & *SI* | PMID: 19713532 |
|  | 4208 & 3748 | *PLG* & *VWF* | PMID: 12783694 |
|  |  | *F2* & *F8* | PMID: 2314614 |
|  |  | *F2* & *F9* | PMID: 2314614 |
|  |  | *F2* & *KLK1* | PMID: 17460389 PMID: 15809361 PMID: 14623828 |
|  | 4208 & 4571 | *PLG* & *PROC* | PMID: 12783694 |
|  |  | *F2* & *F10* | PMID: 19387895 |
|  |  | *F2* & *F5* | PMID: 19415820 PMID: 18849403 PMID: 9038037 PMID: 8188063 PMID: 1363221 PMID: 2314614 |
|  |  | *F2* & *F7* | PMID: 19415820 PMID: 2314614 |
|  |  | *F2* & *CCL2* | PMID: 17460389 |
|  |  | *F2* & *AFP* | PMID: 2604661 |
|  |  | *MMP2* & *PROC* | PMID: 11053050 |
|  |  | *MMP14* & *PROC* | PMID: 11053050 |
|  | 4208 & 5316 | *PLAT* & *AGT* | PMID: 19779330 |
|  |  | *F2* & *AGT* | PMID: 15683714 |
|  |  | *F2* & *PRCP* | PMID: 14557872 |
|  |  | *THBS1* & *PRCP* | PMID: 14557872 |
|  | 4208 & 2280 |  |  |
|  | 4208 & 5497 | *MMP2* & *TIMP1* | PMID: 19958327 PMID: 15561512 |
|  | 4208 & 5920 | *F2* & *REST* | PMID: 15177518 PMID: 9253685 PMID: 8371917 PMID: 1949411 PMID: 3492363 PMID: 7077900 |
|  | 4208 & 6248 | *F2* & *SOD3* | PMID: 12047041 |
|  |  | *F2* & *CAMP* | PMID: 12745201 PMID: 11984003 PMID: 8080119 PMID: 1666433 PMID: 2156677 |
|  |  | *F2* & *SOD2* | PMID: 19592458 |
|  | 4208 & 4294 |  |  |
|  | 4208 & 6091 | *F2* & *CYBB* | PMID: 14504257 |
|  |  | *F2* & *NOX1* | PMID: 19592458 |
|  |  | *F2* & *DBP* | PMID: 17641813 PMID: 15683714 PMID: 15646031 PMID: 12775955 PMID: 12011644 PMID: 11393666 PMID: 11297649 |
|  |  | *F2* & *SAC* | PMID: 3194430 |
|  | 4945 & 4199 | *LRP1* & *THBS2* | PMID: 15121769 |
|  | 4945 & 4209 | *LDLR* & *C3* | PMID: 9878681 |
|  | 4945 & 5928 |  |  |
|  | 4945 & 2437 |  |  |
|  | 4945 & 5725 |  |  |
|  | 4945 & 3748 |  |  |
|  | 4945 & 4571 | *APP* & *GIF* | PMID: 20476671 |
|  |  | *LRP1* & *F7* | PMID: 15121769 |
|  |  | *LRP1* & *FGB* | PMID: 15121769 |
|  | 4945 & 5365 |  |  |
|  | 4945 & 6125 | *LDLR* & *CETP* | PMID: 12476935 |
|  | 4945 & 5934 |  |  |
|  | 4945 & 5600 | *APP* & *TG* | PMID: 15295718 |
|  |  | *LDLR* & *LIPC* | PMID: 14557872 PMID: 9878681 |
|  |  | *LDLR* & *APOB* | PMID: 18160459 PMID: 9878681 PMID: 9409302 |
|  | 4945 & 4847 | *APP* & *CLU* | PMID: 18723004 |
|  | 4945 & 5442 |  |  |
|  | 4945 & 4958 | *APP* & *APOE* | PMID: 16332384 PMID: 12404343 |
|  |  | *LRP1* & *APOE* | PMID: 15121769 |
|  |  | *LDLR* & *APOE* | PMID: 19252100 PMID: 18851860 PMID: 18160459 PMID: 17541027 PMID: 15261890 PMID: 11073835 PMID: 9878681 PMID: 9409302 |
|  | 4945 & 5689 |  |  |
|  | 4945 & 5368 |  |  |
|  | 4945 & 6091 | *APP* & *DBP* | PMID: 12874608 PMID: 11688765 |
|  |  | *LRP2* & *SAC* | PMID: 17989357 |
|  |  | *LDLR* & *APOA1* | PMID: 9878681 |
|  | 4945 & 5406 |  |  |
|  | 4907 & 2292 | *PECAM1* & *MMP3* | PMID: 19424605 |
|  |  | *PECAM1* & *MMP1* | PMID: 19424605 |
|  | 4907 & 5928 |  |  |
|  | 4907 & 5497 |  |  |
|  | 4907 & 5975 |  |  |
|  | 4907 & 4294 |  |  |
|  | 4199 & 2292 | *THBS2* & *MMP7* | PMID: 19958327 |
|  |  | *TIMP2* & *MMP3* | PMID: 17901377 |
|  |  | *TIMP2* & *MMP1* | PMID: 17901377 PMID: 15561512 |
|  |  | *TIMP3* & *MMP3* | PMID: 17901377 |
|  |  | *TIMP3* & *MMP1* | PMID: 17901377 |
|  | 4199 & 5316 | *THBS2* & *AGT* | PMID: 18600213 |
|  |  | *TIMP4* & *AGT* | PMID: 20332185 |
|  | 4199 & 5934 | *COL7A1* & *COL5A3* | PMID: 20194304 |
|  |  | *SERPINE1* & *CDA* | PMID: 19934566 |
|  | 4199 & 5600 | *KISS1* & *KISS1R* | PMID: 19228890 PMID: 16034182 PMID: 15769900 |
|  | 4199 & 6091 | *TMPRSS6* & *HFE* | PMID: 19862010 |
|  |  | *ST14* & *SPINT2* | PMID: 17338914 |
|  | 4891 & 5923 |  |  |
|  | 4891 & 5928 |  |  |
|  | 4891 & 6019 | *GNAS* & *GNB3* | PMID: 16600389 |
|  |  | *ADRBK1* & *PEBP1* | PMID: 14654844 |
|  | 4891 & 4224 |  |  |
|  | 4891 & 4832 | *GNAS* & *ADRA1B* | PMID: 10051321 |
|  |  | *ADRBK1* & *ADRA1B* | PMID: 18723764 |
|  | 4891 & 5475 |  |  |
|  | 4891 & 5931 | *ADRBK1* & *NOS3* | PMID: 16142243 |
|  | 4891 & 5368 |  |  |
|  | 4891 & 4655 |  |  |
|  | 4891 & 6361 | *ADRBK1* & *ADRBK2* | PMID: 14654844 |
|  | 5571 & 5708 |  |  |
|  | 4284 & 5928 |  |  |
|  | 4284 & 6019 |  |  |
|  | 4284 & 4950 |  |  |
|  | 4284 & 5833 |  |  |
|  | 4284 & 4224 |  |  |
|  | 4284 & 5708 |  |  |
|  | 4284 & 5975 |  |  |
|  | 2292 & 4571 | *MMP3* & *PROC* | PMID: 11053050 |
|  | 2292 & 5497 | *MMP1* & *TIMP1* | PMID: 15561512 |
|  |  | *MMP7* & *TIMP1* | PMID: 19958327 |
|  | 2292 & 5442 |  |  |
|  | 4209 & 4571 | *C3* & *CCL2* | PMID: 11463768 |
|  | 4209 & 5365 | *C4B* & *GC* | PMID: 17617859 |
|  | 4209 & 5475 | *C3* & *C1QB* | PMID: 16677633 |
|  | 4209 & 2880 |  |  |
|  | 4209 & 6091 | *C3* & *CFHR1* | PMID: 20202729 |
|  |  | *C3* & *DBP* | PMID: 19479107 PMID: 11518849 |
|  |  | *C3* & *APOA1* | PMID: 18801202 PMID: 14764618 PMID: 11798845 PMID: 9878681 |
|  |  | *CFHR3* & *CFHR1* | PMID: 20202729 |
|  |  | *CFH* & *CFHR1* | PMID: 20202729 |
|  | 5331 & 4709 | *TNF* & *SCD* | PMID: 19145821 PMID: 18245171 |
|  |  | *TNF* & *HBB* | PMID: 18245171 |
|  | 5331 & 2437 |  |  |
|  | 5331 & 5365 | *TNF* & *GC* | PMID: 20082712 PMID: 18234956 |
|  |  | *HP* & *GC* | PMID: 15002924 PMID: 2742764 |
|  | 5331 & 6181 |  |  |
|  | 5331 & 5689 | *TNF* & *HLA-B* | PMID: 20502044 |
|  | 5331 & 6248 | *TNF* & *IL1B* | PMID: 20808962 PMID: 19644155 PMID: 19347053 |
|  |  | *TNF* & *CAMP* | PMID: 15888553 PMID: 9514865 |
|  |  | *TNF* & *NPR1* | PMID: 20881240 PMID: 15710627 |
|  |  | *HP* & *CAMP* | PMID: 10778560 |
|  | 5331 & 4550 | *TNF* & *IFNGR1* | PMID: 20808962 |
|  | 5062 & 3905 | *PPARGC1A* & *CAD* | PMID: 18565194 |
|  | 5062 & 4186 | *PPARGC1A* & *UCP2* | PMID: 19762685 |
|  | 5062 & 5442 |  |  |
|  | 5707 & 5928 | *ADRB1* & *CAV1* | PMID: 17334644 |
|  | 5707 & 6019 | *ADRB1* & *GNB3* | PMID: 20537417 PMID: 20235788 PMID: 20144152 PMID: 15824464 PMID: 15174896 PMID: 15055253 |
|  |  | *ADRB1* & *ADRA2A* | PMID: 15824464 |
|  |  | *ADRB1* & *ADRA2B* | PMID: 18596718 PMID: 16907703 |
|  | 5707 & 5864 | *ADRB1* & *ADD1* | PMID: 19779464 PMID: 15824464 PMID: 15174896 PMID: 15055253 |
|  | 5707 & 5931 | *P2RX4* & *NOS1* | PMID: 15167446 |
|  |  | *ADRB1* & *NOS3* | PMID: 20537417 PMID: 15174896 |
|  | 5707 & 4550 |  |  |
|  | 3426 & 5928 |  |  |
|  | 3426 & 4847 |  |  |
|  | 3426 & 6248 | *ENG* & *GAN* | PMID: 18536821 |
|  | 5002 & 5916 |  |  |
|  | 3905 & 3583 | *TH* & *PIP* | PMID: 1209788 PMID: 4613617 |
|  |  | *CAD* & *PIP* | PMID: 6571002 |
|  | 3905 & 4709 | *CAD* & *CYP1A1* | PMID: 11996959 |
|  |  | *CAD* & *SCD* | PMID: 20888652 PMID: 20620740 PMID: 20443473 PMID: 19687159 PMID: 18269184 PMID: 15356432 PMID: 8989121 PMID: 8901759 |
|  |  | *CAD* & *CYP2C19* | PMID: 20460345 |
|  |  | *CAD* & *CYP2C9* | PMID: 21047199 PMID: 19615687 |
|  | 3905 & 6125 | *CAD* & *LPL* | PMID: 19876761 PMID: 19593950 PMID: 17895300 PMID: 15253101 PMID: 15135251 PMID: 14984018 PMID: 12419479 PMID: 11683775 PMID: 10484057 PMID: 9627528 |
|  |  | *CAD* & *CETP* | PMID: 19876761 PMID: 19784962 PMID: 19691831 PMID: 17587762 PMID: 15337694 |
|  | 3905 & 5975 | *TH* & *CPM* | PMID: 11780312 |
|  |  | *TH* & *ACE* | PMID: 21211411 PMID: 18405793 PMID: 17718243 PMID: 17715832 PMID: 15384026 PMID: 11465718 |
|  |  | *CAD* & *ACE* | PMID: 21265582 PMID: 21186110 PMID: 21180298 PMID: 21157371 PMID: 21104642 PMID: 20812878 PMID: 20799102 PMID: 20577898 PMID: 20223792 PMID: 20054766 PMID: 19878370 PMID: 19857679 PMID: 19847924 PMID: 19689620 PMID: 19379059 PMID: 19280995 PMID: 19203566 PMID: 19082699 PMID: 19043368 PMID: 19032136 PMID: 18991670 PMID: 18806522 PMID: 18759378 PMID: 18697380 PMID: 18637188 PMID: 18239395 PMID: 19337528 PMID: 17972547 PMID: 17917507 PMID: 17825750 PMID: 17487822 PMID: 17380191 PMID: 17223718 PMID: 17203791 PMID: 19847946 PMID: 19845074 PMID: 17193866 PMID: 17146542 PMID: 16905555 PMID: 16635753 PMID: 16512390 PMID: 16492065 PMID: 16451098 PMID: 16389160 PMID: 16168275 PMID: 16114984 PMID: 16061119 PMID: 15857519 PMID: 15857358 PMID: 15814871 PMID: 15754841 PMID: 15694743 PMID: 15631334 PMID: 15543563 PMID: 15261932 PMID: 14727978 PMID: 14680725 PMID: 12723895 PMID: 12669427 PMID: 12574792 PMID: 12394328 PMID: 12297007 PMID: 12010139 PMID: 11951494 PMID: 11759948 PMID: 11583891 PMID: 11416614 PMID: 11407110 PMID: 10781757 PMID: 10652909 PMID: 10608478 PMID: 10488959 PMID: 10488952 PMID: 10440157 PMID: 10370397 PMID: 10359862 PMID: 10028948 PMID: 9832341 PMID: 9822117 PMID: 9769888 PMID: 9736438 PMID: 9693941 PMID: 9667803 PMID: 9605599 PMID: 9591757 PMID: 9587045 PMID: 9443774 PMID: 9409318 PMID: 9388047 PMID: 9264004 PMID: 9215330 PMID: 8558760 PMID: 7955173 PMID: 7952407 PMID: 8061851 PMID: 8357335 PMID: 7508054 PMID: 1575175 PMID: 1671190 |
|  | 4926 & 5708 |  |  |
|  | 4926 & 3485 |  |  |
|  | 5923 & 5832 | *EGF* & *NOS2A* | PMID: 15994432 |
|  |  | *RGS4* & *GEFT* | PMID: 19689474 |
|  | 5923 & 4571 |  |  |
|  | 5923 & 5316 | *AGTR2* & *AGT* | PMID: 17380887 PMID: 17261659 PMID: 15492316 |
|  | 5923 & 5975 | *EGF* & *CPM* | PMID: 1524873 |
|  |  | *EGF* & *ACE* | PMID: 11116050 |
|  |  | *AGTR2* & *ACE* | PMID: 17380887 PMID: 15492316 PMID: 10652034 |
|  | 5928 & 5832 |  |  |
|  | 5928 & 5725 |  |  |
|  | 5928 & 5931 | *CAV1* & *NOS3* | PMID: 20382348 PMID: 19487814 PMID: 18022214 PMID: 16601841 |
|  |  | *CAV1* & *CAV3* | PMID: 19110506 PMID: 15306231 |
|  | 5928 & 4550 |  |  |
|  | 3477 & 5316 | *NTS* & *AGT* | PMID: 16650497 PMID: 8267622 |
|  |  | *NTS* & *ACE2* | PMID: 18356558 |
|  | 3477 & 5475 |  |  |
|  | 3477 & 6361 |  |  |
|  | 5345 & 2437 |  |  |
|  | 5345 & 5365 | *WNK4* & *SLC12A3* | PMID: 21157372 PMID: 20921400 PMID: 19491230 PMID: 19470686 PMID: 18547946 PMID: 17885550 PMID: 15824464 PMID: 15309683 PMID: 14608358 PMID: 12671053 |
|  |  | *SLC12A1* & *SLC12A3* | PMID: 21157372 PMID: 18391953 PMID: 17275579 PMID: 14596636 |
|  |  | *STK39* & *SLC12A3* | PMID: 20889219 PMID: 20091762 PMID: 19470686 |
|  | 5345 & 6181 |  |  |
|  | 5203 & 6019 |  |  |
|  | 5203 & 5797 |  |  |
|  | 5203 & 2880 |  |  |
|  | 3583 & 5234 | *GCK* & *GCKR* | PMID: 19651812 |
|  | 4445 & 4709 |  |  |
|  | 4445 & 5975 | *SCNN1A* & *SCNN1D* | PMID: 18298571 |
|  |  | *SCNN1A* & *ACE* | PMID: 20577119 PMID: 15699455 PMID: 14596636 |
|  |  | *HMOX1* & *ACE* | PMID: 15085064 |
|  | 4445 & 5931 | *SCNN1A* & *NOS3* | PMID: 15699455 |
|  |  | *HMOX1* & *NOS3* | PMID: 18633193 PMID: 15699468 PMID: 12720199 PMID: 12227681 PMID: 11440978 |
|  | 4709 & 5975 | *CYP1A1* & *COMT* | PMID: 12624000 PMID: 8930523 |
|  |  | *CYP1A1* & *ACE* | PMID: 17436021 |
|  |  | *SCD* & *ACE* | PMID: 19113795 PMID: 16480770 |
|  |  | *CYP1A2* & *COMT* | PMID: 19999796 PMID: 12624000 |
|  |  | *CYP2E1* & *ACE* | PMID: 17436021 |
|  | 5327 & 2437 |  |  |
|  | 5327 & 5934 |  |  |
|  | 5327 & 5704 |  |  |
|  | 5327 & 3485 |  |  |
|  | 5327 & 6091 |  |  |
|  | 6019 & 2437 | *GNB3* & *PON1* | PMID: 19254215 |
|  | 6019 & 6125 | *GNB3* & *LPL* | PMID: 20585107 PMID: 19254215 |
|  |  | *GNB3* & *CETP* | PMID: 17785925 |
|  | 6019 & 5920 | *GNB3* & *REST* | PMID: 16002097 PMID: 15831363 PMID: 12890290 PMID: 11875193 |
|  | 2437 & 4950 |  |  |
|  | 2437 & 4847 |  |  |
|  | 2437 & 5975 | *PON1* & *ACE* | PMID: 21044781 PMID: 19535833 PMID: 19280995 PMID: 19254215 |
|  | 2437 & 2880 |  |  |
|  | 2437 & 6091 | *PON1* & *DBP* |  |
|  |  | *PON1* & *APOA1* |  |
|  |  | *PRLHR* & *DBP* | PMID: 14691196 |
|  | 4950 & 5234 |  |  |
|  | 4950 & 5975 | *NR3C2* & *ACE* | PMID: 15824464 PMID: 14596636 |
|  | 4950 & 4550 |  |  |
|  | 4950 & 4655 | *NFKBIZ* & *DUSP1* | PMID: 20144878 |
|  | 4950 & 5406 |  |  |
|  | 5916 & 5708 |  |  |
|  | 5916 & 5864 | *SI* & *ADD1* | PMID: 17189961 |
|  | 5725 & 5475 |  |  |
|  | 5725 & 6248 | *NPPA* & *CAMP* | PMID: 2567705 |
|  | 6103 & 3485 |  |  |
|  | 5833 & 4186 |  |  |
|  | 5833 & 2880 |  |  |
|  | 3748 & 4571 | *VWF* & *F5* | PMID: 17137217 |
|  |  | *VWF* & *PROC* | PMID: 12783694 |
|  |  | *VWF* & *THBD* | PMID: 20144070 |
|  |  | *F8* & *F5* | PMID: 16535967 PMID: 2314614 |
|  |  | *F8* & *F7* | PMID: 16535967 PMID: 2314614 |
|  |  | *F9* & *F5* | PMID: 2314614 |
|  |  | *F9* & *F7* | PMID: 2314614 |
|  |  | *KLK1* & *CCL2* | PMID: 17460389 |
|  | 4571 & 5920 | *FGB* & *REST* | PMID: 19799197 |
|  | 4571 & 2923 | *CCL2* & *CCR2* | PMID: 19730125 PMID: 19506371 PMID: 19420107 PMID: 17938380 PMID: 17922026 PMID: 17823354 PMID: 17604287 PMID: 17417600 PMID: 17003237 PMID: 16794480 PMID: 15262905 PMID: 15059935 PMID: 14597759 PMID: 12411463 |
|  | 5316 & 4224 | *AGT* & *ADRB2* | PMID: 20537417 PMID: 16615274 PMID: 15824464 PMID: 15699455 PMID: 11071381 |
|  |  | *AGT* & *AGTR1* | PMID: 20816596 PMID: 20811292 PMID: 20537417 PMID: 20027122 PMID: 19779330 PMID: 19341158 PMID: 19332265 PMID: 19014923 PMID: 18698212 PMID: 18563171 PMID: 18049108 PMID: 17546276 PMID: 17380887 PMID: 17261659 PMID: 16790149 PMID: 16061119 PMID: 15934435 PMID: 15824464 PMID: 15683714 PMID: 15492316 PMID: 15174896 PMID: 12695419 PMID: 12556231 PMID: 11208365 PMID: 11013071 |
|  | 5316 & 5442 |  |  |
|  | 5316 & 3622 |  |  |
|  | 4186 & 5931 |  |  |
|  | 5365 & 5406 | *GC* & *PON2* | PMID: 17137217 |
|  | 5234 & 5797 |  |  |
|  | 5797 & 5708 |  |  |
|  | 5797 & 6091 |  |  |
|  | 6125 & 5931 | *LPL* & *NOS3* | PMID: 19254215 PMID: 16369102 |
|  |  | *CETP* & *NOS3* | PMID: 19691831 PMID: 18956684 |
|  | 6125 & 6091 | *LPL* & *DBP* | PMID: 16378107 PMID: 15253101 PMID: 15127290 PMID: 12862202 |
|  |  | *LPL* & *APOA1* | PMID: 11798845 |
|  |  | *LPL* & *SST* | PMID: 16430904 |
|  |  | *CETP* & *SST* | PMID: 16430904 |
|  | 4224 & 5975 | *ADRB2* & *ACE* | PMID: 21044781 PMID: 20537417 PMID: 16615274 PMID: 15824464 PMID: 15699455 PMID: 12394950 |
|  |  | *AGTR1* & *COMT* | PMID: 15174896 |
|  |  | *AGTR1* & *ACE* | PMID: 20816596 PMID: 20537417 PMID: 20027122 PMID: 19463113 PMID: 19341158 PMID: 19332265 PMID: 19014923 PMID: 18698212 PMID: 18563171 PMID: 18347611 PMID: 18049108 PMID: 17546276 PMID: 17519002 PMID: 17380887 PMID: 17097490 PMID: 16061119 PMID: 15934435 PMID: 15863668 PMID: 15824464 PMID: 15683714 PMID: 15612584 PMID: 15545843 PMID: 15492316 PMID: 15332573 PMID: 15174896 PMID: 12975417 PMID: 12695419 PMID: 12556231 PMID: 12394950 PMID: 11246471 PMID: 11208365 PMID: 9456365 PMID: 8772690 |
|  |  | *BDKRB2* & *ACE* | PMID: 15894833 PMID: 12848919 |
|  | 4224 & 6248 |  |  |
|  | 4224 & 5931 | *ADRB2* & *NOS3* | PMID: 20537417 PMID: 19373110 PMID: 15699455 |
|  |  | *AGTR1* & *NOS3* | PMID: 20537417 PMID: 19779330 PMID: 18698212 PMID: 16109907 PMID: 15174896 |
|  |  | *BDKRB2* & *NOS3* | PMID: 20944660 |
|  | 4224 & 3470 | *ADRB2* & *GRK4* | PMID: 20537417 PMID: 16636198 |
|  |  | *AGTR1* & *GRK4* | PMID: 20537417 PMID: 16636198 |
|  | 4224 & 6361 | *BDKRB2* & *BDKRB1* | PMID: 15643125 |
|  | 4832 & 5931 |  |  |
|  | 4832 & 6091 |  |  |
|  | 5934 & 5975 | *POLI* & *ACE* | PMID: 2234477 |
|  |  | *MIP* & *ACE* | PMID: 12090726 PMID: 11882625 PMID: 11727501 PMID: 10983731 |
|  |  | *CDA* & *ACE* | PMID: 10359862 |
|  | 5600 & 4847 |  |  |
|  | 5600 & 5920 | *TG* & *REST* | PMID: 19877546 PMID: 19605369 PMID: 19568708 PMID: 19459560 PMID: 18655751 PMID: 11586489 PMID: 10582554 PMID: 7601202 PMID: 7723358 PMID: 1410527 PMID: 2021964 PMID: 2143586 |
|  | 5600 & 6181 | *LIPC* & *KCNJ11* | PMID: 17137217 |
|  |  | *APOB* & *KCNJ11* | PMID: 17137217 |
|  | 5600 & 6091 | *CTSA* & *COL14A1* | PMID: 19772500 |
|  |  | *LIPC* & *APOA1* | PMID: 9878681 |
|  |  | *APOB* & *RBP4* | PMID: 20376890 |
|  |  | *APOB* & *DBP* | PMID: 18855268 PMID: 17729055 PMID: 15061989 PMID: 11378831 PMID: 10423656 PMID: 10090111 |
|  |  | *APOB* & *APOA1* | PMID: 20538481 PMID: 20495830 PMID: 19648057 PMID: 19476111 PMID: 19264209 PMID: 18801202 PMID: 18774540 PMID: 18277343 PMID: 18202666 PMID: 17587723 PMID: 17525004 PMID: 17389257 PMID: 17217166 PMID: 16898503 PMID: 16565870 PMID: 16313772 PMID: 15859024 PMID: 15654296 PMID: 15646744 PMID: 15364185 PMID: 14985310 PMID: 14669600 PMID: 12911531 PMID: 11323443 PMID: 10073983 PMID: 10073988 PMID: 9878681 PMID: 9702430 PMID: 9592639 PMID: 9237284 PMID: 8927338 PMID: 9455355 PMID: 7500552 PMID: 7695175 PMID: 8068899 PMID: 8364758 PMID: 2128269 |
|  |  | *TG* & *CYBB* | PMID: 20833959 |
|  |  | *TG* & *NOX1* | PMID: 20639222 PMID: 17494996 |
|  |  | *TG* & *DBP* | PMID: 21215143 PMID: 21033608 PMID: 20654100 PMID: 20506660 PMID: 20450544 PMID: 20235359 PMID: 20075928 PMID: 20075425 PMID: 19957617 PMID: 19708000 PMID: 19555395 PMID: 19376375 PMID: 19239788 PMID: 19176866 PMID: 19118911 PMID: 19025446 PMID: 19006028 PMID: 19003544 PMID: 18706730 PMID: 18662594 PMID: 18642663 PMID: 18360017 PMID: 18206038 PMID: 18044341 PMID: 17963628 PMID: 17729055 PMID: 17617283 PMID: 17597234 PMID: 17484208 PMID: 17450262 PMID: 17299959 PMID: 16970091 PMID: 16936988 PMID: 16378107 PMID: 16238190 PMID: 16188067 PMID: 15924790 PMID: 15779578 PMID: 15353873 PMID: 15019600 PMID: 14689853 PMID: 14644403 PMID: 14566090 PMID: 14535102 PMID: 12905810 PMID: 12862202 PMID: 12860491 PMID: 12800096 PMID: 12785026 PMID: 12699129 PMID: 12406036 PMID: 12355867 PMID: 12225719 PMID: 11872689 PMID: 11715454 PMID: 11443506 PMID: 11427787 PMID: 11294029 PMID: 11096265 PMID: 10948082 PMID: 10673137 PMID: 11126342 PMID: 10423656 PMID: 10390951 PMID: 8843176 PMID: 8872845 PMID: 8713678 PMID: 7780864 PMID: 7718026 PMID: 7807497 PMID: 8021907 PMID: 7805525 PMID: 8432415 PMID: 8123709 PMID: 1424997 PMID: 2383317 PMID: 2737688 PMID: 2879464 PMID: 7104157 |
|  |  | *TG* & *ALB* | PMID: 19568708 PMID: 18688083 PMID: 17633356 |
|  |  | *TG* & *APOA1* | PMID: 21033077 PMID: 19476111 PMID: 18801202 PMID: 18774540 PMID: 18202666 PMID: 17587723 PMID: 16313772 PMID: 14669600 PMID: 8927338 |
|  | 5475 & 5497 |  |  |
|  | 5475 & 4860 | *ECE1* & *EDN1* | PMID: 17664854 PMID: 17525706 |
|  | 5497 & 5931 |  |  |
|  | 5497 & 3622 |  |  |
|  | 4847 & 6319 |  |  |
|  | 5920 & 6181 |  |  |
|  | 5920 & 6361 | *REST* & *TERT* | PMID: 42418 |
|  |  | *REST* & *ADRBK2* | PMID: 11901213 |
|  | 5920 & 6091 | *REST* & *DBP* | PMID: 21233799 PMID: 21098871 PMID: 20574413 PMID: 20377147 PMID: 20137406 PMID: 20087215 PMID: 20044740 PMID: 19943716 PMID: 19564246 PMID: 19499963 PMID: 19360246 PMID: 19330562 PMID: 19142373 PMID: 18927524 PMID: 18544158 PMID: 18444532 PMID: 18277829 PMID: 18256574 PMID: 18096293 PMID: 18075809 PMID: 17635183 PMID: 17578748 PMID: 17560080 PMID: 17341993 PMID: 17163243 PMID: 16814126 PMID: 16468060 PMID: 16231755 PMID: 16075802 PMID: 15716693 PMID: 15576254 PMID: 15505116 PMID: 15467328 PMID: 15201859 PMID: 15199310 PMID: 15021072 PMID: 14763666 PMID: 14653055 PMID: 14596360 PMID: 14575290 PMID: 14572682 PMID: 12887823 PMID: 12819559 PMID: 12781081 PMID: 12574786 PMID: 12552248 PMID: 12523679 PMID: 12512233 PMID: 12474774 PMID: 12473870 PMID: 12370842 PMID: 12271101 PMID: 12172311 PMID: 12131561 PMID: 11913624 PMID: 11880561 PMID: 11875193 PMID: 11768726 PMID: 11581545 PMID: 11494096 PMID: 11403349 PMID: 11336181 PMID: 11317190 PMID: 11014084 PMID: 10999643 PMID: 10904026 PMID: 10764125 PMID: 10736939 PMID: 10723119 PMID: 10534606 PMID: 10466461 PMID: 10455474 PMID: 10421088 PMID: 10413065 PMID: 10334439 PMID: 10028925 PMID: 9883709 PMID: 9822945 PMID: 9683044 PMID: 9624637 PMID: 9607390 PMID: 9597385 PMID: 9560868 PMID: 9360028 PMID: 9128792 PMID: 9049755 PMID: 9140781 PMID: 8904726 PMID: 8872831 PMID: 8746641 PMID: 8771248 PMID: 7591020 PMID: 8575111 PMID: 8572882 PMID: 7631884 PMID: 7496560 PMID: 7482386 PMID: 7548301 PMID: 7623369 PMID: 11847547 PMID: 7783096 PMID: 7759855 PMID: 7666676 PMID: 7805070 PMID: 7755462 PMID: 7755452 PMID: 7813693 PMID: 8147945 PMID: 7511751 PMID: 16250794 PMID: 8205302 PMID: 7907871 PMID: 8136093 PMID: 8310885 PMID: 8345488 PMID: 8101194 PMID: 8475231 PMID: 1505564 PMID: 1410883 PMID: 1387043 PMID: 1378112 PMID: 1559531 PMID: 1820438 PMID: 1817759 PMID: 1763923 PMID: 1779513 PMID: 2041034 PMID: 1889207 PMID: 1824663 PMID: 1799687 PMID: 1723451 PMID: 1687786 PMID: 2081999 PMID: 2174943 PMID: 2162875 PMID: 11527124 PMID: 2130241 PMID: 2093744 PMID: 2700113 PMID: 2512596 PMID: 2807362 PMID: 2772104 PMID: 2672065 PMID: 2510660 PMID: 2522905 PMID: 2700322 PMID: 2562912 PMID: 3066270 PMID: 3067303 PMID: 3236317 PMID: 3236315 PMID: 3280191 PMID: 3243219 PMID: 3692569 PMID: 3506628 PMID: 3453398 PMID: 3495163 PMID: 2485056 PMID: 2434787 PMID: 3781678 PMID: 2945940 PMID: 3757989 PMID: 2879477 PMID: 2439780 PMID: 4043100 PMID: 3895793 PMID: 6405711 PMID: 6336719 PMID: 7152630 PMID: 7461795 PMID: 7461791 PMID: 6997978 PMID: 7346736 PMID: 1266661 PMID: 1251770 |
|  |  | *REST* & *ALB* | PMID: 19568708 |
|  |  | *REST* & *CORT* | PMID: 8951977 |
|  |  | *REST* & *SAC* | PMID: 10366162 |
|  | 6181 & 5442 |  |  |
|  | 6181 & 5708 |  |  |
|  | 6181 & 5864 |  |  |
|  | 6181 & 3622 |  |  |
|  | 6181 & 6361 |  |  |
|  | 5442 & 6319 |  |  |
|  | 5442 & 6258 | *AVP* & *REN* | PMID: 8092324 PMID: 1288975 |
|  | 5442 & 3622 | *AVP* & *NPY* | PMID: 1835195 |
|  |  | *AVP* & *PYY* | PMID: 1572330 |
|  | 5708 & 5975 |  |  |
|  | 5708 & 6248 |  |  |
|  | 6319 & 6091 | *LEP* & *DBP* | PMID: 19562039 PMID: 19382181 PMID: 11872689 |
|  | 5975 & 5864 | *COMT* & *ADD1* | PMID: 15174896 |
|  |  | *ACE* & *ADD1* | PMID: 20712239 PMID: 19443911 PMID: 19103106 PMID: 18800139 PMID: 17452507 PMID: 16615274 PMID: 16612256 PMID: 15824464 PMID: 15699455 PMID: 15699449 PMID: 15174896 PMID: 12885793 PMID: 12394950 |
|  |  | *ACE* & *ADD2* | PMID: 15699449 |
|  |  | *ACE* & *CD34* | PMID: 18426993 |
|  | 5864 & 4958 | *LIPE* & *APOE* | PMID: 17318300 |
|  | 5864 & 6361 |  |  |
|  | 4958 & 6091 | *APOE* & *CYBA* | PMID: 19853876 |
|  |  | *APOE* & *NOX1* | PMID: 19881226 |
|  |  | *APOE* & *DBP* | PMID: 18855268 |
|  |  | *APOE* & *APOA1* | PMID: 21033077 PMID: 19644050 PMID: 15320783 PMID: 14986172 PMID: 12118912 PMID: 10073988 PMID: 9878681 |
|  |  | *APOE* & *SCARB1* | PMID: 19644050 |
|  | 6248 & 6091 | *SOD3* & *CYBA* | PMID: 15598867 |
|  |  | *SOD3* & *NOX1* | PMID: 17762156 |
|  |  | *ELN* & *DBP* | PMID: 19282817 |
|  |  | *CAMP* & *DBP* | PMID: 9283060 |
|  |  | *SOD2* & *NOX1* | PMID: 19592458 |
|  |  | *GAN* & *DBP* | PMID: 20852445 PMID: 15001821 PMID: 12905810 PMID: 12862202 |
|  | 4860 & 3485 | *EDN1* & *ROCK2* | PMID: 16906334 |
|  | 4860 & 5931 | *EDN1* & *NOS3* | PMID: 20537417 PMID: 19254215 PMID: 15699938 |
|  |  | *EDN1* & *EDNRA* | PMID: 17525706 PMID: 17016617 |
|  |  | *EDN1* & *EDNRB* |  |
|  | 5931 & 5368 |  |  |
|  | 4550 & 6091 | *GHR* & *DBP* | PMID: 11966735 |
|  |  | *GHR* & *ALB* | PMID: 15334695 |
|  | 4655 & 5441 |  |  |
|  | 4655 & 5406 |  |  |
