# Supplementary material for: Disease Gene Interaction Pathways: A Potential Framework for How Disease Genes Associate by Disease-Risk Modules
Source: PLoS One. 2011 Sep 6;6(9):e24495. doi: 10.1371/journal.pone.0024495 (PMC3167857; doi:10.1371/journal.pone.0024495)
Supplement: Table S7 — PubMed ID in which KEGG pathways enriched have been proved to be correlated with T2D. (DOC) [file pone.0024495.s010.doc]

**Table S7. PubMed ID in which KEGG pathways enriched have been proved to be correlated with T2D.**

| **pathway** | **Pubmed ID associated with HT** |
| --- | --- |
| ABC transporters | PMID: 18490524 PMID: 16897043 PMID: 16723355 PMID: 16282967 |
| Acute myeloid leukemia | PMID: 16105753 PMID: 14530879 PMID: 11025599 |
| Adherens junction | PMID: 19254713 |
| Adipocytokine signaling pathway | PMID: 20739398 PMID: 20536390 PMID: 20442402 PMID: 19727662 PMID: 17467106 PMID: 17340225 PMID: 16735449 PMID: 15604363 |
| Aldosterone-regulated sodium reabsorption |  |
| Allograft rejection | PMID: 20402545 PMID: 20150806 PMID: 19911164 PMID: 19307791 PMID: 18535153 PMID: 18405244 PMID: 17922735 PMID: 16830364 PMID: 16556164 PMID: 15803926 PMID: 15777209 PMID: 15691225 PMID: 15689368 PMID: 15378803 PMID: 15331537 PMID: 15194406 PMID: 15194405 PMID: 14698000 PMID: 12866997 PMID: 12857987 PMID: 12727648 PMID: 12520519 PMID: 12482142 PMID: 11872651 PMID: 11455247 PMID: 11375072 PMID: 11038640 PMID: 10830215 PMID: 9849976 PMID: 9647438 PMID: 9112346 PMID: 9352601 PMID: 9163857 PMID: 7607599 PMID: 8178349 PMID: 7858116 PMID: 2642829 PMID: 2432705 PMID: 136874 |
| alpha-Linolenic acid metabolism |  |
| Alzheimer's disease | PMID: 21252113 PMID: 21250986 PMID: 21225619 PMID: 21222567 PMID: 21219420 PMID: 21212741 PMID: 21172066 PMID: 21170561 PMID: 21138275 PMID: 21129330 PMID: 21112383 PMID: 21094907 PMID: 21067307 PMID: 20974195 PMID: 20970452 PMID: 20951129 PMID: 20945041 PMID: 20926102 PMID: 20925423 PMID: 20888602 PMID: 20878582 PMID: 20873820 PMID: 20872319 PMID: 20847403 PMID: 20829391 PMID: 20798963 PMID: 20797821 PMID: 20725514 PMID: 20716961 PMID: 20703363 PMID: 20698813 PMID: 20655383 PMID: 20651283 PMID: 20579740 PMID: 20556444 PMID: 20552420 PMID: 20542701 PMID: 20541662 PMID: 20535861 PMID: 20533901 PMID: 20513336 PMID: 20504892 PMID: 20493414 PMID: 20493190 PMID: 20490276 PMID: 20476794 PMID: 20466032 PMID: 20454453 PMID: 20413862 PMID: 20385830 PMID: 20337586 PMID: 20303720 PMID: 20186753 PMID: 20182043 PMID: 20152041 PMID: 20151005 PMID: 20139653 PMID: 20132476 PMID: 20123114 PMID: 20061608 PMID: 20042103 PMID: 20035739 PMID: 20034403 PMID: 19941173 PMID: 19917634 PMID: 19885299 PMID: 19848310 PMID: 19781557 PMID: 19758471 PMID: 19748969 PMID: 19742390 PMID: 19735971 PMID: 19732809 PMID: 19721899 PMID: 19712582 PMID: 19705099 PMID: 19683583 PMID: 19659459 PMID: 19623253 PMID: 19622391 PMID: 19616957 PMID: 19614577 PMID: 19614573 PMID: 19585955 PMID: 19573562 PMID: 19572104 PMID: 19519303 PMID: 19504347 PMID: 19492079 PMID: 19490145 PMID: 19485934 PMID: 19485707 PMID: 19453298 PMID: 19440546 PMID: 19402773 PMID: 19387119 PMID: 19387114 PMID: 19387113 PMID: 19387106 PMID: 19383491 PMID: 19376202 PMID: 19353221 PMID: 19286253 PMID: 19281826 PMID: 19266159 PMID: 19243006 PMID: 19237574 PMID: 19198769 PMID: 19196369 PMID: 19196246 PMID: 19188609 PMID: 19154729 PMID: 20387270 PMID: 19107993 PMID: 19079806 PMID: 19065799 PMID: 19026743 PMID: 19021543 PMID: 19021537 PMID: 19015532 PMID: 19013138 PMID: 18930564 PMID: 18836752 PMID: 18789386 PMID: 18786878 PMID: 18780965 PMID: 18761467 PMID: 18755632 PMID: 18697612 PMID: 18673219 PMID: 18667359 PMID: 18627032 PMID: 18622365 PMID: 18621727 PMID: 18598780 PMID: 18590347 PMID: 18537546 PMID: 18487142 PMID: 18486611 PMID: 18483616 PMID: 18457428 PMID: 18410739 PMID: 18294929 PMID: 18291688 PMID: 18261472 PMID: 18251521 PMID: 18234983 PMID: 18221107 PMID: 18198460 PMID: 18049445 PMID: 17968435 PMID: 18024186 PMID: 17980978 PMID: 17979302 PMID: 17924651 PMID: 17912358 PMID: 17764626 PMID: 19885123 PMID: 17592527 PMID: 17553629 PMID: 17545740 PMID: 17544131 PMID: 17493579 PMID: 17487340 PMID: 17456849 PMID: 17430240 PMID: 17430239 PMID: 17420320 PMID: 17374166 PMID: 17316694 PMID: 17297730 PMID: 17211725 PMID: 17210813 PMID: 17151862 PMID: 17110895 PMID: 17096857 PMID: 17052186 PMID: 17045417 PMID: 16979422 PMID: 16932542 PMID: 16886098 PMID: 16803995 PMID: 16774767 PMID: 16759099 PMID: 16706683 PMID: 16671891 PMID: 16631207 PMID: 16611073 PMID: 16574064 PMID: 16505506 PMID: 16484636 PMID: 16481120 PMID: 16464680 PMID: 16444902 PMID: 16412093 PMID: 16399206 PMID: 16396227 PMID: 16387690 PMID: 16340084 PMID: 16340082 PMID: 16330295 PMID: 16306348 PMID: 16236384 PMID: 16186174 PMID: 16103997 PMID: 16048553 PMID: 15974903 PMID: 15942449 PMID: 15935563 PMID: 15933386 PMID: 15850385 PMID: 15843464 PMID: 15794122 PMID: 15784251 PMID: 15750214 PMID: 15659827 PMID: 15639310 PMID: 15590460 PMID: 15576053 PMID: 15542273 PMID: 15533050 PMID: 15530996 PMID: 15285797 PMID: 15283924 PMID: 15270203 PMID: 15258737 PMID: 15184599 PMID: 15181020 PMID: 15102433 PMID: 15094077 PMID: 15078145 PMID: 15039230 PMID: 15033922 PMID: 14976144 PMID: 14730686 PMID: 14710446 PMID: 14702136 PMID: 14702004 PMID: 14595538 PMID: 12928438 PMID: 12847226 PMID: 12843569 PMID: 12806897 PMID: 12749025 PMID: 12725709 PMID: 12716786 PMID: 12716134 PMID: 12706318 PMID: 12637985 PMID: 12558712 PMID: 12517724 PMID: 12515900 PMID: 12475085 PMID: 12431815 PMID: 12420649 PMID: 12389706 PMID: 12183643 PMID: 12110243 PMID: 12015594 PMID: 12011802 PMID: 11916953 PMID: 11882522 PMID: 11879773 PMID: 11856825 PMID: 11752480 PMID: 11732264 PMID: 11570983 PMID: 11516996 PMID: 11376469 PMID: 11136994 PMID: 11124436 PMID: 10911966 PMID: 10903851 PMID: 10799482 PMID: 10229302 PMID: 10081287 PMID: 9767914 PMID: 9654911 PMID: 9462471 PMID: 9341206 PMID: 9038143 PMID: 8743979 PMID: 7595053 PMID: 8152488 PMID: 8366714 PMID: 1742146 |
| Amino sugar and nucleotide sugar metabolism | PMID: 20555325 PMID: 19440374 PMID: 17681543 PMID: 17097593 PMID: 16882729 PMID: 15472217 PMID: 14523042 PMID: 12721503 PMID: 12678487 PMID: 12220542 PMID: 11868613 PMID: 11461192 PMID: 10623569 PMID: 10531330 PMID: 14172871 |
| Aminoacyl-tRNA biosynthesis | PMID: 11729521 PMID: 11334437 |
| Amyotrophic lateral sclerosis (ALS) | PMID: 16856151 PMID: 14704796 |
| Antigen processing and presentation | PMID: 20346437 PMID: 19199708 PMID: 18353353 PMID: 18078524 PMID: 16212883 PMID: 11544345 PMID: 10051633 PMID: 9202073 |
| Apoptosis | PMID: 21254394 PMID: 21252113 PMID: 21251764 PMID: 21251686 PMID: 21248143 PMID: 21245056 PMID: 21240658 PMID: 21235725 PMID: 21233852 PMID: 21233843 PMID: 21227528 PMID: 21209957 PMID: 21196169 PMID: 21193743 PMID: 21190961 PMID: 21167294 PMID: 21163363 PMID: 21161439 PMID: 21153483 PMID: 21150342 PMID: 21146390 PMID: 21113299 PMID: 21104267 PMID: 21099312 PMID: 21099287 PMID: 21099280 PMID: 21094907 PMID: 21087777 PMID: 21073655 PMID: 21070766 PMID: 21067307 PMID: 21060752 PMID: 21057731 PMID: 21053274 PMID: 21047942 PMID: 21029312 PMID: 21029303 PMID: 21029302 PMID: 21029296 PMID: 21044045 PMID: 21042774 PMID: 21039588 PMID: 20980462 PMID: 20978346 PMID: 20969475 PMID: 20962845 PMID: 20957341 PMID: 20956533 PMID: 20953387 PMID: 20952489 PMID: 20941661 PMID: 20939194 PMID: 20939106 PMID: 20926102 PMID: 20923823 PMID: 20872961 PMID: 20864282 PMID: 20855893 PMID: 20854178 PMID: 20850033 PMID: 20840078 PMID: 20839851 PMID: 20833797 PMID: 20829391 PMID: 20823566 PMID: 20809534 PMID: 20807334 PMID: 20795946 PMID: 20735358 PMID: 20706996 PMID: 20704278 PMID: 20703435 PMID: 20693577 PMID: 20687985 PMID: 20683626 PMID: 20666272 PMID: 20660625 PMID: 20660595 PMID: 20651283 PMID: 20649634 PMID: 20644627 PMID: 20638688 PMID: 20634940 PMID: 20628728 PMID: 20619299 PMID: 20585581 PMID: 20580385 PMID: 20570597 PMID: 20554742 PMID: 20541363 PMID: 20541219 PMID: 20534453 PMID: 20532833 PMID: 20532811 PMID: 20519332 PMID: 20493839 PMID: 20492683 PMID: 20490276 PMID: 20489606 PMID: 20474066 PMID: 20463468 PMID: 20460909 PMID: 20455996 PMID: 20442404 PMID: 20441053 PMID: 20424162 PMID: 20421300 PMID: 20419448 PMID: 20418481 PMID: 20412748 PMID: 20412743 PMID: 20411998 PMID: 20410100 PMID: 20404103 PMID: 20401454 PMID: 20400513 PMID: 20392809 PMID: 20382683 PMID: 20380516 PMID: 20376052 PMID: 20374643 PMID: 20374430 PMID: 20370677 PMID: 20370652 PMID: 20369225 PMID: 20357380 PMID: 20353585 PMID: 20346323 PMID: 20332197 PMID: 20305008 PMID: 20299332 PMID: 20233722 PMID: 20233282 PMID: 20227390 PMID: 20225396 PMID: 20224724 PMID: 20222158 PMID: 20217513 PMID: 20217512 PMID: 20217509 PMID: 20216988 PMID: 20215707 PMID: 20215397 PMID: 20200776 PMID: 20188823 PMID: 20185813 PMID: 20185809 PMID: 20182043 PMID: 20176723 PMID: 20176718 PMID: 20162295 PMID: 20159982 PMID: 20150295 PMID: 20122988 PMID: 20117255 PMID: 20115936 PMID: 20107108 PMID: 20105289 PMID: 20104262 PMID: 20098273 PMID: 20085125 PMID: 20083151 PMID: 20079380 PMID: 20071694 PMID: 20067833 PMID: 20062967 PMID: 20061378 PMID: 20056169 PMID: 20053495 PMID: 20044906 PMID: 20043885 PMID: 20042670 PMID: 20041964 PMID: 20040361 PMID: 20038265 PMID: 20037497 PMID: 20028968 PMID: 19996363 PMID: 19959758 PMID: 19958109 PMID: 19957848 PMID: 19947945 PMID: 19940263 PMID: 19938874 PMID: 19934005 PMID: 19925395 PMID: 19917066 PMID: 19915011 PMID: 19902175 PMID: 19881493 PMID: 19878257 PMID: 19876772 PMID: 19874568 PMID: 19861418 PMID: 19855005 PMID: 19852514 PMID: 19848310 PMID: 19848202 PMID: 19843875 PMID: 19838981 PMID: 19834040 PMID: 19834009 PMID: 19827031 PMID: 19825843 PMID: 19817790 PMID: 19817783 PMID: 19810772 PMID: 19805912 PMID: 19805580 PMID: 19801360 PMID: 19782947 PMID: 19777393 PMID: 19766909 PMID: 19758361 PMID: 19747951 PMID: 19744947 PMID: 19743879 PMID: 19730126 PMID: 19729020 PMID: 19721009 PMID: 19720788 PMID: 19718675 PMID: 19715772 PMID: 21099255 PMID: 19706790 PMID: 19701591 PMID: 19690174 PMID: 19683803 PMID: 19675133 PMID: 19675071 PMID: 19666551 PMID: 19657235 PMID: 19643665 PMID: 19643162 PMID: 19641898 PMID: 19640329 PMID: 19628574 PMID: 19617631 PMID: 19615802 PMID: 19610036 PMID: 19609006 PMID: 19605553 PMID: 19592596 PMID: 19584314 PMID: 19583950 PMID: 19581419 PMID: 19579421 PMID: 19576699 PMID: 19574449 PMID: 19566839 PMID: 19560552 PMID: 19558691 PMID: 19556421 PMID: 19542207 PMID: 19535454 PMID: 19531639 PMID: 19531027 PMID: 19507278 PMID: 19494813 PMID: 19492079 PMID: 19470833 PMID: 19466549 PMID: 19465539 PMID: 19461479 PMID: 19461116 PMID: 19442898 PMID: 19442239 PMID: 19442094 PMID: 19440038 PMID: 19432816 PMID: 19424493 PMID: 19421860 PMID: 19419817 PMID: 19414648 PMID: 19403868 PMID: 19396427 PMID: 19394661 PMID: 19376147 PMID: 19367387 PMID: 19366357 PMID: 19355910 PMID: 19349192 PMID: 19343319 PMID: 19325917 PMID: 19325007 PMID: 19322660 PMID: 19305497 PMID: 19300090 PMID: 19293267 PMID: 19275680 PMID: 19273167 PMID: 19273118 PMID: 19273093 PMID: 19272793 PMID: 19265784 PMID: 19264107 PMID: 19261693 PMID: 19259271 PMID: 19251722 PMID: 19251449 PMID: 19251050 PMID: 19243577 PMID: 19232402 PMID: 19223597 PMID: 19214472 PMID: 19203089 PMID: 19197367 PMID: 19196831 PMID: 19196457 PMID: 19190890 PMID: 19187771 PMID: 19177142 PMID: 19162042 PMID: 19154511 PMID: 19151199 PMID: 19148619 PMID: 19138703 PMID: 19133313 PMID: 19120277 PMID: 20707216 PMID: 19114643 PMID: 19106615 PMID: 19103817 PMID: 19100955 PMID: 19094928 PMID: 19084986 PMID: 19081854 PMID: 19080172 PMID: 19079765 PMID: 19066457 PMID: 19065603 PMID: 19046232 PMID: 19043985 PMID: 19034421 PMID: 19013511 PMID: 19004984 PMID: 18991026 PMID: 18973752 PMID: 18959742 PMID: 18959471 PMID: 18955471 PMID: 18929807 PMID: 18834448 PMID: 18834443 PMID: 18834442 PMID: 18834430 PMID: 18922796 PMID: 18854421 PMID: 18854316 PMID: 18842989 PMID: 18840766 PMID: 18839335 PMID: 18834896 PMID: 18818302 PMID: 18802678 PMID: 18801483 PMID: 18796632 PMID: 18793159 PMID: 18793158 PMID: 18793116 PMID: 18787107 PMID: 18786775 PMID: 18786605 PMID: 18780776 PMID: 18778365 PMID: 18777495 PMID: 18777097 PMID: 18776938 PMID: 18771589 PMID: 18753253 PMID: 18728232 PMID: 18723371 PMID: 18722695 PMID: 18719127 PMID: 18696046 PMID: 18694973 PMID: 18694957 PMID: 20151040 PMID: 18682384 PMID: 18680108 PMID: 18678790 PMID: 18649770 PMID: 18644846 PMID: 18644072 PMID: 18641056 PMID: 18640587 PMID: 18640585 PMID: 18633110 PMID: 18626456 PMID: 18624766 PMID: 18600596 PMID: 18594270 PMID: 18575325 PMID: 18570678 PMID: 18566681 PMID: 18560272 PMID: 18559892 PMID: 18552236 PMID: 18550852 PMID: 18550819 PMID: 18549351 PMID: 18541378 PMID: 18539434 PMID: 18508639 PMID: 18493032 PMID: 18483268 PMID: 18481955 PMID: 18481952 PMID: 18481951 PMID: 18470603 PMID: 18463842 PMID: 18456717 PMID: 18453752 PMID: 18446238 PMID: 18438526 PMID: 18435775 PMID: 18426796 PMID: 18423412 PMID: 18418065 PMID: 18410524 PMID: 18408913 PMID: 18403591 PMID: 18358424 PMID: 18385666 PMID: 18384109 PMID: 18377870 PMID: 18367022 PMID: 18362112 PMID: 18362060 PMID: 18359095 PMID: 18354376 PMID: 18350521 PMID: 18341726 PMID: 18336274 PMID: 18330713 PMID: 18320241 PMID: 18320236 PMID: 18317728 PMID: 18314421 PMID: 18305124 PMID: 18299579 PMID: 18294929 PMID: 18289831 PMID: 18288891 PMID: 18288288 PMID: 18287563 PMID: 18276926 PMID: 18276765 PMID: 18227473 PMID: 18270862 PMID: 18269177 PMID: 18258687 PMID: 18239070 PMID: 18234907 PMID: 19924273 PMID: 18230899 PMID: 18226850 PMID: 18226577 PMID: 18223009 PMID: 18221102 PMID: 18220630 PMID: 18220582 PMID: 18219478 PMID: 18216263 PMID: 18211828 PMID: 18200618 PMID: 18192848 PMID: 18184927 PMID: 18174526 PMID: 18174523 PMID: 18174159 PMID: 19337542 PMID: 19258739 PMID: 18162526 PMID: 18096375 PMID: 18093836 PMID: 18092938 PMID: 18084116 PMID: 18071026 PMID: 18061583 PMID: 18060615 PMID: 18058943 PMID: 18054361 PMID: 18048764 PMID: 18047804 PMID: 18033815 PMID: 18003756 PMID: 17984043 PMID: 17983555 PMID: 17982509 PMID: 17981116 PMID: 17977957 PMID: 17977949 PMID: 17971205 PMID: 17970760 PMID: 17963344 PMID: 17959932 PMID: 17958029 PMID: 17955017 PMID: 17952368 PMID: 17941874 PMID: 17933753 PMID: 17924122 PMID: 17921177 PMID: 17920690 PMID: 17919189 PMID: 17911348 PMID: 17911343 PMID: 17906960 PMID: 17904375 PMID: 17891928 PMID: 17882804 PMID: 17875968 PMID: 17848846 PMID: 17825811 PMID: 17761111 PMID: 17726733 PMID: 17725654 PMID: 17717515 PMID: 17717015 PMID: 17707128 PMID: 17701919 PMID: 17698735 PMID: 17693612 PMID: 17687076 PMID: 17679833 PMID: 17662078 PMID: 17660247 PMID: 17655515 PMID: 17645549 PMID: 17643134 PMID: 17642432 PMID: 17639046 PMID: 17637698 PMID: 17622750 PMID: 17620318 PMID: 17615956 PMID: 17611735 PMID: 17595242 PMID: 17593690 PMID: 17593232 PMID: 17592031 PMID: 17579776 PMID: 17577098 PMID: 17574431 PMID: 17570252 PMID: 17569617 PMID: 17569252 PMID: 17563269 PMID: 17563070 PMID: 17563067 PMID: 17556534 PMID: 17541845 PMID: 17533199 PMID: 17526769 PMID: 17508912 PMID: 17498508 PMID: 17496365 PMID: 17496235 PMID: 17495249 PMID: 17494630 PMID: 17491680 PMID: 17490981 PMID: 17483206 PMID: 17481767 PMID: 17475933 PMID: 17465340 PMID: 17451426 PMID: 17431229 PMID: 17429083 PMID: 17415412 PMID: 17400580 PMID: 17395747 PMID: 17379930 PMID: 17376428 PMID: 17373958 PMID: 17369526 PMID: 17353769 PMID: 17353506 PMID: 17353295 PMID: 17352408 PMID: 17349291 PMID: 17339833 PMID: 17339545 PMID: 17327433 PMID: 17318810 PMID: 17316868 PMID: 17311546 PMID: 17302974 PMID: 17299038 PMID: 17292559 PMID: 17272402 PMID: 17268797 PMID: 17267600 PMID: 17267584 PMID: 17267004 PMID: 17265033 PMID: 17259069 PMID: 17257271 PMID: 17256056 PMID: 17242177 PMID: 17234706 PMID: 17223037 PMID: 17216280 PMID: 17214554 PMID: 17207275 PMID: 17192466 PMID: 17192463 PMID: 17182546 PMID: 17179917 PMID: 17173503 PMID: 17158450 PMID: 17148734 PMID: 17142723 PMID: 17135364 PMID: 17131386 PMID: 17131145 PMID: 17130815 PMID: 17127438 PMID: 17114484 PMID: 17107885 PMID: 17096857 PMID: 17087876 PMID: 17077282 PMID: 17065351 PMID: 17065336 PMID: 17064973 PMID: 17053028 PMID: 17052195 PMID: 17023529 PMID: 17016695 PMID: 17008395 PMID: 16997142 PMID: 16973905 PMID: 16972538 PMID: 16970282 PMID: 16962100 PMID: 16959961 PMID: 16952202 PMID: 16941568 PMID: 16936197 PMID: 16936193 PMID: 16933212 PMID: 16929363 PMID: 16924412 PMID: 16922922 PMID: 16901490 PMID: 16899580 PMID: 16896941 PMID: 16892141 PMID: 16891625 PMID: 16884500 PMID: 16874076 PMID: 16873690 PMID: 16873687 PMID: 16873683 PMID: 16873681 PMID: 16869889 PMID: 16849627 PMID: 16846552 PMID: 16831155 PMID: 16820737 PMID: 16814095 PMID: 16805218 PMID: 16804197 PMID: 16804196 PMID: 16804082 PMID: 16801598 PMID: 16799131 PMID: 16782054 PMID: 16751422 PMID: 16741044 PMID: 16731826 PMID: 16731822 PMID: 16712485 PMID: 16706639 PMID: 16682937 PMID: 16682935 PMID: 16676355 PMID: 16675523 PMID: 16675487 PMID: 16642022 PMID: 16641553 PMID: 16631807 PMID: 16630750 PMID: 16611685 PMID: 16606331 PMID: 16601575 PMID: 16601139 PMID: 16598900 PMID: 16597547 PMID: 16574657 PMID: 16570161 PMID: 16567524 PMID: 16557185 PMID: 16556731 PMID: 16551748 PMID: 16530127 PMID: 16528573 PMID: 16517403 PMID: 16510765 PMID: 16505252 PMID: 16502254 PMID: 16497186 PMID: 16487789 PMID: 16480422 PMID: 16478293 PMID: 16472117 PMID: 16472114 PMID: 16467130 PMID: 16443786 PMID: 16440211 PMID: 16427178 PMID: 16424911 PMID: 16416271 PMID: 16409149 PMID: 16394656 PMID: 16380497 PMID: 16378747 PMID: 16373675 PMID: 16362279 PMID: 16361876 PMID: 16318402 PMID: 16306347 PMID: 16306329 PMID: 16306327 PMID: 16297610 PMID: 16283237 PMID: 16277639 PMID: 16270195 PMID: 16269465 PMID: 16265596 PMID: 16256381 PMID: 16256069 PMID: 16249450 PMID: 16249198 PMID: 16248975 PMID: 16248970 PMID: 16246168 PMID: 16242708 PMID: 16218490 PMID: 16204373 PMID: 16202636 PMID: 16176268 PMID: 16154537 PMID: 16123348 PMID: 16120142 PMID: 16120042 PMID: 16114871 PMID: 16114068 PMID: 16109478 PMID: 16091421 PMID: 16085563 PMID: 16078017 PMID: 16054982 PMID: 16051229 PMID: 16046410 PMID: 16046307 PMID: 16038259 PMID: 16037383 PMID: 16037235 PMID: 16030184 PMID: 16027122 PMID: 16026317 PMID: 16025228 PMID: 16023560 PMID: 16004588 PMID: 15983220 PMID: 15983205 PMID: 15974903 PMID: 15972661 PMID: 15968469 PMID: 15964236 PMID: 15962320 PMID: 15955371 PMID: 15920034 PMID: 15919807 PMID: 15919800 PMID: 15911336 PMID: 15910737 PMID: 15902400 PMID: 15896716 PMID: 15892647 PMID: 15886257 PMID: 15883500 PMID: 15880076 PMID: 15871360 PMID: 15867147 PMID: 15866237 PMID: 15860929 PMID: 15841036 PMID: 15833798 PMID: 15831571 PMID: 15821158 PMID: 15803113 PMID: 15800853 PMID: 15793246 PMID: 15781756 PMID: 15780433 PMID: 15780432 PMID: 15779361 PMID: 15777748 PMID: 15774474 PMID: 15772126 PMID: 15761720 PMID: 15749809 PMID: 15737001 PMID: 15734860 PMID: 15718270 PMID: 15716683 PMID: 15699492 PMID: 15695426 PMID: 15677515 PMID: 15667332 PMID: 15666485 PMID: 15662003 PMID: 15661873 PMID: 15660203 PMID: 15655721 PMID: 15655719 PMID: 15655712 PMID: 15655709 PMID: 15653566 PMID: 15642122 PMID: 15634886 PMID: 15622524 PMID: 15610048 PMID: 15607333 PMID: 15596565 PMID: 15592487 PMID: 15587404 PMID: 15582286 PMID: 15582161 PMID: 15578154 PMID: 15564445 PMID: 15561943 PMID: 15539803 PMID: 15538738 PMID: 15537654 PMID: 15536197 PMID: 15531508 PMID: 15525588 PMID: 15505070 PMID: 15504977 PMID: 15490413 PMID: 15483097 PMID: 15479216 PMID: 15472206 PMID: 15464423 PMID: 15454397 PMID: 15448084 PMID: 15358243 PMID: 15340105 PMID: 15322087 PMID: 15297438 PMID: 15292354 PMID: 15292032 PMID: 15289644 PMID: 15285799 PMID: 15277383 PMID: 15277379 PMID: 15271644 PMID: 15259301 PMID: 15249995 PMID: 15242807 PMID: 15242488 PMID: 15220215 PMID: 15220194 PMID: 15199129 PMID: 15188490 PMID: 15184981 PMID: 15180456 PMID: 15178687 PMID: 15161755 PMID: 15155383 PMID: 15134281 PMID: 15126294 PMID: 15095038 PMID: 15078165 PMID: 15078145 PMID: 15077172 PMID: 15047614 PMID: 15044459 PMID: 15040151 PMID: 15023346 PMID: 14981761 PMID: 14978124 PMID: 14966349 PMID: 14767595 PMID: 14766004 PMID: 14749285 PMID: 14748069 PMID: 14724691 PMID: 14722646 PMID: 14700743 PMID: 14692396 PMID: 14690455 PMID: 14679077 PMID: 14678863 PMID: 14678861 PMID: 14668049 PMID: 14664701 PMID: 14654976 PMID: 14640937 PMID: 14645111 PMID: 14641066 PMID: 14634862 PMID: 14634011 PMID: 14625688 PMID: 14617753 PMID: 14617576 PMID: 14610263 PMID: 14599964 PMID: 14593613 PMID: 14592444 PMID: 14568969 PMID: 14555218 PMID: 14555214 PMID: 14555211 PMID: 14532296 PMID: 14529935 PMID: 14529486 PMID: 14525917 PMID: 12950026 PMID: 12949498 PMID: 12941770 PMID: 12930893 PMID: 12928773 PMID: 12887285 PMID: 12881480 PMID: 12879149 PMID: 12843569 PMID: 12829636 PMID: 12801084 PMID: 12795417 PMID: 12759426 PMID: 12757020 PMID: 12754126 PMID: 12749025 PMID: 12732844 PMID: 12721668 PMID: 12716748 PMID: 12706864 PMID: 12703062 PMID: 12684506 PMID: 12676457 PMID: 12675249 PMID: 12663471 PMID: 12662297 PMID: 12660412 PMID: 12646174 PMID: 12643211 PMID: 12632104 PMID: 12624711 PMID: 12606514 PMID: 12604246 PMID: 12600983 PMID: 12594227 PMID: 12591159 PMID: 12589707 PMID: 12585335 PMID: 12534368 PMID: 12531555 PMID: 12523500 PMID: 12515900 PMID: 12514170 PMID: 12502499 PMID: 12490536 PMID: 12475794 PMID: 12471115 PMID: 12470987 PMID: 12470657 PMID: 12469357 PMID: 12453906 PMID: 12451262 PMID: 12447984 PMID: 12446608 PMID: 12414951 PMID: 12412268 PMID: 12384545 PMID: 12384512 PMID: 12381965 PMID: 12357262 PMID: 12242036 PMID: 12238086 PMID: 12235117 PMID: 12191616 PMID: 12183643 PMID: 12173733 PMID: 12086956 PMID: 12082115 PMID: 12060768 PMID: 12040173 PMID: 12036921 PMID: 12031968 PMID: 12021097 PMID: 12007724 PMID: 11960604 PMID: 11916941 PMID: 11916613 PMID: 11899081 PMID: 11899077 PMID: 11897677 PMID: 11861804 PMID: 11854325 PMID: 11850586 PMID: 11832466 PMID: 11818477 PMID: 11812749 PMID: 11795838 PMID: 11795511 PMID: 11786083 PMID: 11770125 PMID: 11751624 PMID: 11744330 PMID: 11717249 PMID: 11716549 PMID: 11707430 PMID: 11704806 PMID: 11696198 PMID: 11692177 PMID: 11687580 PMID: 11677087 PMID: 11641234 PMID: 11596676 PMID: 11574401 PMID: 11473037 PMID: 11473033 PMID: 11473025 PMID: 11467344 PMID: 11454519 PMID: 11441119 PMID: 11437491 PMID: 11437072 PMID: 11431335 PMID: 11427504 PMID: 11375353 PMID: 11375329 PMID: 11356709 PMID: 11342558 PMID: 11334407 PMID: 11321834 PMID: 11321039 PMID: 11319840 PMID: 11309529 PMID: 11272210 PMID: 11272209 PMID: 11272206 PMID: 11272204 PMID: 11272200 PMID: 11272176 PMID: 11272169 PMID: 11272167 PMID: 11270678 PMID: 11160146 PMID: 11147797 PMID: 11140462 PMID: 11126236 PMID: 11110476 PMID: 11071652 PMID: 11046041 PMID: 11043861 PMID: 11014617 PMID: 10967112 PMID: 10967106 PMID: 10966825 PMID: 10960489 PMID: 10945145 PMID: 10945143 PMID: 10923635 PMID: 10907126 PMID: 10903806 PMID: 10902571 PMID: 10880889 PMID: 10842665 PMID: 10820254 PMID: 10717000 PMID: 10700186 PMID: 10634421 PMID: 10601879 PMID: 10599690 PMID: 10594015 PMID: 10580429 PMID: 10579924 PMID: 10567588 PMID: 10548879 PMID: 10523611 PMID: 10515579 PMID: 10512758 PMID: 10455420 PMID: 10454216 PMID: 10417742 PMID: 10393700 PMID: 10385418 PMID: 10382745 PMID: 10334931 PMID: 10212493 PMID: 10206484 PMID: 10199124 PMID: 10102689 PMID: 9928426 PMID: 9856487 PMID: 9846494 PMID: 9824642 PMID: 9769338 PMID: 9748221 PMID: 9742505 PMID: 9679966 PMID: 9654911 PMID: 9652687 PMID: 9462471 PMID: 9098622 PMID: 8990188 PMID: 9009215 PMID: 9003372 PMID: 8764141 PMID: 8738973 PMID: 8786019 PMID: 7533382 PMID: 8152488 |
| Arachidonic acid metabolism | PMID: 20976215 PMID: 20159982 PMID: 19268562 PMID: 15166011 PMID: 15047620 PMID: 12566962 PMID: 10963721 PMID: 2886201 PMID: 3037972 |
| Arginine and proline metabolism | PMID: 17380316 PMID: 17192473 PMID: 16603055 PMID: 16356114 PMID: 11520305 PMID: 8267567 PMID: 1972412 PMID: 1195561 |
| Arrhythmogenic right ventricular cardiomyopathy (ARVC) |  |
| Asthma | PMID: 21239605 PMID: 21195808 PMID: 21189220 PMID: 21134413 PMID: 21095606 PMID: 21083567 PMID: 21067254 PMID: 21044362 PMID: 20977421 PMID: 20953658 PMID: 20951781 PMID: 20926152 PMID: 20842323 PMID: 20810049 PMID: 20809911 PMID: 20693905 PMID: 20688047 PMID: 20590749 PMID: 20584522 PMID: 20381783 PMID: 20373858 PMID: 20368314 PMID: 20348195 PMID: 20339873 PMID: 20228001 PMID: 20135885 PMID: 20080911 PMID: 20067162 PMID: 19926696 PMID: 19849760 PMID: 19604068 PMID: 19827919 PMID: 19808222 PMID: 19772447 PMID: 19782552 PMID: 19705789 PMID: 19553520 PMID: 19534611 PMID: 19527717 PMID: 19468225 PMID: 19463522 PMID: 19445271 PMID: 19444913 PMID: 19434036 PMID: 19421404 PMID: 19371271 PMID: 19365600 PMID: 19331277 PMID: 19288426 PMID: 19253132 PMID: 19251584 PMID: 19130149 PMID: 21119973 PMID: 18950252 PMID: 18987275 PMID: 19029235 PMID: 18851683 PMID: 18840374 PMID: 18798460 PMID: 18768484 PMID: 18754753 PMID: 18725905 PMID: 18664294 PMID: 18637769 PMID: 18501314 PMID: 18562982 PMID: 18494753 PMID: 18430314 PMID: 18405959 PMID: 18366306 PMID: 18363445 PMID: 20525124 PMID: 18321936 PMID: 18297432 PMID: 18290917 PMID: 18241617 PMID: 18226895 PMID: 18179620 PMID: 18070293 PMID: 18061654 PMID: 18041101 PMID: 18030180 PMID: 17966884 PMID: 17897077 PMID: 17764214 PMID: 17601183 PMID: 17568517 PMID: 17566142 PMID: 17464160 PMID: 17442118 PMID: 17344572 PMID: 17286919 PMID: 17270109 PMID: 17267571 PMID: 17211725 PMID: 17200270 PMID: 17188840 PMID: 17162357 PMID: 17066246 PMID: 17045197 PMID: 17039328 PMID: 17000564 PMID: 16968414 PMID: 16925332 PMID: 16925217 PMID: 16888333 PMID: 16832355 PMID: 16813481 PMID: 16759099 PMID: 16706706 PMID: 16700887 PMID: 16613348 PMID: 16563035 PMID: 16528893 PMID: 16458290 PMID: 16451303 PMID: 16427178 PMID: 16416887 PMID: 16387690 PMID: 16352115 PMID: 16277033 PMID: 16270716 PMID: 16257992 PMID: 16207641 PMID: 16190176 PMID: 16117010 PMID: 16048553 PMID: 16009053 PMID: 15974215 PMID: 15911336 PMID: 15893131 PMID: 15853687 PMID: 15804132 PMID: 15755851 PMID: 15734727 PMID: 15708532 PMID: 15659827 PMID: 15649101 PMID: 15623392 PMID: 15608802 PMID: 15507333 PMID: 15500899 PMID: 15487110 PMID: 15451919 PMID: 15250842 PMID: 15175863 PMID: 15114276 PMID: 15102433 PMID: 15050029 PMID: 15032089 PMID: 14987333 PMID: 14747686 PMID: 14712310 PMID: 14641605 PMID: 14552435 PMID: 14532367 PMID: 14513541 PMID: 12882854 PMID: 12879830 PMID: 12841425 PMID: 12742804 PMID: 12716786 PMID: 12556276 PMID: 12530119 PMID: 12527352 PMID: 12527343 PMID: 12448803 PMID: 12392119 PMID: 12198000 PMID: 12007704 PMID: 11950134 PMID: 11899077 PMID: 11887620 PMID: 11835120 PMID: 11698039 PMID: 11588082 PMID: 11565063 PMID: 11476433 PMID: 11475277 PMID: 11456001 PMID: 11405452 PMID: 11369722 PMID: 11277472 PMID: 11171680 PMID: 11037336 PMID: 11015800 PMID: 10993010 PMID: 10931791 PMID: 10736928 PMID: 10549054 PMID: 10499817 PMID: 10048503 PMID: 9988276 PMID: 9766480 PMID: 9755281 PMID: 9602451 PMID: 9357379 PMID: 10868186 PMID: 8593722 PMID: 8691781 PMID: 7855158 PMID: 8164243 PMID: 8310115 PMID: 1298192 PMID: 1614803 PMID: 1489551 PMID: 2402719 PMID: 2213076 PMID: 2133906 PMID: 2789974 PMID: 3117081 PMID: 3877654 PMID: 3915545 PMID: 315113 PMID: 82296 PMID: 4100384 |
| Autoimmune thyroid disease | PMID: 21046886 PMID: 20505260 PMID: 19822041 PMID: 19891010 PMID: 19214801 PMID: 18719369 PMID: 18466208 PMID: 18029461 PMID: 17593598 PMID: 17341425 PMID: 17130562 PMID: 16704751 PMID: 16503115 PMID: 16405252 PMID: 15934099 PMID: 15142366 PMID: 15037992 PMID: 12911285 PMID: 12198602 PMID: 11854095 PMID: 11772912 PMID: 11110510 PMID: 1655787 PMID: 2903375 PMID: 3631074 PMID: 3456197 PMID: 301726 |
| Axon guidance | PMID: 18078524 PMID: 12678846 |
| B cell receptor signaling pathway |  |
| Basal cell carcinoma | PMID: 20016592 PMID: 17344960 |
| Base excision repair | PMID: 19514640 PMID: 14641066 PMID: 10856830 |
| Biosynthesis of unsaturated fatty acids | PMID: 21173413 PMID: 20976215 PMID: 20888206 PMID: 20840078 PMID: 20628088 PMID: 20627036 PMID: 20595648 PMID: 20535444 PMID: 20515815 PMID: 20511412 PMID: 20493223 PMID: 20484485 PMID: 20478379 PMID: 20471368 PMID: 20450981 PMID: 20426869 PMID: 20426498 PMID: 20335546 PMID: 20334541 PMID: 20304070 PMID: 20302646 PMID: 20206490 PMID: 20191628 PMID: 20190465 PMID: 20189787 PMID: 20181814 PMID: 20159982 PMID: 20158940 PMID: 20136655 PMID: 20120158 PMID: 20117255 PMID: 20116951 PMID: 20088941 PMID: 20080856 PMID: 20071694 PMID: 20068136 PMID: 20063116 PMID: 20043885 PMID: 20042400 PMID: 20042103 PMID: 20041814 PMID: 20026767 PMID: 20026239 PMID: 19998382 PMID: 19958109 PMID: 19952344 PMID: 19933995 PMID: 19890623 PMID: 19875212 PMID: 19843871 PMID: 19841042 PMID: 19837872 PMID: 19834040 PMID: 19834009 PMID: 19833861 PMID: 19828904 PMID: 19826189 PMID: 19822001 PMID: 19814866 PMID: 19798065 PMID: 19782984 PMID: 19781663 PMID: 19780047 PMID: 19770031 PMID: 19745037 PMID: 19716144 PMID: 19698199 PMID: 19660790 PMID: 19652946 PMID: 19647413 PMID: 19628666 PMID: 19609006 PMID: 19604407 PMID: 19595055 PMID: 19593479 PMID: 19584878 PMID: 19582722 PMID: 19575453 PMID: 19571460 PMID: 19535429 PMID: 19521344 PMID: 19515732 PMID: 19459227 PMID: 19446448 PMID: 19397693 PMID: 19394939 PMID: 19374389 PMID: 19367114 PMID: 19364085 PMID: 19336640 PMID: 19299433 PMID: 19279518 PMID: 19268562 PMID: 19238139 PMID: 19236624 PMID: 19186972 PMID: 19172517 PMID: 19164460 PMID: 19154947 PMID: 19141690 PMID: 19133313 PMID: 19103735 PMID: 19066312 PMID: 19037880 PMID: 19032965 PMID: 19013603 PMID: 18991244 PMID: 18957534 PMID: 18855149 PMID: 18845058 PMID: 18793165 PMID: 18793159 PMID: 18790133 PMID: 18718737 PMID: 18706904 PMID: 18702940 PMID: 18682384 PMID: 18641279 PMID: 18641273 PMID: 18640380 PMID: 18619553 PMID: 18599066 PMID: 18571145 PMID: 18561722 PMID: 18559892 PMID: 18296340 PMID: 18491996 PMID: 18484615 PMID: 18450829 PMID: 18435912 PMID: 18417641 PMID: 18394213 PMID: 18391475 PMID: 18330713 PMID: 18324928 PMID: 18310441 PMID: 18301838 PMID: 18253705 PMID: 18252961 PMID: 18248766 PMID: 18248310 PMID: 18239594 PMID: 18229449 PMID: 18220672 PMID: 18199587 PMID: 18174526 PMID: 18065585 PMID: 18060615 PMID: 18059580 PMID: 18059573 PMID: 18042831 PMID: 17943024 PMID: 17876199 PMID: 17922004 PMID: 17914131 PMID: 17898498 PMID: 17888088 PMID: 17880934 PMID: 17878672 PMID: 17825794 PMID: 17804680 PMID: 17765364 PMID: 17719033 PMID: 17717282 PMID: 17712117 PMID: 17708582 PMID: 17704823 PMID: 17699519 PMID: 17698319 PMID: 17667864 PMID: 17644349 PMID: 17641276 PMID: 17640561 PMID: 17616780 PMID: 17612649 PMID: 17597010 PMID: 17581795 PMID: 17568784 PMID: 17555759 PMID: 17546512 PMID: 17521952 PMID: 17513496 PMID: 17513398 PMID: 17490984 PMID: 17467667 PMID: 17389722 PMID: 17379187 PMID: 17377208 PMID: 17351370 PMID: 17346075 PMID: 17344486 PMID: 17341567 PMID: 17339025 PMID: 17323614 PMID: 17319106 PMID: 17292728 PMID: 17284731 PMID: 17257774 PMID: 17254607 PMID: 17241282 PMID: 17213792 PMID: 17211560 PMID: 17170520 PMID: 17148742 PMID: 17143195 PMID: 17111027 PMID: 17110421 PMID: 17098406 PMID: 17093147 PMID: 17065351 PMID: 17049925 PMID: 17015262 PMID: 17014955 PMID: 17003343 PMID: 17002473 PMID: 16997891 PMID: 16979408 PMID: 16960167 PMID: 16920849 PMID: 16873378 PMID: 16870193 PMID: 16869997 PMID: 16841860 PMID: 16788801 PMID: 16784183 PMID: 16777940 PMID: 16731826 PMID: 16731825 PMID: 16709900 PMID: 16697496 PMID: 16644178 PMID: 16616147 PMID: 16609090 PMID: 16601139 PMID: 16596812 PMID: 16584877 PMID: 16569732 PMID: 16555470 PMID: 16525722 PMID: 16512956 PMID: 16472050 PMID: 16458290 PMID: 16444875 PMID: 16412866 PMID: 16399491 PMID: 16394171 PMID: 16367888 PMID: 16367886 PMID: 16335699 PMID: 16332647 PMID: 16297610 PMID: 16285996 PMID: 16280424 PMID: 16269450 PMID: 16249527 PMID: 16222068 PMID: 16202646 PMID: 16186383 PMID: 16179348 PMID: 16150913 PMID: 16125723 PMID: 16111676 PMID: 16096283 PMID: 16079262 PMID: 15983525 PMID: 15975108 PMID: 15967412 PMID: 15947245 PMID: 15939440 PMID: 15935394 PMID: 15905055 PMID: 15882258 PMID: 15877301 PMID: 15871306 PMID: 15863958 PMID: 15854663 PMID: 15746832 PMID: 15735215 PMID: 15735084 PMID: 15734839 PMID: 15726821 PMID: 15716329 PMID: 15653566 PMID: 15642122 PMID: 15626691 PMID: 15624100 PMID: 15598677 PMID: 15589689 PMID: 15555528 PMID: 15489540 PMID: 15477793 PMID: 15467284 PMID: 15462110 PMID: 15458541 PMID: 15447895 PMID: 15370192 PMID: 15338976 PMID: 15334378 PMID: 15333496 PMID: 15321807 PMID: 15313105 PMID: 15304032 PMID: 15297438 PMID: 15292032 PMID: 15261979 PMID: 15240650 PMID: 15233403 PMID: 15226463 PMID: 15168245 PMID: 15166011 PMID: 15159252 PMID: 15130939 PMID: 15114276 PMID: 15081318 PMID: 15073188 PMID: 15063832 PMID: 15047620 PMID: 15037525 PMID: 15037160 PMID: 15015713 PMID: 15013642 PMID: 15006938 PMID: 15001601 PMID: 14984449 PMID: 14767868 PMID: 14749748 PMID: 14651988 PMID: 14634862 PMID: 14634727 PMID: 14607907 PMID: 14534304 PMID: 14526661 PMID: 12936956 PMID: 12923230 PMID: 12912808 PMID: 12878589 PMID: 12871497 PMID: 12837757 PMID: 12824244 PMID: 12814394 PMID: 12810609 PMID: 12766112 PMID: 12734208 PMID: 12716760 PMID: 12706573 PMID: 12679187 PMID: 12663471 PMID: 12648158 PMID: 12627189 PMID: 12606514 PMID: 12601631 PMID: 12600850 PMID: 12595963 PMID: 12566962 PMID: 12566135 PMID: 12536730 PMID: 12514304 PMID: 12482637 PMID: 12461476 PMID: 12428181 PMID: 12421024 PMID: 12399622 PMID: 12399271 PMID: 12355797 PMID: 12354173 PMID: 12204421 PMID: 12145331 PMID: 12145143 PMID: 12082115 PMID: 12054917 PMID: 12051519 PMID: 12044579 PMID: 12036809 PMID: 12031964 PMID: 12031899 PMID: 12031597 PMID: 12012138 PMID: 11974950 PMID: 11960511 PMID: 11960303 PMID: 11953170 PMID: 11925664 PMID: 11924732 PMID: 11916915 PMID: 11887169 PMID: 11874943 PMID: 11874925 PMID: 11841597 PMID: 11834254 PMID: 11834092 PMID: 11819026 PMID: 11774104 PMID: 11758971 PMID: 11755918 PMID: 11673365 PMID: 11584100 PMID: 11563968 PMID: 11557664 PMID: 11555841 PMID: 11535127 PMID: 11527989 PMID: 11522680 PMID: 11521381 PMID: 11520945 PMID: 11494668 PMID: 11473037 PMID: 11443197 PMID: 11425290 PMID: 11385059 PMID: 11378531 PMID: 11377701 PMID: 11319719 PMID: 11311735 PMID: 11272131 PMID: 11246888 PMID: 11246821 PMID: 11241890 PMID: 11229438 PMID: 11214722 PMID: 11181530 PMID: 11172785 PMID: 11168342 PMID: 11147797 PMID: 11063437 PMID: 11052098 PMID: 11023139 PMID: 11015481 PMID: 10963721 PMID: 10889809 PMID: 10884525 PMID: 10859688 PMID: 10757544 PMID: 10700478 PMID: 10670825 PMID: 10667368 PMID: 10637124 PMID: 10622744 PMID: 10580615 PMID: 10559003 PMID: 10556990 PMID: 10509765 PMID: 10501817 PMID: 10479232 PMID: 10403584 PMID: 10386239 PMID: 10363159 PMID: 10329986 PMID: 10097906 PMID: 10078565 PMID: 10078552 PMID: 9888203 PMID: 9722689 PMID: 9690343 PMID: 9664946 PMID: 9658700 PMID: 9655393 PMID: 9576750 PMID: 9598837 PMID: 9568691 PMID: 9538963 PMID: 9519749 PMID: 9465095 PMID: 9451469 PMID: 9229225 PMID: 9135961 PMID: 9217887 PMID: 9161931 PMID: 9058329 PMID: 8826976 PMID: 8706072 PMID: 8778000 PMID: 8740245 PMID: 8593943 PMID: 8723029 PMID: 8893973 PMID: 7595053 PMID: 7626978 PMID: 7865979 PMID: 7800581 PMID: 7956714 PMID: 8181259 PMID: 7946534 PMID: 8108349 PMID: 7505737 PMID: 8322798 PMID: 8352447 PMID: 8352446 PMID: 8102516 PMID: 8495604 PMID: 8354639 PMID: 1478157 PMID: 1406295 PMID: 1636624 PMID: 1628764 PMID: 1601393 PMID: 1563246 PMID: 1543584 PMID: 1490671 PMID: 1346344 PMID: 1294269 PMID: 1962521 PMID: 1749204 PMID: 1827815 PMID: 1851897 PMID: 1973671 PMID: 2146101 PMID: 2379765 PMID: 2338079 PMID: 2526814 PMID: 2583313 PMID: 2527675 PMID: 2653924 PMID: 2527507 PMID: 2492785 PMID: 2667842 PMID: 2650692 PMID: 3379125 PMID: 2965654 PMID: 2827307 PMID: 3355569 PMID: 2978874 PMID: 2886201 PMID: 3123643 PMID: 3900961 PMID: 3838894 PMID: 6715541 PMID: 6322880 PMID: 6607315 PMID: 6671043 PMID: 6194801 PMID: 7030050 PMID: 7027326 PMID: 330566 PMID: 192618 PMID: 185093 PMID: 1029016 PMID: 970068 PMID: 1133176 PMID: 4257822 |
| Bladder cancer | PMID: 20973689 PMID: 20526368 PMID: 19286253 PMID: 18630627 PMID: 15133541 |
| Calcium signaling pathway | PMID: 20346437 PMID: 19199708 |
| Cardiac muscle contraction | PMID: 17675413 |
| Cell adhesion molecules (CAMs) | PMID: 21241985 PMID: 20718759 PMID: 18083068 |
| Cell cycle | PMID: 21186350 PMID: 21099336 PMID: 21099295 PMID: 21088486 PMID: 21071958 PMID: 21057731 PMID: 21038470 PMID: 20962845 PMID: 20956556 PMID: 20697199 PMID: 20676397 PMID: 20659423 PMID: 20644627 PMID: 20592469 PMID: 20581827 PMID: 20567803 PMID: 20534483 PMID: 20529852 PMID: 20461355 PMID: 20406885 PMID: 20404041 PMID: 20393693 PMID: 20392809 PMID: 20372078 PMID: 20299467 PMID: 20227390 PMID: 20202486 PMID: 20182580 PMID: 20161975 PMID: 20158461 PMID: 20103709 PMID: 20090419 PMID: 19924153 PMID: 19902175 PMID: 19902174 PMID: 19901535 PMID: 19897022 PMID: 19874425 PMID: 19843526 PMID: 19833888 PMID: 19779039 PMID: 19695727 PMID: 19681769 PMID: 19670153 PMID: 19634152 PMID: 19633416 PMID: 19607911 PMID: 19580824 PMID: 19541499 PMID: 19492410 PMID: 19491042 PMID: 19440038 PMID: 19416712 PMID: 19292868 PMID: 19291425 PMID: 19265784 PMID: 19260450 PMID: 19197367 PMID: 19177142 PMID: 19171749 PMID: 19139803 PMID: 19122346 PMID: 19107194 PMID: 19106615 PMID: 19106089 PMID: 19079765 PMID: 18984671 PMID: 18949453 PMID: 18927507 PMID: 18845673 PMID: 18834896 PMID: 18771589 PMID: 18719369 PMID: 18708447 PMID: 18624957 PMID: 18624766 PMID: 18597805 PMID: 18593768 PMID: 18567820 PMID: 18566945 PMID: 18534819 PMID: 18505768 PMID: 18418065 PMID: 18396382 PMID: 18391968 PMID: 18347327 PMID: 18347054 PMID: 18288288 PMID: 18230899 PMID: 18226850 PMID: 18199129 PMID: 19258739 PMID: 17955017 PMID: 17952368 PMID: 17900474 PMID: 17726733 PMID: 17688680 PMID: 17643134 PMID: 17611413 PMID: 17577098 PMID: 17569617 PMID: 17562312 PMID: 17438370 PMID: 17372192 PMID: 17371253 PMID: 17329594 PMID: 17320348 PMID: 17307971 PMID: 17130512 PMID: 17065351 PMID: 17016695 PMID: 17003479 PMID: 16996226 PMID: 16988714 PMID: 16980585 PMID: 16970282 PMID: 16873690 PMID: 16721061 PMID: 16675523 PMID: 16638909 PMID: 16527893 PMID: 16505226 PMID: 16427178 PMID: 16421191 PMID: 16387690 PMID: 16361876 PMID: 16283526 PMID: 16252239 PMID: 16248975 PMID: 16223861 PMID: 16123373 PMID: 16121806 PMID: 16085563 PMID: 16078017 PMID: 16024793 PMID: 15846093 PMID: 15774581 PMID: 15762051 PMID: 15698853 PMID: 15691225 PMID: 15685168 PMID: 15615695 PMID: 15596565 PMID: 15569940 PMID: 15494484 PMID: 15369805 PMID: 15277379 PMID: 15107844 PMID: 14724187 PMID: 14712063 PMID: 14702136 PMID: 14679077 PMID: 14678872 PMID: 14640937 PMID: 14500580 PMID: 13680128 PMID: 12958197 PMID: 12928786 PMID: 12881480 PMID: 12829636 PMID: 12742988 PMID: 12482142 PMID: 12475794 PMID: 12470657 PMID: 12444170 PMID: 12223530 PMID: 12034374 PMID: 12028372 PMID: 12010913 PMID: 11978627 PMID: 11932410 PMID: 11786083 PMID: 11733490 PMID: 11544286 PMID: 11440895 PMID: 11431335 PMID: 11270678 PMID: 10510592 PMID: 10444593 PMID: 10417742 PMID: 9824642 PMID: 9614078 PMID: 9341881 PMID: 8995379 PMID: 9352601 PMID: 7557392 PMID: 8087098 PMID: 8409387 PMID: 3289893 PMID: 2965106 PMID: 3097892 PMID: 6381187 PMID: 6336699 PMID: 6761212 |
| Chemokine signaling pathway |  |
| Chronic myeloid leukemia | PMID: 20466781 PMID: 17490925 PMID: 16873671 PMID: 8347786 |
| Circadian rhythm | PMID: 21188086 PMID: 21134267 PMID: 21089455 PMID: 21067753 PMID: 20978217 PMID: 20977575 PMID: 20852621 PMID: 20801941 PMID: 20719784 PMID: 20714447 PMID: 20704750 PMID: 20587581 PMID: 20536488 PMID: 20515582 PMID: 20391959 PMID: 20371702 PMID: 20337199 PMID: 20303779 PMID: 20215452 PMID: 20200113 PMID: 20168303 PMID: 20167180 PMID: 20167164 PMID: 20144320 PMID: 20086313 PMID: 20086254 PMID: 20017345 PMID: 19949414 PMID: 19934019 PMID: 19931237 PMID: 19911281 PMID: 19849798 PMID: 19820277 PMID: 19730439 PMID: 19649379 PMID: 19647043 PMID: 19640600 PMID: 19637052 PMID: 19574505 PMID: 19542260 PMID: 19454385 PMID: 19396423 PMID: 19387896 PMID: 19376230 PMID: 19368099 PMID: 19318453 PMID: 19229515 PMID: 19218356 PMID: 19204728 PMID: 20481040 PMID: 19098379 PMID: 19060909 PMID: 18974966 PMID: 18926758 PMID: 18833198 PMID: 18821999 PMID: 18708447 PMID: 18677589 PMID: 18632179 PMID: 18624957 PMID: 18611975 PMID: 18565832 PMID: 18547740 PMID: 18537095 PMID: 18475045 PMID: 18445997 PMID: 18435852 PMID: 18355327 PMID: 18291549 PMID: 18250492 PMID: 18202529 PMID: 18201207 PMID: 18174766 PMID: 18157388 PMID: 18155533 PMID: 18060321 PMID: 18055507 PMID: 18033815 PMID: 18021495 PMID: 17984670 PMID: 17984657 PMID: 17936663 PMID: 17903691 PMID: 17846932 PMID: 17803699 PMID: 17728404 PMID: 17696960 PMID: 17674598 PMID: 17536225 PMID: 17516289 PMID: 17495595 PMID: 17495205 PMID: 17485018 PMID: 17459509 PMID: 17387040 PMID: 17372301 PMID: 17351155 PMID: 17299078 PMID: 17264230 PMID: 17190729 PMID: 17177134 PMID: 17002929 PMID: 16968545 PMID: 16933182 PMID: 16926523 PMID: 16893914 PMID: 16817500 PMID: 16752180 PMID: 16752164 PMID: 16730846 PMID: 16703331 PMID: 16670002 PMID: 16632233 PMID: 16627386 PMID: 16567517 PMID: 16531792 PMID: 16513202 PMID: 16508584 PMID: 16452548 PMID: 16389896 PMID: 16343098 PMID: 16342516 PMID: 16197556 PMID: 16188167 PMID: 16188164 PMID: 16186263 PMID: 16166217 PMID: 16151976 PMID: 16141627 PMID: 16043755 PMID: 16033737 PMID: 16032524 PMID: 15935772 PMID: 15921073 PMID: 15835381 PMID: 15787659 PMID: 15773667 PMID: 15771217 PMID: 15738376 PMID: 15736102 PMID: 15715886 PMID: 15620438 PMID: 15612451 PMID: 15589062 PMID: 15533587 PMID: 15506069 PMID: 15470952 PMID: 15333478 PMID: 15321011 PMID: 15309293 PMID: 15306836 PMID: 15126524 PMID: 15111485 PMID: 15096902 PMID: 15070752 PMID: 14671173 PMID: 14633813 PMID: 14617230 PMID: 14601484 PMID: 14520604 PMID: 12898471 PMID: 12823235 PMID: 12783235 PMID: 12765958 PMID: 12759896 PMID: 12725703 PMID: 12702002 PMID: 12690088 PMID: 12678465 PMID: 12643178 PMID: 12610053 PMID: 12606508 PMID: 12547905 PMID: 12450440 PMID: 12206997 PMID: 12208479 PMID: 12198220 PMID: 12196337 PMID: 12188392 PMID: 12085419 PMID: 11994332 PMID: 11956158 PMID: 11939615 PMID: 11919134 PMID: 11895469 PMID: 11850098 PMID: 11788653 PMID: 11728411 PMID: 11483228 PMID: 11481396 PMID: 11469634 PMID: 11469633 PMID: 11353879 PMID: 11333703 PMID: 11323088 PMID: 11319671 PMID: 11315821 PMID: 11298733 PMID: 11246884 PMID: 11238483 PMID: 11221809 PMID: 11220353 PMID: 11195180 PMID: 11120658 PMID: 11092282 PMID: 11071082 PMID: 10989751 PMID: 10937511 PMID: 10937510 PMID: 10906034 PMID: 10868852 PMID: 10821300 PMID: 10811590 PMID: 10799372 PMID: 10770179 PMID: 10730548 PMID: 10633880 PMID: 10587845 PMID: 10580431 PMID: 10535452 PMID: 10445830 PMID: 10331415 PMID: 10321421 PMID: 9611154 PMID: 9610651 PMID: 9540013 PMID: 9435533 PMID: 9405900 PMID: 9300232 PMID: 9276727 PMID: 9324678 PMID: 8931842 PMID: 8931637 PMID: 8949375 PMID: 8757028 PMID: 8807356 PMID: 8542099 PMID: 8548941 PMID: 8544414 PMID: 7485122 PMID: 8573735 PMID: 8003272 PMID: 8306591 PMID: 8256949 PMID: 8411838 PMID: 8433075 PMID: 8361872 PMID: 8427656 PMID: 1473323 PMID: 1771927 PMID: 1955099 PMID: 1953255 PMID: 1647314 PMID: 1785335 PMID: 2086278 PMID: 2201497 PMID: 2124448 PMID: 2307090 PMID: 2272633 PMID: 2217326 PMID: 2666058 PMID: 2696935 PMID: 2697868 PMID: 3401978 PMID: 3423777 PMID: 3781477 PMID: 3518782 PMID: 4060795 PMID: 6376243 PMID: 6350027 PMID: 6354700 PMID: 7172871 PMID: 7047271 PMID: 7018146 PMID: 7018102 PMID: 6776763 PMID: 1030662 PMID: 964510 |
| Citrate cycle (TCA cycle) | PMID: 21076574 PMID: 20981252 PMID: 20804614 PMID: 19892241 PMID: 19887598 PMID: 19369366 PMID: 19193946 PMID: 18728221 PMID: 18653763 PMID: 18587560 PMID: 18561209 PMID: 18543755 PMID: 17726021 PMID: 17709878 PMID: 17416903 PMID: 17287462 PMID: 17125967 PMID: 16081739 PMID: 15562253 PMID: 15507531 PMID: 12832613 PMID: 12769660 PMID: 12446586 PMID: 9228023 PMID: 1532153 PMID: 1897977 PMID: 6969570 PMID: 124319 |
| Colorectal cancer | PMID: 21233058 PMID: 20937982 PMID: 20633560 PMID: 20463425 PMID: 20460980 PMID: 20406885 PMID: 20227390 PMID: 20123124 PMID: 20056134 PMID: 19773552 PMID: 19664792 PMID: 19509565 PMID: 19449106 PMID: 19413174 PMID: 19223903 PMID: 19000846 PMID: 18704691 PMID: 18465362 PMID: 18409001 PMID: 18226850 PMID: 18219529 PMID: 18172741 PMID: 18030529 PMID: 17924122 PMID: 17914103 PMID: 17701919 PMID: 17559371 PMID: 17397419 PMID: 17390024 PMID: 17311671 PMID: 17186167 PMID: 17164361 PMID: 16938722 PMID: 16877536 PMID: 16790036 PMID: 16790032 PMID: 16546242 PMID: 16435997 PMID: 15975186 PMID: 15952101 PMID: 15823719 PMID: 15668486 PMID: 15480982 PMID: 15301667 PMID: 15077571 PMID: 15069117 PMID: 14641066 PMID: 12750235 PMID: 12674437 PMID: 12540503 PMID: 10659641 PMID: 10433629 PMID: 9805955 PMID: 7881343 |
| Complement and coagulation cascades | PMID: 18078524 |
| Cytokine-cytokine receptor interaction | PMID: 18078524 |
| Cytosolic DNA-sensing pathway |  |
| Dilated cardiomyopathy | PMID: 21158358 PMID: 20977421 PMID: 20632954 PMID: 20208968 PMID: 19765477 PMID: 19729020 PMID: 19681866 PMID: 19440062 PMID: 19399964 PMID: 19397880 PMID: 19249488 PMID: 19091203 PMID: 18693776 PMID: 18655459 PMID: 18621940 PMID: 17940554 PMID: 17850632 PMID: 17514627 PMID: 17320219 PMID: 17298395 PMID: 17189427 PMID: 17096077 PMID: 16669965 PMID: 16585666 PMID: 16218427 PMID: 16000322 PMID: 15795345 PMID: 15660737 PMID: 15378803 PMID: 14634011 PMID: 12559622 PMID: 11941369 PMID: 11932410 PMID: 11901893 PMID: 11721723 PMID: 11418155 PMID: 11309529 PMID: 10993005 PMID: 10579734 PMID: 10432437 PMID: 9254324 PMID: 9238419 PMID: 9173705 PMID: 10352469 PMID: 8751254 PMID: 7926382 PMID: 8292308 PMID: 8390713 PMID: 1808940 |
| DNA replication | PMID: 19679549 PMID: 19491042 PMID: 19344660 PMID: 20707216 PMID: 19005641 PMID: 18847490 PMID: 18227995 PMID: 17639046 PMID: 17560613 PMID: 16791617 PMID: 16443874 PMID: 16037382 PMID: 15561953 PMID: 14988275 PMID: 12073009 PMID: 11861804 PMID: 11714744 PMID: 11067779 PMID: 10421666 PMID: 8521467 PMID: 8116045 PMID: 8276833 PMID: 1714602 PMID: 2280024 PMID: 2575478 PMID: 3262027 PMID: 3428598 PMID: 3299389 PMID: 3097892 PMID: 2933209 PMID: 3894121 PMID: 2986610 PMID: 6324221 |
| ECM-receptor interaction |  |
| Endocytosis | PMID: 20885388 PMID: 20421297 PMID: 20132771 PMID: 19644050 PMID: 19351151 PMID: 19143591 PMID: 19129372 PMID: 18552213 PMID: 18551197 PMID: 17639303 PMID: 17593905 PMID: 17551591 PMID: 17480204 PMID: 17264162 PMID: 16999216 PMID: 16929141 PMID: 16858541 PMID: 16794223 PMID: 16714477 PMID: 16377570 PMID: 16174284 PMID: 15817468 PMID: 15262490 PMID: 14678267 PMID: 12879249 PMID: 12621157 PMID: 12473645 PMID: 12128284 PMID: 11016456 PMID: 10623672 PMID: 9426315 PMID: 8995379 PMID: 8864419 PMID: 8674898 PMID: 8672532 PMID: 8764141 PMID: 8178053 PMID: 8244769 PMID: 8463286 PMID: 7678433 PMID: 2192846 PMID: 2900763 PMID: 6389224 PMID: 6991325 PMID: 4602673 |
| Epithelial cell signaling in Helicobacter pylori infection |  |
| ErbB signaling pathway |  |
| Ether lipid metabolism |  |
| Fc epsilon RI signaling pathway |  |
| Fc gamma R-mediated phagocytosis | PMID: 12562876 |
| Focal adhesion | PMID: 19741193 PMID: 19545622 PMID: 19503617 PMID: 17406055 PMID: 17369469 PMID: 15970590 PMID: 15866871 PMID: 15277383 PMID: 11809746 PMID: 9405068 |
| Folate biosynthesis |  |
| Fructose and mannose metabolism | PMID: 16161184 PMID: 1372573 PMID: 2108731 PMID: 322443 |
| Galactose metabolism | PMID: 9773720 |
| Gap junction | PMID: 19034495 PMID: 19000992 PMID: 16820374 PMID: 11483892 PMID: 6414863 |
| Glutathione metabolism | PMID: 1934175 |
| Glycerolipid metabolism | PMID: 19294363 PMID: 18606873 |
| Glycerophospholipid metabolism |  |
| Glycine, serine and threonine metabolism | PMID: 19095443 PMID: 1158036 |
| Glycolysis / Gluconeogenesis |  |
| Glycosylphosphatidylinositol(GPI)-anchor biosynthesis |  |
| Glyoxylate and dicarboxylate metabolism |  |
| GnRH signaling pathway |  |
| Graft-versus-host disease | PMID: 21190531 PMID: 19502956 PMID: 20428325 PMID: 18550852 PMID: 18294353 PMID: 15940051 PMID: 12503936 PMID: 12430895 PMID: 11468548 PMID: 9587393 PMID: 7525472 PMID: 8191569 PMID: 3309229 PMID: 3301484 PMID: 6986308 PMID: 136874 |
| Hedgehog signaling pathway |  |
| Hematopoietic cell lineage |  |
| Homologous recombination | PMID: 19934373 PMID: 18853578 PMID: 10856830 |
| Huntington's disease | PMID: 20542701 PMID: 20238082 PMID: 18450172 PMID: 18299198 PMID: 17052186 PMID: 12110243 PMID: 11704263 PMID: 8366714 |
| Hypertrophic cardiomyopathy (HCM) | PMID: 20632954 PMID: 15977098 |
| Inositol phosphate metabolism |  |
| Insulin signaling pathway | PMID: 21208058 PMID: 21136963 PMID: 21108831 PMID: 21033698 PMID: 20979575 PMID: 20815278 PMID: 20814956 PMID: 20649552 PMID: 20600772 PMID: 20554184 PMID: 20522973 PMID: 20493190 PMID: 20401454 PMID: 20090912 PMID: 19946718 PMID: 19929783 PMID: 19927140 PMID: 19826769 PMID: 19690174 PMID: 19647719 PMID: 19492410 PMID: 19485896 PMID: 19299910 PMID: 19292965 PMID: 19282820 PMID: 19261841 PMID: 19176946 PMID: 19118409 PMID: 19007436 PMID: 18984735 PMID: 18855718 PMID: 18854421 PMID: 18728222 PMID: 18633161 PMID: 18627032 PMID: 18516099 PMID: 18453752 PMID: 18288891 PMID: 18220643 PMID: 18192543 PMID: 18022395 PMID: 17914103 PMID: 17901049 PMID: 17761768 PMID: 17467106 PMID: 17437648 PMID: 17347799 PMID: 17242212 PMID: 17149545 PMID: 17062773 PMID: 17003352 PMID: 17003350 PMID: 16804078 PMID: 16622294 PMID: 16487789 PMID: 16449300 PMID: 16448741 PMID: 16436010 PMID: 16365106 PMID: 16311104 PMID: 16150913 PMID: 16125891 PMID: 15930959 PMID: 15905322 PMID: 15832492 PMID: 15590659 PMID: 20704943 PMID: 15504952 PMID: 15502249 PMID: 15240629 PMID: 15161747 PMID: 15078145 PMID: 15061645 PMID: 14769918 PMID: 14736545 PMID: 14683460 PMID: 14516196 PMID: 12807888 PMID: 12588884 PMID: 12477292 PMID: 12447984 PMID: 12006370 PMID: 11954667 PMID: 11872692 PMID: 11806463 PMID: 11795838 PMID: 11473054 PMID: 11390966 PMID: 11334414 PMID: 11042464 PMID: 11016459 PMID: 10842665 PMID: 10751417 PMID: 10665340 PMID: 10642598 PMID: 10446394 PMID: 10430617 PMID: 10426388 PMID: 10212843 PMID: 8386184 |
| Intestinal immune network for IgA production |  |
| Jak-STAT signaling pathway | PMID: 11960303 |
| Leukocyte transendothelial migration |  |
| Linoleic acid metabolism | PMID: 15166011 PMID: 9881236 |
| Long-term depression | PMID: 11908465 |
| Lysine degradation |  |
| Lysosome | PMID: 20392164 PMID: 19367387 PMID: 19087113 PMID: 18343263 PMID: 18329387 PMID: 10525662 PMID: 7043996 PMID: 162266 PMID: 761710 |
| MAPK signaling pathway | PMID: 20211236 PMID: 19689793 PMID: 19080172 PMID: 18764868 PMID: 11755918 |
| Melanogenesis | PMID: 15235770 |
| Methane metabolism |  |
| Mismatch repair | PMID: 1436502 |
| mTOR signaling pathway | PMID: 18342407 PMID: 17273556 |
| Natural killer cell mediated cytotoxicity |  |
| Neuroactive ligand-receptor interaction | PMID: 18078524 |
| Neurotrophin signaling pathway | PMID: 18191198 PMID: 12591159 |
| N-Glycan biosynthesis |  |
| NOD-like receptor signaling pathway |  |
| Non-homologous end-joining |  |
| Non-small cell lung cancer | PMID: 20722569 PMID: 19139117 PMID: 11716549 PMID: 9593699 |
| Notch signaling pathway | PMID: 17003479 PMID: 11272202 |
| Nucleotide excision repair |  |
| One carbon pool by folate |  |
| Oocyte meiosis |  |
| Other glycan degradation | PMID: 20813836 PMID: 20739398 PMID: 20720201 PMID: 20565994 PMID: 20501675 PMID: 20028967 PMID: 19902125 PMID: 19855071 PMID: 19727895 PMID: 19689250 PMID: 19615701 PMID: 19568704 PMID: 19502413 PMID: 19270448 PMID: 19156536 PMID: 19151107 PMID: 19137265 PMID: 19106327 PMID: 18651836 PMID: 18603822 PMID: 18566522 PMID: 18561209 PMID: 18541139 PMID: 18522808 PMID: 18335327 PMID: 18309462 PMID: 18301937 PMID: 18271277 PMID: 18058598 PMID: 18041436 PMID: 17951500 PMID: 17925502 PMID: 17804609 PMID: 17804583 PMID: 17563062 PMID: 17375400 PMID: 17306985 PMID: 17272392 PMID: 17263697 PMID: 17254607 PMID: 17148845 PMID: 17093946 PMID: 17054235 PMID: 16959366 PMID: 16936159 PMID: 16925670 PMID: 16877123 PMID: 16352671 PMID: 16140165 PMID: 15928243 PMID: 15708700 PMID: 15655244 PMID: 15577646 PMID: 15562253 PMID: 15526156 PMID: 15466941 PMID: 15287678 PMID: 15287677 PMID: 15242012 PMID: 15231693 PMID: 15206126 PMID: 15099203 PMID: 14978124 PMID: 14977582 PMID: 14747278 PMID: 14694834 PMID: 14674716 PMID: 14667179 PMID: 14624282 PMID: 14581156 PMID: 14513073 PMID: 13129452 PMID: 12913667 PMID: 12860582 PMID: 12828193 PMID: 12808881 PMID: 12766119 PMID: 12670620 PMID: 12648158 PMID: 12606498 PMID: 12401707 PMID: 12354173 PMID: 12145167 PMID: 11985000 PMID: 11809746 PMID: 11737853 PMID: 11554775 PMID: 11498024 PMID: 11377701 PMID: 11354252 PMID: 11350173 PMID: 11309391 PMID: 11289034 PMID: 11242714 PMID: 11172621 PMID: 11162573 PMID: 11161985 PMID: 11146312 PMID: 11118016 PMID: 11095432 PMID: 11087006 PMID: 10645038 PMID: 10512360 PMID: 10480189 PMID: 10436259 PMID: 10372241 PMID: 10361873 PMID: 10329986 PMID: 10102716 PMID: 9805641 PMID: 9568695 PMID: 8971586 PMID: 8835920 PMID: 8906894 PMID: 8952871 PMID: 8788435 PMID: 8529760 PMID: 7733656 PMID: 7848543 PMID: 8089146 PMID: 7988042 PMID: 8458616 PMID: 1323491 PMID: 1535403 PMID: 1835720 PMID: 1774015 PMID: 2226312 PMID: 2158410 PMID: 2156655 PMID: 2303431 PMID: 2745433 PMID: 3666887 PMID: 3525361 PMID: 6365944 PMID: 6319215 PMID: 6106098 PMID: 6257458 PMID: 523435 PMID: 387488 PMID: 461384 PMID: 175093 |
| Oxidative phosphorylation | PMID: 21195351 PMID: 21187013 PMID: 21079817 PMID: 21046015 PMID: 20964553 PMID: 20885388 PMID: 20833797 PMID: 20804614 PMID: 20819414 PMID: 20714348 PMID: 20674860 PMID: 20649552 PMID: 20600152 PMID: 20445742 PMID: 20410232 PMID: 20386866 PMID: 20217188 PMID: 20156986 PMID: 20144584 PMID: 20012595 PMID: 19966034 PMID: 19931409 PMID: 19903739 PMID: 19902174 PMID: 19887598 PMID: 19859074 PMID: 19796990 PMID: 19700791 PMID: 19595436 PMID: 19587359 PMID: 19553674 PMID: 19448711 PMID: 19448691 PMID: 19349201 PMID: 19332493 PMID: 19274082 PMID: 19197367 PMID: 19197047 PMID: 20707216 PMID: 19065050 PMID: 19056574 PMID: 18984658 PMID: 18846047 PMID: 18719883 PMID: 18687824 PMID: 18628517 PMID: 18625112 PMID: 18541051 PMID: 18560589 PMID: 18488190 PMID: 18456717 PMID: 18448125 PMID: 18433713 PMID: 18370748 PMID: 18349383 PMID: 18285554 PMID: 18279023 PMID: 18270681 PMID: 18248766 PMID: 18220945 PMID: 18220643 PMID: 18220630 PMID: 18220607 PMID: 18202132 PMID: 18194668 PMID: 18171433 PMID: 18162502 PMID: 18089950 PMID: 18078524 PMID: 18042831 PMID: 17981116 PMID: 17948130 PMID: 17909944 PMID: 17693571 PMID: 17660267 PMID: 17651698 PMID: 17563058 PMID: 17515919 PMID: 17512017 PMID: 17496364 PMID: 17485446 PMID: 17451836 PMID: 17416903 PMID: 17373958 PMID: 17334651 PMID: 17287462 PMID: 17269531 PMID: 17213881 PMID: 20161908 PMID: 17187250 PMID: 17184976 PMID: 17139575 PMID: 17052202 PMID: 17013887 PMID: 16899312 PMID: 16773565 PMID: 16731844 PMID: 16442843 PMID: 20021031 PMID: 16385762 PMID: 16326070 PMID: 16246967 PMID: 16173834 PMID: 16089501 PMID: 16082529 PMID: 15983313 PMID: 15983191 PMID: 15857235 PMID: 15793231 PMID: 15738989 PMID: 15734868 PMID: 15675189 PMID: 15662004 PMID: 15610050 PMID: 15576053 PMID: 15546003 PMID: 15545992 PMID: 15493637 PMID: 15257916 PMID: 15175861 PMID: 15175449 PMID: 15111510 PMID: 15100410 PMID: 14960743 PMID: 14694206 PMID: 14659011 PMID: 14607783 PMID: 14580754 PMID: 14502105 PMID: 12833045 PMID: 12832613 PMID: 12808136 PMID: 12637257 PMID: 12626321 PMID: 12606496 PMID: 12547248 PMID: 12391595 PMID: 12351431 PMID: 12020694 PMID: 11775531 PMID: 11748057 PMID: 11017142 PMID: 10783895 PMID: 8904921 PMID: 8899301 PMID: 7860167 PMID: 8366714 PMID: 8383698 PMID: 1579154 PMID: 1803023 PMID: 6752493 PMID: 6969524 PMID: 124319 |
| p53 signaling pathway |  |
| Pancreatic cancer | PMID: 21241523 PMID: 21129444 PMID: 21067267 PMID: 20961843 PMID: 20662478 PMID: 20571492 PMID: 20455996 PMID: 20388847 PMID: 20375642 PMID: 20183795 PMID: 20127570 PMID: 20119682 PMID: 19908334 PMID: 19890202 PMID: 19876688 PMID: 19844672 PMID: 19679549 PMID: 19608732 PMID: 19572116 PMID: 19470740 PMID: 19426140 PMID: 19286253 PMID: 19276450 PMID: 19190162 PMID: 19111249 PMID: 19085911 PMID: 19083105 PMID: 18995844 PMID: 18591845 PMID: 18268120 PMID: 18098271 PMID: 18067213 PMID: 17903450 PMID: 17897335 PMID: 17879406 PMID: 17584141 PMID: 17498513 PMID: 17241865 PMID: 17164367 PMID: 16644631 PMID: 16638847 PMID: 16225471 PMID: 16191762 PMID: 16172241 PMID: 16054982 PMID: 15823719 PMID: 15656235 PMID: 15577289 PMID: 15211110 PMID: 15077571 PMID: 14654569 PMID: 14613990 PMID: 12757407 PMID: 11953187 PMID: 11555835 PMID: 11025248 PMID: 10720068 PMID: 8647623 PMID: 8651908 PMID: 1946316 PMID: 1773717 PMID: 1688389 PMID: 2963553 |
| Parkinson's disease | PMID: 21222567 PMID: 21138275 PMID: 20977575 PMID: 20970452 PMID: 20878582 PMID: 20703363 PMID: 20702796 PMID: 20628041 PMID: 20542701 PMID: 20476794 PMID: 20186753 PMID: 20182043 PMID: 20039208 PMID: 19941173 PMID: 19810772 PMID: 19758471 PMID: 19769453 PMID: 19633703 PMID: 19614577 PMID: 19527717 PMID: 19394305 PMID: 19164583 PMID: 19021543 PMID: 18836752 PMID: 18805403 PMID: 18697612 PMID: 18599528 PMID: 18593082 PMID: 18486611 PMID: 18483616 PMID: 18457428 PMID: 18282481 PMID: 18261472 PMID: 18198460 PMID: 18194447 PMID: 19662137 PMID: 17980978 PMID: 17924651 PMID: 17701919 PMID: 17555416 PMID: 17493579 PMID: 17397638 PMID: 17251276 PMID: 17052186 PMID: 16997142 PMID: 16611073 PMID: 16507475 PMID: 16028004 PMID: 16026317 PMID: 15576053 PMID: 15501164 PMID: 12391595 PMID: 12375058 PMID: 12054433 PMID: 11456001 PMID: 11277472 PMID: 11106835 PMID: 8743979 PMID: 7788833 PMID: 8366714 PMID: 8426269 |
| Pathogenic Escherichia coli infection |  |
| Pathways in cancer |  |
| Pentose and glucuronate interconversions |  |
| Pentose phosphate pathway | PMID: 20711518 PMID: 20450981 PMID: 20188835 PMID: 19805580 PMID: 19230846 PMID: 18930068 PMID: 17125967 PMID: 15466941 PMID: 15067373 PMID: 12592403 PMID: 11756333 PMID: 9576750 PMID: 9417069 PMID: 9228023 PMID: 1579154 PMID: 1764469 PMID: 5810081 |
| Phenylalanine metabolism | PMID: 1443120 |
| Phosphatidylinositol signaling system | PMID: 19199708 |
| PPAR signaling pathway | PMID: 20346437 |
| Primary immunodeficiency |  |
| Prion diseases | PMID: 21129330 PMID: 20476794 PMID: 18755632 PMID: 18537546 PMID: 18483616 PMID: 17912358 PMID: 15533050 PMID: 15095987 PMID: 12110243 |
| Progesterone-mediated oocyte maturation |  |
| Prostate cancer | PMID: 21203859 PMID: 21148757 PMID: 21118287 PMID: 21094992 PMID: 20956995 PMID: 20924966 PMID: 20920685 PMID: 20702155 PMID: 20541662 PMID: 20535861 PMID: 20526366 PMID: 20518947 PMID: 20479278 PMID: 20406958 PMID: 20388847 PMID: 20309918 PMID: 20203524 PMID: 20153946 PMID: 20148361 PMID: 20142250 PMID: 20090828 PMID: 20063085 PMID: 19897022 PMID: 19863187 PMID: 19768259 PMID: 19616957 PMID: 19580824 PMID: 19572116 PMID: 19558691 PMID: 19534611 PMID: 19440546 PMID: 19439916 PMID: 19357973 PMID: 19197355 PMID: 19020265 PMID: 19066370 PMID: 19011290 PMID: 18824832 PMID: 18772488 PMID: 18768484 PMID: 18719369 PMID: 18704722 PMID: 18704691 PMID: 18696045 PMID: 18375048 PMID: 18368555 PMID: 18285837 PMID: 18276768 PMID: 18220653 PMID: 18042326 PMID: 18006554 PMID: 17897447 PMID: 17634714 PMID: 17603485 PMID: 17549824 PMID: 17498513 PMID: 17343664 PMID: 17265528 PMID: 16804532 PMID: 16546242 PMID: 16434606 PMID: 16299398 PMID: 16243513 PMID: 16193288 PMID: 15794122 PMID: 15621066 PMID: 15562386 PMID: 15562380 PMID: 15301667 PMID: 15167319 PMID: 15128610 PMID: 15032627 PMID: 12198000 PMID: 11901534 PMID: 11752200 PMID: 10984506 PMID: 10696282 PMID: 9951446 |
| Proteasome | PMID: 21070190 PMID: 20980462 PMID: 20885344 PMID: 20466847 PMID: 20364085 PMID: 20167927 PMID: 20069546 PMID: 19877155 PMID: 19833877 PMID: 19744947 PMID: 19732809 PMID: 19647043 PMID: 19616267 PMID: 19607692 PMID: 19587264 PMID: 19541767 PMID: 19390091 PMID: 19223597 PMID: 19165168 PMID: 19154729 PMID: 19144543 PMID: 19073451 PMID: 19007436 PMID: 19011169 PMID: 18812463 PMID: 18560589 PMID: 18551186 PMID: 18478125 PMID: 18451670 PMID: 18358479 PMID: 18321762 PMID: 18289831 PMID: 18053817 PMID: 17971205 PMID: 17698029 PMID: 17563070 PMID: 17555133 PMID: 17535269 PMID: 17516568 PMID: 17491658 PMID: 17205920 PMID: 17135326 PMID: 17130554 PMID: 17052186 PMID: 16954342 PMID: 16788801 PMID: 16675523 PMID: 16644676 PMID: 16596812 PMID: 16505224 PMID: 16415018 PMID: 16339897 PMID: 16335791 PMID: 16306379 PMID: 16030147 PMID: 15915823 PMID: 15910615 PMID: 15902099 PMID: 15848047 PMID: 15746249 PMID: 15737467 PMID: 15537654 PMID: 15078938 PMID: 15075914 PMID: 15036795 PMID: 15016802 PMID: 14664701 PMID: 14604833 PMID: 14584587 PMID: 12795417 PMID: 12759879 PMID: 12716748 PMID: 12672454 PMID: 12621037 PMID: 12547652 PMID: 12482142 PMID: 11793848 PMID: 11751962 PMID: 11717249 PMID: 11714744 PMID: 11467344 PMID: 11321039 PMID: 11272138 PMID: 11158874 PMID: 11140462 PMID: 11051286 PMID: 10973971 PMID: 10748014 PMID: 10681642 PMID: 10567588 PMID: 10438810 PMID: 10051633 PMID: 9793760 PMID: 9568691 PMID: 8878419 PMID: 8087098 |
| Purine metabolism | PMID: 19526867 PMID: 18326285 PMID: 10212666 |
| Pyrimidine metabolism |  |
| Pyruvate metabolism | PMID: 21187013 PMID: 18769905 PMID: 18728221 PMID: 16081739 PMID: 15073188 PMID: 14767867 PMID: 10909972 PMID: 4336555 |
| Regulation of actin cytoskeleton | PMID: 19251039 PMID: 18078524 PMID: 16682005 PMID: 15147333 PMID: 11996947 PMID: 11784718 PMID: 11563968 PMID: 11502567 PMID: 9006895 |
| Regulation of autophagy |  |
| Renal cell carcinoma | PMID: 21221210 PMID: 19401427 PMID: 18841151 PMID: 16567755 PMID: 12373491 PMID: 8635068 PMID: 7993128 PMID: 7518188 |
| Renin-angiotensin system | PMID: 21235695 PMID: 21234720 PMID: 21219847 PMID: 21208837 PMID: 21178817 PMID: 21166851 PMID: 21075694 PMID: 21055801 PMID: 20939795 PMID: 20939014 PMID: 20923486 PMID: 20923243 PMID: 20859806 PMID: 20809236 PMID: 20660994 PMID: 20660625 PMID: 20620720 PMID: 20592051 PMID: 20582734 PMID: 20580725 PMID: 20532698 PMID: 20499825 PMID: 20467188 PMID: 20440277 PMID: 20438446 PMID: 20424485 PMID: 20384386 PMID: 20339374 PMID: 20223228 PMID: 20215707 PMID: 20214262 PMID: 20182412 PMID: 20136508 PMID: 20099993 PMID: 20077598 PMID: 19940415 PMID: 19932519 PMID: 19884815 PMID: 19864959 PMID: 19817934 PMID: 19809363 PMID: 19791837 PMID: 19768259 PMID: 19768253 PMID: 19751467 PMID: 19738369 PMID: 19672111 PMID: 19630751 PMID: 19502253 PMID: 19491538 PMID: 19485896 PMID: 19479237 PMID: 19475778 PMID: 19475773 PMID: 19472100 PMID: 19465040 PMID: 19459520 PMID: 19436651 PMID: 19401427 PMID: 19392760 PMID: 19375596 PMID: 19363847 PMID: 19327134 PMID: 19286758 PMID: 19275680 PMID: 19247266 PMID: 19243623 PMID: 19227810 PMID: 19227807 PMID: 19220522 PMID: 19218356 PMID: 19194567 PMID: 19193728 PMID: 19164322 PMID: 19145003 PMID: 19114643 PMID: 19114589 PMID: 19029483 PMID: 19021699 PMID: 19012063 PMID: 18994672 PMID: 19001516 PMID: 18996825 PMID: 18983326 PMID: 18977550 PMID: 18947896 PMID: 18937992 PMID: 18855718 PMID: 18842316 PMID: 18841263 PMID: 18794817 PMID: 18753291 PMID: 18726873 PMID: 18716365 PMID: 18715217 PMID: 18710793 PMID: 18704745 PMID: 18689957 PMID: 18648151 PMID: 18580864 PMID: 18580863 PMID: 18625117 PMID: 18604370 PMID: 18593543 PMID: 18584585 PMID: 18584583 PMID: 18568313 PMID: 18564334 PMID: 18548089 PMID: 18525047 PMID: 18509209 PMID: 18494810 PMID: 18474174 PMID: 18469441 PMID: 18449380 PMID: 18454258 PMID: 18454253 PMID: 18450829 PMID: 18398815 PMID: 18359095 PMID: 18354383 PMID: 18334242 PMID: 18327991 PMID: 18326228 PMID: 18325305 PMID: 18316950 PMID: 18305124 PMID: 18264628 PMID: 18256393 PMID: 18248492 PMID: 18239590 PMID: 18220697 PMID: 18202667 PMID: 18199587 PMID: 18093407 PMID: 18096375 PMID: 18095912 PMID: 18078928 PMID: 18069999 PMID: 17922442 PMID: 18035185 PMID: 17978603 PMID: 17993725 PMID: 17978748 PMID: 17978595 PMID: 17978591 PMID: 17941464 PMID: 17936396 PMID: 17911338 PMID: 17762658 PMID: 17724226 PMID: 17702098 PMID: 17684464 PMID: 17682668 PMID: 17679833 PMID: 17679832 PMID: 17675900 PMID: 17674598 PMID: 17637271 PMID: 17630947 PMID: 17624939 PMID: 17607758 PMID: 17606856 PMID: 17601392 PMID: 17596525 PMID: 17586614 PMID: 17583170 PMID: 17581838 PMID: 17568278 PMID: 17564559 PMID: 17560613 PMID: 17547838 PMID: 17521525 PMID: 17516840 PMID: 17508916 PMID: 17508912 PMID: 17487296 PMID: 17487251 PMID: 17474500 PMID: 17461301 PMID: 17446534 PMID: 17418686 PMID: 17416265 PMID: 17398202 PMID: 17391169 PMID: 17379017 PMID: 17337492 PMID: 17333128 PMID: 17324119 PMID: 17321627 PMID: 17320999 PMID: 17295666 PMID: 17291601 PMID: 17257271 PMID: 17254516 PMID: 17241528 PMID: 17227234 PMID: 17214277 PMID: 17213573 PMID: 17209507 PMID: 17186681 PMID: 17170378 PMID: 17143591 PMID: 17139580 PMID: 17137404 PMID: 17130203 PMID: 17113349 PMID: 17110423 PMID: 17093063 PMID: 17091602 PMID: 17081084 PMID: 17075213 PMID: 17064808 PMID: 17058853 PMID: 17054027 PMID: 17048976 PMID: 17029631 PMID: 17020539 PMID: 17003295 PMID: 16999659 PMID: 16972730 PMID: 16959581 PMID: 16949519 PMID: 16932362 PMID: 16914072 PMID: 16895678 PMID: 16883325 PMID: 16879778 PMID: 16868143 PMID: 16855517 PMID: 16827595 PMID: 16789486 PMID: 16781084 PMID: 16770980 PMID: 16767297 PMID: 16761901 PMID: 16760545 PMID: 16723864 PMID: 16703216 PMID: 16672053 PMID: 16601575 PMID: 16601559 PMID: 16598556 PMID: 16572114 PMID: 16567826 PMID: 16565237 PMID: 16563947 PMID: 16563946 PMID: 16563944 PMID: 16563943 PMID: 16529551 PMID: 16509553 PMID: 16508590 PMID: 16508208 PMID: 16492203 PMID: 16471111 PMID: 16452552 PMID: 16444875 PMID: 16392299 PMID: 16378791 PMID: 16354940 PMID: 16340660 PMID: 16331123 PMID: 16331093 PMID: 16295047 PMID: 16290123 PMID: 16286578 PMID: 16273289 PMID: 16269993 PMID: 16269991 PMID: 16262560 PMID: 16249540 PMID: 16246214 PMID: 16232022 PMID: 16218882 PMID: 16208306 PMID: 16198140 PMID: 16188798 PMID: 16186402 PMID: 16165393 PMID: 16160726 PMID: 16160725 PMID: 16157071 PMID: 16133490 PMID: 16120823 PMID: 16112244 PMID: 16088848 PMID: 16086646 PMID: 16046901 PMID: 16035305 PMID: 16035298 PMID: 15963208 PMID: 15946912 PMID: 15942051 PMID: 15928338 PMID: 15918671 PMID: 15917336 PMID: 15913505 PMID: 15889697 PMID: 15880190 PMID: 15868115 PMID: 15853685 PMID: 15841036 PMID: 15830186 PMID: 15829899 PMID: 15821452 PMID: 15819371 PMID: 15799209 PMID: 15772526 PMID: 15765616 PMID: 15752922 PMID: 15736290 PMID: 15733008 PMID: 15716683 PMID: 15716329 PMID: 15715558 PMID: 15698420 PMID: 15694745 PMID: 15691626 PMID: 15671919 PMID: 15671918 PMID: 15617852 PMID: 15614015 PMID: 15587680 PMID: 15579515 PMID: 15544523 PMID: 15539011 PMID: 15536109 PMID: 15516153 PMID: 15500387 PMID: 15485765 PMID: 15451759 PMID: 15367829 PMID: 15346097 PMID: 15308993 PMID: 15257254 PMID: 15257186 PMID: 15242241 PMID: 15225108 PMID: 15201543 PMID: 15183078 PMID: 15173127 PMID: 15155118 PMID: 15149345 PMID: 15117108 PMID: 15090864 PMID: 15078862 PMID: 15073492 PMID: 15073491 PMID: 15047641 PMID: 15047614 PMID: 15025842 PMID: 15023892 PMID: 15017527 PMID: 14768849 PMID: 14766673 PMID: 14766373 PMID: 14744624 PMID: 14720103 PMID: 14694146 PMID: 14693989 PMID: 14684677 PMID: 14684676 PMID: 14684663 PMID: 14678876 PMID: 14678866 PMID: 14632699 PMID: 14625161 PMID: 14617233 PMID: 14610338 PMID: 12975597 PMID: 12969130 PMID: 12948434 PMID: 12948427 PMID: 12929472 PMID: 12882873 PMID: 12882847 PMID: 12877077 PMID: 12868322 PMID: 12819310 PMID: 12817909 PMID: 12817061 PMID: 12781906 PMID: 12769164 PMID: 12742799 PMID: 12724050 PMID: 12716844 PMID: 12693071 PMID: 12676168 PMID: 12675638 PMID: 12673778 PMID: 12671327 PMID: 12647281 PMID: 12643209 PMID: 12643207 PMID: 12643181 PMID: 12643159 PMID: 12642018 PMID: 12608526 PMID: 12563564 PMID: 12553490 PMID: 12477155 PMID: 12476891 PMID: 12466317 PMID: 12425206 PMID: 12418374 PMID: 12411725 PMID: 12411451 PMID: 12410857 PMID: 12358875 PMID: 12243375 PMID: 12233220 PMID: 12224817 PMID: 12224046 PMID: 12217258 PMID: 12182249 PMID: 12163426 PMID: 12140729 PMID: 12086929 PMID: 12032106 PMID: 12010090 PMID: 11991225 PMID: 11982815 PMID: 11979350 PMID: 11950631 PMID: 11930647 PMID: 11881118 PMID: 11822535 PMID: 11821641 PMID: 11776100 PMID: 11774217 PMID: 11772299 PMID: 11765585 PMID: 11711563 PMID: 11601118 PMID: 11601117 PMID: 11601112 PMID: 11576953 PMID: 11565519 PMID: 11565518 PMID: 11554775 PMID: 11498653 PMID: 11431175 PMID: 11427096 PMID: 11397648 PMID: 11369831 PMID: 11334766 PMID: 11294048 PMID: 11293231 PMID: 11293230 PMID: 11258145 PMID: 11213892 PMID: 11200871 PMID: 11158852 PMID: 11110735 PMID: 11060823 PMID: 10981168 PMID: 10862639 PMID: 10842655 PMID: 10798278 PMID: 10652041 PMID: 10585308 PMID: 10528643 PMID: 9678776 PMID: 9540028 PMID: 9407416 PMID: 9291199 PMID: 8971089 PMID: 8819510 PMID: 8518536 PMID: 2250149 PMID: 1972412 PMID: 3023151 PMID: 6131004 PMID: 7204578 PMID: 215821 |
| Ribosome | PMID: 20943765 PMID: 20804614 PMID: 20584901 PMID: 20220085 PMID: 19309774 PMID: 17079314 PMID: 1533230 |
| RIG-I-like receptor signaling pathway |  |
| RNA degradation | PMID: 19358092 |
| RNA polymerase | PMID: 21099283 PMID: 20943765 PMID: 20929976 PMID: 20570862 PMID: 20406885 PMID: 19737542 PMID: 19292868 PMID: 19264844 PMID: 18710473 PMID: 18688044 PMID: 16326070 PMID: 16206511 PMID: 15225313 PMID: 14988562 PMID: 10075593 PMID: 8636021 PMID: 2668290 |
| Small cell lung cancer | PMID: 20722569 PMID: 19139117 PMID: 11716549 PMID: 9593699 |
| SNARE interactions in vesicular transport | PMID: 17919184 |
| Sphingolipid metabolism | PMID: 19798445 PMID: 18801905 |
| Spliceosome |  |
| Systemic lupus erythematosus | PMID: 21189220 PMID: 21146219 PMID: 21084753 PMID: 21043003 PMID: 20972946 PMID: 20962850 PMID: 20946654 PMID: 20809911 PMID: 20704100 PMID: 20585820 PMID: 20406958 PMID: 20377061 PMID: 20374339 PMID: 19822041 PMID: 20016505 PMID: 19743458 PMID: 19643665 PMID: 19616319 PMID: 19595612 PMID: 19522557 PMID: 19519464 PMID: 19478036 PMID: 19275684 PMID: 19207938 PMID: 19162250 PMID: 20948646 PMID: 19083191 PMID: 19081854 PMID: 19075293 PMID: 19065799 PMID: 19028607 PMID: 18781962 PMID: 18771589 PMID: 18719369 PMID: 18714012 PMID: 18613843 PMID: 18603025 PMID: 18594491 PMID: 18508588 PMID: 18508507 PMID: 18500428 PMID: 18372362 PMID: 18270862 PMID: 18263783 PMID: 18221926 PMID: 18061654 PMID: 17924403 PMID: 17701919 PMID: 17641666 PMID: 17562353 PMID: 17360301 PMID: 17318274 PMID: 17237411 PMID: 17039177 PMID: 17002901 PMID: 16909327 PMID: 16855160 PMID: 19803968 PMID: 16804532 PMID: 16762598 PMID: 16639700 PMID: 16467750 PMID: 16425035 PMID: 16331857 PMID: 16288998 PMID: 16273769 PMID: 16218483 PMID: 16123497 PMID: 16054351 PMID: 16021278 PMID: 15991605 PMID: 15934099 PMID: 15910639 PMID: 15803926 PMID: 15564718 PMID: 15196997 PMID: 15184534 PMID: 15124249 PMID: 15027585 PMID: 15024932 PMID: 14625819 PMID: 14593902 PMID: 12960309 PMID: 12520821 PMID: 12430864 PMID: 12225387 PMID: 12223102 PMID: 11725293 PMID: 11602489 PMID: 11590194 PMID: 11484692 PMID: 11460976 PMID: 11334493 PMID: 11244051 PMID: 11237130 PMID: 11071967 PMID: 10778663 PMID: 10704418 PMID: 10648110 PMID: 10604234 PMID: 10595462 PMID: 10319267 PMID: 9856487 PMID: 9767451 PMID: 9669409 PMID: 9587393 PMID: 9558194 PMID: 9532339 PMID: 9285016 PMID: 9347876 PMID: 9161931 PMID: 9352601 PMID: 9002012 PMID: 7654061 PMID: 7851276 PMID: 7946894 PMID: 8072454 PMID: 7920083 PMID: 7858116 PMID: 8274234 PMID: 8255671 PMID: 8486790 PMID: 8248641 PMID: 1445452 PMID: 1727740 PMID: 1793205 PMID: 1750798 PMID: 1751309 PMID: 2055095 PMID: 2031154 PMID: 1696188 PMID: 2570594 PMID: 2664212 PMID: 2498774 PMID: 2848890 PMID: 2973422 PMID: 3263240 PMID: 2979815 PMID: 2839425 PMID: 2826058 PMID: 3495582 PMID: 3554539 PMID: 4017287 PMID: 3966755 PMID: 3916923 PMID: 3873410 PMID: 6085691 PMID: 6369977 PMID: 6668704 PMID: 6758201 PMID: 7156819 PMID: 6981833 PMID: 7235501 PMID: 6968086 PMID: 388639 PMID: 315113 PMID: 5361407 |
| T cell receptor signaling pathway |  |
| Taste transduction |  |
| Taurine and hypotaurine metabolism | PMID: 19239167 |
| Terpenoid backbone biosynthesis |  |
| TGF-beta signaling pathway | PMID: 19265200 PMID: 17622752 |
| Thyroid cancer | PMID: 21172028 PMID: 20738033 PMID: 20718682 PMID: 20517537 PMID: 20492683 PMID: 20305008 PMID: 20001715 PMID: 19079271 PMID: 18708169 PMID: 18179620 PMID: 16446424 PMID: 15695785 PMID: 15285001 PMID: 12592128 |
| Tight junction | PMID: 20693346 PMID: 20470857 PMID: 20346437 PMID: 19808897 PMID: 19254713 PMID: 19103180 PMID: 17039425 PMID: 15063759 PMID: 1707214 |
| Toll-like receptor signaling pathway |  |
| Tryptophan metabolism | PMID: 20943816 PMID: 6398613 PMID: 349901 PMID: 1131652 PMID: 1244098 PMID: 4560708 PMID: 14264725 |
| Type I diabetes mellitus | PMID: 20838752 PMID: 20455442 PMID: 20223594 PMID: 19957870 PMID: 19607951 PMID: 19261693 PMID: 18814430 PMID: 18809297 PMID: 18306698 PMID: 18254467 PMID: 18181226 PMID: 17426748 PMID: 16755119 PMID: 16635342 PMID: 16573556 PMID: 16441980 PMID: 16160411 PMID: 15789919 PMID: 15648963 PMID: 15589481 PMID: 15270855 PMID: 15123604 PMID: 12741355 PMID: 12680017 PMID: 12640102 PMID: 12637983 PMID: 12520821 PMID: 12469623 PMID: 12458886 PMID: 11819535 PMID: 11698422 PMID: 11441119 PMID: 11342558 PMID: 11049787 PMID: 10946998 PMID: 10907126 PMID: 10885155 PMID: 10761866 PMID: 10719679 PMID: 10670626 PMID: 10567588 PMID: 10459165 PMID: 10432174 PMID: 10358748 PMID: 10205747 PMID: 9589684 PMID: 9269908 PMID: 9201596 PMID: 9179756 PMID: 9377367 PMID: 9200026 PMID: 8922599 PMID: 8843258 PMID: 8949364 PMID: 8883311 PMID: 8768297 PMID: 8681772 PMID: 8685494 PMID: 8542099 PMID: 7796348 PMID: 7740039 PMID: 7800871 PMID: 8030444 PMID: 8301143 PMID: 8159480 PMID: 8143357 PMID: 8415518 PMID: 8361872 PMID: 8058658 PMID: 8058654 PMID: 1400876 PMID: 1595221 PMID: 1556273 PMID: 1727741 PMID: 1312561 PMID: 1762457 PMID: 1838564 PMID: 1799897 PMID: 1867879 PMID: 2265492 PMID: 2135645 PMID: 2252527 PMID: 1976653 PMID: 2397610 PMID: 2225153 PMID: 1696188 PMID: 2159034 PMID: 2369974 PMID: 2330358 PMID: 2188459 PMID: 2606174 PMID: 2671601 PMID: 2504577 PMID: 2513568 PMID: 2527507 PMID: 2854031 PMID: 3262666 PMID: 3064076 PMID: 3285671 PMID: 3146849 PMID: 3128878 PMID: 3223420 PMID: 3124343 PMID: 3075153 PMID: 3329227 PMID: 3125628 PMID: 3501924 PMID: 3309126 PMID: 3631074 PMID: 3502987 PMID: 3311781 PMID: 3111859 PMID: 3301605 PMID: 3296470 PMID: 3493414 PMID: 3601357 PMID: 3460915 PMID: 3460220 PMID: 3520389 PMID: 3516938 PMID: 3514327 PMID: 3461234 PMID: 3081208 PMID: 3456197 PMID: 4090971 PMID: 2999322 PMID: 3159965 PMID: 3907236 PMID: 3874341 PMID: 3888733 PMID: 2931586 PMID: 3898983 PMID: 3869086 PMID: 2413613 PMID: 6395625 PMID: 6334852 PMID: 6594040 PMID: 6433149 PMID: 6746903 PMID: 6333238 PMID: 6368290 PMID: 6363603 PMID: 6201223 PMID: 6134178 PMID: 6354781 PMID: 6333959 PMID: 6181576 PMID: 6818071 PMID: 7036337 PMID: 6764847 PMID: 7034534 PMID: 77236 |
| Type II diabetes mellitus | PMID: 21189693 PMID: 20879969 PMID: 20713144 PMID: 20476794 PMID: 20408053 PMID: 20400141 PMID: 20388246 PMID: 20092834 PMID: 20049302 PMID: 19822102 PMID: 19789948 PMID: 19395096 PMID: 19232230 PMID: 19162042 PMID: 19115606 PMID: 20428325 PMID: 19014008 PMID: 18991816 PMID: 18974575 PMID: 18937486 PMID: 18936798 PMID: 18814931 PMID: 18755632 PMID: 18726873 PMID: 18684421 PMID: 18627637 PMID: 18617965 PMID: 17936231 PMID: 17993259 PMID: 19885145 PMID: 17721698 PMID: 17704557 PMID: 17682309 PMID: 17662196 PMID: 17642450 PMID: 20535390 PMID: 17544131 PMID: 17473387 PMID: 17318769 PMID: 17277529 PMID: 17087190 PMID: 17073295 PMID: 17044598 PMID: 16807708 PMID: 16784405 PMID: 16712524 PMID: 16710267 PMID: 16647437 PMID: 16613735 PMID: 16557003 PMID: 16538969 PMID: 16408124 PMID: 16179727 PMID: 16160411 PMID: 16115720 PMID: 16115320 PMID: 15992403 PMID: 15956930 PMID: 15941146 PMID: 15936088 PMID: 15935339 PMID: 15855877 PMID: 15833293 PMID: 15772518 PMID: 15708541 PMID: 15707755 PMID: 15673357 PMID: 15618250 PMID: 15592481 PMID: 15554428 PMID: 15326564 PMID: 15138631 PMID: 15054439 PMID: 15006938 PMID: 14985776 PMID: 14704745 PMID: 14601482 PMID: 12870018 PMID: 12674176 PMID: 12651051 PMID: 12476894 PMID: 12454518 PMID: 12356630 PMID: 12090547 PMID: 12085419 PMID: 11928586 PMID: 11928585 PMID: 11876970 PMID: 11798874 PMID: 11561475 PMID: 11389877 PMID: 11285041 PMID: 11284486 PMID: 11250941 PMID: 11151219 PMID: 11117574 PMID: 10819243 PMID: 10692751 PMID: 10573728 PMID: 10440123 PMID: 10424314 PMID: 9882852 PMID: 9855387 PMID: 9714141 PMID: 9702383 PMID: 9642961 PMID: 9631887 PMID: 9543100 PMID: 9589946 PMID: 9516221 PMID: 9516220 PMID: 9291199 PMID: 9274899 PMID: 9214407 PMID: 9186883 PMID: 9158162 PMID: 9235072 PMID: 9296890 PMID: 8960075 PMID: 8902158 PMID: 8768534 PMID: 8637455 PMID: 9377371 PMID: 8782353 PMID: 9035838 PMID: 7589426 PMID: 8834126 PMID: 7474436 PMID: 7558466 PMID: 7753801 PMID: 7788833 PMID: 7483508 PMID: 7926425 PMID: 8413045 PMID: 8248641 PMID: 1436176 PMID: 1601393 PMID: 1563116 PMID: 1498529 PMID: 1545765 PMID: 1906584 PMID: 2087939 PMID: 2263755 PMID: 2103699 PMID: 2180210 PMID: 2367737 PMID: 2361594 PMID: 2330358 PMID: 2587885 PMID: 2636698 PMID: 2488772 PMID: 2568958 PMID: 2561256 PMID: 2528471 PMID: 2557805 PMID: 3072813 PMID: 3379125 PMID: 3669495 PMID: 3014383 PMID: 6538751 PMID: 6383778 PMID: 7051718 PMID: 7287908 |
| Tyrosine metabolism |  |
| Ubiquitin mediated proteolysis | PMID: 18078524 |
| Valine, leucine and isoleucine biosynthesis | PMID: 17914241 PMID: 17413098 PMID: 11436175 PMID: 9371476 PMID: 3090087 PMID: 6109479 PMID: 5032528 |
| Vascular smooth muscle contraction |  |
| VEGF signaling pathway | PMID: 18246670 PMID: 18067719 |
| Vibrio cholerae infection |  |
| Viral myocarditis | PMID: 20392896 PMID: 9107563 |
| Wnt signaling pathway | PMID: 20558665 PMID: 20217507 PMID: 20056134 PMID: 19577541 PMID: 19252133 PMID: 18599616 PMID: 18555673 PMID: 18541996 PMID: 18445358 PMID: 17671643 PMID: 17442272 PMID: 17259383 PMID: 16936217 PMID: 16415884 |
