# Supplementary material for: Disease Gene Interaction Pathways: A Potential Framework for How Disease Genes Associate by Disease-Risk Modules
Source: PLoS One. 2011 Sep 6;6(9):e24495. doi: 10.1371/journal.pone.0024495 (PMC3167857; doi:10.1371/journal.pone.0024495)
Supplement: Table S8 — PubMed ID in which gene pairs between interacting disease-risk terms have been proved to be correlated with T2D. (DOC) [file pone.0024495.s011.doc]

**Table S8. PubMed ID in which gene pairs between interacting disease-risk terms have been proved to be correlated with T2D.**

| **interacting disease risk terms pairs** | | **gene pairs between disease risk terms** | **PumMed ID that support the relationship between disease risk term pairs** |
| --- | --- | --- | --- |
|  | 6464 & 4938 |  |  |
|  | 6464 & 5062 |  |  |
|  | 6464 & 4958 |  |  |
|  | 6398 & 3237 |  |  |
|  | 6398 & 5742 |  |  |
|  | 6398 & 5928 | *PTEN* & *PTPN1* | PMID: 15998263 |
|  | 6398 & 4686 |  |  |
|  | 6398 & 5934 |  |  |
|  | 6398 & 5704 |  |  |
|  | 6398 & 5944 |  |  |
|  | 6398 & 5909 |  |  |
|  | 6383 & 5633 |  |  |
|  | 1280 & 3346 |  |  |
|  | 1280 & 4199 | *MET* & *HGF* | PMID: 17709889 |
|  | 1280 & 5742 |  |  |
|  | 1280 & 5414 |  |  |
|  | 1280 & 4568 |  |  |
|  | 1280 & 3511 |  |  |
|  | 1280 & 4686 |  |  |
|  | 1280 & 5975 | *MET* & *ACE* | PMID: 21235695 PMID: 20668156 PMID: 19445773 PMID: 15302632 PMID: 14693997 PMID: 12766114 |
|  | 6177 & 4945 |  |  |
|  | 6177 & 4199 |  |  |
|  | 6177 & 5576 |  |  |
|  | 6177 & 5928 | *CAV2* & *CAV1* | PMID: 16868246 |
|  | 6177 & 5475 |  |  |
|  | 4938 & 3351 | *AHSG* & *IRS1* | PMID: 18316360 |
|  | 4938 & 3378 |  |  |
|  | 4938 & 5401 |  |  |
|  | 4516 & 4231 |  |  |
|  | 4516 & 5148 | *ATF6* & *MAFA* | PMID: 18450959 |
|  | 4516 & 5708 |  |  |
|  | 4516 & 4958 |  |  |
|  | 2936 & 638 |  |  |
|  | 2936 & 4945 |  |  |
|  | 2936 & 5742 |  |  |
|  | 2936 & 5414 |  |  |
|  | 2936 & 5576 |  |  |
|  | 2936 & 5327 |  |  |
|  | 2936 & 6131 |  |  |
|  | 2936 & 6210 |  |  |
|  | 2936 & 6125 |  |  |
|  | 2936 & 4039 |  |  |
|  | 2936 & 5633 | *HLA-DQA1* & *MICA* | PMID: 16698430 PMID: 11118029 |
|  | 2936 & 5497 | *HLA-DQA1* & *HLA-DRB1* | PMID: 20603341 PMID: 19845915 PMID: 19143813 PMID: 17617163 PMID: 16254435 PMID: 15301863 PMID: 12595901 PMID: 12559622 PMID: 12373032 PMID: 11295472 PMID: 11118029 PMID: 10819246 PMID: 10053014 PMID: 9199707 PMID: 8950668 PMID: 8226139 |
|  |  | *HLA-DQA1* & *HLA-DOB* | PMID: 12595901 |
|  | 2936 & 5442 |  |  |
|  | 2936 & 4294 |  |  |
|  | 638 & 3237 | *ALOX5AP* & *AR* | PMID: 20592751 |
|  |  | *SHE* & *AR* | PMID: 10925727 |
|  |  | *MTHFR* & *AR* | PMID: 11583707 |
|  |  | *AKR1B1* & *AR* | PMID: 14582038 |
|  | 638 & 4231 |  |  |
|  | 638 & 4945 | *MVK* & *LDLR* | PMID: 18193043 |
|  |  | *KY* & *LDLR* | PMID: 20110440 |
|  | 638 & 5331 | *INDO* & *TNF* | PMID: 20972877 PMID: 18790831 |
|  |  | *GPX1* & *TNF* | PMID: 19325139 |
|  |  | *PI3* & *TNF* | PMID: 19026743 PMID: 10842657 PMID: 10542046 PMID: 10320053 PMID: 9312188 |
|  |  | *CTA-216E10.6* & *TNF* | PMID: 20495833 |
|  |  | *SMS* & *TNF* | PMID: 19959761 |
|  | 638 & 5414 |  |  |
|  | 638 & 5928 | *PI3* & *PTPN1* | PMID: 12169659 |
|  | 638 & 5690 | *ALOX5AP* & *IRAK1* | PMID: 19288030 |
|  | 638 & 5327 | *INDO* & *CDKN2A* | PMID: 20424228 |
|  |  | *PEPD* & *CDKN2A* | PMID: 19401414 |
|  | 638 & 3104 |  |  |
|  | 638 & 5337 | *GLIS3* & *CRY2* | PMID: 21103350 PMID: 20419449 PMID: 20081858 |
|  | 638 & 2437 | *WNT5B* & *NKX2-5* | PMID: 15754042 |
|  |  | *KIF6* & *PON1* | PMID: 21044781 |
|  |  | *ENTPD6* & *PYGB* | PMID: 19430760 |
|  | 638 & 5832 | *ALOX5AP* & *ALOX5* | PMID: 20592751 |
|  |  | *SOX13* & *SRY* | PMID: 10871192 |
|  | 638 & 6131 |  |  |
|  | 638 & 3511 | *PPP1R3B* & *PPP1R3A* | PMID: 16907705 |
|  | 638 & 5481 |  |  |
|  | 638 & 6125 | *GPD1* & *LPL* | PMID: 18448614 |
|  |  | *MVK* & *LPL* | PMID: 18193043 |
|  |  | *MVK* & *CETP* | PMID: 18193043 |
|  |  | *PI3* & *LPL* | PMID: 20693566 |
|  |  | *COX5B* & *LPL* | PMID: 20142634 |
|  |  | *SHE* & *LPL* | PMID: 16188578 PMID: 12927682 PMID: 12413775 |
|  | 638 & 5633 |  |  |
|  | 638 & 6093 |  |  |
|  | 638 & 5442 | *PI3* & *IDE* | PMID: 15033922 |
|  | 638 & 6282 |  |  |
|  | 638 & 5944 | *SHE* & *ACHE* | PMID: 15508343 |
|  | 638 & 5975 | *HAVCR1* & *ACE* | PMID: 20413516 |
|  |  | *PI3* & *ACE* | PMID: 18855718 PMID: 17170378 |
|  |  | *CARS* & *ACE* | PMID: 21127830 |
|  |  | *KIF6* & *ACE* | PMID: 21044781 |
|  |  | *SHE* & *ACE* | PMID: 20587399 |
|  |  | *MTHFR* & *ACE* | PMID: 20134102 PMID: 19857476 PMID: 15151261 |
|  |  | *AKR1B1* & *ACE* | PMID: 21127830 |
|  | 638 & 5864 |  |  |
|  | 638 & 4958 | *MVK* & *APOE* | PMID: 18193043 |
|  |  | *GPX1* & *APOE* | PMID: 19325139 PMID: 17420349 |
|  |  | *CARS* & *APOE* | PMID: 21127830 |
|  |  | *SHE* & *APOE* | PMID: 17173705 PMID: 15191354 |
|  |  | *MTHFR* & *APOE* | PMID: 19742390 PMID: 16862278 |
|  |  | *LSR* & *APOE* | PMID: 19188430 |
|  |  | *AKR1B1* & *APOE* | PMID: 21127830 |
|  |  | *ABCB4* & *APOE* | PMID: 18501722 |
|  | 638 & 5909 | *PGD* & *PGDS* | PMID: 20136655 PMID: 16367886 PMID: 14695455 |
|  | 638 & 6422 | *INDO* & *CA2* | PMID: 18790831 |
|  |  | *CACNA1G* & *CA2* | PMID: 21216827 |
|  | 638 & 6377 | *ETV5* & *KCTD15* | PMID: 20215397 |
|  | 638 & 5743 | *INDO* & *TCF7L2* | PMID: 20424228 PMID: 18706099 |
|  |  | *PEPD* & *TCF7L2* | PMID: 19401414 |
|  |  | *WNT5B* & *TCF7L2* | PMID: 18555673 |
|  |  | *GLIS3* & *TCF7L2* | PMID: 21103350 |
|  | 638 & 6361 | *KY* & *TERT* | PMID: 16556873 |
|  | 638 & 6396 |  |  |
|  | 638 & 6160 | *PI3* & *SLC2A4* | PMID: 19491300 PMID: 16373417 PMID: 16337244 PMID: 15223127 PMID: 10542046 |
|  |  | *SLC2A3* & *SLC2A4* | PMID: 19110659 PMID: 11334413 |
|  | 3754 & 4344 |  |  |
|  | 3754 & 5331 | *RIPK1* & *TNF* | PMID: 19432816 PMID: 12706864 |
|  |  | *TRADD* & *TNF* | PMID: 16424911 PMID: 15322087 |
|  |  | *CD40* & *TNF* | PMID: 19707732 PMID: 19621420 PMID: 19283894 PMID: 18509975 PMID: 18446238 PMID: 17519305 PMID: 16936191 PMID: 15690148 PMID: 11298122 PMID: 10798271 |
|  |  | *TNFRSF1B* & *TNF* | PMID: 11315843 |
|  | 3754 & 5742 | *RIPK1* & *FADD* | PMID: 19432816 PMID: 12706864 |
|  |  | *RIPK1* & *FAS* | PMID: 19432816 PMID: 12706864 |
|  |  | *TRADD* & *FADD* | PMID: 16424911 |
|  |  | *TRADD* & *FAS* | PMID: 16424911 |
|  |  | *CD40* & *FADD* | PMID: 18446238 |
|  |  | *CD40* & *FAS* | PMID: 18446238 PMID: 11872651 |
|  | 3754 & 5414 |  |  |
|  | 3754 & 5928 |  |  |
|  | 3754 & 5680 |  |  |
|  | 3754 & 6210 |  |  |
|  | 3754 & 5599 | *CD40* & *APCS* | PMID: 19922665 PMID: 15448088 PMID: 9780221 |
|  |  | *CD40* & *CRP* | PMID: 21084403 PMID: 19282610 PMID: 19132203 PMID: 16490430 PMID: 16412866 |
|  | 3754 & 3216 | *CD40* & *TNFRSF17* | PMID: 19336675 |
|  | 3754 & 5170 | *RIPK1* & *TNFRSF10B* | PMID: 19432816 PMID: 12706864 |
|  | 3237 & 3269 | *AR* & *VDR* | PMID: 12716975 PMID: 12520530 PMID: 10948206 |
|  |  | *AR* & *PPARG* | PMID: 16350721 PMID: 15774022 |
|  |  | *ESR1* & *PPARG* | PMID: 15774022 |
|  | 3237 & 4231 |  |  |
|  | 3237 & 3351 | *AR* & *IRS1* | PMID: 17046546 PMID: 16350721 PMID: 16339499 PMID: 10826514 |
|  |  | *AR* & *IRS2* | PMID: 10409618 |
|  |  | *AR* & *IGF1R* | PMID: 16350721 |
|  | 3237 & 5033 |  |  |
|  | 3237 & 4449 | *AR* & *INS* | PMID: 16350721 PMID: 10409121 |
|  |  | *AR* & *IGF1* | PMID: 16350721 |
|  | 3237 & 5742 | *AR* & *FAS* | PMID: 18622897 PMID: 15034081 PMID: 10548879 |
|  | 3237 & 5414 | *AR* & *BLM* | PMID: 17601993 |
|  | 3237 & 5062 | *AR* & *PPARGC1A* | PMID: 20371735 PMID: 18380005 |
|  | 3237 & 3505 |  |  |
|  | 3237 & 5006 |  |  |
|  | 3237 & 5928 | *AR* & *PTPN1* | PMID: 16505227 |
|  | 3237 & 4944 |  |  |
|  | 3237 & 5613 | *AR* & *AKT2* | PMID: 11809761 |
|  | 3237 & 5680 | *AR* & *IL6* | PMID: 15774022 |
|  |  | *ESR1* & *IL6* | PMID: 15774022 |
|  | 3237 & 5601 |  |  |
|  | 3237 & 2437 |  |  |
|  | 3237 & 5959 |  |  |
|  | 3237 & 5832 | *AR* & *ALOX5* | PMID: 20592751 |
|  | 3237 & 5275 |  |  |
|  | 3237 & 5833 | *AR* & *BTC* | PMID: 16869959 |
|  |  | *AR* & *FOXP3* | PMID: 20855871 |
|  |  | *AR* & *TBC1D4* | PMID: 18380005 |
|  |  | *AR* & *CAT* | PMID: 14515207 PMID: 11940333 |
|  | 3237 & 6210 |  |  |
|  | 3237 & 5204 |  |  |
|  | 3237 & 4186 | *AR* & *UCP2* | PMID: 16350721 PMID: 16075814 PMID: 15774022 PMID: 15061987 PMID: 12915397 |
|  |  | *AR* & *UCP3* | PMID: 16075814 |
|  |  | *ESR1* & *UCP2* | PMID: 15774022 |
|  | 3237 & 5148 |  |  |
|  | 3237 & 5442 |  |  |
|  | 3237 & 5975 | *AR* & *ACE* | PMID: 19825824 PMID: 18348167 PMID: 18239590 PMID: 16492203 |
|  | 3237 & 4958 |  |  |
|  | 3237 & 3216 |  |  |
|  | 3237 & 6404 | *AR* & *HNF1A* | PMID: 20581827 |
|  | 3237 & 6396 | *AR* & *XBP1* | PMID: 18544642 |
|  | 3237 & 5170 | *AR* & *ERN1* | PMID: 18544642 |
|  | 3237 & 5406 | *AR* & *HNRNPA1* | PMID: 12716975 |
|  | 3269 & 4231 |  |  |
|  | 3269 & 4634 | *PPARG* & *NEUROD1* | PMID: 17192490 |
|  |  | *PPARG* & *TCF4* | PMID: 20550665 |
|  | 3269 & 5033 | *PPARG* & *SREBF1* | PMID: 21178094 |
|  | 3269 & 4449 | *PPARA* & *INS* | PMID: 20972533 |
|  |  | *PPARG* & *INS* | PMID: 16350721 |
|  |  | *PPARG* & *IGF1* | PMID: 16350721 |
|  | 3269 & 5742 |  |  |
|  | 3269 & 5062 | *PPARA* & *PPARGC1A* | PMID: 18026715 PMID: 16896940 PMID: 16538490 |
|  |  | *PPARG* & *HNF4A* | PMID: 20079163 PMID: 18498634 PMID: 17601994 |
|  |  | *PPARG* & *PPARGC1A* | PMID: 19536736 PMID: 18996470 PMID: 18026715 PMID: 17342473 |
|  | 3269 & 5006 | *PPARA* & *PPARGC1B* | PMID: 16896940 |
|  |  | *NR1H2* & *NR1H3* | PMID: 21042792 PMID: 18949453 |
|  | 3269 & 5959 |  |  |
|  | 3269 & 5051 |  |  |
|  | 3269 & 4335 |  |  |
|  | 3269 & 5148 | *PPARG* & *MAF* | PMID: 20923526 PMID: 17903298 |
|  | 3269 & 5442 | *PPARG* & *IDE* | PMID: 20703447 PMID: 20548773 PMID: 19862325 PMID: 19592620 PMID: 19455305 PMID: 18984664 PMID: 18426861 |
|  | 3269 & 6282 |  |  |
|  | 3269 & 5975 | *VDR* & *ACE* | PMID: 20099993 |
|  |  | *RARA* & *ACE* | PMID: 10528828 |
|  |  | *PPARG* & *ACE* | PMID: 20099993 |
|  | 3269 & 4774 |  |  |
|  | 3269 & 6404 | *VDR* & *HNF1A* | PMID: 20099993 |
|  |  | *PPARG* & *HNF1A* | PMID: 20099993 PMID: 18498634 PMID: 17192490 PMID: 10843190 |
|  | 3269 & 6396 | *PPARA* & *NRF1* | PMID: 18026715 |
|  |  | *PPARG* & *NRF1* | PMID: 18678618 PMID: 18026715 |
|  | 4231 & 5033 |  |  |
|  | 4231 & 5414 |  |  |
|  | 4231 & 5327 |  |  |
|  | 4231 & 5725 |  |  |
|  | 4231 & 5051 |  |  |
|  | 4231 & 4883 |  |  |
|  | 4231 & 4335 |  |  |
|  | 4231 & 5148 |  |  |
|  | 4231 & 5442 |  |  |
|  | 4231 & 6404 |  |  |
|  | 4231 & 6091 | *MPG* & *CYBA* | PMID: 15479219 |
|  | 3346 & 3351 | *SOCS2* & *IRS1* | PMID: 20930063 |
|  |  | *SOCS2* & *IRS2* | PMID: 20930063 |
|  | 3346 & 5414 |  |  |
|  | 3346 & 5576 |  |  |
|  | 3346 & 4944 |  |  |
|  | 3346 & 5201 |  |  |
|  | 3346 & 5772 |  |  |
|  | 3346 & 5442 |  |  |
|  | 3346 & 4550 |  |  |
|  | 3351 & 4004 |  |  |
|  | 3351 & 4449 | *IRS1* & *INS* | PMID: 17914103 PMID: 16350721 PMID: 14693412 |
|  |  | *IRS1* & *IGF1* | PMID: 16350721 |
|  |  | *IRS2* & *INS* | PMID: 17914103 PMID: 16091421 |
|  |  | *IGF1R* & *INS* | PMID: 16350721 |
|  |  | *IGF1R* & *IGF1* | PMID: 19589910 PMID: 17476475 PMID: 17315038 PMID: 16350721 |
|  |  | *IGF1R* & *IGF2* | PMID: 19589910 PMID: 17476475 PMID: 17315038 |
|  |  | *INSR* & *INS* | PMID: 21239487 PMID: 20879971 PMID: 17914103 PMID: 14693412 PMID: 14551916 PMID: 12889122 |
|  |  | *INSR* & *IGF1* | PMID: 18828733 |
|  |  | *INSR* & *IGF2* | PMID: 20879971 |
|  | 3351 & 5742 |  |  |
|  | 3351 & 5414 |  |  |
|  | 3351 & 5576 |  |  |
|  | 3351 & 5928 | *IRS1* & *PTPN1* | PMID: 20515652 PMID: 18599621 PMID: 17545163 PMID: 17068137 PMID: 17008371 PMID: 14596593 PMID: 12502489 PMID: 10751417 |
|  |  | *IRS2* & *PTPN1* | PMID: 17068137 PMID: 12145151 |
|  |  | *INSR* & *PTPN1* | PMID: 21113646 |
|  | 3351 & 4568 | *IRS1* & *PTPRJ* | PMID: 17400802 |
|  |  | *IRS1* & *ACP1* | PMID: 17353188 |
|  | 3351 & 5680 | *IRS1* & *IL6* | PMID: 20930063 |
|  |  | *IRS1* & *ENPP1* | PMID: 19924153 |
|  |  | *IRS1* & *AGA* | PMID: 20930063 |
|  |  | *IRS2* & *IL6* | PMID: 20930063 |
|  |  | *IRS2* & *AGA* | PMID: 20930063 |
|  |  | *IGF1R* & *IL6* | PMID: 18249219 |
|  |  | *INSR* & *IL6* | PMID: 17180352 |
|  | 3351 & 5327 |  |  |
|  | 3351 & 3104 |  |  |
|  | 3351 & 4224 | *IGF1R* & *ADRB2* | PMID: 18249219 |
|  | 3351 & 5442 | *IGF1R* & *GHRL* | PMID: 18249219 |
|  |  | *INSR* & *IDE* | PMID: 19013138 |
|  | 3351 & 5499 |  |  |
|  | 3351 & 5931 | *IRS1* & *NOS3* | PMID: 12644458 |
|  |  | *IRS1* & *MTNR1B* | PMID: 21188353 |
|  |  | *IRS2* & *NOS3* | PMID: 12644458 |
|  | 3351 & 4550 | *IRS2* & *IFNG* | PMID: 15983045 |
|  | 3351 & 6361 | *IRS2* & *GCGR* | PMID: 21283589 |
|  | 3351 & 6160 | *IRS1* & *SLC2A4* | PMID: 20516259 PMID: 20089785 PMID: 19775880 PMID: 19764108 PMID: 19587264 PMID: 19567513 PMID: 19556298 PMID: 19035854 PMID: 17063325 PMID: 17038556 PMID: 17019595 PMID: 16755011 PMID: 15764607 PMID: 15322693 PMID: 14736545 PMID: 12882907 PMID: 12813916 PMID: 12807888 PMID: 12679424 PMID: 12606502 PMID: 12534369 PMID: 12388133 PMID: 12355825 PMID: 11563968 PMID: 11473054 PMID: 11238471 PMID: 11149909 PMID: 10542046 PMID: 10187855 PMID: 10078575 |
|  |  | *IRS2* & *SLC2A4* | PMID: 19764108 PMID: 17019595 PMID: 16755011 PMID: 12534369 PMID: 12355825 PMID: 11563968 PMID: 11238471 PMID: 11149909 |
|  |  | *INSR* & *SLC2A4* | PMID: 21113646 PMID: 15924147 |
|  | 4344 & 5742 |  |  |
|  | 4344 & 5928 | *PTPN22* & *PTPN1* | PMID: 20401454 PMID: 18310307 |
|  |  | *PTPN22* & *PTPN2* | PMID: 20401454 |
|  | 4344 & 4294 |  |  |
|  | 4634 & 5033 |  |  |
|  | 4634 & 5742 |  |  |
|  | 4634 & 5062 | *NEUROD1* & *HNF4A* | PMID: 19228875 PMID: 18811724 PMID: 17327436 PMID: 16936201 PMID: 16873704 PMID: 15883474 PMID: 11272211 |
|  |  | *NEUROD1* & *PPARGC1A* | PMID: 19520786 |
|  | 4634 & 5601 | *MYF5* & *PRDM16* | PMID: 19641492 |
|  | 4634 & 6019 |  |  |
|  | 4634 & 5337 | *ID2* & *CLOCK* | PMID: 16567517 |
|  | 4634 & 5051 |  |  |
|  | 4634 & 5148 | *NEUROD1* & *MAF* | PMID: 20184103 |
|  |  | *NEUROD1* & *MAFA* | PMID: 20184103 |
|  |  | *MYOG* & *PAX3* | PMID: 19121967 |
|  |  | *MYF5* & *PAX3* | PMID: 19121967 |
|  | 4634 & 5975 | *NEUROD1* & *PDX1* | PMID: 21060967 PMID: 19228875 |
|  | 4634 & 5864 |  |  |
|  | 4945 & 4199 |  |  |
|  | 4945 & 4449 | *APP* & *INS* | PMID: 18950899 |
|  |  | *LDLR* & *IGF1* | PMID: 18522411 |
|  | 4945 & 5928 |  |  |
|  | 4945 & 4391 | *APP* & *BACE1* | PMID: 19237574 PMID: 16186174 |
|  | 4945 & 4944 |  |  |
|  | 4945 & 5337 |  |  |
|  | 4945 & 2437 |  |  |
|  | 4945 & 5959 |  |  |
|  | 4945 & 5725 |  |  |
|  | 4945 & 5481 |  |  |
|  | 4945 & 4956 |  |  |
|  | 4945 & 4571 | *LDLR* & *CCL2* | PMID: 16634114 |
|  | 4945 & 6125 | *LDLR* & *LPL* | PMID: 18193043 |
|  |  | *LDLR* & *CETP* | PMID: 18193043 |
|  |  | *VLDLR* & *LPL* | PMID: 21087152 PMID: 18375436 |
|  | 4945 & 5934 |  |  |
|  | 4945 & 5600 | *LDLR* & *LIPC* | PMID: 18193043 |
|  |  | *LDLR* & *APOB* | PMID: 19273907 PMID: 19007590 PMID: 18346984 PMID: 18193043 PMID: 17872464 PMID: 16634114 PMID: 14514640 PMID: 12870170 PMID: 11352975 PMID: 9636203 PMID: 9409302 |
|  |  | *VLDLR* & *TG* | PMID: 18375436 |
|  | 4945 & 5442 | *APP* & *IDE* | PMID: 16186174 PMID: 15850385 |
|  |  | *LDLR* & *HK1* | PMID: 9166680 |
|  | 4945 & 5944 |  |  |
|  | 4945 & 4958 | *APP* & *APOE* | PMID: 9767914 |
|  |  | *LDLR* & *APOE* | PMID: 20229095 PMID: 18193043 PMID: 16634114 PMID: 11739102 PMID: 9409302 |
|  | 4945 & 6091 | *LRP1* & *NOX1* | PMID: 19818091 |
|  |  | *LDLR* & *NOX1* | PMID: 20489161 PMID: 20167927 |
|  |  | *LDLR* & *APOA1* | PMID: 18193043 |
|  | 4945 & 5406 |  |  |
|  | 4004 & 4907 |  |  |
|  | 4004 & 4199 |  |  |
|  | 4004 & 4696 |  |  |
|  | 4004 & 3378 |  |  |
|  | 4004 & 5331 |  |  |
|  | 4004 & 4391 |  |  |
|  | 4004 & 4944 |  |  |
|  | 4004 & 5401 |  |  |
|  | 4004 & 5316 |  |  |
|  | 4004 & 5934 |  |  |
|  | 4004 & 5497 | *HLA-DMB* & *HLA-DRB1* | PMID: 12391221 |
|  |  | *HLA-DRA* & *HLA-DRB1* | PMID: 19143813 |
|  | 4004 & 5944 |  |  |
|  | 4004 & 3847 |  |  |
|  | 4004 & 4294 | *CD63* & *CD36* | PMID: 15025679 |
|  | 4004 & 6396 |  |  |
|  | 4907 & 3378 |  |  |
|  | 4907 & 5414 |  |  |
|  | 4907 & 5576 |  |  |
|  | 4907 & 5928 |  |  |
|  | 4907 & 5292 |  |  |
|  | 4907 & 6210 |  |  |
|  | 4907 & 5497 |  |  |
|  | 4907 & 5975 |  |  |
|  | 4907 & 4294 |  |  |
|  | 4907 & 6396 |  |  |
|  | 4907 & 6160 |  |  |
|  | 4199 & 4449 |  |  |
|  | 4199 & 3378 | *TIMP2* & *MMP9* | PMID: 19581416 |
|  |  | *TIMP3* & *MMP9* | PMID: 19581416 |
|  |  | *TIMP4* & *MMP9* | PMID: 19581416 |
|  | 4199 & 5401 |  |  |
|  | 4199 & 5316 |  |  |
|  | 4199 & 5934 |  |  |
|  | 4199 & 5600 |  |  |
|  | 4199 & 6091 | *LCN2* & *RBP4* | PMID: 18710473 PMID: 18487287 |
|  | 4696 & 5742 | *TMPO* & *FAS* | PMID: 11272204 |
|  | 4696 & 5201 |  |  |
|  | 4696 & 5690 | *CD14* & *TLR2* | PMID: 20959532 PMID: 19210958 PMID: 19010563 |
|  |  | *CD14* & *TLR4* | PMID: 20959532 PMID: 19442172 PMID: 19210958 PMID: 19010563 |
|  | 4696 & 6131 |  |  |
|  | 4696 & 3511 |  |  |
|  | 4696 & 5934 | *CD14* & *MIP* | PMID: 17593905 |
|  | 4696 & 5708 |  |  |
|  | 4696 & 5944 |  |  |
|  | 4696 & 4294 | *CD14* & *CD36* | PMID: 17412916 PMID: 16313757 |
|  | 4696 & 6091 |  |  |
|  | 5033 & 5331 | *SREBF1* & *TNF* | PMID: 11916923 |
|  |  | *NFKB1* & *TNF* | PMID: 19696185 PMID: 19070859 |
|  |  | *RELA* & *TNF* | PMID: 18650421 PMID: 18322021 PMID: 18073321 PMID: 17079333 PMID: 16277639 PMID: 16002729 PMID: 15905055 PMID: 15364812 PMID: 15181049 PMID: 14744635 |
|  | 5033 & 5742 | *SREBF1* & *FAS* | PMID: 16597000 PMID: 14690455 |
|  | 5033 & 5062 | *SREBF1* & *PPARGC1A* | PMID: 20461355 PMID: 15834118 |
|  |  | *MEF2C* & *PPARGC1A* | PMID: 19065516 PMID: 18067759 PMID: 17680531 |
|  |  | *NR2F2* & *HNF4A* | PMID: 10331424 |
|  | 5033 & 5680 | *MEF2A* & *KLF4* | PMID: 12097321 |
|  |  | *NFKB1* & *IL6* | PMID: 20923958 |
|  |  | *RELA* & *IL6* | PMID: 19243600 |
|  | 5033 & 5201 |  |  |
|  | 5033 & 5327 |  |  |
|  | 5033 & 3104 |  |  |
|  | 5033 & 5959 |  |  |
|  | 5033 & 5725 |  |  |
|  | 5033 & 4335 |  |  |
|  | 5033 & 6093 |  |  |
|  | 5033 & 5442 | *NFKB1* & *IDE* | PMID: 20381502 |
|  | 5033 & 5975 |  |  |
|  | 5033 & 4458 | *NFKB1* & *RAGE* | PMID: 20381502 |
|  |  | *RELA* & *RAGE* | PMID: 19591173 PMID: 17427284 PMID: 15599399 PMID: 11723063 |
|  | 5033 & 3945 |  |  |
|  | 5033 & 6404 | *NR2F2* & *HNF1A* |  |
|  | 5033 & 6361 |  |  |
|  | 5033 & 6396 |  |  |
|  | 5033 & 5170 | *SREBF1* & *HIF1A* | PMID: 18448614 |
|  | 4449 & 4944 |  |  |
|  | 4449 & 3583 | *INS* & *HLA-DQB1* | PMID: 20805278 PMID: 18839133 PMID: 18310307 PMID: 17257313 |
|  |  | *INS* & *GCK* | PMID: 20879971 PMID: 20854389 PMID: 20531501 PMID: 7575335 |
|  |  | *INS* & *AK5* | PMID: 18243136 |
|  |  | *IGF1* & *GCK* | PMID: 20185807 PMID: 20081858 PMID: 18828733 |
|  |  | *IGF2* & *GCK* | PMID: 20879971 |
|  | 4449 & 5772 |  |  |
|  | 4449 & 4571 | *INS* & *CCL2* | PMID: 20872723 PMID: 16896941 PMID: 12540607 |
|  | 4449 & 3379 | *INS* & *CCL5* | PMID: 20872723 |
|  |  | *INS* & *CXCL10* | PMID: 20872723 |
|  | 4449 & 5316 |  |  |
|  | 4449 & 5600 | *INS* & *HSF1* | PMID: 20817212 |
|  |  | *INS* & *LIPC* | PMID: 15949705 |
|  |  | *INS* & *APOB* | PMID: 18590467 PMID: 16613269 |
|  |  | *INS* & *TG* | PMID: 21179733 PMID: 20459021 PMID: 19758361 PMID: 19729020 PMID: 19556421 PMID: 16613269 PMID: 16468376 PMID: 14749349 PMID: 12889122 PMID: 12133483 PMID: 10509601 PMID: 10222059 |
|  |  | *IGF2* & *TG* | PMID: 14749349 |
|  | 4449 & 5497 |  |  |
|  | 4449 & 5442 | *INS* & *IDE* | PMID: 20007922 |
|  | 4449 & 4294 | *INS* & *THY1* | PMID: 1686010 |
|  |  | *INS* & *CD36* | PMID: 16896941 PMID: 15282206 |
|  | 4449 & 6091 | *INS* & *DBP* | PMID: 14636794 |
|  |  | *INS* & *APOA1* | PMID: 16613269 |
|  |  | *INS* & *SAC* | PMID: 11147788 |
|  |  | *IGF2* & *SAC* | PMID: 11147788 |
|  | 4449 & 6160 |  |  |
|  | 3378 & 4391 |  |  |
|  | 3378 & 5833 |  |  |
|  | 3378 & 5772 |  |  |
|  | 3378 & 4571 | *MMP9* & *CCL2* | PMID: 19357773 |
|  | 3378 & 3379 | *MMP9* & *IL8* | PMID: 19357773 |
|  | 5331 & 4391 |  |  |
|  | 5331 & 2437 | *TNF* & *PON1* | PMID: 16803995 |
|  | 5331 & 6210 |  |  |
|  | 5331 & 6181 | *TNF* & *CD163* | PMID: 17593905 |
|  |  | *TNF* & *KCNJ11* | PMID: 17192490 |
|  | 5331 & 6248 | *TNF* & *SOD3* | PMID: 20594416 |
|  |  | *TNF* & *IL1B* | PMID: 16283249 PMID: 7605869 |
|  |  | *TNF* & *CAMP* | PMID: 20501675 PMID: 19819972 PMID: 8746558 |
|  | 5331 & 4550 | *TNF* & *IFNG* | PMID: 16292462 |
|  | 5742 & 4926 |  |  |
|  | 5742 & 5928 |  |  |
|  | 5742 & 5601 |  |  |
|  | 5742 & 5690 |  |  |
|  | 5742 & 5327 |  |  |
|  | 5742 & 3104 |  |  |
|  | 5742 & 2437 |  |  |
|  | 5742 & 5051 |  |  |
|  | 5742 & 4883 |  |  |
|  | 5742 & 4335 |  |  |
|  | 5742 & 4571 | *FAS* & *CCL2* | PMID: 19119030 |
|  | 5742 & 5316 |  |  |
|  | 5742 & 4186 | *FAS* & *UCP2* | PMID: 12127570 |
|  |  | *FAS* & *UCP3* | PMID: 12127570 |
|  | 5742 & 4039 |  |  |
|  | 5742 & 5633 |  |  |
|  | 5742 & 5704 |  |  |
|  | 5742 & 5600 | *FAS* & *TG* | PMID: 18622897 PMID: 15716584 PMID: 15479216 PMID: 11872664 |
|  | 5742 & 5475 | *FAS* & *ADM* | PMID: 19216096 |
|  | 5742 & 5497 |  |  |
|  | 5742 & 6181 |  |  |
|  | 5742 & 5864 | *FAS* & *ADD1* | PMID: 17369526 |
|  | 5742 & 3216 |  |  |
|  | 5742 & 6248 | *FAS* & *CAMP* | PMID: 19843871 PMID: 17563067 PMID: 16753576 |
|  | 5742 & 4746 |  |  |
|  | 5742 & 6404 |  |  |
|  | 5742 & 5743 |  |  |
|  | 5742 & 6361 | *FAS* & *FFAR1* | PMID: 16487789 |
|  | 5742 & 6091 | *FADD* & *SCARB1* | PMID: 19628574 |
|  |  | *FAS* & *SCARB1* | PMID: 19628574 |
|  | 5742 & 5170 | *FADD* & *TNFRSF10B* | PMID: 19432816 PMID: 12706864 |
|  |  | *FAS* & *TNFRSF10B* | PMID: 19432816 PMID: 12706864 |
|  | 5414 & 5062 |  |  |
|  | 5414 & 5327 |  |  |
|  | 5414 & 5051 |  |  |
|  | 5414 & 3511 |  |  |
|  | 5414 & 4335 |  |  |
|  | 5414 & 4186 |  |  |
|  | 5414 & 5599 |  |  |
|  | 5414 & 5600 |  |  |
|  | 5414 & 5148 |  |  |
|  | 5414 & 5975 |  |  |
|  | 5414 & 6422 |  |  |
|  | 5414 & 4550 |  |  |
|  | 5414 & 6361 |  |  |
|  | 5414 & 6160 |  |  |
|  | 5062 & 5006 | *PPARGC1A* & *PPARGC1B* | PMID: 20461355 PMID: 20175989 PMID: 19504086 PMID: 18385139 PMID: 16896940 PMID: 16326070 |
|  | 5062 & 4186 | *PPARGC1A* & *CRTC2* | PMID: 20093281 |
|  |  | *PPARGC1A* & *UCP2* | PMID: 17701054 |
|  |  | *NR0B2* & *UCP2* | PMID: 15525581 |
|  | 5062 & 5148 | *HNF4A* & *MAF* | PMID: 20878384 |
|  |  | *PPARGC1A* & *MAF* | PMID: 16755280 |
|  |  | *PPARGC1A* & *USF1* | PMID: 20461355 |
|  |  | *NR0B2* & *MAFA* | PMID: 18450959 PMID: 17259388 |
|  | 5062 & 5513 |  |  |
|  | 5062 & 5442 | *PPARGC1A* & *ESRRA* | PMID: 16755280 |
|  | 5062 & 6404 | *HNF4A* & *HNF1A* | PMID: 21270186 PMID: 21105491 PMID: 20531501 PMID: 20361036 PMID: 19929997 PMID: 19646184 PMID: 19565026 PMID: 19228875 PMID: 18498634 PMID: 18391435 PMID: 17407388 PMID: 17327436 PMID: 16936201 PMID: 14988562 PMID: 11315828 PMID: 11272211 PMID: 11232004 PMID: 11023148 PMID: 10946909 PMID: 10868855 PMID: 10690901 PMID: 10606640 PMID: 10417964 PMID: 10331424 PMID: 9839108 PMID: 9231664 PMID: 8945471 |
|  | 5062 & 6396 | *PPARGC1A* & *NRF1* | PMID: 18719883 PMID: 18026715 PMID: 16326070 |
|  | 3505 & 5006 |  |  |
|  | 3505 & 5680 |  |  |
|  | 3505 & 6404 | *HNF1B* & *HNF1A* | PMID: 20531501 PMID: 19228875 PMID: 18593771 PMID: 17327436 PMID: 16936201 PMID: 16380477 PMID: 14988562 PMID: 14598263 PMID: 14593617 PMID: 14575972 PMID: 12910053 PMID: 12832318 PMID: 12773136 PMID: 12726923 PMID: 12712243 PMID: 12605834 PMID: 12529398 PMID: 12453976 PMID: 12453961 PMID: 12442280 PMID: 11904435 PMID: 11904371 PMID: 11827432 PMID: 11806470 PMID: 11679424 PMID: 11414121 PMID: 11393552 PMID: 11315828 PMID: 11296466 PMID: 11293229 PMID: 11272211 PMID: 11232004 PMID: 11058894 PMID: 11023148 PMID: 11014387 PMID: 10951547 PMID: 10946909 PMID: 10944108 PMID: 10872540 PMID: 10868855 PMID: 10843190 PMID: 10840995 PMID: 10793009 PMID: 10754480 PMID: 10690959 PMID: 10690901 PMID: 10634821 PMID: 10606640 PMID: 10588527 PMID: 10581189 PMID: 10510948 PMID: 10417964 PMID: 10333942 PMID: 10331424 PMID: 10227025 PMID: 10097940 PMID: 10084598 PMID: 9840451 PMID: 9839108 PMID: 9733737 PMID: 9689059 PMID: 9685261 PMID: 9626139 PMID: 9621514 PMID: 9604876 PMID: 9231664 PMID: 8945471 PMID: 8945470 |
|  | 5006 & 6404 |  |  |
|  | 2869 & 5928 |  |  |
|  | 2869 & 5316 |  |  |
|  | 4926 & 5959 |  |  |
|  | 4926 & 4039 |  |  |
|  | 4926 & 5708 |  |  |
|  | 5576 & 5928 | *SORBS1* & *PTPN1* | PMID: 15181035 |
|  | 5576 & 4568 |  |  |
|  | 5576 & 5601 |  |  |
|  | 5576 & 5327 |  |  |
|  | 5576 & 5275 |  |  |
|  | 5576 & 5931 |  |  |
|  | 5928 & 5401 |  |  |
|  | 5928 & 5832 |  |  |
|  | 5928 & 5725 |  |  |
|  | 5928 & 5931 | *CAV1* & *CAV3* | PMID: 16868246 |
|  | 5928 & 4550 |  |  |
|  | 5928 & 6404 |  |  |
|  | 5928 & 6396 |  |  |
|  | 4391 & 5690 |  |  |
|  | 4391 & 4956 |  |  |
|  | 4391 & 5475 |  |  |
|  | 4391 & 6181 |  |  |
|  | 4391 & 3847 |  |  |
|  | 4391 & 5499 |  |  |
|  | 4944 & 5680 | *IL6R* & *IL6* | PMID: 14680981 |
|  | 4944 & 5204 |  |  |
|  | 4944 & 5316 |  |  |
|  | 4944 & 4958 |  |  |
|  | 4944 & 4550 |  |  |
|  | 4944 & 6396 |  |  |
|  | 5613 & 5959 |  |  |
|  | 5613 & 6377 |  |  |
|  | 5613 & 6223 | *APPL1* & *ADIPOR1* | PMID: 20536390 PMID: 18854421 |
|  | 5613 & 6160 | *AKT2* & *SLC2A4* | PMID: 15764607 |
|  | 4568 & 3847 |  |  |
|  | 4568 & 3945 |  |  |
|  | 5640 & 5204 |  |  |
|  | 5640 & 6396 |  |  |
|  | 5680 & 5327 |  |  |
|  | 5680 & 5833 |  |  |
|  | 5680 & 5148 | *ENPP1* & *MAF* | PMID: 18719658 |
|  | 5680 & 5975 | *AGA* & *ACE* | PMID: 20305326 |
|  | 5680 & 6404 | *IL6* & *HNF1A* | PMID: 17192490 |
|  |  | *ENPP1* & *HNF1A* | PMID: 18498634 PMID: 17192490 |
|  |  | *ZFAND6* & *HNF1A* | PMID: 21267535 |
|  | 5680 & 6396 | *ENPP1* & *NRF1* | PMID: 21283750 PMID: 18678618 |
|  | 5601 & 2437 |  |  |
|  | 5601 & 5599 | *ABCA1* & *CRP* | PMID: 16740417 |
|  | 5601 & 6093 |  |  |
|  | 5601 & 6181 |  |  |
|  | 5601 & 5975 | *INSC* & *COMT* | PMID: 18366720 |
|  | 5601 & 6248 |  |  |
|  | 5601 & 5931 |  |  |
|  | 5601 & 6091 | *ABCA1* & *APOA1* | PMID: 19644050 PMID: 18758514 PMID: 18193043 |
|  |  | *ABCA1* & *SCARB1* | PMID: 20212278 PMID: 19941833 PMID: 19644050 PMID: 18802933 PMID: 18640393 |
|  | 4445 & 3511 |  |  |
|  | 4445 & 4039 |  |  |
|  | 4445 & 5975 |  |  |
|  | 4445 & 5931 | *HMOX1* & *NOS3* | PMID: 20299332 PMID: 17906103 |
|  | 5201 & 6210 |  |  |
|  | 5201 & 6093 |  |  |
|  | 5201 & 5944 |  |  |
|  | 5690 & 3104 |  |  |
|  | 5690 & 5959 |  |  |
|  | 5690 & 5725 |  |  |
|  | 5690 & 3511 |  |  |
|  | 5690 & 5600 |  |  |
|  | 5690 & 5708 | *TLR2* & *RETN* | PMID: 17062773 |
|  |  | *TLR4* & *RETN* | PMID: 17062773 |
|  | 5690 & 6404 | *TLR2* & *IRF1* | PMID: 18669160 |
|  | 5690 & 6377 |  |  |
|  | 5690 & 4294 | *TLR4* & *CD36* | PMID: 19782984 PMID: 18818414 |
|  | 5401 & 6210 |  |  |
|  | 5401 & 6377 | *GRPR* & *GRP* | PMID: 15670577 PMID: 12021405 |
|  |  | *VIPR2* & *VIP* | PMID: 11978642 |
|  | 5401 & 4294 |  |  |
|  | 5327 & 2437 |  |  |
|  | 5327 & 5934 | *CDKN2A* & *SLC2A2* | PMID: 20142250 |
|  | 5327 & 5704 |  |  |
|  | 5327 & 6091 |  |  |
|  | 5327 & 5170 |  |  |
|  | 6019 & 3104 |  |  |
|  | 6019 & 2437 |  |  |
|  | 6019 & 6125 |  |  |
|  | 6019 & 6422 |  |  |
|  | 3104 & 5772 |  |  |
|  | 3104 & 6181 |  |  |
|  | 3104 & 5442 |  |  |
|  | 3104 & 6422 |  |  |
|  | 5337 & 2437 |  |  |
|  | 5337 & 5725 |  |  |
|  | 5337 & 4335 |  |  |
|  | 5337 & 5481 |  |  |
|  | 5337 & 5909 |  |  |
|  | 5337 & 6422 | *DIO2* & *UCP1* | PMID: 19690071 |
|  | 5337 & 6091 | *CLOCK* & *DBP* | PMID: 19387896 PMID: 18708447 PMID: 18624957 PMID: 16567517 PMID: 16166217 |
|  |  | *CRY2* & *DBP* | PMID: 16166217 |
|  | 2437 & 5959 |  |  |
|  | 2437 & 5975 | *PON1* & *ACE* | PMID: 21044781 |
|  | 2437 & 6091 | *PON1* & *CYBA* | PMID: 19407065 |
|  |  | *PON1* & *APOA1* | PMID: 19201174 PMID: 17525004 |
|  |  | *PON1* & *SCARB1* | PMID: 20724701 |
|  | 5959 & 6366 |  |  |
|  | 5959 & 5801 |  |  |
|  | 5959 & 6361 |  |  |
|  | 5832 & 5944 |  |  |
|  | 5292 & 5148 |  |  |
|  | 5292 & 5708 | *HHEX* & *WFS1* | PMID: 21153532 PMID: 20802253 PMID: 20703447 PMID: 20509872 PMID: 20215779 PMID: 20043853 PMID: 19862325 PMID: 19741467 PMID: 19502414 PMID: 19455305 PMID: 19258404 PMID: 19082521 PMID: 19020324 PMID: 18991055 PMID: 18984664 PMID: 18710364 PMID: 18597214 PMID: 18426861 |
|  | 5292 & 4746 |  |  |
|  | 5725 & 5475 |  |  |
|  | 5725 & 6248 |  |  |
|  | 5725 & 6396 |  |  |
|  | 6131 & 6248 |  |  |
|  | 6131 & 6366 |  |  |
|  | 6131 & 5801 |  |  |
|  | 5275 & 5599 |  |  |
|  | 5051 & 5513 |  |  |
|  | 3511 & 6282 |  |  |
|  | 5833 & 6210 |  |  |
|  | 5833 & 4186 |  |  |
|  | 5772 & 6210 |  |  |
|  | 5772 & 4571 | *CSF2* & *CCL2* | PMID: 19647751 |
|  | 5772 & 5316 |  |  |
|  | 5772 & 4958 |  |  |
|  | 5772 & 5801 |  |  |
|  | 5772 & 6091 |  |  |
|  | 6210 & 5204 |  |  |
|  | 6210 & 4186 |  |  |
|  | 6210 & 5475 |  |  |
|  | 6210 & 5497 |  |  |
|  | 6210 & 6181 |  |  |
|  | 6210 & 5801 |  |  |
|  | 6210 & 6091 |  |  |
|  | 6062 & 5148 |  |  |
|  | 6062 & 6091 | *APOA4* & *APOA1* | PMID: 18193043 PMID: 16781717 |
|  |  | *GM2A* & *HEXA* | PMID: 9223328 |
|  | 6062 & 6160 |  |  |
|  | 4883 & 5481 |  |  |
|  | 4883 & 5704 |  |  |
|  | 4883 & 5600 |  |  |
|  | 4883 & 5497 |  |  |
|  | 4883 & 6282 |  |  |
|  | 4883 & 5499 |  |  |
|  | 4883 & 6091 |  |  |
|  | 4335 & 5934 |  |  |
|  | 4335 & 6361 |  |  |
|  | 4335 & 6160 |  |  |
|  | 5481 & 6377 |  |  |
|  | 5481 & 6160 |  |  |
|  | 5204 & 6282 |  |  |
|  | 5204 & 6366 |  |  |
|  | 4956 & 4571 | *CP* & *PROC* | PMID: 1525154 |
|  | 4956 & 5934 |  |  |
|  | 4956 & 3216 |  |  |
|  | 4956 & 6248 | *CP* & *CAMP* | PMID: 11916944 |
|  | 4956 & 6377 | *FURIN* & *IAPP* | PMID: 15802374 PMID: 8557106 |
|  | 4571 & 5513 |  |  |
|  | 4571 & 3847 | *FGB* & *FGA* | PMID: 21258858 |
|  |  | *CCL2* & *VCAM1* | PMID: 19195861 |
|  |  | *CCL2* & *ICAM1* | PMID: 19195861 PMID: 17724226 |
|  | 3379 & 4686 |  |  |
|  | 5316 & 4224 | *AGT* & *AGTR1* | PMID: 19247266 PMID: 17143591 PMID: 11776100 |
|  | 5316 & 5442 |  |  |
|  | 5316 & 5944 |  |  |
|  | 5316 & 3847 |  |  |
|  | 5316 & 6377 |  |  |
|  | 5316 & 3622 |  |  |
|  | 4186 & 6422 | *UCP2* & *UCP1* | PMID: 17603293 PMID: 17447170 PMID: 17389766 PMID: 16968550 PMID: 16487034 PMID: 16075814 PMID: 15738989 PMID: 12127570 PMID: 11850613 |
|  |  | *UCP3* & *UCP1* | PMID: 17603293 PMID: 17447170 PMID: 17389766 PMID: 16968550 PMID: 16384603 PMID: 16075814 PMID: 15738989 PMID: 12127570 |
|  | 4186 & 5931 | *UCP2* & *NOS3* | PMID: 18556349 PMID: 15774022 |
|  | 6125 & 5931 | *LPL* & *NOS3* | PMID: 21193198 PMID: 20406163 |
|  | 6125 & 4774 |  |  |
|  | 6125 & 6377 |  |  |
|  | 6125 & 5743 |  |  |
|  | 6125 & 6091 | *LPL* & *APOA1* | PMID: 18193043 |
|  |  | *CETP* & *APOA1* | PMID: 18193043 PMID: 16399491 PMID: 15952120 PMID: 10523010 |
|  |  | *CETP* & *SCARB1* | PMID: 12957326 |
|  | 4224 & 4686 | *ADRB2* & *ADRB3* | PMID: 19576569 PMID: 18249219 |
|  | 4224 & 5975 | *ADRB2* & *ACE* | PMID: 21044781 |
|  |  | *AGTR1* & *ACE* | PMID: 19247266 PMID: 17143591 PMID: 15946912 PMID: 11776100 |
|  | 4224 & 6248 |  |  |
|  | 4224 & 5931 |  |  |
|  | 4224 & 6361 |  |  |
|  | 4686 & 5975 |  |  |
|  | 4686 & 6091 | *SSTR2* & *SSTR5* | PMID: 11230804 |
|  |  | *SSTR2* & *SST* | PMID: 17525126 |
|  |  | *SSTR2* & *SSTR1* | PMID: 11230804 |
|  |  | *ADRB3* & *APOA1* | PMID: 17727676 |
|  | 5934 & 5975 | *POLL* & *PDX1* | PMID: 16380477 |
|  |  | *SLC2A2* & *PDX1* | PMID: 19651901 |
|  | 5599 & 5600 | *APCS* & *TG* | PMID: 9780221 |
|  |  | *CRP* & *APOB* | PMID: 21129046 PMID: 20816677 PMID: 20019460 PMID: 19642911 PMID: 18264836 PMID: 20368963 PMID: 16963013 PMID: 16752182 PMID: 15220246 |
|  |  | *CRP* & *TG* | PMID: 21122635 PMID: 21092050 PMID: 21077943 PMID: 21070297 PMID: 20862801 PMID: 20720348 PMID: 20683173 PMID: 20580627 PMID: 20205104 PMID: 20075928 PMID: 19951570 PMID: 19737872 PMID: 19728847 PMID: 19642911 PMID: 19555253 PMID: 19436663 PMID: 19254210 PMID: 19227901 PMID: 19187587 PMID: 19075492 PMID: 18786739 PMID: 18492490 PMID: 18360022 PMID: 17917325 PMID: 17767051 PMID: 17426996 PMID: 17254464 PMID: 17239306 PMID: 17066589 PMID: 16536309 PMID: 16483174 PMID: 16377022 PMID: 16171015 PMID: 15505088 PMID: 15356091 PMID: 15207835 PMID: 15117561 PMID: 14694274 |
|  | 5599 & 6181 | *CRP* & *LDHA* | PMID: 21124955 |
|  | 5599 & 6319 |  |  |
|  | 5599 & 4294 | *TRPC5* & *TRPC6* | PMID: 20068131 PMID: 19135126 PMID: 18258814 |
|  |  | *TRPC4* & *TRPC6* | PMID: 19135126 PMID: 18258814 |
|  |  | *TRPC4* & *TRPM2* | PMID: 21290315 |
|  |  | *CRP* & *CD36* | PMID: 21262584 PMID: 20134099 |
|  | 5600 & 6093 | *LIPC* & *LCAT* | PMID: 11279518 |
|  |  | *APOB* & *PLTP* | PMID: 12401722 PMID: 11433351 |
|  |  | *APOB* & *LCAT* | PMID: 18283261 PMID: 17044598 |
|  |  | *TG* & *PLTP* | PMID: 10093000 |
|  |  | *TG* & *LCAT* | PMID: 9469511 PMID: 8139482 |
|  | 5600 & 6181 |  |  |
|  | 5600 & 6091 | *LIPC* & *APOA1* | PMID: 18193043 |
|  |  | *APOB* & *APOA1* | PMID: 20683626 PMID: 20631030 PMID: 20538481 PMID: 20213498 PMID: 19035165 PMID: 18991244 PMID: 18277343 PMID: 18193043 PMID: 17587723 PMID: 17525004 PMID: 17319473 PMID: 16922701 PMID: 16752182 PMID: 16613269 PMID: 16399491 PMID: 15952120 PMID: 15910557 PMID: 15780110 PMID: 14513073 PMID: 12401722 PMID: 11874951 PMID: 10868852 PMID: 10751747 PMID: 10523010 PMID: 9702430 PMID: 9237284 PMID: 8578698 PMID: 7500552 PMID: 7569454 PMID: 8171526 PMID: 8354314 PMID: 1289018 PMID: 1562163 PMID: 2137219 PMID: 2562831 PMID: 3181646 |
|  |  | *APOB* & *SAC* | PMID: 10744773 |
|  |  | *TG* & *RBP4* | PMID: 19671994 PMID: 19360015 PMID: 19282820 PMID: 19195734 PMID: 18937078 |
|  |  | *TG* & *DBP* | PMID: 21179727 PMID: 20075928 PMID: 20058619 PMID: 19484969 PMID: 19176866 PMID: 19125899 PMID: 19097668 PMID: 18849600 PMID: 18786739 PMID: 18618352 PMID: 18577787 PMID: 18360017 PMID: 17617283 PMID: 17373637 PMID: 17345784 PMID: 17296511 PMID: 16961271 PMID: 16572495 PMID: 16213608 PMID: 15495227 PMID: 15223224 PMID: 15019600 PMID: 14746741 PMID: 12860491 PMID: 12355867 PMID: 12225719 PMID: 11096265 PMID: 10390951 |
|  |  | *TG* & *APOA1* | PMID: 19035165 PMID: 17587723 PMID: 16781717 PMID: 16613269 PMID: 15952120 PMID: 8578698 PMID: 1562163 PMID: 2137219 |
|  |  | *TG* & *SST* | PMID: 15364160 |
|  | 5475 & 5497 |  |  |
|  | 5475 & 5513 |  |  |
|  | 5475 & 6093 |  |  |
|  | 5475 & 5909 |  |  |
|  | 5475 & 6377 |  |  |
|  | 5497 & 5931 |  |  |
|  | 5497 & 3622 |  |  |
|  | 5148 & 5975 | *MAFA* & *PDX1* | PMID: 20980260 |
|  | 5148 & 6396 |  |  |
|  | 5513 & 6181 | *MARK2* & *MARK4* | PMID: 15068958 |
|  | 5513 & 6319 |  |  |
|  | 6093 & 5975 |  |  |
|  | 6093 & 4958 | *PLTP* & *APOE* | PMID: 16644710 PMID: 11433351 |
|  | 6093 & 6377 | *ADCYAP1R1* & *ADCYAP1* | PMID: 15056942 |
|  |  | *VIPR1* & *VIP* | PMID: 11978642 |
|  | 6093 & 6361 |  |  |
|  | 6093 & 6091 | *HPR* & *RBP4* | PMID: 19482004 |
|  |  | *PLTP* & *APOA1* | PMID: 12401722 |
|  |  | *PLTP* & *SCARB1* | PMID: 11473048 |
|  | 6181 & 5442 | *KCNJ11* & *IDE* | PMID: 20703447 PMID: 20548773 PMID: 19862325 PMID: 19808892 PMID: 19592620 PMID: 19455305 PMID: 19082521 PMID: 18984664 PMID: 18719881 PMID: 18710364 PMID: 18426861 |
|  | 6181 & 5708 | *KCNJ11* & *RETN* | PMID: 18498634 |
|  |  | *KCNJ11* & *WFS1* | PMID: 21153532 PMID: 20802253 PMID: 20703447 PMID: 20361036 PMID: 20215779 PMID: 20043853 PMID: 19862325 PMID: 19502414 PMID: 19455305 PMID: 19082521 PMID: 19020324 PMID: 18984664 PMID: 18710364 PMID: 18597214 PMID: 18426861 |
|  |  | *ABCC8* & *RETN* | PMID: 18828733 |
|  | 6181 & 5864 |  |  |
|  | 6181 & 3622 | *KCNJ11* & *POMC* | PMID: 17728716 |
|  | 6181 & 6366 | *ABCC8* & *ENSA* | PMID: 14728986 |
|  | 6181 & 6361 |  |  |
|  | 5442 & 6282 | *AVP* & *AVPR2* | PMID: 19812297 |
|  | 5442 & 6319 |  |  |
|  | 5442 & 3622 |  |  |
|  | 5708 & 5975 |  |  |
|  | 5708 & 6248 | *WFS1* & *CAMP* | PMID: 12073007 |
|  | 6282 & 6404 |  |  |
|  | 6282 & 6160 |  |  |
|  | 5944 & 6091 | *HEXB* & *HEXA* | PMID: 9223328 PMID: 8896570 PMID: 8634145 PMID: 7550345 PMID: 7959736 |
|  | 6319 & 3001 | *LEP* & *LEPR* | PMID: 18828733 PMID: 18564365 PMID: 18490929 PMID: 16918594 |
|  | 6319 & 6091 |  |  |
|  | 6319 & 6160 |  |  |
|  | 5975 & 5864 | *ACE* & *ADD1* | PMID: 19247266 |
|  | 5975 & 5909 |  |  |
|  | 5975 & 6377 | *PDX1* & *IAPP* | PMID: 21099283 |
|  | 5975 & 6160 | *PDX1* & *SLC2A4* | PMID: 19864158 |
|  | 5864 & 4958 | *LIPE* & *APOE* | PMID: 14984467 |
|  | 5864 & 6361 |  |  |
|  | 5864 & 6160 | *ADD1* & *SLC2A4* | PMID: 15834118 |
|  | 4958 & 6377 | *APOE* & *IAPP* | PMID: 18243137 PMID: 12637985 |
|  | 4958 & 6091 | *APOE* & *CYBB* | PMID: 19325139 PMID: 17420349 |
|  |  | *APOE* & *APOA1* | PMID: 19644050 PMID: 18193043 PMID: 8354314 |
|  |  | *APOE* & *SCARB1* | PMID: 19644050 PMID: 12788901 PMID: 12069854 |
|  | 3847 & 6091 | *AMBP* & *DBP* | PMID: 8743518 |
|  | 6248 & 6091 | *CAMP* & *RBP4* | PMID: 20019678 PMID: 19389484 |
|  |  | *CAMP* & *DBP* | PMID: 18624957 |
|  | 5499 & 5406 |  |  |
|  | 5909 & 6396 |  |  |
|  | 4746 & 6366 |  |  |
|  | 4746 & 5170 |  |  |
|  | 6422 & 6366 |  |  |
|  | 6422 & 6396 |  |  |
|  | 4550 & 6091 |  |  |
|  | 6404 & 6396 |  |  |
|  | 6404 & 6091 |  |  |
|  | 6377 & 3622 | *VIP* & *NPY* | PMID: 17900700 PMID: 15582161 PMID: 11193191 PMID: 9934817 PMID: 7912158 PMID: 1943736 |
|  |  | *VIP* & *PYY* | PMID: 9934817 |
|  |  | *GRP* & *NPY* | PMID: 2905198 |
|  | 6377 & 6366 | *PCSK2* & *GPD2* | PMID: 9166680 |
|  | 6366 & 6396 |  |  |
|  | 6396 & 6091 |  |  |
